# Infliximab for pediatric patients with Crohn’s disease: A Phase 3, open-label, uncontrolled, multicenter trial in Japan

**DOI:** 10.1371/journal.pone.0201956

**Published:** 2018-08-16

**Authors:** Hitoshi Tajiri, Satoshi Motoya, Fukunori Kinjo, Atsuo Maemoto, Takayuki Matsumoto, Noriko Sato, Hiroshi Yamada, Mieko Nagano, Yutaka Susuta, Kunihiko Ozaki, Kazuoki Kondo, Toshifumi Hibi

**Affiliations:** 1 Department of Pediatrics, Osaka General Medical Center, Osaka, Japan; 2 Inflammatory Bowel Diseases Center, Sapporo-Kosei General Hospital, Sapporo, Japan; 3 Department of Endoscopy, University of the Ryukyus Hospital, Okinawa, Japan; 4 Inflammatory Bowel Disease Center, Sapporo Higashi Tokushukai Hospital, Sapporo, Japan; 5 Department of Medicine and Clinical Science, Graduate School of Medical Sciences, Kyushu University, Fukuoka, Japan; 6 Ikuyaku. Integrated Value Development Division, Mitsubishi Tanabe Pharma Corporation, Tokyo, Japan; 7 Center for Advanced IBD Research and Treatment, Kitasato Institute Hospital, Kitasato University, Tokyo, Japan; Kurume University School of Medicine, JAPAN

## Abstract

**Objectives:**

The prevalence of pediatric Crohn’s disease (CD) is increasing in Japan and other countries, and many patients are unresponsive to or do not tolerate current treatment options. This study aimed to investigate the efficacy, safety, and pharmacokinetic profile of infliximab (IFX) in pediatric patients with moderate-to-severe CD and inadequate response to existing treatment.

**Study design:**

This was an open-label, uncontrolled, multicenter Phase 3 study conducted at nine sites in Japan between April 2012 and March 2015. Pediatric patients (aged 6–17 years) with moderate-to-severe CD were treated with IFX 5 mg/kg at Weeks 0, 2, and 6, and at 8-week intervals thereafter until Week 46, with final evaluation at Week 54. IFX dose was increased to 10 mg/kg in patients who showed loss of response to IFX from Week 14 onwards.

**Results:**

A total of 14 patients fulfilled eligibility criteria and were treated. Dose-escalation criteria were met by five patients who then received 10 mg/kg IFX. The remaining nine patients continued to receive an IFX dose of 5 mg/kg. IFX rapidly improved clinical symptoms and its effect was maintained for up to 54 weeks. Overall Pediatric Crohn’s Disease Activity Index (PCDAI) response rate was 85.7%, and overall PCDAI remission rate was 64.3%. Three out of five patients who increased IFX dose regained PCDAI remission by retrieval of serum IFX concentration. Adverse events and serious adverse events occurred in 100.0% and 14.3% of patients, respectively. There was no substantial difference in the safety profiles of patients taking a constant dose of 5 mg/kg and those taking an increased dose of 10 mg/kg.

**Conclusions:**

These findings support the effective use of IFX in the treatment of pediatric patients with CD where other treatments have proven ineffective.

## Introduction

Crohn’s disease (CD) is an intractable inflammatory bowel condition of unknown cause and is increasing in incidence in Japan and other countries [1–6]. The incidence of pediatric CD is also increasing [7]. Malnutrition and hormonal imbalances associated with CD can have a serious impact during childhood, potentially impairing growth or delaying the onset of puberty [8–10]. Consequently, optimizing growth and improving quality of life (QOL) are key treatment goals for pediatric CD [11–13]. Current clinical practice guidelines for pediatric CD recommend nutrition therapy as first-line treatment; however, nutrition therapy causes issues with a high relapse rate and decrement in QOL [12,14]. Pharmacological treatment options include aminosalicylic acid preparations, corticosteroids, and immunomodulators, although many patients are unresponsive or unable to take these drugs due to side effects. Corticosteroids in particular cause issues with growth impairment and corticosteroid dependency [12].

Infliximab (IFX) is an anti-tumor necrosis factor-α (anti-TNF-α) monoclonal antibody that has been shown to be effective and well tolerated in the treatment of adult and pediatric CD in randomized controlled trials [15,16], and has been approved for the treatment of adult and pediatric CD outside of Japan. In Japan, IFX can be used to treat pediatric patients with CD based on results from a Phase 3 study of Japanese adults with CD [17]. However, in the absence of clinical trial data, it is unknown whether the efficacy and tolerability of IFX in Japanese pediatric patients with CD are similar to adult Japanese patients.

To determine the efficacy, safety, and pharmacokinetic (PK) profile of IFX in Japanese pediatric patients with moderate-to-severe CD and inadequate response to existing treatment, we have conducted a Phase 3 trial with a treatment protocol consisting of 5 mg/kg IFX administered at Weeks 0, 2, and 6, and at 8-week intervals thereafter, and a dose increase to 10 mg/kg if treatment became less effective. This study was the first Phase 3 study of IFX in Asian pediatric patients with CD.

## Methods

This was an open-label, uncontrolled, multicenter Phase 3 study conducted at nine sites in Japan between April 2012 and March 2015 in accordance with the ethical principles of the Declaration of Helsinki and Good Clinical Practice guidelines. All patients or their legal representatives provided written informed consents. Prior to the conduct of the study, the protocol was reviewed and approved by each and every institutional review board of participating institution at Sapporo-Kosei General Hospital, Tokushukai Group, Iwate Medical University, Saitama Children’s Medical Center, Yokohama City University Medical Center, Osaka City University Hospital, Kyushu University Hospital, Oita Red Cross Hospital, and University of the Ryukyus Hospital. The trial is registered at ClinicalTrials.gov (NCT01580670; https://clinicaltrials.gov/ct2/show/NCT01580670).

### Patients

All patients were children (aged 6–17 years) with moderate-to-severe CD and an inadequate response to existing therapies, as determined by Pediatric Crohn’s Disease Activity Index (PCDAI) [18] >30 at screening. The presence or absence of external fistulas was not required in the inclusion criteria. All patients had been diagnosed with CD at least 3 months prior to the start of the screening period. Patients on enteral nutrition were required to be on a stable dose for at least 2 weeks prior to the start of the screening period. Patients receiving azathioprine, 6-mercaptopurine, or methotrexate were required to have received treatment for at least 16 weeks and be on a stable dose for at least 8 weeks prior to the start of the screening period. Patients receiving corticosteroids were required to have received treatment for at least 8 weeks and be on a stable dose for at least 2 weeks prior to the start of the screening period. Patients receiving 5-aminosalicylates were required to have received treatment for at least 8 weeks and be on a stable dose for at least 4 weeks prior to the start of the screening period. Patients receiving metronidazole and ciprofloxacin were required to be on a stable dose for at least 2 weeks prior to the start of the screening period. Main exclusion criteria included patients with severe intestinal strictures, diagnosed short bowel syndrome, history of enterostomy, or those who had received previous treatment with IFX or other biologic products (such as anti-TNF-α agents or anti-interleukin-6 agents) or had been inoculated with a live vaccine in the previous 3 months. Patients who had received immunomodulators (excluding azathioprine, 6-mercaptopurine, and methotrexate) or injections of corticosteroids within 4 weeks of the screening period, or who were currently receiving these treatments, were excluded from participation. Concomitant or recent (within 4 weeks) use of total parenteral nutrition, fasting, surgery, cytapheresis, or blood transfusion was prohibited, as was the use of other investigational products.

### Study design

**Fig 1:**
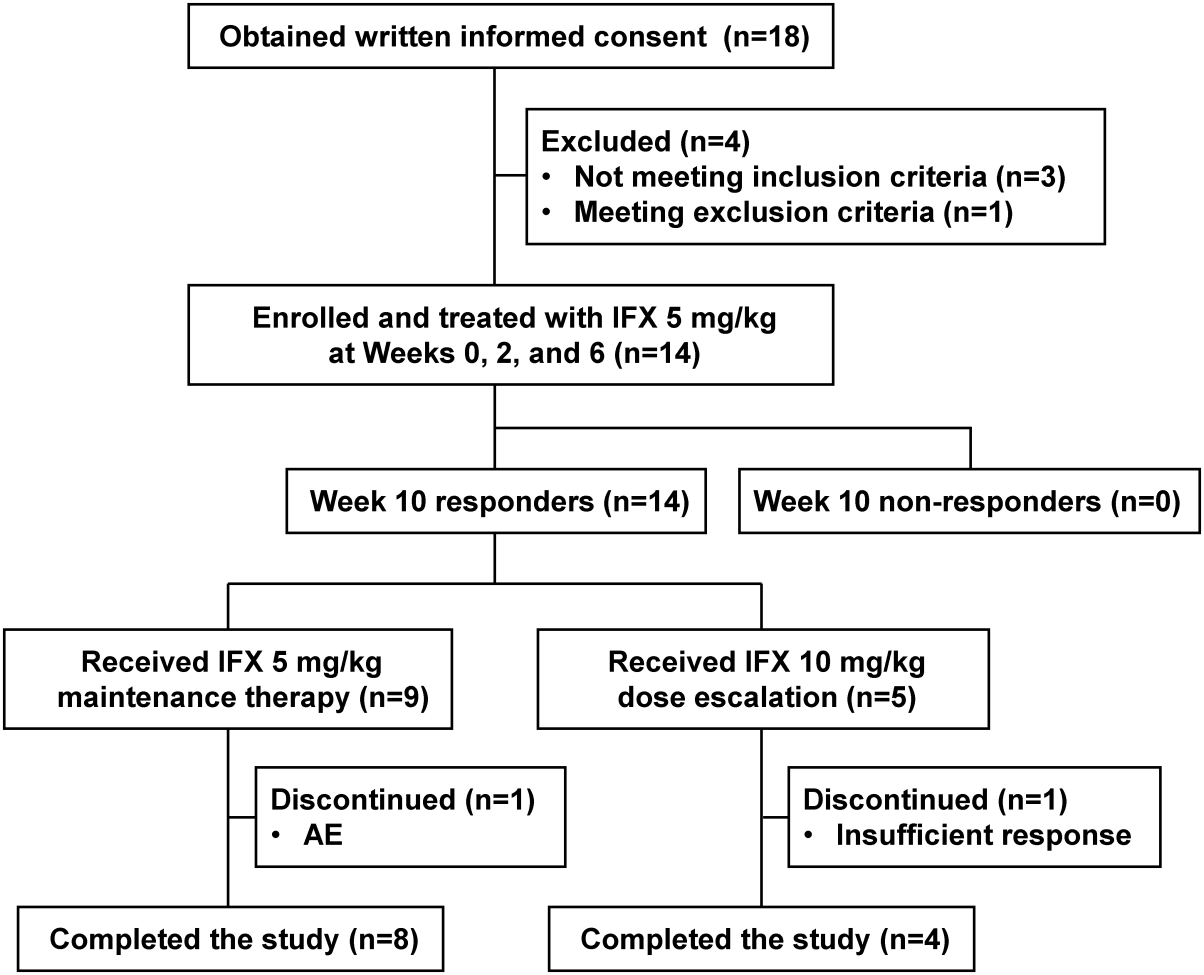
CONSORT flow chart of patient dispositions. AE, adverse event; IFX, infliximab.

**Fig 2:**
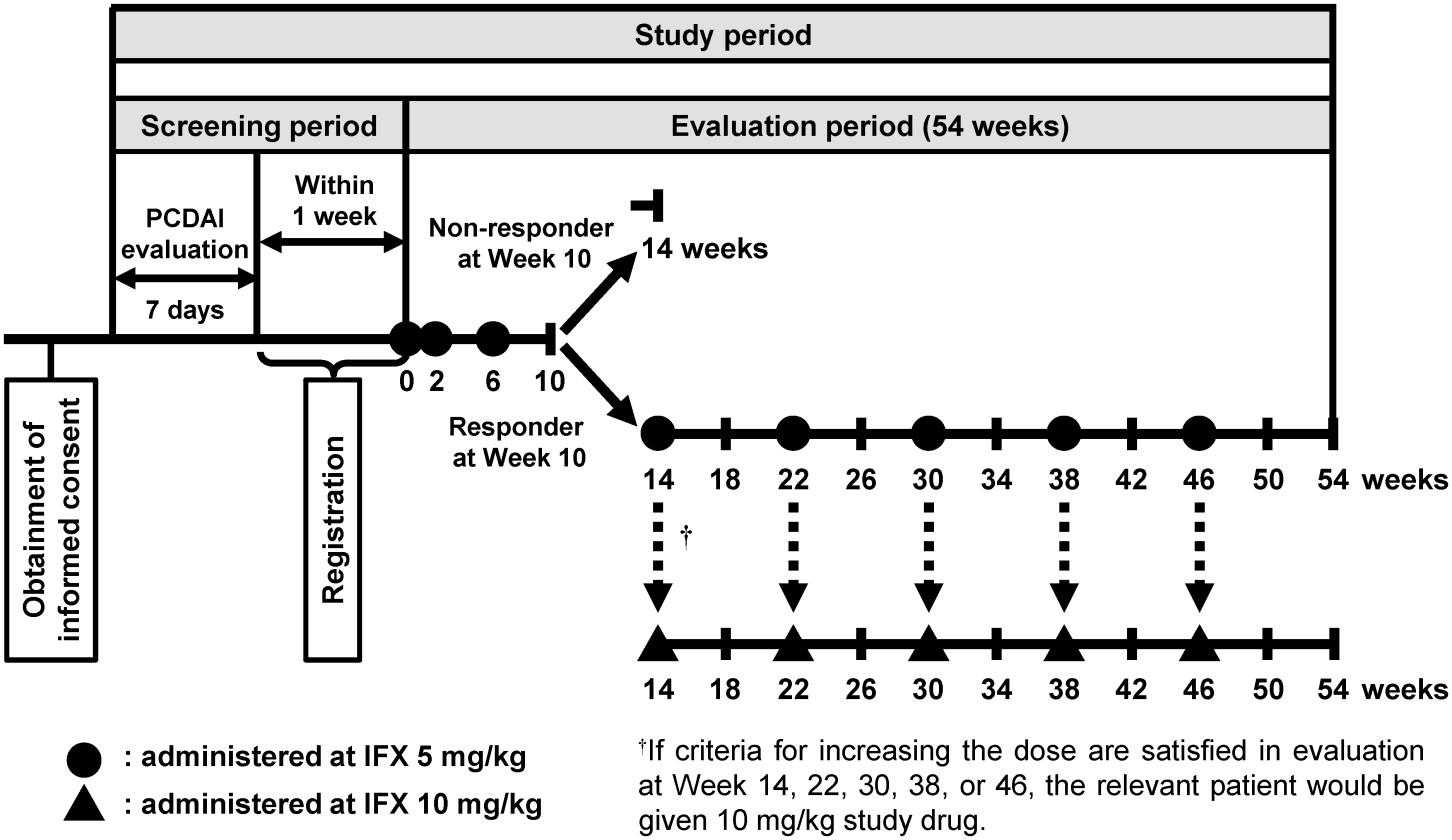
Overview of the study design. IFX, infliximab; PCDAI, Pediatric Crohn’s Disease Activity Index. Study period: period from the starting day of the screening period to the last day of the evaluation period. Screening period: period from the starting day of PCDAI evaluation to the start of the study period. Evaluation period: period from the start of IFX treatment to the evaluation day at Week 54. Evaluation period for non-responders at Week 10: the period from the start of IFX treatment to the end of evaluation at Week 14. Evaluation period for discontinued patients: the period from the start of IFX treatment to the end of evaluation after 8 weeks of the last treatment.

A summary of the patient disposition and study design are presented in Figs 1 and 2. Following initial PCDAI evaluation and determination of eligibility, all patients were administered 5 mg/kg IFX by intravenous infusion over a minimum 2-hour period. Treatment was repeated at Weeks 2 and 6, followed by administration at 8-week intervals until Week 46. Patients with PCDAI score ≤30 and a decrease in PCDAI score from baseline of ≥15 points were deemed to be responsive to IFX treatment. Any patients who did not show a PCDAI response at Weeks 2, 6, and 10 (Week 10 non-responders) were not administered IFX after Week 14. IFX dose was increased to 10 mg/kg in patients who showed inadequate responses to IFX after Week 14, as determined by either an increase in PCDAI score of ≥15 points compared with the lowest score observed at Weeks 2, 6, and 10, or a PCDAI score >30.

### Endpoints

Study endpoints included comprehensive outcome measures of efficacy, PK, and safety.

#### Efficacy endpoints

The efficacy outcome measures were median PCDAI score over time (Weeks 0, 2, 6, and at 4-week intervals until Week 54) and the percentages of patients in clinical response (decrease in PCDAI score of ≥15 points from baseline, with a total score of ≤30 points) and clinical remission (PCDAI score ≤10) calculated at Weeks 2, 6, 10, 14, 30, 54. PCDAI score was determined by medical interview, laboratory scores, and measurements of body weight and height on the day of evaluation [18]. Additional outcome measures included corticosteroid dose (equivalent to prednisolone) over time (Weeks 0, 2, 6, and at 4-week intervals until Week 54), median percentage change in corticosteroid dose at Weeks 2, 6, and 10 among patients using concomitant oral corticosteroids at baseline, and the percentage of patients who achieved corticosteroid withdrawal (corticosteroid dose of zero on evaluation day) during the study, median C-reactive protein (CRP) levels over time (Weeks 0, 2, 6, and at 4-week intervals until Week 54), number of draining fistulas at Weeks 10, 30, and 54, and the percentages of patients who achieved improvement of draining fistulas (improvement in number of draining fistulas by patient of ≥50%) and complete closure of draining fistulas at Weeks 10, 30, and 54.

#### Pharmacokinetics

Serum concentrations of IFX were recorded at Weeks 0, 2, and every 4 weeks until Week 54. Concentrations of IFX were measured by enzyme-linked immunosorbent assay using anti-IFX monoclonal antibodies from Janssen Biotech, Inc. (Horsham, PA, USA), with a detection limit of 0.10 μg/mL [19]. Serum concentrations of antibodies to IFX (ATI) were evaluated at Weeks 0, 14, and every 8 weeks until Week 54 using enzyme-linked immunosorbent assay [19]. Concentrations of IFX and ATI were measured at Mitsubishi Tanabe Pharma (Osaka, Japan).

#### Safety

Adverse events (AEs) and adverse drug reactions (ADRs) were classified according to the Medical Dictionary for Regulatory Activities version 17.1 and documented until Week 54.

### Statistical analysis

The number of patients able to participate in this study was limited, as moderate-to-severe pediatric CD is a rare condition with an assumed indication of around 250 patients in Japan when this study was planned. Furthermore, some of these patients may have been previously treated with IFX and would not, therefore, be eligible for inclusion. Consequently, a sample size of 20 patients was considered to be reasonable and feasible. This number of patients was deemed sufficient to detect the occurrence of ADRs, assuming that they occurred at the same frequency as in adults. Given the potentially serious impact of CD on growth and development, no control group was included in the study design.

The efficacy analysis set was defined as the full analysis set, consisting of patients who received study drug and evaluated efficacy at least once. Tabulation of patient data was performed for each time point, both with and without imputation of missing values. Missing values were added using the last observation carried forward method and were shown as Week 54 (overall). For patients who received an increased dose of 10 mg/kg IFX after Week 14, the time of dose increase was considered as Week 0. PCDAI score, PCDAI response rate, and PCDAI remission rate were determined at Weeks -8, -4, and 0, then every 4 weeks thereafter. PCDAI response rates for each time point were calculated based on the current observation period. All patients who received IFX treatment with available safety data were included in the safety analysis set. The PK analysis set consisted of patients receiving at least one dose of IFX and from whom serum IFX or ATI data were obtained on at least one occasion.

A subgroup analysis was conducted for trough IFX concentration among patients with or without PCDAI remission at Weeks 30 and 54. An additional post-hoc analysis of trough IFX concentration among patients with or without PCDAI remission was conducted at Week 14. We computed the two-sided 95% confidence interval [CI] for a proportion in one sample by means of F-distribution, which means Clopper-Pearson (Exact). The 95% CI in 100 person years incident rate were calculated based on Poisson distribution.

## Results

### Patients

In total, 18 patients provided informed consent, of whom 14 completed registration and fulfilled the eligibility criteria. Patient characteristics are summarized in Table 1. Of those participating, 11 patients were male and 13 patients were 12–17 years old. CD type was classified as ileum in two patients and ileocolon in 12 patients. The median PCDAI (interquartile range [IQR]) at baseline was 35.00 (35.00, 35.00). None of the patients who received 5 mg/kg IFX treatment at Weeks 0, 2, and 6 were Week 10 non-responders. Dose-escalation criteria were met by five patients who then started 10 mg/kg IFX treatment at Week 14 (n = 1), Week 30 (n = 1), Week 38 (n = 1), and Week 46 (n = 2). The nine patients in whom dose-escalation criteria were not met continued to receive 5 mg/kg IFX during the study. One patient in the 5 mg/kg group was identified with surgical indication for fistulas and discontinued treatment (Fig 1). This discontinuation was judged as due to an AE (worsening CD). One patient in the 10 mg/kg group discontinued the study due to insufficient response.

**Table 1:** Patient characteristics (FAS population).

	n = 14
Sex	
Male, n (%)	11 (78.6)
Female, n (%)	3 (21.4)
Age (years), median (IQR)	15.0 (14.0, 15.0)
6 to <12 years, n (%)	1 (7.1)
12 to 17 years, n (%)	13 (92.9)
Weight (kg), median (IQR)	48.68 (41.40, 52.60)
Height (cm), median (IQR)	165.0 (153.0, 172.0)
BMI (kg/m^2^), median (IQR)	18.40 (17.19, 20.72)
Disease duration (years), median (IQR)	0.80 (0.60, 1.30)
Type of disease	
Ileum, n (%)	2 (14.3)
Colon, n (%)	0 (0.0)
Ileocolon, n (%)	12 (85.7)
Concomitant medications at baseline	
Corticosteroids, n (%)	3 (21.4)
6-MP/azathioprine/methotrexate, n (%)	3 (21.4)
Aminosalicylates, n (%)	11 (78.6)
Metronidazole/ciprofloxacin, n (%)	1 (7.1)
Enteral nutrition, n (%)	6 (42.9)
PCDAI, median (IQR)	35.00 (35.00, 35.00)
CRP (mg/dL), median (IQR)	2.25 (0.70, 4.30)
TNF-α (pg/mL), median (IQR)^a^	1.265 (0.000, 1.720)

^a^Plasma concentration of TNF-α was measured using a chemiluminescence enzyme immunoassay only at baseline, because accurate measurement is not possible in the presence of IFX. The measured plasma concentration of TNF-α under the detection limit (0.55 pg/mL) was defined as 0.00 pg/mL.

6-MP, 6-mercaptopurine; BMI, body mass index; CRP, C-reactive protein; FAS, full analysis set; IQR, interquartile range; PCDAI, Pediatric Crohn’s Disease Activity Index; TNF-α, tumor necrosis factor-α.

### Efficacy

**Fig 3:**
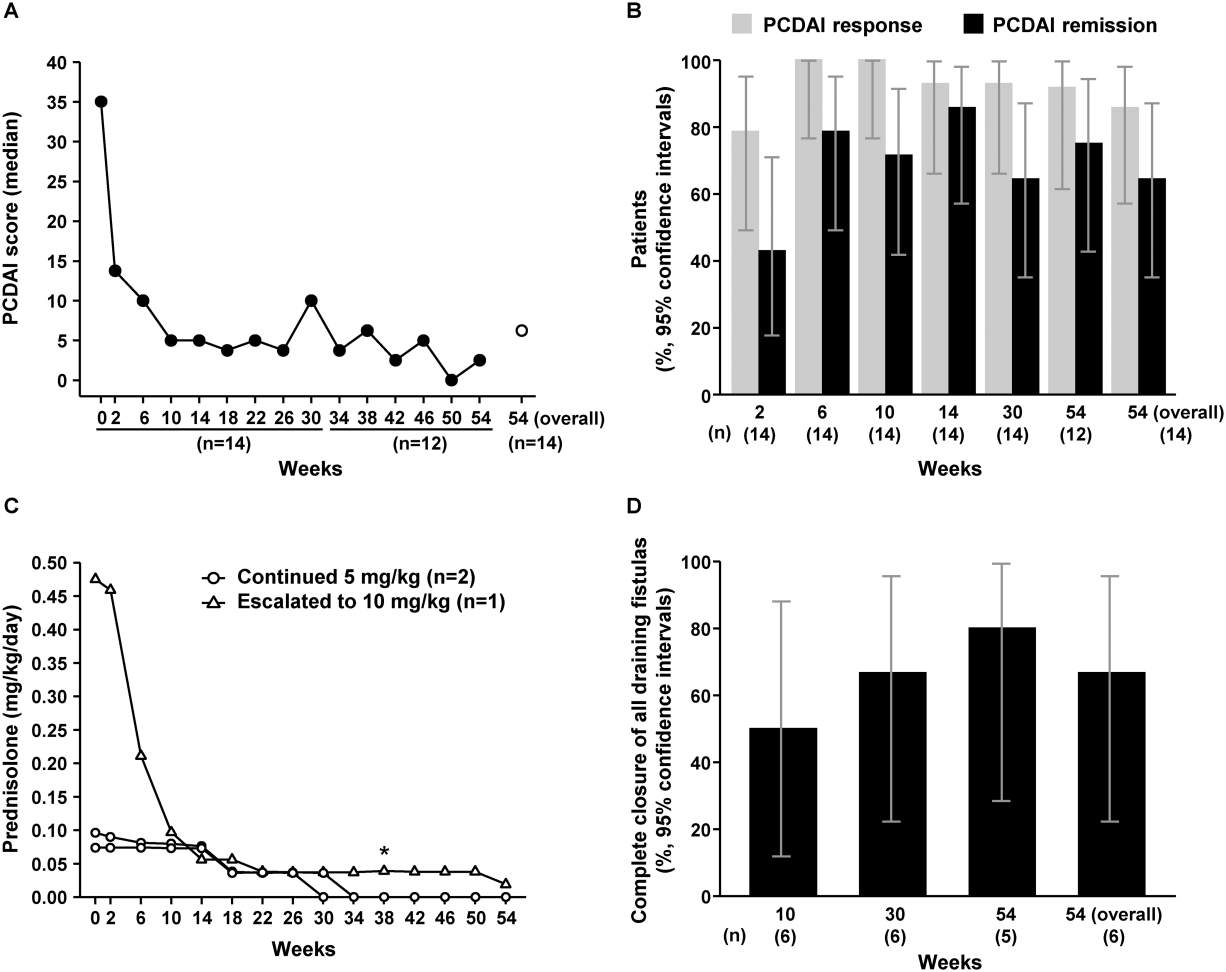
Efficacy responses to IFX treatment over time in Japanese pediatric patients with CD. (A) Median PCDAI score at Weeks 0–54 in the FAS population. Open circle represents the median PCDAI score at Week 54 (overall). (B) PCDAI response and remission rates at Weeks 2–54. (C) Corticosteroid dose at Weeks 0–54 in three patients with baseline corticosteroid use. Asterisk represents the start of dose escalation. (D) Rates of complete closure of draining fistulas at Weeks 10–54. CD, Crohn’s disease; FAS, full analysis set; IFX, infliximab; PCDAI, Pediatric Crohn’s Disease Activity Index.

PCDAI-related assessments of IFX efficacy are shown in Fig 3. Median (IQR) PCDAI score decreased from 35.00 (35.00, 35.00) at baseline to 13.75 (10.00, 17.50) at Week 2, 10.00 (5.00, 10.00) at Week 6, and 5.00 (0.00, 12.50) at Week 10, then ranged from 0.00 to 10.00 from Week 14 to Week 54. The Week 54 (overall) median (IQR) PCDAI score was 6.25 (0.00, 20.00) (Fig 3A). The percentage of patients with a PCDAI response at Weeks 2, 6, and 10 were 78.6% (11/14, 95% CI: 49.2 to 95.3%), 100.0% (14/14, 95% CI: 76.8 to 100.0%), and 100.0% (14/14, 95% CI: 76.8 to 100.0%), respectively, and ranged from 91.7% (11/12, 95% CI: 61.5 to 99.8%) to 100.0% (12/12, 95% CI: 73.5 to 100.0%) between Weeks 14 and 54 (Fig 3B). PCDAI response rate at Week 54 (overall) was 85.7% (12/14, 95% CI: 57.2 to 98.2%). The PCDAI remission rates at Weeks 2, 6, and 10 were 42.9% (6/14, 95% CI: 17.7 to 71.1%), 78.6% (11/14, 95% CI: 49.2 to 95.3%), and 71.4% (10/14, 95% CI: 41.9 to 91.6%), respectively, and ranged from 64.3% (9/14, 95% CI: 35.1 to 87.2%) to 91.7% (11/12, 95% CI: 61.5 to 99.8%) from Week 14 to Week 54. PCDAI remission rate at Week 54 (overall) was 64.3% (9/14, 95% CI: 35.1 to 87.2%) (Fig 3B).

### Corticosteroid dose

Among patients using concomitant oral corticosteroids at baseline (median dose 0.10 mg/kg/day; n = 3), median percentage change in corticosteroid dose during the study was -3.37%, -15.63%, and -16.67% at Weeks 2, 6, and 10, respectively. Thereafter, two of the three patients withdrew from corticosteroid treatment (one at Week 30 and one at Week 34; Fig 3C), and both achieved PCDAI remission.

### CRP measurements

At baseline, median CRP levels were 2.25 mg/dL (n = 14). Following IFX administration, median CRP levels decreased to 0.10 mg/dL at Week 2, then ranged from 0.00 to 0.20 mg/dL over the 54-week study period. Median CRP levels at Week 54 and Week 54 (overall) were 0.10 mg/dL and 0.35 mg/dL, respectively.

### Draining fistulas

A total of six patients had draining fistulas at registration (two patients had three fistulas, and four patients had one). Improvement (a decrease in the number of draining fistulas of ≥50% from baseline) was seen in four patients (66.7%) at last observation following IFX treatment. The same four patients also had complete closure of fistulas at last observation following IFX treatment (Fig 3D). Of the patients with draining fistulas, two received an increased dose of 10 mg/kg IFX. In both cases, complete closure was achieved before the dose increase and was maintained until the end of the study. No new draining fistulas were observed over the course of the study, regardless of whether or not patients had draining fistulas at registration.

### Pharmacokinetics

Median (IQR) IFX concentration 1 hour after administration was 91.48 (84.91, 98.68) μg/mL at Week 0, 104.80 (93.02, 116.85) μg/mL at Week 22, and 126.13 (105.87, 133.07) μg/mL at Week 46. Median (IQR) IFX concentrations before administration at Weeks 2 and 6 were 17.47 (15.87, 23.74) and 13.47 (10.57, 17.08), respectively. Median IFX concentration at Week 10 was 16.46 (12.12, 20.09) μg/mL. The median trough concentration at Week 14 was 4.54 (2.32, 5.39) μg/mL and ranged from 1.42 to 3.75 μg/mL during Weeks 22–54.

**Fig 4:**
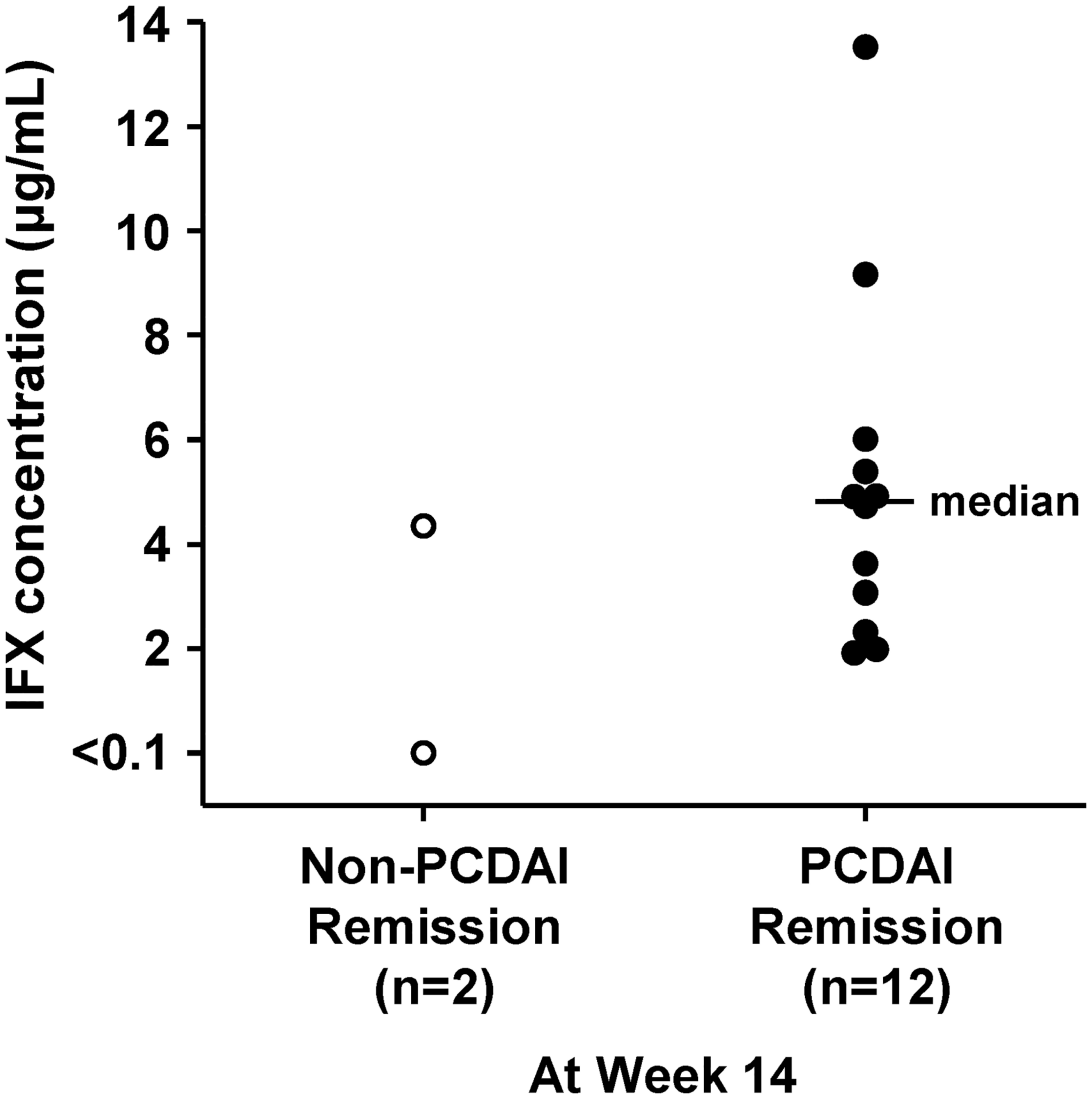
Trough serum IFX concentration at week 14 in patients who achieved PCDAI remission and in those who did not achieve PCDAI remission. IFX, infliximab; PCDAI, Pediatric Crohn’s Disease Activity Index.

The median trough serum IFX concentrations in patients achieving PCDAI remission versus patients who did not achieve remission were 3.75 μg/mL (n = 9) versus 1.38 μg/mL (n = 5) at Week 30 and 5.35 μg/mL (n = 9) versus 1.16 μg/mL (n = 3) at Week 54. The median trough serum IFX concentration in patients (n = 12) who achieved PCDAI remission at Week 14 was 4.82 μg/mL, and the individual trough serum IFX concentrations in patients (n = 2) who did not achieve PCDAI remission were 4.35 and <0.10 μg/mL (Fig 4). The patient with an IFX concentration of 4.35 μg/mL achieved a PCDAI response. ATI determination was inconclusive in all patients.

### Dose escalation

**Fig 5:**
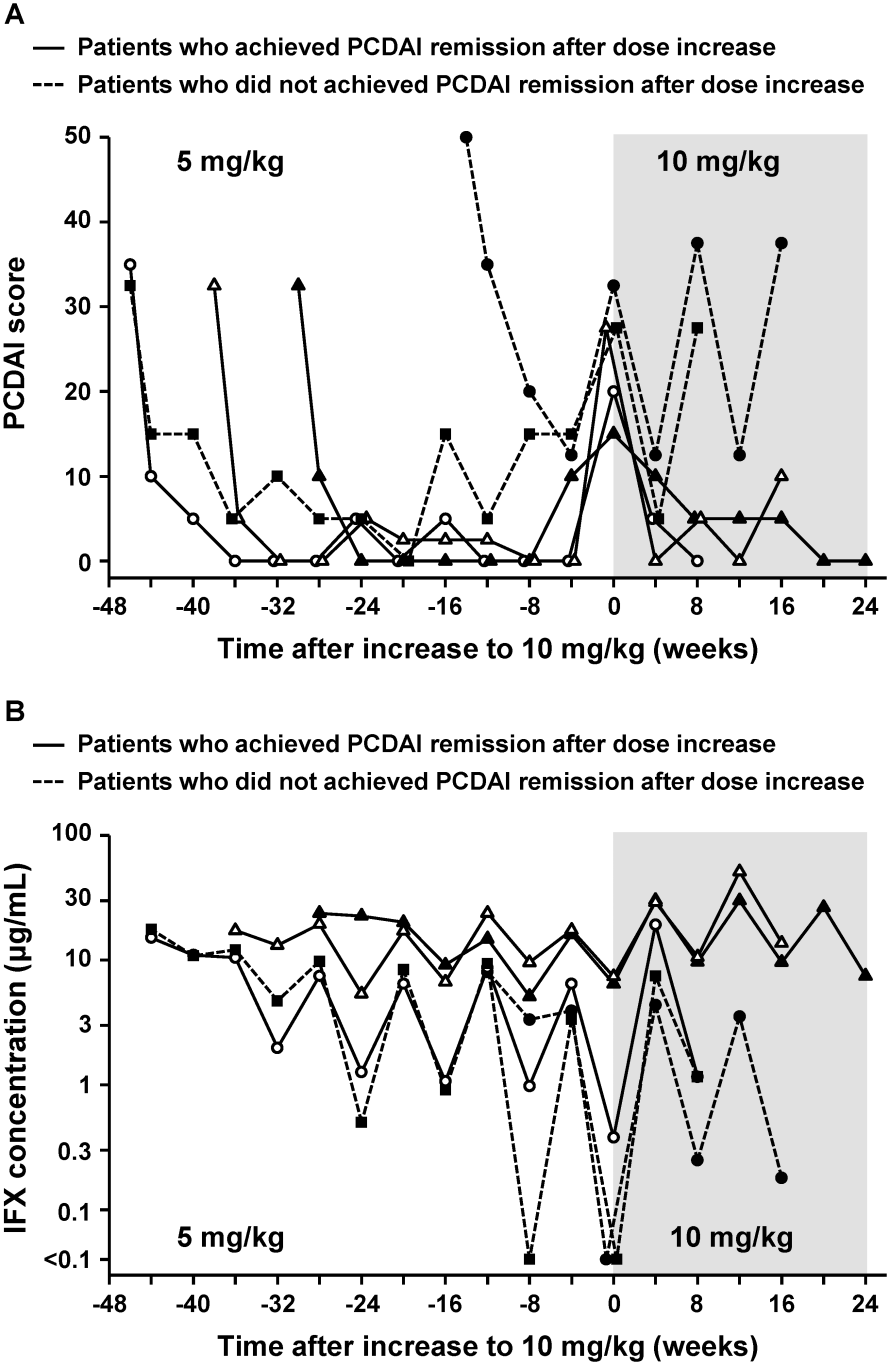
Individual responses from five patients who met criteria for IFX dose increasing from 5 mg/kg to 10 mg/kg. (A) PCDAI from dose-increase Weeks -48 to 24. (B) IFX serum concentrations from dose-increase Weeks -48 to 24. IFX, infliximab; PCDAI, Pediatric Crohn’s Disease Activity Index. The time point of dose increase was defined as Week 0 of dose increase.

Fig 5A shows individual change in PCDAI score over time among the five patients who received a dose increase to 10 mg/kg, with data aligned to Week 0 of each dose increase. The median PCDAI score 8 weeks before the dose increase was zero and increased to 27.5 by dose-increase Week 0. From dose-increase Week -8 to Week 0, there was a decrease in the percentage of patients with a PCDAI response (from 100.0% [5/5; 95% CI: 47.8 to 100.0%] to 40.0% [2/5; 95% CI: 5.3 to 85.3%]) and in PCDAI remission rate (from 60.0% [3/5; 95% CI: 14.7 to 94.7%] to 0.0% [0/5; 95% CI: 0.0 to 52.2%]). By 8 weeks post-dose increase, PCDAI response rates and remission rates were both 60.0% (3/5; 95% CI: 14.7 to 94.7%).

Fig 5B shows individual changes in IFX concentration over time among the five patients who received a dose increase to 10 mg/kg, with data aligned to Week 0 of each dose increase. In two patients with IFX concentrations of <0.10 μg/mL at dose-increase Week 0, IFX concentration increased to 0.25 and 1.16 μg/mL at 8 weeks post-dose increase; however, neither achieved PCDAI remission. In the three remaining patients, IFX concentration rose at 8 weeks post-dose increase (0.38 to 1.16 μg/mL, 6.47 to 9.67 μg/mL, and 7.38 to 10.63 μg/mL) and PCDAI remission was achieved.

### Safety

The safety profile of IFX is presented in Table 2. Incidence proportions of AEs and ADRs in the overall population were 100.0% (14/14,95% CI: 76.8 to 100.0%) and 64.3% (9/14, 95% CI: 35.1 to 87.2%), respectively. Most AEs in the overall population belonged to the system organ class “infections and infestations”, with an incidence proportion of 71.4% (10/14, 95% CI: 41.9 to 91.6%). The incidence proportion of serious AEs (SAEs) in the overall population was 14.3% (2/14, 95% CI: 1.8 to 42.8%), and SAEs were observed only in the patients receiving continuous treatment with 5 mg/kg (worsened CD and small intestinal obstruction in one patient each). A causal relationship between the events and the study drug was ruled out in both cases. No serious ADRs were observed in the overall population.

**Table 2:** Incidence of AEs following 5 mg/kg or 10 mg/kg IFX.

	**All patients**		**5 mg/kg IFX** **maintenance therapy**		**10 mg/kg IFX** **increase therapy**	
Number of treated patients	14		9		5	
Mean duration of follow-up (days)	353.9		359.9		242.6 (5 mg/kg), 100.4 (10 mg/kg)	
Mean number of infusions	7.6		7.7		5.6 (5 mg/kg), 1.8 (10 mg/kg)	
AEs, n (% [95% CI])	14 (100.0 [76.8 to 100.0])		9 (100.0 [66.4 to 100.0])		5 (100.0 [47.8 to 100.0])	
ADRs, n (% [95% CI])	9 (64.3 [35.1 to 87.2])		6 (66.7 [29.9 to 92.5])		3 (60.0 [14.7 to 94.7])	
AEs leading to discontinuation of study drug, n (% [95% CI])	1 (7.1 [0.2 to 33.9])		1 (11.1 [0.3 to 48.2])		0 (0.0 [0.0 to 52.2])	
SAEs, n (% [95% CI])	2 (14.3 [1.8 to 42.8])		2 (22.2 [2.8 to 60.0])		0 (0.0 [0.0 to 52.2])	
Serious ADRs, n (% [95% CI])	0 (0.0 [0.0 to 23.2])		0 (0.0 [0.0 to 33.6])		0 (0.0 [0.0 to 52.2])	
Infections, n (% [95% CI])	10 (71.4 [41.9 to 91.6])		7 (77.8 [40.0 to 97.2])		3 (60.0 [14.7 to 94.7])	
Serious infections, n (% [95% CI])	0 (0.0 [0.0 to 23.2])		0 (0.0 [0.0 to 33.6])		0 (0.0 [0.0 to 52.2])	
Infusion reactions, n (% [95% CI])	0 (0.0 [0.0 to 23.2])		0 (0.0 [0.0 to 33.6])		0 (0.0 [0.0 to 52.2])	
	**All patients**^**†**^		**5 mg/kg**^**‡**^		**10 mg/kg**^**§**^	
Number of treated patients	14		14		5	
Patient-years	13.6		12.2		1.4	
	**Events**	**Events/ 100 PYs (95% CI)**	**Events**	**Events/ 100 PYs (95% CI)**	**Events**	**Events/ 100 PYs (95% CI)**
AEs	62	457.1 (356.6 to 585.9)	59	484.0 (375.3 to 624.3)	3	218.3 (74.3 to 642.0)
ADRs	15	110.6 (67.0 to 182.5)	14	114.9 (68.4 to 192.8)	1	72.8 (12.8 to 412.3)
SAEs	2	14.7 (4.0 to 53.8)	2	16.4 (4.5 to 59.8)	0	0.0 (0.0 to 279.6)
Infections	23	169.6 (113.0 to 254.5)	22	180.5 (119.2 to 273.3)	1	72.8 (12.8 to 412.3)

AE, adverse event; ADR, adverse drug reaction; CI, confidence interval; IFX, infliximab; PY, patient-year; SAE, serious adverse event.

Patient-years were defined as the total person-years by exposure to dose (^†^overall; ^‡^only 5 mg/kg administration; ^§^10 mg/kg administration).

The incidence proportions of AEs and ADRs in patients receiving continuous treatment with 5 mg/kg IFX were 100.0% (9/9) and 66.7% (6/9), respectively. The incidence proportions of AEs and ADRs in the patients receiving an increased dose of 10 mg/kg were 100.0% (5/5) and 60.0% (3/5), respectively. These were of a similar incidence and severity to those seen in patients receiving continuous treatment with 5 mg/kg IFX. The incidence rate of AEs by person-years was also similar in the increased-dose and continuous 5 mg/kg treatment groups.

## Discussion

This was the first Phase 3 study to demonstrate the efficacy and safety of IFX for the treatment of moderate-to-severe CD in an Asian pediatric population. Administration of IFX was well tolerated and rapidly improved clinical symptoms, and the effects were maintained for up to 54 weeks.

Our findings are comparable with those reported by a previous Phase 3 study that assessed IFX efficacy in 57 Japanese adults with CD using CDAI score as an outcome measure [17]. In the current study, PCDAI was assessed, as this measure has been shown to be better at discriminating between levels of disease activity in pediatric patients and has been reported to have good correlation with CDAI [20]. Week 10 responder rate was similar in both studies (100.0% versus 96.9%). The PCDAI response rate at Week 14 in the present study was also similar to the CDAI response at Week 14 in the Japanese adult Phase 3 study (92.9% versus 84.2%); however, the remission rate at Week 14 in the present study exceeded that observed in the Japanese adult Phase 3 study (85.7% [95% CI: 57.2 to 98.2%] versus 49.1%). One potential explanation for the difference in efficacy between the studies is that in the Japanese adult Phase 3 study, patients with CD had a greater median disease duration (5.8 years) [17] compared with a median of 0.8 years in the present study and, therefore, may have been harder to treat.

Elevation of IFX dose in response to loss of response (LOR) is an effective means of regaining clinical effectiveness [16,21,22]. IFX dose was increased to 10 mg/kg in five patients who showed LOR to IFX after Week 14 in the present study. LOR rate in the present study is similar to that seen in the Japanese adult Phase 3 study (35.7% [95% CI: 12.8 to 64.9%] versus 35%) [17]. The changes in overall clinical response and remission rates from Weeks 14 to 54 were comparable between both studies, although the means of intensifying the IFX dose was different between the two studies (increased dose versus shortened dosing interval).

Reduction in corticosteroid use is an important objective in CD management, particularly in pediatric populations [12]. The results from this study show that two of three patients (66.7%) receiving corticosteroids at baseline withdrew corticosteroid treatment by Week 54. These patient numbers are small, but reductions in concomitant corticosteroid use following IFX treatment have been reported previously [15–17,23]. The Japanese adult Phase 3 study reported that 13 of 18 patients (72.2%) withdrew corticosteroid treatment by Week 54 of IFX therapy [17]. The efficacy of IFX on closure of fistulas was also shown in the present study and in the previous Japanese adult Phase 3 study [17], although the number of patients was small.

These results suggest that efficacy of IFX in pediatric Japanese patients with CD is similar to that in adult Japanese patients with CD.

In the REACH study, which investigated the efficacy and safety of IFX for non-Japanese pediatric populations with CD on concomitant immunomodulator use [9,16], the Week 10 PCDAI response and remission rates were 88.4% and 58.9%, respectively, and the mean PCDAI score decreased by around 30 points from baseline at Weeks 10 and 54 [16]. Considering the small sample size and different study design of the present study, the Week 10 PCDAI response and remission rates (100.0% [95% CI: 76.8 to 100.0%] and 71.4% [95% CI: 41.9 to 91.6%], respectively), and the decrease in median PCDAI score from baseline (35.00 at baseline, 5.00 at Week 10, and 6.25 at Week 54 [overall]) are broadly comparable with the results seen in the REACH study.

Another key treatment aim in pediatric patients with CD is optimization of growth. The REACH study reported that IFX had a positive effect on growth in pediatric patients with a delayed bone age [9,16]. The effect of IFX on growth could not be investigated in this study due to the small number of pediatric patients with CD indicated for IFX treatment.

The relationship between clinical efficacy and serum IFX trough level has been reported in adult and pediatric patients with CD [17,24–26]. In the Japanese adult Phase 3 study, the efficacy of IFX at Week 14 correlated with its serum trough level at Week 14 [17]. In the present study, the trough serum IFX concentration in two patients who did not achieve PCDAI remission at Week 14 was lower than the median trough serum IFX concentration in patients who achieved PCDAI remission at Week 14. Median trough serum IFX concentrations in patients receiving therapy of 5 mg/kg was maintained at a high level in the present study and in the Japanese adult Phase 3 study [17]. On the other hand, the IFX trough concentration in the three patients who showed LOR at dose-increase Week 0 was <1.0 μg/mL. This correlates with the Japanese adult Phase 3 study as the threshold of trough concentrations of IFX to obtain clinical efficacy was reported to be around 1.0 μg/mL [17]. In the Japanese adult Phase 3 escalation study, IFX dose escalation to 10 mg/kg led to remission in approximately 40% of patients who showed LOR to 5 mg/kg IFX treatment and the effectiveness of IFX dose escalation was dependent on pre-escalation concentrations of IFX [21]. The results from the present study show that PCDAI remission was achieved in 60.0% (95% CI: 14.7 to 94.7%) of patients following an increase in IFX dose from 5 mg/kg to 10 mg/kg, and the two patients with IFX concentrations of <0.10 μg/mL at dose-increase Week 0 did not achieve PCDAI remission.

Overall, these findings suggest that the PK profile of IFX and the relationship between clinical efficacy and serum IFX trough level in pediatric Japanese patients with CD is similar to that in adult Japanese patients with CD. In addition, IFX dose escalation retrieves its trough concentration and may be a useful treatment option for patients who showed LOR, although the number of patients was small.

IFX treatment was well tolerated in this study. The incidence proportions of AEs (100.0%), SAEs (14.3%), and infections (71.4%) were comparable with results obtained for patients in the Japanese adult Phase 3 study (98.2%, 12.3%, and 75.4%, respectively) [17] and the REACH study (96.2%, 15.1%, and 73.6% in patients receiving treatment at 8-week intervals, respectively) [16]. There was no substantial difference in safety profile between patients who received 5 mg/kg continuously and patients who received an increased dose of 10 mg/kg. No AEs that may be of concern with IFX treatment (such as systemic lupus erythematosus, malignant tumor, demyelinating disease, interstitial pneumonia, liver function disorder, delayed hypersensitive reaction, congestive heart failure, serious blood disorder, or rhabdomyolysis) were reported, and no new events of concern were observed.

The main limitations of this study were the small sample size and the lack of a control group. The sample size was decided on consultation with the Pharmacological and Medical Device Agency based on the rarity and severity of the disease, the target population, and the commercial availability of IFX. The small sample size restricted the ability of this study to detect less-common AEs. For ethical reasons, a control group was not included in the study design.

Despite these limitations, we have shown that 5 mg/kg IFX treatment in Japanese pediatric patients with moderate-to-severe CD and inadequate response to existing therapies improved clinical outcome measures from Weeks 2–54. In cases where treatment became less effective due to decreased trough IFX concentrations, an increased dose of 10 mg/kg was administered, leading to restoration of the clinical effect. No AEs of concern were observed at either dose.

## Supporting information

S1 File Protocol in Japanese. (PDF)

S2 File Protocol summary in English. (PDF)

S3 File CONSORT 2010 checklist. (DOC)
