# Correction: Infliximab for pediatric patients with Crohn’s disease: A Phase 3, open-label, uncontrolled, multicenter trial in Japan

**DOI:** 10.1371/journal.pone.0261932

**Published:** 2022-04-19

**Authors:** Hitoshi Tajiri, Satoshi Motoya, Fukunori Kinjo, Atsuo Maemoto, Takayuki Matsumoto, Noriko Sato, Hiroshi Yamada, Mieko Nagano, Yutaka Susuta, Kunihiko Ozaki, Kazuoki Kondo, Toshifumi Hibi

An incorrect version of S1 File was published as two separate Supporting Information documents (S1 and S2 Files in the original article) in error. Please view the correct S1 File below.

## Supporting information

S1 File Protocol summary in English. (PDF)
