# Supplementary material for: Infliximab for pediatric patients with Crohn’s disease: A Phase 3, open-label, uncontrolled, multicenter trial in Japan
Source: PLoS One. 2018 Aug 16;13(8):e0201956. doi: 10.1371/journal.pone.0201956 (PMC6095542; doi:10.1371/journal.pone.0201956)
Supplement: S1 File — (PDF) [file pone.0201956.s001.pdf]

総括報告書

TA-650 の小児の潰瘍性大腸炎を対象とした臨床試験

治験依頼者

田辺三菱製薬株式会社

治験実施計画書番号：TA-650-21
版番号：第 2 版

1. 標題ページ

治験の標題	TA-650 の小児の潰瘍性大腸炎を対象とした臨床試験
被験薬名	TA-650
対象とした適応	潰瘍性大腸炎
試験デザイン<br>比較<br>期間<br><br><br><br><br><br>用量<br><br>患者集団	非盲検・非対照，多施設共同試験<br>該当なし<br>観察期間：CAI スコア評価開始日から治験薬の投与開始までの期間とする．<br>評価期間：治験薬の投与開始から 30 週の評価までの期間とする．ただし，CAI スコア不応例及び中止した被験者は，投与開始から最終投与 8 週後の評価までの期間とする．<br>各投与日の体重 1 kg 当たり 5 mg の TA-650 を 1 回の投与量とし，2 時間以上かけて緩徐に点滴静注する．<br>中等症から重症の小児の潰瘍性大腸炎患者
治験依頼者名	田辺三菱製薬株式会社
治験実施計画書番号	TA-650-21
開発のフェーズ	第 III 相
治験開始日	2012 年 4 月 25 日（最初の登録被験者の同意日）
治験中止日	該当せず
治験終了日	2014 年 9 月 25 日「最終の被験者の最終評価日（追跡調査を除く）」
治験依頼者の医学専門家の所属・氏名	・学校法人北里研究所 北里大学北里研究所病院<br>炎症性腸疾患先進治療センター センター長 日比 紀文<br>・田辺三菱製薬株式会社 顧問 近藤 和興
治験依頼者の責任者名	田辺三菱製薬株式会社 創薬本部 臨床開発第三部<br>マネジャー 山田 博司
GCP 遵守の陳述	本治験は，医薬品の臨床試験の実施の基準（GCP）に関する省令及び関連法規を遵守して実施された．なお，各治験記録は同基準に基づいて実施医療機関又は治験依頼者で保管されている．
報告書作成日	2016 年 2 月 4 日（第 2 版）

2.　概要

<table>
<tr><td>治験依頼者名：
田辺三菱製薬株式会社</td><td rowspan="3">各治験の要約表

申請資料中の該当箇所

分冊番号：
ページ：</td><td rowspan="3">（審査当局使用欄）</td></tr>
<tr><td>商品名：
レミケード®点滴静注用 100</td></tr>
<tr><td>有効成分名：インフリキシマブ（遺伝子組換え）</td></tr>
</table>

<table>
<tr><td colspan="2">治験の標題：TA-650 の小児の潰瘍性大腸炎を対象とした臨床試験</td></tr>
<tr><td colspan="2">実施医療機関及び治験責任医師名：付録 16.1.4 参照（合計 34 施設，38 名）</td></tr>
<tr><td colspan="2">公表文献（引用文献）：該当なし</td></tr>
<tr><td>治験期間：約 2 年 5 ヶ月
（最初の登録被験者の同意日）　2012 年 4 月 25 日
「最終の被験者の最終評価日（追跡調査を除く）」　2014 年 9 月 25 日</td><td>開発のフェーズ：
第Ⅲ相</td></tr>
<tr><td colspan="2">目的：
中等症から重症の小児の潰瘍性大腸炎患者を対象に TA-650 5 mg/kg を 0，2，6 週に投与し，以降 8 週間隔で 14，22 週に投与し，30 週までの有効性について，CAI スコアなどの評価指標を用いて検討する．また，併せて安全性及び薬物動態を検討する．</td></tr>
<tr><td colspan="2">治験方法：非盲検・非対照，多施設共同試験</td></tr>
<tr><td colspan="2">被験者数（計画時及び解析時）：
計画時：目標被験者数 治験薬が投与された被験者数として 20 名
解析時：登録被験者数 21 名，有効性解析対象 21 名，安全性解析対象 21 名，薬物動態解析対象 21 名</td></tr>
</table>

治験依頼者名：<br>田辺三菱製薬株式会社	各治験の要約表<br><br>申請資料中の該当箇所<br><br>分冊番号：<br>ページ：	（審査当局使用欄）
商品名：<br>レミケード®点滴静注用 100		
有効成分名：インフリキシマブ（遺伝子組換え）		

診断及び主要な組入れ基準：

以下の選択基準をすべて満たす患者を対象とする（入院，外来は不問）.

(1) 対象疾患：潰瘍性大腸炎.
厚生労働省「難治性炎症性腸管障害に関する調査研究」班の潰瘍性大腸炎診断基準（案）（2010 年 2 月 13 日改訂）において潰瘍性大腸炎と診断され，同意取得時点で少なくとも 3 ヶ月以上罹患している患者.

(2) 年齢 6 歳以上 17 歳以下（同意取得時）の患者.

(3) 本治験への参加について，代諾者から文書同意，患者本人から口頭又は文書によるアセントが得られる患者.

(4) 登録時の CAI スコアが 7 以上かつ CAI スコアの血便スコアが 2 以上の患者.

(5) 下記に示す 1）～4）の治療経験のうち，少なくとも 1 つを満たす患者.

1) 6-メルカプトプリン又はアザチオプリン：観察期間開始日の 12 週以上前より使用しており 4 週以上前から一定用量で使用している.

2) ステロイド（経口剤）：観察期間開始日の 2 週以上前からプレドニゾロン換算で 1 mg/kg/日以上又は 20 mg/日以上で一定用量使用している.

3) 観察期間開始日前 5 年以内に，以下のどちらかを満たす患者.
・6-メルカプトプリン又はアザチオプリンを 12 週以上使用し効果不十分を経験した.
・6-メルカプトプリン又はアザチオプリンを用い，治療継続が困難となる副作用を認めた.

4) 観察期間開始日前 18 ヶ月以内に，以下の少なくとも 1 つを満たす患者.
・ステロイドの減量に伴って増悪又は再燃が起こり離脱困難を経験した.
・ステロイドを使用し効果不十分（プレドニゾロン換算で 1 mg/kg/日以上又は 40 mg/日以上を経口で少なくとも 2 週間，静注で少なくとも 1 週間投与して治療効果が得られない）を経験した.
・ステロイドを使用し，治療継続が困難となる副作用を認めた.

(6) 観察期間開始時に使用している薬剤について以下を満たす患者.

1) 6-メルカプトプリン又はアザチオプリン：観察期間開始日の 12 週以上前から使用しており 4 週以上前から用量一定で使用している, 又は観察期間開始日の 4 週以上前から使用していない患者.

2) ステロイド（経口剤）：観察期間開始日の 2 週以上前から用量一定で使用している, 又は観察期間開始日の 2 週以上前から使用していない患者.

3) 5-アミノサリチル酸製剤（経口剤）又はサラゾスルファピリジン製剤（経口剤）：観察期間開始日の 2 週以上前から用量一定で使用している, 又は観察期間開始日の 2 週以上前から使用していない患者.

<table>
<tr><td>治験依頼者名：
田辺三菱製薬株式会社</td><td rowspan="3">各治験の要約表

申請資料中の該当箇所

分冊番号：
ページ：</td><td rowspan="3">（審査当局使用欄）</td></tr>
<tr><td>商品名：
レミケード®点滴静注用 100</td></tr>
<tr><td>有効成分名：インフリキシマブ（遺伝子組換え）</td></tr>
</table>

被験薬，用量及び投与方法，ロット番号：
・被験薬：TA-650（インフリキシマブ（遺伝子組換え））
・用量及び投与方法：
各投与日の体重 1 kg 当たり 5 mg の TA-650 を 1 回の投与量とし，2 時間以上かけて緩徐に点滴静注する．初回投与（0 週），2 週，6 週に投与し，8 週 CAI スコア判定による CAI スコア応答例は，14 週，22 週に治験薬投与を同様に行う．ただし，CAI スコア不応例に対しては 14 週以降の治験薬投与は行わない．
・ロット番号：110001，120075

対照治療，用量及び投与方法，ロット番号：該当なし

治療期間：
観察期間：CAI スコア評価開始日から治験薬の投与開始までの期間.
評価期間：治験薬の投与開始から 30 週の評価までの期間．ただし，CAI スコア不応例及び中止した被験者は，投与開始から最終投与 8 週後の評価までの期間.

評価基準
有効性：
(1) CAI スコア
(2) CAI スコア寛解
(3) パーシャル Mayo スコア
(4) Mayo スコア
(5) Mayo スコア改善
(6) Mayo スコア寛解
(7) 粘膜治癒
(8) PUCAI スコア
(9) PUCAI スコア寛解
(10) PUCAI スコア変化量 20 ポイント以上減少
(11) ステロイド使用量
(12) ステロイド離脱

安全性：
(1) 有害事象及び副作用
・有害事象
・副作用

薬物動態：
(1) 血清中インフリキシマブ濃度（血清中 TA-650 濃度）
(2) 抗インフリキシマブ抗体（TA-650 に対する抗体，以下 ATI）

治験依頼者名：<br>田辺三菱製薬株式会社	各治験の要約表<br><br>申請資料中の該当箇所<br><br>分冊番号：<br>ページ：	（審査当局使用欄）
商品名：<br>レミケード®点滴静注用 100		
有効成分名：インフリキシマブ（遺伝子組換え）		

統計手法：

(1) 有効性

有効性の解析は最大の解析対象集団｛Full Analysis Set（以下，FAS）｝を対象として行った．ステロイドの使用量以外は，以下の記載と同様に解析を行った．

欠測値に対する補完をしない場合（各評価時点に「最終」の時点を含む）と補完（Treatment Failure，以下 TF）を適用した場合の両方の結果を示した（各サブスコアの要約統計量はこの限りではない）．

1) CAI スコア

(a) 評価項目

・CAI スコア，CAI スコア寛解率

(b) 解析方法

・各評価時点における，CAI スコア及び CAI スコア変化量の要約統計量と CAI スコア寛解率を算出した．

2) パーシャル Mayo スコア

(a) 評価項目

・パーシャル Mayo スコア

(b) 解析方法

・各評価時点における，パーシャル Mayo スコア及びパーシャル Mayo スコア変化量の要約統計量を算出した．

3) Mayo スコア

(a) 評価項目

・Mayo スコア，Mayo スコア改善率，Mayo スコア寛解率，粘膜治癒率

(b) 解析方法

・各評価時点における，Mayo スコア及び Mayo スコア変化量の要約統計量と Mayo スコア改善率，Mayo スコア寛解率，粘膜治癒率を算出した．

4) PUCAI スコア

(a) 評価項目

・PUCAI スコア，PUCAI スコア寛解率，PUCAI スコア変化量 20 ポイント以上減少率

(b) 解析方法

・各評価時点における，PUCAI スコア及び PUCAI スコア変化量の要約統計量と PUCAI スコア寛解率，PUCAI スコア変化量 20 ポイント以上減少率を算出した．

5) ステロイド使用量

(a) 評価項目

・ステロイド使用量，ステロイド離脱率

(b) 解析方法

・FAS のうち登録時にステロイド（経口剤）を使用していた被験者を対象とした．

・欠測値に対する補完をしない場合（各評価時点に「最終」の時点を含む）と補完（TF）を適用した場合の両方の結果を示した．

・各評価時点における，ステロイド使用量及びステロイド使用量の変化率の要約統

治験依頼者名：<br>田辺三菱製薬株式会社	各治験の要約表<br><br>申請資料中の該当箇所<br><br>分冊番号：<br>ページ：	（審査当局使用欄）
商品名：<br>レミケード®点滴静注用 100		
有効成分名：インフリキシマブ（遺伝子組換え）		

計量とステロイド離脱率を算出した.

(2) 安全性

安全性の解析は安全性解析対象集団を対象として行った.

評価期間中の有害事象及び副作用の発現率を算出した．その他，時期別の有害事象，投与回数別の Infusion reaction，治験薬投与 1 回あたりの Infusion reaction，ATI 別の Infusion reaction，程度別の有害事象の発現率を算出した．更に，一般臨床試験については異常変動の発現率，免疫血液学的検査については各判定のクロス表，理学的検査については要約統計量を集計した.

(3) 薬物動態の評価

1) 解析方法

・各評価時点の血清中インフリキシマブ濃度の要約統計量を示した.

・ATI の判定（陰性，陽性，評価不能）について度数分布，割合を示すとともに各評価時点の血清中インフリキシマブ濃度の要約統計量を ATI 判定別に示した.

治験依頼者名：<br>田辺三菱製薬株式会社	各治験の要約表<br><br>申請資料中の該当箇所<br><br>分冊番号：<br>ページ：	（審査当局使用欄）
商品名：<br>レミケード®点滴静注用 100		
有効成分名：インフリキシマブ（遺伝子組換え）		

要約－結論

有効性の結果：

(1) CAI スコア（Mean±SD）は，登録時には 9.7±2.7 であったが，投与 2，6，8 週ではそれぞれ 4.0±3.2，3.5±3.5，3.2±3.5 と経時的に減少した．投与 10～30 週では 2.5±2.3～3.5±2.2 で推移した．また，最終は 5.6±3.8 であった．CAI スコア変化量（Mean±SD）は，投与 2，6，8 週で-5.5±2.7，-6.0±4.0，-6.2±3.7 と大きくなり，投与 10～30 週では-5.9±3.8～-6.9±3.8 の間で推移した．また，最終では-4.1±4.2 であった．CAI スコア及び CAI スコア変化量共に，投与 2 週時点から症状改善効果が認められ，その効果が投与 30 週まで持続した．なお，TF 補完を行った場合でも，CAI スコア及び CAI スコア変化量共に，TF 補完前のデータと同様の傾向を示した．

(2) CAI スコア寛解率は，投与 2 週で 60.0%（12/20 名）となり，投与 6，8 週共に 80.0%（16/20 名）と増加し，投与 10～30 週までの間では 64.3～87.5%で推移した．また，最終では 42.9%（9/21 名）であった．CAI スコア寛解率において，本剤を投与することにより，投与 2 週から寛解の状態に導く効果が認められ，その効果が投与 30 週まで持続することが確認された．なお，TF 補完した場合も同様の傾向であった．

(3) パーシャル Mayo スコア（Mean±SD）は，登録時では 5.6±1.6 であったが，投与 2，6，8 週ではそれぞれ 2.6±2.3，2.2±2.0，1.7±1.7 と経時的に減少し，投与 10～30 週では 1.8±1.7～2.8±1.9 の間で推移した．また，最終では 3.7±2.2 であった．パーシャル Mayo スコア変化量（Mean±SD）は，投与 2，6，8 週ではそれぞれ-2.9±2.2，-3.3±2.2，-3.8±1.7 と経時的に大きくなり，投与 10～30 週では-2.6±2.8～-3.6±2.5 の間で推移した．また，最終では-1.9±2.7 であった．パーシャル Mayo スコア及びパーシャル Mayo スコア変化量共に，投与 2 週時点から症状改善効果を認め，その効果が投与 30 週まで持続した．なお，TF 補完した場合もパーシャル Mayo スコア及びパーシャル Mayo スコア変化量では同様の傾向であった．

(4) Mayo スコア（Mean±SD）は，登録時では 7.0±2.4 であったが，投与 30 週では 4.9±2.4 に減少した．また，最終では 5.0±2.3 であった．Mayo スコア変化量（Mean±SD）は，投与 30 週に-2.6±3.9 であった．また，最終では-2.0±3.9 であった．なお，TF 補完した場合も Mayo スコア及び Mayo スコア変化量においても補完前と同様の傾向が確認された．

(5) Mayo スコアの改善率及び寛解率は，投与 30 週ではそれぞれ 42.9%（3/7 名），14.3%（1/7 名）であった．また，最終ではそれぞれ 37.5%（3/8 名），12.5%（1/8 名）であった．なお，TF 補完した場合も Mayo スコアの改善率及び寛解率においても補完前と同様の値を呈した．

治験依頼者名：<br>田辺三菱製薬株式会社	各治験の要約表<br><br>申請資料中の該当箇所<br><br>分冊番号：<br>ページ：	（審査当局使用欄）
商品名：<br>レミケード®点滴静注用 100		
有効成分名：インフリキシマブ（遺伝子組換え）		

(6) PUCAI スコア（Mean±SD）は，登録時では 47.1±15.2 であったが，投与 2，6，8 週ではそれぞれ 20.3±16.3，17.3±17.5，12.5±13.5 と経時的に減少した．投与 10～30 週では 12.2±12.0～19.3±18.8 の間で推移した．また，最終では 28.8±22.5 であった．PUCAI スコア変化量（Mean±SD）は，投与 2，6，8 週でそれぞれ-25.8±17.5，-28.8±21.1，-33.5±14.0 を示した．投与 10～30 週では-26.8±27.8～-34.1±17.7 の間で推移した．また，最終では-18.3±28.4 であった．PUCAI スコア及び PUCAI スコア変化量共に，投与 2 週時点から症状改善効果を認め，その効果が投与 30 週まで持続した．なお，TF 補完した場合も，PUCAI スコア及び PUCAI スコア変化量については，補完前と同様の傾向であった．

(7) PUCAI スコア寛解率は，投与 2 週で 35.0%（7/20 名）となり，投与 6，8 週共に 40.0%（8/20 名）と増加した．その後，投与 10～30 週までの間では 28.6～42.9%で推移した．また，最終では 19.0%（4/21 名）となった．投与 2 週から寛解の状態へ導く効果を認め，その効果は持続することが確認された．なお，TF 補完した場合も同様の傾向であった．

(8) PUCAI スコア変化量が 20 ポイント以上減少した被験者の割合は，投与 2 週で 68.4%（13/19 名）となり，投与 6，8 週ではそれぞれ 73.7%（14/19 名），89.5%（17/19 名）と増加した．その後，投与 10～30 週までの間では 64.3～88.2%の間で推移した．また，最終では 60.0%（12/20 名）であった．投与 2 週から改善効果を認め，その効果はその後も持続することが確認された．なお，TF 補完した場合も同様の傾向であった．

(9) 粘膜治癒率において，投与 30 週及び最終ではそれぞれ 33.3%（2/6 名），28.6%（2/7 名）であった．なお，TF 補完した場合も同様の傾向であった．

(10) 登録時にステロイド（経口剤）を使用していた被験者 12 名において，ステロイド使用量（Median）は，登録時に 0.20 mg/kg/day であったが，投与 2，6，8 週ではそれぞれ 0.19，0.17，0.16 mg/kg/day と減少した．また，投与 10～30 週では 0.04～0.15 mg/kg/day の間で推移した．また，最終では 0.05 mg/kg/day であった．ステロイド使用量の変化率（Median）は，投与 2，6，8 週ではそれぞれ-1.63，-25.05，-43.91%と経時的にその割合は大きくなった．投与 10～30 週では-61.72～-86.93%の間で推移した．また，最終は-85.44%であった．なお，TF 補完した場合もステロイド使用量及びステロイド使用量の変化率では同様の傾向であり，本剤投与により，ステロイドを減量できる可能性が示唆された．

(11) ステロイド離脱率は，投与 2 週で 8.3%，投与 6，8 週で共に 25.0%とその割合は増加した．投与 10～30 週では 25.0～37.5%で推移した．また，最終は 41.7%であった．なお，TF 補完した場合も，その推移は補完前のデータと同様の傾向であった．投与 30 週時点でステロイド離脱した被験者は 2 名であり，そのうち 1 名が CAI スコア寛

治験依頼者名：<br>田辺三菱製薬株式会社	各治験の要約表<br><br>申請資料中の該当箇所<br><br>分冊番号：<br>ページ：	（審査当局使用欄）
商品名：<br>レミケード®点滴静注用 100		
有効成分名：インフリキシマブ（遺伝子組換え）		

解に該当していた.

(12) 有効性解析対象被験者を 6～12 歳未満と 12～17 歳以下の 2 つの集団に分けて有効性について検討した結果，両集団の被験者数に違いはあったものの，両集団における有効性に関して，大きく異なるものではないと考えられた.

(13) CAI スコア応答例におけるトラフの血清中インフリキシマブ濃度（Median）である投与 14，22 及び 30 週では，それぞれ 2.58，1.54，1.34 μg/mL と血清中インフリキシマブ濃度を維持していた.

(14) 投与 30 週の CAI スコア変化量（Median）は，0.1 μg/mL 未満，0.1 以上 1 μg/mL 未満，1 以上 10 μg/mL 未満，10 μg/mL 以上でそれぞれ-4.5，NC（算出不能），-6.0，NC であった．NC を呈した濃度の集団の個別の CAI スコア変化量は，0.1 以上 1 μg/mL 未満で-4，-3，10 μg/mL 以上で-10 であった．これらの結果から，被験者数が少ない血清中インフリキシマブ濃度の集団があるものの，血清中インフリキシマブ濃度が高いほど有効性も高くなる傾向が認められた.

(15) ATI の判定は，治験期間中，全体において，評価不能が 81.0%（17/21 名），陰性が 19.0%（4/21 名）であり，陽性を示した被験者はなかった.

以上，本剤を 5 mg/kg で 0，2，6 週に投与することにより，中等症から重症の小児の潰瘍性大腸炎に対する臨床症状及び粘膜治癒の改善効果が得られることが示された. その後, 14, 22 週と 8 週間隔で継続して投与することにより，その効果が持続することが確認された．また，本剤を投与することにより，ステロイドを減量又は離脱できる可能性が示唆された.

安全性の結果：

治験薬を少なくとも 1 回投与され，かつ，治験薬投与開始後の安全性データが得られた 21 名を対象に安全性評価を行い，以下の結論を得た.

(1) 有害事象及び副作用の発現率は，それぞれ 95.2%（20/21 名）及び 71.4%（15/21 名）であった．発現率が高かった有害事象の器官別大分類については「感染症および寄生虫症」及び「臨床検査」が共に 57.1%（12/21 名），「胃腸障害」52.4%（11/21 名）で，発現率が高かった有害事象は「二本鎖 DNA 抗体陽性」57.1%（12/21 名），「鼻咽頭炎」33.3%（7/21 名）であった．また，発現率が高かった副作用の器官別大分類は，「臨床検査」57.1%（12/21 名）で，発現率が高かった副作用は「二本鎖 DNA 抗体陽性」57.1%（12/21 名）であった.

(2) 重篤な有害事象及び重篤な副作用の発現率は，それぞれ 14.3%（3/21 名）及び 4.8%（1/21 名）であった．「潰瘍性大腸炎」（医師記載名「潰瘍性大腸炎の悪化」）が 2 名に認められたが，治験薬との因果関係は否定された．重篤な副作用としては「腸炎」

治験依頼者名：<br>田辺三菱製薬株式会社	各治験の要約表<br><br>申請資料中の該当箇所<br><br>分冊番号：<br>ページ：	（審査当局使用欄）
商品名：<br>レミケード®点滴静注用 100		
有効成分名：インフリキシマブ（遺伝子組換え）		

が認められ，程度は中等度で，無治療で回復した．

(3) 中止に至った有害事象の発現率は，4.8%（1/21 名）で，「潰瘍性大腸炎」（医師記載名「潰瘍性大腸炎の悪化」）であった．中止に至った副作用は認められなかった．

(4) 感染症及び感染症（副作用）の発現率は，それぞれ 61.9%（13/21 名）及び 23.8%（5/21 名）であった．最も発現率が高かった感染症の事象は「鼻咽頭炎」33.3%（7/21 名）であった．重篤な感染症の発現率は 4.8%（1/21 名）で，「腸炎」のみであった．投与中止に至った感染症は認められなかった．

(5) Infusion reaction 及び Infusion reaction（副作用）の発現率は共に 9.5%（2/21 名）であった．事象の程度は中等度が 1 名と軽度が 1 名に認められたが，いずれの事象も発現日同日で回復に至った．また，重篤な Infusion reaction 及び投与中止に至った Infusion reaction は認められなかった．

(6) 免疫血清学的検査の有害事象の発現率は 57.1%（12/21 名）であり，「二本鎖 DNA 抗体(IgM)陽性」57.1%（12/21 名），「抗核抗体増加」4.8%（1/21 名）であった．ループス様症候群に相関するといわれている抗 dsDNA IgG 抗体が陽性化した被験者は認められなかった．また，ループス様症候群を発現した被験者も認められなかった．

(7) 本剤において留意すべき事象である悪性腫瘍（小児や若年成人で報告されている肝脾 T 細胞リンパ腫を含む），脱髄疾患，間質性肺炎，肝機能障害，遅発性過敏反応（血清病様反応を含む），うっ血性心不全，重篤な血液障害及び横紋筋融解症は認められなかった．

(8) 一般臨床検査の異常変動発現率が 10%以上であった臨床検査項目は 7 項目認められ，尿蛋白（増加），尿潜血（増加）が共に 47.6%（10/21 名），ALT（GPT）（減少）33.3%（7/21 名），白血球数（減少），ALP（減少），γ-GTP（減少），BUN（減少）がそれぞれ 14.3%（3/21 名）であった．臨床上問題となるような変動は認められなかった．

(9) 理学的検査（収縮期血圧，拡張期血圧，脈拍数，体温）の要約統計量の変化から特に問題となる傾向は認められなかった．また，理学的検査に関連した有害事象は「血圧低下」が 4.8%（1/21 名）で発現したが，治験薬との因果関係は否定された．

以上，中等症から重症の小児の潰瘍性大腸炎患者に対して，本剤を 22 週まで投与した際の 30 週間の結果から，忍容性はおおむね良好であることが確認された．また，成人の潰瘍性大腸炎を含む既承認疾患の安全性プロファイルと同様，感染症，Infusion reaction，免疫血清学的検査に関連する事象が認められた．また，本剤投与に際して懸念される事象の発現頻度や重篤度が大きく高まることはなく，新たに留意すべき事象はないと考えられた．

<table>
<tr><td>治験依頼者名：
田辺三菱製薬株式会社</td><td rowspan="3">各治験の要約表

申請資料中の該当箇所

分冊番号：
ページ：</td><td rowspan="3">（審査当局使用欄）</td></tr>
<tr><td>商品名：
レミケード®点滴静注用 100</td></tr>
<tr><td>有効成分名：インフリキシマブ（遺伝子組換え）</td></tr>
</table>

結論：<br>本治験において，中等症から重症の小児の潰瘍性大腸炎患者に対して，TA-650 5 mg/kg を 0，2，6 週に投与し，以降 8 週間隔で投与することにより，臨床症状，粘膜治癒などの改善効果が認められ，また，その効果を維持しながらステロイドの減量又は離脱ができる可能性も示唆された．安全性については成人の潰瘍性大腸炎患者の安全性プロファイルと同様に，感染症，Infusion reaction，免疫血清学的検査に関連する事象が多く認められるものの，本剤投与に際して懸念される事象の発現頻度や重篤度が大きく高まることはなかった．これらのことから小児の潰瘍性大腸炎患者に対して本剤が有用であると考えられた．

3. 目次

1. 標題ページ 2
2. 概要 3
3. 目次 13
4. 略号及び用語の定義一覧 17
5. 倫理 21
5.1 治験審査委員会（IRB） 21
5.2 治験の倫理的実施 21
5.3 患者への情報及び同意 21
6. 治験責任医師等及び治験管理組織 23
6.1 治験依頼者 23
6.1.1 治験依頼者 23
6.1.2 治験依頼責任者 23
6.1.3 医学専門家 24
6.1.4 クリニカルリーダー 25
6.1.5 モニタリング担当責任者 26
6.1.6 モニター 27
6.1.7 監査責任者 27
6.1.8 統計解析責任者 29
6.1.9 データ管理責任者 30
6.1.10 臨床薬理解析責任者 30
6.1.11 薬物濃度等測定責任者 31
6.1.12 開発業務受託機関 32
6.2 治験調整医師 34
6.3 安全性評価委員 34
6.4 実施医療機関及び治験責任医師 35
7. 緒言 36
8. 治験の目的 38
9. 治験の計画 39
9.1 治験の全般的デザイン及び計画－記述 39
9.1.1 検討した治療法 39
9.1.2 検討した患者集団及び計画された被験者数 39
9.1.3 盲検化の水準と手法 39
9.1.4 対照の種類及び試験の構成 39
9.1.5 治療への割付手法 39
9.1.6 治験期間の順序と長さ 39

9.1.7 いつ患者が無作為化されたかの特定 40
9.1.8 設置した各種委員会とその役割 40
9.1.9 中間解析 41
9.2 対照群の選択を含む治験デザインについての考察 41
9.3 治験対象集団の選択 41
9.3.1 組み入れ基準 41
9.3.2 除外基準 43
9.3.3 被験者の治療又は評価の打ち切り 47
9.4 治療法 49
9.4.1 治療法 49
9.4.2 治験薬の同定 50
9.4.3 治療群への患者の割付方法 51
9.4.4 治験における用量の選択 51
9.4.5 各患者の用量の選択及び投与時期 52
9.4.6 盲検化 52
9.4.7 前治療及び併用療法 52
9.4.8 治療方法の遵守 56
9.5 有効性及び安全性の項目 57
9.5.1 有効性及び安全性の評価項目及びフローチャート 57
9.5.2 測定項目の適切性 77
9.5.3 薬物濃度の測定 78
9.6 データの品質保証 79
9.7 治験実施計画書で計画された統計手法及び被験者数の決定 79
9.7.1 統計及び解析計画 79
9.7.2 有効性の評価 85
9.7.3 安全性の評価 86
9.7.4 その他の評価 89
9.7.5 薬物動態の評価 89
9.7.6 被験者数の決定 89
9.8 治験の実施又は計画された解析に関する変更 90
9.8.1 治験実施計画書の改訂 90
9.8.2 解析計画の変更 90
10. 治験対象患者 90
10.1 患者の内訳 90
10.2 治験実施計画書からの逸脱 92
11. 有効性の評価 93
11.1 解析したデータセット 93

11.2　人口統計学的及び他の基準値の特性 93
11.3　治療の遵守状況の測定 98
11.4　有効性に関する成績及び個別患者データ一覧表 99
11.4.1　有効性の解析 99
11.4.2　統計・解析上の論点 119
11.4.3　個別反応データの作表 121
11.4.4　薬剤の用量，薬物濃度及びそれらと反応との関係 121
11.4.5　薬物－薬物及び薬物－疾患の相互作用 125
11.4.6　患者ごとの表示 125
11.4.7　有効性の結論 125
12. 安全性の評価 129
12.1　治験薬が投与された被験者数，期間及び用量 129
12.2　有害事象 129
12.2.1　有害事象の簡潔な要約 129
12.2.2　有害事象の表示 130
12.2.3　有害事象の分析 132
12.2.4　患者ごとの有害事象の一覧表 134
12.3　死亡，その他の重篤な有害事象及び他の重要な有害事象 134
12.3.1　死亡，その他の重篤な有害事象及び他の重要な有害事象の一覧表 134
12.3.2　死亡，その他の重篤な有害事象及び他のいくつかの重要な有害事象の叙述 134
12.3.3　死亡，その他の重篤な有害事象及び他の重要な有害事象の分析及び考察 134
12.4　臨床検査値の評価 141
12.4.1　患者ごとの個々の臨床検査異常値の一覧表 141
12.4.2　各臨床検査項目の評価 141
12.5　バイタルサイン，身体的所見及び安全性に関連する他の観察項目 142
12.5.1　理学的検査（収縮期血圧，拡張期血圧，脈拍数，体温） 142
12.5.2　免疫血清学的検査 143
12.6　安全性の結論 144
13. 考察と全般的結論 146
14. 本文中には含めないが，引用する表，図及びグラフ 148
14.1　人口統計学的データ 148
14.2　有効性データ 148
14.3　安全性データ 149
14.3.1　有害事象の表示 149
14.3.2　死亡，その他の重篤な有害事象及び他の重要な有害事象の一覧表 151
14.3.3　死亡，その他の重篤な有害事象及び他の特に重要な有害事象の叙述 152
14.3.4　患者ごとの個々の臨床検査異常値の一覧表 155

15. 引用文献の一覧表 167
16. 付録 168
16.1 治験に関する情報 168
16.1.1 治験実施計画書及びその改訂 168
16.1.2 症例記録用紙の見本 168
16.1.3 治験審査委員会の一覧，患者への説明文書及び同意文書の見本 168
16.1.4 治験責任医師及び他の重要な治験参加者の一覧表及び説明 168
16.1.5 治験依頼者の医学責任者の署名 168
16.1.6 各被験者に投与された薬剤のロット番号一覧表 168
16.1.7 無作為化の方法及びコード（患者の識別及び割付けられた治療） 168
16.1.8 監査手順に関する資料，監査証明書 168
16.1.9 統計手法に関する文書 168
16.1.10 臨床検査に関して施設間の標準化及び品質保証の方法と手順に関する文書 169
16.1.11 治験に基づく公表文献 169
16.1.12 総括報告書で引用された重要な公表文献 169
16.1.13 その他の付録 169
16.1.14 薬物動態に関する文書　（該当する場合） 169
16.2 被験者データ一覧表 169
16.2.1 中止被験者 169
16.2.2 治験実施計画から逸脱した被験者 169
16.2.3 有効性の解析から除外された被験者 169
16.2.4 人口統計学的データ 169
16.2.5 服薬遵守及び（又は）薬物濃度データ 169
16.2.6 個々の有効性反応データ 170
16.2.7 被験者ごとの有害事象一覧表 170
16.2.8 被験者ごとの臨床検査値一覧表 170
16.3 症例記録 170
16.3.1 死亡，その他の重篤な有害事象を発現した被験者及び有害事象による投与中止した被験者の症例記録 170
16.3.2 提出された他の症例記録 170

4.　略号及び用語の定義一覧

略号一覧表

略号	省略していない表現英語	日本語訳
ATI	Antibodies to Infliximab	抗インフリキシマブ抗体
BCG	Bacille de Calmette et Guérin	カルメット-ゲラン桿菌
BLQ	Below lower limit of quantification	定量限界値未満
CAI	Clinical activity index	－
CRP	C-reactive protein	C-反応性蛋白
CT 検査	Computed tomography	コンピューター断層検査
dsDNA	double stranded DNA	二本鎖 DNA
EDC	Electronic Data Capture	電子的データ収集
ESR	Erythrocyte sedimentation rate	赤血球沈降速度
FAS	Full analysis set	最大の解析対象集団
GCP	Good clinical practice	医薬品の臨床試験の実施の基準
HBc	Hepatitis B core	－
HBs	Hepatitis B surface	－
HIV	Human immunodeficiency virus	ヒト免疫不全ウイルス
IL-6	Interleukin-6	インターロイキン-6
INH	Isoniazid	イソニアジド
LOCF	Last Observation Carried Forward	－
NC	Not calculated	算出不能
NSAIDs	Non-steroidal anti-inflammatory drug	非ステロイド性消炎鎮痛剤
PUCAI	Pediatric Ulcerative Colitis Activity Index	－
Max	Maximum	最大値
Min	Minimum	最小値
n	Number of subjects	被験者数
Q1	Lower Quartile	第 1 四分位点
Q3	Upper Quartile	第 3 四分位点
QFT	QuantiFERON	クォンティフェロン
QOL	Quality of life	生活の質
SD	Standard deviation	標準偏差
TB	Tuberculosis	結核
TF	Treatment Failure	－
TNFα	Tumor necrosis factor - alpha	腫瘍壊死因子 α

用語の定義一覧表

用語	定義
登録日	被験者の適格性確認が終了した日
治験期間	観察期間開始日から評価期間最終日までとする
観察期間	CAI スコア評価開始日から治験薬の投与開始までの期間とする
有効性評価期間	治験薬の投与開始から 30 週の評価までの期間とする．ただし，CAI スコア不応例では投与開始から 8 週の評価までの期間とし，中止した被験者では投与開始から中止時評価までの期間とする
安全性及び薬物動態評価期間（=評価期間）	治験薬の投与開始から 30 週の評価までの期間．ただし，CAI スコア不応例及び中止した被験者は，投与開始から最終投与 8 週後の評価までの期間とする
評価日	CAI スコア，パーシャル Mayo スコア，PUCAI スコアの算出に必要な評価（診察）を行った日
CAI スコア応答例	8 週の CAI スコアが登録時と比較して減少（改善）した被験者
CAI スコア不応例	8 週の CAI スコアが登録時と比較して不変又は増加（悪化）した被験者
CAIスコア症状シート評価期間<br>パーシャル Mayo スコア症状シート評価期間	CAI スコアは評価日 7 日前から評価前日までの期間とし，パーシャル Mayo スコアは評価日 3 日前から評価前日までの期間とする．ただし，その期間内に排便回数に影響する薬剤の使用がある場合は，以下に示す評価除外日を除いた評価日前 7 日間又は 3 日間をそれぞれの症状シート評価期間とする．<br>［評価除外日］<br>・便秘や下痢の治療薬の使用日<br>・強力な止瀉作用を有する薬剤（塩酸ロペラミド，アヘンアルカロイド，硫酸アトロピン含有製剤など）の使用日及び使用後 2 日間（計 3 日間）<br>・S 状結腸鏡検査などの前処置として排便回数に影響を与える薬剤など（下剤など）を使用した日<br>・大腸内視鏡検査又は S 状結腸鏡検査の実施日及び実施後 3 日間（計 4 日間）
CAIスコア	1 週間の排便回数，血便（1 週間平均で），医師の症状アセスメント，腹痛，潰瘍性大腸炎による体温上昇，腸管外合併症，臨床検査の 7 つの項目におけるそれぞれのスコアの合計スコア（0～29）
CAIスコア寛解	評価日（登録時評価を除く）の CAI スコアが 4 以下となった場合
Mayoスコア	治験責任（分担）医師が 0～3 の 4 段階で評価する 4 つのサブスコア（排便回数，直腸からの出血，医師による全般評価，内視鏡所見）の合計スコア（0～12）
パーシャル<br>Mayo スコア	Mayo スコアのサブスコアのうち，排便回数サブスコア，直腸からの出血サブスコア，医師による全般評価サブスコアの合計スコア（0～9）

Mayo スコア改善	評価日（登録時評価を除く）の Mayo スコアが以下の 2 つを満たす場合とする．<br>-Mayo スコア：登録時と比較して 30%以上減少かつ 3 ポイント以上減少<br>-直腸からの出血サブスコア：登録時と比較して 1 ポイント以上減少，又は 1 以下
Mayo スコア寛解	評価日（登録時評価を除く）の Mayo スコアが 2 以下かつすべてのサブスコアが 1 以下
PUCAI スコア	腹痛，直腸からの出血，便の硬さ，24 時間あたりの排便回数，夜間排便，活動性レベルの 6 項目におけるそれぞれのスコアの合計スコア（0～85）
PUCAI スコア寛解	評価日（登録時評価を除く）の PUCAI スコアが 10 未満となった場合
ステロイド離脱	評価日（登録時評価を除く）のステロイド使用量が0となった場合
粘膜治癒	評価日（登録時評価を除く）の Mayo スコアの内視鏡所見サブスコアが 1 以下でかつ，登録時内視鏡スコア 1 の被験者を除くものとする
infusion reaction	治験薬投与中又は投与終了後2時間以内に認められた有害事象とする
左側大腸炎	病変の範囲が脾彎曲部を越えていないもの
全大腸炎	病変の範囲が脾彎曲部を越えているもの
代諾者	治験への参加について，被験者に十分な同意の能力がない場合に，被験者とともに，又は被験者に代わって同意をすることが正当なものと認められる者．被験者の親権者，後見人，その他これらに準じる者で，両者の生活の実質や精神的共同関係から見て，被験者の最善の利益を図りうる者でなければならない．
アセント	法的規制を受けない小児被験者からの同意

測定単位一覧表

測定項目	測定単位	測定項目	測定単位
血圧	mmHg	アルブミン	g/dL
脈拍数	拍／分	総コレステロール	mg/dL
体温	°C	総ビリルビン	mg/dL
赤血球数	10^4/μL	BUN	mg/dL
ヘモグロビン	g/dL	血清クレアチニン	mg/dL
ヘマトクリット値	%	Na	mEq/L
白血球数	/μL	K	mEq/L
白血球分画（好中球）	%	Cl	mEq/L
白血球分画（好酸球）	%	CRP	mg/dL
白血球分画（好塩基球）	%	TNFα	pg/mL
白血球分画（単球）	%	IL-6	pg/mL
白血球分画（リンパ球）	%	抗 ds DNA IgG 抗体	IU/mL
血小板数	10^4/μL	抗 ds DNA IgM 抗体	U/mL
AST（GOT）	U/L	抗核抗体	倍
ALT（GPT）	U/L	尿糖	定性
ALP	U/L	尿蛋白	定性
LDH	U/L	尿ウロビリノーゲン	定性
γ-GTP	U/L	尿潜血	定性
総蛋白	g/dL		

5. 倫理

5.1 治験審査委員会（IRB）

本治験実施に先立ち，参加 34 施設の各治験審査委員会において，治験薬概要書，治験実施計画書，代諾者への同意・説明文書，患者へのアセント・説明文書，症例報告書の見本の記載内容に基づき，倫理的，科学的及び医学的妥当性の観点から本治験実施の適否に関する審査が行われた. その結果, 22 施設で承認され, 13 施設で修正の上承認とされた. その内容は, 同意・説明文書やアセント・説明文書及び補償に関する資料の記載内容の一部修正などであった．なお，これらの変更はいずれも軽微な内容であり，治験実施計画全体に係るものではなかった．また，治験実施中の治験実施計画書，治験薬概要書及び同意・説明文書やアセント・説明文書の改訂時にも，治験審査委員会の承認を得た上で治験が継続された．治験審査委員会の一覧を付録 16.1.3a に添付した.

5.2 治験の倫理的実施

本治験は，ヘルシンキ宣言に基づく倫理的原則に留意し，「医薬品，医療機器等の品質，有効性及び安全性の確保等に関する法律」，「医薬品の臨床試験の実施の基準に関する省令［Good Clinical Practice（GCP）］」及び治験実施計画書を遵守して実施した.

治験実施計画書及び契約書には GCP 遵守の記載があり, 治験依頼者は定期的に実施医療機関を訪問し，実際に GCP を遵守して治験が実施されていることを確認した．更に，治験期間中に新たに入手した安全性情報は直ちに治験責任医師に報告し，安全に治験が実施されるよう配慮した．また，症例報告書の作成・取扱い，規制当局への資料提出などにおいては，被験者の秘密を保全するため，被験者の特定が不可能な被験者識別コードを用いた.

治験全体を通して実施医療機関より重大な GCP 違反の報告はなく，被験者の安全が確保され，倫理的に治験が実施されたことを確認した.

5.3 患者への情報及び同意

治験責任（分担）医師は治験実施に先立ち，治験審査委員会にて承認を得た同意・説明文書を対象となる代諾者に，アセント・説明文書を患者に手渡して，十分に説明した．また，治験協力者が補足的に説明することもできることとした．なお，説明に際し，本治験に関する説明文書に基づき，代諾者及び患者が理解できるように可能な限り平易な言葉を用いて説明し，代諾者及び患者の質問に対して十分に答えるよう努めた．代諾者及び患者が内容をよく理解したことを確認した上で，本治験への参加について，代諾者及び患者本人の自由意思により，代諾者からは文書同意，患者からは可能な限り文書でアセント（法的規制を受けない小児被験者からの同意）を取得した.

同意文書には説明を行った治験責任（分担）医師及び代諾者が，アセント文書には説明を

行った治験責任（分担）医師及び患者が，記名捺印又は署名し，各自日付を記載した．治験協力者が補足的に説明を行った場合は，当該治験協力者も記名捺印又は署名し，日付を記載した．また，治験責任（分担）医師は，代諾者が記載した同意文書に，代諾者と患者本人との関係が記載されていることを確認した．

治験責任（分担）医師は，被験者が治験に参加する前に，記名捺印又は署名と日付が記載された同意・説明文書及びアセント・説明文書を代諾者及び被験者に交付するとともに，同意文書及びアセント文書の原本は当該実施医療機関の規定に従って適切に保管した．

同意取得日及び説明に用いた同意・説明文書の版番号を症例報告書に記録した．

代諾者の同意又は被験者のアセントに関連し得る新たな重要な情報が得られた場合に，治験責任医師は，速やかに当該情報に基づき同意・説明文書及びアセント・説明文書改訂の要否を判断した．治験責任医師は，同意・説明文書及びアセント・説明文書を改訂する必要があると認めた場合には，速やかに同意・説明文書及びアセント・説明文書を改訂し，治験依頼者に提出するとともに，改めて治験審査委員会の承認を得た．また，この場合において，治験責任（分担）医師は，既に治験に参加している被験者及びその代諾者に当該情報を口頭で速やかに伝え，治験に継続して参加するか否かについて確認し，診療録に記録した．治験責任（分担）医師は，既に治験に参加している被験者及びその代諾者に対して，治験審査委員会にて改めて承認されたアセント・説明文書及び同意・説明文書を用いて説明し，治験への参加の継続について被験者及び代諾者からの自由意思により，被験者からはアセント，代諾者からは文書同意を取得した．初回の同意取得時と同様に，説明を行った治験責任（分担）医師，被験者及び代諾者が，記名捺印又は署名し，各自日付を記載した．治験協力者が補足的に説明を行った場合は，当該治験協力者も記名捺印又は署名し，日付を記載した．治験責任（分担）医師は，記名捺印又は署名と日付が記載された同意・説明文書及びアセント・説明文書を代諾者及び被験者に交付するとともに，同意文書及びアセント文書の原本は当該実施医療機関の規定に従って適切に保管した．再同意取得日及び説明に用いた同意・説明文書の版番号を症例報告書に記録した．

治験期間中，被験者の同意に関連し得る新たな安全性情報の入手及び国内添付文書改訂により同意・説明文書が 5 回改訂された．また，アセント・説明文書（13 歳以上対象）が 4 回改訂され，アセント・説明文書（12 歳以下対象）は改訂されなかった．これに伴い，それぞれの改訂時点で治験に参加しているすべての代諾者及び被験者から文書による同意及びアセントを取得した．

同意・説明文書（会社案最終版）の第 6 版（作成年月日：2014 年 8 月 6 日），アセント・説明文書（13 歳以上対象，会社案最終版）の第 5 版（作成年月日：2014 年 8 月 6 日），アセント・説明文書（12 歳以下対象，会社案最終版）の第 1 版（作成年月日：2011 年 12 月 20 日）並びに変更箇所一覧，施設版の一覧を付録 16.1.3b～3i に添付した．

6. 治験責任医師等及び治験管理組織

治験依頼責任者，医学専門家，クリニカルリーダー，監査責任者については，本報告書作成日までの変更履歴を記載した．その他の項目については，治験終了届の提出日（2015 年 4 月 24 日）までの変更履歴を記載した．

6.1 治験依頼者

6.1.1 治験依頼者

治験開始時：
田辺三菱製薬株式会社
〒541-8505 大阪府大阪市中央区北浜二丁目 6 番 18 号

2015 年 3 月 30 日変更：
田辺三菱製薬株式会社
〒541-8505 大阪市中央区道修町 三丁目 2 番 10 号

6.1.2 治験依頼責任者

治験開始時：
田辺三菱製薬株式会社　開発本部　臨床計画部
臨床計画部長　田中 正彦
〒103-8405 東京都中央区日本橋本町二丁目 2 番 6 号
Tel：03-3241-4136，Fax：03-3241-4807

業務内容：当該治験計画を承認し，治験実施計画書及び症例報告書の見本の内容，並びに当該治験実施計画書の遵守について，治験責任医師との合意を取得する．

2012 年 4 月 1 日変更：
田辺三菱製薬株式会社　開発本部　臨床計画第一部
臨床計画第一部長　田中 正彦
〒103-8405 東京都中央区日本橋本町二丁目 2 番 6 号
Tel：03-3241-4136，Fax：03-3241-4807

2012 年 5 月 7 日変更：
田辺三菱製薬株式会社　開発本部　臨床計画第一部
臨床計画第一部長　田中 正彦

〒103-8405 東京都中央区日本橋小網町 17 番 10 号
Tel：03-6748-7691，Fax：03-3663-6258

2013 年 4 月 5 日変更：
田辺三菱製薬株式会社　開発本部　臨床計画第一部
臨床計画第一部長　小林 義広
〒103-8405 東京都中央区日本橋小網町 17 番 10 号
Tel：03-6748-7691，Fax：03-3663-6258

2014 年 10 月 7 日変更：
田辺三菱製薬株式会社　開発本部　臨床開発第三部
臨床開発第三部長　井崎 仁史
〒103-8405 東京都中央区日本橋小網町 17 番 10 号
Tel：03-6748-7691，Fax：03-3663-6277

2015 年 10 月 1 日変更：
田辺三菱製薬株式会社　創薬本部　臨床開発第三部
臨床開発第三部長　井崎 仁史
〒103-8405 東京都中央区日本橋小網町 17 番 10 号
Tel：03-6748-7691，Fax：03-3663-6277

6.1.3 医学専門家

治験開始時：
慶應義塾大学医学部　内科学
教授　日比 紀文
〒160-8582 東京都新宿区信濃町 35 番地
Tel：03-3353-1211，Fax：03-3357-6156

田辺三菱製薬株式会社
顧問　近藤 和興
〒103-8405 東京都中央区日本橋本町二丁目 2 番 6 号
Tel：03-3241-4954，Fax：03-3241-4785

業務内容：治験に関する医学的な問題に対して治験依頼者へ速やかに指導・助言を行う.

2012 年 5 月 7 日変更：

田辺三菱製薬株式会社
顧問　近藤 和興
〒103-8405 東京都中央区日本橋小網町 17 番 10 号
Tel：03-6748-7685，Fax：03-3663-6254

2013 年 4 月 5 日変更：
学校法人北里研究所　北里大学北里研究所病院
炎症性腸疾患先進医療センター　センター長　日比 紀文
〒108-8642 東京都港区白金五丁目 9 番 1 号
Tel：03-3444-6161

2014 年 4 月 7 日変更：
学校法人北里研究所　北里大学北里研究所病院
炎症性腸疾患先進治療センター　センター長　日比 紀文
〒108-8642 東京都港区白金五丁目 9 番 1 号
Tel：03-3444-6161

田辺三菱製薬株式会社
顧問　近藤 和興
〒103-8405 東京都中央区日本橋小網町 17 番 10 号
Tel：03-6748-7681，Fax：03-3663-6254

6.1.4　クリニカルリーダー

治験開始時：
田辺三菱製薬株式会社 開発本部　臨床計画部
尾崎 邦彦
〒103-8405 東京都中央区日本橋本町二丁目 2 番 6 号
Tel：03-3241-4713，Fax：03-3241-4785

業務内容：当該治験計画の立案，治験結果の取りまとめ及び総括報告書の作成などを担当するグループを総括する責任を有する．また，当該治験実施計画書の作成責任を有する．

2012 年 4 月 1 日変更：
田辺三菱製薬株式会社　開発本部　臨床計画第一部
マネジャー　尾崎 邦彦
〒103-8405 東京都中央区日本橋本町二丁目 2 番 6 号

Tel：03-3241-4713，Fax：03-3241-4785

2012 年 5 月 7 日変更：
田辺三菱製薬株式会社　開発本部　臨床計画第一部
マネジャー　尾崎 邦彦
〒103-8405 東京都中央区日本橋小網町 17 番 10 号
Tel：03-6748-7696，Fax：03-3663-6258

2014 年 4 月 7 日変更：
COT リーダー　※COT：Clinical Operation Team
田辺三菱製薬株式会社　開発本部　臨床計画第一部
マネジャー　吉成 透
〒103-8405 東京都中央区日本橋小網町 17 番 10 号
Tel：03-6748-7696，Fax：03-3663-6258

2014 年 10 月 7 日変更：
田辺三菱製薬株式会社　開発本部　臨床開発第三部
マネジャー　尾崎 邦彦
〒103-8405 東京都中央区日本橋小網町 17 番 10 号
Tel：03-6748-7696，Fax：03-3663-6277

2015 年 10 月 1 日変更：
田辺三菱製薬株式会社　創薬本部　臨床開発第三部
マネジャー　山田 博司
〒103-8405 東京都中央区日本橋小網町 17 番 10 号
Tel：03-6748-7696，Fax：03-3663-6277

6.1.5　モニタリング担当責任者

治験開始時：
田辺三菱製薬株式会社　開発本部　臨床開発センター　臨床開発第二部
マネジャー　吉田 裕道
〒103-8405 東京都中央区日本橋本町二丁目 2 番 6 号
Tel：03-3241-4740，Fax：03-3241-4744

業務内容：治験の依頼，モニタリングなどを担当するグループを総括する責任を有する．

2012 年 4 月 1 日変更：
田辺三菱製薬株式会社　開発本部　臨床開発センター　臨床開発第一部
マネジャー　吉田 裕道
〒103-8405 東京都中央区日本橋本町二丁目 2 番 6 号
Tel：03-3241-4740，Fax：03-3241-4744

2012 年 5 月 7 日変更：
田辺三菱製薬株式会社　開発本部　臨床開発センター　臨床開発第一部
マネジャー　松本 幸雄
〒103-8405 東京都中央区日本橋小網町 17 番 10 号
Tel：03-6748-7722，Fax：03-3663-6277

2014 年 4 月 7 日変更：
田辺三菱製薬株式会社　開発本部　臨床開発第二部
マネジャー　尾崎 邦彦
〒103-8405 東京都中央区日本橋小網町 17 番 10 号
Tel：03-6748-7722，Fax：03-3663-6277

2014 年 10 月 7 日変更：
田辺三菱製薬株式会社　開発本部　臨床開発第三部
マネジャー　水流 裕信
〒103-8405 東京都中央区日本橋小網町 17 番 10 号
Tel：03-6748-7721，Fax：03-3663-6277

6.1.6　モニター

添付 16.1.1a 別紙 1 に示した．

業務内容：被験者の人権，安全及び福祉が保護されていること，治験が最新の治験実施計画書及び GCP などを遵守して実施され，治験データが正確かつ完全で，原資料などの治験関連記録に照らして検証できることを確認する．

6.1.7　監査責任者

治験開始時：
田辺三菱製薬株式会社　信頼性保証本部　薬事監査部　GCP 監査グループ
グループマネジャー　楠田 雅弘

〒103-8405 東京都中央区日本橋本町二丁目 2 番 6 号
Tel：03-3241-3571，Fax：03-3241-5205

業務内容：治験に係わる業務の実施，データの記録，解析，その正確な報告が，「医薬品，医療機器等の品質，有効性及び安全性の確保等に関する法律」，GCP 省令などの規制要件，標準業務手順書及び治験実施計画書に従って行われたか否かを評価するため，治験に係わる業務及び文書を体系的かつ独立に検証する．

2012 年 4 月 1 日変更：
田辺三菱製薬株式会社　信頼性保証本部　薬事 QA 部　臨床 QA グループ
グループマネジャー　楠田 雅弘
〒103-8405 東京都中央区日本橋本町二丁目 2 番 6 号
Tel：03-3241-3571，Fax：03-3241-5205

2012 年 5 月 7 日変更：
田辺三菱製薬株式会社　信頼性保証本部　薬事 QA 部　臨床 QA グループ
グループマネジャー　楠田 雅弘
〒103-8405 東京都中央区日本橋小網町 17 番 10 号
Tel：03-6748-7706，Fax：03-3663-6698

2013 年 4 月 5 日変更：
田辺三菱製薬株式会社　信頼性保証本部　薬事 QA 部　臨床 QA グループ
楠田 雅弘
〒103-8405 東京都中央区日本橋小網町 17 番 10 号
Tel：03-6748-7706，Fax：03-3663-6698

2013 年 10 月 1 日変更：
田辺三菱製薬株式会社　開発本部　臨床保証部
楠田 雅弘
〒103-8405 東京都中央区日本橋小網町 17 番 10 号
Tel：03-6748-7743，Fax：03-3663-6382

2014 年 4 月 7 日変更：
田辺三菱製薬株式会社　開発本部　臨床保証部
臨床保証部長　猪原 京子
〒103-8405 東京都中央区日本橋小網町 17 番 10 号
Tel：03-6748-7741，Fax：03-3663-6382

2015 年 10 月 1 日変更：
田辺三菱製薬株式会社　創薬本部　創薬保証部
創薬保証部長　猪原 京子
〒103-8405 東京都中央区日本橋小網町 17 番 10 号
Tel：03-6748-7741，Fax：03-3663-6382

6.1.8　統計解析責任者

治験開始時：
田辺三菱製薬株式会社　開発本部　データサイエンス部　臨床解析グループ
グループマネジャー　石川 千香夫
〒103-8405 東京都中央区日本橋小網町 17 番 10 号
Tel：03-6748-7735，Fax：03-3663-6371

業務内容：治験の統計学的事項に関して責任を持ち，治験実施計画書に基づき統計解析計画書を作成し，統計解析を実施するとともにその確認を行う．

2012 年 4 月 1 日変更：
田辺三菱製薬株式会社　開発本部　データサイエンスセンター　統計解析室
室長　石川 千香夫
〒103-8405 東京都中央区日本橋本町二丁目 2 番 6 号
Tel：03-3241-4198，Fax：03-3241-4717

2012 年 5 月 7 日変更：
田辺三菱製薬株式会社　開発本部　データサイエンスセンター　統計解析室
室長　石川 千香夫
〒103-8405 東京都中央区日本橋小網町 17 番 10 号
Tel：03-6748-7735，Fax：03-3663-6371

2014 年 4 月 7 日変更：
田辺三菱製薬株式会社　開発本部　データサイエンス部
石川 千香夫
〒103-8405 東京都中央区日本橋小網町 17 番 10 号
Tel：03-6748-7735，Fax：03-3663-6371

6.1.9　データ管理責任者

治験開始時：
田辺三菱製薬株式会社　開発本部　データサイエンス部　臨床 DM グループ
田中 嘉浩
〒103-8405 東京都中央区日本橋本町二丁目 2 番 6 号
Tel：03-3241-7902，Fax：03-3241-4717

業務内容:治験の品質管理のために,治験データの包括的な質の確保に努める責任を持ち,症例報告書により収集されたデータの固定を行う.

2012 年 4 月 1 日変更：
田辺三菱製薬株式会社　開発本部　データサイエンスセンター　統計解析室
マネジャー　後川 芳輝
〒103-8405 東京都中央区日本橋本町二丁目 2 番 6 号
Tel：03-3241-7902，Fax：03-3241-4717

2012 年 5 月 7 日変更：
田辺三菱製薬株式会社　開発本部　データサイエンスセンター　統計解析室
マネジャー　後川 芳輝
〒103-8405 東京都中央区日本橋小網町 17 番 10 号
Tel：03-6748-7737，Fax：03-3663-6371

2013 年 4 月 5 日変更：
田辺三菱製薬株式会社　開発本部　データサイエンスセンター　データマネジメント部
マネジャー　後川 芳輝
〒103-8405 東京都中央区日本橋小網町 17 番 10 号
Tel：03-6748-7737，Fax：03-3663-6451

2014 年 4 月 7 日変更：
田辺三菱製薬株式会社　開発本部　データサイエンス部　データマネジメントグループ
グループマネジャー　後川 芳輝
〒103-8405 東京都中央区日本橋小網町 17 番 10 号
Tel：03-6748-7737，Fax：03-3663-6451

6.1.10 臨床薬理解析責任者

治験開始時：

田辺三菱製薬株式会社
開発本部　臨床薬理センター　臨床薬理部　ファーマコメトリクスグループ
グループマネジャー　秋元 啓
〒103-8405　東京都中央区日本橋本町二丁目 2 番 6 号
Tel：03-3241-4735，Fax：03-3241-4757

業務内容：治験の薬物動態関連事項に関して責任を持ち，治験実施計画書に基づき薬物動態等解析計画書を作成し，薬物動態や薬力学解析を実施するとともにその確認を行う．

2012 年 4 月 1 日変更：
田辺三菱製薬株式会社　開発本部　臨床薬理センター　臨床薬理部
マネジャー　秋元 啓
〒103-8405 東京都中央区日本橋本町二丁目 2 番 6 号
Tel：03-3241-4735，Fax：03-3241-4757

2012 年 5 月 7 日変更：
田辺三菱製薬株式会社　開発本部　臨床薬理センター　臨床薬理部
マネジャー　秋元 啓
〒103-8405 東京都中央区日本橋小網町 17 番 10 号
Tel：03-6748-7763，Fax：03-3663-6449

2013 年 4 月 5 日変更：
田辺三菱製薬株式会社　開発本部　データサイエンスセンター　臨床薬理部
マネジャー　中島 成和
〒103-8405 東京都中央区日本橋小網町 17 番 10 号
Tel：03-6748-7763，Fax：03-3663-6449

2014 年 4 月 7 日変更：
田辺三菱製薬株式会社　開発本部　臨床薬理部
マネジャー　川口 敦弘
〒103-8405 東京都中央区日本橋小網町 17 番 10 号
Tel：03-6748-7763，Fax：03-3663-6449

6.1.11 薬物濃度等測定責任者

治験開始時：
田辺三菱製薬株式会社

開発本部　臨床薬理センター　臨床薬理部　バイオサイエンスグループ
グループマネジャー　筒井 尚久
〒103-8405 東京都中央区日本橋本町二丁目 2 番 6 号
Tel：03-3241-4734，Fax：03-3241-4757

業務内容：治験の薬物濃度及び薬力学的指標の測定に関して責任を持ち，治験実施計画書に基づき薬物濃度測定計画書を作成し，薬物濃度及び薬力学的指標の測定を実施するとともにその確認を行う.

2012 年 4 月 1 日変更：
田辺三菱製薬株式会社　開発本部　臨床薬理センター　臨床薬理部
マネジャー　筒井 尚久
〒532-8505 大阪府大阪市淀川区加島三丁目 16 番 89 号
Tel：06-6300-2565，Fax：06-6300-2586

2013 年 4 月 5 日変更：
田辺三菱製薬株式会社　開発本部　データサイエンスセンター　臨床薬理部
マネジャー　酒井 正樹
〒541-8505 大阪府大阪市中央区北浜二丁目 6 番 18 号
Tel：06-6205-5571，Fax：06-6205-5241

2014 年 4 月 7 日変更：
田辺三菱製薬株式会社　開発本部　臨床薬理部
マネジャー　酒井 正樹
〒541-8505 大阪府大阪市中央区北浜二丁目 6 番 18 号
Tel：06-6205-5571，Fax：06-6205-5241

6.1.12 開発業務受託機関

6.1.12.1 データ管理関連業務受託機関

イーピーエス株式会社
〒162-0822 東京都新宿区下宮比町 2 番 23 号
Tel：03-5684-7797，Fax：03- 5684-4785

業務内容：業務委託契約書に基づき，データ管理業務を行う.

6.1.12.2 EDC ベンダー

日本オラクル株式会社
〒107-0061 東京都港区北青山二丁目 5 番 8 号　オラクル青山センター
Tel：03-6834-6666

業務内容：EDC システムの運用保守管理を行う.

6.1.12.3 統計解析関連業務受託機関

株式会社タクミインフォメーションテクノロジー
〒171-0014 東京都豊島区池袋 2-40-13 池袋デュープレックス B's
Tel：03-5979-7381，Fax：03-5979-7382

業務内容：業務委託契約書に基づき，治験依頼者の下記の業務の一部を行う.
(1) 統計解析に関わる業務
(2) 上記業務に関する品質管理に関わる業務

6.1.12.4 治験薬管理関連業務受託機関

三菱倉庫株式会社
神戸支店 倉庫事業第一課
〒650-8691 兵庫県神戸市中央区東川崎町一丁目 7 番 4 号
Tel：078-360-8030，Fax：078-360-8050

業務内容：業務委託契約書に基づき，治験依頼者の治験薬管理業務の一部を行う.

6.1.12.5 薬物濃度測定機関

株式会社田辺アールアンドディー・サービス
分析化学部　青木 修
〒335-8505 埼玉県戸田市川岸二丁目 2 番 50 号
Tel：048-433-2551，Fax：048-433-2554

業務内容：薬物濃度測定に係わる試験計画書に基づき，測定及び報告の業務を行う.

6.1.12.6 臨床検査受託機関

株式会社 LSI メディエンス　メディカルソリューション本部

業務運営統括部
受託管理者：戸塚 史浩　業務推進担当者：伊東 英樹
〒174-8555 東京都板橋区志村三丁目 30 番 1 号
Tel：03-5943-9270，Fax：03- 5375-9211

業務内容：臨床検査測定に係わる治験検査業務手順書に基づき，測定及び報告の業務を行う．

6.1.12.7 緊急連絡センター

株式会社ベル・メディカルソリューションズ　臨床管理本部
緊急連絡センター責任者　田中 美保
〒171-0022 東京都豊島区南池袋二丁目 16 番 8 号 藤久ビル東三号館
Tel：0120-3274-99

業務内容：夜間及び休日の緊急連絡に対応する．

6.2 治験調整医師

地方独立行政法人大阪府立病院機構大阪府立急性期・総合医療センター
小児科　主任部長　田尻 仁
〒558-8558　大阪府大阪市住吉区万代東三丁目1番56号
Tel：06-6692-1201，Fax：06-6695-3559

業務内容：実施医療機関における治験実施計画書の解釈や治験の細目について調整する．

6.3 安全性評価委員

東北大学加齢医学研究所　抗感染症薬開発研究部門
教授　渡辺 彰

業務内容：本治験中に感染症などの重篤な有害事象が発現した場合には，安全性情報を評価し，治験の継続，変更又は中止に関して治験依頼者への提言を行う．

6.4 実施医療機関及び治験責任医師

付録 16.1.4 に示した.

治験責任医師の業務内容：治験依頼者が作成した治験実施計画書への合意，同意・説明文書の作成及び改訂，被験者の選定及び同意の取得，治験の実施，被験者に対する医療及び情報の提供，治験分担医師及び治験協力者への指導及び監督，資料及び情報の提供，モニタリング及び監査への協力，治験実施計画書からの逸脱又は変更及び有害事象の報告，症例報告書の作成，治験に係わる文書又は記録の保存を行う.

7. 緒言

治験薬 TA-650（一般名：インフリキシマブ，商品名：レミケード®）はセントコア社（現 Janssen Biotech, Inc.，米国）が遺伝子組換え技術によって創製した抗ヒト TNFαモノクローナル抗体で，TNFαが関与する種々の炎症性疾患に対し，TNFαの中和及び膜結合型 TNFαの発現細胞の傷害により効果を発揮する．本剤は 1998 年以降，2014 年 8 月までに本邦を含め，世界 105 ヶ国で承認され約 221 万人に投与されている．国内ではクローン病，関節リウマチ，強直性脊椎炎，尋常性乾癬，関節症性乾癬，膿疱性乾癬，乾癬性紅皮症，潰瘍性大腸炎及びベーチェット病による難治性網膜ぶどう膜炎，腸管型ベーチェット病，神経型ベーチェット病，血管型ベーチェット病の治療薬として承認されている．更に，関節リウマチについては投与量の増量及び投与間隔の短縮が，クローン病及び腸管型ベーチェット病，神経型ベーチェット病，血管型ベーチェット病については投与量の増量が承認されている．なお，現在, 川崎病, 乾癬の増量投与について医薬品製造販売承認事項一部変更承認申請中である．海外では小児クローン病，小児潰瘍性大腸炎についても承認されている．

潰瘍性大腸炎は，主として大腸粘膜を侵し，しばしばびらんや潰瘍を形成する原因不明のびまん性非特異性炎症疾患である．病変は直腸から口側に向かって連続に広がり，最大で直腸から結腸全体に及び，特徴的な症状は慢性の粘血便，血便である．患者によっては再燃と寛解を繰り返し，また，治療に難渋するため，長期間の医学的管理を必要とする．現在，根治的治療は大腸の摘出以外になく，指定難病とされている．その患者数は増加の一途をたどり，本治験立案当時の平成 21 年度の潰瘍性大腸炎医療受給者証交付件数は 11 万人を超えており，平成 25 年度の潰瘍性大腸炎医療受給者証所持者数は 15 万人を超えている．小児期発症の患者数は成人に比べ少ないものの，成人と同様に増加傾向にあると思われる．

潰瘍性大腸炎の治療は，薬物治療が中心である．患者によっては再燃と寛解を繰り返すため，治療に難渋することが多い．本治験立案当時の厚生労働省「難治性炎症性腸管障害に関する調査研究」班による成人の潰瘍性大腸炎治療指針（平成 22 年度）によると，重症度や病変範囲の両面から治療を決定することとされていた．軽症から中等症には，アミノサリチル酸製剤の経口剤及び注腸製剤，ステロイドの注腸製剤が使用される．これらの治療に効果不十分な場合や重症例には，ステロイドの経口剤又は点滴静注が使用される．ステロイドは中心的な治療であり，適切な用法・用量で使用すれば，寛解導入には優れた治療法であるが，寛解維持の治療には効果が乏しく，漫然とした投与は副作用を増やすこととなる．難治例であるステロイド依存例, ステロイド抵抗例には免疫調節薬が用いられる. 寛解導入目的では，シクロスポリン，タクロリムス，血球成分除去療法が使用され，寛解維持目的では，アザチオプリン，6-メルカプトプリンが使用される．しかし，既存の薬物療法では効果不十分となる場合も多く，最終的に外科的手術の適応となる患者は少なくない．このような背景の中，本剤は潰瘍性大腸炎の治療薬として 2010 年 6 月に承認され, 先の治療指針においてもステロイド依存例，ステロイド抵抗例の治療法として位置付けられた．これらの治療に関する問題点は, 小児でも同様である. 更に小児に特有な特徴として, 成人と比較して病変の広範囲化,

重症化が見られやすい．また，ステロイドの長期投与により，成長障害をはじめとするステロイド関連合併症を起こしやすく，ステロイド依存例にもなりやすいため，免疫調節薬などの積極的な治療をする場合が多い.

海外では，本剤について，小児の潰瘍性大腸炎を対象とした C0168T72 試験と成人を対象とした ACT1，ACT2 試験の結果から，小児における有効性，安全性プロファイル及び薬物動態は成人と類似していることが示唆されている．その結果，成人と同様に小児においても 5 mg/kg を 0，2，6 週に投与し，その後 8 週間隔で投与する用法・用量にて，米国では 2011 年 9 月，EU では 2012 年 2 月に承認されている.

一方，国内においては，本剤の小児の潰瘍性大腸炎患者を対象とした臨床試験が実施されていないこと，用法・用量に明示的に「小児」と記載されていないことから，本剤を必要とする小児患者に使用を躊躇される場合がある．この問題を解決するため，今回，小児の潰瘍性大腸炎患者を対象に，TA-650 5 mg/kg を 0，2，6 週に投与し，以降 8 週間隔で 14 週，22 週に投与した際の有効性，安全性及び薬物動態を検討することとした.

なお，本治験の計画に当たっては対面助言を 2010 年 12 月 21 日に実施した．議事録（作成年月日：2011 年 2 月 3 日）を付録 16.1.13h に添付した.

本対面助言において，小児の潰瘍性大腸炎を対象とした臨床試験では限られた被験者数のデータで検討することになるため，薬物動態のみでなく，有効性及び安全性についても広く評価し，総合的に判断することで合意した．評価指標については，内視鏡検査は被験者に身体的及び精神的苦痛を与え, 更には 6～8 歳の低年齢の被験者では全身麻酔下で検査を行うこともあるなど，被験者だけでなく医師にも非常に負担がかかる検査のため，必須項目とするのは困難であると考えられた．しかし，潰瘍性大腸炎に対する有効性を適切に評価するために重要な項目であるため，可能な限り実施し情報を集積することで合意した．また，対象患者が特定の年齢層に偏ることがないよう配慮することとした.

8. 治験の目的

中等症から重症の小児の潰瘍性大腸炎患者を対象にTA-650 5 mg/kgを0, 2, 6週に投与し，以降8週間隔で14，22週に投与し，30週までの有効性について，CAIスコアなどの評価指標を用いて検討する．また，併せて安全性及び薬物動態を検討する．

有効性評価項目：
(1) CAI スコア
(2) CAI スコア寛解
(3) パーシャルMayo スコア
(4) Mayo スコア
(5) Mayo スコア改善
(6) Mayo スコア寛解
(7) 粘膜治癒
(8) PUCAI スコア
(9) PUCAI スコア寛解
(10) PUCAI スコア変化量20 ポイント以上減少
(11) ステロイド使用量
(12) ステロイド離脱

安全性評価項目：
(1) 有害事象及び副作用
・有害事象
・副作用

薬物動態評価項目：
(1) 血清中インフリキシマブ濃度（血清中TA-650濃度）
(2) ATI

9. 治験の計画

9.1 治験の全般的デザイン及び計画－記述

治験実施計画書（第 02.00.00000 版，作成年月日：2013 年 5 月 22 日）を付録 16.1.1a，その変更箇所一覧を付録 16.1.1b に，症例報告書（第 01.00.00000 版，作成年月日：2011 年 12 月 20 日）を付録 16.1.2 に添付した．治験を依頼したすべての実施医療機関において，治験依頼者が作成した治験実施計画書及び症例報告書の見本を使用し，各資料の施設版は発生しなかった．

9.1.1 検討した治療法

各投与日の体重 1 kg 当たり 5 mg の TA-650 を 1 回の投与量とし，2 時間以上かけて緩徐に点滴静注した．初回投与（0 週），2 週，6 週に投与し，8 週 CAI スコア判定による CAI スコア応答例は，14 週，22 週に治験薬投与を同様に行った．ただし，CAI スコア不応例に対しては 14 週以降の治験薬投与は行わなかった．

9.1.2 検討した患者集団及び計画された被験者数

検討した患者集団：中等症から重症の小児の潰瘍性大腸炎患者
計画された被験者数：治験薬を投与した被験者数として 20 名

9.1.3 盲検化の水準と手法

本治験は，非盲検試験であるため該当しない．

9.1.4 対照の種類及び試験の構成

本治験は，非対照試験であるため該当しない．

9.1.5 治療への割付手法

本治験は，非対照試験であるため該当しない．

9.1.6 治験期間の順序と長さ

治験のデザインを図 9.1－1 に示した．

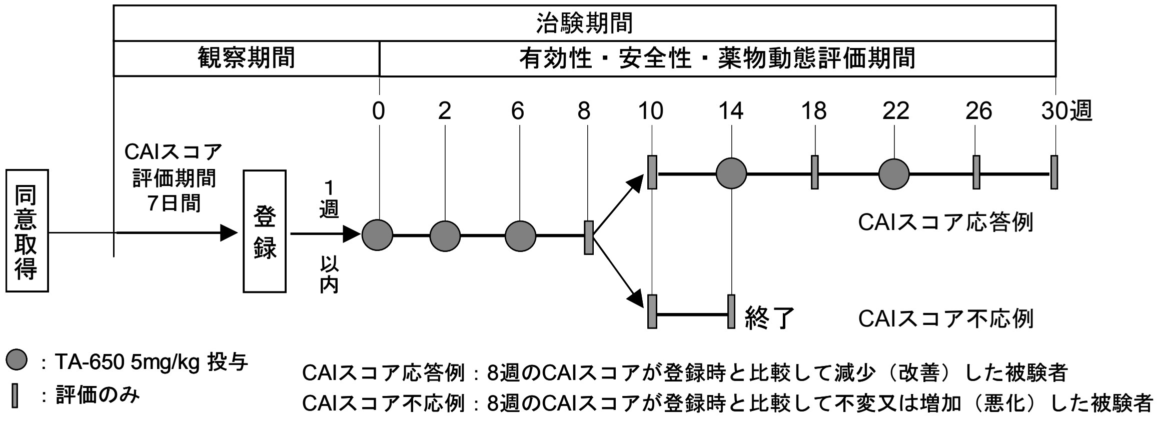

図 9.1−1　治験デザイン

8 週の CAI スコアが登録時と比較して不変又は増加（悪化）した被験者（CAI スコア不応例）は，14 週以降の投与は行わず，10 週，14 週に安全性及び薬物動態の評価を実施して治験を終了した．

治験期間：観察期間開始日から評価期間最終日までの期間とした．

観察期間：CAI スコア評価開始日から治験薬の投与開始までの期間とした．

評価期間：治験薬の投与開始から 30 週の評価までの期間とした．ただし，CAI スコア不応例及び中止した被験者は，投与開始から最終投与 8 週後の評価までの期間とした．

【各評価期間の定義】

有効性評価期間：治験薬の投与開始から 30 週の評価までの期間とした．ただし，CAI スコア不応例では投与開始から 8 週の評価までの期間とし，中止した被験者では投与開始から中止時評価までの期間とした．

安全性及び薬物動態評価期間（＝評価期間）：治験薬の投与開始から 30 週の評価までの期間とした．ただし，CAI スコア不応例及び中止した被験者は，投与開始から最終投与 8 週後の評価までの期間とした．

9.1.7　いつ患者が無作為化されたかの特定

本治験は，非対照試験であるため該当しない．

9.1.8　設置した各種委員会とその役割

本剤は，これまでの国内外における臨床試験並びに市販後の安全性情報から，結核，日和見感染症，敗血症などの重篤な感染症に注意が必要であり，本治験の実施にあたっては，安

全性を留意して推進する必要があった．したがって，治験中に得られた安全性情報について客観的な立場で評価し，治験の継続，変更又は中止に関して治験依頼者へ提言を行うことを目的とし，安全性評価委員を任命した．

安全性評価委員は，治験依頼者，医学専門家並びに治験責任（分担）医師から独立し，感染症の専門知識を有する医師 1 名に依頼し，安全性評価委員会業務手順書に従って安全性情報の評価を行った．安全性評価委員会業務手順書は付録 16.1.13b に添付した．

9.1.9 中間解析

中間解析は計画せず，実施しなかった．

9.2 対照群の選択を含む治験デザインについての考察

非盲検・非対照とした設定根拠：

小児の潰瘍性大腸炎に特有な特徴として，成人と比較して病変の広範囲化，重症化が見られやすいこと，また，成長障害をはじめとするステロイド関連合併症が起こりやすいこと，ステロイド依存例にもなりやすいことが挙げられ，小児では疾患活動性を抑え，Quality of life（以下 QOL）向上と成長障害の回避が治療目標となっている．よって，本治験で対象とする既存治療で効果不十分な被験者に対して，プラセボを対照薬とした治験の実施は成長と発達に悪影響を与えるため，倫理的問題があると考えられた．加えて，平成 21 年度の潰瘍性大腸炎医療受給者証交付を受ける，本治験の対象となる「小児」で「中等症～重症」の患者には，既存治療で寛解維持が可能である患者，成長障害や低栄養状態，小児特有の QOL 悪化による手術適応例が含まれることから，実際に治験適応となる患者は，更に少ないと考えられ，集積可能被験者数に限界があると考えた．これらの理由により，非盲検・非対照試験にて実施することとした．

9.3 治験対象集団の選択

9.3.1 組み入れ基準

以下の選択基準をすべて満たす患者を対象とした（入院，外来は不問）．

(1) 対象疾患：潰瘍性大腸炎．
厚生労働省「難治性炎症性腸管障害に関する調査研究」班の潰瘍性大腸炎診断基準（案）（2010 年 2 月 13 日改訂）（付録 16.1.1a 添付 1）において潰瘍性大腸炎と診断され，同意取得時点で少なくとも 3 ヶ月以上罹患している患者．

(2) 年齢 6 歳以上 17 歳以下（同意取得時）の患者．

(3) 本治験への参加について，代諾者から文書同意，患者本人から口頭又は文書によるアセントが得られる患者（患者の理解能力に応じるが，おおむね中学生以上の患者

については文書によるアセントを取得した．また，おおむね中学生未満の患者についても可能な限り文書によるアセントを取得することとした．)．

(4) 登録時の CAI スコアが 7 以上かつ CAI スコアの血便スコアが 2 以上の患者．

(5) 下記に示す 1）～4）の治療経験のうち，少なくとも 1 つを満たす患者．

1) 6-メルカプトプリン又はアザチオプリン：観察期間開始日の 12 週以上前より使用しており 4 週以上前から一定用量で使用している．

2) ステロイド（経口剤）：観察期間開始日の 2 週以上前からプレドニゾロン換算で 1 mg/kg/日以上又は 20 mg/日以上で一定用量使用している．

3) 観察期間開始日前 5 年以内に，以下のどちらかを満たす患者．

・6-メルカプトプリン又はアザチオプリンを 12 週以上使用し効果不十分を経験した．

・6-メルカプトプリン又はアザチオプリンを用い，治療継続が困難となる副作用を認めた．

4) 観察期間開始日前 18 ヶ月以内に，以下の少なくとも 1 つを満たす患者．

・ステロイドの減量に伴って増悪又は再燃が起こり離脱困難を経験した．

・ステロイドを使用し効果不十分（プレドニゾロン換算で 1 mg/kg/日以上又は 40 mg/日以上を経口で少なくとも 2 週間，静注で少なくとも 1 週間投与して治療効果が得られない）を経験した．

・ステロイドを使用し，治療継続が困難となる副作用を認めた．

(6) 観察期間開始時に使用している薬剤について以下を満たす患者．

1) 6-メルカプトプリン又はアザチオプリン：観察期間開始日の 12 週以上前から使用しており 4 週以上前から用量一定で使用している，又は観察期間開始日の 4 週以上前から使用していない患者．

2) ステロイド（経口剤）：観察期間開始日の 2 週以上前から用量一定で使用している，又は観察期間開始日の 2 週以上前から使用していない患者．

3) 5-アミノサリチル酸製剤（経口剤）又はサラゾスルファピリジン製剤（経口剤）：観察期間開始日の 2 週以上前から用量一定で使用している，又は観察期間開始日の 2 週以上前から使用していない患者．

【設定根拠】

(1)(4)(5) 既存療法で効果不十分な潰瘍性大腸炎患者を対象とするため設定した．

(2)本治験の目的に合わせて，小児潰瘍性大腸炎治療指針改訂案（2008 年）（日本小児栄養消化器肝臓学会潰瘍性大腸炎治療指針作成ワーキンググループ）にて，小児とは成長過程にあるという観点から 18 歳未満を意味するとされていることから，上限を 17 歳以下とした．また，疫学的な観点から，6 歳未満の有病者は極めて少ないと考えられるため，6 歳を下限として設定した．

(3)治験を倫理的に遂行するため，設定した．

(6)瘍性大腸炎治療に用いられる薬剤については，治験期間中の有効性評価を適切に行うた

めに，有効性評価開始前の使用状況を一定に設定した.

9.3.2 除外基準

以下の除外基準に 1 つでも該当する患者は対象から除外した.

(1) 全大腸炎（病変の範囲が脾彎曲部を越えているもの）であり，かつ以下の 1）又は 2）のいずれかに該当する患者.
  1) 登録時に結腸切除術が必要と判断されている患者.
  2) 登録時に，以下の(a) ～(e) のうち，4 つ以上に該当する患者.
    (a) 血性下痢が 6 回/日以上
    (b) 強い腹痛又は反跳痛がある
    (c) 37.5°C 以上の持続する発熱がある
    (d) 脈拍数：90 拍/分超
    (e) ヘモグロビン：8.5 g/dL 未満

(2) 登録前 8 週以内に潰瘍性大腸炎に対する外科的手術が施行された患者，又は登録時に潰瘍性大腸炎に対する外科的手術が必要と判断されている患者.

(3) 登録前 4 週以内に術後感染に留意が必要な外科的手術を行った患者.

(4) 大腸又は小腸に高度で症状を有する線維性の狭窄を有する患者.

(5) 腸狭窄又は腸閉塞を有することが明らかな患者，又は登録前 6 ヶ月以内に注腸 X 線検査，大腸内視鏡検査又は S 状結腸鏡検査により狭窄又は閉塞が確認されている患者.

  ［狭窄とは，注腸 X 線検査において狭窄部位と隣接する口側腸管に膨張を認める狭窄，又は内視鏡が通過できない狭窄とする.］

(6) 瘻孔を有する又は有していた患者.

(7) 中毒性巨大結腸症を合併している患者.

(8) 以下の 1）から 4）のいずれかに該当する患者.
  1) 大腸粘膜異形成（Dysplasia）を有する患者又は既往のある患者.
  2) 腺腫様大腸ポリープのある患者.
  3) 罹病期間 8 年以上の全大腸炎を有する患者.
  4) 罹病期間 10 年以上の左側大腸炎（病変の範囲が脾彎曲部を越えていないもの）を有する患者.

  ただし，3)又は 4)に該当する患者は登録前 1 年以内に大腸癌スクリーニング検査（大腸内視鏡検査及び生検）が実施され，大腸粘膜異形成（Dysplasia）及び腺腫様大腸ポリープがないことが確認できた場合は登録可能とする.

(9) ストーマ造設患者.

(10) 過去に広範囲の腸管切除術を行った患者（例えば，腸管切除後の結腸が 30cm 未満の患者など）.

(11) 直腸のみ又は結腸の 20 cm 未満に病変範囲が限局している患者.

(12) 過去にインフリキシマブ又は他の生物学的製剤（抗 TNFα 剤，抗 IL-6 剤など）を使用したことがある患者.

(13) 観察期間開始日前 8 週以内に，シクロスポリン，タクロリムス（外用剤除く），メトトレキサート，ミコフェノール酸モフェチル，ミゾリビンなどの免疫調節薬（ただし，アザチオプリン，6-メルカプトプリンは該当しない）を使用した患者.

(14) 観察期間開始日前 4 週以内に血球成分除去療法を実施した患者.

(15) 観察期間開始日前 1 週以内に，以下のいずれかの薬剤・療法を使用又は実施した患者.

1) 中心静脈栄養療法
2) 完全経腸栄養療法［完全経腸栄養療法とは，通常食，低残渣食，半消化態栄養剤の摂取をせず，経腸栄養療法（成分栄養剤，消化態栄養剤）のみで総栄養を摂取するよう治験責任（分担）医師の指示に基づき実施しているものとする.］
3) 絶食［絶食とは，潰瘍性大腸炎治療を目的として治験責任（分担）医師の指示に基づき実施しているものとする.］
4) 輸血
5) ステロイド（注射剤，注腸剤，坐剤，痔軟膏剤）
6) 5-アミノサリチル酸製剤（注腸剤）
7) サラゾスルファピリジン製剤（坐剤）
8) 潰瘍性大腸炎治療を目的としたシプロフロキサシン又はメトロニダゾール
9) 排便回数のコントロールを目的とした止瀉薬や下痢の治療薬（やむを得ない場合の頓用を除く）
10) 下剤（S 状結腸鏡検査などの前処置を除く）

(16) 非ステロイド性消炎鎮痛剤（以下 NSAIDs）（注射剤，経口剤，坐剤）を連用している患者．ただし，有害事象発現時などの一時的な使用（最大 4 週間）や，心臓発作，不安定狭心症や一時的な虚血発作の予防などを目的とする低用量のアスピリンは使用可能とする.

(17) 感染症：

1) 入院加療が必要となる重篤な感染症（活動性肝炎，肺炎，腎盂腎炎など）を合併又は登録前 6 ヶ月以内に既往のある患者.
2) 日和見感染症（サイトメガロウイルス感染症，全身性真菌感染症，ニューモシスティス肺炎，非結核性抗酸菌感染症など）を合併又は登録前 6 ヶ月以内に既往のある患者.
3) 活動性結核を合併している患者.
4) 結核感染の既往のある患者又はその疑いがある患者．ただし，治験薬投与開始日の 3 週間以上前から抗結核薬［原則としてイソニアジド（以下 INH)］を使用する場合は除外基準に該当しない.

［結核感染の疑いがある患者とは，以下のいずれかに該当する患者とする.］
・画像検査で陳旧性肺結核に合致する所見（胸膜癒着像や石灰化のみも含む）のある患者.
・活動性結核患者と接触がある患者.
・クォンティフェロン（以下 QFT）検査又は T-スポット.TB 検査で陽性の患者.
QFT 検査又は T-スポット.TB 検査で判定保留又はツベルクリン反応検査で陽性（BCG 接種の影響を考慮した上で，結核感染がより疑われる場合）の患者については, 他の結核スクリーニング検査（胸部 X 線検査, 胸部 CT 検査及び診察など）により，治験責任（分担）医師が結核感染の疑いを否定できると判断する場合は抗結核薬の使用は必須としない.
なお，画像検査などの結果の評価については，必要に応じて専門医と相談することとした.

5) 活動性 B 型肝炎又は活動性 C 型肝炎を合併している患者．若しくは，B 型肝炎ウイルスキャリアであることが確認されている患者.
6) HIV 感染症が確認されている患者.
7) その他，慢性感染症（慢性腎感染症，気管支拡張症を伴う慢性呼吸器感染症，慢性副鼻腔炎など）を合併している患者.

(18) マウス由来のたん白質（マウス型，キメラ型，ヒト化抗体など）に対する過敏症の既往のある患者.
(19) 脱髄疾患（多発性硬化症など）を合併している患者又はその既往がある患者.
(20) うっ血性心不全を合併している患者.
(21) リンパ腫を含むリンパ増殖性疾患，通常の大きさ又は場所でないリンパ節症のようなリンパ増殖性疾患の徴候，臨床的に意義のある肝腫脹又は脾腫の既往又は合併を有する患者.
(22) 家族歴にリンパ腫又は白血病を有している患者.
(23) 悪性腫瘍を合併している患者又は登録前 5 年以内に悪性腫瘍の既往のある患者.
(24) 登録時に，以下の観察検査 10 項目のうち，いずれか一つでもあてはまる患者．測定値は，同意後かつ登録前 2 週以内に測定された値とする.

1) ヘモグロビン：8.0 g/dL 未満
2) 白血球数：3500×10^6/L 未満
3) 好中球数：1500×10^6/L 未満（桿状核球，分葉核球の場合は，その合計値とする）
4) リンパ球数：500×10^6/L 未満
5) 血小板数：10×10^4/μL 未満
6) AST (GOT)：基準範囲上限の 2 倍超
7) ALT (GPT)：基準範囲上限の 2 倍超
8) ALP：基準範囲上限の 2 倍超
9) HBs 抗原，HBs 抗体，HBc 抗体：陽性

10) HIV 抗体：陽性

(25) ループス様症候群を合併し，かつ抗二本鎖 DNA (dsDNA) 抗体が検査機関の基準値を超える患者.

(26) 重大な疾患を合併している患者（医薬品などの副作用の重篤度分類基準におけるグレード 3（付録 16.1.1a 添付 2）を参考にする）.

(27) 治験薬投与開始前 3 ヶ月以内に生ワクチンを接種した患者.

(28) 治験期間中に継続して採血が実施困難な患者.

(29) 治験薬投与開始日から最終投与 6 ヶ月後まで避妊することに同意が得られない患者.

(30) 妊娠中，授乳中，妊娠している可能性のある女性患者.

(31) 同意取得前 12 週以内に他の治験に参加し，治験薬の投与を受けた患者.

(32) その他，治験責任（分担）医師が本治験の対象として不適格であると判断した患者.

注）期間については，以下のとおり定める．なお，ここには，年，月又は週の最小単位につき例示した.
・観察期間開始日の 1 週以上前は，観察期間開始日の前週の同じ曜日から前とする.
・観察期間開始日前 1 ヶ月は，観察期間開始日の前月の同日とする.
・観察期間開始日前 1 年は，観察期間開始日の前年の同日とする.

【設定根拠】

(1) 被験者の安全性を確保するため，全身症状の悪い潰瘍性大腸炎患者を除外した.

(2) 治験開始前及び治験期間中に潰瘍性大腸炎に対する外科的手術を行うことは本剤の有効性及び安全性の評価に影響を与えることから，手術実施例に加え手術実施の可能性のある患者を除外した.

(3) (17) TA-650 は免疫応答に影響を及ぼす可能性があることから，感染に対する感受性の上昇が考えられるため.

(4) (5) 手術が必要となる可能性や，排便回数や腹痛などの有効性評価に影響を与えるため.

(6) 手術が必要となる可能性があり，有効性及び安全性の評価に影響を与えるため.

(7) 極めて重症度が高く，手術の適応となるため.

(8) (24) (26) (27) 被験者の安全性を確保するため.

(9) ストーマ造設患者は，排便回数や直腸からの出血など有効性評価を適切に実施できないため.

(10) (11) 病変部位が限局している患者は，TA-650 の評価上不適と考えられるため.

(12) 治験薬と同一成分であるインフリキシマブの過去の臨床評価などから本治験の安全性及び有効性に対するバイアスがかかる可能性があること，本剤の血清中濃度や ATI に対する影響の可能性もあることから，設定した．また，他の生物学的製剤も同様に安全性及び有効性の評価に影響を与える可能性があることから，設定した.

(13)～(16) TA-650 の有効性評価に影響を及ぼすと考えられるため.

(18) TA-650 投与による過敏反応が発現する恐れがあるため.

(19) (20) 症状の再燃又は悪化の恐れがあるため.
(21)～(23) TA-650 は免疫応答に影響を及ぼす可能性があり，悪性リンパ腫などの悪性腫瘍の発現リスクを高める可能性があるため.
(25) TA-650 投与後にループス様症候群を疑わせる症状が発現することがあるため.
(28) 治験薬の評価が困難なため.
(29) (30) 生殖発生毒性に対するヒトでの安全性が確立されていないため.
(31) 本治験を倫理的に遂行するため．また，評価の確立していない薬剤は，有効性及び安全性に及ぼす影響が予測不可能であるため.
(32) 本治験を安全及び倫理的に遂行するため.

9.3.3 被験者の治療又は評価の打ち切り

(1) 被験者の中止基準

以下の中止基準に該当する場合は，治験を中止した.

1) 代諾者又は被験者より中止の申し入れがあった場合.
2) 潰瘍性大腸炎の症状増悪のため，治験責任（分担）医師が治験の継続が妥当でないと判断した場合（例えば併用禁止薬剤・療法の併用を行った場合など．ただし，排膿のための切開を行った場合は該当しない）.
3) 有害事象などの発現のため，治験責任（分担）医師が治験の継続を困難と判断した場合．なお，ループス様症候群が認められ，かつ，抗 dsDNA 抗体陽性（抗 dsDNA IgM 抗体 6 U/mL 以上，又は抗 dsDNA IgG 抗体 12 IU/mL 超）となった場合も治験を中止する.
4) 治験薬投与開始後，安全性の確保又は有効性評価の上で，本治験の対象として明らかに不適格であることが判明した場合.
5) その他，治験責任（分担）医師が治験を中止すべきであると判断した場合.

【設定根拠】

治験を倫理的に実施するため，また，被験者の安全性に配慮して設定した.

(2) 中止の手順

治験責任（分担）医師は，評価期間中に治験を中止した場合には，当該被験者に対し適切な措置を講ずるとともに，中止した旨を速やかにモニターに連絡することとした．また，治験責任（分担）医師は，中止時及び最終投与から 8 週後に規定された検査，観察を行うこととした.

治験責任（分担）医師は，中止日，中止理由及びその詳細，中止に至った経緯や実施した処置などについて症例報告書に記録することとした．更に，有害事象発現により治験を中止した場合は，中止に至る原因となった事象名を症例報告書の中止欄に記録する

こととした．なお，中止日とは，中止時の評価を実施した日（中止時評価のための診察を行った日）とするが，中止時評価が行えなかった場合は中止と判断した日とした．

最終投与から8週以内に中止時の規定の観察・検査ができなかった被験者又は中止後に来院のない被験者については，その理由及びその後の経過などについて，文書（封書）又は電話などにより追跡調査を実施し，その内容を症例報告書の中止欄に記録することとした．

治験責任（分担）医師又は治験協力者は，来院しない被験者の症状シートを郵送などにより可能な限り回収することとした．

(3) 治験の中止又は中断

1) 治験の中止又は中断の基準

治験依頼者は次の事例があった場合，実施医療機関全体又は一部の実施医療機関における治験実施継続の可否を検討することとした．

(a) 治験薬の品質，有効性及び安全性に関する事項，その他，治験を適正に行うために重要な情報を知った場合．

(b) 安全性評価委員より，治験中止の勧告を受けた場合．

(c) 治験実施計画書の変更が必要となり，実施医療機関がこれに対応できない場合．

(d) 治験審査委員会の意見に基づく実施医療機関の長からの治験実施計画書などに対する修正の指示があり，治験依頼者がこれを承諾できない場合．

(e) 治験審査委員会の判断に基づき，実施医療機関の長が治験の中止を指示した場合．

(f) 実施医療機関がGCP，本治験実施計画書又は治験の契約に重大な又は継続的な違反を行った場合．

2) 治験依頼者による治験全体の中止又は中断

治験依頼者は，治験全体の中止又は中断を決定した場合，速やかにその旨及びその理由を実施医療機関の長及び規制当局に文書で通知することとした．実施医療機関の長は，治験依頼者より治験を中止又は中断する旨通知を受けた場合，速やかにその旨及びその理由の詳細を治験責任医師及び治験審査委員会に文書で通知することとした．

治験責任医師は，治験依頼者から実施医療機関の長を通じて治験の中止又は中断の通知を受けた場合，被験者に速やかにその旨を通知し，適切な治療を保証することとした．

治験を中止した場合の被験者への対応については，「(2) 中止の手順」に従うこととした．

3) 治験責任医師，治験審査委員会による当該実施医療機関の治験の中止又は中断

治験責任医師自らの判断により治験を中止又は中断した場合には，速やかにその旨及びその理由の詳細を実施医療機関の長に文書で通知することとした．実施医療

機関の長は，治験依頼者及び治験審査委員会に速やかにその旨を文書で通知することとした．

治験審査委員会の判断により治験の中止又は中断を決定した場合には，速やかにその旨及びその理由の詳細を実施医療機関の長に文書で通知することとした．実施医療機関の長は，治験責任医師及び治験依頼者に速やかにその旨を文書で通知することとした．

4) 実施医療機関との契約解除による治験の中止

実施医療機関が，治験期間中に，GCP，本治験実施計画書又は治験の契約に対して，重大又は継続的な違反を行ったことにより治験依頼者が治験を中止した場合，治験依頼者は規制当局に速やかにその旨を報告することとした．

9.4 治療法

9.4.1 治療法

9.4.1.1 治験薬の用法・用量，投与経路

各投与日の体重 1 kg 当たり 5 mg の TA-650 を 1 回の投与量とし，2 時間以上かけて緩徐に点滴静注した．初回投与（0 週），2 週，6 週に投与し，8 週 CAI スコア判定による CAI スコア応答例は，14 週，22 週に治験薬投与を同様に行った．ただし，CAI スコア不応例に対しては 14 週以降の治験薬投与は行わなかった．

9.4.1.2 調整方法

投与当日の被験者の体重（小数点第 1 位を四捨五入した整数値）当たりで計算した必要量を約 250 mL の生理食塩水に希釈して投与することとした．本剤は用時溶解とし，溶解後 3 時間以内に投与を開始した．なお，投与当日の体重が 20 kg 未満の被験者の場合，約 100 mL の生理食塩水に希釈して投与した．

9.4.1.3 治験薬の包装及び表示

(1) 包装

以下に示したとおり，表面ラベルを貼付したバイアルを 1 バイアルずつ箱に詰めたものを使用した．

(2) 表示

1) バイアル上の表示

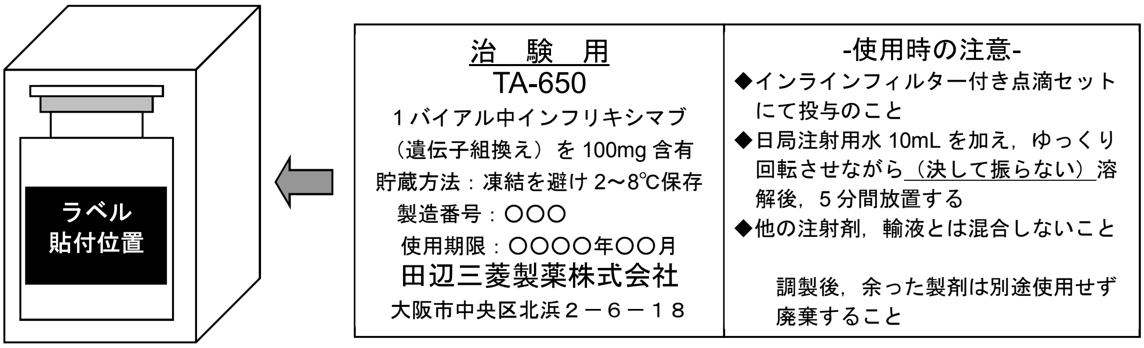

2) 外箱の表示

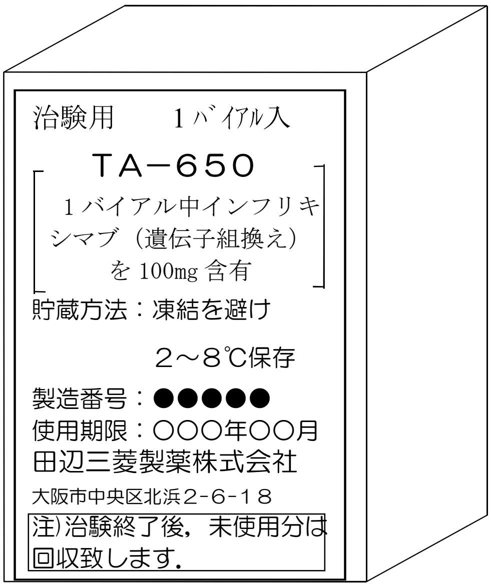

9.4.2 治験薬の同定

9.4.2.1 治験薬の名称等

名称：TA-650

一般名：Infliximab，インフリキシマブ（遺伝子組換え）

9.4.2.2 剤型及び含量

TA-650 100 mg バイアル：

1 バイアル中にインフリキシマブとして 100 mg を含有する凍結乾燥品．用時，日局注射用水で溶解し，日局生理食塩液で希釈して用いる注射剤．

9.4.2.3 製造元及びロット番号

TA-650 100 mg バイアル：110001，120075（ロット番号）
治験薬は，Janssen Biotech, Inc.社より入手した．

9.4.2.4 治験薬の管理

治験依頼者は，実施医療機関との間で治験契約が締結された後，治験薬を交付した．治験薬管理者は，治験依頼者が定める「治験薬管理手順書」（付録 16.1.13a）に従い治験薬を保管・管理し，治験終了後，未使用治験薬，処方後に返却された未調整の治験薬，空箱をモニターに返却した． なお, 治験薬は本治験実施計画書に則った目的以外（他の臨床試験, 動物実験, 基礎実験など）に使用されなかった.

9.4.3 治療群への患者の割付方法

9.4.3.1 治験薬の割付

本治験は，非対照試験であるため該当しない.

9.4.3.2 被験者の登録

被験者登録方法「治験手順のフローチャート（付録 16.1.1a, 添付 3）」の手順及び以下の記載に従って被験者の登録を行った．なお，登録日は適格性を確認した日とした.

同意取得後，治験責任（分担）医師は,「同意取得連絡票（付録 16.1.1a, 添付 4）」を記載し，治験依頼者に FAX にて連絡することとした.

同意取得以降，治験責任（分担）医師は，観察及び検査を実施し，被験者の適格性を確認した後,「登録票（付録 16.1.1a, 添付 5）」に必要事項を記載して，治験依頼者に FAX にて連絡することとした．なお，観察期間の CAI スコア評価日から初回投与まで 1 週間を超える場合は，再度 CAI スコア評価を実施し再登録するものとした．また，この際，パーシャル Mayo スコア，PUCAI スコアの評価も行い，S 状結腸鏡検査についても，できる限り実施することとした.

9.4.4 治験における用量の選択

各投与日の体重 1 kg 当たり 5 mg の TA-650 を 1 回の投与量とし，2 時間以上かけて緩徐に点滴静注した．初回投与（0 週），2 週，6 週に投与し，8 週 CAI スコア判定による CAI スコア応答例は，14 週，22 週に治験薬投与を同様に行った．ただし，CAI スコア不応例に対しては 14 週以降の治験薬投与は行わなかった.

【設定根拠】

国内では，既存治療で効果不十分な中等症から重症の潰瘍性大腸炎の治療として，5 mg/kg を 0，2，6 週に投与し，その後 8 週間隔で投与することが承認されている．また，海外では，小児の潰瘍性大腸炎を対象とした C0168T72 試験と成人を対象とした ACT1 試験の結果から，小児における有効性，安全性プロファイル及び薬物動態は成人と類似していることが示唆されている．そのため，成人の用法・用量と同様に小児の中等症から重症の活動期の潰瘍性大腸炎の治療法として，5 mg/kg を 0，2，6 週に投与し，その後 8 週間隔で投与することが欧米において承認されている．以上のことから，TA-650 5 mg/kg を 0，2，6 週に投与し，その後，14 週，22 週に投与することとした．

9.4.5 各患者の用量の選択及び投与時期

すべての被験者に 5 mg/kg の TA-650 が投与された．各患者の投与時期については 9.4.1.1 治験薬の用法・用量，投与経路に記載した．

9.4.6 盲検化

本治験は，非盲検試験であるため該当しない．

9.4.7 前治療及び併用療法

9.4.7.1 併用禁止薬・療法

以下に示す薬剤・療法のうち(1)～(15)は，観察期間及び有効性評価期間を通じて使用を禁止し，(16)，(17)は観察期間及び評価期間を通じて使用を禁止した．

(1) 市販薬レミケード®，又は他の生物学的製剤（抗 TNFα 剤，IL-6 剤など）

(2) 免疫調節薬（アザチオプリン，6-メルカプトプリンを除く）
シクロスポリン，タクロリムス（外用剤を除く），メトトレキサート，ミコフェノール酸モフェチル，ミゾリビンなどの免疫調節薬（ただし，アザチオプリン，6-メルカプトプリンは該当しない）

(3) ステロイド（注射剤，注腸剤，坐剤，痔軟膏剤）
ステロイド注射剤を投与時反応などの有害事象に対する治療，又は前処置として使用する場合は可能とした．ただし，前処置として使用する場合は有効性評価終了後に実施することとした．

(4) サラゾスルファピリジン製剤（坐剤）

(5) 5-アミノサリチル酸製剤（注腸剤）

(6) 潰瘍性大腸炎に対する抗菌剤（シプロフロキサシン，メトロニダゾール）

ただし，感染症治療など，潰瘍性大腸炎治療以外の目的での一時的な使用（最大 3 週間）は可能とした．

(7) 下剤

ただし, S 状結腸鏡検査又は大腸内視鏡検査やその他検査の前処置としての使用は可能とした．

(8) 止瀉薬，下痢の治療薬

ただし，やむを得ない場合の一時的な使用（およそ 3 日以内の連続使用）は可能としたが，各 CAI スコア評価開始日前 3 日から評価日まで使用を禁止した．

(9) NSAIDs（注射剤，経口剤，坐剤）

ただし，有害事象発現時などの一時的な使用（最大 4 週間）や，心臓発作，不安定狭心症や一時的な虚血発作の予防などを目的とする低用量のアスピリンは使用可能とした．

(10) 中心静脈栄養療法

(11) 完全経腸栄養療法

なお，完全経腸栄養療法とは，通常食，低残渣食，半消化態栄養剤の摂取をせず，経腸栄養療法（成分栄養剤，消化態栄養剤）のみで総栄養を摂取するよう治験責任（分担）医師の指示に基づき実施しているものとした．

(12) 絶食

なお，絶食とは，潰瘍性大腸炎治療を目的として治験責任（分担）医師の指示に基づき実施しているものとした．

(13) 血球成分除去療法

(14) 外科的手術

1) 潰瘍性大腸炎に対する外科的手術

2) その他，術後感染に留意が必要な外科的手術

(15) 輸血

(16) 生ワクチン

(17) TA-650 以外の治験薬

【設定根拠】

(1) 治験薬と同一成分であるインフリキシマブの投与により，本治験における有効性，安全性及び薬物動態は評価不能となるため設定した．また，他の生物学的製剤も同様に安全性及び有効性の評価に影響を与える可能性があることから併用禁止とした．

(2)～(6) 有効性の適正な評価が困難となると考え禁止とした．ただし，シプロフロキサシン，メトロニダゾールについては，被験者の安全面に配慮し，有害事象などの治療のために一時的に使用される場合は可能とした．また，ステロイド（注射剤）については，被験者の安全面に配慮し，投与時反応などの有害事象に対して一時的に使用される場合は使用可能とした．

(7)，(8) CAI スコア，パーシャル Mayo スコア，Mayo スコア，PUCAI スコアの構成項目である排便回数に影響を与えるため，使用禁止とした．しかし，医療実態を考慮に入れ，検査の前処置ややむを得ない場合の一時的使用は可能とした．

(9) 潰瘍性大腸炎を悪化させるとの報告があることから，本剤の有効性評価に影響を与えると考え原則禁止とした．しかし，有害事象発現時や一時的な虚血発作を予防するために使われるような低用量のアスピリンの使用については，被験者の安全性に配慮して使用可能とした．

(10)〜(14)1)，(15) 有効性の適正な評価が困難と考え設定した．

(14)2) 術後の感染症が危惧され，また，有効性評価にも影響することから設定した．

(16) ワクチン接種に対する応答が不明であり，また，生ワクチンによる二次感染の可能性が否定できないため併用禁止とした．

(17) 有効性及び安全性への影響が不明であるため併用禁止とした．

9.4.7.2 併用制限薬・療法

上記「9.4.7.1 併用禁止薬・療法」で規定された以外の薬剤又は治療法は観察期間及び評価期間を通じて併用を可能とした．ただし，ステロイド（経口剤），アザチオプリン，6-メルカプトプリン，5-アミノサリチル酸製剤（経口剤），サラゾスルファピリジン製剤（経口剤）については以下の条件で使用とすることとした．

(1) ステロイド（経口剤）

ステロイド（経口剤）は，観察期間及び有効性評価期間を通じて原則用量一定とし，新たな開始及び増量を禁止した．ただし，医療上やむを得ない理由による減量は可能とした．

治験薬投与開始日の評価以降は，治験責任（分担）医師が潰瘍性大腸炎の臨床的改善を認めた場合，減量を可能とした．ステロイド（経口剤）の減量は，以下に示す減量を最大とし，臨床所見やその経過を十分に勘案し慎重に実施した．

[ステロイド（経口剤）減量]

プレドニゾロン換算 20 mg/日以上：プレドニゾロン換算で最大 10 mg/週ずつ減量

プレドニゾロン換算 10 mg/日以上 20 mg/日未満：プレドニゾロン換算で最大 5 mg/週ずつ減量

プレドニゾロン換算 10 mg/日未満：プレドニゾロン換算で最大 2.5 mg/週ずつ減量

また，このようなステロイド（経口剤）の減量又は中止により症状増悪を認めた場合，これらの症状を改善するために一時的（最大 4 週間）に観察期間の用量を上回って使用することを可能とした．この場合，1 日用量は用量変更後 4 週以内に観察期間の用量又はそれ以下に減量することとした．

原疾患治療以外で医療上やむを得ない場合は，一時的（最大 4 週間）に観察期間の用量を上回って使用することを可能とした．

(2) アザチオプリン，6-メルカプトプリン，5-アミノサリチル酸製剤（経口剤），サラゾスルファピリジン製剤（経口剤）

これらの薬剤は，観察期間及び有効性評価期間を通じて原則用量一定とし，新たな開始及び増量を禁止した．ただし，治験薬投与開始日の評価以降は，原疾患以外の理由（有害事象やその他医療上の理由）により減量が必要となった場合のみ，減量を可能とした．減量後の有効性評価期間中は一定量又は観察期間の用量を上回らない範囲で投与した．

【設定根拠】

これらの経口治療薬は,有効性評価開始前より一定の期間･用量で使用されている場合は,本剤の有効性評価に影響を与えないと考え使用可能とした.

ステロイド治療は，多くの副作用を認めることが知られており，臨床症状の改善を認めた場合，できる限り減量することが望ましいとされている．よって，有効性指標の一つとして,ステロイド使用量を評価することとし,原疾患の症状改善に伴うステロイド（経口剤）の減量・中止を可能とした．なお，減量後に症状が増悪した場合，一時的な増量は可能とした．

9.4.7.3 その他併用薬剤に関する注意事項

(1) ワクチン接種

TA-650 投与期間中の生ワクチン接種は二次感染の危険性があるため，TA-650 投与前 3 ヶ月間に生ワクチンを接種した患者を除外する基準を設けるとともに，評価期間中の生ワクチン接種を禁止とした．また，TA-650 投与期間中に不活化ワクチンを接種する場合には，ワクチン接種に対する応答が不明であることを考慮の上，使用することとした．

これらを踏まえ，登録前に被験者の定期／任意接種のワクチン接種歴やワクチンにて予防可能な感染症の既往歴を確認し，ワクチン接種の必要性について検討することとした．

(2) 結核予防の対応

結核感染の既往のある患者又は疑いのある患者に対して，治験薬投与開始日の 3 週間以上前から評価期間を通じて INH を投与することとした．用量は 10～15 mg/kg を目安として最大量 300 mg/日とした．なお，治験薬投与開始前 3 週以内に副作用などにより INH の使用を中止した場合には治験薬を投与しなかった．治験薬投与開始後に副作用などにより INH の使用ができない場合には，専門医と相談の上対応することとした．

［結核感染の疑いのある患者とは，以下のいずれかに該当する患者とする.］

・画像検査で陳旧性肺結核に合致する所見（胸膜癒着像や石灰化のみも含む）のある患者

・活動性結核患者と接触がある患者

・QFT 検査又は T-スポット.TB 検査で陽性の患者

QFT 検査又は T-スポット.TB 検査で判定保留又はツベルクリン反応検査で陽性（BCG

接種の影響を考慮した上で，結核感染がより疑われる場合）の患者については，他の結核スクリーニング検査（胸部 X 線検査，胸部 CT 検査及び診察など）により，治験責任（分担）医師が結核感染の疑いを否定できると判断する場合は抗結核薬の使用は必須としないこととした．

なお，画像検査などの結果の評価については，必要に応じて専門医と相談することとした．

9.4.7.4 併用薬及び併用療法の記載

治験責任（分担）医師は，観察期間から有効性評価期間終了までに併用した薬剤及び療法の以下の内容について症例報告書の併用薬及び併用療法欄に記録した．

(1) 併用薬：薬剤名，使用理由，用量変更理由，1 日投与量，投与経路，投与期間

(2) 潰瘍性大腸炎治療を目的とした処置・療法：処置・療法，開始日，終了日

以下の薬剤は症例報告書に記録しないこととした．

・注射剤の溶解を目的とした生理食塩液など

・点滴の際の補液として使用された生理食塩液や注射用水など

・有害事象の処置として実施される手術の処置薬（麻酔薬，輸液，生理食塩液，消毒薬など）．ただし，併用禁止薬剤及び併用制限薬剤は症例報告書に記録した．

なお，治験協力者は，原資料に記録されている項目に限り症例報告書に転記できることとした．

9.4.8 治療方法の遵守

(1) 生活指導

治験責任（分担）医師又は治験協力者は，以下の点に注意して，代諾者及び被験者に生活指導を行った．

1) 指定された日に，診察・検査を受けること．もし指定された日に来院できない場合は，必ず治験責任（分担）医師又は治験協力者に連絡しその指示に従うこと．
2) 治験参加カードを携帯し，他病院又は他科診療時には提示すること．また，この治験以外の担当医師から処方されている薬剤や薬局で購入した薬剤を使用している場合でも，必ず治験責任（分担）医師又は治験協力者に申し出ること．更に治験中に新たに薬剤を使用する場合やインフルエンザワクチンなどのワクチン接種（特に生ワクチンは併用禁止とされている点に注意すること．）を受ける場合は，事前に必ず治験責任（分担）医師又は治験協力者に申し出ること．
3) 生活スタイル（特に食生活や健康食品）を可能な限り変化させないこと．
4) 感染症の徴候など，体に異常を感じた場合（軽い風邪であっても）や妊娠したことが分かった場合，潰瘍性大腸炎の症状（排便回数，血便，腹痛など）が明らかに悪

化した場合は，速やかに治験責任（分担）医師又は治験協力者に連絡し，受診の必要性を確認すること．

5) 登録後，最終投与 6 ヶ月後まで避妊すること．

(2) 症状シートの記入指導

治験責任（分担）医師又は治験協力者は，同意取得後，被験者又は代諾者に症状シートを渡した．その際，被験者又は代諾者に記入方法などについて説明し，下記の項目について指定された来院日の 7 日前から毎日記入し，来院日には必ず持参するよう指導した．

1) 症状シート記入項目

・1 日の排便回数

・血便

・便秘又は下痢の治療薬の使用有無

2) 症状シート記入方法

・指定された来院日の 7 日前から毎日記入すること．

・ボールペンなど，簡単に消えない筆記用具で記入すること．

・症状シートの記入事項を変更又は修正する時は，訂正前の記入内容が判読できるように訂正箇所に二重線を引くこと．

・実施医療機関記入欄には記入しないこと．

・便を伴わず出血のみの場合でも，排便回数に数えること．

・便意を催したが実際は排便しなかった場合は，排便回数に数えないこと．

・しぶり腹や不完全な排便に関連して，いくつかの小さな糞便が非常に短期間に排出された場合は 1 回の排便とすること．

9.5 有効性及び安全性の項目

9.5.1 有効性及び安全性の評価項目及びフローチャート

9.5.1.1 観察，検査，調査項目及び時期（スケジュール表）

本治験は，以下のフローチャート及び表 9.5－1 に従い実施した．

治験手順のフローチャート

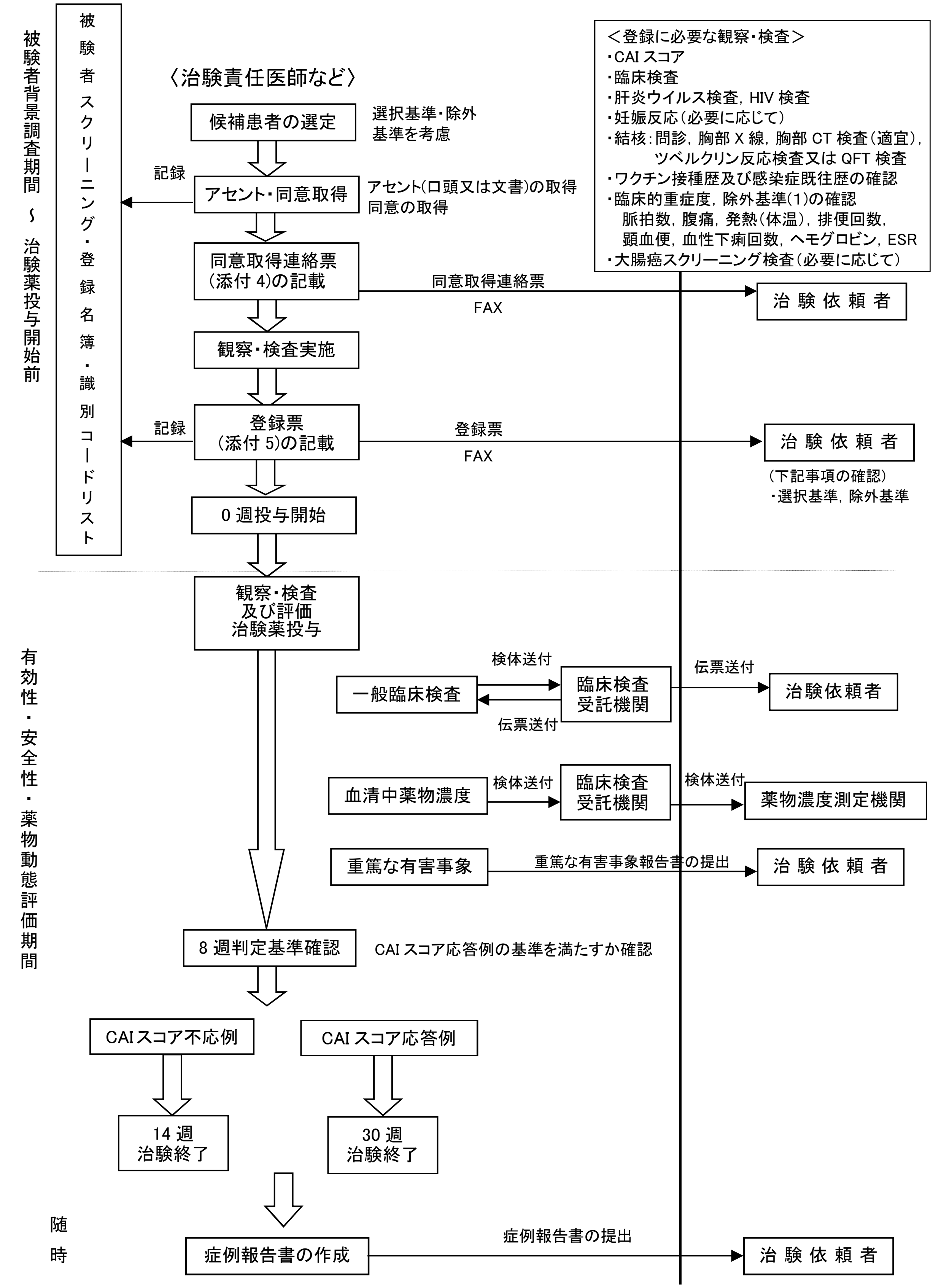

登録に際し，治験責任（分担）医師は，適格性を確認するために以下の検査・観察を行った．

(1) 臨床検査

治験責任（分担）医師は，同意後かつ登録前 2 週以内に下記の臨床検査項目を測定した．また，検査値が複数ある場合は，投与開始時に集中測定される検査値を除き，登録日から直近の検査値を用いた．

ヘモグロビン，白血球数，好中球数*，リンパ球数，血小板数，AST（GOT），ALT（GPT），ALP，HBs 抗原，HBs 抗体，HBc 抗体，HIV 抗体，妊娠検査（生理的に妊娠可能と判断される女性のみ）

* 好中球数：桿状核球・分葉核球の場合はその合計値とする．

(2) 結核検査

治験責任（分担）医師は，同意後かつ登録前 4 週以内に，問診，胸部 X 線検査，胸部 CT 検査（適宜），ツベルクリン反応検査又は QFT 検査（T-スポット.TB 検査も可）を実施し，結核を始めとした呼吸器感染症の有無を確認した．また，これらが複数回検査されている場合には，登録日から直近のものを用いた．

結核検査により，結核感染の既往のある患者又はその疑いがある患者に対して，治験責任(分担)医師は治験薬投与開始日の 3 週間以上前から抗結核薬[原則として INH]を投与した．

[結核感染の疑いのある患者とは，以下のいずれかに該当する患者とする.]

・画像検査で陳旧性肺結核に合致する所見（胸膜癒着像や石灰化のみも含む）のある患者
・活動性結核患者と接触がある患者
・QFT 検査又は T-スポット.TB 検査で陽性の患者

QFT 検査又は T-スポット.TB 検査で判定保留又はツベルクリン反応検査で陽性（BCG 接種の影響を考慮した上で，結核感染がより疑われる場合）の患者については，他の結核スクリーニング検査（胸部 X 線検査，胸部 CT 検査及び診察など）により，治験責任（分担）医師が結核感染の疑いを否定できると判断する場合は抗結核薬の使用は必須としない．

なお，画像検査などの結果の評価については，必要に応じて専門医と相談することとする．

(3) ワクチン接種

治験責任（分担）医師は，登録前に被験者の定期／任意接種のワクチン接種歴やワクチンにて予防可能な感染症の既往歴を確認し，ワクチン接種の必要性について検討することとした．なお，被験者のワクチン接種歴及び感染症の既往歴の確認は少なくとも登録日までに実施した．

(4) 臨床的重症度，除外基準（1）に該当するか否かの確認

治験責任（分担）医師は，登録時に，脈拍数，ヘモグロビン，ESR，排便回数，顕血便，血性下痢回数，発熱（体温），腹痛を確認し，これより 9.3.2 除外基準（1）

に抵触しないこと及び原疾患の臨床的重症度を評価した.

(5) 大腸癌スクリーニング検査

治験責任（分担）医師は，以下のいずれかに該当する場合は，同意後かつ観察期間開始前4日までに大腸癌スクリーニング検査（大腸内視鏡検査及び生検）を実施し，大腸粘膜異形成（Dysplasia）及び腺腫様大腸ポリープがないことを確認した.

・罹病期間8年以上の全大腸炎を有し，登録前1年以内に大腸癌スクリーニング検査を実施していない患者.

・罹病期間10年以上の左側大腸炎を有し，登録前1年以内に大腸癌スクリーニング検査を実施していない患者.

なお，大腸癌スクリーニング検査のために実施する大腸内視鏡検査は，Mayo スコア評価のために実施するS状結腸鏡検査として兼ねることを可能とした.

表 9.5−1　治験スケジュール

		同意日	観察期間	評価期間 0週	2週	6週	8週	10週	14週	18週	22週	26週	30週	不応例 10週	14週	中止した被験者 中止日	最終投与8週後
評価日の許容範囲（日）		—		—	±3	±3	±3	±7	±7	±7	±7	±7	±7	±7	±7	—	±7
文書同意取得		●															
登録時に必要な観察検査項目[*1]			●														
背景調査			●														
治験薬投与の許容範囲（日）				—	±3	±3			±7		±7						
治験薬投与				●	●	●			●		●						
身長・体重				●	●	●	●	●	●	●	●	●	●			●	
有効性	CAI スコア[*2]		●		●	●	●	●	●	●	●	●	●			●[a]	
	パーシャル Mayo スコア[*3]		●		●	●	●	●	●	●	●	●	●			●[a]	
	S 状結腸鏡検査[*4]		●[b]										●[b,d]			●[a,b,d]	
	PUCAI スコア[*5]		●		●	●	●	●	●	●	●	●	●			●[a]	
安全性	自覚症状・他覚所見			←→	←→	←→	←→	←→	←→	←→	←→	←→	←→	←→	←→	←→	←→
	血圧･脈拍数･体温[*6]			●	●	●			●		●						
	一般臨床検査[*7]			●	●	●	●	●	●	●	●	●	●	●	●	●	●
	免疫血清学的検査[*8]			●					●				●		●	●	●
TNFα，IL-6[*9]				●													
CRP[*10]				●	●	●	●	●	●	●	●	●	●			●[a]	
胸部 X 線検査，胸部 CT 検査（適宜）			●						●				●		●	●	
薬物動態	血清中インフリキシマブ濃度[*11]			●[c]	●	●	●	●	●[c]	●	●[c]	●	●	●	●	●	●
	抗インフリキシマブ抗体			●					●		●		●		●	●	●

治験薬が投与される評価日は，治験薬投与に先立ち評価及び検査を実施した．

a：CAI スコア不応例が 8 週の評価後に中止した場合は不要とした．

b：できる限り実施した．ただし，観察期間（原則，登録以降，治験薬投与開始までに実施した．やむを得ない場合，治験薬投与開始日から 2 週以内かつ観察期間開始前 4 日までに実施することを可能とした）に実施できなかった場合，30 週又は中止時は実施不要とした．

c：TA-650 投与直前及び投与終了 1 時間後の合計 2 回採血を行った．

d：S 状結腸鏡検査の許容範囲は-13～+14 日とした．

（例）登録時及び治験薬投与開始までに必要な検査・観察（CAI スコア評価日と登録日が同一の場合）

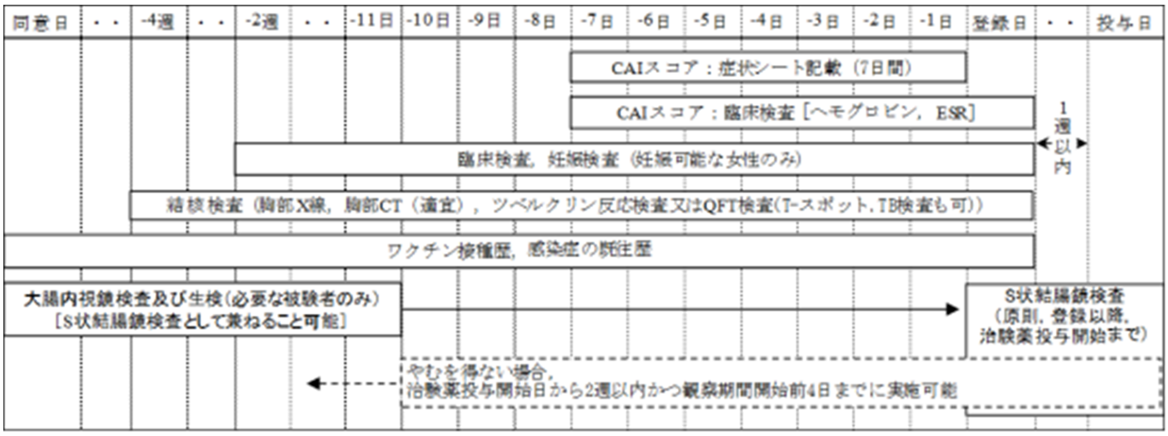

＊1　登録時に必要な観察検査項目：

潰瘍性大腸炎の活動性	CAI スコア
臨床検査	[同意後かつ登録前 2 週以内に実施]<br>ヘモグロビン，白血球数，好中球数*，リンパ球数，血小板数，AST（GOT），ALT（GPT），ALP，HBs 抗原，HBs 抗体，HBc 抗体，HIV 抗体，妊娠検査（生理的に妊娠可能な女性のみ）<br>＊ 好中球数：桿状核球・分葉核球の場合はその合計値とした
結核	[同意後かつ登録前 4 週以内に確認]<br>問診，胸部 X 線検査，胸部 CT 検査（適宜），ツベルクリン反応検査又は QFT 検査（T-スポット.TB 検査も可）
ワクチン接種歴<br>感染症の既往歴	[登録前に確認]<br>過去のワクチンの接種歴及び感染症の既往歴の確認
臨床的重症度及び除外基準(1)の確認	[登録時に以下の検査・観察により判定]<br>脈拍数，腹痛，発熱（体温），排便回数，顕血便，血性下痢回数，<br>ヘモグロビン，ESR
大腸癌スクリーニング検査（必要な被験者のみ）	[同意後かつ観察期間開始前 4 日までに実施]<br>大腸内視鏡検査及び生検

・観察期間の CAI スコア評価日から 1 週以内に治験薬投与を開始した．観察期間の CAI スコア評価日から初回投与まで 1 週間を超える場合には，再度 CAI スコアの評価を実施し再登録した．
・検査値が複数ある場合には，投与開始時に集中測定される検査値を除き，登録日から直近の検査値を用いた．
・胸部 X 線検査，胸部 CT 検査，ツベルクリン反応検査，QFT 検査又は T-スポット.TB 検査は，同意後に複数回確認されている場合には，登録日から直近のものとした．
・ツベルクリン反応検査又は QFT 検査（T-スポット.TB 検査も可）のどちらかは必ず実施した．また，ツベルクリン反応検査結果は BCG 接種の影響を考慮して判断した．
・大腸癌スクリーニング検査は，除外基準(8)に基づき検査が必要な被験者のみ実施した．なお，大腸癌スクリーニング検査のために実施する大腸内視鏡検査は，Mayo スコア評価のために実施する S 状結腸鏡検査として兼ねることを可能とした．

＊2　CAI スコア算出に必要な検査，観察項目：

検査（臨床検査など）	[評価日に実施]　体温<br>[評価日前 7 日から評価日までに実施]　ヘモグロビン（院内），ESR（院内）
臨床症状（観察・問診）	医師の症状アセスメント，腹痛，腸管外合併症
症状シート<br>（被験者又は代諾者が記入）	1 週間の排便回数，血便（1 週間平均で）

・ヘモグロビン及び ESR については，CAI スコア評価前 7 日から評価日までに院内で測定された値を用いた．
・治験薬が投与される評価週については，該当週の治験薬投与前に評価及び検査を実施した．
・ヘモグロビン又は ESR が許容範囲内で複数回測定された場合は，CAI スコア評価日から直近の測定値を用いた．

＊3，＊4　パーシャル Mayo スコア及び Mayo スコア算出に必要な検査，観察項目：

臨床症状（観察・問診）	医師による全般評価
症状シート<br>（被験者又は代諾者が記入）	排便回数，直腸からの出血
S 状結腸鏡検査	内視鏡所見（Mayo スコアのみ）

・S 状結腸鏡検査は Mayo スコアの算出に用いた．
・観察期間の S 状結腸鏡検査は，原則，登録以降，治験薬投与開始までに実施した．やむを得ない場合，治験薬投与開始日から 2 週以内かつ観察期間開始前 4 日までに実施することを可能とした．
・30 週の S 状結腸鏡検査の許容範囲は-13 日～+14 日としたが，30 週の有効性評価に対する影響を考慮し，CAI スコア症状シート評価期間中の S 状結腸鏡検査の実施はできるだけ避けた．

＊5　PUCAI スコア算出に必要な観察項目：腹痛，直腸からの出血，便の硬さ，24 時間あたりの排便回数，夜間排便，活動性レベルの 6 項目について，被験者より聴取した．

＊6　血圧・脈拍数・体温：投与開始直前，投与中は 30 分ごと，投与終了から 2 時間までは 30 分ごとに測定した．終了時とその直前の測定との間隔が 15 分以上の場合は，投与終了時にも測定した．

＊7　一般臨床検査：臨床検査受託機関にて測定した．

血液学的検査	赤血球数，ヘモグロビン，ヘマトクリット値，白血球数，<br>白血球分画（好中球，好酸球，好塩基球，単球，リンパ球），血小板数
血液生化学的検査	AST（GOT），ALT（GPT），ALP，LDH，γ-GTP，総蛋白，アルブミン，<br>総コレステロール，総ビリルビン，BUN，血清クレアチニン，<br>血清電解質（Na，K，Cl）
尿検査（定性）	蛋白，糖，潜血，ウロビリノーゲン

＊8　免疫血清学的検査：抗核抗体，抗 ds DNA 抗体（抗 ds DNA IgG 抗体，抗 ds DNA IgM 抗体）は，臨床検査受託機関にて集中測定を行った．

＊9　TNFα，IL-6：臨床検査受託機関にて集中測定を行った．

＊10　CRP：臨床検査受託機関にて集中測定を行った．

＊11　血清中インフリキシマブ濃度：治験薬投与日に該当する場合は，投与に先立ち採血を行った．
また，投与開始日，14 週，22 週については，投与前及び投与終了 1 時間後の合計 2 回採血した．

9.5.1.2　被験者特性の調査項目

(1)　被験者背景

治験責任（分担）医師は，以下の被験者の背景因子を調査し，その内容を症例報告書に記録した．なお，原資料から転記可能な項目は，治験協力者が症例報告書に転記できることとした．

1)　性別
2)　生年月日（西暦）
3)　身長（単位 cm，小数点第 1 位を四捨五入して整数とした）
4)　体重（単位 kg，測定可能な桁数までとした）
5)　選択基準（既存治療抵抗性）の確認
6)　潰瘍性大腸炎の発症時期（初発時期）
7)　病型（左側大腸炎，全大腸炎）
8)　臨床的重症度（軽症，中等症，重症）
9)　臨床経過（再燃寛解型，慢性持続型，急性劇症型（急性電撃型），初回発作型）
10)　病気（潰瘍性大腸炎）になる前の 1 日排便回数*[1]
11)　過去に潰瘍性大腸炎治療のために使用した経験のある薬剤・療法*[2]
12)　潰瘍性大腸炎に対する腸切除
13)　潰瘍性大腸炎に対する処置，手術歴（腸切除を除く）
14)　合併症*[3]
15)　アレルギー歴

16) 結核検査

問診（問診日，所見），胸部 X 線検査（検査日，所見），胸部 CT 検査（検査日，所見），ツベルクリン反応検査（判定日，発赤径，硬結径），QFT 検査又は T-スポット.TB 検査（採血日，測定値，判定結果），投与開始日の 3 週間以上前からの抗結核薬投与の有無

17) ワクチン接種歴

18) 感染症の既往歴

調査時期：登録時（ただし，合併症の調査時期は投与開始時とした.）

*1：原則整数とするが，やむを得ず幅がある場合はその最小値と最大値の平均値を用いた.

*2：過去に潰瘍性大腸炎治療のために使用した経験のある薬剤・療法については，登録前 1 年以内にシクロスポリン不応例，タクロリムス不応例，血球成分除去療法不応例の該当の有無を症例報告書に記録した.

*3：合併症については，投与開始時における合併症の有無を症例報告書に記録し，有の場合にはその内容も合わせて記録することとした.

9.5.1.3　有効性の評価

9.5.1.3.1　有効性評価項目

(1) CAI スコア
(2) CAI スコア寛解
(3) パーシャル Mayo スコア
(4) Mayo スコア
(5) Mayo スコア改善
(6) Mayo スコア寛解
(7) 粘膜治癒
(8) PUCAI スコア
(9) PUCAI スコア寛解
(10) PUCAI スコア変化量 20 ポイント以上減少
(11) ステロイド使用量
(12) ステロイド離脱

9.5.1.3.2　症状シートの内容確認

CAI スコア，パーシャル Mayo スコア，Mayo スコア及び PUCAI スコアの評価実施に先立ち，治験責任（分担）医師又は治験協力者は，以下に定める各評価時期に，症状シートを被験者又は代諾者から回収し，内容の確認及び実施医療機関記入欄への記載を行った．症状シ

ートについては，原本を実施医療機関にて保存し，写しを治験依頼者に提出した．なお，CAI スコア不応例は 8 週で評価を終了した．また，CAI スコア不応例が 8 週の評価後に中止した場合，中止時の評価は不要とした．

(1) 被験者，代諾者記入欄

治験責任（分担）医師又は治験協力者は，登録時，投与 2，6，8，10，14，18，22，26，30 週，中止時の診察時に，被験者，代諾者記入欄の内容に漏れがないことを確認した．記入内容に不備があった場合，治験責任（分担）医師は，被験者又は代諾者に症状シートへの追記を要請した．症状シートに訂正があった場合には，治験責任（分担）医師は，被験者又は代諾者に確認し，訂正印と日付を記載した．

(2) 実施医療機関記入欄

治験責任（分担）医師又は治験協力者は，登録時，投与 2，6，8，10，14，18，22，26，30 週，中止時の診察時に，便秘又は下痢の治療薬が使用されている場合は，使用した薬剤について被験者又は代諾者から聴取し，その薬剤名を記載した．また，S 状結腸鏡検査などの前処置及び実施の有無を症状シートの実施医療機関記入欄に記載した．

治験責任（分担）医師又は治験協力者は，症状シートの記入内容から以下の基準に従って有効性評価に用いるか否かを判定し，実施医療機関記入欄に記載した．

［評価除外日］*

・便秘や下痢の治療薬の使用日

・強力な止瀉作用を有する薬剤（塩酸ロペラミド，アヘンアルカロイド，硫酸アトロピン含有製剤など）使用日及び使用後 2 日間（計 3 日間）

・S 状結腸鏡検査などの前処置として排便回数に影響を与える薬剤など（下剤など）を使用した日

・大腸内視鏡検査又は S 状結腸鏡検査の実施日及び実施後 3 日間（計 4 日間）

*：CAI スコア（排便回数，血便（1 週間平均で）），パーシャル Mayo スコア（排便回数，直腸からの出血），PUCAI スコアの評価期間から除外する日

(3) 治験責任（分担）医師確認欄

治験責任（分担）医師は，登録時，投与 2，6，8，10，14，18，22，26，30 週，中止時に，症状シートの記入内容について問題がないことを確認した上で署名を記載した．

9.5.1.3.3 CAI スコア

CAI スコアは，1 週間の排便回数，血便（1 週間平均で），医師の症状アセスメント，腹痛，潰瘍性大腸炎による体温上昇，腸管外合併症，臨床検査（ヘモグロビン及び ESR）の 7 項目におけるそれぞれのスコアの合計（0～29）とした．

治験責任（分担）医師は，登録時，投与 2，6，8，10，14，18，22，26，30 週，中止時に，

診察，検査［臨床検査（ヘモグロビン及びESR：院内），体温］，症状シートに基づく評価を行い症例報告書に記録した．

評価日が治験薬投与日の場合は，治験薬投与に先立ち，所定の検査・観察を行った．また，評価日がS状結腸鏡検査日の場合は，S状結腸鏡検査の実施前に所定の検査・観察を行った．なお，CAIスコア不応例は8週の評価で終了した．また，CAIスコア不応例が8週の評価後に中止した場合，中止時の評価は不要とした．

治験責任（分担）医師が評価した各スコアが診療記録などの原資料に記録されている場合は，治験協力者が各スコアを症例報告書に転記できることとした．

(1) No. of stools weekly（1週間の排便回数）

治験責任（分担）医師は，各評価日において，「9.5.1.3.2 症状シートの内容確認」にて定められた評価除外日を除いた評価日前7日間（CAIスコア症状シート評価期間）の排便回数から，以下の変換表に従いスコアを評価して症例報告書に記録した．なお，症状シートの評価期間が7日間に満たない場合，4日以上の評価があれば，計算式：［症状シート評価期間（N日分）の排便回数の和］×7/N により換算してスコアを評価して症例報告書に記録した．

症状シート参照箇所：排便回数		スコア
症状シート評価期間（7日間）の排便回数の和	18回未満	0
	18回以上 35回以下	1
	36回以上 60回以下	2
	61回以上	3

(2) Blood in stools (based on weekly average) （血便（1週間平均で））

治験責任（分担）医師は，各評価日において，「9.5.1.3.2 症状シートの内容確認」にて定められた評価除外日を除いた評価日前7日間（CAIスコア症状シート評価期間）の血便から，以下の変換表に従いスコアを評価して症例報告書に記録した．なお，症状シートの評価期間が7日間に満たない場合，4日以上の評価があれば，以下の変換表に準じてスコアを評価して症例報告書に記録した．

症状シート参照箇所：血便	スコア	
症状シート評価期間（7日間）のすべてにおいて「0」	なし	0
その他の場合	少量：明らかな粘血便ではないが，肉眼的に血液の混入が確認できる	2
症状シート評価期間（7日間）のうち4日以上（半分以上）において，「2」又は「3」	粘血便がある	4

(3) Investigator's global assessment of symptomatic state（医師の症状アセスメント）
治験責任（分担）医師は，各評価日に被験者を診察し，評価日前 7 日間の状態などについて総合的に以下の 4 段階でスコアを評価して症例報告書に記録した.

0 = Good （潰瘍性大腸炎による症状がない）
1 = Average （例えば，軽度の症状はあるが，勤務，勉学，家事などの日常生活に差し支えない程度）
2 = Poor （例えば，症状があり，通勤，通学，家事などの生活活動が制限される程度）
3 = Very poor（例えば，症状があり，入院安静を要する程度）

(4) Abdominal pain/cramps（腹痛）
治験責任（分担）医師は，各評価日に被験者を診察し，評価日前 7 日間の腹痛について被験者より聴取した内容から以下の 4 段階でスコアを評価して症例報告書に記録した.

0 = None （なし）
1 = Mild （時々気になる程度）
2 = Moderate（いつも気になる程度）
3 = Severe （がまんできない程度）

(5) Temperature due to colitis (℃) （潰瘍性大腸炎による体温上昇）
治験責任（分担）医師は，各評価日に被験者の体温を測定し，症状・所見と勘案して以下の 2 段階でスコアを評価して症例報告書に記録した.

0 = 37-38（38 度以下）
3 = >38（38 度超）

(6) Extraintestinal manifestations（腸管外合併症）
治験責任（分担）医師は，各評価日に腸管外合併症（虹彩炎，結節性紅斑，関節炎）の有無を調査し，症例報告書に記録した．スコアはそれぞれの腸管外合併症のスコアを合計したものとした（0～9）.

3 = Iritis （虹彩炎）
3 = Erythema nodusum （結節性紅斑）
3 = Arthritis （関節炎）

(7) Laboratory findings（臨床検査）
治験責任（分担）医師は，各評価日において，評価日前 7 日から評価日までに ESR 及びヘモグロビンを，以下のカテゴリーに従いスコアを評価して症例報告書に記録した．観察期間及び有効性評価期間を通じて，ESR，ヘモグロビンの測定は院内で実施することとし変更しなかった．複数回実施された場合は，CAI スコア評価日から直近の測定値を用いた.

1 = ESR > 50 mm in 1st h （ESR > 50 mm/hr）
2 = ESR > 100 mm in 1st h（ESR > 100 mm/hr）

4 = Haemoglobin < 100 g/L（Hb < 10 g/dL）

ヘモグロビンの測定結果が 10 g/dL 未満の場合，ESR の測定結果にかかわらず，スコアは 4 とした．また，ESR の測定結果が 50 mm/hr 以下で，かつ，ヘモグロビンの測定結果が 10 g/dL 以上の場合，スコアは 0 とした．

9.5.1.3.4 パーシャル Mayo スコア及び Mayo スコア

Mayo スコアは，4 つのサブスコア（排便回数，直腸からの出血，医師による全般評価，内視鏡所見）を 0～3 の 4 段階で評価したものであり，これら 4 つのサブスコアの合計（0～12）とした．また，排便回数サブスコア，直腸からの出血サブスコア，医師による全般評価サブスコアの合計（0～9）をパーシャル Mayo スコアとした．

治験責任（分担）医師は，登録時，投与 2，6，8，10，14，18，22，26，30 週，中止時に，パーシャル Mayo スコア算出のために診察，症状シートに基づく評価を行った．また，観察期間（原則，登録以降，治験薬投与開始までに実施した．やむを得ない場合，治験薬投与開始日から 2 週以内かつ観察期間開始前 4 日までに実施することを可能とした），30 週，中止時に，Mayo スコア算出のために S 状結腸鏡検査を可能な限り行った．これらの評価を症例報告書に記録した．

評価日が治験薬投与日の場合は，治験薬投与に先立ち，所定の検査・観察を行った．また，評価日が S 状結腸鏡検査日の場合は，S 状結腸鏡検査の実施前に所定の観察を行った．なお，CAI スコア不応例は 8 週の評価で終了した．また，CAI スコア不応例が 8 週の評価後に中止した場合，中止時の評価は不要とした．

治験責任（分担）医師が評価した各サブスコアが診療記録などの原資料に記録されている場合は，治験協力者が各サブスコアを症例報告書に転記できることとした．

(1) Stool frequency（排便回数）

治験責任（分担）医師は，各評価日において，「9.5.1.3.2 症状シートの内容確認」にて定められた評価除外日を除いた評価日前 3 日間（パーシャル Mayo スコア症状シート評価期間）の排便回数と病気（潰瘍性大腸炎）になる前の 1 日排便回数から，以下の変換表に従いスコアを評価して症例報告書に記録した．

症例報告書：スコア		症状シート参照箇所：排便回数	
0	Normal number stools for this patient<br>［病気（潰瘍性大腸炎）になる前の 1 日排便回数と同程度］	0.5 未満	［(3 日間の排便回数の和）／3］－［病気（潰瘍性大腸炎）になる前の 1 日排便回数］
1	1-2 stools more than normal<br>［病気（潰瘍性大腸炎）になる前の 1 日排便回数より 1-2 回多い］	0.5 以上<br>2.5 未満	
2	3-4 stools more than normal<br>［病気（潰瘍性大腸炎）になる前の 1 日排便回数より 3-4 回多い］	2.5 以上<br>4.5 未満	
3	5 or more stools more than normal<br>［病気（潰瘍性大腸炎）になる前の 1 日排便回数より 5 回以上多い］	4.5 以上	

病気（潰瘍性大腸炎）になる前の 1 日排便回数は，被験者背景として症例報告書に記録した値を用いた．また，病気（潰瘍性大腸炎）になる前の 1 日排便回数は，原則整数とするが，やむを得ず幅がある場合はその最小値と最大値の平均値を用いた（例：”1～2” の場合は 1.5 として記録した）.

(2) Rectal bleeding（直腸からの出血）

治験責任（分担）医師は，各評価日において，「9.5.1.3.2 症状シートの内容確認」にて定められた評価除外日を除いた評価日前 3 日間（パーシャル Mayo スコア症状シート評価期間）の血便から，最もひどい（大きい）スコアを症例報告書に記録した.

0 = No blood seen
［血液なし］

1 = Streaks of blood with stool less than half the time
［少量の血液，排便回数の半分以下］

2 = Obvious blood with stool most of the time
［はっきりした血液，ほぼ毎回］

3 = Blood alone passed
［ほぼ血液ばかり］

(3) Physician’s global assessment（医師による全般評価）

治験責任（分担）医師は，各評価日において，その他のサブスコア及び問診（被験者の訴え，全身状態など），その他所見などから総合的に以下の 4 段階で評価し，スコアを症例報告書に記録した.

0 = Normal ［正常と区別がつかない状態（完全な寛解期）］

1 = Mild disease ［軽度の活動期（軽症）］

2 = Moderate disease ［中等度の活動期（中等症）］

3 = Severe disease ［高度の活動期（重症）］

(4) Findings of endoscopy（内視鏡所見）

治験責任（分担）医師は，観察期間（原則，登録以降，治験薬投与開始までに実施した．やむを得ない場合，治験薬投与開始日から 2 週以内かつ観察期間開始前 4 日までに実施することを可能とした），30 週，中止時にできる限り S 状結腸鏡検査を実施し，内視鏡写真アトラス（付録 16.1.13c, 別紙 5）を目安として，内視鏡所見を以下の 4 段階でスコアを評価して症例報告書に記録した．なお，観察期間に S 状結腸鏡検査を実施できなかった場合には 30 週又は中止時の実施は不要とした．

0 = Normal or inactive disease
［正常又は寛解期粘膜］
1 = Mild disease (erythema, decreased vascular pattern, mild friability)
［軽症（発赤，血管透見の減少，軽度の脆弱性）］
2 = Moderate disease (marked erythema, absent vascular pattern, friability, erosions)
［中等症（著明な発赤，血管透見の消失，脆弱性，びらん）］
3 = Severe disease (spontaneous bleeding, ulceration)
［重症（自然出血，潰瘍）］

9.5.1.3.5 PUCAI スコア

PUCAI スコアは，腹痛，直腸からの出血，便の硬さ，24 時間あたりの排便回数，夜間排便，活動性レベルの 6 項目におけるそれぞれのスコアの合計（0～85）とした．

治験責任（分担）医師は，登録時，投与 2，6，8，10，14，18，22，26，30 週，中止時に，被験者より聴取して評価を行い症例報告書に記録した．

評価日が治験薬投与日の場合は，治験薬投与に先立ち，評価を実施した．また，評価日が S 状結腸鏡検査日の場合は，S 状結腸鏡検査の実施前に評価を行った．なお，CAI スコア不応例は 8 週の評価で終了した．また，CAI スコア不応例が 8 週の評価後に中止した場合，中止時の評価は不要とした．

治験責任（分担）医師が評価した各項目のスコアが診療記録などの原資料に記録されている場合は，治験協力者が PUCAI スコアを症例報告書に転記できることとした．

なお，PUCAI スコアは下記の規定に従い，被験者からの聴取内容に基づいて，6 項目の評価を行うこととした．

・各評価日において，「9.5.1.3.2 症状シートの内容確認」にて定められた評価除外日を除いた評価日前 2 日間の 1 日平均とした．
・臨床症状が急速に変化した場合は，最新の 24 時間の状態を考慮した．

(1) Abdominal pain（腹痛）

0 = No pain（なし）
5 = Pain can be ignored（がまんできる痛み）
10 = Pain cannot be ignored（がまんできない痛み）

(2) Rectal bleeding（直腸からの出血）
0 = None（なし）
10 = Small amount only, in less than 50% of stools（ほんの少量，排便回数の半分未満）
20 = Small amount with most stools（少量，ほぼ毎回の排便で）
30 = Large amount (>50% of the stool content)（大量（1 回の便の半分を超える量））
・ほぼ毎回の排便に大量の血液が存在している場合は“Large amount”を選択した．

(3) Stool consistency of most stools（便の硬さ）
0 = Formed（有形便）
5 = Partially formed（一部有形便）
10 = Completely unformed（完全に形を成さない便）

(4) Number of stools per 24 hours（24 時間あたりの排便回数）
0 = 0-2（0～2 回）
5 = 3-5（3～5 回）
10 = 6-8（6～8 回）
15 = >8（8 回を超える）
・しぶり腹や不完全な排便に関連して，いくつかの小さな糞便が非常に短期間に排出された場合は 1 回の排便とする．

(5) Nocturnal stools（any episode causing wakening）（夜間排便[起きる原因となる出来事]）
0 = No（なし）
10 = Yes（あり）

(6) Activity level（活動性レベル）
0 = No limitation of activity（活動制限なし）
5 = Occasional limitation of activity（不定期に活動制限を受ける）
10 = Severe restricted activity（極端に活動制限を受ける）
・“Occasional limitation of activity”とは学校に通う又はそれと同等のことが可能であるが，活動が制限される（例えば学校に通えるが，休憩時間に遊べないなど）
・“Severe restricted activity”とは学校に通う又はそれと同等の活動ができないこと

9.5.1.4 安全性の評価

9.5.1.4.1 安全性評価項目

(1) 有害事象及び副作用
・有害事象
・副作用

(2) 他覚所見
・理学的検査
血圧・脈拍数・体温
・一般臨床検査

9.5.1.4.2 他覚所見

(1) 一般臨床検査

治験責任（分担）医師は，治験薬投与開始日（0 週），投与 2，6，8，10，14，18，22，26，30 週に採血・採尿し，下記臨床検査項目を臨床検査受託機関にて測定した．CAI スコア不応例は 14 週で終了した．中止した被験者は中止時及び最終投与 8 週後にも測定した．

検査日が治験薬投与日の場合は治験薬投与に先立ち採血・採尿した．検体の回収は，臨床検査受託機関が行った．臨床検査受託機関より提供される検査伝票は実施医療機関及び治験依頼者が保存した．

1) 血液学的検査：

赤血球数，ヘモグロビン，ヘマトクリット値，白血球数，白血球分画（好中球，好酸球，好塩基球，単球，リンパ球），血小板数

2) 血液生化学的検査：

AST（GOT），ALT（GPT），ALP，LDH，γ-GTP，総蛋白，アルブミン，総コレステロール，総ビリルビン，BUN，血清クレアチニン，血清電解質（Na，K，Cl）

3) 尿検査（定性）:

蛋白，糖，潜血，ウロビリノーゲン

1 回あたりの採血量は約 4 mL（CRP 測定用も含む），1 回あたりの採尿量は約 5 mL とした．

(2) 免疫血清学的検査

治験責任（分担）医師は，治験薬投与開始日（0 週），投与 14，30 週に抗核抗体，抗 dsDNA 抗体（抗 dsDNA IgG 抗体，抗 dsDNA IgM 抗体）の測定のために採血を行った．CAI スコア不応例は 14 週で評価終了となるため，30 週は実施しないこととした．中止した被験者は中止時及び最終投与 8 週後にも測定した．

検査日が治験薬投与日の場合は治験薬投与に先立ち採血した．検体の回収及び測定は，臨床検査受託機関が行った．臨床検査受託機関より提供される検査伝票は実施医療機関及び治験依頼者が保存した．1 回あたりの採血量は約 3 mL とした．

(3) 理学的検査

治験責任（分担）医師は，各投与日において，血圧，脈拍数，体温を投与開始直前，投与中は 30 分ごと，投与終了から 2 時間までは 30 分ごとに測定した．投与終了時とその直前の測定との間隔が 15 分以上の場合は，投与終了時にも測定した．測定結果は，症例報告書に記録した．

例 1）投与時間が 2 時間 00 分の場合（↓は測定ポイント）

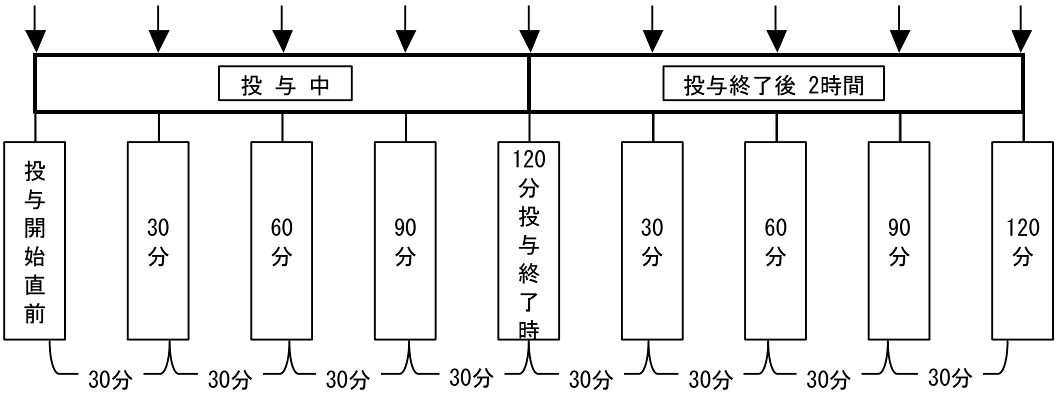

例 2）投与時間が 2 時間 15 分の場合（↓は測定ポイント）

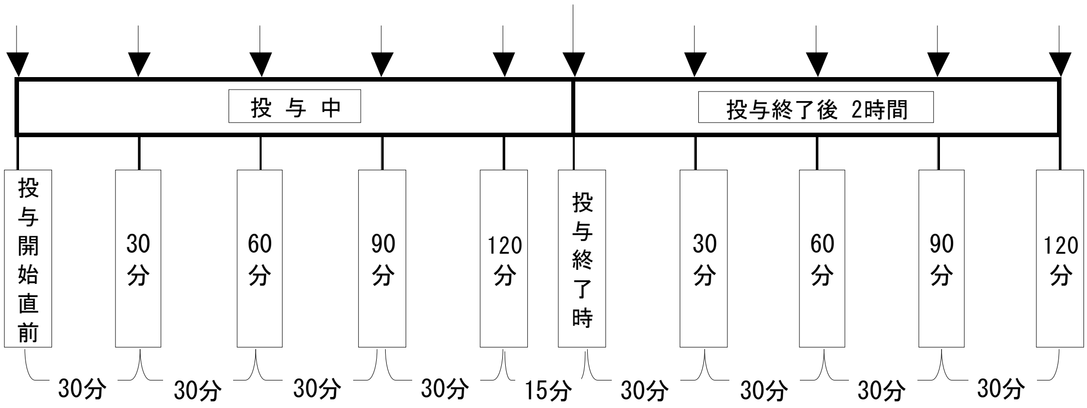

9.5.1.4.3　有害事象

有害事象とは，治験薬投与後，評価期間中に認められた臨床上好ましくない，又は意図しない徴候（臨床的に意義のある検査値の異常を含む），症状又は疾患のことであり，本剤との因果関係の有無は問わないこととした．有効性評価項目の悪化は，有害事象として取り扱わないこととするが，重篤な場合又は治験責任（分担）医師が有害事象として取り上げると判断した場合はその限りではない．

有害事象を認めた場合，治験責任（分担）医師は，適切に処置を行うこととした．また，治験薬との因果関係の有無に係わらず，原則として正常化又は有害事象として捉えないレベルに回復するまで追跡調査を実施することとした．器質的な障害で不可逆的な有害事象が認められた場合は，症状が安定又は固定するまで追跡調査を行うこととした．

(1) 症状又は疾患

治験責任（分担）医師は，治験薬投与時から安全性評価期間終了時までの被験者来院時に，問診及び診察などにより有害事象の有無を確認することとした．

また，結核を始めとした呼吸器感染症の有無を確認するために，治験責任（分担）医師は，同意取得後かつ登録前 4 週以内に胸部 X 線検査，胸部 CT 検査（適宜）を実施することとした．更に評価期間中の投与 14，30 週（CAI スコア応答例のみ）又は中止時に胸部 X 線検査，胸部 CT 検査（適宜）を実施し，異常所見の有無を確認することとした．異常所見を認めた場合は，その原因となる疾患又は症状を有害事象として取り扱うこととした．

(2) 他覚所見

1) 一般臨床検査，理学的検査（血圧，脈拍数，体温）

治験責任（分担）医師は，臨床的に意義のある異常※と判断した場合には有害事象として取り上げた．

※：「臨床的に意義のある異常」は以下を判断基準として判定した．

－臨床的徴候又は臨床症状に関連性のある場合

ただ，これらの症状，徴候が別途有害事象として報告されている場合は，当該検査値異常を有害事象とする必要はないこととした．

－当該検査値異常に対して内科的又は外科的治療を行った場合

－当該検査値異常のため治験薬の投与方法が変更（用量変更，休薬，中止など）された場合

－その他，治験責任（分担）医師が臨床的に意義のある異常と判断した場合

2) 免疫血清学的検査

抗核抗体，抗 dsDNA IgG 抗体，抗 dsDNA IgM 抗体は，以下に示す基準範囲を逸脱したものを「異常」として取り扱うこととした．また，以下の有害事象として取り扱う基準に該当した場合を有害事象として取り扱うこととした．

検査項目	基準範囲	有害事象として取扱う基準
抗核抗体	40倍未満	抗体力価3ランク以上の悪化
抗dsDNA IgG抗体	12 IU/mL以下	陰性から陽性，陽性から陽性（悪化方向）
抗dsDNA IgM抗体	6 U/mL未満	陰性から陽性，陽性から陽性（悪化方向）

3) 有害事象の評価及び基準

(a) 発現日

症状が認められた日又は臨床検査異常などの認められた検査日とした．なお，発現した日が明確でない場合は，報告を受けた日とした．

(b) 程度

有害事象の程度は，次の基準で分類した．

1. 軽　度：被験者の日常生活に影響がない程度
2. 中等度：被験者の日常生活にその事象が原因で多少の支障がある程度
3. 高　度：被験者の日常生活がその事象が原因でできない程度

(c) 重篤度

有害事象の重篤度は，次のように分類した.

1. 重篤でない：2 以外
2. 重篤：以下の a)～g)

a) 死亡
b) 死亡につながるおそれのあるもの
c) 治療のために病院又は診療所への入院，又は入院期間の延長が必要とされるもの
d) 障害
e) 障害につながるおそれのあるもの
f) a) から e) に掲げる被験者に準じて重篤であるもの
g) 後世代における先天性の疾病又は異常

(d) 治験薬との因果関係

治験薬との因果関係は，次のように分類した.

1. 明らかに関連あり

治験薬との時間的関連性があり，治験薬以外の要因が考えられない又は治験薬以外の要因の関与を考慮する必要がない場合.

2. おそらく関連あり

治験薬との時間的関連性があり，原疾患，合併症，併用薬，併用療法などの他の要因の関与が考えにくい場合.

3. 関連あるかもしれない

治験薬との時間的関連性が否定できず，原疾患，合併症，併用薬，併用療法などの他の要因で当該有害事象の可能性を説明できるが，治験薬との関連性を完全に否定できない場合.

4. 関連なし

治験薬との時間的関連性がない場合.

原疾患，合併症，併用薬，併用療法など，他の要因で合理的に説明できる場合.

なお，治験薬との因果関係が「関連なし」以外の有害事象を副作用とした.

(e) 転帰

有害事象の転帰は，次の基準で分類した.

1. 回復：正常化又は有害事象として捉えないレベルまでに回復したもの
2. 継続：その時点で回復に至っていないもの
3. 死亡：当該有害事象により死亡したもの
4. 不明：当該有害事象以外の原因で被験者死亡のため転帰が不明だったもの，又は被験者との連絡がつかずに転帰が確認できなかったもの

(f) 転帰日

転帰日は，それぞれ次の基準で分類した.

回復：回復した日．ただし，回復した日が特定できない場合は転帰を確認又は判断

した日とした.

継続：継続を確認又は判断した日

死亡：死亡日．ただし，死亡日が特定できない場合は死亡を確認又は判断した日とした.

不明：当該有害事象以外の原因で被験者死亡のため転帰が不明だった場合は死亡日．その他は確認又は判断した日とした.

(g) 追跡調査

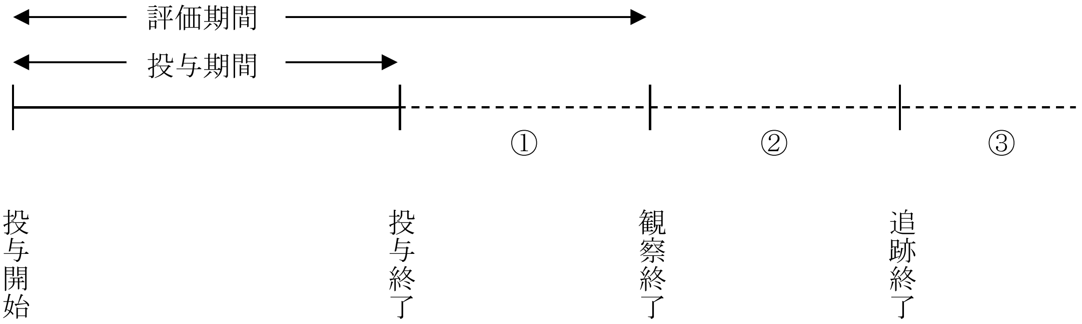

- 投与終了後①の期間は 56 日間とし，有害事象の有無を調査した.
- 観察終了後②の期間は 28 日間とし，評価期間中（投与期間+①）に発生した有害事象を追跡調査した.
- 観察終了後②の期間中に追跡調査した有害事象の経過を症例報告書に記録した.
- 有害事象が未回復の場合の症例報告書への転帰日は，観察終了後②の期間終了日以後の観察日とした.
- 観察終了後②の期間終了時に未回復の副作用については，その後の経過（③）を調査した.
- -評価期間終了後（①終了以降）の調査において，途中で調査を打ち切る妥当な理由がある場合には，その理由を症例報告書に記録して追跡調査を終了とした.

4) 症例報告書への記録事項

有害事象を認めた場合，治験責任（分担）医師は，その有害事象名※，程度，重篤度，感染症，発現日時（治験薬の投与開始から投与終了 2 時間以内に発生した有害事象については時刻も記入），転帰日，転帰，処置内容，治験薬との因果関係を症例報告書の有害事象欄に記録した．また，有害事象名に疾患名を記入する場合，その疾患に付随する症状又は臨床検査値の異常化，胸部 X 線検査／CT 検査の異常所見の内容は有害事象欄に記録しなかった．なお，免疫血清学的検査については別途安全性の評価を行うため有害事象欄には記録しなかった.

有害事象の「コメント欄」には, 転帰が回復以外で追跡調査不要と判断した場合，その理由，治験薬との因果関係の判定理由なども記録した．また，当該有害事象に

より治験中止に至った場合は，症例報告書の中止欄に中止に至った有害事象として記録した．

なお，これらの内容が診療記録などの原資料に記録されている場合は，治験協力者が症例報告書に転記することができることとした．

※：「有害事象名」は以下の基準に従った．
- 原則として診断名を用いる．
- 診断名が明確でない場合は症状名を用いる．
- 複数の症状が発現していて，それらが1つの診断名で示すことができる場合は，その診断名を用いる．
- 外科的処置などは有害事象とせず，外科的処置などを必要とする病気や症状が確認されている場合は，それを有害事象とする．

9.5.1.5 その他の臨床検査項目

(1) TNFα，IL-6，CRP

治験責任（分担）医師は，TNFα，IL-6 は投与開始日（0 週）のみ，CRP は投与開始日（0 週），投与 2，6，8，10，14，18，22，26，30 週，中止時に採血を行った．CAI スコア不応例は 8 週に測定して終了した．また，CAI スコア不応例が 8 週の評価後に中止した場合，中止時の評価は不要とした．なお，検査実施日が治験薬投与日の場合は，治験薬投与に先立ち採血した．

採取した TNFα 及び IL-6 測定用の血液検体は遠心分離し，血清又は血漿の一定量を採取した後，凍結保存した．CRP は一般臨床検査測定用の検体と同様に取り扱った．検体の回収及び測定は，臨床検査受託機関が行った．臨床検査受託機関より提供される検査伝票は，実施医療機関及び治験依頼者が保存した．

1 回あたりの採血量は TNFα 及び IL-6 測定用に約 2 mL，CRP 測定用は血液生化学的検査と合わせて約 4 mL とした．

9.5.2 測定項目の適切性

(1) 有効性評価項目の設定根拠

対象が小児であるため，すべての被験者において Mayo スコア評価に必要な S 状結腸鏡検査を実施することは難しいと考えられた．よって，国内成人を対象とした臨床試験で評価項目として使用した非侵襲的スコアである CAI スコアにて評価することとした．CAI スコアは非侵襲的な評価指標として高い頻度で使用され，臨床症状と臨床検査データがバランスよく配分された指標である．また，CAI スコアと Mayo スコアは高い相関（r=0.92）を示すことが報告されている [1)]．その他，国内外の臨床試験で用いられているパーシャル Mayo スコアについても検討することとした．なお，

S 状結腸鏡検査を実施した被験者については Mayo スコアの評価を実施することとした．更に，海外小児の臨床試験で使用した PUCAI スコアについても本治験で検討することとした．

　また，TA-650 の小児の潰瘍性大腸炎に対するステロイドの減量効果を評価するために各評価日のステロイド使用量を評価項目に設定した．

(2) 安全性評価項目の設定根拠

　これまでに実施された本剤の臨床試験と同様に，一般的な項目に加えループス様症状の発現に注意するため，抗核抗体，抗 dsDNA IgG 抗体及び抗 dsDNA IgM 抗体の測定を行うこととした．また，本剤は結核を含む感染症などの有害事象に注意する必要があるため，胸部 X 線検査及び必要に応じて胸部 CT 検査を実施することとした．

9.5.3 薬物濃度の測定

9.5.3.1.1 検査，観察項目及び時期

　治験責任（分担）医師又は治験協力者は，血清中インフリキシマブ濃度及び ATI 測定のために採血を行った．

　薬物濃度測定機関は，測定開始までに試験計画書（付録 16.1.13d）を別途作成して血清中インフリキシマブ濃度及び ATI の測定を行い，測定結果について，最終報告書（付録 16.1.13e）を作成した．臨床薬理解析責任者は，データ固定時までに薬物動態解析計画書（第 1 版）（作成日 : 2015 年 1 月 19 日）（付録 16.1.9c）を別途作成した．データ固定後，薬物動態を評価し，薬物動態解析報告書（付録 16.1.9d）を作成した．

(1) 採血時期

治験責任（分担）医師は，治験薬投与開始日（0 週），投与 2，6，8，10，14，18，22，26，30 週に採血を行った．CAI スコア不応例は 14 週で終了する．中止した被験者は中止時及び最終投与 8 週後にも採血を行った．

採血日が治験薬投与日の場合は，投与に先立ち採血を行ったが，治験薬投与開始日（0 週），14 週，22 週については，投与前及び投与終了 1 時間後の合計 2 回採血を行った．なお，CAI スコア不応例は 14 週の投与終了 1 時間後の採血は行わなかった．

(2) 採血量 : 1 回あたり約 2 mL

(3) 検体の処理

採取した血液（約 2 mL）を遠心分離し，薬物動態測定用検体として，血清の一定量を分取し，-20℃以下で凍結保存した．

凍結保存した薬物動態測定用検体は，臨床検査受託機関が実施医療機関より回収した．臨床検査受託機関は，別途定める「検体送付手順書」に従って薬物濃度測定機関に送付した．

(4) 症例報告書への記録事項

治験責任（分担）医師又は治験協力者は，採血日時を症例報告書に記録した．

【設定根拠】

海外で行われた ACT1, ACT2 試験及び国内で行われた潰瘍性大腸炎の臨床試験を参考に，TA-650 を 0，2，6 週に 5 mg/kg を投与し，14 週，22 週に投与した場合の薬物動態を検討するために必要な採血時期を設定した．

9.6 データの品質保証

治験依頼者は，本治験の品質及び信頼性維持のために，田辺三菱製薬株式会社 GCP 標準業務手順書に基づく「治験の品質管理」，及び田辺三菱製薬株式会社の GCP 監査標準業務手順書に基づく「治験の品質保証」を行った．また，実施医療機関及び治験責任医師は，治験依頼者による治験の品質管理及び品質保証に協力した．

治験の品質管理においては，モニターは，適宜，直接閲覧を行い，本治験が実施医療機関の治験に係わる業務に関する手順書, 最新の治験実施計画書及び GCP を遵守して実施されていることを確認した．また，治験責任（分担）医師から報告された症例報告書の記録内容が正確かつ完全であることを，原資料などの治験関連記録に照らして検証できることを確認した．モニタリングについては，詳細な手順を定めたモニタリング手順書（付録 16.1.13f）を作成し，それに基づいて実施した．

また，治験が治験実施計画書及び GCP を遵守して行われていることを保証するため，監査担当者は GCP 監査標準業務手順書に従って監査を行い, 品質管理が適切に行われていることを確認した．

9.7 治験実施計画書で計画された統計手法及び被験者数の決定

9.7.1 統計及び解析計画

詳細については統計解析計画書（第 3.0 版）（付録 16.1.9a），薬物動態解析計画書（第 1.0 版）（付録 16.1.9c）を参照．

9.7.1.1 解析対象集団

(1) 有効性に関する解析対象集団

有効性解析対象集団は，以下の被験者を除いた最大の解析対象集団（Full Analysis Set : 以下 FAS）とした．

(a) 対象疾患（潰瘍性大腸炎）以外の被験者．

(b) 評価期間に治験薬を 1 度も投与されなかった被験者．

(c) 評価期間に有効性データが全く得られなかった被験者．

(2) 安全性に関する解析対象集団

安全性解析対象集団は，以下の被験者を除いたものとした．

(a) 評価期間に治験薬を 1 度も投与されなかった被験者．

(b) 評価期間の治験薬投与開始後の安全性データが全く得られなかった被験者．

(3) 薬物動態に関する解析対象集団

薬物動態解析対象集団の定義は以下のとおりとするが，症例の取扱いの詳細については症例検討会の取り決めに従った．

治験薬を少なくとも 1 回投与された被験者のうち，投与後に 1 度でも血清中インフリキシマブ濃度又は ATI のデータが得られた被験者の集団を薬物動態の解析対象集団とした．

9.7.1.2 データの取扱い

データの取扱いは以下のとおりとした．本項に規定していない事項でデータの取扱いを検討する必要がある場合には，治験依頼者の GCP 標準業務手順書に定められた手順に従って，データの取扱い規定及びデータの取扱いを決定した．なお，詳細は統計解析計画書（付録 16.1.9a）に記載した．

9.7.1.2.1 欠測値

(1) 検査の欠測又は検査検体の問題などにより測定不能となった場合，当該項目を欠測値として取扱った．
(2) 治験薬の投与量が各投与日の規定用量の 75%未満の投与となった場合には，それ以降の次の治験薬投与までの有効性評価は欠測値として取扱うこととした．
(3) 併用禁止薬剤・療法の違反があった場合には，当該症例のすべてのデータを欠測値扱いとはせず，症例検討会の取決めに従った．

9.7.1.2.2 有効性評価データの脱落又は欠測値の取扱い

9.7.1.2.2.1 評価時期の逸脱の許容範囲

測定時点ごとの集計には，許容範囲に合致するデータを採用し，許容範囲外データによるデータ補完は行わなかった．許容範囲は 2，6，8 週（±6 日)，10 週(±7 日)，14 週以降（-13～+14 日）とした．

有効性評価（その他臨床検査の評価項目を除く）については，治験薬投与週は該当週の治験薬投与後の評価は採用しなかった．許容範囲内で複数データがある場合は，以下のとおりに従った．

・基準日により近い評価を採用する．

・基準日からのずれが前後で等しい評価が存在する場合には，前の評価を採用する．

・同一測定日の評価が複数存在する場合には，規定時期ラベルの評価を採用する．

9.7.1.2.2.2　治験薬最終投与週ごとの集計における時点データの取扱い

治験薬の最終投与週別に時点の許容範囲を以下のとおりとした.

治験薬最終投与	許容範囲週
0 週	0，2
2 週	0，2，6
6 週	0，2，6，8，10，14
14 週	0，2，6，8，10，14，18，22
22 週	0，2，6，8，10，14，18，22，26，30

9.7.1.2.2.3　中止時の有効性評価データの取扱い

中止時の有効性評価は，治験薬の最終投与別に規定した.
計画された次の投与までの評価が時点採用候補とした.

治験薬最終投与	中止評価時点採用候補の許容範囲週
0 週後	2
2 週後	6
6 週後	10，14
14 週後	18，22
22 週後	26，30

9.7.1.2.2.4　最終時点データの取扱い

(1)　CAI スコア不応例
完了例：8 週時点の許容範囲内にある，より後ろの評価を採用した.
(2)　CAI スコア応答例
完了例：30 週時点の許容範囲内にある，より後ろの評価を採用した.
(3)　中止例（CAI スコア不応例及び CAI スコア応答例）
中止例：最終時点データの許容範囲は以下のとおりとした.

治験薬最終投与	許容範囲日
0週後	初回投与から20日以内にある，より後ろの評価を採用する.
2週後	初回投与から48日以内にある，より後ろの評価を採用する.
6週後	初回投与から112日以内にある，より後ろの評価を採用する.
14週後	初回投与から168日以内にある，より後ろの評価を採用する.
22週後	初回投与から224日以内にある，より後ろの評価を採用する.

9.7.1.2.2.5　Treatment failure

以下に示す条件に該当した被験者（「Treatment failure」と定義する）には，実際の有効性評価項目のスコア，ステロイド使用量にかかわらず，該当した時期以降の評価において，CAI スコア，パーシャル Mayo スコア，Mayo スコア，PUCAI スコアについては登録時のスコアを使用した．また，同様に各スコア（CAI，Mayo，PUCAI）寛解率，Mayo スコア改善率，粘膜治癒率，PUCAI スコア変化量 20 ポイント以上減少した被験者の割合率の補完（TF）は，非寛解，非改善，非治癒，20 ポイント以上減少非該当としてそれぞれ取扱った．ステロイド使用量については実際の使用量の代わりに 0 週の使用量を使用し，ステロイド離脱率については非離脱として取扱った．

Treatment failure

(1) 腸管切除術又はストーマ造設術を施行した被験者
(2) 原疾患悪化又は効果不十などにより治験を中止した被験者
(3) 原疾患悪化などにより，下記薬剤の新規開始又は増量を行った被験者
　・ステロイド（経口剤，注射剤，注腸剤，坐剤）
　・アザチオプリン，6-メルカプトプリン
　・サラゾスルファピリジン製剤（経口剤，坐剤）
　・5-アミノサリチル酸製剤（経口剤，注腸剤）

この Treatment failue の補完を実施する場合には，存在する直前の評価時点の値で補完する方法（Last Observation Carried Forward 補完法，以下 LOCF）による欠測値の補完を先に適用させた後で該当した時期以降の評価結果に対する補完（TF）を行った．

なお，Treatment failure に該当するか否かについては症例検討会の検討結果に従った．

9.7.1.2.2.6　各スコアのデータの取扱い

(1) CAI スコア

各時点の CAI サブスコアに欠測がある場合には, 存在する直前の評価時点の値（CAI サブスコア）で補完（LOCF）した値を当該時点のサブスコアとし，これらを用いて当該時点の CAI スコアを算出した．

中止例，CAI スコア不応例について，それぞれ中止時より後，8 週より後の未評価の時点の CAI サブスコアについても同様に補完して，CAI スコアを算出した．

(2) パーシャル Mayo スコア

各時点のパーシャル Mayo スコアの 3 つのサブスコア（排便回数，直腸からの出血，医師による全般評価）に欠測がある場合には， LOCF 法による値を当該時点のサブスコアとし，これらを用いて当該時点のパーシャル Mayo スコアを算出することとした．

中止例，CAI スコア不応例について，それぞれ中止時より後，8 週より後の未評価の時点の 3 つのサブスコアについても同様に補完して，パーシャル Mayo スコアを算出した．

(3) Mayo スコア

観察期間に内視鏡所見サブスコアを評価した被験者を対象に Mayo スコアを算出した．30 週の内視鏡所見サブスコアに欠測がある場合には，中止時評価を補完（LOCF）した値を 30 週のサブスコアとし，これらを用いて 30 週の Mayo スコアを算出した．

また，30 週，若しくは中止時の評価が実施されていない場合，補完するデータがないため，Mayo スコアの算出は行わなかった．

(4) PUCAI スコア

各時点の PUCAI サブスコアに欠測がある場合には，存在する直前の評価時点の値（PUCAI サブスコア）で補完（LOCF）した値を当該時点のサブスコアとし，これらを用いて当該時点の PUCAI スコアを算出することとした．中止例，CAI スコア不応例について，それぞれ中止時より後，8 週より後の未評価の時点の PUCAI サブスコアについても同様に補完して，PUCAI スコアを算出した．

(5) 各スコア（CAI，Mayo，PUCAI）の寛解

各スコア（CAI，Mayo，PUCAI）の寛解については，当該時点のすべてのサブスコアが欠測の場合を除き，上記(1)，(3)，(4)に従い算出した各スコア（CAI，Mayo，PUCAI）に基づき，寛解又は非寛解を判定した．

すべてのサブスコアが欠測の場合は，非寛解を補完した．

また，中止例，CAI スコア不応例について，それぞれ中止時より後，8 週より後の未評価の時点の各スコア（CAI，Mayo，PUCAI）寛解率については，非寛解を補完した．

(6) Mayo スコア改善，粘膜治癒

Mayo スコア改善については，当該時点のすべてのサブスコアが欠測の場合を除き，上記(3)に従い算出した Mayo スコアに基づき，改善又は非改善を判定した．粘膜治癒については，上記(3)に従い算出した内視鏡所見サブスコアに基づき，治癒又は非治癒を判定した．また，30 週，若しくは中止時に内視鏡所見サブスコアの評価が実施されていない場合，補完するデータがないため，判定は行わなかった．

中止例については，中止時より後の未評価の時点の Mayo スコア改善に対し非改善を，粘膜治癒に対し非治癒を補完した．

(7) PUCAI スコア変化量 20 ポイント以上減少

PUCAI スコア変化量 20 ポイント以上減少については，当該時点のすべてのサブスコアが欠測の場合を除き，上記(4)に従い算出した PUCAI スコアに基づき，その変化量 20 ポイント以上減少の該当又は非該当を判定した．

すべてのサブスコアが欠測の場合は，その変化量 20 ポイント以上減少の非該当を補完した．中止例，CAI スコア不応例について，それぞれ中止時より後，8 週より後の未評価の時点の PUCAI スコア変化量 20 ポイント以上減少に対し非該当を補完した．

9.7.1.2.2.7 ステロイド使用量のデータの取扱い

(1) ステロイド使用量

中止例の中止時より後，CAI スコア不応例の 8 週より後のステロイド使用量については，存在する直前の評価時点の値（ステロイド使用量）で補完（LOCF）した．

(2) ステロイド離脱

各時点のステロイド離脱については，上記(1)に従い算出したステロイド使用量に基づき，離脱又は非離脱を判定した．

中止例，CAI スコア不応例について，それぞれ中止時より後，8 週より後の未評価の時点のステロイド離脱に対し非離脱を補完した．

9.7.1.2.3 安全性評価データの取扱い

(1) 評価時期の逸脱の許容範囲

測定時点ごとの集計には，許容範囲に合致するデータを採用し，許容範囲外データによるデータ補完は行わなかった．許容範囲は 2，6，8 週（±6 日），10 週(±7 日)，14 週以降（-13～+14 日）とした．

安全性評価（その他臨床検査の評価項目（CRP）を含める）については，治験薬投与週は投与開始日（0 週）を除き，該当週の治験薬投与後の評価でも採用した．許容範囲内で複数データがある場合は，以下のとおりに従った．

・基準日により近い評価を採用する．

・基準日からのずれが前後で等しい評価が存在する場合には，後の評価を採用する．

・同一測定日の評価が複数存在する場合には，規定時期ラベルの評価を採用する．

治験薬の最終投与週別に時点の許容範囲を以下のとおりとした．

・臨床検査値及びその他臨床検査項目（CRP）の場合

治験薬最終投与	許容範囲週
0 週後	0，2
2 週後	0，2，6
6 週後	0，2，6，8，10，14
14 週後	0，2，6，8，10，14，18，22
22 週後	0，2，6，8，10，14，18，22，26，30

・免疫血清学的検査の場合

治験薬最終投与	許容範囲週
0，2 週後	0
6，14 週後	0，14
22 週後	0，14，30

9.7.1.2.4 薬物動態データの取扱い

(1) 脱落又は欠測値の取扱い

検査値の欠測又は検査検体の問題などにより測定不能となった場合，当該測定結果を欠値として取扱った．その他の取扱いについては症例検討会（承認日：2014 年 12 月 15 日）及び PK/PD/PGx Data Handling Assessment（承認日：2014 年 12 月 24 日）の取決めに従った．

なお，PK/PD/PGx Data Handling Assessment にて，市販レミケード投与後の ATI 判定結果について取扱いを決定した．市販レミケードの投与は ATI の評価に影響を与

えることが明確であるため，当該被験者（被験者識別コード：TA-650UC-003-01, TA-650UC-022-02）については市販レミケード投与後の ATI 時点判定（最終投与 8 週後）を不採用とし，欠値として取扱うこととした．当該被験者の ATI 判定は，欠値とした ATI 時点判定以外のデータを使用し，[「TA-650 の小児の潰瘍性大腸炎を対象とした臨床試験」における血清中インフリキシマブ濃度及び抗インフリキシマブ抗体（ATI）の測定] 試験計画書（付録 16.1.13d）1)の ATI 判定フローチャート（図 13.6.7—2—1 及び図 13.6.7—2—2）に従って決定した．

(2) 定量下限未満の取扱い

血清中インフリキシマブ濃度の測定結果が定量下限未満（定量下限：0.1 μg/mL）であった場合，該当測定結果を計算過程では 0 μg/mL として取扱い，測定結果の表記は「BLQ」とした．

9.7.2 有効性の評価

有効性解析対象集団を解析対象として，以下の解析を実施した．

9.7.2.1 CAI スコア

(1) 評価項目
- CAI スコア，CAI スコア寛解

(2) 解析方法
- 欠測値に対する補完をしない場合（各評価時点に「最終」の時点を含む）と補完（TF）を適用した場合の両方の結果を示した（各サブスコアの要約統計量はこの限りではない）．
- 各評価時点における，CAI スコア及び CAI スコア変化量の要約統計量と CAI スコア寛解率を算出した．

9.7.2.2 パーシャル Mayo スコア

(1) 評価項目
- パーシャル Mayo スコア

(2) 解析方法
- 欠測値に対する補完をしない場合（各評価時点に「最終」の時点を含む）と補完（TF）を適用した場合の両方の結果を示した（各サブスコアの要約統計量はこの限りではない）．
- 各評価時点における，パーシャル Mayo スコア及びパーシャル Mayo スコア変化量の要約統計量を算出した．

9.7.2.3 Mayo スコア

(1) 評価項目
- Mayo スコア，Mayo スコア改善，Mayo スコア寛解，粘膜治癒

(2) 解析方法

・欠測値に対する補完をしない場合（各評価時点に「最終」の時点を含む）と補完（TF）を適用した場合の両方の結果を示した（各サブスコアの要約統計量はこの限りではない）.

・各評価時点における，Mayo スコア及び Mayo スコア変化量の要約統計量と Mayo スコア改善率，Mayo スコア寛解率，粘膜治癒率を算出した.

9.7.2.4 PUCAI スコア

(1) 評価項目

・PUCAI スコア，PUCAI スコア寛解，PUCAI スコア変化量 20 ポイント以上減少

(2) 解析方法

・欠測値に対する補完をしない場合（各評価時点に「最終」の時点を含む）と補完（TF）を適用した場合の両方の結果を示した（各サブスコアの要約統計量はこの限りではない）.

・各評価時点における，PUCAI スコア及び PUCAI スコア変化量の要約統計量と PUCAI スコア寛解率，PUCAI スコア変化量 20 ポイント以上減少した被験者の割合を算出した.

9.7.2.5 ステロイド使用量

(1) 評価項目

・ステロイド使用量，ステロイド離脱

(2) 解析方法

・FAS のうち登録時にステロイド（経口剤）を使用していた被験者を対象とした.

・欠測値に対する補完をしない場合（各評価時点に「最終」の時点を含む）と補完（TF）を適用した場合の両方の結果を示した.

・各評価時点における，ステロイド使用量及びステロイド使用量の変化率の要約統計量とステロイド離脱率を算出した.

9.7.3 安全性の評価

安全性解析対象集団を解析対象とし，以下の解析を実施した．有害事象名の MedDRA/J による読替えについては，MedDRA/J のバージョン 17.1 を用いた.

9.7.3.1 安全性データの要約

(1) 評価項目

有害事象，有害事象（SOC=臨床検査以外），副作用，副作用（SOC=臨床検査以外），重篤な有害事象，重篤な副作用，中止に至った有害事象，程度別有害事象，時期別有害事象，中止に至った副作用，感染症，感染症（副作用），重篤な感染症，重篤な感染症（副作用），投与中止に至った感染症，Infusion reaction，Infusion reaction（副作用），重篤な Infusion reaction，投与中止に至った Infusion reaction，有害事象（免疫血清学的検査），一般臨床検査，理学的検査

(2) 解析方法
解析項目ごとに，発現被験者数，発現率（%）を示した．

9.7.3.2 有害事象の内訳

(1) 評価項目
有害事象，副作用，重篤な有害事象，重篤な副作用，中止に至った有害事象，感染症，感染症（副作用），重篤な感染症，重篤な感染症（副作用），Infusion reaction，Infusion reaction（副作用）
(2) 解析方法
各項目について，発現被験者数，発現率（%），発現件数を，全体及び MedDRA における器官大分類（SOC）別，SOC 別・基本語（PT）別に示した．SOC については国際合意番号昇順とし，PT については発現被験者数降順・PT コード昇順で表示した．

9.7.3.3 有害事象（免疫血清学的検査）

有害事象（免疫血清学的検査）について，発現被験者数，発現率（%），発現件数を，「抗核抗体↑」，「二本鎖 DNA 抗体(IgG)↑」「二本鎖 DNA 抗体(IgM)↑」別に示した．

9.7.3.4 Infusion reaction

(1) 評価項目
Infusion reaction，Infusion reaction（副作用）
(2) 解析方法
1) 投与回数別の Infusion reaction 発現率
投与回数別の発現被験者数，発現率（%）を示した．
2) 投与 1 回あたりの Infusion reaction 発現率
全投与回数（全被験者の治験薬投与回数の合計），発現回数（Infusion reaction が認められた投与回数），投与 1 回あたりの Infusion reaction 発現率（全投与回数に対する発現回数の割合の百分率）を示した．
3) ATI 別の Infusion reaction 発現率
ATI 別（陰性，陽性，評価不能）の発現被験者数，発現率（%）を示した．

9.7.3.5 程度別有害事象の内訳

(1) 評価項目
有害事象，副作用，感染症，感染症（副作用）
(2) 解析方法
各評価項目（免疫血清学的検査以外）について，発現被験者数，発現率（%）を程度別（軽度，中等度，高度）で，全体及び MedDRA における SOC 別，SOC 別・PT 別に示した．

9.7.3.6 時期別有害事象

(1) 評価項目
有害事象，副作用，感染症，感染症（副作用）

(2) 解析方法
各評価項目（臨床検査以外）について，発現被験者数，発現率（%）を次の時期の分類区分ごとに示した．
時期の分類区分：投与開始日（0 週）～14 週投与前まで，14 週投与以降～22 週投与前まで，22 週投与以降～30 週評価日
治験薬投与日に発現した有害事象は，Infusion reaction 等の明らかに投与後に発現した有害事象を除き治験薬投与前に発現したものとして集計した．

9.7.3.7 免疫血清学的検査

(1) 評価項目
「抗核抗体↑」，「二本鎖 DNA 抗体(IgG)↑」，「二本鎖 DNA 抗体(IgM)↑」
(2) 解析方法
各項目について，各判定（陰性，陽性）について，0 週（投与開始日）と 14，30 週及び投与後の全時点のクロス表を示した．

9.7.3.8 一般臨床検査

(1) 評価項目
血液学的検査，血液生化学的検査，尿検査（定性）
(2) 解析方法
臨床検査値の異常増加，異常減少別の異常変動の発現被験者数及び発現率を示した．また，血液学的検査，血液生化学的検査については，各評価時点における要約統計量（n，Median，Q1，Q3，Min，Max）を算出した．なお，要約統計量は，被験者の年齢及び性別別に算出せず，全被験者の測定値から算出した．
尿検査（定性）については，各評価時点における判定区分ごとに度数分布を示した．
尿検査（定性，尿ウロビリノーゲン以外）については，各評価時点（8，14，22，30 週のみ）の各判定「正」「異」の，尿検査（尿ウロビリノーゲン）については，各評価時点（8，14，22，30 週のみ）の各判定「低」「正」「高」の度数分布を示した．
なお，小児は年齢及び性別により臨床検査値の基準範囲が異なることから，臨床検査値の臨床検査値の基準範囲は「新しい小児の臨床検査基準値ポケットガイド」[2)] を参考に性別年齢別の基準値（同意取得時年齢を用いる）を採用した．この資料に基準範囲が記載されていない白血球分画は異常変動を判定しなかった．尿検査（定性）は，成人の基準範囲を小児に代用しても臨床的に問題ないと判断したため，臨床検査受託機関から提供された基準値（成人用）を用いた．

9.7.3.9 理学的検査

(1) 評価項目
収縮期血圧，拡張期血圧，脈拍数，体温
(2) 解析方法
各項目について，各評価時点における投与時別・測定時間別の要約統計量（n，Mean，SD，Min，Max）を示した．

9.7.4　その他の評価

有効性解析対象集団を解析対象として，以下の解析を実施した．

(1) その他の臨床検査項目（CRP）
(2) 身長及び体重

9.7.5　薬物動態の評価

薬物動態の解析対象集団を対象とし，本剤の薬物動態を検討するために以下の解析を実施した．より詳細な解析方法は薬物動態等解析計画書に記載した．薬物動態等解析計画書は症例検討会後に薬物動態等関連データの取扱いが決定された後，速やかに固定した．

(1) 評価項目
・血清中インフリキシマブ濃度（血清中 TA-650 濃度）
・ATI
(3) 評価時期
0 週投与前，0 週投与終了 1 時間後，2 週投与前，6 週投与前，8 週，10 週，14 週投与前，14 週投与終了 1 時間後，18 週，22 週投与前，22 週投与終了 1 時間後，26 週，30 週に評価した．
(4) 解析方法
各評価時点の血清中インフリキシマブ濃度の要約統計量を示した．
ATI の判定（陰性，陽性，評価不能）について度数分布，割合を示すとともに各評価時点の血清中インフリキシマブ濃度の要約統計量を ATI 判定別に示した．

9.7.6　被験者数の決定

治験薬投与例数として 20 名

【設定根拠】

潰瘍性大腸炎は特定疾患に指定されており，計画立案当時の 2011 年度の特定疾患医療受給者証交付件数は約 11 万人である．このうち，本治験の対象となる「小児」で「中等症～重症」の患者は約 1200 人程度と推定される．その上，既存治療で寛解維持が可能である患者，成長障害や低栄養状態，小児特有の QOL 悪化による手術適応例が含まれることから，実際に治験適応となる患者は，更に少ないと考えられ，集積可能例数に限界がある．よって，実施可能性の観点から目標症例数を 20 例とした．なお，医薬品医療機器総合機構との本治験についての対面助言において，限られた症例数の検討であり，有効性及び安全性について一定の達成基準を設定することは困難であるため，有効性，安全性及び薬物動態を総合的に検討すること，また，対象年齢が 6～17 歳であり児童期（6～11 歳）と青少年期（12～17 歳）にまたがるため，特定の年齢層に偏ることがないよう配慮することが望ましい旨，助言を受けていた．よって，それぞれの年齢層で一定例数を集積することとした．

20 例で実施することにより，有効性及び安全性について，以下のとおり，評価が可能と考えた．なお，薬物動態の評価は得られた例数で実施した．

◆有効性

成人の潰瘍性大腸炎患者を対象とした国内臨床試験において，8 週の CAI スコア寛解率（FAS のうち登録時の CAI スコアが 7 以上かつ血便スコア 2 以上）は，38.5%（30/78）であった．症例数 20 例で，これらの結果を再現したとすると 95%CI は次のとおりとなる．

・CAI スコア寛解率（38.5%）の 95%CI；20.7%～59.9%.

◆安全性

本剤の副作用で注意すべきものの一つとして感染症が考えられた．成人の潰瘍性大腸炎患者を対象とした国内臨床試験の感染症（副作用）の発現率は 18.3%（19/104）である．成人と小児で発現率が同じとすると 98.2%の確率で 1 例検出することが可能であり，本剤の感染症のリスクを評価できる例数である．

9.8 治験の実施又は計画された解析に関する変更

9.8.1 治験実施計画書の改訂

本治験開始後に治験実施計画書を 2 回改訂した． 1 回目の改訂の主な変更内容は，有害事象の追跡調査の調査期間，悪性腫瘍発現時の対応及び妊娠報告手順の明確化であり，併せて誤記訂正も行った．2 回目の改訂の主な変更内容は結核検査方法の追加であった．治験実施計画書（第 02.00.00000 版，作成年月日：2013 年 5 月 22 日）を付録 16.1.1a，その変更箇所一覧を付録 16.1.1b に添付した．

9.8.2 解析計画の変更

統計解析計画書及び薬物動態解析計画書は，データベース固定前の 2014 年 12 月 15 日（第 3.0 版），2015 年 1 月 19 日（第 1.0 版）に固定し，その内容は「9.7.1 統計及び解析計画」に記載した．

10. 治験対象患者

10.1 患者の内訳

被験者の内訳を図 10.1－1に，試験の中止理由の内訳を表 10.1－1に示した．

本治験では被験者の代諾者から文書同意を取得した．代諾者の同意が得られた被験者数は30名であり，そのうち21名が登録され治験薬の投薬が開始された．1名は投与8週のCAIスコア評価前に中止したため，投与8週のCAIスコア評価例は20名であった．投与8週のCAIスコア評価例のうちCAIスコア応答例が18名で，そのうち14名が治験期間を完了し，4名は治験期間中に中止した．中止理由の内訳は有害事象の発現のためが1名，効果不十分が1名，原疾患悪化のためが2名であった．CAIスコア不応例は2名で，2名とも投与14週の評価を完了した．

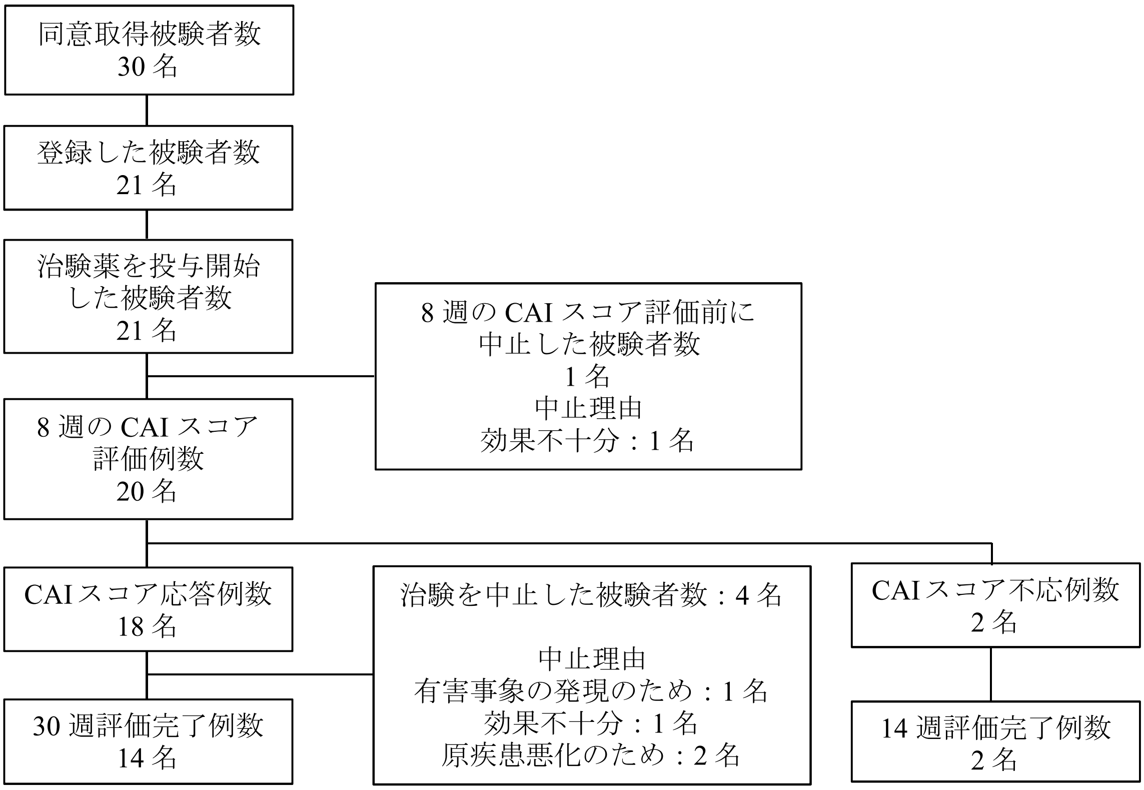

図 10.1－1　被験者の内訳

表 10.1－1　中止理由の内訳

				n
治験薬が投与された被験者			有害事象の発現のため	1
			効果不十分	2
			原疾患悪化のため	2
			その他	0
	8週CAIスコア評価例		有害事象の発現のため	1
			効果不十分	1
			原疾患悪化のため	2
			その他	0
		CAIスコア応答例	有害事象の発現のため	1
			効果不十分	1
			原疾患悪化のため	2
			その他	0
		CAIスコア不応例	有害事象の発現のため	0
			効果不十分	0
			原疾患悪化のため	0
			その他	0
	8週CAIスコア評価前中止例		有害事象の発現のため	0
			効果不十分	1
			原疾患悪化のため	0
			その他	0

10.2 治験実施計画書からの逸脱

治験実施計画書からの逸脱は次の 5 つに分類した.

A：組入れ基準を満たしていないにもかかわらず，治験に組入れられた被験者
B：治験期間中に中止基準に該当するようになったが，中止されなかった被験者
C：治療方法や用量が不適切であった被験者
D：禁止されている併用薬，併用療法を受けた被験者
E：その他

逸脱を認めたすべての被験者の一覧を付録 16.2.2 に添付した. 治験実施計画書からの逸脱は 27 件であった. なお，治験実施計画書からの重要な逸脱は認められなかった.

11. 有効性の評価

11.1 解析したデータセット

治験薬が投与された被験者 21 名について，2014 年 12 月 15 日に実施した症例検討会にてデータの取扱いを決定した．症例検討会の議事録は付録 16.1.13g に添付した．対象被験者は治験薬が投与されたすべての被験者とした．

有効性解析対象集団は，対象疾患以外の被験者，評価期間に治験薬を 1 度も投与されなかった被験者及び評価期間に有効性データが得られなかった被験者を除いた FAS とし，治験薬が投与された全 21 名が採用となった．また，薬物動態解析対象集団は，治験薬が少なくとも 1 回投与された被験者のうち，投与後に 1 度でも血清中インフリキシマブ濃度又は ATI のデータが得られた 21 名とした．

11.2 人口統計学的及び他の基準値の特性

有効性解析対象集団の被験者背景（FAS）を表 11.2－1 に示した．

被験者の性別は，男児 52.4%（11/21 名），女児 47.6%（10/21 名）と男女ほぼ均等であった．また，同意取得時の年齢（Median）は，14.0 歳であり，12～17 歳が 81.0%（17/21 名）と多く，身長及び体重（Median）は，それぞれ 158.0 cm，45.40 kg であった．BMI（Median）は 17.78 kg/m^2 であり，18.5 kg/m^2 未満の割合が 61.9%（13/21 名）と多かった．罹病期間（Median）は，2.10 年であり，3 年未満の被験者がほぼ 8 割を占めた．病変の拡がりによる病型は，全大腸炎型が 95.2%（20/21 名）でほとんどを占め，臨床的重症度及び臨床経過では，中等症及び再燃寛解型が共に 76.2%（16/21 名）で最も多かった．過去に潰瘍性大腸炎治療のために使用した経験のある薬剤・療法として，シクロスポリン不応例，タクロリムス不応例，血球成分除去療法不応例について調査したが，血球成分除去療法不応例が 9.5%（2/21 名）であり，シクロスポリン不応例及びタクロリムス不応例は認められなかった．また，潰瘍性大腸炎に対する腸切除又は処置，手術歴（腸切除を除く）を有している被験者はなかった．

既存治療抵抗性については，ステロイド不応例（過去又は登録時）が 81.0%（17/21 名）であり，そのうち，ステロイド不応例（離脱困難）が最も多く 76.2%（16/21 名）であった．ステロイド不応例（重症）とステロイド不耐例（過去）に該当する被験者はなかった．また，6-メルカプトプリン又はアザチオプリン不応例（過去又は登録時）は 47.6%（10/21 名）であった．6-メルカプトプリン又はアザチオプリン不耐例（過去）は 4.8%（1/21 名）であり，該当する被験者はほとんどなかった．

潰瘍性大腸炎の治療目的の併用薬は，アミノサリチル酸類（経口剤）が 90.5%（19/21 名）と最も多く，ステロイド（経口剤）及び免疫調節薬（アザチオプリン又は 6-メルカプトプリン）は，それぞれ 57.1%（12/21 名），42.9%（9/21 名）であった．

表 11.2－1　被験者背景（FAS）

被験者数			(N=21)
性別	n(%)	男	11(52.4)
		女	10(47.6)
年齢（歳）	n(%)	6～12未満	4(19.0)
		12～17以下	17(81.0)
	n		21
	Mean±SD		13.7±2.1
	Median		14.0
	[Q1 ,Q3]		[12.0,15.0]
	Min～Max		10～17
身長（cm）	n(%)	150未満	5(23.8)
		150～160未満	8(38.1)
		160以上	8(38.1)
	n		21
	Mean±SD		156.5±10.1
	Median		158.0
	[Q1 ,Q3]		[150.0,162.0]
	Min～Max		135～170
体重（kg）	n(%)	40未満	5(23.8)
		40～50未満	9(42.9)
		50以上	7(33.3)
	n		21
	Mean±SD		44.79±10.73
	Median		45.40
	[Q1 ,Q3]		[40.00,54.50]
	Min～Max		25.6～62.6
BMI（kg/m^2）	n(%)	18.5未満	13(61.9)
		18.5～25.0未満	8(38.1)
		25.0以上	0
	n		21
	Mean±SD		18.04±2.86
	Median		17.78
	[Q1 ,Q3]		[15.71,19.55]
	Min～Max		14.0～24.2
アレルギー歴	n(%)	無	8(38.1)
		有	13(61.9)
合併症（潰瘍性大腸炎の合併症を除く）	n(%)	無	6(28.6)
		有	15(71.4)
罹病期間（年）	n(%)	1未満	4(19.0)
		1～3未満	13(61.9)
		3～5未満	2(9.5)
		5以上	2(9.5)
	n		21
	Mean±SD		2.35±1.64
	Median		2.10
	[Q1 ,Q3]		[1.10,2.80]
	Min～Max		0.6～6.4
病変の拡がりによる病型	n(%)	左側大腸炎	1(4.8)
		全大腸炎	20(95.2)
臨床的重症度	n(%)	軽症	3(14.3)
		中等症	16(76.2)
		重症	2(9.5)
臨床経過	n(%)	再燃寛解型	16(76.2)
		慢性持続型	4(19.0)
		急性劇症型（急性電撃型）	0
		初回発作型	1(4.8)
過去に潰瘍性大腸炎治療のために使用した経験のある薬剤･療法	n(%)	シクロスポリン不応例	0
		タクロリムス不応例	0
		血球成分除去療法不応例	2(9.5)

表 11.2－1　被験者背景（FAS）（続き）

被験者数			(N=21)
潰瘍性大腸炎の合併症（腸管外合併症）	n(%)	無<br>有	14(66.7)<br>7(33.3)
潰瘍性大腸炎に対する腸切除	n(%)	無<br>有	21(100.0)<br>0
潰瘍性大腸炎に対する処置，<br>手術歴（腸切除を除く）	n(%)	無<br>有	21(100.0)<br>0
6-メルカプトプリン又はアザチオプリン<br>不応例（登録時）*1	n(%)	該当せず<br>該当	12(57.1)<br>9(42.9)
6-メルカプトプリン又はアザチオプリン<br>不応例（過去）*2	n(%)	該当せず<br>該当	16(76.2)<br>5(23.8)
6-メルカプトプリン又はアザチオプリン<br>不応例（過去又は登録時）*3	n(%)	該当せず<br>該当	11(52.4)<br>10(47.6)
6-メルカプトプリン又は<br>アザチオプリン不耐例（過去）*4	n(%)	該当せず<br>該当	20(95.2)<br>1(4.8)
ステロイド不応例（登録時）*5	n(%)	該当せず<br>該当	18(85.7)<br>3(14.3)
ステロイド不応例（離脱困難）<br>（過去又は登録時）*6	n(%)	該当せず<br>該当	5(23.8)<br>16(76.2)
ステロイド不応例（重症）<br>（過去又は登録時）*7	n(%)	該当せず<br>該当	21(100.0)<br>0
ステロイド不応例（過去又は登録時）*8	n(%)	該当せず<br>該当	4(19.0)<br>17(81.0)
ステロイド不耐例（過去）*9	n(%)	該当せず<br>該当	21(100.0)<br>0
併用薬（ステロイド<br>（経口剤））	n(%)	無<br>有	9(42.9)<br>12(57.1)
併用薬（アミノサリチル酸類<br>（経口剤））	n(%)	無<br>有	2(9.5)<br>19(90.5)
併用薬（5-アミノサリチル酸製剤<br>（経口剤））	n(%)	無<br>有	4(19.0)<br>17(81.0)
併用薬（サラゾスルファピリジン製剤<br>（経口剤））	n(%)	無<br>有	18(85.7)<br>3(14.3)
併用薬（アザチオプリン）	n(%)	無<br>有	13(61.9)<br>8(38.1)
併用薬（6-メルカプトプリン）	n(%)	無<br>有	20(95.2)<br>1(4.8)
併用薬（免疫調節薬（アザチオプリン<br>又は6-メルカプトプリン））	n(%)	無<br>有	12(57.1)<br>9(42.9)
IL-6（pg/mL）	n(%)	2未満<br>2～5未満<br>5以上	9(42.9)<br>4(19.0)<br>8(38.1)
	n<br>Mean±SD<br>Median<br>[Q1 ,Q3]<br>Min～Max		21<br>9.6063±21.5610<br>2.5200<br>[1.2600,7.9600]<br>0.000～99.900
TNFα（pg/mL）	n(%)	0.55未満<br>0.55～1.0未満<br>1.0～1.5未満<br>1.5以上	9(42.9)<br>1(4.8)<br>5(23.8)<br>6(28.6)
	n<br>Mean±SD<br>Median<br>[Q1 ,Q3]<br>Min～Max		21<br>0.998±1.080<br>1.020<br>[0.000,1.640]<br>0.00～3.41

*1：観察期間開始日の12週以上前から6-メルカプトプリン又はアザチオプリンを使用し，4週以上前から一定用量使用し効果不十分．
*2：観察期間開始日前5年以内に6-メルカプトプリン又はアザチオプリンを12週以上使用し効果不十分．
*3：6-メルカプトプリン又はアザチオプリン不応例（過去）又は（登録時）のいずれかに該当する被験者．
*4：観察期間開始日前5年以内に6-メルカプトプリン又はアザチオプリンを用い，治療継続が困難となる副作用を発現．
*5：観察期間開始日の2週以上前からプレドニゾロン換算で1 mg/kg/日以上又は20 mg/日以上で一定用量使用し効果不十分．
*6：観察期間開始日前18ヶ月以内にステロイドの減量に伴って原疾患の増悪又は再燃が起こり離脱困難．
*7：観察期間開始日前18ヶ月以内にステロイドを使用し効果不十分（プレドニゾロン換算1 mg/kg/日以上又は40 mg/日以上を経口で少なくとも2週間，静注で少なくとも1週間投与して治療効果が得られない）．
*8：ステロイド不応例（登録時），離脱困難（過去又は登録時），重症（過去又は登録時）のいずれかに該当する被験者．
*9：観察期間開始日前18ヶ月以内にステロイドを使用し，治療継続が困難となる副作用を発現．

評価項目の基準値（FAS）を表 11.2－2 に示した．

登録時の CAI スコア（Mean±SD）は，9.7±2.7 であり，スコア 7～8，9～10，11 以上の被験者は，それぞれ 42.9%（9/21 名），28.6%（6/21 名），28.6%（6/21 名）であった．パーシャル Mayo スコア（Mean±SD）は，5.6±1.6 であり，スコア 4～6 が 57.1%（12/21 名）と最も多かった．また, S 状結腸鏡検査を実施できた 8 名のみで評価した Mayo スコア（Mean±SD）では 7.0±2.4 であり，Mayo スコアにおける中等症（スコア 6～10）及び重症（スコア 11～12）がそれぞれ 50.0%（4/8 名），12.5%（1/8 名）であった．PUCAI スコア（Mean±SD）では 47.1±15.2 であり，スコア 31～60 が 66.7%（14/21 名）と最も多かった．

登録時にステロイドを使用していた被験者は 57.1%（12/21 名）であり，ステロイドの使用量については, すべての被験者がプレドニゾロン換算で 1 mg/kg/day 以下での使用であった．また，CRP（Median）は 0.20 mg/dL であった．

表 11.2－2　評価項目の基準値（FAS）

被験者数			(N=21)
CAIスコア	n(%)	7～8	9(42.9)
		9～10	6(28.6)
		11以上	6(28.6)
	n		21
	Mean±SD		9.7±2.7
	Median		9.0
	[Q1 ,Q3]		[8.0,11.0]
	Min～Max		7～16
CAI 1) 1週間の排便回数	n(%)	0	3(14.3)
		1	8(38.1)
		2	6(28.6)
		3	4(19.0)
CAI 2) 血便（1週間平均で）	n(%)	0	0
		2	5(23.8)
		4	16(76.2)
CAI 3) 医師の症状アセスメント	n(%)	0	0
		1	3(14.3)
		2	16(76.2)
		3	2(9.5)
CAI 4) 腹痛	n(%)	0	1(4.8)
		1	9(42.9)
		2	10(47.6)
		3	1(4.8)
CAI 5) 潰瘍性大腸炎による体温上昇	n(%)	0	21(100.0)
		3	0
CAI 6)腸管外合併症	n(%)	0	18(85.7)
		3	3(14.3)
		6	0
		9	0
CAI 7) 臨床検査	n(%)	0	13(61.9)
		1	5(23.8)
		2	1(4.8)
		4	2(9.5)

表 11.2－2　評価項目の基準値（FAS）（続き）

被験者数				(N=21)
パーシャルMayoスコア		n(%)	0～3	3(14.3)
			4～6	12(57.1)
			7～9	6(28.6)
		n		21
		Mean±SD		5.6±1.6
		Median		6.0
		[Q1 ,Q3]		[5.0,7.0]
		Min～Max		2～8
Mayoスコア（8週CAIスコア不応例を除く）		n(%)	0～5	3(37.5)
			6～8	3(37.5)
			9～10	1(12.5)
			11～12	1(12.5)
		n		8
		Mean±SD		7.0±2.4
		Median		7.0
		[Q1 ,Q3]		[5.0,8.5]
		Min～Max		4～11
	Mayo 1) 排便回数	n(%)	0	2(9.5)
			1	6(28.6)
			2	4(19.0)
			3	9(42.9)
	Mayo 2) 直腸からの出血	n(%)	0	2(9.5)
			1	3(14.3)
			2	16(76.2)
			3	0
	Mayo 3) 医師による全般評価	n(%)	0	0
			1	3(14.3)
			2	16(76.2)
			3	2(9.5)
	Mayo 4) 内視鏡所見（8週CAIスコア不応例を除く）	n(%)	0	0
			1	1(12.5)
			2	6(75.0)
			3	1(12.5)

表 11.2－2　評価項目の基準値（FAS）（続き）

被験者数				(N=21)
PUCAIスコア		n(%)	0～30	3(14.3)
			31～60	14(66.7)
			61～85	4(19.0)
		n		21
		Mean±SD		47.1±15.2
		Median		45.0
		[Q1 ,Q3]		[40.0,60.0]
		Min～Max		15～70
	PUCAI 1) 腹痛	n(%)	0	1(4.8)
			5	18(85.7)
			10	2(9.5)
	PUCAI 2) 直腸からの出血	n(%)	0	2(9.5)
			10	3(14.3)
			20	14(66.7)
			30	2(9.5)
	PUCAI 3) 便の硬さ	n(%)	0	0
			5	14(66.7)
			10	7(33.3)
	PUCAI 4) 24時間あたりの排便回数	n(%)	0	1(4.8)
			5	11(52.4)
			10	4(19.0)
			15	5(23.8)
	PUCAI 5) 夜間排便［起きる原因となる出来事］	n(%)	0	11(52.4)
			10	10(47.6)
	PUCAI 6) 活動性レベル	n(%)	0	3(14.3)
			5	16(76.2)
			10	2(9.5)
ステロイド使用量（mg/kg/day）（プレドニゾロン換算）		n(%)	無	9(42.9)
			1以下	12(57.1)
			1超	0
		n		12
		Mean±SD		0.25±0.23
		Median		0.20
		[Q1 ,Q3]		[0.10,0.32]
		Min～Max		0.0～0.9
CRP（mg/dL）		n(%)	0.5以下	14(66.7)
			0.5超	7(33.3)
		n		21
		Mean±SD		1.75±3.68
		Median		0.20
		[Q1 ,Q3]		[0.00,0.60]
		Min～Max		0.0～15.1

11.3 治療の遵守状況の測定

被験者ごとの投与状況を付録 16.2.8f に添付した．

被験者識別コード：TA-650UC-022-01 において，投与 6 週の治験薬の投与量を算出する際に体重換算を誤り，220 mg（規定投与量の 97.78%）と少ない投与量で投与されたが，規定投与量の 75%以上は投与されていることから，有効性及び薬物動態の評価上問題となるものではないと考えた．

被験者識別コード：TA-650UC-025-01 において，治験薬投与開始日（0 週）の治験薬投与が治験実施計画書で規定した投薬時間（2 時間以上）より短い 1 時間 52 分で投与されたが，規定投与量の全量が投与されていることから，有効性の評価上問題となるものではないと考えた．また，投与日当日に有害事象の発現はなく，安全性を評価する上でも問題ないと考えた．

被験者識別コード：TA-650UC-013-01 において，投与 6 週の治験薬投与日が治験実施計画書で規定した許容範囲（投与 6 週：±3 日）を超えていた．規定日の 6 日後に投与されており，規定日からのずれは大きくないことから，有効性の評価上問題となるものではないと考えた．

11.4 有効性に関する成績及び個別患者データ一覧表

11.4.1 有効性の解析

各有効性評価項目については，主な集計として評価時点として採用されたデータに基づく集計結果を提示することとした．また，中止した被験者や CAI スコア不応例に該当した被験者など投与 30 週評価が欠測した場合，存在する直前の評価時点の値で LOCF した集計結果を最終として表示した．

なお，投与 30 週までの各評価時点のデータが欠測した場合，データを補完した集計結果も提示することとした．欠測した各評価時点のデータの補完方法は，存在する直前の評価時点の値で LOCF した後に，TF に該当した時期以降の評価時点の値は，TF の規定に従うデータで補完することとした（9.7.1.2.2.5 Treatment failure 参照）．TF に該当した被験者は 5 名であり，すべての被験者が「原疾患悪化又は効果不十分等により治験中止した被験者」に該当し，そのうち 1 名が「原疾患悪化等により，ステロイド（経口剤，注射剤，注腸剤，坐剤）の新規開始又は増量を行った被験者」にも該当していた．TF に該当した被験者の詳細は，症例検討会議事録に記載した（詳細は付録 16.1.13g 参照）．

11.4.1.1 症状改善効果

11.4.1.1.1 CAI スコア

CAI スコアは，1 週間の排便回数，血便（1 週間平均で），医師の症状アセスメント，腹痛，潰瘍性大腸炎による体温上昇，腸管外合併症，臨床検査（ヘモグロビン又は ESR）の 7 項目におけるそれぞれのスコアを合計（0～29）して算出した，疾患活動性を評価する活動性指標であり，スコアが大きいほど，疾患活動性が高いことを示している．また，各評価日の CAI スコアから登録時の CAI スコアを差し引いた値を CAI スコア変化量とした．

CAI スコア及び CAI スコア変化量の各評価時点の要約統計量を表 11.4－1，CAI スコアの推移を図 11.4－1 に示した．なお，TF 補完した場合の各データをそれぞれ表 11.4－2，図 11.4－2 に示した．

登録時の CAI スコア（Mean±SD）は 9.7±2.7 であり，投与 2，6，8 週ではそれぞれ 4.0 ±3.2，3.5±3.5，3.2±3.5 と経時的に減少した．その後，投与 10～30 週では 2.5±2.3～3.5±2.2 で推移した．また，最終は 5.6±3.8 であった．CAI スコア変化量（Mean±SD）は，投与 2，6，8 週で-5.5±2.7，-6.0±4.0，-6.2±3.7 と経時的に大きくなり，投与 10～30 週では-5.9 ±3.8～-6.9±3.8 の間で推移した．また，最終では-4.1±4.2 であった．CAI スコア及び CAI スコア変化量共に，投与 2 週時点から症状改善効果が認められ，その効果が投与 30 週まで持続した．

なお，TF 補完を行った場合では，投与 2，6，8 週の CAI スコア（Mean±SD）は，それぞれ 4.5±3.9，4.0±4.3，3.8±4.3 と減少し，投与 10～30 週では 4.1±4.6～5.8±4.1 の間で推移した．また，投与 2，6，8 週の CAI スコア変化量（Mean±SD）は，それぞれ-5.2±2.9，-5.7 ±4.2，-5.9±3.8 と大きくなり，投与 10～30 週では-3.9±4.3～-5.5±4.4 の間で推移した．CAI スコア及び CAI スコア変化量共に，TF 補完前のデータと同様の傾向を示した．

表 11.4－1　CAI スコア及び CAI スコア変化量の要約統計量（FAS）

	CAIスコア						CAIスコア変化量								
	n	Mean	SD	Median	Q1	Q3	Mean	SD	Median	Q1	Q3	Min	Max	95%CI下限	95%CI上限
登録時	21	9.7	2.7	9.0	8.0	11.0	-	-	-	-	-	-	-	-	-
2週	20	4.0	3.2	4.0	1.0	5.5	-5.5	2.7	-6.0	-7.0	-4.0	-10	0	-6.7	-4.2
6週	20	3.5	3.5	3.0	0.5	4.0	-6.0	4.0	-5.5	-8.0	-3.5	-13	3	-7.8	-4.1
8週	20	3.2	3.5	3.0	0.5	3.5	-6.2	3.7	-6.0	-8.0	-5.0	-13	2	-7.9	-4.5
10週	18	2.7	2.9	3.0	0.0	3.0	-6.5	3.9	-6.0	-10.0	-4.0	-13	1	-8.4	-4.6
14週	16	2.7	3.0	2.0	0.0	3.5	-6.6	4.3	-6.5	-9.5	-5.0	-13	2	-8.9	-4.4
18週	16	2.6	2.6	2.5	0.0	3.0	-6.8	4.0	-6.0	-10.0	-4.5	-13	1	-8.9	-4.6
22週	14	3.3	3.1	3.0	0.0	5.0	-6.1	4.6	-5.5	-10.0	-3.0	-13	2	-8.8	-3.5
26週	14	2.5	2.3	2.5	1.0	4.0	-6.9	3.8	-6.5	-10.0	-5.0	-14	1	-9.1	-4.7
30週	14	3.5	2.2	3.0	2.0	5.0	-5.9	3.8	-5.5	-8.0	-3.0	-14	0	-8.1	-3.8
最終	21	5.6	3.8	5.0	3.0	7.0	-4.1	4.2	-3.0	-6.0	-1.0	-14	2	-6.0	-2.2

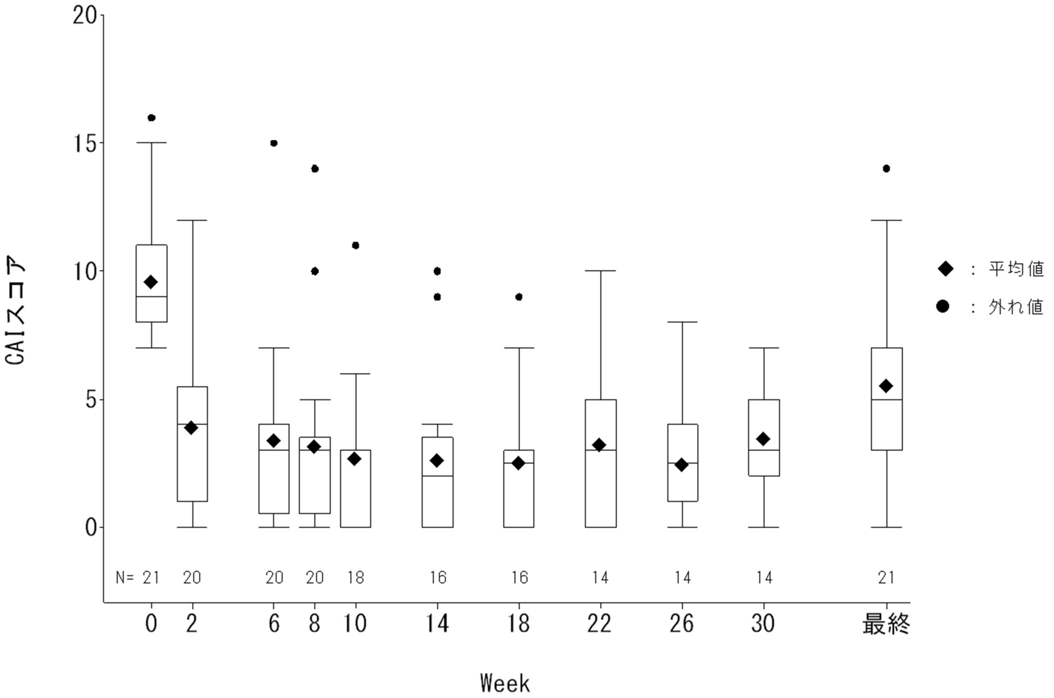

※外れ値は四分位範囲の1.5倍を超える観察結果．箱の下辺，中線，上辺は，
それぞれQ1, Median, Q3，ひげの端は外れ値を除いた最小値及び最大値．

図 11.4ー1　CAI スコアの推移（FAS）

表 11.4－2　CAI スコア及び CAI スコア変化量（TF）の要約統計量（FAS）

	CAIスコア（TF）						CAIスコア変化量（TF）								
	n	Mean	SD	Median	Q1	Q3	Mean	SD	Median	Q1	Q3	Min	Max	95%CI下限	95%CI上限
登録時	21	9.7	2.7	9.0	8.0	11.0	-	-	-	-	-	-	-	-	-
2週	21	4.5	3.9	4.0	1.0	6.0	-5.2	2.9	-6.0	-7.0	-4.0	-10	0	-6.5	-3.9
6週	21	4.0	4.3	3.0	1.0	4.0	-5.7	4.2	-5.0	-8.0	-3.0	-13	3	-7.6	-3.8
8週	21	3.8	4.3	3.0	1.0	4.0	-5.9	3.8	-6.0	-8.0	-5.0	-13	2	-7.6	-4.2
10週	21	4.1	4.6	3.0	0.0	6.0	-5.5	4.4	-6.0	-8.0	-2.0	-13	2	-7.5	-3.5
14週	21	4.7	4.8	3.0	1.0	9.0	-5.0	4.8	-6.0	-8.0	0.0	-13	2	-7.1	-2.8
18週	21	4.6	4.6	3.0	2.0	7.0	-5.1	4.6	-5.0	-8.0	0.0	-13	2	-7.2	-3.0
22週	21	5.7	4.6	5.0	3.0	9.0	-4.0	4.9	-3.0	-7.0	0.0	-13	2	-6.2	-1.8
26週	21	5.1	4.6	4.0	1.0	8.0	-4.5	4.7	-5.0	-8.0	0.0	-14	2	-6.7	-2.4
30週	21	5.8	4.1	5.0	3.0	8.0	-3.9	4.3	-3.0	-6.0	0.0	-14	2	-5.8	-1.9

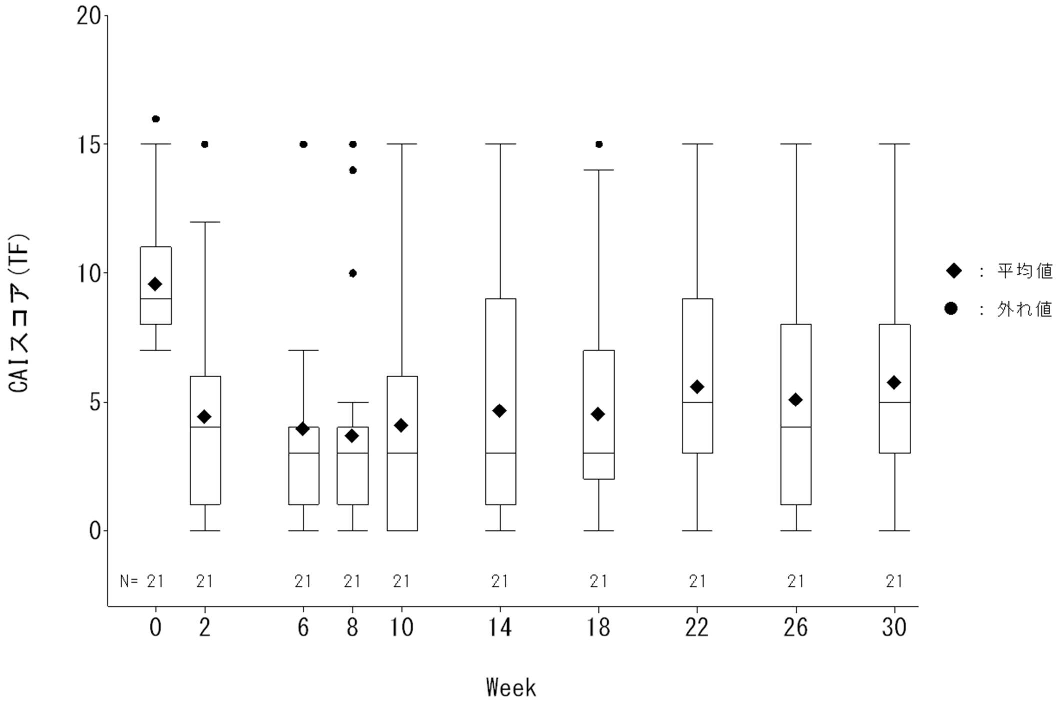

※外れ値は四分位範囲の1.5倍を超える観察結果．箱の下辺，中線，上辺は，それぞれQ1，Median，Q3，ひげの端は外れ値を除いた最小値及び最大値．

図 11.4－2　CAI スコア（TF）の推移（FAS）

CAI スコア寛解は，登録時を除いた各評価日の CAI スコアが 4 以下となった場合と定義した．各評価時点の CAI スコア寛解率を表 11.4－3 に示した．なお，TF 補完した場合の CAI スコア寛解率を表 11.4－4 に示した．

CAI スコア寛解率は，投与 2 週で 60.0%（12/20 名）となり，投与 6，8 週で共に 80.0%（16/20 名）と増加した．その後，投与 10～30 週までの間では 64.3～87.5%で推移した．また，最終では 42.9%（9/21 名）であった．CAI スコア寛解率において，本剤を投与することにより，投与 2 週から 60%以上の被験者において寛解の状態に導く効果が認められ，その効果が投与 30 週まで持続することが確認された．

なお，TF 補完を行った場合では，CAI スコア寛解率は，投与 2 週で 57.1%（12/21 名），投与 6，8 週で共に 76.2%（16/21 名）となり，投与 10～30 週までの間では 42.9～66.7%で推移した．投与 2 週から寛解の状態に導く効果が認められ，その効果が持続するという傾向は TF 補完前と同様であることが確認された．

表 11.4－3　CAI スコア寛解率（%）（FAS）

	CAIスコア		
	n	寛解	%
2週	20	12	60.0
6週	20	16	80.0
8週	20	16	80.0
10週	18	14	77.8
14週	16	14	87.5
18週	16	14	87.5
22週	14	9	64.3
26週	14	12	85.7
30週	14	9	64.3
最終	21	9	42.9

表 11.4－4　CAI スコア（TF）寛解率（%）（FAS）

	CAIスコア（TF）		
	n	寛解	%
2週	21	12	57.1
6週	21	16	76.2
8週	21	16	76.2
10週	21	14	66.7
14週	21	14	66.7
18週	21	14	66.7
22週	21	9	42.9
26週	21	12	57.1
30週	21	9	42.9

11.4.1.1.2 パーシャル Mayo スコア

パーシャル Mayo スコアは，排便回数，直腸からの出血，医師による全般評価の 3 項目におけるそれぞれのスコアを合計（0～9）して算出した，疾患活動性を評価する活動性指標である．スコアが大きいほど，疾患活動性が高いことを示している．また，各評価日のパーシャル Mayo スコアから登録時のパーシャル Mayo スコアを差し引いた値をパーシャル Mayo スコア変化量とした．

各評価時点におけるパーシャル Mayo スコア及びパーシャル Mayo スコア変化量の要約統計量を表 11.4－5 に示した．なお，TF 補完した場合のパーシャル Mayo スコア及びパーシャル Mayo スコア変化量の要約統計量を表 11.4－6 に示した．

登録時のパーシャル Mayo スコア（Mean±SD）は 5.6±1.6 であり，投与 2，6，8 週ではそれぞれ 2.6±2.3，2.2±2.0，1.7±1.7 と経時的に減少し，投与 10～30 週では 1.8±1.7～2.8±1.9 の間で推移した．また，最終では 3.7±2.2 であった．パーシャル Mayo スコア変化量（Mean±SD）は，投与 2，6，8 週ではそれぞれ-2.9±2.2，-3.3±2.2，-3.8±1.7 と経時的に大きくな

り，投与 10～30 週では-2.6±2.8～-3.6±2.5 の間で推移した．また，最終では-1.9±2.7 であった．このように，パーシャル Mayo スコア及びパーシャル Mayo スコア変化量共に，投与 2 週時点から症状改善効果が認められ，その効果が投与 30 週まで持続した.

なお，TF 補完を行った場合では，投与 2，6，8 週のパーシャル Mayo スコア（Mean±SD）は，それぞれ 2.9±2.6，2.5±2.3，2.0±2.1 と減少し，投与 10～30 週では 2.6±2.3～3.6±2.2 の間で推移した．また，投与 2，6，8 週のパーシャル Mayo スコア変化量（Mean±SD）は，それぞれ-2.7±2.2，-3.1±2.3，-3.6±1.9 と大きくなり，投与 10～30 週では-2.0±2.5～-3.0±2.5 の間で推移した．投与 2 週時点から症状改善効果を認め，その効果が投与 30 週まで持続する傾向は，TF 補完後のパーシャル Mayo スコア及びパーシャル Mayo スコア変化量においても同様であった.

表 11.4−5　パーシャル Mayo スコア及びパーシャル Mayo スコア変化量の要約統計量（FAS）

	パーシャルMayoスコア						パーシャルMayoスコア変化量								
	n	Mean	SD	Median	Q1	Q3	Mean	SD	Median	Q1	Q3	Min	Max	95%CI下限	95%CI上限
登録時	21	5.6	1.6	6.0	5.0	7.0	-	-	-	-	-	-	-	-	-
2週	20	2.6	2.3	2.5	0.5	4.5	-2.9	2.2	-3.0	-4.5	-0.5	-6	0	-3.9	-1.8
6週	20	2.2	2.0	2.5	0.0	3.0	-3.3	2.2	-3.5	-5.0	-1.0	-7	1	-4.3	-2.2
8週	20	1.7	1.7	1.5	0.0	3.0	-3.8	1.7	-3.0	-5.5	-3.0	-6	0	-4.6	-2.9
10週	18	2.2	2.1	2.5	0.0	3.0	-3.1	2.5	-3.0	-5.0	-2.0	-6	1	-4.3	-1.9
14週	16	1.9	1.9	2.0	0.0	3.0	-3.6	2.5	-4.5	-5.5	-1.5	-7	1	-4.9	-2.2
18週	16	2.1	1.8	2.5	0.0	3.0	-3.4	2.4	-3.5	-5.5	-2.0	-6	1	-4.7	-2.2
22週	14	2.4	2.1	3.0	0.0	4.0	-3.0	2.7	-3.5	-5.0	0.0	-7	1	-4.6	-1.4
26週	14	1.8	1.7	1.5	0.0	3.0	-3.6	2.4	-3.5	-5.0	-3.0	-7	3	-5.0	-2.3
30週	14	2.8	1.9	3.0	2.0	4.0	-2.6	2.8	-2.5	-5.0	-1.0	-7	4	-4.3	-1.0
最終	21	3.7	2.2	4.0	3.0	4.0	-1.9	2.7	-2.0	-3.0	0.0	-7	4	-3.1	-0.7

表 11.4−6　パーシャル Mayo スコア及びパーシャル Mayo スコア変化量（TF）の要約統計量（FAS）

	パーシャルMayoスコア（TF）						パーシャルMayoスコア変化量（TF）								
	n	Mean	SD	Median	Q1	Q3	Mean	SD	Median	Q1	Q3	Min	Max	95%CI下限	95%CI上限
登録時	21	5.6	1.6	6.0	5.0	7.0	-	-	-	-	-	-	-	-	-
2週	21	2.9	2.6	3.0	1.0	5.0	-2.7	2.2	-3.0	-4.0	0.0	-6	0	-3.7	-1.7
6週	21	2.5	2.3	3.0	0.0	3.0	-3.1	2.3	-3.0	-5.0	-1.0	-7	1	-4.1	-2.0
8週	21	2.0	2.1	2.0	0.0	3.0	-3.6	1.9	-3.0	-5.0	-3.0	-6	0	-4.4	-2.7
10週	21	2.6	2.2	3.0	1.0	4.0	-3.0	2.3	-3.0	-5.0	-2.0	-6	1	-4.0	-1.9
14週	21	2.6	2.3	3.0	0.0	4.0	-3.0	2.5	-3.0	-5.0	0.0	-7	1	-4.1	-1.8
18週	21	2.7	2.2	3.0	1.0	4.0	-2.9	2.3	-3.0	-5.0	0.0	-6	0	-4.0	-1.8
22週	21	3.3	2.4	4.0	1.0	5.0	-2.2	2.6	-2.0	-4.0	0.0	-7	1	-3.4	-1.1
26週	21	2.9	2.4	3.0	1.0	4.0	-2.7	2.5	-3.0	-4.0	0.0	-7	3	-3.8	-1.5
30週	21	3.6	2.2	3.0	3.0	4.0	-2.0	2.5	-2.0	-3.0	0.0	-7	4	-3.2	-0.8

11.4.1.1.3 Mayo スコア

Mayo スコアは, パーシャル Mayo スコアの 3 項目に内視鏡所見を加えた 4 項目のサブスコアを合計（0～12）した疾患活動性を評価する活動性指標である．本治験では被験者が小児であることから，被験者への負担を考慮し，Mayo スコアの算出のための S 状結腸鏡検査を必須とせずに可能な限り実施することとした．また，登録時及び，投与 30 週又は中止日のいずれか一時点のみしか実施できない場合には，投与前後での比較ができないことから，Mayo スコアを集計しないこととした．そのため，評価できた被験者は 8 名と少なかった．また，各評価日の Mayo スコアから登録時の Mayo スコアを差し引いた値を Mayo スコア変化量とした．

Mayo スコア及び Mayo スコア変化量の各評価時点の要約統計量を表 11.4－7 に示した．なお，TF 補完した Mayo スコア及び Mayo スコア変化量の要約統計量を表 11.4－8 に示した．

登録時の Mayo スコア（Mean±SD）は 7.0±2.4 であり，投与 30 週では 4.9±2.4 に減少した．また，最終では 5.0±2.3 であった．Mayo スコア変化量（Mean±SD）は，投与 30 週に-2.6±3.9 であった．また，最終では-2.0±3.9 であった．

なお，TF 補完を行った場合では，投与 30 週の Mayo スコア（Mean±SD）は，4.8±2.3 を示し，補完前のデータと同様の傾向であった．また，投与 30 週の Mayo スコア変化量（Mean±SD）は-2.3±3.7 であった．TF 補完後の Mayo スコア及び Mayo スコア変化量においても補完前と同様の傾向が確認された．

表 11.4−7　Mayo スコア及び Mayo スコア変化量の要約統計量（FAS）

	Mayoスコア						Mayoスコア変化量								
	n	Mean	SD	Median	Q1	Q3	Mean	SD	Median	Q1	Q3	Min	Max	95%CI下限	95%CI上限
登録時	8	7.0	2.4	7.0	5.0	8.5	-	-	-	-	-	-	-	-	-
30週	7	4.9	2.4	5.0	4.0	6.0	-2.6	3.9	-2.0	-7.0	-1.0	-7	4	-6.1	1.0
最終	8	5.0	2.3	5.0	4.0	6.0	-2.0	3.9	-1.5	-5.5	0.5	-7	4	-5.3	1.3

表 11.4−8　Mayo スコア及び Mayo スコア変化量（TF）の要約統計量（FAS）

	Mayoスコア（TF）						Mayoスコア変化量（TF）								
	n	Mean	SD	Median	Q1	Q3	Mean	SD	Median	Q1	Q3	Min	Max	95%CI下限	95%CI上限
登録時	8	7.0	2.4	7.0	5.0	8.5	-	-	-	-	-	-	-	-	-
30週	8	4.8	2.3	4.5	4.0	5.5	-2.3	3.7	-1.5	-5.5	-0.5	-7	4	-5.3	0.8

Mayo スコア改善は，各評価時点で「Mayo スコアが登録時と比較して 30%以上減少かつ 3 ポイント以上減少」，「直腸からの出血の項目で登録時と比較して 1 ポイント以上減少又は 1 以下」の 2 つを満たす場合と定義した．また，Mayo スコア寛解は，登録時を除いた各評価時点の Mayo スコアが 2 以下かつ Mayo スコアの 4 項目において，すべての項目で 1 以下となった場合と定義した．

Mayo スコア改善率及び寛解率をそれぞれ表 11.4－9，表 11.4－10 に示した．なお，TF 補完した場合の Mayo スコア改善率及び寛解率をそれぞれ表 11.4－11，表 11.4－12 に示した．

投与 30 週の Mayo スコアの改善率及び寛解率はそれぞれ 42.9%（3/7 名），14.3%（1/7 名）であった．また，最終ではそれぞれ 37.5%（3/8 名），12.5%（1/8 名）であった．

なお，TF 補完を行った場合では，投与 30 週の Mayo スコアの改善率及び寛解率はそれぞれ 37.5%（3/8 名），12.5%（1/8 名）であった．TF 補完後の Mayo スコアの改善率及び寛解率においても補完前と同様の値を呈した．

表 11.4－9　Mayo スコア改善率（%）（FAS）

	Mayoスコア		
	n	改善	%
30週	7	3	42.9
最終	8	3	37.5

表 11.4－10　Mayo スコア寛解率（%）（FAS）

	Mayoスコア		
	n	寛解	%
30週	7	1	14.3
最終	8	1	12.5

表 11.4－11　Mayo スコア（TF）改善率（%）（FAS）

	Mayoスコア（TF）		
	n	改善	%
30週	8	3	37.5

表 11.4－12　Mayo スコア（TF）寛解率（%）（FAS）

	Mayoスコア（TF）		
	n	寛解	%
30週	8	1	12.5

11.4.1.1.4 PUCAI スコア

PUCAI スコアは，腹痛，直腸からの出血，便の硬さ，24 時間あたりの排便回数，夜間排便，活動性レベルの 6 項目におけるそれぞれのスコアを合計（0～85）して算出した，小児の潰瘍性大腸炎の活動性指標である．PUCAI スコアもスコアが大きいほど，疾患活動性が高いことを示す．また，各評価日の PUCAI スコアから登録時の PUCAI スコアを差し引いた値を PUCAI スコア変化量とした．

PUCAI スコア及び PUCAI スコア変化量の各評価時点の要約統計量を表 11.4－13 に示した．なお，TF 補完した場合の PUCAI スコア及び PUCAI スコア変化量の各評価時点の要約統計量を表 11.4－14 に示した．

登録時の PUCAI スコア（Mean±SD）は，47.1±15.2 であり，投与 2，6，8 週ではそれぞれ 20.3±16.3，17.3±17.5，12.5±13.5 と経時的に減少した．投与 10～30 週では 12.2±12.0～19.3±18.8 の間で推移した．また，最終では 28.8±22.5 であった．PUCAI スコア変化量（Mean±SD）は，投与 2，6，8 週でそれぞれ-25.8±17.5，-28.8±21.1，-33.5±14.0 を示した.投与 10～30 週では-26.8±27.8～-34.1±17.7 の間で推移した．また，最終では-18.3±28.4 であった．PUCAI スコア及び PUCAI スコア変化量共に，投与 2 週時点から症状改善効果が認められ，その効果が投与 30 週まで持続した．

なお，TF 補完を行った場合において，投与 2，6，8 週の PUCAI スコア（Mean±SD）は，それぞれ 22.6±19.3，19.8±20.6，15.2±18.2 と減少し，投与 10～30 週では 19.3±20.5～27.6±21.9 で推移した．投与 2，6，8 週の PUCAI スコア変化量（Mean±SD）は，それぞれ-24.5±18.0，-27.4±21.5，-31.9±15.4 と大きくなり，投与 10～30 週では-19.5±25.8～-27.9±19.9 の間で推移した．投与 2 週時点から症状改善効果が認められ，その効果が投与 30 週まで持続する傾向は，補完後の PUCAI スコア及び PUCAI スコア変化量においても同様であった．

表 11.4－13　PUCAI スコア及び PUCAI スコア変化量の要約統計量（FAS）

	PUCAIスコア						PUCAIスコア変化量								
	n	Mean	SD	Median	Q1	Q3	Mean	SD	Median	Q1	Q3	Min	Max	95%CI下限	95%CI上限
登録時	21	47.1	15.2	45.0	40.0	60.0	-	-	-	-	-	-	-	-	-
2週	20	20.3	16.3	22.5	2.5	35.0	-25.8	17.5	-25.0	-40.0	-7.5	-55	0	-33.9	-17.6
6週	20	17.3	17.5	15.0	2.5	32.5	-28.8	21.1	-35.0	-45.0	-10.0	-70	10	-38.6	-18.9
8週	20	12.5	13.5	10.0	0.0	22.5	-33.5	14.0	-40.0	-42.5	-22.5	-55	0	-40.0	-27.0
10週	18	14.7	16.9	10.0	0.0	20.0	-30.6	19.1	-37.5	-45.0	-20.0	-55	10	-40.0	-21.1
14週	16	12.2	12.0	10.0	2.5	15.0	-34.1	17.7	-40.0	-42.5	-27.5	-60	5	-43.5	-24.6
18週	16	14.4	16.8	10.0	2.5	20.0	-31.9	21.0	-40.0	-42.5	-22.5	-60	25	-43.0	-20.7
22週	14	18.2	15.6	12.5	5.0	35.0	-27.9	24.2	-40.0	-45.0	-10.0	-60	20	-41.8	-13.9
26週	14	13.2	14.0	10.0	0.0	20.0	-32.9	20.5	-37.5	-45.0	-25.0	-60	25	-44.7	-21.0
30週	14	19.3	18.8	15.0	5.0	30.0	-26.8	27.8	-32.5	-40.0	-15.0	-65	50	-42.8	-10.7
最終	21	28.8	22.5	30.0	10.0	40.0	-18.3	28.4	-20.0	-35.0	0.0	-65	50	-31.3	-5.4

表 11.4－14　PUCAI スコア及び PUCAI スコア変化量（TF）の要約統計量（FAS）

	PUCAIスコア（TF）						PUCAIスコア変化量（TF）								
	n	Mean	SD	Median	Q1	Q3	Mean	SD	Median	Q1	Q3	Min	Max	95%CI下限	95%CI上限
登録時	21	47.1	15.2	45.0	40.0	60.0	-	-	-	-	-	-	-	-	-
2週	21	22.6	19.3	25.0	5.0	35.0	-24.5	18.0	-25.0	-40.0	-5.0	-55	0	-32.7	-16.4
6週	21	19.8	20.6	15.0	5.0	35.0	-27.4	21.5	-35.0	-45.0	-10.0	-70	10	-37.2	-17.6
8週	21	15.2	18.2	10.0	0.0	25.0	-31.9	15.4	-40.0	-40.0	-20.0	-55	0	-38.9	-24.9
10週	21	19.3	20.5	15.0	5.0	25.0	-27.9	19.9	-35.0	-45.0	-20.0	-55	10	-36.9	-18.8
14週	21	19.5	19.7	15.0	5.0	25.0	-27.6	20.6	-35.0	-40.0	-5.0	-60	5	-37.0	-18.2
18週	21	20.0	19.7	15.0	5.0	30.0	-27.1	20.0	-30.0	-40.0	-5.0	-60	0	-36.2	-18.1
22週	21	26.9	20.6	25.0	10.0	40.0	-20.2	23.5	-15.0	-40.0	0.0	-60	20	-30.9	-9.5
26週	21	23.6	21.4	20.0	5.0	40.0	-23.6	22.5	-30.0	-40.0	0.0	-60	25	-33.8	-13.3
30週	21	27.6	21.9	20.0	10.0	40.0	-19.5	25.8	-20.0	-35.0	0.0	-65	50	-31.3	-7.8

PUCAI スコア寛解は，登録時を除いた各評価時点の PUCAI スコアが 10 未満となった場合と定義した．PUCAI スコア寛解率を表 11.4－15 に示した．なお，TF 補完した場合の PUCAI スコア寛解率を表 11.4－16 に示した．

PUCAI スコア寛解率は，投与 2 週で 35.0%（7/20 名）となり，投与 6，8 週共に 40.0%（8/20 名）と増加した．その後，投与 10～30 週までの間では 28.6～42.9%で推移した．また，最終では 19.0%（4/21 名）となった．投与 2 週から寛解の状態へ導く効果を認め，その効果は持続することが確認された．

なお，TF 補完を行った場合において，PUCAI スコア寛解率は，投与 2 週で 33.3%（7/21 名），投与 6，8 週共に 38.1%（8/21 名）となり，投与 10～30 週までの間では 19.0～33.3%で推移した．投与 2 週から効果が認められ，その効果が持続する傾向は補完前と同様であった．

表 11.4－15　PUCAI スコア寛解率（%）（FAS）

	PUCAIスコア		
	n	寛解	%
2週	20	7	35.0
6週	20	8	40.0
8週	20	8	40.0
10週	18	7	38.9
14週	16	6	37.5
18週	16	6	37.5
22週	14	4	28.6
26週	14	6	42.9
30週	14	4	28.6
最終	21	4	19.0

表 11.4－16　PUCAI スコア（TF）寛解率（%）（FAS）

	PUCAIスコア（TF）		
	n	寛解	%
2週	21	7	33.3
6週	21	8	38.1
8週	21	8	38.1
10週	21	7	33.3
14週	21	6	28.6
18週	21	6	28.6
22週	21	4	19.0
26週	21	6	28.6
30週	21	4	19.0

PUCAI スコアが 20 ポイント以上減少した場合は臨床的な意義がある大きさであり [3)]，その PUCAI スコア変化量が 20 ポイント以上減少した被験者の割合を表 11.4－17 に示した．な

お，TF 補完した場合の PUCAI スコア変化量が 20 ポイント以上減少した被験者の割合を表 11.4－18 に示した．また，登録時の PUCAI スコアが 20 ポイント未満の被験者は本集計から除外した．

PUCAI スコア変化量が 20 ポイント以上減少した被験者の割合は, 投与 2 週で 68.4%（13/19 名）となり，投与 6，8 週ではそれぞれ 73.7%（14/19 名），89.5%（17/19 名）と増加した．その後，投与 10～30 週では 64.3～88.2%の間で推移した．また，最終では 60.0%（12/20 名）であった．投与 2 週から改善効果を認め，その効果はその後も持続することが確認された．

なお，TF 補完を行った場合での PUCAI スコア変化量 20 ポイント以上減少率は，投与 2 週で 65.0%（13/20 名），投与 6，8 週ではそれぞれ 70.0%（14/20 名），85.0%（17/20 名）となり，投与 10～30 週までの間では 45.0～75.0%で推移した．その推移は補完前と同様の傾向であった．

表 11.4－17　PUCAI スコア変化量 20 ポイント以上減少率（%）（FAS）

	n	20以上	%
2週	19	13	68.4
6週	19	14	73.7
8週	19	17	89.5
10週	17	15	88.2
14週	16	13	81.3
18週	16	13	81.3
22週	14	9	64.3
26週	14	12	85.7
30週	14	10	71.4
最終	20	12	60.0

表 11.4－18　PUCAI スコア（TF）変化量 20 ポイント以上減少率（%）（FAS）

	n	20以上	%
2週	20	13	65.0
6週	20	14	70.0
8週	20	17	85.0
10週	20	15	75.0
14週	20	13	65.0
18週	20	13	65.0
22週	20	9	45.0
26週	20	12	60.0
30週	20	10	50.0

11.4.1.2　粘膜治癒率

粘膜治癒は，登録時を除いた各評価日の Mayo スコアの内視鏡所見の項目が 1 以下となっ

た場合と定義した．また，登録時及び，投与 30 週又は中止日の 2 時点で S 状結腸鏡検査が実施できた被験者のうち，登録時の Mayo スコアの内視鏡所見の項目が 1 以下の被験者は本集計から除外した．

粘膜治癒率を表 11.4－19 に示した．なお，TF 補完した場合の粘膜治癒率を表 11.4－20 に示した．

投与 30 週及び最終の粘膜治癒率は，それぞれ 33.3%（2/6 名），28.6%（2/7 名）であった．

なお，TF 補完を行った場合では，投与 30 週の粘膜治癒率は，28.6%（2/7 名）であり，TF 補完前と同様の傾向であった．

表 11.4－19　粘膜治癒率（%）（FAS）

	n	治癒	%
30週	6	2	33.3
最終	7	2	28.6

表 11.4－20　粘膜治癒率（%）（TF）（FAS）

	n	治癒	%
30週	7	2	28.6

11.4.1.3 ステロイド使用量

登録時にステロイド（経口剤）を使用していた被験者 12 名について，ステロイド（経口剤，注射剤）の使用量を検討した．なお，ステロイド（経口剤）は，観察期間開始日の 2 週以上前から用量一定とし，新たな開始及び増量は禁止とした．治験薬投与開始日以降は，治験責任（分担）医師が潰瘍性大腸炎の臨床的改善を認めた場合，減量を可能とし，治験実施計画書で定めた規定に従って実施することとした．なお，各評価週のステロイド使用量は，プレドニゾロン換算後，該当週の CAI スコア評価日の 6 日前から前日までのステロイド使用量の平均値を該当週の体重測定値で除算した値とした．

各評価時点のステロイド（経口剤，注射剤）の使用量を表 11.4－21 に示した．なお，TF 補完した場合のステロイド使用量を表 11.4－22 に示した．

登録時のステロイド使用量（Median）は 0.20 mg/kg/day であり，投与 2，6，8 週ではそれぞれ 0.19，0.17，0.16 mg/kg/day と減少した．投与 10～30 週では 0.04～0.15 mg/kg/day の間で推移した．また，最終では 0.05 mg/kg/day であった．

なお，TF 補完を行った場合では，投与 2，6，8 週のステロイド使用量（Median）は，それぞれ 0.19, 0.17, 0.16 mg/kg/day であり，投与 10～30 週では 0.05～0.15 mg/kg/day で推移した．補完前のデータの推移と同様の傾向であった．

表 11.4－21　ステロイド使用量（mg/kg/day）の要約統計量（FAS のうち登録時にステロイド（経口剤）を使用していた被験者）

	ステロイド使用量（mg/kg/day）									
	n	Mean	SD	Median	Q1	Q3	Min	Max	95%CI下限	95%CI上限
登録時	12	0.25	0.23	0.20	0.10	0.32	0.0	0.9	0.11	0.40
2週	12	0.24	0.21	0.19	0.10	0.32	0.0	0.8	0.10	0.37
6週	12	0.17	0.14	0.17	0.04	0.24	0.0	0.5	0.08	0.26
8週	12	0.15	0.12	0.16	0.04	0.20	0.0	0.4	0.07	0.23
10週	10	0.14	0.12	0.15	0.00	0.19	0.0	0.4	0.05	0.22
14週	9	0.11	0.12	0.09	0.00	0.17	0.0	0.4	0.02	0.20
18週	9	0.09	0.10	0.05	0.00	0.16	0.0	0.3	0.02	0.16
22週	8	0.08	0.09	0.04	0.00	0.17	0.0	0.2	0.01	0.15
26週	8	0.09	0.09	0.07	0.00	0.18	0.0	0.2	0.01	0.16
30週	8	0.18	0.29	0.10	0.02	0.16	0.0	0.9	-0.06	0.42
最終	12	0.13	0.24	0.05	0.00	0.16	0.0	0.9	-0.02	0.29

表 11.4－22　ステロイド使用量（mg/kg/day）（TF）の要約統計量（FAS のうち登録時にステロイド（経口剤）を使用していた被験者）

	ステロイド使用量（mg/kg/day）（TF）									
	n	Mean	SD	Median	Q1	Q3	Min	Max	95%CI下限	95%CI上限
登録時	12	0.25	0.23	0.20	0.10	0.32	0.0	0.9	0.11	0.40
2週	12	0.24	0.21	0.19	0.10	0.32	0.0	0.8	0.10	0.37
6週	12	0.17	0.14	0.17	0.04	0.24	0.0	0.5	0.08	0.26
8週	12	0.15	0.12	0.16	0.04	0.20	0.0	0.4	0.07	0.23
10週	12	0.14	0.11	0.15	0.03	0.19	0.0	0.4	0.06	0.21
14週	12	0.10	0.11	0.07	0.00	0.18	0.0	0.4	0.03	0.17
18週	12	0.09	0.09	0.05	0.00	0.17	0.0	0.3	0.03	0.15
22週	12	0.08	0.08	0.05	0.00	0.17	0.0	0.2	0.03	0.13
26週	12	0.08	0.08	0.05	0.00	0.17	0.0	0.2	0.03	0.14
30週	12	0.14	0.24	0.05	0.02	0.16	0.0	0.9	-0.01	0.29

各評価日のステロイド使用量から登録時のステロイド使用量を差し引いて算出した割合をステロイド使用量の変化率とした．ステロイド使用量の変化率の要約統計量を表 11.4－23 に示した．なお，TF 補完した場合のステロイド使用量の変化率の要約統計量を表 11.4－24 に示した．

ステロイド使用量の変化率（Median）は，投与 2，6，8 週ではそれぞれ-1.63，-25.05，-43.91%と経時的にその割合は大きくなった．投与 10～30 週では-61.72～-86.93%の間で推移した．また，最終は-85.44%であった．

なお，TF 補完を行った場合では，投与 2，6，8 週では，それぞれ-1.63，-25.05，-43.91%であり，投与 10～30 週では-45.01～-79.31%で推移した．補完前のデータの推移と同様の傾向であった．本剤を投与することにより，ステロイド使用量を減らすことができる可能性が示唆された．

表 11.4−23　ステロイド使用量変化率（%）の要約統計量（FAS のうち登録時にステロイド（経口剤）を使用していた被験者）

	ステロイド使用量変化率（%）							
	n	Mean	SD	Median	Q1	Q3	Min	Max
2週	12	-10.76	28.22	-1.63	-5.51	-1.17	-100.0	0.7
6週	12	-39.00	40.11	-25.05	-73.31	-4.36	-100.0	-0.6
8週	12	-44.96	39.72	-43.91	-78.94	-4.28	-100.0	1.1
10週	10	-55.39	38.56	-61.72	-100.00	-28.64	-100.0	0.6
14週	9	-64.39	40.15	-77.97	-100.00	-47.89	-100.0	0.0
18週	9	-71.25	34.18	-77.00	-100.00	-64.79	-100.0	-5.4
22週	8	-75.26	34.12	-86.93	-100.00	-63.22	-100.0	-1.8
26週	8	-68.15	39.87	-79.23	-100.00	-41.29	-100.0	-4.2
30週	8	-58.85	41.36	-73.26	-94.07	-18.79	-100.0	1.4
最終	12	-64.14	42.98	-85.44	-100.00	-18.79	-100.0	1.4

表 11.4−24　ステロイド使用量変化率（%）（TF）の要約統計量（FAS のうち登録時にステロイド（経口剤）を使用していた被験者）

	ステロイド使用量変化率（%）（TF）							
	n	Mean	SD	Median	Q1	Q3	Min	Max
2週	12	-10.76	28.22	-1.63	-5.51	-1.17	-100.0	0.7
6週	12	-39.00	40.11	-25.05	-73.31	-4.36	-100.0	-0.6
8週	12	-44.96	39.72	-43.91	-78.94	-4.28	-100.0	1.1
10週	12	-46.07	41.12	-45.01	-83.86	-1.50	-100.0	1.1
14週	12	-56.53	44.58	-75.37	-100.00	0.00	-100.0	1.1
18週	12	-61.68	41.98	-76.73	-100.00	-15.83	-100.0	1.1
22週	12	-58.42	45.24	-79.31	-100.00	-0.90	-100.0	1.1
26週	12	-53.68	46.43	-75.38	-100.00	-2.10	-100.0	1.1
30週	12	-47.48	45.37	-47.38	-94.07	0.00	-100.0	1.4

登録時にステロイド（経口剤）を使用していた被験者 12 名のうち，各評価日のステロイド使用量が 0 となった場合をステロイド離脱と定義した．各評価時点のステロイド離脱率を表 11.4−25 に，TF 補完した場合のステロイド離脱率を表 11.4−26 に示した．

ステロイド離脱率は，投与 2 週で 8.3%，投与 6，8 週で共に 25.0%とその割合は増加した．投与 10～30 週では 25.0～37.5%で推移した．また，最終は 41.7%であった．

なお，TF 補完を行った場合では，投与 2 週で 8.3%，投与 6，8 週で共に 25.0%とその割合は増加し，投与 10～30 週では 16.7～25.0%で推移した．補完前のデータの推移と同様の傾向であった．

投与 30 週時点でステロイド離脱した被験者は 2 名であり，そのうち 1 名が CAI スコア寛解に該当していた．また，CAI スコア寛解に該当しなかった 1 名も投与 30 週以外の多くの時点において CAI スコア寛解を呈していた．

表 11.4－25　ステロイド離脱率（%）
（FAS のうち登録時にステロイド（経口剤）
を使用していた被験者）

	ステロイド離脱率		
	n	離脱	%
2週	12	1	8.3
6週	12	3	25.0
8週	12	3	25.0
10週	10	3	30.0
14週	9	3	33.3
18週	9	3	33.3
22週	8	3	37.5
26週	8	3	37.5
30週	8	2	25.0
最終	12	5	41.7

表 11.4－26　ステロイド離脱率（%）（TF）
（FAS のうち登録時にステロイド（経口剤）
を使用していた被験者）

	ステロイド離脱率（TF）		
	n	離脱	%
2週	12	1	8.3
6週	12	3	25.0
8週	12	3	25.0
10週	12	2	16.7
14週	12	3	25.0
18週	12	3	25.0
22週	12	3	25.0
26週	12	3	25.0
30週	12	2	16.7

11.4.1.4　その他の臨床検査項目

CRP（mg/dL）について表 11.4－27 に示した.

登録時の CRP（Median）は，0.2 mg/dL であり，投与 2 週以降はすべての時点で 0.00 mg/dL であった.

表 11.4－27　CRP（FAS）

	CRP（mg/dL）							
	n	Mean	SD	Median	Q1	Q3	Min	Max
0週	21	1.75	3.68	0.20	0.00	0.60	0.0	15.1
2週	20	0.22	0.53	0.00	0.00	0.10	0.0	2.1
6週	20	0.28	0.98	0.00	0.00	0.10	0.0	4.4
8週	19	0.29	0.65	0.00	0.00	0.40	0.0	2.7
10週	18	0.36	0.62	0.00	0.00	0.30	0.0	1.8
14週	16	0.08	0.12	0.00	0.00	0.10	0.0	0.4
18週	16	0.26	0.53	0.00	0.00	0.15	0.0	1.7
22週	14	0.13	0.29	0.00	0.00	0.10	0.0	1.1
26週	14	0.06	0.12	0.00	0.00	0.10	0.0	0.4
30週	14	0.09	0.14	0.00	0.00	0.10	0.0	0.5

11.4.2 統計・解析上の論点

11.4.2.1 共変量による調整

本治験は非対照試験であり，該当しなかった．

11.4.2.2 脱落又は欠測値の取扱い

脱落又は欠測値の取扱いは，事前に規定した「9.7.1.2 データの取扱い」に準じた．

11.4.2.3 中間解析及びデータモニタリング

本治験では中間解析は計画しなかった．

11.4.2.4 多施設共同治験

本治験は多施設共同にて実施し，17 施設において FAS 採用被験者があった．実施医療機関別の被験者数を表 11.4－28 に示した．本治験では，1 施設あたり 1～2 名と少数であったため，施設間交互作用による検討は行わなかった．

表 11.4－28　実施医療機関別の被験者数

実施医療機関名	登録被験者数
ＪＡ北海道厚生連札幌厚生病院	1
医療法人徳洲会札幌東徳洲会病院	1
国立大学法人群馬大学医学部附属病院	1
埼玉県立小児医療センター	2
東邦大学医療センター佐倉病院	1
順天堂大学医学部附属順天堂医院	1
独立行政法人地域医療機能推進機構東京山手メディカルセンター	1
独立行政法人国立成育医療研究センター	2
公立大学法人横浜市立大学附属市民総合医療センター	2
大阪市立大学医学部附属病院	1
地方独立行政法人大阪府立病院機構大阪府立急性期・総合医療センター	1
大阪医科大学附属病院	1
兵庫医科大学病院	2
九州大学病院	1
福岡大学筑紫病院	1
久留米大学病院	1
大分赤十字病院	1
計	21

11.4.2.5 多重比較・多重性

本治験では検定を実施しないので，多重比較・多重性の問題は発生しなかった．

11.4.2.6 患者の「有効性評価の部分集団」の使用

本治験では有効性評価の部分集団を使用した解析は実施しなかった．

11.4.2.7 同等性を示すことを意図した実対照薬を用いた試験

本治験は非対照試験であり，該当しなかった．

11.4.2.8 部分集団の検討

本治験の有効性解析対象被験者が 21 名と少数であることから，統計的な観点から部分集団の検討は行わなかったが，有効性解析対象被験者を 6～12 歳未満と 12～17 歳以下の 2 つの集団に分け，年齢別の 0 週（登録時），投与 8 週の CAI スコア，パーシャル Mayo スコア，PUCAI スコアの Median，投与 8 週の各スコア変化量，CAI スコア及び PUCAI スコアの寛解率を表 11.4－29 に示した．

6～12 歳未満（以下，低年齢群），12～17 歳以下（以下，高年齢群）では，被験者数がそれぞれ 4，17 名と両集団で大きく異なるものの，0 週の CAI スコア，パーシャル Mayo スコア及び PUCAI スコアは各群においてほぼ同程度であった．また，投与 8 週の各スコア及び変化量，CAI スコア及び PUCAI スコアの寛解率においても各群の被験者数に違いはあったものの，

各群における有効性に関して，大きく異なるものではないと考えられた．

表 11.4－29　年齢別の有効性評価の要約

			年齢別（歳）					
			6～12未満			12～17以下		
評価項目			n	Median	寛解（%）	n	Median	寛解（%）
CAI スコア	スコア	0週	4	10.0		17	9.0	
		8週	3	3.0		17	3.0	
	変化量	8週	3	-6.0		17	-6.0	
	寛解率	8週	3		3（100.0）	17		13（76.5）
パーシャル Mayo スコア	スコア	0週	4	6.5		17	6.0	
		8週	3	0.0		17	2.0	
	変化量	8週	3	-4.0		17	-3.0	
PUCAI スコア	スコア	0週	4	52.5		17	45.0	
		8週	3	0.0		17	10.0	
	変化量	8週	3	-45.0		17	-40.0	
	寛解率	8週	3		2（66.7）	17		6（35.3）

11.4.3 個別反応データの作表

個々の被験者における各評価時期の有効性評価データを付録 16.2.6 に添付した．

11.4.4 薬剤の用量，薬物濃度及びそれらと反応との関係

11.4.4.1 血清中インフリキシマブ濃度

各時点における血清中インフリキシマブ濃度の推移を表 11.4－30 に示した．なお，各被験者は 0 週（治験薬投与開始日），投与 2，6 週時に治験薬を 5 mg/kg にて投与された．その後，投与 8 週の CAI スコアの結果から，CAI スコア応答例又は不応例の判定を行い，CAI スコア応答例のみ投与 14，22 週に治験薬を 5 mg/kg で投与された．また，CAI スコア不応例については，投与 8 週評価終了後，併用禁止薬剤・療法の制限がなくなることから全被験者（全体）の集計は投与 8 週までとした．

全体及び CAI スコア応答例の 0 週投与終了 1 時間後の血清中インフリキシマブ濃度（Median）はそれぞれ 97.17，96.35 μg/mL であり，CAI スコア不応例（2 名）ではそれぞれ 106.65，124.05 μg/mL であった．また，全体及び CAI スコア応答例の投与 8 週の血清中インフリキシマブ濃度（Median）は共に 25.64 μg/mL であり，CAI スコア不応例（2 名）ではそれぞれ 13.74，28.09 μg/mL であった．CAI スコア応答例におけるトラフの血清中インフリキシマブ濃度（Median）である投与 14，22 及び 30 週では，それぞれ 2.58，1.54，1.34 μg/mL と血清中インフリキシマブ濃度を維持していた．また，CAI スコア応答例の 0 週，投与 14，22 週の投与終了 1 時間後の血清中インフリキシマブ濃度はそれぞれ 96.35，95.91，102.78 μg/mL と大きく異ならず，これらのことを勘案すると小児の被験者において本剤を複数回投与する

ことによる薬剤の蓄積性はないと考えた．一方，CAI スコア不応例（2 名）の投与 14 週の血清中インフリキシマブ濃度はそれぞれ 0.26，1.74 μg/mL であり，CAI スコア応答例の投与 14 週の血清中インフリキシマブ濃度（Median）より低かった．

表 11.4－30　血清中インフリキシマブ濃度（μg/mL）

		n	Mean	SD	Median	Q1	Q3	Min	Max
全体	0週投与前	21	0.00	0.00	0.00	0.00	0.00	0.00	0.00
	0週投与終了1時間後	21	96.18	15.65	97.17	80.06	107.43	65.81	124.05
	2週投与前	20	21.75	5.46	21.14	18.91	24.30	9.94	36.10
	6週投与前	20	12.34	7.51	10.35	6.64	16.55	1.70	30.17
	8週	19	26.36	9.70	25.64	18.13	34.72	11.66	43.15
CAIスコア応答例	0週投与前	18	0.00	0.00	0.00	0.00	0.00	0.00	0.00
	0週投与終了1時間後	18	95.04	14.70	96.35	80.06	107.43	65.81	114.71
	2週投与前	18	21.46	5.36	21.14	18.97	24.12	9.94	36.10
	6週投与前	18	12.27	7.42	10.35	6.79	15.08	1.70	30.17
	8週	17	27.00	9.77	25.64	20.65	34.72	11.66	43.15
	10週	18	12.78	7.03	13.81	6.85	17.31	1.98	28.80
	14週投与前	18	3.30	3.03	2.58	0.80	4.09	0.00	10.92
	14週投与終了1時間後	16	100.32	19.25	95.91	82.63	118.82	74.03	130.68
	18週	16	11.49	7.00	10.32	5.52	16.63	2.79	23.33
	22週投与前	15	2.68	3.02	1.54	0.33	4.74	0.00	10.02
	22週投与終了1時間後	14	103.29	18.61	102.78	83.78	116.62	72.85	137.61
	26週	14	10.50	8.45	8.13	5.05	17.32	0.00	27.17
	30週	14	2.74	3.52	1.34	0.00	4.80	0.00	12.27
CAIスコア不応例	0週投与前	2	NC	NC	NC	NC	NC	0.00	0.00
	0週投与終了1時間後	2	NC	NC	NC	NC	NC	106.65	124.05
	2週投与前	2	NC	NC	NC	NC	NC	18.84	30.04
	6週投与前	2	NC	NC	NC	NC	NC	4.87	21.19
	8週	2	NC	NC	NC	NC	NC	13.74	28.09
	10週	2	NC	NC	NC	NC	NC	5.08	6.32
	14週	2	NC	NC	NC	NC	NC	0.26	1.74

NC：算出不能

各時点での血清中インフリキシマブ濃度の定量下限（0.1 μg/mL）未満の割合を表 11.4－31 に示した．治験薬投与後の血清中インフリキシマブ濃度が定量下限未満を示したのは，CAI スコア応答例の投与 14 週投与前で 5.6%（1/18 名），投与 22 週投与前で 6.7%（1/15 名），投与 26 週で 7.1%（1/14 名），投与 30 週で 28.6%（4/14 名）のみであり，多くの被験者で定量下限以上の血清中インフリキシマブ濃度を維持していた．また，CAI スコア不応例では，治験薬投与後に血清中インフリキシマブ濃度が定量下限未満を示した被験者はなかった．

表 11.4－31　血清中インフリキシマブ濃度の定量下限（0.1 μg/mL）未満の割合

		n	BLQ	%
全体	0週投与前	21	21	100.0
	0週投与終了1時間後	21	0	0.0
	2週投与前	20	0	0.0
	6週投与前	20	0	0.0
	8週	19	0	0.0
CAIスコア応答例	0週投与前	18	18	100.0
	0週投与終了1時間後	18	0	0.0
	2週投与前	18	0	0.0
	6週投与前	18	0	0.0
	8週	17	0	0.0
	10週	18	0	0.0
	14週投与前	18	1	5.6
	14週投与終了1時間後	16	0	0.0
	18週	16	0	0.0
	22週投与前	15	1	6.7
	22週投与終了1時間後	14	0	0.0
	26週	14	1	7.1
	30週	14	4	28.6
CAIスコア不応例	0週投与前	2	2	100.0
	0週投与終了1時間後	2	0	0.0
	2週投与前	2	0	0.0
	6週投与前	2	0	0.0
	8週	2	0	0.0
	10週	2	0	0.0
	14週	2	0	0.0

BLQ : 定量限界値未満

11.4.4.2 血清中インフリキシマブ濃度と有効性評価項目との関連性

CAIスコア寛解／非寛解別の投与8週の血清中インフリキシマブ濃度を表 11.4－32に示した．全被験者を対象とした時のCAIスコア寛解及び非寛解における血清中インフリキシマブ濃度（Median）は，それぞれ24.42，26.87 μg/mLであった．また，CAIスコア寛解／非寛解別の投与30週の血清中インフリキシマブ濃度を表 11.4－33に示した．CAIスコア寛解及び非寛解における血清中インフリキシマブ濃度（Median）は，それぞれ1.20，1.48 μg/mLであった．これらの結果から，CAIスコア寛解及び非寛解における血清中インフリキシマブ濃度（Median）に大きな差異は認められず，被験者数も少ないことから有効性と血清中インフリキシマブ濃度との関連性は明確にはできなかった．

表 11.4－32 CAI スコア寛解／非寛解別の投与 8 週の血清中インフリキシマブ濃度(µg/mL)

		血清中インフリキシマブ濃度（µg/mL）							
		n	Mean	SD	Median	Q1	Q3	Min	Max
全体	寛解	15	26.30	9.91	24.42	18.13	34.72	11.66	43.15
	非寛解	4	26.59	10.31	26.87	19.69	33.48	13.74	38.87
CAIスコア応答例	寛解	15	26.30	9.91	24.42	18.13	34.72	11.66	43.15
	非寛解	2	NC	NC	NC	NC	NC	25.64	38.87
CAIスコア不応例	寛解	-	-	-	-	-	-	-	-
	非寛解	2	NC	NC	NC	NC	NC	13.74	28.09

NC：算出不能

表 11.4－33 CAI スコア寛解／非寛解別の投与 30 週の血清中インフリキシマブ濃度（µg/mL）

	血清中インフリキシマブ濃度（µg/mL）							
	n	Mean	SD	Median	Q1	Q3	Min	Max
寛解	9	3.10	4.12	1.20	0.00	4.80	0.00	12.27
非寛解	5	2.10	2.37	1.48	0.25	2.98	0.00	5.78

投与 8，30 週の血清中インフリキシマブ濃度（µg/mL）別の CAI スコア変化量を表 11.4－34，表 11.4－35 に示した．投与 8 週の CAI スコア変化量（Median）ではすべての被験者が 10 µg/mL 以上を示したため, 血清中インフリキシマブ濃度と CAI スコア変化量の関連性は明確ではなかった. また, 0.1 µg/mL 未満, 0.1 以上 1 µg/mL 未満, 1 以上 10 µg/mL 未満, 10 µg/mL 以上の血清中インフリキシマブ濃度別での投与 30 週の CAI スコア変化量（Median）は，それぞれ-4.5，NC，-6.0，NC であった．NC を呈した濃度の集団の個別の CAI スコア変化量は，0.1 以上 1 µg/mL 未満で-4，-3，10 µg/mL 以上で-10 であった．これらの結果から，被験者数が少ない血清中インフリキシマブ濃度の集団があるものの，血清中インフリキシマブ濃度が高いほど CAI スコアの変化量が大きい傾向が認められた．

表 11.4－34 投与 8 週の血清中インフリキシマブ濃度（µg/mL）別の CAI スコア変化量

		CAIスコア変化量							
	血清中インフリキシマブ濃度（µg/mL）	n	Mean	SD	Median	Q1	Q3	Min	Max
全体	0.1未満	-	-	-	-	-	-	-	-
	0.1以上1未満	-	-	-	-	-	-	-	-
	1以上10未満	-	-	-	-	-	-	-	-
	10以上	19	-6.2	3.8	-6.0	-8.0	-5.0	-13	2
CAIスコア応答例	0.1未満	-	-	-	-	-	-	-	-
	0.1以上1未満	-	-	-	-	-	-	-	-
	1以上10未満	-	-	-	-	-	-	-	-
	10以上	17	-7.0	2.9	-6.0	-8.0	-5.0	-13	-2
CAIスコア不応例	0.1未満	-	-	-	-	-	-	-	-
	0.1以上1未満	-	-	-	-	-	-	-	-
	1以上10未満	-	-	-	-	-	-	-	-
	10以上	2	NC	NC	NC	NC	NC	0	2

NC：算出不能

表 11.4－35　投与 30 週の血清中インフリキシマブ濃度（μg/mL）別の CAI スコア変化量

	血清中インフリキシマブ濃度（μg/mL）	CAIスコア変化量 n	Mean	SD	Median	Q1	Q3	Min	Max
CAIスコア応答例	0.1未満	4	-4.3	3.5	-4.5	-7.0	-1.5	-8	0
	0.1以上1未満	2	NC	NC	NC	NC	NC	-4	-3
	1以上10未満	7	-7.0	4.0	-6.0	-10.0	-4.0	-14	-2
	10以上	1	NC	NC	NC	NC	NC	-10	-10

NC : 算出不能

11.4.4.3 ATI

ATI の判定結果を表 11.4－36 に示した．また，被験者ごとの ATI 測定結果を付録 16.1.13e に示した．全体では，評価不能が 81.0%（17/21 名），陰性が 19.0%（4/21 名）であり，陽性を示した被験者はなかった．CAI スコア応答例では，評価不能が 77.8%（14/18 名），陰性が 22.2%（4/18 名）であり，CAI スコア不応例では，評価不能が 100.0%（2/2 名），陰性は 0.0%（0/2 名）であった．

表 11.4－36　ATI の判定

	陰性	陽性	評価不能
全体*1	19.0% (4/21)	0.0% (0/21)	81.0% (17/21)
CAIスコア応答例*2	22.2% (4/18)	0.0% (0/18)	77.8% (14/18)
CAIスコア不応例*3	0.0% (0/2)	0.0% (0/2)	100.0% (2/2)

*1　ATI判定をした全被験者における全期間
*2　30週まで
*3　14週まで

11.4.5 薬物－薬物及び薬物－疾患の相互作用

本治験において薬物－薬物及び薬物－疾患の相互作用については検討していない．

11.4.6 患者ごとの表示

本治験における被験者ごとのデータは付録 16.2.4 に添付した．

11.4.7 有効性の結論

(1) CAI スコア（Mean±SD）は，登録時には 9.7±2.7 であったが，投与 2，6，8 週ではそれぞれ 4.0±3.2，3.5±3.5，3.2±3.5 と経時的に減少した．投与 10～30 週では 2.5±2.3～3.5±2.2 で推移した．また，最終は 5.6±3.8 であった．CAI スコア変化量（Mean±SD）は，投与 2，6，8 週で-5.5±2.7，-6.0±4.0，-6.2±3.7 と大きくなり，投与 10～30 週では-5.9±3.8～-6.9±3.8 の間で推移した．また，最終では-4.1±4.2 であった．

CAI スコア及び CAI スコア変化量共に，投与 2 週時点から症状改善効果が認められ，その効果が投与 30 週まで持続した．なお，TF 補完を行った場合でも，CAI スコア及び CAI スコア変化量共に，TF 補完前のデータと同様の傾向を示した．

(2) CAI スコア寛解率は，投与 2 週で 60.0%（12/20 名）となり，投与 6，8 週共に 80.0%（16/20 名）と増加し，投与 10～30 週までの間では 64.3～87.5%で推移した．また，最終では 42.9%（9/21 名）であった．CAI スコア寛解率において，本剤を投与することにより，投与 2 週から寛解の状態に導く効果が認められ，その効果が投与 30 週まで持続することが確認された．なお，TF 補完した場合も同様の傾向であった．

(3) パーシャル Mayo スコア（Mean±SD）は，登録時では 5.6±1.6 であったが，投与 2，6，8 週ではそれぞれ 2.6±2.3，2.2±2.0，1.7±1.7 と経時的に減少し，投与 10～30 週では 1.8±1.7～2.8±1.9 の間で推移した．また，最終では 3.7±2.2 であった．パーシャル Mayo スコア変化量（Mean±SD）は，投与 2，6，8 週ではそれぞれ-2.9±2.2，-3.3±2.2，-3.8±1.7 と経時的に大きくなり，投与 10～30 週では-2.6±2.8～-3.6±2.5 の間で推移した．また，最終では-1.9±2.7 であった．パーシャル Mayo スコア及びパーシャル Mayo スコア変化量共に，投与 2 週時点から症状改善効果を認め，その効果が投与 30 週まで持続した．なお，TF 補完した場合もパーシャル Mayo スコア及びパーシャル Mayo スコア変化量では同様の傾向であった．

(4) Mayo スコア（Mean±SD）は，登録時では 7.0±2.4 であったが，投与 30 週では 4.9±2.4 に減少した．また，最終では 5.0±2.3 であった．Mayo スコア変化量（Mean±SD）は，投与 30 週に-2.6±3.9 であった．また，最終では-2.0±3.9 であった．なお，TF 補完した場合も Mayo スコア及び Mayo スコア変化量においても補完前と同様の傾向が確認された．

(5) Mayo スコアの改善率及び寛解率は，投与 30 週ではそれぞれ 42.9%（3/7 名），14.3%（1/7 名）であった．また，最終ではそれぞれ 37.5%（3/8 名），12.5%（1/8 名）であった．なお，TF 補完した場合も Mayo スコアの改善率及び寛解率においても補完前と同様の値を呈した．

(6) PUCAI スコア（Mean±SD）は，登録時では 47.1±15.2 であったが，投与 2，6，8 週ではそれぞれ 20.3±16.3，17.3±17.5，12.5±13.5 と経時的に減少した．投与 10～30 週では 12.2±12.0～19.3±18.8 の間で推移した．また，最終では 28.8±22.5 であった．PUCAI スコア変化量（Mean±SD）は，投与 2，6，8 週でそれぞれ-25.8±17.5，-28.8±21.1，-33.5±14.0 を示した．投与 10～30 週では-26.8±27.8～-34.1±17.7 の間で推

移した．また，最終では-18.3±28.4 であった．PUCAI スコア及び PUCAI スコア変化量共に, 投与 2 週時点から症状改善効果を認め, その効果が投与 30 週まで持続した．なお，TF 補完した場合も，PUCAI スコア及び PUCAI スコア変化量については，補完前と同様の傾向であった．

(7) PUCAI スコア寛解率は，投与 2 週で 35.0%（7/20 名）となり，投与 6，8 週共に 40.0%（8/20 名）と増加した．その後，投与 10～30 週までの間では 28.6～42.9%で推移した．また，最終では 19.0%（4/21 名）となった．投与 2 週から寛解の状態へ導く効果を認め，その効果は持続することが確認された．なお，TF 補完した場合も同様の傾向であった．

(8) PUCAI スコア変化量が 20 ポイント以上減少した被験者の割合は，投与 2 週で 68.4%（13/19 名）となり，投与 6，8 週ではそれぞれ 73.7%（14/19 名），89.5%（17/19 名）と増加した．その後，投与 10～30 週までの間では 64.3～88.2%の間で推移した．また，最終では 60.0%（12/20 名）であった．投与 2 週から改善効果を認め，その効果はその後も持続することが確認された．なお，TF 補完した場合も同様の傾向であった．

(9) 粘膜治癒率において，投与 30 週及び最終ではそれぞれ 33.3%（2/6 名），28.6%（2/7 名）であった．なお，TF 補完した場合も同様の傾向であった．

(10) 登録時にステロイド（経口剤）を使用していた被験者 12 名において，ステロイド使用量（Median）は，登録時に 0.20 mg/kg/day であったが，投与 2，6，8 週ではそれぞれ 0.19, 0.17, 0.16 mg/kg/day と減少した. また, 投与 10～30 週では 0.04～0.15 mg/kg/day の間で推移した．また，最終では 0.05 mg/kg/day であった．ステロイド使用量の変化率（Median）は，投与 2，6，8 週ではそれぞれ-1.63，-25.05，-43.91%と経時的にその割合は大きくなった．投与 10～30 週では-61.72～-86.93%の間で推移した．また，最終は-85.44%であった．なお，TF 補完した場合もステロイド使用量及びステロイド使用量の変化率では同様の傾向であり，本剤投与により，ステロイドを減量できる可能性が示唆された．

(11) ステロイド離脱率は，投与 2 週で 8.3%，投与 6，8 週で共に 25.0%とその割合は増加した．投与 10～30 週では 25.0～37.5%で推移した．また，最終は 41.7%であった．なお，TF 補完した場合も，その推移は補完前のデータと同様の傾向であった．投与 30 週時点でステロイド離脱した被験者は 2 名であり，そのうち 1 名が CAI スコア寛解に該当していた．

(12) 有効性解析対象被験者を 6～12 歳未満と 12～17 歳以下の 2 つの集団に分けて有効性について検討した結果，両集団の被験者数に違いはあったものの，両集団における有効性に関して，大きく異なるものではないと考えられた．

(13) CAI スコア応答例におけるトラフの血清中インフリキシマブ濃度（Median）である投与 14，22 及び 30 週では，それぞれ 2.58，1.54，1.34 μg/mL と血清中インフリキシマブ濃度を維持していた．

(14) 投与 30 週の CAI スコア変化量（Median）は，0.1 μg/mL 未満，0.1 以上 1 μg/mL 未満，1 以上 10 μg/mL 未満，10 μg/mL 以上でそれぞれ-4.5，NC（算出不能），-6.0，NC であった．NC を呈した濃度の集団の個別の CAI スコア変化量は，0.1 以上 1 μg/mL 未満で-4，-3，10 μg/mL 以上で-10 であった．これらの結果から，被験者数が少ない血清中インフリキシマブ濃度の集団があるものの，血清中インフリキシマブ濃度が高いほど有効性も高くなる傾向が認められた．

(15) ATI の判定は，治験期間中，全体において，評価不能が 81.0%（17/21 名），陰性が 19.0%（4/21 名）であり，陽性を示した被験者はなかった．

以上，本剤を 5 mg/kg で 0，2，6 週に投与することにより，中等症から重症の小児の潰瘍性大腸炎に対する臨床症状及び粘膜治癒の改善効果が得られることが示された．その後，14，22 週と 8 週間隔で継続して投与することにより，その効果が持続することが確認された．また，本剤を投与することにより，ステロイドを減量又は離脱できる可能性が示唆された．

12. 安全性の評価

12.1 治験薬が投与された被験者数，期間及び用量

安全性解析対象集団は治験薬を少なくとも 1 回投与され，かつ，治験薬投与開始後の安全性データが得られた 21 名とした.

安全性評価期間の分布を表 12.1－1 に示した．安全性評価期間の平均値は 176.7 日で，最小値が 61 日，最大値が 239 日であった.

表 12.1－1 安全性評価期間（日）の分布

度数分布				要約統計量			
55日以下	56～97日	98～153日	154日以上	n	Mean	Min	Max
0	5	2	14	21	176.7	61	239

投与回数の度数分布を表 12.1－2 に示した．投与回数の平均値は 4.3 回で，最小である投与 1 回が 1 名であり，ほとんどの被験者は最大の投与 5 回であった.

表 12.1－2 被験者あたりの投与回数の度数分布

1回	2回	3回	4回	5回	Mean
1	0	4	2	14	4.3

12.2 有害事象

12.2.1 有害事象の簡潔な要約

安全性解析対象集団の有害事象発現率を表 12.2－1 に示した.

有害事象の発現率は 95.2%（20/21 名）であり，副作用の発現率は 71.4%（15/21 名）であった．重篤な有害事象の発現率は 14.3%（3/21 名）であり，重篤な副作用の発現率は 4.8%（1/21 名）であった．中止に至った有害事象の発現率は 4.8%（1/21 名）であったが，中止に至った副作用は認められなかった．感染症の発現率は 61.9%（13/21 名）であり，感染症（副作用）の発現率は 23.8%（5/21 名）であった．そのうち，重篤な感染症及び重篤な感染症（副作用）の発現率は共に 4.8%（1/21 名）であった．投与中止に至った感染症は認められなかった．Infusion reaction 及び Infusion reaction（副作用）の発現率は共に 9.5%（2/21 名）であった．重篤な Infusion reaction 及び投与中止に至った Infusion reaction は認められなかった.

表 12.2−1　有害事象発現率

MedDRA version: 17.1

	(N=21) n (%)
有害事象	20 (95.2)
有害事象（SOC=臨床検査以外）	19 (90.5)
副作用	15 (71.4)
副作用（SOC=臨床検査以外）	9 (42.9)
重篤な有害事象	3 (14.3)
重篤な副作用	1 (4.8)
中止に至った有害事象	1 (4.8)
中止に至った副作用	0 (0.0)
感染症	13 (61.9)
感染症（副作用）	5 (23.8)
重篤な感染症	1 (4.8)
重篤な感染症（副作用）	1 (4.8)
投与中止に至った感染症	0 (0.0)
Infusion reaction	2 (9.5)
Infusion reaction（副作用）	2 (9.5)
重篤なInfusion reaction	0 (0.0)
投与中止に至ったInfusion reaction	0 (0.0)
有害事象（免疫血清学的検査）	12 (57.1)

12.2.2 有害事象の表示

有害事象名はICH国際医薬用語集日本語版（MedDRA/J）Version 17.1に従って下層語（LLT）に読み替えた．集計には基本語（PT）を使用し，器官別大分類（SOC）を用いて器官分類を行った．

有害事象の一覧を表 12.2−2 に，副作用の一覧を表 12.2−3 に示した．

表 12.2−2　有害事象

MedDRA version: 17.1　　SOC-PT 集計

SOC<br>PT	(N=21)<br>n	 (%)	 件数
計	20	(95.2)	71
感染症および寄生虫症	12	(57.1)	23
鼻咽頭炎	7	(33.3)	13
上気道感染	2	(9.5)	4
感染性腸炎	2	(9.5)	2
気管支炎	1	(4.8)	1
胃腸炎	1	(4.8)	1
副鼻腔炎	1	(4.8)	1
皮下組織膿瘍	1	(4.8)	1

表 12.2－2　有害事象（続き）

MedDRA version: 17.1　　　　SOC-PT 集計

SOC<br>PT	(N=21) n	(%)	件数
血液およびリンパ系障害	3	(14.3)	3
鉄欠乏性貧血	2	(9.5)	2
貧血	1	(4.8)	1
神経系障害	1	(4.8)	1
頭痛	1	(4.8)	1
眼障害	1	(4.8)	1
強膜炎	1	(4.8)	1
血管障害	1	(4.8)	1
血管炎	1	(4.8)	1
呼吸器、胸郭および縦隔障害	4	(19.0)	4
喘息	1	(4.8)	1
アレルギー性鼻炎	1	(4.8)	1
上気道の炎症	1	(4.8)	1
口腔咽頭痛	1	(4.8)	1
胃腸障害	11	(52.4)	13
潰瘍性大腸炎	2	(9.5)	2
悪心	2	(9.5)	2
嘔吐	2	(9.5)	2
下腹部痛	1	(4.8)	1
上腹部痛	1	(4.8)	1
口唇炎	1	(4.8)	1
便秘	1	(4.8)	1
腸炎	1	(4.8)	1
膵炎	1	(4.8)	1
自己免疫性膵炎	1	(4.8)	1
皮膚および皮下組織障害	2	(9.5)	3
ざ瘡	2	(9.5)	2
そう痒症	1	(4.8)	1
筋骨格系および結合組織障害	3	(14.3)	4
関節痛	1	(4.8)	1
背部痛	1	(4.8)	1
筋肉痛	1	(4.8)	2
一般・全身障害および投与部位の状態	2	(9.5)	2
胸部不快感	1	(4.8)	1
注入部位疼痛	1	(4.8)	1
臨床検査	12	(57.1)	15
二本鎖ＤＮＡ抗体陽性	12	(57.1)	12
血圧低下	1	(4.8)	1
尿中蛋白陽性	1	(4.8)	1
抗核抗体増加	1	(4.8)	1
傷害、中毒および処置合併症	1	(4.8)	1
熱傷	1	(4.8)	1

表 12.2－3　副作用

MedDRA version: 17.1　　SOC-PT 集計

SOC<br>PT	(N=21) n	(%)	件数
計	15	(71.4)	27
感染症および寄生虫症	4	(19.0)	6
鼻咽頭炎	2	(9.5)	3
気管支炎	1	(4.8)	1
皮下組織膿瘍	1	(4.8)	1
感染性腸炎	1	(4.8)	1
血液およびリンパ系障害	1	(4.8)	1
鉄欠乏性貧血	1	(4.8)	1
胃腸障害	3	(14.3)	3
腸炎	1	(4.8)	1
悪心	1	(4.8)	1
自己免疫性膵炎	1	(4.8)	1
皮膚および皮下組織障害	1	(4.8)	1
ざ瘡	1	(4.8)	1
筋骨格系および結合組織障害	2	(9.5)	2
関節痛	1	(4.8)	1
筋肉痛	1	(4.8)	1
一般・全身障害および投与部位の状態	1	(4.8)	1
注入部位疼痛	1	(4.8)	1
臨床検査	12	(57.1)	13
二本鎖ＤＮＡ抗体陽性	12	(57.1)	12
抗核抗体増加	1	(4.8)	1

12.2.3 有害事象の分析

12.2.3.1 有害事象及び副作用

評価期間中に認められた有害事象及び副作用の一覧を表 12.2－2 及び表 12.2－3 に示した．20%以上に発現した有害事象の器官別大分類は,「感染症および寄生虫症」,「臨床検査」が共に 57.1%（12/21 名）,「胃腸障害」52.4%（11/21 名）であった．5%以上に発現した有害事象は,「二本鎖 DNA 抗体陽性」57.1%（12/21 名）,「鼻咽頭炎」33.3%（7/21 名）,「上気道感染」,「感染性腸炎」,「鉄欠乏性貧血」,「潰瘍性大腸炎」(医師記載名「潰瘍性大腸炎の悪化」),「悪心」,「嘔吐」,「ざ瘡」がそれぞれ 9.5%（2/21 名）であった．

また，20%以上に発現した副作用の器官別大分類は,「臨床検査」57.1%（12/21 名）であった．5%以上に発現した副作用は,「二本鎖 DNA 抗体陽性」57.1%（12/21 名）,「鼻咽頭炎」9.5%（2/21 名）であった．有害事象及び副作用共に「二本鎖 DNA 抗体陽性」及び「鼻咽頭炎」の発現率が高かった．

12.2.3.2 程度別の有害事象及び副作用（免疫血清学的検査以外）

程度別の有害事象及び副作用（免疫血清学的検査以外）を表 14.3－1 及び表 14.3－2 に示した．

有害事象の程度別判定は，治験責任（分担）医師が行った．被験者の日常生活への影響の観点から，軽度（被験者の日常生活に影響がない程度），中等度（被験者の日常生活にその事象が原因で多少の支障がある程度）及び高度（被験者の日常生活がその事象が原因でできない程度）に分類した．

程度別の有害事象の発現率は，軽度 61.9%（13/21 名），中等度 23.8%（5/21 名），高度 4.8%（1/21 名）であった．高度の有害事象は「潰瘍性大腸炎」で，原疾患の悪化と判断されており治験薬との因果関係は否定された．程度別の副作用の発現率は，軽度 23.8%（5/21 名），中等度 19.0%（4/21 名）であり，高度の副作用は認められなかった．

12.2.3.3 評価時期別の有害事象及び副作用

評価時期別の有害事象（器官別大分類「臨床検査」以外）及び副作用（器官別大分類「臨床検査」以外）の発現率を表 12.2－4 に示した．

「投与開始日（0 週）～投与 14 週投与前まで」の有害事象及び副作用の発現率は，それぞれ 76.2%（16/21 名）及び 28.6%（6/21 名）であり，そのうち感染症及び感染症（副作用）の発現率は，それぞれ 42.9%（9/21 名）及び 9.5%（2/21 名）であった．「投与開始日（0 週）～投与 14 週投与前まで」の有害事象及び副作用の発現率は，「投与 14 週投与以降～投与 22 週投与前まで」と「投与 22 週投与以降～投与 30 週評価日」の期間と比べて高かった．

一方，「投与 22 週投与以降～投与 30 週評価日」の有害事象及び副作用の発現率は，それぞれ 35.7%，0.0%であり，「投与 14 週投与以降～投与 22 週投与前まで」の 31.3%，18.8%と比較して，同程度もしくは大きく下回っており，投与回数増加に伴った有害事象又は副作用発現率の増加は認められなかった．評価時期別の感染症及び感染症（副作用）についても，有害事象及び副作用と同様の傾向であり，投与を繰り返すことで発現率の上昇は認められなかった．

表 12.2－4 評価時期別の有害事象（器官別大分類「臨床検査」以外）及び副作用（器官別大分類「臨床検査」以外）の発現率

MedDRA version: 17.1　　SOC-PT集計

	有害事象（臨床検査以外）						副作用（臨床検査以外）					
	有害事象			感染症			副作用			感染症（副作用）		
	n	発現	%	n	発現	%	n	発現	%	n	発現	%
投与開始日（0週）～14週投与前まで	21	16	76.2	21	9	42.9	21	6	28.6	21	2	9.5
14週投与*以降～22週投与前まで	16	5	31.3	16	5	31.3	16	3	18.8	16	3	18.8
22週投与*以降～30週評価日	14	5	35.7	14	1	7.1	14	0	0.0	14	0	0.0

臨床検査以外：有害事象のうちSOC=臨床検査を除いた事象

*：治験薬投与日に発現した有害事象は，Infusion reaction等の明らかに投与後に発現した有害事象を除き，治験薬投与前に発現したものとする

12.2.4 患者ごとの有害事象の一覧表

被験者ごとの有害事象一覧表を付録 16.2.7a に添付した．

12.3 死亡，その他の重篤な有害事象及び他の重要な有害事象

12.3.1 死亡，その他の重篤な有害事象及び他の重要な有害事象の一覧表

12.3.1.1 死亡

評価期間を通じて，死亡した被験者は認められなかった．

12.3.1.2 その他の重篤な有害事象

その他の重篤な有害事象として，原疾患悪化である「潰瘍性大腸炎」が 2 名，「腸炎」が 1 名に認められた．被験者ごとの重篤な有害事象一覧を付録 16.2.7b に添付した．

12.3.1.3 他の重要な有害事象

本治験では，投与中止に至った感染症及び投与中止に至った Infusion reaction を重要な有害事象として治験実施計画書に規定した．なお，感染症とは治験責任（分担）医師が感染症と判断した有害事象とし，Infusion reaction とは治験薬投与中又は投与終了後 2 時間以内に発現した有害事象とした．評価期間を通じて，重要な有害事象は認められなかった．

12.3.2 死亡，その他の重篤な有害事象及び他のいくつかの重要な有害事象の叙述

評価期間を通じて，死亡した被験者は認められなかった．その他の重篤な有害事象の叙述については，表 14.3－5 に示した．

12.3.3 死亡，その他の重篤な有害事象及び他の重要な有害事象の分析及び考察

12.3.3.1 死亡

評価期間を通じて，死亡した被験者は認められなかった．

12.3.3.2 その他の重篤な有害事象

重篤な有害事象及び重篤な副作用の一覧を表 12.3－1 及び表 12.3－2 に示した．

評価期間を通じて，14.3%（3/21 名）の重篤な有害事象が認められた．以下，重篤な有害事象の詳細を記載した．

「潰瘍性大腸炎」の 1 名は，13 歳の男児で，治験薬を 3 回投与後の投与 10 週評価時に腹痛と便回数の増加，血便が認められた．翌日より入院し内視鏡検査施行後，潰瘍性大腸炎の再燃が確認されたため，治験中止となった．中止理由は有害事象「潰瘍性大腸炎」（医師記載名「潰瘍性大腸炎の悪化」）の発現のためと判定された．その後，プレドニゾロン投与や血球成分除去療法の施行により症状は改善したため，退院となった．程度は中等度であった．本事象は原疾患の悪化であり，治験薬との因果関係は否定された．

「潰瘍性大腸炎」の他の 1 名は，17 歳の女児で，投与 8 週評価時に CAI スコア不応例と判定された．治験薬を 3 回投与後の投与 10 週評価時に便回数の増加，泥状便，発熱（38.0℃）が認められた．プレドニゾロンの増量及び血球成分除去療法を施行するも症状の悪化が認められ，ステロイドパルスと抗菌薬の併用のため，投与 10 週評価の 15 日後に入院となった．しかし，その後も症状の改善が見られないため，タクロリムス内服を開始した．大腸内視鏡検査の結果，びまん性の深掘れ潰瘍と偽ポリポーシスが認められ，血便持続，下痢頻回及び夜間腹痛増強が発現した．内服薬をすべて中止し，他院へ転院後手術（大腸亜全摘・回腸瘻造設術）が施行された．その後症状が改善したため，退院となった．程度は高度であった．本事象は原疾患の悪化であり，治験薬との因果関係は否定された．

「腸炎」の 1 名は，14 歳の男児で，治験薬を 4 回投与後の投与 18 週評価時に CRP の軽度上昇が確認された．その後，39℃の発熱や嘔吐，便回数の増加が確認され，就眠中に排便のために覚醒する状況であったことから，投与 18 週評価の 4 日後に入院となった．アデノウイルス腸炎が最も疑われたが，検査の結果アデノウイルス感染の診断に至らなかった．入院後は補液と水分摂取のみ実施され，薬剤治療なく自然経過で軽快し，退院となった．程度は中等度であった．発症への関与は否定できないため治験薬との因果関係は「関連あるかもしれない」と判断された．

なお，すべての事象の転帰は回復であった．

表 12.3－1　重篤な有害事象

MedDRA version: 17.1　　SOC-PT 集計

SOC<br>PT	(N=21)<br>n	 (%)	 件数
計	3	(14.3)	3
胃腸障害	3	(14.3)	3
潰瘍性大腸炎	2	(9.5)	2
腸炎	1	(4.8)	1

表 12.3－2　重篤な副作用

MedDRA version: 17.1	SOC-PT 集計		
SOC	(N=21)		
PT	n	(%)	件数
計	1	(4.8)	1
胃腸障害	1	(4.8)	1
腸炎	1	(4.8)	1

12.3.3.3 その他の重要な有害事象

本治験では，投与中止に至った感染症及び投与中止に至ったInfusion reaction（治験薬投与中又は投与終了後2時間以内に発現した有害事象）を重要な有害事象として治験実施計画書に規定した. 本項ではこれらの事象に加えて, 中止に至った有害事象, 感染症, Infusion reactionなどについて検討した.

12.3.3.3.1 中止に至った有害事象

中止に至った有害事象の一覧を表 12.3－3に示した．なお，中止に至った有害事象の特定は治験責任（分担）医師による判定を用いた.

中止に至った有害事象は，原疾患の悪化である「潰瘍性大腸炎」4.8%（1/21名）であった. なお，本事象の詳細は12.3.3.2に前述した.

評価期間を通じて，中止に至った副作用は認められなかった.

表 12.3－3　中止に至った有害事象

MedDRA version: 17.1	SOC-PT 集計		
SOC	(N=21)		
PT	n	(%)	件数
計	1	(4.8)	1
胃腸障害	1	(4.8)	1
潰瘍性大腸炎	1	(4.8)	1

12.3.3.3.2 感染症

感染症及び感染症（副作用）の一覧を表 12.3－4 及び表 12.3－5 に示した．なお，感染症の特定は治験責任（分担）医師による判定を用いた．

評価期間を通じた感染症の発現率は 61.9%（13/21 名）であった．20%以上に発現した感染症の器官別大分類は，「感染症および寄生虫症」57.1%（12/21 名）であった．5%以上に発現した感染症は，「鼻咽頭炎」33.3%（7/21 名）及び「上気道感染」，「感染性腸炎」が共に 9.5%（2/21 名）であった．

最も多く発現した感染症（副作用）の器官別大分類は，「感染症および寄生虫症」19.0%（4/21 名）であった．5%以上に発現した感染症（副作用）は，「鼻咽頭炎」9.5%（2/21 名）であった．

次に，重篤な感染症及び重篤な感染症（副作用）の一覧を表 14.3－3 及び表 14.3－4 に示した．

重篤な感染症及び重篤な感染症（副作用）は，共に「腸炎」4.8%（1/21 名）であった．詳細については，12.3.3.2 に前述した．

評価期間を通じて，投与中止に至った感染症は認められなかった．

表 12.3－4　感染症

MedDRA version: 17.1　　SOC-PT 集計

SOC<br>PT	(N=21) n	(%)	件数
計	13	(61.9)	26
感染症および寄生虫症	12	(57.1)	23
鼻咽頭炎	7	(33.3)	13
上気道感染	2	(9.5)	4
感染性腸炎	2	(9.5)	2
気管支炎	1	(4.8)	1
胃腸炎	1	(4.8)	1
副鼻腔炎	1	(4.8)	1
皮下組織膿瘍	1	(4.8)	1
呼吸器、胸郭および縦隔障害	1	(4.8)	1
上気道の炎症	1	(4.8)	1
胃腸障害	1	(4.8)	1
腸炎	1	(4.8)	1
皮膚および皮下組織障害	1	(4.8)	1
ざ瘡	1	(4.8)	1

表 12.3－5　感染症（副作用）

MedDRA version: 17.1　　SOC-PT 集計

SOC<br>PT	(N=21)<br>n	 (%)	 件数
計	5	(23.8)	8
感染症および寄生虫症	4	(19.0)	6
鼻咽頭炎	2	(9.5)	3
気管支炎	1	(4.8)	1
皮下組織膿瘍	1	(4.8)	1
感染性腸炎	1	(4.8)	1
胃腸障害	1	(4.8)	1
腸炎	1	(4.8)	1
皮膚および皮下組織障害	1	(4.8)	1
ざ瘡	1	(4.8)	1

12.3.3.3.3 Infusion reaction

Infusion reaction 及び Infusion reaction（副作用）の一覧を表 12.3－6 及び表 12.3－7 に示した．なお，Infusion reaction は治験薬投与中又は投与終了後 2 時間以内に発現した有害事象とした．

Infusion reaction 及び Infusion reaction（副作用）の発現率は，共に 9.5%（2/21 名）であり，「悪心」,「注入部位疼痛」が共に 4.8%（1/21 名）であった．程度は「悪心」が中等度で,「注入部位疼痛」が軽度と判定された．いずれの事象も発現日と同日で回復に至った．また，重篤な Infusion reaction 及び投与中止に至った Infusion reaction は認められなかった．

表 12.3－6　Infusion reaction

MedDRA version: 17.1　　SOC-PT 集計

SOC<br>PT	(N=21)<br>n	 (%)	 件数
計	2	(9.5)	2
胃腸障害	1	(4.8)	1
悪心	1	(4.8)	1
一般・全身障害および投与部位の状態	1	(4.8)	1
注入部位疼痛	1	(4.8)	1

表 12.3－7　Infusion reaction（副作用）

MedDRA version: 17.1　　SOC-PT 集計

SOC<br>PT	(N=21)<br>n	 (%)	 件数
計	2	(9.5)	2
胃腸障害	1	(4.8)	1
悪心	1	(4.8)	1
一般・全身障害および投与部位の状態	1	(4.8)	1
注入部位疼痛	1	(4.8)	1

投与回数別の Infusion reaction の発現率を表 12.3−8 に示した.

投与回数別の Infusion reaction は，1 回目及び 2 回目では認められず，3 回目以降に発現した．Infusion reaction 及び Infusion reaction（副作用）の発現率は，共に 3 回目では 5.0%（1/20 名），4 回目は 6.3%（1/16 名）であり，5 回目はいずれも発現しなかった.

以上より，投与を繰り返すごとに発現率が高まる傾向は認められなかった.

表 12.3−8 投与回数別の Infusion reaction の発現率

		n	発現	%
Infusion reaction	1回目	21	0	0.0
	2回目	20	0	0.0
	3回目	20	1	5.0
	4回目	16	1	6.3
	5回目	14	0	0.0
Infusion reaction（副作用）	1回目	21	0	0.0
	2回目	20	0	0.0
	3回目	20	1	5.0
	4回目	16	1	6.3
	5回目	14	0	0.0

投与 1 回あたりの Infusion reaction の発現率を表 12.3−9 に示した.

投与 1 回あたりの Infusion reaction 及び Infusion reaction（副作用）の発現率は，共に 2.2%（2/91 回）であった.

表 12.3−9 投与 1 回あたりの Infusion reaction の発現率

	全投与回数	発現回数	%
Infusion reaction	91	2	2.2
Infusion reaction（副作用）	91	2	2.2

全投与回数：全被験者の治験薬投与回数の合計
発現回数：Infusion reactionが認められた投与回数
%：発現回数／全投与回数×100

ATI 判定結果別（陰性，陽性，評価不能）の Infusion reaction の発現率を表 12.3−10 に示した.

Infusion reaction 及び Infusion reaction（副作用）は，いずれも ATI 評価不能の被験者のみに認められ，発現率は共に 11.8%（2/17 名）であった.

表 12.3－10　ATI 別の Infusion reaction の発現率

		n	発現	%
Infusion reaction	陰性	4	0	0.0
	陽性	0	-	-
	評価不能	17	2	11.8
Infusion reaction（副作用）	陰性	4	0	0.0
	陽性	0	-	-
	評価不能	17	2	11.8

12.3.3.3.4 その他

12.3.3.3.4.1 免疫血清学的検査

有害事象（免疫血清学的検査）の一覧を表 12.3－11 に示した．抗核抗体については，前値と比較して，抗体力価 3 ランク以上の悪化が認められた場合を有害事象として取り扱った．抗 dsDNA IgM 抗体，抗 dsDNA IgG 抗体については，陰性から陽性に変動した場合及び陽性から陽性（悪化方向）に変動した場合を有害事象として取り扱った．なお，抗 dsDNA IgG 抗体については 12 IU/mL を上回ったものを，抗 dsDNA IgM 抗体については 6 U/mL 以上を陽性と判定した．

有害事象（免疫血清学的検査）の発現率は 57.1%（12/21 名）であり，「二本鎖 DNA 抗体(IgM)陽性」57.1%（12/21 名），「抗核抗体増加」4.8%（1/21 名）であった．ループス様症候群に相関があるといわれている抗 dsDNA IgG 抗体の陽性化が認められた被験者はなかった．また，これらの有害事象（免疫血清学的検査）が認められた被験者を含め，ループス様症候群を発現した被験者は認められなかった．

表 12.3－11　有害事象（免疫血清学的検査）

	(N=21)		
	n	(%)	件数
計	12	(57.1)	13
免疫血清学的検査	12	(57.1)	13
抗核抗体↑	1	(4.8)	1
二本鎖DNA抗体(IgM)↑	12	(57.1)	12
二本鎖DNA抗体(IgG)↑	0	(0.0)	0

その他，本剤において留意すべき事象である悪性腫瘍（小児や若年成人で報告されている肝脾 T 細胞リンパ腫を含む），結核，脱髄疾患，間質性肺炎，肝機能障害，遅発性過敏反応（血清病様反応を含む），うっ血性心不全，重篤な血液障害及び横紋筋融解症は認められなかった．また，評価期間を通じて，女児被験者又は男児被験者の配偶者（パートナー）の妊娠に関する報告はなかった．

12.4 臨床検査値の評価

12.4.1 患者ごとの個々の臨床検査異常値の一覧表

被験者ごとの臨床検査値一覧（一般臨床検査，免疫血清学的検査）を付録 16.2.8a～付録 16.2.8e，付録 16.2.8g に添付した．

12.4.2 各臨床検査項目の評価

12.4.2.1 治験期間を通しての臨床検査値

臨床検査値の異常変動発現率を表 14.3－6 に示した．また，血液学的検査及び血液生化学的検査の各評価時点における要約統計量を表 14.3－7 に，尿検査（定性）の判定の度数分布を表 14.3－8 に示した．

なお，小児は年齢及び性別により臨床検査値の基準範囲が異なることから，臨床検査値の基準範囲は「新しい小児の臨床検査基準値ポケットガイド」[2)] の年齢及び性別ごとの基準値（同意取得時年齢を用いる）を用いた．この資料に基準範囲が記載されていない白血球分画は異常変動を判定しなかった．尿検査（定性）は，成人の基準範囲を小児に代用しても臨床的に問題ないと判断したため，臨床検査受託機関から提供された基準範囲（成人用）を用いて異常変動を判定した．

各検査項目の各評価時点における要約統計量は，被験者の年齢及び性別別に算出せず，全被験者の測定値から算出した．

尿検査を除いた一般臨床検査については, 以下に定義した異常変動を項目ごとに判定した．

- 0 週の検査値が基準範囲内（限界値を含む）であり，投与後のいずれかの値が基準範囲外（限界値を含まない）へ悪化し，かつ 0 週の検査値より 25%以上の変動
- 0 週の検査値が基準範囲外であり，投与後のいずれかの値が同方向へ悪化し，かつ 0 週の検査値より 25%以上の変動
- 0 週の検査値が基準範囲外であり，投与後のいずれかの値が逆方向の基準範囲外へ悪化
- 0 週の検査値が欠測であり，投与後のいずれかの値が基準範囲外

また，尿検査については，以下に定義した異常変動を項目ごとに判定した．

- 0 週の検査値が基準範囲内（限界値を含む）であり，投与後のいずれかの値が基準範囲外（限界値を含まない）へ悪化し，かつ 1 段階以上の変動
- 0 週の検査値が基準範囲外であり，投与後のいずれかの値が同方向へ悪化し，かつ 0 週の検査値より 1 段階以上の変動
- 0 週の検査値が基準範囲外であり，投与後のいずれかの値が逆方向の基準範囲外へ悪化
- 0 週の検査値が欠測であり，投与後のいずれかの値が基準範囲外

異常変動発現率が 10%以上認められた項目は，尿蛋白（増加），尿潜血（増加）が共に 47.6%（10/21 名），ALT（GPT）（減少）33.3%（7/21 名），白血球数（減少），ALP（減少），γ-GTP（減少），BUN（減少）がそれぞれ 14.3%（3/21 名）であった．

これらの 7 項目のうち，尿検査（尿蛋白，尿潜血）以外の 5 項目について，投与開始日（0 週）とそれ以降の測定値（Median）を比較した．ALT（GPT）は 0 週が 10.0 U/L で，投与 2 週以降は 9.0～12.0 U/L の間で推移した．白血球数は 0 週が 8600.0 /uL で，投与 2 週以降は 5800.0～7300.0 /uL の間で推移した. ALP は 0 週が 382.0 U/L で, 投与 2 週以降は 394.5～545.0 U/L の間で推移した．γ-GTP は 0 週が 13.0 U/L で，投与 2 週以降は 10.5～15.5 U/L の間で推移した．BUN は 0 週が 9.0 mg/dL で，投与 2 週以降は 11.0～13.0 mg/dL の間で推移した．これら 5 項目の測定値（Median）は，本治験に登録された 10～17 歳における基準範囲の最小値から最大値の範囲内で推移した．また，血液学的検査及び血液生化学的検査に関連した有害事象は認められなかった．

尿検査（尿蛋白，尿潜血）については，分布の推移に大きな変動は認められなかったが，尿検査に関連した有害事象として「尿中蛋白陽性」4.8%（1/21 名）が認められた．治験責任（分担）医師は被験者が生理中であったため，生理によるものと判断し，治験薬との因果関係は否定された．

以上，異常変動発現率が 10%以上認められた上記 7 項目の臨床検査において，臨床上問題となるような変動は認められなかった．

12.4.2.2 個々の患者の変化

被験者ごとの臨床検査値一覧を付録 16.2.8a～付録 16.2.8e，付録 16.2.8g に添付した．

12.4.2.3 個々の臨床的に重要な異常

一般臨床検査の異常変動のうち，重篤な事象や中止に至った事象は認められなかった．

12.5 バイタルサイン，身体的所見及び安全性に関連する他の観察項目

12.5.1 理学的検査（収縮期血圧，拡張期血圧，脈拍数，体温）

理学的検査（収縮期血圧，拡張期血圧，脈拍数，体温）の測定は，各投与日において投与開始直前，投与中は 30 分ごと，投与終了から 2 時間までは 30 分ごとに行った．投与終了時とその直前の測定との間隔が 15 分以上の場合は投与終了時にも測定を行った．

理学的検査の要約統計量の推移を表 14.3－9 に示した．また，被験者ごとの理学的検査の一覧を付録 16.2.8f に添付した．

本剤投与に際し測定された各理学的検査の平均値は，それぞれ収縮期血圧 99.6～111.3 mmHg，拡張期血圧 53.2～63.1 mmHg，脈拍数 75.6～88.3 拍/分，体温 36.45～37.06℃で推移し，0 週の投与前の平均値と比べ，大きな変動は認められなかった．

また，評価期間を通じて理学的検査に関連する事象として「血圧低下」4.8%（1/21 名）が認められた．なお，本事象は治験薬投与日の治験薬投与前に認められたため，治験薬との因果関係は否定された．

12.5.2 免疫血清学的検査

免疫血清学的検査（抗核抗体，抗 dsDNA IgG 抗体，抗 dsDNA IgM 抗体）の評価時期ごとの分布を表 12.5－1 に示した．また，被験者ごとの免疫血清学的検査値の一覧及び免疫血清学的検査における有害事象一覧を付録 16.2.8e 及び付録 16.2.7a に添付した．

表 12.5－1　免疫血清学的検査

		14週		30週		投与後の全時点*	
		陰	陽	陰	陽	陰	陽
抗核抗体	0週陰	7	2	5	1	7	2
	0週陽	1	10	1	7	1	11
抗dsDNAIgG抗体	0週陰	20		14		21	
	0週陽						
抗dsDNAIgM抗体	0週陰	11	8	4	10	9	11
	0週陽		1				1

0，14，30週の陽：陽性，陰：陰性
*陽:30週評価完了例の測定時点(14週，30週)，中止例の測定時点(中止日，最終投与8週又は，14週，中止日，最終投与8週)において，いずれかの時点で陽性，CAIスコア不応例の測定時点(14週)において陽性
陰:全時点で陰性

12.5.2.1 抗核抗体

投与開始日（0 週）の抗核抗体の陰性，陽性別の評価時期ごとの抗核抗体の陰性/陽性の分布を表 12.5－1 に示した．

投与開始日（0 週）で陰性であった被験者のうち，投与後いずれかの時点で陽性となった被験者は 9 名中 2 名であった．

12.5.2.2 抗 dsDNA 抗体

投与開始日（0 週）の抗 dsDNA 抗体（IgG 抗体，IgM 抗体）の陰性，陽性別の評価時期ごとの抗 dsDNA 抗体（IgG 抗体，IgM 抗体）の陰性/陽性の分布を表 12.5－1 に示した．

抗 dsDNA IgG 抗体について，投与開始日（0 週）に陰性であった被験者のうち，投与後いずれかの時点で陽性となった被験者は認められなかった．また，抗 dsDNA IgM 抗体について，投与開始日（0 週）に陰性であった被験者のうち，投与後いずれかの時点で陽性となった被

験者は 20 名中 11 名であった.

12.6 安全性の結論

治験薬を少なくとも 1 回投与され，かつ，治験薬投与開始後の安全性データが得られた 21 名を対象に安全性評価を行い，以下の結論を得た.

(1) 有害事象及び副作用の発現率は，それぞれ 95.2%（20/21 名）及び 71.4%（15/21 名）であった．発現率が高かった有害事象の器官別大分類については，「感染症および寄生虫症」及び「臨床検査」が共に 57.1%（12/21 名），「胃腸障害」52.4%（11/21 名）で，発現率が高かった有害事象は「二本鎖 DNA 抗体陽性」57.1%（12/21 名），「鼻咽頭炎」33.3%（7/21 名）であった．また，発現率が高かった副作用の器官別大分類は，「臨床検査」57.1%（12/21 名）で，発現率が高かった副作用は「二本鎖 DNA 抗体陽性」57.1%（12/21 名）であった.

(2) 重篤な有害事象及び重篤な副作用の発現率は，それぞれ 14.3%（3/21 名）及び 4.8%（1/21 名）であった．「潰瘍性大腸炎」（医師記載名「潰瘍性大腸炎の悪化」）が 2 名に認められたが，治験薬との因果関係は否定された．重篤な副作用としては「腸炎」が認められ，程度は中等度で，無治療で回復した.

(3) 中止に至った有害事象の発現率は，4.8%（1/21 名）で，「潰瘍性大腸炎」（医師記載名「潰瘍性大腸炎の悪化」）であった．中止に至った副作用は認められなかった.

(4) 感染症及び感染症（副作用）の発現率は，それぞれ 61.9%（13/21 名）及び 23.8%（5/21 名）であった．最も発現率が高かった感染症の事象は「鼻咽頭炎」33.3%（7/21 名）であった．重篤な感染症の発現率は 4.8%（1/21 名）で，「腸炎」のみであった．投与中止に至った感染症は認められなかった.

(5) Infusion reaction 及び Infusion reaction（副作用）の発現率は共に 9.5%（2/21 名）であった．事象の程度は中等度が 1 名と軽度が 1 名に認められたが，いずれの事象も発現日同日で回復に至った．また，重篤な Infusion reaction 及び投与中止に至った Infusion reaction は認められなかった.

(6) 免疫血清学的検査の有害事象の発現率は 57.1%（12/21 名）であり，「二本鎖 DNA 抗体(IgM)陽性」57.1%（12/21 名），「抗核抗体増加」4.8%（1/21 名）であった．ループス様症候群に相関するといわれている抗 dsDNA IgG 抗体が陽性化した被験者は認められなかった．また，ループス様症候群を発現した被験者も認められなかった.

(7) 本剤において留意すべき事象である悪性腫瘍（小児や若年成人で報告されている肝脾 T 細胞リンパ腫を含む），脱髄疾患，間質性肺炎，肝機能障害，遅発性過敏反応（血清病様反応を含む），うっ血性心不全，重篤な血液障害及び横紋筋融解症は認められなかった.

(8) 一般臨床検査の異常変動発現率が 10%以上であった臨床検査項目は 7 項目認められ，尿蛋白（増加），尿潜血（増加）が共に 47.6%（10/21 名），ALT（GPT）（減少）33.3%（7/21 名），白血球数（減少），ALP（減少），γ-GTP（減少），BUN（減少）がそれぞれ 14.3%（3/21 名）であった．臨床上問題となるような変動は認められなかった．

(9) 理学的検査（収縮期血圧，拡張期血圧，脈拍数，体温）の要約統計量の変化から特に問題となる傾向は認められなかった．また，理学的検査に関連した有害事象は「血圧低下」が 4.8%（1/21 名）で発現したが，治験薬との因果関係は否定された．

以上，中等症から重症の小児の潰瘍性大腸炎患者に対して，本剤を 22 週まで投与した際の 30 週間の結果から，忍容性はおおむね良好であることが確認された．また，成人の潰瘍性大腸炎を含む既承認疾患の安全性プロファイルと同様，感染症，Infusion reaction，免疫血清学的検査に関連する事象が認められた．また，本剤投与に際して懸念される事象の発現頻度や重篤度が大きく高まることはなく，新たに留意すべき事象はないと考えられた．

13. 考察と全般的結論

潰瘍性大腸炎は，主として大腸粘膜を侵し，しばしばびらんや潰瘍を形成する原因不明のびまん性非特異性炎症疾患で，患者数は増加の一途をたどっている．小児期発症例は少ないものの成人と同様に増加傾向にある．潰瘍性大腸炎の治療は，薬物治療が中心であるが，既存の薬物療法では効果不十分となる場合も多く，最終的に外科的手術の適応となる症例は少なくない．また，小児に特有な特徴として，成人と比較して病変の広範囲化，重症化が見られやすく，ステロイドの長期投与による成長障害やステロイド依存などがあり，問題となっている．こうした背景の中，本剤（インフリキシマブ）は潰瘍性大腸炎の治療薬として 2010 年 6 月に国内で承認され，ステロイド依存例やステロイド抵抗例の治療法として位置づけられている．しかしながら小児においては，小児の潰瘍性大腸炎患者を対象とした臨床試験が実施されていないこと，用法・用量に明示的に「小児」と記載されていないことから，本剤を必要とする小児の潰瘍性大腸炎患者への使用が躊躇される場合があり問題となっている．海外では，6～17 歳の小児を対象として第 III 相試験（試験番号 : C0168T72）が実施された．本剤を 5 mg/kg で 0，2，6 週に投与後，8 週間隔投与での本剤の有効性及び安全性が確認され，既に欧米で承認を得ている．そこで国内でも，小児の潰瘍性大腸炎患者を対象に，本剤 5 mg/kg を 0，2，6 週に投与し，以降 8 週間隔で 14，22 週に投与した際の有効性，安全性及び薬物動態を検討した．

本治験では，21 名の被験者に治験薬が投与された．CAI スコアは，投与 2 週から減少し，その後，投与 6，8 週においても経時的に減少が認められ，投与 10 週以降もその減少効果が認められた．また，パーシャル Mayo スコア及び PUCAI スコアでも，投与 8 週までの経時的なスコアの減少及び投与 10 週以降の減少効果が認められた．CAI スコア寛解については，投与 2 週で半数以上の被験者が寛解と判定され，この高い寛解率を投与 30 週まで維持することが確認された．PUCAI スコア寛解についても，その寛解率は CAI スコア寛解率ほど高くなかったが，投与 2 週で寛解と判定された被験者が認められ，投与 30 週まで維持する事が確認された．また，PUCAI スコアが 20 ポイント以上減少した場合は臨床的に意義があることが報告されていることから，PUCAI スコアが 20 ポイント以上減少した被験者の割合も評価した．その結果，投与 2 週において半数以上の被験者で PUCAI スコアの 20 ポイント以上減少が認められ，この効果を投与 30 週まで維持することが確認された．これらの結果より，本剤は投与 2 週という早期から症状を改善及び寛解に導く効果を示し，その効果は投与を継続することで維持されることが示唆された．

Mayo スコアの評価には S 状結腸鏡検査を必要とするが，S 状結腸鏡検査は被験者への侵襲性が大きいことから，本治験では可能な限り評価することとした．評価可能であった被験者は 8 名で，治験薬投与に至った被験者の半数に満たなかったが，投与 30 週で Mayo スコアの減少が確認された．また，Mayo スコア改善及び Mayo スコア寛解を示した被験者も確認された．更に，投与 30 週で粘膜治癒に至った被験者が 2 名確認されており，本剤は粘膜治癒効果を有することが示唆された．

ステロイドには，成人で報告されている副作用に加え，成長障害という小児特有の大きな問題がある．潰瘍性大腸炎・クローン病治療指針（平成 26 年度改訂版）の小児潰瘍性大腸炎治療指針では，成長障害の原因となるステロイドは寛解維持の目的には使用しないとされているが，ステロイドの減量に伴って増悪，再燃が起こるステロイド離脱困難例の存在が報告されている．本治験においても，登録時にステロイド離脱困難と判断された被験者は 76.2%（16/21 名）と半数以上であった．登録時にステロイド（経口剤）を使用していた被験者は 12 名で，これらの被験者のステロイドの使用量は，投与 2 週から減少が認められ，その後，投与 6，8 週においても経時的に減少が認められた．投与 10 週以降もその減少効果が確認された．投与 30 週でステロイドを離脱できた被験者は 2 名であったが，そのうち 1 名は CAI スコア寛解に該当した．以上の結果より，本剤を継続投与することにより，ステロイドの減量又は離脱が可能となることが示唆された．

CAI スコア応答例におけるトラフの血清中インフリキシマブ濃度では，血清中インフリキシマブ濃度を維持しており，CAI スコア応答例の投与終了 1 時間後の血清中インフリキシマブ濃度も大きく異ならないことから，本剤を複数回投与することによる薬剤の蓄積性はないと考えた．また，投与 30 週における血清中インフリキシマブ濃度別の CAI スコア変化量から，被験者数が少ない血清中インフリキシマブ濃度の集団があるものの，血清中インフリキシマブ濃度が高いほど有効性も高くなる傾向が認められた．

安全性について，有害事象及び副作用の発現率は，それぞれ 95.2%（20/21 名）及び 71.4%（15/21 名）であった．特に発現率の高かった器官別大分類は「感染症および寄生虫症」及び「臨床検査」であった．原疾患の悪化である「潰瘍性大腸炎」以外で確認された重篤な有害事象は「腸炎」のみであった．本事象は感染症と判断されたが，無治療で回復したこともあり治験中止には至らなかった．一方，中止に至った有害事象は「潰瘍性大腸炎」が 1 名で，中止に至った副作用は認められておらず，本剤の忍容性はおおむね良好であると考えられた．また，その他本剤投与において留意すべき事象である悪性腫瘍（小児や若年成人で報告されている肝脾 T 細胞リンパ腫を含む），脱髄疾患，間質性肺炎，肝機能障害，遅発性過敏反応（血清病様反応を含む），うっ血性心不全，重篤な血液障害及び横紋筋融解症は認められなかった．

以上の結果より，成人の潰瘍性大腸炎を含む既承認疾患で報告されている安全性プロファイルとの大きな相違は確認されず，本剤投与に際して懸念される事象の発現頻度や重篤度が大きく高まることもなく，新たに留意すべき事象はないと考えられた．

以上，小児の潰瘍性大腸炎の患者に本剤 5 mg/kg を 0，2，6 週に投与することで，潰瘍性大腸炎の症状改善効果が得られることが示された．更に，本剤を 14，22 週と 8 週間隔で継続投与することにより，その治療効果が持続することが示され，粘膜治癒効果が得られること及びステロイドを減量又は離脱できる可能性も示唆された．安全性について，本剤の忍容性はおおむね良好であり，本剤のこれまでに知られている安全性プロファイルと大きな相違はなく，新たに留意すべき事象はないと考えられた．

14. 本文中には含めないが，引用する表，図及びグラフ

14.1 人口統計学的データ

該当なし

14.2 有効性データ

該当なし

14.3 安全性データ

14.3.1 有害事象の表示

表 14.3－1 程度別の有害事象（免疫血清学的検査以外）

	(N=21)					
	軽度		中等度		高度	
	n	(%)	n	(%)	n	(%)
計	13	(61.9)	5	(23.8)	1	(4.8)
感染症および寄生虫症	11	(52.4)	1	(4.8)	0	(0.0)
気管支炎	0	(0.0)	1	(4.8)	0	(0.0)
鼻咽頭炎	7	(33.3)	0	(0.0)	0	(0.0)
上気道感染	2	(9.5)	0	(0.0)	0	(0.0)
感染性腸炎	2	(9.5)	0	(0.0)	0	(0.0)
胃腸炎	1	(4.8)	0	(0.0)	0	(0.0)
副鼻腔炎	1	(4.8)	0	(0.0)	0	(0.0)
皮下組織膿瘍	1	(4.8)	0	(0.0)	0	(0.0)
血液およびリンパ系障害	2	(9.5)	1	(4.8)	0	(0.0)
鉄欠乏性貧血	1	(4.8)	1	(4.8)	0	(0.0)
貧血	1	(4.8)	0	(0.0)	0	(0.0)
神経系障害	1	(4.8)	0	(0.0)	0	(0.0)
頭痛	1	(4.8)	0	(0.0)	0	(0.0)
眼障害	1	(4.8)	0	(0.0)	0	(0.0)
強膜炎	1	(4.8)	0	(0.0)	0	(0.0)
血管障害	1	(4.8)	0	(0.0)	0	(0.0)
血管炎	1	(4.8)	0	(0.0)	0	(0.0)
呼吸器、胸郭および縦隔障害	4	(19.0)	0	(0.0)	0	(0.0)
喘息	1	(4.8)	0	(0.0)	0	(0.0)
アレルギー性鼻炎	1	(4.8)	0	(0.0)	0	(0.0)
上気道の炎症	1	(4.8)	0	(0.0)	0	(0.0)
口腔咽頭痛	1	(4.8)	0	(0.0)	0	(0.0)
胃腸障害	6	(28.6)	4	(19.0)	1	(4.8)
潰瘍性大腸炎	0	(0.0)	1	(4.8)	1	(4.8)
悪心	1	(4.8)	1	(4.8)	0	(0.0)
腸炎	0	(0.0)	1	(4.8)	0	(0.0)
自己免疫性膵炎	0	(0.0)	1	(4.8)	0	(0.0)
嘔吐	2	(9.5)	0	(0.0)	0	(0.0)
下腹部痛	1	(4.8)	0	(0.0)	0	(0.0)
上腹部痛	1	(4.8)	0	(0.0)	0	(0.0)
口唇炎	1	(4.8)	0	(0.0)	0	(0.0)
便秘	1	(4.8)	0	(0.0)	0	(0.0)
膵炎	1	(4.8)	0	(0.0)	0	(0.0)
皮膚および皮下組織障害	2	(9.5)	0	(0.0)	0	(0.0)
ざ瘡	2	(9.5)	0	(0.0)	0	(0.0)
そう痒症	1	(4.8)	0	(0.0)	0	(0.0)
筋骨格系および結合組織障害	2	(9.5)	1	(4.8)	0	(0.0)
筋肉痛	0	(0.0)	1	(4.8)	0	(0.0)
関節痛	1	(4.8)	0	(0.0)	0	(0.0)
背部痛	1	(4.8)	0	(0.0)	0	(0.0)
一般・全身障害および投与部位の状態	2	(9.5)	0	(0.0)	0	(0.0)
胸部不快感	1	(4.8)	0	(0.0)	0	(0.0)
注入部位疼痛	1	(4.8)	0	(0.0)	0	(0.0)
臨床検査	2	(9.5)	0	(0.0)	0	(0.0)
血圧低下	1	(4.8)	0	(0.0)	0	(0.0)
尿中蛋白陽性	1	(4.8)	0	(0.0)	0	(0.0)
傷害、中毒および処置合併症	1	(4.8)	0	(0.0)	0	(0.0)
熱傷	1	(4.8)	0	(0.0)	0	(0.0)

表 14.3－2　程度別の副作用（免疫血清学的検査以外）

	(N=21)					
	軽度		中等度		高度	
	n	(%)	n	(%)	n	(%)
計	5	(23.8)	4	(19.0)	0	(0.0)
感染症および寄生虫症	3	(14.3)	1	(4.8)	0	(0.0)
気管支炎	0	(0.0)	1	(4.8)	0	(0.0)
鼻咽頭炎	2	(9.5)	0	(0.0)	0	(0.0)
皮下組織膿瘍	1	(4.8)	0	(0.0)	0	(0.0)
感染性腸炎	1	(4.8)	0	(0.0)	0	(0.0)
血液およびリンパ系障害	1	(4.8)	0	(0.0)	0	(0.0)
鉄欠乏性貧血	1	(4.8)	0	(0.0)	0	(0.0)
胃腸障害	0	(0.0)	3	(14.3)	0	(0.0)
腸炎	0	(0.0)	1	(4.8)	0	(0.0)
悪心	0	(0.0)	1	(4.8)	0	(0.0)
自己免疫性膵炎	0	(0.0)	1	(4.8)	0	(0.0)
皮膚および皮下組織障害	1	(4.8)	0	(0.0)	0	(0.0)
ざ瘡	1	(4.8)	0	(0.0)	0	(0.0)
筋骨格系および結合組織障害	1	(4.8)	1	(4.8)	0	(0.0)
筋肉痛	0	(0.0)	1	(4.8)	0	(0.0)
関節痛	1	(4.8)	0	(0.0)	0	(0.0)
一般・全身障害および投与部位の状態	1	(4.8)	0	(0.0)	0	(0.0)
注入部位疼痛	1	(4.8)	0	(0.0)	0	(0.0)

表 14.3－3　重篤な感染症

	(N=21)		
	n	(%)	件数
計	1	(4.8)	1
胃腸障害	1	(4.8)	1
腸炎	1	(4.8)	1

表 14.3－4　重篤な感染症（副作用）

	(N=21)		
	n	(%)	件数
計	1	(4.8)	1
胃腸障害	1	(4.8)	1
腸炎	1	(4.8)	1

14.3.2 死亡，その他の重篤な有害事象及び他の重要な有害事象の一覧表

該当なし

14.3.3 死亡，その他の重篤な有害事象及び他の特に重要な有害事象の叙述

表 14.3－5 重篤な有害事象の叙述

被験者識別コード	性<br>年齢*	一日投与量<br>投与日	症例報告書記載症状名	潰瘍性大腸炎の悪化	転帰<br>（転帰日）	医師コメント
			年月日	経過及び処置		
TA-650UC-011-01	男<br>13 歳	5 mg/kg<br>2012/12/25<br>2013/1/9<br>2013/2/6	2012/11	潰瘍性大腸炎再燃，近医にて入院，安静にして保守的治療.	回復<br>(2013/4/17)	今回の事象は原疾患である潰瘍性大腸炎の悪化と考えられ，治験薬との因果関係は否定できると判断する.
			2012/12/12	当院へ加療目的に入院.		
			2012/12/14	本治験へ同意.		
			2012/12/25	TA-650 5 mg/kg 投与開始．投与後特にトラブルなし.		
			2013/1/9	TA-650 5 mg/kg 2 週目投与．投与後特にトラブルなく退院.		
			2013/2/20	外来にて 8 週目検査実施．寛解状態.		
			2013/3/5	外来にて 10 週目検査実施．腹痛，排便回数の増加（1 日 5，6 回の下痢），血便があったことを訴えた．潰瘍性大腸炎再燃，内視鏡による評価が必要と判断.		
			2013/3/6	入院.		
			2013/3/8	内視鏡検査施行，潰瘍性大腸炎再燃を確認し，治験中止となる．夕方よりプレドニン注開始（20 mg/日）.		
			2013/3/9	プレドニン注 40mg/日へ．排便回数 2 回.		
			2013/3/10	アルタットカプセル 75mg/日開始.		
			2013/3/11	LCAP 1 回目実施，問題なく終了.		
			2013/3/12	ブレディニン錠 300mg/日開始.		
			2013/3/15	LCAP 2 回目実施，左肘部に疼痛と発赤の訴えがあったがすぐに消失．排便回数 1 回.		
			2013/3/18	LCAP 3 回目実施，左肘部に疼痛と発赤が認められたが帰室時には消失.		
			2013/3/19	プレドニン注 40 mg/日→プレドニゾロン錠 40 mg/日へ変更．イムラン錠 50 mg/日開始.		
			2013/3/22	LCAP 4 回目実施，左肘部に疼痛と発赤.		
			2013/3/25	LCAP 5 回目実施，疼痛及び発赤なし.		
			2013/3/28	LCAP 6 回目実施，疼痛及び発赤なし．排便回数は 0〜1 回．退院，外来フォローとなる．ブレディニン錠 300mg/日→400mg/日へ変更．プレドニゾロン錠 40 mg/日→30mg/日へ変更，4/6 より 20 mg/日の指示.		
			2013/4/17	外来受診時寛解状態を確認.		
＜治験薬との関連性＞否定できる						

*重篤な有害事象発現時の年齢

表 14.3－5　重篤な有害事象の叙述（続き）

被験者識別コード	性<br>年齢*	一日投与量<br>投与日	症例報告書記載症状名	潰瘍性大腸炎の悪化	転帰<br>（転帰日）	医師コメント
			年月日	経過及び処置		
TA-650UC-024-01	女<br>17 歳	5 mg/kg<br>2012/8/17<br>2012/8/31<br>2012/9/28	2012/8/1	本臨床試験参加同意.	回復<br>(2013/1/4)	本事例は 9 月 28 日に治験薬投与終了した不応例である.<br>今回の事象は原疾患である潰瘍性大腸炎の悪化と考えられ，治験薬との因果関係は否定できると判断する.
			2012/8/17	治験薬投与（2012/8/17～9/28）.		
			2012/10/15	投与 8 週後評価にて CAI スコア不応例となる.		
			2012/10/24	10 週評価来院．排便回数の増加，泥状便，発熱（38.0℃）あり．潰瘍性大腸炎の悪化と考えられ，プレドニゾロン内服増量となる.		
			2012/10/31	腹痛と出血のため来院．次週から 2 回/週の血球成分除去療法開始となる.		
			2012/11/6	血球成分除去療法施行．前日から 39.0℃台の発熱あり.<br>血球成分除去療法の効果を待つには症状悪化が目立つため，ステロイドパルス＋抗菌薬併用のため入院加療予定となる.		
			2012/11/8	入院．ステロイドパルス療法開始.		
			2012/11/13	症状あり．改善はしてきている.		
			2012/11/22	血球成分除去療法（LCAP）施行．溶血が出現したため同日で中止.		
			2012/11/27	GCAP を再開.		
			2012/11/28	その後症状の改善が見られないため，タクロリムス内服開始．大腸内視鏡検査実施.<br>びまん性の深掘れ潰瘍と pseudopolyposis を認める．易出血性あり.<br>手術を検討.		
			2012/12/5	血便持続，下痢頻回，夜間腹痛増強.		
			2012/12/6	内服薬すべて中止．経口での水分，食事摂取中止.		
			2012/12/10	手術のため，他院へ転院.		
			2012/12/14	手術（大腸亜全摘・回腸瘻造設術）施行.		
			2012/12/28	退院.		
			2013/1/4	当院を外来受診．本事象の回復を確認.		
＜治験薬との関連性＞否定できる						

*重篤な有害事象発現時の年齢

表 14.3−5　重篤な有害事象の叙述（続き）

被験者識別コード	性<br>年齢*	一日投与量<br>投与日	症例報告書記載症状名<br>年月日	腸炎<br>経過及び処置	転帰<br>（転帰日）	医師コメント
TA-650PS-010-02	男<br>14 歳	5 mg/kg<br>2013/11/25<br>2013/12/9<br>2014/1/6<br>2014/2/28<br>2014/4/28	2014/3/31<br>2014/4/1<br>2014/4/2<br>2014/4/3<br>2014/4/4<br><br><br>2014/4/6<br>2014/4/7<br>2014/4/8<br>2014/4/9<br>2014/4/14<br>2014/4/28<br>2014/5/21	治験薬投与後 18 週の来院．CRP 軽度上昇が認められたが，経過観察とした．<br>排便回数 10 回<br>38.8 度の発熱．カロナールで軽快．排便回数は 15 回．<br>39 度の発熱．18 時より嘔気出現し，5〜6 回嘔吐．排便回数は 30 回以上．<br>解熱したが，1 時間に数回の排便あり．嘔気はなし．外来受診．<br>腸炎の合併として，入院となった．<br>補液と水分摂取にて経過観察．<br>アデノウイルス（便・咽頭）陰性．CD 毒素陰性．便培養陰性．<br>CRP 低下，排便回数 12 回と減少し，症状改善傾向．血便なし．<br>排便回数 8 回．血便なし．<br>全身状態改善したため，退院とした．<br>排便回数 8 回．血便なし．状態は落ち着いているが，排便回数は多い．<br>排便回数 6 回．血便なし．下痢はあるが，有形便もあり．22 週目の治験薬投与．<br>排便回数 3〜4 回．血便，腹痛なし．下痢はあり．全身状態は落ち着いており，臨床検査値も改善しているため，有害事象の回復とした．	回復<br>(2014/5/21)	レミケードの維持療法中に，抗菌薬や潰瘍性大腸炎の治療薬の変更なしに軽快した，発熱と CRP の上昇を伴う一過性の腸炎を合併した．<br>経過より，アデノウイルス腸炎を最も疑ったが，便と咽頭の検査ではアデノウイルス感染の診断はつかなかった．<br>3 月 31 日に発症後，4 月 3 日に腸炎症状のピークを認め，4 月 4 日の受診日には，下痢の回数の減少と解熱傾向を認めていた．それでも，就眠中に排便のために覚醒する状況もあり，入院での管理とした．入院後には自然経過で軽快し 4 月 9 日に退院となっている．<br>経過より，なんらかの感染による腸炎が最も疑われる．抗菌薬なしで軽快した．<br>治験薬の投与がなくても起きえた事象とは思われるが，その発症への関与は否定できないことになる．<br>しかしながら，治験薬，免疫調整薬（アザチオプリン）の減量や中止を要しておらず，原疾患の治療としての治験薬の継続には問題がないと考えている．
＜治験薬との関連性＞否定できない						

*重篤な有害事象発現時の年齢

14.3.4 患者ごとの個々の臨床検査異常値の一覧表

表 14.3－6 臨床検査値の異常変動発現率

	異常増加			異常減少		
	n	増加	%	n	減少	%
赤血球数(×10^4/μL)	21	1	4.8	21	0	0.0
ヘモグロビン(g/dL)	21	0	0.0	21	0	0.0
ヘマトクリット(%)	21	0	0.0	21	0	0.0
白血球数(/μL)	21	2	9.5	21	3	14.3
血小板数(×10^4/μL)	20	1	5.0	20	1	5.0
AST(GOT)(U/L)	21	2	9.5	21	1	4.8
ALT(GPT)(U/L)	21	1	4.8	21	7	33.3
ALP(U/L)	21	0	0.0	21	3	14.3
LDH(U/L)	21	1	4.8	21	2	9.5
γ-GTP(U/L)	21	2	9.5	21	3	14.3
総蛋白(g/dL)	21	0	0.0	21	0	0.0
アルブミン(g/dL)	21	2	9.5	21	1	4.8
総コレステロール(mg/dL)	21	1	4.8	21	1	4.8
総ビリルビン(mg/dL)	21	1	4.8	21	2	9.5
BUN(mg/dL)	21	2	9.5	21	3	14.3
血清クレアチニン(mg/dL)	21	0	0.0	21	1	4.8
Na(mEq/L)	21	0	0.0	21	0	0.0
K(mEq/L)	21	0	0.0	21	0	0.0
Cl(mEq/L)	21	0	0.0	21	0	0.0
尿蛋白(定性)	21	10	47.6	21	0	0.0
尿糖(定性)	21	0	0.0	21	0	0.0
尿潜血(定性)	21	10	47.6	21	0	0.0
尿ウロビリノーゲン(定性)	21	0	0.0	21	0	0.0

表 14.3－7　血液学的検査，血液生化学的検査の要約統計量

		n	Median	Q1	Q3	Min	Max
赤血球数(×10^4/μL)	0週	21	414.0	389.0	463.0	275	510
	2週	20	435.5	403.0	461.0	278	509
	6週	20	450.0	385.5	486.5	258	534
	8週	19	444.0	385.0	494.0	263	509
	10週	19	466.0	407.0	506.0	268	557
	14週	20	466.5	394.5	495.0	266	553
	18週	16	465.5	425.5	496.5	323	550
	22週	16	473.0	418.5	485.5	307	553
	26週	14	480.0	437.0	502.0	312	511
	30週	14	473.5	455.0	513.0	289	543
ヘモグロビン(g/dL)	0週	21	11.80	10.80	12.60	9.0	14.0
	2週	20	12.05	10.60	12.35	9.4	14.1
	6週	20	11.85	11.25	13.10	8.8	13.6
	8週	19	12.10	10.70	13.00	8.2	14.4
	10週	19	12.50	11.20	13.50	9.2	15.3
	14週	20	11.95	11.20	13.60	8.2	14.8
	18週	16	12.15	11.35	13.40	9.5	15.4
	22週	16	12.25	11.05	13.85	9.1	15.2
	26週	14	12.30	11.40	14.40	10.0	14.8
	30週	14	12.20	11.40	14.80	10.2	15.9
ヘマトクリット(%)	0週	21	35.10	32.60	38.00	28.9	41.6
	2週	20	36.45	33.00	38.50	29.1	41.8
	6週	20	36.90	33.60	39.10	28.2	42.3
	8週	19	37.60	31.50	39.20	25.4	43.6
	10週	19	38.30	36.10	40.10	27.8	45.7
	14週	20	36.80	34.40	39.95	24.5	44.8
	18週	16	37.60	34.55	40.15	29.6	46.2
	22週	16	37.35	34.75	41.00	29.1	45.1
	26週	14	37.60	35.80	41.90	31.8	43.6
	30週	14	37.35	34.80	43.30	30.0	47.4
白血球数(/μL)	0週	21	8600.0	6400.0	10500.0	4200	17000
	2週	20	6750.0	5850.0	7700.0	4300	14300
	6週	20	7150.0	5850.0	7850.0	4800	9300
	8週	19	6800.0	4700.0	8200.0	3400	9900
	10週	19	7300.0	5300.0	8400.0	3800	10600
	14週	20	7250.0	6100.0	8900.0	3400	13900
	18週	16	5900.0	4950.0	6950.0	3600	8200
	22週	16	6500.0	5100.0	8700.0	4000	12000
	26週	14	5800.0	5100.0	7200.0	3600	11800
	30週	14	6700.0	5400.0	8100.0	4200	18100

表 14.3−7　血液学的検査，血液生化学的検査の要約統計量（続き）

		n	Median	Q1	Q3	Min	Max
好中球(%)	0週	21	69.00	57.40	80.00	35.0	85.0
	2週	20	60.65	49.15	69.45	23.9	87.0
	6週	20	53.05	42.00	64.00	29.5	87.5
	8週	19	49.60	33.40	63.00	21.6	80.5
	10週	19	54.50	40.50	66.50	27.1	84.0
	14週	20	57.35	52.30	70.25	32.2	86.5
	18週	16	51.05	44.35	58.30	29.0	69.5
	22週	16	58.40	51.90	63.55	32.3	81.5
	26週	14	48.90	42.00	63.90	35.6	67.6
	30週	14	56.45	52.60	62.80	41.0	78.5
好酸球(%)	0週	21	3.60	1.00	6.00	0.0	18.5
	2週	20	3.50	1.60	4.75	0.0	14.0
	6週	20	3.50	1.00	7.35	0.3	22.0
	8週	19	3.60	2.00	6.00	0.9	29.5
	10週	19	3.00	1.00	7.70	0.3	13.3
	14週	20	2.40	1.00	5.60	0.0	17.5
	18週	16	3.40	1.80	5.05	1.0	19.5
	22週	16	3.45	2.15	4.85	0.5	9.5
	26週	14	3.00	1.80	4.90	0.1	11.0
	30週	14	3.95	2.00	6.00	0.0	13.5
好塩基球(%)	0週	21	0.00	0.00	0.40	0.0	3.0
	2週	20	0.50	0.30	0.70	0.0	1.0
	6週	20	0.35	0.00	0.75	0.0	3.0
	8週	19	0.50	0.40	0.90	0.0	1.5
	10週	19	0.40	0.30	0.50	0.0	1.2
	14週	20	0.50	0.35	0.70	0.0	1.2
	18週	16	0.60	0.35	0.85	0.0	1.5
	22週	16	0.50	0.20	0.65	0.0	1.0
	26週	14	0.50	0.30	0.70	0.0	1.0
	30週	14	0.05	0.00	0.50	0.0	0.7
単球(%)	0週	21	6.50	5.00	8.50	4.0	13.5
	2週	20	6.50	4.50	7.30	3.0	12.5
	6週	20	7.85	5.35	9.00	3.8	13.0
	8週	19	7.00	6.00	10.50	4.0	24.0
	10週	19	8.30	6.10	9.50	5.0	15.0
	14週	20	6.50	5.20	9.05	4.4	12.1
	18週	16	9.25	5.95	10.25	4.1	12.5
	22週	16	7.10	5.35	8.10	3.5	10.0
	26週	14	7.70	5.50	11.70	4.8	14.0
	30週	14	5.75	4.10	9.50	3.0	13.0

表 14.3−7　血液学的検査，血液生化学的検査の要約統計量（続き）

		n	Median	Q1	Q3	Min	Max
リンパ球(%)	0週	21	18.00	14.00	24.80	5.5	49.6
	2週	20	25.55	20.25	34.35	8.5	64.0
	6週	20	29.75	22.70	44.60	7.5	64.0
	8週	19	34.40	24.60	46.30	7.5	68.0
	10週	19	31.90	20.10	42.70	5.5	59.0
	14週	20	28.10	20.75	37.50	7.5	59.6
	18週	16	36.50	31.15	40.40	17.6	57.0
	22週	16	29.55	24.50	36.95	14.5	54.3
	26週	14	39.40	26.50	44.70	17.5	54.2
	30週	14	31.50	28.10	38.80	16.3	44.2
血小板数(×10^4/µL)	0週	20	36.40	30.40	42.40	18.6	57.6
	2週	19	32.00	26.20	39.90	18.6	56.0
	6週	19	32.20	24.80	37.80	19.1	48.9
	8週	18	28.70	26.10	34.30	15.5	49.2
	10週	19	31.20	24.80	36.50	14.3	54.5
	14週	19	27.30	23.20	39.10	14.6	43.3
	18週	15	29.60	22.90	37.80	15.3	40.4
	22週	15	29.60	26.60	35.90	16.3	45.9
	26週	13	27.70	26.40	31.60	19.8	42.8
	30週	13	27.20	24.60	32.00	16.2	44.3
AST(GOT)(U/L)	0週	21	17.0	16.0	20.0	12	47
	2週	20	19.0	16.0	22.5	12	30
	6週	20	19.0	17.5	21.5	11	26
	8週	19	20.0	17.0	22.0	11	26
	10週	20	19.5	16.5	21.5	11	35
	14週	20	19.0	16.0	21.0	11	25
	18週	16	19.5	18.0	22.5	11	49
	22週	16	19.5	16.5	21.0	12	32
	26週	14	19.5	17.0	23.0	13	30
	30週	14	19.0	16.0	21.0	11	31
ALT(GPT)(U/L)	0週	21	10.0	8.0	12.0	7	49
	2週	20	12.0	9.0	16.5	7	35
	6週	20	10.0	8.5	13.5	6	21
	8週	19	10.0	8.0	13.0	6	18
	10週	20	9.5	7.0	12.5	5	18
	14週	20	10.5	9.5	14.5	5	23
	18週	16	10.0	8.0	13.0	5	28
	22週	16	9.0	7.0	13.0	6	50
	26週	14	9.5	8.0	12.0	7	32
	30週	14	9.0	8.0	12.0	6	29

表 14.3−7　血液学的検査，血液生化学的検査の要約統計量（続き）

		n	Median	Q1	Q3	Min	Max
ALP(U/L)	0週	21	382.0	244.0	472.0	115	785
	2週	20	394.5	238.5	460.5	110	990
	6週	20	426.5	329.0	566.0	115	1143
	8週	19	451.0	278.0	560.0	111	1095
	10週	20	470.5	310.5	597.5	108	1057
	14週	20	407.5	267.5	657.5	185	823
	18週	16	545.0	344.0	744.5	200	948
	22週	16	542.5	291.0	708.0	205	910
	26週	14	511.5	300.0	699.0	208	980
	30週	14	496.0	245.0	611.0	208	833
LDH(U/L)	0週	21	179.0	153.0	247.0	126	436
	2週	20	187.0	146.0	211.0	137	494
	6週	20	184.0	159.5	227.5	143	453
	8週	19	192.0	152.0	238.0	133	494
	10週	20	195.5	162.5	248.5	119	455
	14週	20	181.0	151.5	266.5	132	460
	18週	16	199.5	167.0	249.0	128	435
	22週	16	185.0	171.0	235.0	136	375
	26週	14	188.5	164.0	240.0	146	391
	30週	14	186.0	163.0	278.0	126	383
γ-GTP(U/L)	0週	21	13.0	9.0	18.0	5	42
	2週	20	15.5	11.5	18.5	7	28
	6週	20	11.5	9.5	14.5	7	21
	8週	19	12.0	8.0	15.0	6	20
	10週	20	10.5	8.5	14.5	4	17
	14週	20	11.5	8.5	16.5	5	92
	18週	16	10.5	8.5	15.0	6	19
	22週	16	10.5	8.0	12.5	6	59
	26週	14	11.0	7.0	13.0	6	25
	30週	14	11.0	8.0	13.0	6	25
総蛋白(g/dL)	0週	21	7.10	6.80	7.40	6.5	8.4
	2週	20	7.35	7.00	7.75	6.8	8.2
	6週	20	7.30	7.10	7.65	6.7	8.4
	8週	19	7.40	7.00	7.90	6.3	8.2
	10週	20	7.45	7.15	7.85	6.4	8.7
	14週	20	7.25	6.80	7.75	6.6	8.3
	18週	16	7.40	7.05	7.70	6.5	8.5
	22週	16	7.10	6.85	7.80	5.8	8.6
	26週	14	7.50	7.30	7.90	6.6	8.6
	30週	14	7.30	7.20	7.90	7.0	8.5

表 14.3−7　血液学的検査，血液生化学的検査の要約統計量（続き）

		n	Median	Q1	Q3	Min	Max
アルブミン(g/dL)	0週	21	4.00	3.80	4.40	3.4	4.8
	2週	20	4.35	4.20	4.55	3.8	4.9
	6週	20	4.25	4.20	4.45	3.9	4.8
	8週	19	4.40	4.10	4.50	3.8	4.9
	10週	20	4.30	4.15	4.60	3.8	5.0
	14週	20	4.25	4.00	4.40	3.5	4.9
	18週	16	4.30	4.10	4.50	3.9	5.2
	22週	16	4.20	4.05	4.35	3.1	5.0
	26週	14	4.40	4.20	4.60	3.8	5.1
	30週	14	4.30	4.10	4.40	4.0	5.0
総コレステロール(mg/dL)	0週	21	143.0	120.0	170.0	89	220
	2週	20	167.0	146.0	186.0	98	266
	6週	20	155.0	127.0	169.5	98	229
	8週	19	147.0	126.0	162.0	90	201
	10週	20	157.0	131.5	181.0	102	240
	14週	20	152.5	137.0	175.5	97	232
	18週	16	148.0	125.0	165.5	95	232
	22週	16	158.0	130.0	168.0	93	197
	26週	14	149.5	142.0	162.0	114	212
	30週	14	154.5	140.0	178.0	104	192
総ビリルビン(mg/dL)	0週	21	0.40	0.30	0.50	0.2	0.9
	2週	20	0.50	0.40	0.65	0.2	1.7
	6週	20	0.50	0.35	0.70	0.3	1.0
	8週	19	0.50	0.40	0.70	0.2	1.3
	10週	20	0.50	0.40	0.75	0.2	1.2
	14週	20	0.50	0.30	0.60	0.2	1.3
	18週	16	0.40	0.35	0.80	0.3	1.0
	22週	16	0.50	0.35	0.75	0.3	1.4
	26週	14	0.55	0.30	0.80	0.3	3.0
	30週	14	0.60	0.40	0.70	0.3	1.2
BUN(mg/dL)	0週	21	9.0	7.0	11.0	5	17
	2週	20	12.5	9.0	14.5	6	24
	6週	20	11.0	10.0	12.5	5	16
	8週	19	11.0	9.0	14.0	6	16
	10週	20	11.5	10.0	13.0	7	16
	14週	20	11.5	9.5	13.5	8	24
	18週	16	11.0	9.0	13.0	6	16
	22週	16	11.0	9.5	13.5	7	16
	26週	14	11.5	9.0	13.0	7	16
	30週	14	13.0	10.0	14.0	6	14

表 14.3−7　血液学的検査，血液生化学的検査の要約統計量（続き）

		n	Median	Q1	Q3	Min	Max
血清クレアチニン(mg/dL)	0週	21	0.490	0.450	0.560	0.34	0.72
	2週	20	0.500	0.460	0.570	0.34	0.70
	6週	20	0.495	0.420	0.595	0.32	0.77
	8週	19	0.560	0.430	0.590	0.38	0.83
	10週	20	0.515	0.460	0.595	0.33	0.76
	14週	20	0.485	0.455	0.520	0.34	0.63
	18週	16	0.540	0.475	0.585	0.31	0.71
	22週	16	0.510	0.415	0.590	0.36	0.72
	26週	14	0.565	0.390	0.610	0.31	0.69
	30週	14	0.520	0.400	0.590	0.35	0.72
Na(mEq/L)	0週	21	139.0	138.0	140.0	134	142
	2週	20	139.0	138.0	140.0	136	143
	6週	20	140.0	139.0	141.0	137	143
	8週	19	140.0	139.0	141.0	138	143
	10週	20	139.5	139.0	140.5	136	143
	14週	20	139.0	138.0	140.0	136	143
	18週	16	140.0	139.0	141.0	137	143
	22週	16	140.5	138.5	141.0	136	142
	26週	14	140.0	138.0	142.0	137	143
	30週	14	140.0	139.0	141.0	136	141
K(mEq/L)	0週	21	3.80	3.70	4.10	3.1	4.6
	2週	20	3.90	3.70	4.00	3.2	4.3
	6週	20	3.80	3.60	4.05	3.5	4.5
	8週	19	4.00	3.60	4.10	3.1	4.4
	10週	20	4.00	3.80	4.15	3.4	4.4
	14週	20	3.95	3.80	4.25	3.2	4.4
	18週	16	4.10	3.85	4.20	3.2	4.9
	22週	16	3.85	3.70	4.10	3.4	4.5
	26週	14	3.90	3.60	4.20	3.3	4.6
	30週	14	4.10	3.70	4.30	3.5	4.7
Cl(mEq/L)	0週	21	101.0	100.0	103.0	93	105
	2週	20	102.0	100.5	102.5	98	104
	6週	20	103.0	101.0	104.0	98	105
	8週	19	103.0	102.0	105.0	97	107
	10週	20	102.5	101.0	104.0	98	107
	14週	20	103.0	100.5	104.0	98	108
	18週	16	103.0	102.0	104.5	98	106
	22週	16	104.0	101.0	105.0	98	107
	26週	14	102.5	102.0	104.0	99	105
	30週	14	103.0	101.0	104.0	98	105

表 14.3－8　尿検査（定性）の判定の度数分布

正：正常，異：異常

		8週		14週		22週		30週	
		正	異	正	異	正	異	正	異
尿蛋白(定性)	0週正	10	4	13	2	12		7	3
	0週異	4	1	4	1	2	2	2	2
尿糖(定性)	0週正	19		20		16		14	
	0週異								
尿潜血(定性)	0週正	16	3	19	1	16		13	1
	0週異								

低：低値，正：正常，高：高値

		8週			14週			22週			30週		
		低	正	高	低	正	高	低	正	高	低	正	高
尿ウロビリノーゲン(定性)	0週低												
	0週正		19			20			16			14	
	0週高												

表 14.3－9　理学的検査の要約統計量

				n	Mean	SD	Min	Max
収縮期血圧（mmHg）	0週	投与前	投与前	21	106.3	13.0	88	132
		投与中	30分後	21	102.0	11.4	79	122
			60分後	21	103.0	11.7	81	129
			90分後	21	107.8	11.7	90	133
			120分後	21	104.6	10.4	89	125
		投与後	30分後	21	107.4	11.2	88	128
			60分後	21	107.7	13.0	89	144
			90分後	21	105.6	12.9	89	145
			120分後	21	106.5	12.8	85	142
	2週	投与前	投与前	20	103.5	8.0	88	122
		投与中	30分後	20	105.3	9.7	88	127
			60分後	20	105.1	12.6	86	126
			90分後	20	105.9	12.0	89	130
			120分後	20	105.2	10.7	89	131
		投与後	30分後	20	106.6	13.1	86	135
			60分後	20	107.9	15.1	85	133
			90分後	20	109.5	13.9	88	136
			120分後	20	111.3	15.5	86	145
	6週	投与前	投与前	20	102.2	11.2	80	126
		投与中	30分後	20	101.0	10.6	86	124
			60分後	20	101.8	11.7	82	132
			90分後	20	100.9	11.5	83	125
			120分後	20	102.7	12.3	82	128
		投与後	30分後	20	103.9	14.3	81	137
			60分後	20	102.9	13.3	75	128
			90分後	20	104.5	14.4	81	140
			120分後	20	105.5	13.9	81	129
	14週	投与前	投与前	16	101.2	12.3	84	124
		投与中	30分後	16	102.0	11.1	80	124
			60分後	16	99.6	15.3	76	134
			90分後	16	101.2	10.5	86	122
			120分後	16	103.5	12.4	87	124
		投与後	30分後	16	101.4	7.0	92	115
			60分後	16	105.2	11.5	92	131
			90分後	16	103.8	15.1	70	125
			120分後	16	109.7	10.1	98	130
	22週	投与前	投与前	14	106.0	12.3	86	125
		投与中	30分後	14	102.4	12.8	86	128
			60分後	13	104.3	11.7	87	127
			90分後	13	101.5	13.3	83	128
			120分後	14	106.4	12.8	88	131
		投与後	30分後	14	103.6	15.1	82	137
			60分後	14	106.5	12.5	88	132
			90分後	14	107.8	14.4	94	140
			120分後	14	109.8	14.2	90	139

表 14.3−9　理学的検査の要約統計量（続き）

				n	Mean	SD	Min	Max
拡張期血圧（mmHg）	0週	投与前	投与前	21	60.7	10.3	42	84
		投与中	30分後	21	58.5	8.9	44	81
			60分後	21	57.0	10.6	33	77
			90分後	21	60.0	9.5	40	77
			120分後	21	60.0	8.5	40	75
		投与後	30分後	21	62.4	11.2	42	89
			60分後	21	60.9	9.0	46	78
			90分後	21	58.5	9.9	42	87
			120分後	21	56.6	10.1	40	76
	2週	投与前	投与前	20	56.8	9.0	40	72
		投与中	30分後	20	62.4	8.6	45	80
			60分後	20	58.4	9.9	38	77
			90分後	20	61.0	12.1	41	82
			120分後	20	60.2	7.3	41	71
		投与後	30分後	20	59.6	8.7	44	74
			60分後	20	58.9	10.5	40	83
			90分後	20	63.1	10.0	40	80
			120分後	20	60.0	9.6	39	73
	6週	投与前	投与前	20	56.3	7.9	40	74
		投与中	30分後	20	54.3	9.8	37	74
			60分後	20	55.2	7.8	36	68
			90分後	20	56.1	11.5	42	84
			120分後	20	56.1	10.9	34	70
		投与後	30分後	20	56.8	7.7	45	69
			60分後	20	57.5	7.9	44	70
			90分後	20	57.6	8.4	37	74
			120分後	20	59.4	7.7	44	75
	14週	投与前	投与前	16	57.4	8.4	40	72
		投与中	30分後	16	53.2	7.1	38	69
			60分後	16	55.4	16.4	36	106
			90分後	16	54.5	6.9	40	65
			120分後	16	56.0	10.0	42	79
		投与後	30分後	16	56.9	11.9	44	81
			60分後	16	58.0	9.1	44	75
			90分後	16	57.3	9.8	40	76
			120分後	16	59.6	6.8	48	69
	22週	投与前	投与前	14	57.6	9.3	41	75
		投与中	30分後	14	55.4	8.5	43	70
			60分後	13	59.2	9.2	43	73
			90分後	13	53.9	10.0	30	73
			120分後	14	54.1	9.2	38	77
		投与後	30分後	14	59.2	10.5	39	81
			60分後	14	57.5	8.3	46	74
			90分後	14	60.5	5.8	52	72
			120分後	14	60.2	9.8	49	77

表 14.3－9　理学的検査の要約統計量（続き）

				n	Mean	SD	Min	Max
脈拍数（拍/分）	0週	投与前	投与前	21	86.6	16.3	59	125
		投与中	30分後	21	82.3	19.2	53	133
			60分後	21	85.8	18.9	59	136
			90分後	21	85.0	19.0	59	136
			120分後	21	88.3	17.3	62	139
		投与後	30分後	21	85.7	13.9	62	109
			60分後	21	87.3	13.6	65	109
			90分後	21	86.9	12.6	66	107
			120分後	21	87.1	13.4	67	115
	2週	投与前	投与前	20	78.7	14.6	61	115
		投与中	30分後	20	78.7	13.0	60	111
			60分後	20	80.2	16.5	57	109
			90分後	20	79.3	15.9	54	113
			120分後	20	78.2	13.3	54	105
		投与後	30分後	20	82.5	11.6	63	101
			60分後	20	84.5	9.6	68	102
			90分後	20	87.2	10.6	65	106
			120分後	20	85.9	9.6	67	101
	6週	投与前	投与前	20	83.7	13.6	60	109
		投与中	30分後	20	80.6	14.5	60	103
			60分後	20	80.7	15.5	56	102
			90分後	20	80.2	17.2	52	115
			120分後	20	83.2	15.3	60	111
		投与後	30分後	20	83.0	14.7	63	109
			60分後	20	82.7	13.2	65	115
			90分後	20	81.3	12.5	64	108
			120分後	20	82.7	13.3	62	112
	14週	投与前	投与前	16	78.6	12.7	55	101
		投与中	30分後	16	76.2	12.5	54	92
			60分後	16	76.8	12.0	54	100
			90分後	16	77.0	13.3	56	98
			120分後	16	75.6	12.9	56	101
		投与後	30分後	16	79.6	12.7	60	100
			60分後	16	78.8	10.7	62	93
			90分後	16	79.3	13.8	54	102
			120分後	16	81.6	10.8	64	104
	22週	投与前	投与前	14	80.5	14.9	51	108
		投与中	30分後	14	78.9	13.4	59	108
			60分後	13	78.4	13.6	61	109
			90分後	13	78.8	11.9	63	102
			120分後	14	78.1	13.6	59	107
		投与後	30分後	14	80.6	11.7	63	104
			60分後	14	81.2	12.2	64	98
			90分後	14	81.3	10.3	61	98
			120分後	14	83.6	12.4	63	107

表 14.3－9　理学的検査の要約統計量（続き）

				n	Mean	SD	Min	Max
体温（℃）	0週	投与前	投与前	21	36.55	0.64	35.7	38.1
		投与中	30分後	21	36.75	0.55	35.7	38.2
			60分後	21	36.98	0.75	36.2	39.5
			90分後	21	37.06	0.85	36.1	40.1
			120分後	21	37.06	0.79	35.8	39.3
		投与後	30分後	21	36.76	0.52	36.0	38.3
			60分後	21	36.83	0.54	35.8	38.3
			90分後	21	36.66	0.49	35.8	37.8
			120分後	21	36.70	0.55	35.2	37.9
	2週	投与前	投与前	20	36.57	0.49	35.5	37.2
		投与中	30分後	20	36.63	0.47	35.7	37.3
			60分後	20	36.58	0.42	35.5	37.2
			90分後	20	36.64	0.44	35.6	37.4
			120分後	20	36.73	0.42	35.7	37.4
		投与後	30分後	20	36.73	0.32	36.0	37.1
			60分後	20	36.80	0.26	36.2	37.2
			90分後	20	36.74	0.35	35.7	37.2
			120分後	20	36.65	0.40	35.9	37.2
	6週	投与前	投与前	20	36.61	0.38	35.6	37.3
		投与中	30分後	20	36.73	0.33	35.7	37.1
			60分後	20	36.80	0.22	36.5	37.3
			90分後	20	36.85	0.32	35.9	37.4
			120分後	20	36.72	0.40	35.6	37.4
		投与後	30分後	20	36.72	0.30	36.2	37.2
			60分後	20	36.75	0.31	36.0	37.3
			90分後	20	36.73	0.32	36.1	37.2
			120分後	20	36.72	0.40	35.8	37.3
	14週	投与前	投与前	16	36.45	0.37	35.8	37.2
		投与中	30分後	16	36.60	0.28	35.9	37.0
			60分後	16	36.73	0.30	36.2	37.2
			90分後	16	36.62	0.32	35.9	37.1
			120分後	16	36.64	0.36	35.6	37.1
		投与後	30分後	16	36.61	0.38	35.6	37.1
			60分後	16	36.56	0.47	35.7	37.2
			90分後	16	36.67	0.34	35.8	37.1
			120分後	16	36.57	0.37	35.9	37.1
	22週	投与前	投与前	14	36.57	0.44	35.6	37.4
		投与中	30分後	13	36.59	0.53	35.7	37.3
			60分後	12	36.67	0.59	35.1	37.4
			90分後	12	36.61	0.52	35.8	37.2
			120分後	13	36.65	0.34	36.1	37.2
		投与後	30分後	13	36.81	0.46	36.0	37.6
			60分後	13	36.72	0.54	35.4	37.4
			90分後	13	36.80	0.56	35.2	37.6
			120分後	13	36.75	0.42	35.6	37.2

15. 引用文献の一覧表

1) Turner D, Seow CH, Greenberg GR, Griffiths AM, Silverberg MS, Steinhart AH. A systematic prospective comparison of noninvasive disease activity indices in ulcerative colitis. Clin Gastroenterol Hepatol. 2009;7(10):1081-8. Epub 2009 Jul 1.
2) 田中敏章, 編集. 新しい小児の臨床検査基準値ポケットガイド. 東京: じほう; 2009.
3) Turner D, Otley AR, Mack D, Hyams J, de Bruijne J, Uusoue K, et al. Development, validation, and evaluation of a pediatric ulcerative colitis activity index: a prospective multicenter study. Gastroenterology. 2007;133(2):423-32.

16. 付録

16.1 治験に関する情報

16.1.1 治験実施計画書及びその改訂

16.1.1a 治験実施計画書（第 02.00.00000 版，作成年月日：2013 年 5 月 22 日）
16.1.1b 治験実施計画書 変更対比表

16.1.2 症例記録用紙の見本

16.1.2 症例報告書（第 01.00.00000 版，作成年月日：2011 年 12 月 20 日）

16.1.3 治験審査委員会の一覧，患者への説明文書及び同意文書の見本

16.1.3a 治験審査委員会の一覧（確認が行われた年月日，並びに委員の氏名及び職名）
16.1.3b 説明文書・同意文書（会社案）の最終版
（版番号：06.00.00000，作成年月日：2014 年 8 月 6 日）
16.1.3c 説明文書・同意文書（会社案）の変更箇所一覧
16.1.3d 説明文書・同意文書（施設版）の一覧
16.1.3e 説明文書・アセント文書（13 歳以上，会社案）の最終版
（版番号：05.00.00000，作成年月日：2014 年 8 月 6 日）
16.1.3f 説明文書・アセント文書（13 歳以上，会社案）の最終版の変更箇所一覧
16.1.3g 説明文書・アセント文書（13 歳以上，施設版）の一覧
16.1.3h 説明文書・アセント文書（12 歳以下，会社案）の最終版
（版番号：01.00.00000，作成年月日：2011 年 12 月 20 日）
16.1.3i 説明文書・アセント文書（12 歳以下，施設版）の一覧

16.1.4 治験責任医師及び他の重要な治験参加者の一覧表及び説明

16.1.5 治験依頼者の医学責任者の署名

16.1.6 各被験者に投与された薬剤のロット番号一覧表

16.1.7 無作為化の方法及びコード（患者の識別及び割付けられた治療）

該当なし

16.1.8 監査手順に関する資料，監査証明書

16.1.9 統計手法に関する文書

16.1.9a 統計解析計画書（第 3.0 版）
16.1.9b 統計解析報告書（第 1.0 版）
16.1.9c 薬物動態解析計画書（第 1.0 版）
16.1.9d 薬物動態解析報告書（第 1.0 版）

16.1.10 臨床検査に関して施設間の標準化及び品質保証の方法と手順に関する文書

該当なし

16.1.11 治験に基づく公表文献

該当なし

16.1.12 総括報告書で引用された重要な公表文献

該当なし

16.1.13 その他の付録

16.1.13a 治験薬の管理に関する手順書（第 6 版）
16.1.13b 安全性評価委員業務手順書（第 1 版）
16.1.13c 内視鏡写真アトラス（別紙 5）
16.1.13d 試験計画書
（血清中インフリキシマブ濃度及び抗インフリキシマブ抗体（ATI）の測定）
16.1.13e 最終報告書
（血清中インフリキシマブ濃度及び抗インフリキシマブ抗体（ATI）の測定）
16.1.13f モニタリング手順書
16.1.13g 症例検討会議事録
16.1.13h 独立行政法人医薬品医療機器総合機構 対面助言議事録

16.1.14 薬物動態に関する文書 （該当する場合）

16.2 被験者データ一覧表

16.2.1 中止被験者

16.2.2 治験実施計画から逸脱した被験者

16.2.3 有効性の解析から除外された被験者

16.1.13g 参照

16.2.4 人口統計学的データ

16.2.4a 被験者背景一覧表 1
16.2.4b 被験者背景一覧表 2
16.2.4c 被験者背景一覧表 3

16.2.5 服薬遵守及び（又は）薬物濃度データ

16.2.8f, 16.1.13e 参照

16.2.6 個々の有効性反応データ

16.2.6a 被験者ごとの有効性一覧表 1 （CAI スコア）（TF）（FAS）
16.2.6b 被験者ごとの有効性一覧表 1 （CAI サブスコア）（LOCF）（FAS）
16.2.6c 被験者ごとの有効性一覧表 2（パーシャル Mayo スコア/Mayo スコア）（TF）（FAS）
16.2.6d 被験者ごとの有効性一覧表 2 （パーシャル Mayo サブスコア/Mayo サブスコア）（LOCF）（FAS）
16.2.6e 被験者ごとの有効性一覧表 3（PUCAI スコア）（TF）（FAS）
16.2.6f 被験者ごとの有効性一覧表 3（PUCAI サブスコア）（LOCF）（FAS）
16.2.6g 被験者ごとの有効性一覧表 4（ステロイド使用量）（FAS）

16.2.7 被験者ごとの有害事象一覧表

16.2.7a 被験者ごとの有害事象一覧（安全性解析対象集団）
16.2.7b 被験者ごとの重篤な有害事象一覧（安全性解析対象集団）

16.2.8 被験者ごとの臨床検査値一覧表

16.2.8a 被験者ごとの血液学的検査一覧表（安全性解析対象集団）
16.2.8b 被験者ごとの血液生化学的検査一覧表 1（安全性解析対象集団）
16.2.8c 被験者ごとの血液生化学的検査一覧表 2（安全性解析対象集団）
16.2.8d 被験者ごとの尿検査（定性）一覧表（安全性解析対象集団）
16.2.8e 被験者ごとの免疫血清学的検査一覧表（安全性解析対象集団）
16.2.8f 被験者ごとの理学的検査（血圧，脈拍数，体温）一覧表（安全性解析対象集団）
16.2.8g 被験者ごとのその他の臨床検査項目（CRP）（FAS）
16.2.8h 被験者ごとの身長及び体重一覧表（FAS）

16.3 症例記録

16.3.1 死亡，その他の重篤な有害事象を発現した被験者及び有害事象による投与中止した被験者の症例記録

該当なし

16.3.2 提出された他の症例記録

該当なし
