# Supplementary material for: Infliximab for pediatric patients with Crohn’s disease: A Phase 3, open-label, uncontrolled, multicenter trial in Japan
Source: PLoS One. 2018 Aug 16;13(8):e0201956. doi: 10.1371/journal.pone.0201956 (PMC6095542; doi:10.1371/journal.pone.0201956)
Supplement: S2 File — (PDF) [file pone.0201956.s002.pdf]

Clinical Study Report

Clinical Study of TA-650 in Pediatric Patients with Ulcerative Colitis

Sponsor
Mitsubishi Tanabe Pharma Corporation

Protocol No.: TA-650-21
Version Number.: 2.0

1. Title Page

Study title	Clinical Study of TA-650 in Pediatric Patients with Ulcerative Colitis
Name of the study drug	TA-650
Indications studied	Ulcerative colitis
Study design<br>Comparator<br>Study duration<br><br><br><br><br><br><br><br>Dosage regimen<br><br><br>Study patient population	Open label, uncontrolled multi-center study<br>Not applicable<br>Screening period: Period from the starting day of the clinical activity index (CAI) score evaluation to the start of the study treatment.<br>Evaluation period: Period from the start of the study treatment to the evaluation day at Week 30; for CAI score-based non-responders and discontinued patients, period from the start of the study treatment to the end of the evaluation at 8 weeks after the last treatment.<br>TA-650 is administered at a dose of 5 mg/kg based on the body weight on each day of administration by intravenous drip infusion over at least a 2-hr period.<br>Pediatric patients with moderate to severe ulcerative colitis
Study sponsor	Mitsubishi Tanabe Pharma Corporation
Protocol No.	TA-650-21
Phase of development	Phase III
Date of study initiation	April 25, 2012 (date when the first patient gave informed consent)
Date of early study termination	Not applicable.
Date of study completion	September 25, 2014 (date when the last patient completed the specified evaluation [excluding the follow-up])
Name/affiliation of the sponsor's medical expert	• Toshifumi Hibi, Director of Center for Advanced IBD Research and Treatment, Kitasato University Kitasato Institute Hospital, The Kitasato Institute.<br>• Kazuoki Kondo, Consultant of Mitsubishi Tanabe Pharma Corporation
Sponsor's representative/ person in charge	Clinical Research Department III, Sohyaku. Innovative Research Division, Mitsubishi Tanabe Pharma Corporation<br><br>Manager Hiroshi Yamada
Compliance with GCP	The study was conducted in compliance with the Ordinance on Good Clinical Practice (GCP), and related laws and regulations.<br>Study-related documents are retained at the study site or by the sponsor according to the GCP.
Date of the report	February 4, 2016

2. Synopsis

Study sponsor:<br>Mitsubishi Tanabe Pharma Corporation	Individual Summary Table<br><br>Referring to Part of the Dossier<br><br>Volume:<br><br>Page:	(For Regulatory Authority Use only)
Trade name:<br>REMICADE® for IV Infusion 100		
Name of Active Ingredient:<br>Infliximab<br>(genetically-modified)		

Study title: Clinical Study of TA-650 in Pediatric Patients with Ulcerative Colitis	
Study centers and investigators: See Appendix 16.1.4 (34 sites and 38 investigators in total)	
Publication: Not applicable	
Study duration: Approximately 2 years and 5 months<br>(Date when the first patient gave informed consent) April 25, 2012<br>(Date when the last patient completed the specified evaluation [excluding the follow-up]) September 25, 2014	Phase of development:<br>Phase III
Objectives: To assess the efficacy of TA-650 until Week 30 in pediatric patients with moderate to severe ulcerative colitis when TA-650 is administered at a dose of 5 mg/kg at Weeks 0, 2 and 6, followed by administration at 8-week intervals at Weeks 14 and 22, based on the evaluation index such as CAI score. Also to assess the safety and pharmacokinetics of TA-650.	
Methodology: Open label, uncontrolled multi-center study	
Number of patients (Planned and analyzed):<br>Planned: Target number of patients: 20 patients, as the number of patients who received the study drug<br>Analyzed: 21 patients registered; 21 patients in the efficacy analysis set, 21 patients in the safety analysis set; 21 patients in the pharmacokinetic analysis set	

Study sponsor: Mitsubishi Tanabe Pharma Corporation	Individual Summary Table<br><br>Referring to Part of the Dossier<br><br>Volume:<br><br>Page:	(For Regulatory Authority Use only)
Trade name: REMICADE® for IV Infusion 100		
Name of Active Ingredient: Infliximab (genetically-modified)		

Diagnosis and primary criteria for inclusion:

Patients who satisfied all of the following inclusion criteria (including both inpatients and outpatients).

(1) Target disease: ulcerative colitis.

Patients who were diagnosed as having ulcerative colitis according to the revised draft of the Diagnostic Criteria for Ulcerative Colitis established by the Ministry of Health, Labour and Welfare (MHLW) Study Group for Specific Diseases/Intractable Inflammatory Bowel Disease (revised on February 13, 2010), and had been suffering for at least 3 months at the time of obtainment of informed consent.

(2) Patients aged not less than 6 years and not more than 17 years old (at the time of obtaining consent).

(3) Patients whose legal representative can give consent in writing and patients who can give themselves give oral or written assent for participation in this study.

(4) Patients with a CAI score of not less than 7 and a score for blood in stools of not less than 2 at the time of registration

(5) Patients who had at least one of the treatment experiences listed in the following 1) to 4).

1) 6-mercaptopurine or azathioprine: Patients who had been using the drug for at least 12 weeks before the starting day of screening period, and receiving the drug at a stable dose for at least 4 weeks before the starting day of screening period.

2) Steroids (oral preparation): Patients using steroids at a stable dose of at least 1 mg/kg/day or at least 20 mg/day calculated on a prednisolone basis for at least 2 weeks before the starting day of screening period.

3) Patients who had either of the following experiences within 5 years before the starting day of screening period.

- Use of 6-mercaptopurine or azathioprine for at least 12 weeks, but having an inadequate response.
- Use of 6-mercaptopurine or azathioprine, and having the experience of an adverse drug reaction that made it difficult to continue the treatment.

4) Patients who had at least one of the following experiences within 18 months before the starting day of screening period.

- Experience of an exacerbation or relapse in response to a reduction in the steroid dose, and failure of steroid withdrawal.
- Use of steroid but having an inadequate response (using steroids at a dose of at least 1 mg/kg/day or at least 40 mg/day calculated on a prednisolone basis for at least 2 weeks

Study sponsor:<br>Mitsubishi Tanabe Pharma Corporation	Individual Summary Table<br><br>Referring to Part of the Dossier<br><br>Volume:<br><br>Page:	(For Regulatory Authority Use only)
Trade name:<br>REMICADE® for IV Infusion 100		
Name of Active Ingredient:<br>Infliximab<br>(genetically-modified)		

orally or for at least 1 week by intravenous injection but no therapeutic effects were obtained).

- Use of steroids and experiencing an adverse drug reaction that made it difficult to continue treatment.

(6) Patients satisfying the following criteria for drug therapy used on the starting day of the screening period.

1) 6-mercaptopurine or azathioprine: Patients who had been using the drug for at least 12 weeks before the starting day of screening period, and receiving the drug at a stable dose for at least 4 weeks before the starting day of screening period, or had not used the drug for at least 4 weeks before the starting day of the screening period.

2) Steroids (oral preparations): Patients who had been using the drug at a stable dose for at least 2 weeks before the starting day of the screening period, or had not used the drug for at least 2 weeks before the starting day of screening period.

3) 5-aminosalicylate agents (oral preparations) or salazosulfapyridine agents (oral preparations): Patients who had been using the drug at a stable dose for at least 2 weeks before the starting day of the screening period, or had not used the drug for at least 2 weeks before the starting day of the screening period.

Study drug, dose and mode of administration, lot number:

- Study drug: TA-650 (infliximab [genetically-modified])
- Dose and mode of administration:
  TA-650 was administered at a dose of 5 mg/kg based on the body weight on each day of administration by intravenous drip infusion over at least a 2-hr period on the starting day of the study drug administration (Week 0) and at Weeks 2 and 6. Patients who had a clinical response measured by CAI score at Week 8 were further given the study drug at Weeks 14 and 22 in a similar manner. However, patients who had no response measured by the CAI score would not be given the study drug after Week 14.
- Lot No.:110001, 120075

Control drug, dose and mode of administration, lot number: Not applicable

Duration of treatment:

Screening period: Period from the starting day of the CAI score evaluation to the start of the study treatment.

Evaluation period: Period from the start of the study treatment to the evaluation day at Week 30; for CAI score-based non-responders and discontinued patients, period from the start of the study treatment to the end of the evaluation at 8 weeks after the last treatment.

Study sponsor:<br>Mitsubishi Tanabe Pharma Corporation	Individual Summary Table<br><br>Referring to Part of the Dossier<br><br>Volume:<br><br>Page:	(For Regulatory Authority Use only)
Trade name:<br>REMICADE® for IV Infusion 100		
Name of Active Ingredient:<br>Infliximab<br>(genetically-modified)		

Criteria for evaluation

Efficacy:

(1) CAI score

(2) CAI score-based remission

(3) Partial Mayo score

(4) Mayo score

(5) Mayo score-based response

(6) Mayo score-based remission

(7) Mucosal healing

(8) PUCAI score

(9) PUCAI score-based remission

(10) PUCAI score decrease by at least 20 points

(11) Steroid dose

(12) Steroid withdrawal

Safety:

(1) Adverse events (AEs) and adverse drug reactions (ADRs)

- Adverse events
- Adverse drug reactions

Pharmacokinetics:

(1) Serum infliximab concentrations (serum TA-650 concentrations)

(2) Antibodies to infliximab (antibodies to TA-650, hereinafter referred to as ATI)

Study sponsor: Mitsubishi Tanabe Pharma Corporation	Individual Summary Table<br><br>Referring to Part of the Dossier<br><br>Volume:<br><br>Page:	(For Regulatory Authority Use only)
Trade name: REMICADE® for IV Infusion 100		
Name of Active Ingredient: Infliximab (genetically-modified)		

Statistical methods:

(1) Efficacy

Efficacy analysis set was the full analysis set (hereinafter referred to as FAS). Analysis of each endpoint other than steroid dose was performed in the same manner in the descriptions shown below.

Both of the results for which an imputation of missing data was not performed (data at each evaluation point include those at the “last” time point) and those for which an imputation (treatment failure, hereinafter referred to as TF) was applied were shown (except for summary statistics of each subscore).

1) CAI score

(a) Endpoints

- CAI score and CAI score-based remission rate

(b) Analysis method

- Summary statistics of CAI score and CAI score change, and CAI score-based remission rate at each evaluation point were calculated.

2) Partial Mayo score

(a) Endpoint

- Partial Mayo score

(b) Analysis method

- Summary statistics of partial Mayo score and partial Mayo score change at each evaluation point were calculated.

3) Mayo score

(a) Endpoints

- Mayo score, Mayo score-based response rate, Mayo score-based remission rate, and rate of mucosal healing

(b) Analysis method

- Summary statistics of Mayo score and Mayo score change, and Mayo score-based response rate, Mayo score-based remission rate and rate of mucosal healing at each evaluation point were calculated.

4) PUCAI score

(a) Endpoints

- PUCAI score, PUCAI score-based remission rate, and proportion of patients who achieved a PUCAI score decrease by at least 20 points

(b) Analysis method

Study sponsor: Mitsubishi Tanabe Pharma Corporation	Individual Summary Table<br><br>Referring to Part of the Dossier<br><br>Volume:<br><br>Page:	(For Regulatory Authority Use only)
Trade name: REMICADE® for IV Infusion 100		
Name of Active Ingredient: Infliximab (genetically-modified)		

- Summary statistics of PUCAI score and PUCAI score change, PUCAI score-based remission rates, and the proportion of patients who achieved a PUCAI score decrease by at least 20 points at each evaluation point were calculated.

5) Steroid dose

(a) Endpoints

- Steroid dose and rate of steroid withdrawal

(b) Analysis method

- Among FAS, those patients who had been using steroids (oral preparation) at the time of registration were included in the analysis set.
- Both of the results for which an imputation of missing data was not performed (data at each evaluation point include those at the "last" time point) and those for which an imputation (TF) was applied were shown.
- Summary statistics of steroid dose and rate of steroid dose change, and rate of steroid withdrawal at each evaluation point were calculated.

(2) Safety

Patients in the safety analysis set were analyzed.

The incidence rates of AEs and ADRs during the evaluation period were calculated. In addition, the incidence rates of AEs by timing, infusion reactions by the number of doses, infusion reactions per one dose of the study drug, infusion reactions by ATI, and AEs by severity were calculated. Furthermore, the incidence rates of abnormal changes for the general laboratory tests, cross tabulation of each determination result for the immunoserological tests, and summary statistics for the physical examination were tabulated.

(3) Evaluation of pharmacokinetics

1) Analysis method

- Summary statistics of serum infliximab concentrations at each evaluation time point were listed.
- The frequency distribution and proportions of ATI determination (negative, positive or inconclusive) were listed. In addition, the summary statistics of serum infliximab concentration by ATI determination at each evaluation time point were listed.

Study sponsor:<br>Mitsubishi Tanabe Pharma Corporation	Individual Summary Table<br><br>Referring to Part of the Dossier<br><br>Volume:<br><br>Page:	(For Regulatory Authority Use only)
Trade name:<br>REMICADE® for IV Infusion 100		
Name of Active Ingredient:<br>Infliximab<br>(genetically-modified)		

Summary- Conclusions

Efficacy results:

(1) The CAI score (Mean ± SD) was 9.7 ± 2.7 at the time of registration, but decreased over time to 4.0 ± 3.2, 3.5 ± 3.5 and 3.2 ± 3.5 respectively at Weeks 2, 6 and 8. The CAI score ranged from 2.5 ± 2.3 to 3.5 ± 2.2 during the period from Week 10 to Week 30, and that at the last time point was 5.6 ± 3.8. The CAI score changes (Mean ± SD) were -5.5 ± 2.7, -6.0 ± 4.0 and -6.2 ± 3.7 at Weeks 2, 6 and 8, indicating an increase in the degree of score change with time, and ranged from -5.9 ± 3.8 to -6.9 ± 3.8 during the period from Week 10 to Week 30, and that at the last time point was -4.1 ± 4.2. In terms of both CAI score and CAI score change, the effect of the study drug in improving symptoms was observed from Week 2, and the effect lasted up to Week 30. Data on both CAI score and CAI score change obtained by TF imputation showed a similar tendency to data obtained before TF imputation.

(2) The CAI score-based remission rate was 60.0% (12/20) at Week 2, and increased to 80.0% (16/20) at both Weeks 6 and 8, and ranged from 64.3% to 87.5% during the period from Week 10 to Week 30. The CAI score-based remission rate at the last time point was 42.9% (9/21). In terms of CAI score-based remission rate, the study drug was observed to have the effect of bringing the clinical condition of patients to remission from Week 2, and this effect was confirmed to last up to Week 30. Data obtained by TF imputation showed a similar tendency to data obtained before TF imputation.

(3) The partial Mayo score (Mean ± SD) was 5.6 ± 1.6 at the time of registration, and decreased over time to respectively 2.6 ± 2.3, 2.2 ± 2.0 and 1.7 ± 1.7 at Weeks 2, 6 and 8, and ranged from 1.8 ± 1.7 to 2.8 ± 1.9 during the period from Week 10 to Week 30. The partial Mayo score at the last time point was 3.7 ± 2.2. The partial Mayo score changes (Mean ± SD) at Weeks 2, 6 and 8 were respectively -2.9 ± 2.2, -3.3 ± 2.2 and -3.8 ± 1.7, indicating an increase in the degree of score change with time. The partial Mayo changes ranged from -2.6 ± 2.8 to -3.6 ± 2.5 during the period from Week 10 to Week 30, and the change at the last time point was -1.9 ± 2.7. In terms of partial Mayo score and partial Mayo score change, the effect of the study drug in improving symptoms was observed from Week 2, and the effect lasted up to Week 30. Data on partial Mayo score and partial Mayo score change obtained by TF imputation showed a similar tendency to data obtained before TF imputation.

(4) The Mayo score (Mean ± SD) was 7.0 ± 2.4 at the time of registration, but decreased to 4.9 ± 2.4 at Week 30. The Mayo score at the last time point was 5.0 ± 2.3.The Mayo score changes (Mean ± SD) at Week 30 and the last time point were respectively -2.6 ± 3.9 and -2.0 ± 3.9. Data on both Mayo score and Mayo score change obtained by TF imputation showed a similar tendency to data obtained before TF imputation.

(5) The Mayo score-based response rate and Mayo score-based remission rate at Week 30 were respectively 42.9% (3/7) and 14.3% (1/7), and at the last point were respectively 37.5% (3/8) and 12.5% (1/8). Both Mayo score-based response rate and Mayo score-based remission rate calculated based on data obtained by TF imputation were similar to those obtained before imputation.

Study sponsor: Mitsubishi Tanabe Pharma Corporation	Individual Summary Table<br><br>Referring to Part of the Dossier<br><br>Volume:<br><br>Page:	(For Regulatory Authority Use only)
Trade name: REMICADE® for IV Infusion 100		
Name of Active Ingredient: Infliximab (genetically-modified)		

(6) The PUCAI score (Mean ± SD) was 47.1 ± 15.2 at the time of registration and decreased over time to 20.3 ± 16.3, 17.3 ± 17.5 and 12.5 ± 13.5 respectively at Weeks 2, 6 and 8. The score ranged from 12.2 ± 12.0 to 19.3 ± 18.8 during the period from Week 10 to Week 30, and was 28.8 ± 22.5 at the last time point. The PUCAI score changes (Mean ± SD) were -25.8 ± 17.5, -28.8 ± 21.1 and -33.5 ± 14.0 at Weeks 2, 6 and 8 respectively, and ranged from -26.8 ± 27.8 to -34.1 ± 17.7 during the period from Week 10 to Week 30. The score at the last time point was -18.3 ± 28.4. In terms of both PUCAI score and PUCAI score change, the effect of the study drug in improving symptoms was observed from Week 2, and lasted up to Week 30. Data on PUCAI score and PUCAI score change obtained by TF imputation showed a similar tendency to data obtained before TF imputation.

(7) The PUCAI score-based remission rate was 35.0% (7/20) at Week 2, and increased to 40.0% (8/20) at both Weeks 6 and 8. Afterward, the rate ranged from 28.6% to 42.9% during the period from Week 10 to Week 30, and was 19.0% (4/21) at the last time point. It was observed that the drug had the effect of leading the clinical condition of patients to remission from Week 2, and it was confirmed that the effect lasted. Data obtained by TF imputation showed a similar tendency to data obtained before TF imputation.

(8) The proportion of patients who achieved a PUCAI score decrease by at least 20 points was 68.4% (13/19) at Week 2, and increased to 73.7% (14/19) and 89.5% (17/19) at Weeks 6 and 8, respectively. Afterward, the proportion ranged from 64.3% to 88.2% during the period from Week 10 to Week 30, and was 60.0% (12/20) at the last time point. The effect of the study drug to improve the score was observed from Week 2, and it was confirmed that the effect lasted thereafter. Data obtained by TF imputation showed a similar tendency to data obtained before TF imputation.

(9) The rate of mucosal healing at Week 30 and the last time point were 33.3% (2/6) and 28.6% (2/7), respectively. Data obtained by TF imputation showed a similar tendency to data obtained before TF imputation.

(10) In 12 patients who had been using steroids (oral preparations) at the time of registration, the median steroid dose was 0.20 mg/kg/day at the time of registration, but decreased to 0.19, 0.17 and 0.16 mg/kg/day respectively at Weeks 2, 6 and 8. The median steroid dose ranged from 0.04 mg/kg/day to 0.15 mg/kg/day during the period from Week 10 to Week 30, and was 0.05 mg/kg/day at the last time point. The median rates of steroid dose change were -1.63%, -25.05% and -43.91% respectively at Weeks 2, 6 and 8, and the degree of change increased over time. The median rate of steroid dose change ranged from -61.72% to -86.93% during the period from Week 10 to Week 30, and was -85.44% at the last time point. Data on both steroid dose and rate of steroid dose change obtained by TF imputation showed a similar tendency to data obtained before TF imputation, suggesting that the study drug may possibly decrease the steroid dose.

(11) The rate of steroid withdrawal was 8.3% at Week 2, and increased over time to 25.0% at both

Study sponsor: Mitsubishi Tanabe Pharma Corporation	Individual Summary Table<br><br>Referring to Part of the Dossier<br><br>Volume:<br><br>Page:	(For Regulatory Authority Use only)
Trade name: REMICADE® for IV Infusion 100		
Name of Active Ingredient: Infliximab (genetically-modified)		

Weeks 6 and 8. The rate of steroid withdrawal ranged from 25.0% to 37.5% during the period from Week 10 to Week 30, and was 41.7% at the last time point. Data obtained by TF imputation showed a similar tendency to data obtained before TF imputation. Two patients achieved steroid withdrawal at Week 30, and one of these patients also achieved CAI score-based remission.

(12) The patients included in the efficacy analysis set were divided into two groups, one for those aged not less than 6 years and less than 12 years, and another for those aged not less than 12 years and not more than 17 years, and efficacy of the study drug was evaluated for each group. As a result, both of the two groups included a different number of patients, but the efficacy of the study drug between the two groups was not considered significantly different.

(13) The median trough serum infliximab concentrations in CAI score-based responders were respectively 2.58, 1.54 and 1.34 μg/mL, at Weeks 14, 22 and 30 thus indicating that the serum infliximab concentration was maintained.

(14) The median CAI score changes at Week 30 in each of the 4 groups by serum infliximab concentration, i.e. less than 0.1 μg/mL, not less than 0.1 and less than 1 μg/mL, not less than 1 and less than 10 μg/mL, and not less than 10 μg/mL, were respectively -4.5 and NC (impossible to calculate), -6.0, and NC. The CAI score changes in individual patients included in the group which exhibited NC were -4 and -3 in the group of not less than 0.1 and less than 1 μg/mL , and -10 in the group of not less than 10 μg/mL. Based on these results, though some of the serum infliximab concentration groups included a small number of patients, it was observed that a higher serum infliximab concentration tended to show better efficacy.

(15) Throughout the study period, the results for ATI determination were inconclusive in 81.0% (7/21) and negative in 19.0% (4/21) in the overall population, and none of the patients exhibited positive results.

Based on the above results, it was shown that TA-650 given to pediatric patients with moderate to severe ulcerative colitis at a dose of 5 mg/kg at Weeks 0, 2 and 6 was able to improve the clinical symptoms of ulcerative colitis and achieve mucosal healing. Continuous administration at 8-week intervals at Weeks 14 and 22 was also confirmed to maintain the effect of the drug. In addition, it was suggested that administration of TA-650 might enable decreased steroid dose or steroid withdrawal.

Safety results:

Safety evaluation was performed on 21 patients who received the study drug at least once and whose safety data after the start of administration of the study drug was obtained. Based on that evaluation, the following conclusions were obtained.

(1) The incidence rates of AEs and ADRs were respectively 95.2% (20/21) and 71.4% (15/21). The system organ classes (SOCs) of AEs having the highest incidence rate were “infections and infestations” and “investigations,” each having an incidence rate of 57.1% (12/21), and “gastrointestinal disorders” having an incidence rate of 52.4% (11/21). The AEs with a high incidence rate were “double stranded DNA antibody positive” having an incidence rate of

Study sponsor:<br>Mitsubishi Tanabe Pharma Corporation	Individual Summary Table<br><br>Referring to Part of the Dossier<br><br>Volume:<br><br>Page:	(For Regulatory Authority Use only)
Trade name:<br>REMICADE® for IV Infusion 100		
Name of Active Ingredient:<br>Infliximab<br>(genetically-modified)		

57.1% (12/21), and “nasopharyngitis” having an incidence rate of 33.3% (7/21). The SOCs of ADRs having a high incidence rate were “investigations” having an incidence rate of 57.1% (12/21), and the ADR having a high incidence rate was “double stranded DNA antibody positive” having an incidence rate of 57.1% (12/21).

(2) The incidence rates of serious AEs and serious ADRs were 14.3% (3/21) and 4.8% (1/21), respectively. “Colitis ulcerative” (recorded as “worsened ulcerative colitis” by the investigator) occurred in 2 patients, but a causal relationship of this event with the study drug was ruled out. Since serious ADR, “enterocolitis” was observed. This event was moderate and recovery occurred without any treatment.

(3) The incidence rate of AEs leading to discontinuation of the study was 4.8% (1/21), and the event was “colitis ulcerative” (recorded as “worsened ulcerative colitis” by the investigator). No ADRs leading to discontinuation of the study were observed.

(4) The incidence rates of infections and infections (ADRs) were 61.9% (13/21) and 23.8% (5/21), respectively. The event having the highest incidence rate was “nasopharyngitis” having an incidence rate of 33.3% (7/21). The incidence rate of serious infections was 4.8% (1/21), and the only one event was “enterocolitis.” No infections leading to discontinuation of treatment were observed.

(5) The incidence rates of infusion reactions and infusion reactions (ADRs) were both 9.5% (2/21). The events were moderate and mild, each observed in one patient, but in both of the events recovered occurred on the same day that the reactions occurred. No serious infusion reactions and infusion reactions leading to discontinuation of treatment were observed.

(6) The incidence rate of immunoserological test-related AEs was 57.1% (12/21), and the incidence rates of “double stranded DNA (IgM) antibody positive” and “antinuclear antibody increased” were respectively 57.1% (12/21) and 4.8% (1/21). No patients were observed to have experienced a change from negative to positive to double stranded DNA IgG antibody, which is said to be correlated with lupus-like syndrome. Furthermore, lupus-like syndrome did not occur in any patients.

(7) No events to be noted related to the study drug, such as malignancy (including hepatosplenic T-cell lymphoma which has been reported in children and young adults), demyelinating disorders, interstitial pneumonia, liver dysfunction, delayed hypersensitivity (including serum sickness-like reaction), congestive cardiac failure, serious blood disorder and rhabdomyolysis, were observed.

(8) There were 7 laboratory parameters where the incidence rate of abnormal change in general laboratory values was not less than 10%, including urine protein (increase) and urinary occult blood (increase) each having an incidence rate of 47.6% (10/21), ALT (GPT) (decrease) having an incidence rate of 33.3% (7/21), white blood cell count (decrease), ALP (decrease),

Study sponsor: Mitsubishi Tanabe Pharma Corporation	Individual Summary Table<br><br>Referring to Part of the Dossier<br><br>Volume:<br><br>Page:	(For Regulatory Authority Use only)
Trade name: REMICADE® for IV Infusion 100		
Name of Active Ingredient: Infliximab (genetically-modified)		

γ-GTP (decrease) and BUN (decrease) each having an incidence rate of 14.3% (3/21). No changes in laboratory parameters which were clinically significant were observed.

(9) The changes in summary statistics of physical examinations (systolic and diastolic blood pressure, pulse rate, and body temperature) did not show any problematic tendencies. As an AE related to physical examinations, "blood pressure decreased" occurred in 4.8% (1/21) of the patients, but a causal relationship of this event with the study drug was ruled out.

Based on the results of safety evaluation obtained for 30 weeks in this study where TA-650 was administered to pediatric patients with moderate to severe ulcerative colitis up to Week 22, it was confirmed that this study drug was mostly tolerable. In addition, similarly to the safety profiles for the approved indications including ulcerative colitis in adults, some events related to infections, infusion reactions, and immunoserological tests were observed. The frequency and severity of the events of concern related to the study drug administration had not increased significantly, and it was considered that no new events to be noted were observed.

Conclusions:

In this study, it was observed that TA-650 had the effect of improving clinical conditions and achieving mucosal healing when administered to pediatric patients with moderate to severe ulcerative colitis at a dose of 5 mg/kg at Weeks 0, 2 and 6 and at 8-week intervals thereafter. It was also suggested that the treatment with the study drug in the above-mentioned manner might allow for a decreased steroid dose or steroid withdrawal while maintaining the effect. In terms of safety of the drug, though many infections, infusion reactions and events related to immunoserological tests were observed similarly to the safety profile observed in adult patients with ulcerative colitis, the frequency of occurrence and severity of the events of concern related to the study drug administration were not significantly increased. Based on these results, TA-650 was considered effective in pediatric patients with ulcerative colitis.

3. Table of Contents

1. Title Page 2
2. Synopsis 3
3. Table of Contents 14
4. List of Abbreviations and Definitions of Terms 18
5. Ethics 22
5.1 Institutional Review Board (IRB) 22
5.2 Ethical Conduct of the Study 22
5.3 Patient Information and Consent 22
6. Investigators and Study Administrative Structure 25
6.1 Sponsor 25
6.1.1 Sponsor 25
6.1.2 Person in Charge at the Sponsor 25
6.1.3 Medical Experts 26
6.1.4 Clinical Leader 27
6.1.5 Person Responsible for Monitoring 27
6.1.6 Monitors 28
6.1.7 Person in Charge of Audit 28
6.1.8 Statistical Analysis Manager 29
6.1.9 Data Control Manager 30
6.1.10 Person Responsible for Clinical Pharmacological Analysis 31
6.1.11 Person Responsible for Pharmacokinetics and Other Measurement Items 32
6.1.12 Contract Research Organization 32
6.2 Coordinating Investigator 34
6.3 Safety Evaluation Committee 34
6.4 Study Centers and Investigators 34
7. Introduction 35
8. Study Objective 37
9. Investigational Plan 38
9.1 Overall Study Design and Plan–Description 38
9.1.1 Treatment Method Studied 38
9.1.2 Patient Population Studied and Planned Number of Patients to be Included 38
9.1.3 Level and Method of Blinding 38
9.1.4 Type of Control and Study Configuration 38
9.1.5 Method of Assignment to Treatment 38
9.1.6 Sequence and Duration of Study Period 38
9.1.7 Identification of When Patients were Randomized 40
9.1.8 Various Committees Established and Their Roles 40
9.1.9 Interim Analyses 40
9.2 Discussion of Study Design, Including the Choice of Control Groups 40
9.3 Selection of Study Population 41
9.3.1 Inclusion Criteria 41
9.3.2 Exclusion Criteria 42
9.3.3 Removal of Patients from Therapy or Assessment 47
9.4 Treatments 50

9.4.1 Treatments ... 50
9.4.2 Identity of Study Drug ... 51
9.4.3 Method of Assigning Patients to Treatment Groups ... 52
9.4.4 Selection of Doses in the Study ... 52
9.4.5 Selection and Timing of Dose for Each Patient ... 53
9.4.6 Blinding ... 53
9.4.7 Prior and Concomitant Therapy ... 53
9.4.8 Treatment Compliance ... 58
9.5 Efficacy and Safety Variables ... 59
9.5.1 Efficacy and Safety Endpoints and Flow Chart ... 59
9.5.2 Appropriateness of Measurements ... 82
9.5.3 Drug Concentration Measurements ... 82
9.6 Data Quality Assurance ... 83
9.7 Statistical Methods Planned in the Protocol and Determination of Sample Size ... 84
9.7.1 Statistical and Analytical Plans ... 84
9.7.2 Efficacy Evaluation ... 91
9.7.3 Safety Evaluation ... 92
9.7.4 Other Evaluations ... 95
9.7.5 Evaluation of Pharmacokinetics ... 95
9.7.6 Determination of Sample Size ... 96
9.8 Changes in the Conduct of the Study or Planned Analyses ... 97
9.8.1 Revision of the Protocol ... 97
9.8.2 Revision of Analysis Plans ... 97
10. Study Patients ... 97
10.1 Disposition of Patients ... 97
10.2 Protocol Deviations ... 99
11. Efficacy Evaluation ... 100
11.1 Data Sets Analyzed ... 100
11.2 Demographic and Other Baseline Characteristics ... 100
11.3 Measurements of Treatment Compliance ... 105
11.4 Efficacy Results and Tabulations of Individual Patient Data ... 106
11.4.1 Analysis of Efficacy ... 106
11.4.2 Statistical/Analytical Issues ... 126
11.4.3 Tabulation of Individual Response Data ... 128
11.4.4 Drug Dose, Drug Concentration, and Relationships to Response ... 128
11.4.5 Drug-Drug and Drug-Disease Interactions ... 132
11.4.6 By-Patient Display ... 132
11.4.7 Efficacy Conclusions ... 132
12. Safety Evaluation ... 136
12.1 Extent of Exposure ... 136
12.2 Adverse Events (AEs) ... 136
12.2.1 Brief Summary of Adverse Events ... 136
12.2.2 Display of Adverse Events ... 137
12.2.3 Analysis of Adverse Events ... 139
12.2.4 Listing of Adverse Events by Patient ... 141
12.3 Deaths, Other Serious Adverse Events, and Other Significant Adverse Events ... 141
12.3.1 Listing of Deaths, Other Serious Adverse Events and Other Significant Adverse Events 141

12.3.2 Narratives of Deaths, Other Serious Adverse Events and Certain Other Significant Adverse Events ... 141
12.3.3 Analysis and Discussion of Deaths, Other Serious Adverse Events and Other Significant Adverse Events ... 141
12.4 Clinical Laboratory Evaluation ... 147
12.4.1 List of Individual Laboratory Measurements by Patient and Each Abnormal Laboratory Value ... 147
12.4.2 Evaluation of Each Laboratory Parameter ... 148
12.5 Vital Signs, Physical Findings and Other Observations Related to Safety ... 150
12.5.1 Physical Examination (Systolic and Diastolic Blood Pressure, Pulse Rate, and Body Temperature) ... 150
12.5.2 Immunoserological Tests ... 150
12.6 Safety Conclusions ... 151
13. Discussion and Overall Conclusions ... 153
14. Tables, Figures and Graphs Referred to but Not Included in the Text ... 156
14.1 Demographic Data ... 156
14.2 Efficacy Data ... 156
14.3 Safety Data ... 157
14.3.1 Displays of Adverse Events ... 157
14.3.2 Listing of Deaths, Other Serious Adverse Events and Other Significant Adverse Events 159
14.3.3 Narratives of Deaths, Other Serious and Certain Other Significant Adverse Events ... 160
14.3.4 List of Individual Laboratory Measurements by Patient and Each Abnormal Laboratory Value ... 163
15. Reference List ... 175
16. Appendices ... 176
16.1 Study Information ... 176
16.1.1 Protocol and Protocol Amendments ... 176
16.1.2 Sample Case Report Form ... 176
16.1.3 List of IRBs, Representative Written Information for Patient and Sample Consent Form 176
16.1.4 List and Description of Investigators and Other Important Participants in the Study ... 176
16.1.5 Signature of Sponsor’s Responsible Medical Officer ... 176
16.1.6 Listing of Batch Numbers of Test Drug/Investigational Product Administered to Individual Patients ... 176
16.1.7 Randomized Scheme and Code (Patient Identification and Allocated Treatment) ... 176
16.1.8 Documentation of Audit Procedures and Audit Certificates ... 177
16.1.9 Documentation of Statistical Methods ... 177
16.1.10 Documentation of Inter-Laboratory Standardization Methods and Quality Assurance Procedures ... 177
16.1.11 Publication Based on the Study ... 177
16.1.12 Important Publications Referenced in the Report ... 177
16.1.13 Other Appendices ... 177
16.1.14 Documantation of Pharmacokinetics (if appricable) ... 177
16.2 Patient Data Listings ... 178
16.2.1 Patients Who Discontinued ... 178
16.2.2 Protocol Deviations ... 178
16.2.3 Patients Excluded from the Efficacy Analysis ... 178
16.2.4 Demographic Data ... 178

16.2.5 Compliance and/or Drug Concentration Data 178
16.2.6 Individual Efficacy Response Data 178
16.2.7 Listing of Adverse Events by Patient 178
16.2.8 List of Clinical Laboratory Test Values by Patient 178
16.3 Case Report Forms 179
16.3.1 CRFs for Deaths, Other Serious Adverse Events and Withdrawals for AE 179
16.3.2 Other CRFs Submitted 179

4. List of Abbreviations and Definitions of Terms

List of Abbreviation

Abbreviations	Terms not abbreviated<br>English
ATI	Antibodies to Infliximab
BCG	Bacille de Calmette et Guérin
BLQ	Below lower limit of quantification
CAI	Clinical activity index
CRP	C-reactive protein
CT	Computed tomography
dsDNA	double stranded DNA
EDC	Electronic Data Capture
ESR	Erythrocyte sedimentation rate
FAS	Full analysis set
GCP	Good clinical practice
HBc	Hepatitis B core
HBs	Hepatitis B surface
HIV	Human immunodeficiency virus
IL-6	Interleukin-6
INH	Isoniazid
LOCF	Last Observation Carried Forward
NC	Not calculated
NSAID	Non-steroidal anti-inflammatory drug
PUCAI	Pediatric Ulcerative Colitis Activity Index
Max	Maximum
Min	Minimum
n	Number of subjects
Q1	Lower Quartile
Q3	Upper Quartile
QFT	QuantiFERON
QOL	Quality of life
SD	Standard deviation
TB	Tuberculosis
TF	Treatment Failure
TNFα	Tumor necrosis factor - alpha

List of Definitions of Terms

Term	Definition
Registration day	Day when eligibility confirmation was completed.
Study period	Period from the starting day of the screening period to the last day of the evaluation period.
Screening period	Period from the starting day of the CAI score evaluation to the start of administration of the study drug.
Efficacy evaluation period	Period from the start of administration of the study drug to the evaluation day at Week 30; for patients who had no response measured by CAI score (CAI score-based non-responders), period from the start of administration of the study drug to the evaluation at Week 8; for discontinued patients, period from the start of administration of the study drug to the evaluation at the time of discontinuation.
Safety and pharmacokinetics evaluation period<br>(= Evaluation period)	Period from the start of administration of the study drug to the evaluation day at Week 30; for CAI score-based non-responders and discontinued patients, period from the start of administration of the study drug to the evaluation 8 weeks after the last administration.
Evaluation day	Day when the evaluation (medical examination) necessary to determine CAI score, partial Mayo score and PUCAI score was made.
CAI score-based responder	Patient who had a decreased (improved) CAI score at Week 8 compared to that measured at the time of registration.
CAI score-based non-responder	Patient who had an unchanged or increased (worsened) CAI score at Week 8 compared to that measured at the time of registration.
Evaluation period for CAI score based on Symptom Assessment Sheet<br>Evaluation period for partial Mayo score based on Symptom Assessment Sheet	The evaluation period for CAI score is the period from 7 days before the evaluation day to the day before the evaluation day; the evaluation period for Mayo score is the period from 3 days before the evaluation day to the day before the evaluation day. However, if drugs that might affect stool frequency were used, 7 days or 3 days before the evaluation day excluding the following days is used as the evaluation period for Symptom Assessment Sheet.<br>[Days excluded from evaluation]<br>• Days when drugs for the treatment of constipation or diarrhea were used<br>• Days when drugs having a potent antidiarrheal effect (e.g., loperamide hydrochloride, opium alkaloids, atropine sulfate-containing preparations) were used and for 2 days after the use of the above drugs (3 days in total)<br>• Days when drugs that might affect stool frequency (e.g., laxatives) were used as pretreatment of sigmoidoscopy<br>• Days when colonoscopy or sigmoidoscopy was performed and for 3 days after the examination (4 days in total)
CAI score	A sum (0 to 29 points) of subscores for 7 clinical criteria, consisting of the number of stools per week, blood in stools (based on weekly average), investigator's global assessment of symptomatic state, abdominal pain, temperature elevation due to ulcerative colitis, extraintestinal manifestations, and laboratory findings.

CAI score-based remission	Cases where a CAI score was not more than 4 on the evaluation day (excluding the evaluation at the time of registration).
Mayo score	A sum (0 to 12 points) of 4 Mayo subscores (stool frequency, rectal bleeding, physician's global assessment, and findings of endoscopy) assessed by the investigator (subinvestigator) based on a 4-point scale of 0 to 3.
Partial Mayo score	A sum (0 to 9 points) of Mayo subscores for stool frequency, rectal bleeding, and physician's global assessment.
Mayo score-based response	Case where the Mayo score measured on the evaluation day (excluding the evaluation at the time of registration) satisfied the following two criteria.<br>-Mayo score: A decrease by at least 30% and by at least 3 points compared to those measured at the time of registration.<br>- Rectal bleeding subscore: A decrease by at least 1 point compared to that measured at the time of registration or a rectal bleeding subscore of not more than 1 point.
Mayo score-based remission	Mayo score on the evaluation day (excluding the evaluation at the time of registration) was not more than 2 points and none of the subscores were more than 1 point.
PUCAI score	A sum (0 to 85 points) of subscores for 6 clinical conditions, consisting of abdominal pain, rectal bleeding, stool consistency, stool frequency per 24 hours, nocturnal stool, and activity level.
PUCAI score-based remission	Case where PUCAI score on the evaluation day (excluding the evaluation at the time of registration) was less than 10.
Steroid withdrawal	Case where the steroid dose on the evaluation day (excluding the evaluation at the time of registration) was 0.
Mucosal healing	A subscore for endoscopy findings of the Mayo score on the evaluation day (excluding the evaluation at the time of registration) was not more than 1, excluding patients whose subscore for findings of endoscopy at the time of registration was 1.
Infusion reaction	AEs observed during study drug administration or within 2 hours after the end of administration
Left-sided colitis	Lesion limited distal to the splenic flexure
Total colitis	Lesion extending proximal to the splenic flexure
Legal representative	An individual or judicial or other body authorized to consent to the subject's participation in a clinical study on behalf of a prospective subject who is incapable of giving informed consent. Legal representative includes parents, guardian, and any equivalent individual, and must be one who can act in the subject's best interest in terms of the mutual relation between actual quality of life and mental aspects.
Assent	Consent obtained from pediatric patients who were not subject to legal regulations.

List of Measurement Units

Measurement items	Measurement units	Measurement items	Measurement units
Blood pressure	mmHg	Albumin	g/dL
Pulse rate	beats/min	Total cholesterol	mg/dL
Body temperature	°C	Total bilirubin	mg/dL
Red blood cell (RBC) count	10^4/μL	BUN	mg/dL
Hemoglobin	g/dL	Serum creatinine	mg/dL
Hematocrit	%	Na	mEq/L
WBC count	μL	K	mEq/L
Differential count of WBC (neutrophils)	%	Cl	mEq/L
Differential count of WBC (eosinophils)	%	CRP	mg/dL
Differential count of WBC (basophils)	%	TNFα	pg/mL
Differential count of WBC (monocytes)	%	IL-6	pg/mL
Differential count of WBC (lymphocytes)	%	Double stranded DNA IgG antibody	IU/mL
Platelet count	10^4/μL	Double stranded DNA IgM antibody	U/mL
AST (GOT)	U/L	Antinuclear antibody	times
ALT (GPT)	U/L	Glucose in urine	Qualitative
ALP	U/L	Urine protein	Qualitative
LDH	U/L	Urobilinogen	Qualitative
γ-GTP	U/L	Occult blood in urine	Qualitative
Total protein	g/dL		

5. Ethics

5.1 Institutional Review Board (IRB)

Prior to the start of the study, the appropriateness of how the study was conducted was reviewed by each IRB for the 34 participating study sites from ethical, scientific and medical standpoints based on the investigator's brochure, protocol, written information for legal representatives and informed consent form, written information for patients and assent form, and sample CRF. As results, the conduct of the study was approved at 22 sites, and was decided to be approved after correction at the other 13 sites. The conditions for approval include partial modification of the written information for legal representatives and informed consent form, written information for patients and assent form and document about compensation to patients. All of these changes were minor and did not affect the entire aspect of the protocol. When the protocol, the investigator's brochure, written information for legal representatives and informed consent form, and written information for patients and assent form were revised during the study, the study was continued after obtaining approval from the IRBs. A list of the IRBs is provided in Appendix 16.1.3a.

5.2 Ethical Conduct of the Study

This study was conducted in accordance with ethical principles that have their origins in the Declaration of Helsinki and in compliance with "Act on Securing Quality, Efficacy, and Safety of Pharmaceuticals, Medical Devices, Regenerative and Cellular Therapy Products, Gene Therapy Products and Cosmetics," "Ministry of Health and Welfare Ordinance on Good Clinical Practice (GCP)" and the protocol.

Statements regarding GCP compliance were described in the protocol and the study contract, and the sponsor visited the medical institutions regularly and confirmed that the study was actually conducted in compliance with GCP. Safety information obtained during the study was immediately reported to investigators who made arrangements for a safe study. In preparation and handling of CRFs and submission of data to the regulatory authority, patient identification codes not linkable to the identity of the patients were used to ensure confidentiality of information of the patients.

Throughout the study period, no significant violation of GCP was reported from the medical institutions, and it was confirmed that this study was conducted ethically while ensuring safety of the patients.

5.3 Patient Information and Consent

Prior to the start of the study, the investigator (subinvestigator) provided legal representatives and candidate patients with the IRB-approved written information for legal representatives and informed consent forms, and written information for patients and assents form respectively, and fully explained the contents of the study. Supplemental explanations by a study collaborator were also allowed. The investigator (subinvestigator) tried to provide the explanations using language that was as simple as possible in a way that legal representatives and patients could easily understand, and sufficiently answer the questions from legal representatives and patients. After confirming that the legal representative and

patient had fully understood the contents of the study, the investigator (subinvestigator) obtained written informed consent from each legal representative and written assent (consent obtained from pediatric patients who were not subject to legal regulations), if possible from each patient of their own free will.

On the written informed consent form, the investigator (subinvestigator) who provided an explanation and the legal representative signed and sealed or placed their signatures, and the dates respectively. On the written assent form, the investigator (subinvestigator) who provided an explanation and the patient signed and sealed or placed their signature and the dates respectively. In the case where a study collaborator provided supplemental explanation, the relevant study collaborator also signed and sealed, or placed their signature, and the date. In addition, the investigator (subinvestigator) confirmed that the relationship between a legal representative and a patient was described on the written consent form signed by the legal representative. Before the start of administration of the study drug, consent was obtained from all of the patients by means of a written assent form.

Before patients participated in this study, the investigator (subinvestigator) issued a written information and consent form, and written information and assent form, both signed/sealed or signed and dated, to the legal representatives and patients, while the original copies of the consent form and assent form were properly stored in accordance with the rules of the relevant medical institution.

The date when informed consent was obtained and the version numbers of the written information and informed consent form used for explanation were recorded in the CRF.

When new important information which might affect the consent from legal representatives or the assent from patients were obtained, the investigator immediately judged the necessity for revision of the written information and consent form and the written information and assent form based on the obtained information. When revision of the written information and consent form and the written information and assent form was judged to be necessary, the investigator immediately revised the written information and consent form and the written information and assent form and submitted the revised documents to the sponsor, while obtaining approval from the IRB again. In this case, the investigator (subinvestigator) reported the relevant information to the patients who had already participated in the study and their legal representatives orally, confirmed whether they would continue to participate in the study or not, and recorded them in the medical record. The investigator (subinvestigator) gave an explanation to the patients who had already participated in the study and their legal representatives using the IRB-reapproved written information for patients and assent form and written information for legal representatives and informed consent form respectively, and obtained assent and written consent for continuous participation in the study from the patients and legal representatives respectively of their own free will. Similar to obtaining of the first consent, the investigator (subinvestigator) who provided an explanation, patients and the legal representative signed and sealed, or placed their signature and the dates respectively. In the case where a study collaborator provided supplemental explanation, the relevant study collaborators also signed and sealed or placed their signature and the dates. The investigator (subinvestigator) issued written information and consent form, and written information and assent form, both signed/sealed or signed and dated, to legal representatives and patients, while the original copies of the consent form and assent form were properly stored in accordance with the rules of the relevant medical institution. The dates when informed consent was again obtained and the version numbers of the written information and informed consent form used for the explanation were recorded in the CRF.

During the study period, the written information for legal representatives and informed consent form was revised 5 times due to the obtaining of safety information that might affect the patient's consent and amendments to the Japanese package insert. The written information for patients (for those aged 13 years or older) and assent form was revised 4 times, and the written information for patients (for those aged 12 years or younger) and assent form has not been revised. At each revision, written informed consent and assent were reobtained from all of the legal representatives and patients respectively participating in the study.

The 6th version (prepared on August 6, 2014) of written information for legal representatives and informed consent form (sponsor's final version), the 5th version (prepared on August 6, 2014) of written information for patients and assent form (for those aged 13 years or older, sponsor's final version), the 1st version (prepared on December 20, 2011) of written information for patients and assent form (for those aged 12 years or younger, sponsor's final version), a list of revisions, and a list of versions for medical institutions are attached in Appendix 16.1.3b to Appendix 16.1.3i.

6. Investigators and Study Administrative Structure

The history of changes until the date of preparation of this report is described as regards persons in charge at the sponsor, medical experts, clinical operation team leaders, and persons in charge of audit. For other items, the history of changes until the date of submission of clinical trial completion notification (April 24, 2015) is described.

6.1 Sponsor

6.1.1 Sponsor

At the start of study:
Mitsubishi Tanabe Pharma Corporation
2-6-18, Kitahama, Chuo-ku, Osaka 541-8505 Japan

Changed on March 30, 2015:
Mitsubishi Tanabe Pharma Corporation
3-2-10, Dosho-machi, Chuo-ku, Osaka 541-8505 Japan

6.1.2 Person in Charge at the Sponsor

At the start of study:
Masahiko Tanaka, Department Manager, Clinical Planning Department, Development Division, Mitsubishi Tanabe Pharma Corporation
2-2-6, Nihonbashi-Honcho, Chuo-ku, Tokyo 103-8405 Japan
Phone: 03-3241-4136, Fax: 03-3241-4807

Scope of services: Approval of the study plan and obtaining agreement with investigators about contents of sample of protocol and CRF and compliance with the protocol.

Changed on April 1, 2012:
Masahiko Tanaka, Department Manager, Clinical Planning Department I, Development Division, Mitsubishi Tanabe Pharma Corporation
2-2-6, Nihonbashi-Honcho, Chuo-ku, Tokyo 103-8405 Japan
Phone: 03-3241-4136, Fax: 03-3241-4807

Changed on May 7, 2012:
Masahiko Tanaka, Department Manager, Clinical Planning Department I, Development Division, Mitsubishi Tanabe Pharma Corporation
Tel: 03-6748-7691, Fax: 03-3663-6258
17-10, Nihonbashi-Koamicho, Chuo-ku, Tokyo 103-8405 Japan
Phone: 03-6748-7691, Fax: 03-3663-6258

Changed on April 5, 2013:
Yoshihiro Kobayashi, Department Manager, Clinical Planning Department I, Development Division, Mitsubishi Tanabe Pharma Corporation

17-10, Nihonbashi-Koamicho Chuo-ku, Tokyo 103-8405 Japan
Phone: 03-6748-7691, Fax: 03-3663-6258

Changed on October 7, 2014:
Hitoshi Izaki, Department Manager, Clinical Development Department III, Development Division, Mitsubishi Tanabe Pharma Corporation
17-10, Nihonbashi-Koamicho, Chuo-ku, Tokyo 103-8405 Japan
Phone: 03-6748-7691, Fax: 03-3663-6277

Changed on October 1, 2015:
Hitoshi Izaki, Department Manager, Clinical Development Department III, Sohyaku Innovative Research Division, Mitsubishi Tanabe Pharma Corporation
17-10, Nihonbashi-Koamicho, Chuo-ku, Tokyo 103-8405 Japan
Phone: 03-6748-7691, Fax: 03-3663-6277

6.1.3 Medical Experts

At the start of study:
Toshifumi Hibi, Professor, Department of Internal Medicine, Keio University School of Medicine
35, Shinano-machi, Shinjyuku-ku, Tokyo 160-8582 Japan
Phone: 03-3353-1211, Fax: 03-3357-6156

Kazuoki Kondo, Advisor, Mitsubishi Tanabe Pharma Corporation
2-2-6, Nihonbashi-Honcho, Chuo-ku, Tokyo 103-8405 Japan
Phone: 03-3241-4954, Fax: 03-3241-4785

Scope of services: Providing guidance and advice rapidly to the sponsor about medical issues in the study.

Changed on May 7, 2012:
Kazuoki Kondo, Advisor, Mitsubishi Tanabe Pharma Corporation
17-10, Nihonbashi-Koamicho, Chuo-ku, Tokyo 103-8405 Japan
Phone: 03-6748-7685, Fax: 03-3663-6254

Changed on April 5, 2013:
Toshifumi Hibi, Director, Center for Advanced IBD Research and Treatment, Kitasato University Kitasato Institute Hospital, The Kitasato Institute
5-9-1, Shirogane, Minato-ku, Tokyo 108-8642 Japan
Phone: 03-3444-6161

Changed on April 7, 2014:
Toshifumi Hibi, Director, Center for Advanced IBD Research and Treatment, Kitasato University Kitasato Institute Hospital, The Kitasato Institute
5-9-1, Shirogane, Minato-ku, Tokyo 108-8642 Japan
Phone: 03-3444-6161

Kazuoki Kondo, Advisor, Mitsubishi Tanabe Pharma Corporation
17-10, Nihonbashi-Koamicho, Chuo-ku, Tokyo 103-8405 Japan
Phone: 03-6748-7681, Fax: 03-3663-6254

6.1.4 Clinical Leader

At the start of study:
Kunihiko Ozaki, Clinical Leader, Clinical Planning Department, Development Division, Mitsubishi Tanabe Pharma Corporation
2-2-6, Nihonbashi-Honcho, Chuo-ku, Tokyo 103-8405 Japan
Phone: 03-3241-4713, Fax: 03-3241-4785

Scope of services: Generally responsible for the group that handled planning of the study, completion of results of the study and preparation of the clinical study report.

Changed on April 1, 2012:
Kunihiko Ozaki, Manager, Clinical Planning Department I, Development Division, Mitsubishi Tanabe Pharma Corporation
2-2-6, Nihonbashi-Honcho, Chuo-ku, Tokyo 103-8405 Japan
Phone: 03-3241-4713, Fax: 03-3241-4785

Changed on May 7, 2012:
Kunihiko Ozaki, Manager, Clinical Planning Department I, Development Division, Mitsubishi Tanabe Pharma Corporation
17-10, Nihonbashi-Koamicho, Chuo-ku, Tokyo 103-8405 Japan
Phone: 03-6748-7696, Fax: 03-3663-6258

Changed on April 7, 2014:
COT Leader *COT: Clinical Operation Team
Toru Yoshinari, Manager, Clinical Planning Department I, Development Division, Mitsubishi Tanabe Pharma Corporation
17-10, Nihonbashi-Koamicho, Chuo-ku, Tokyo 103-8405 Japan
Phone: 03-6748-7696, Fax: 03-3663-6258

Changed on October 7, 2014:
Kunihiko Ozaki, Manager, Clinical Development Department III, Development Division, Mitsubishi Tanabe Pharma Corporation
17-10, Nihonbashi-Koamicho, Chuo-ku, Tokyo 103-8405 Japan
Phone: 03-6748-7696, Fax: 03-3663-6277

Changed on October 1, 2015:
Hiroshi Yamada, Manager, Clinical Development Department III, Sohyaku. Innovative Research Division, Mitsubishi Tanabe Pharma Corporation
17-10, Nihonbashi-Koamicho, Chuo-ku, Tokyo 103-8405 Japan
Phone: 03-6748-7696, Fax: 03-3663-6277

6.1.5 Person Responsible for Monitoring

At the start of study:
Hiromichi Yoshida, Manager, Clinical Development Department II, Clinical Development Center, Development Division, Mitsubishi Tanabe Pharma Corporation

2-2-6, Nihonbashi-Honcho, Chuo-ku, Tokyo 103-8405 Japan
Phone: 03-3241-4740, Fax: 03-3241-4744

Scope of services: Generally responsible for the group making requests for participation in the study and monitoring the study.

Changed on April 1, 2012:
Hiromichi Yoshida, Manager, Clinical Development Department I, Clinical Development Center, Development Division, Mitsubishi Tanabe Pharma Corporation
2-2-6, Nihonbashi-Honcho, Chuo-ku, Tokyo 103-8405 Japan
Phone: 03-3241-4740, Fax: 03-3241-4744

Changed on May 7, 2012
Yukio Matsumoto, Manager, Clinical Development Department I, Clinical Development Center, Development Division, Mitsubishi Tanabe Pharma Corporation
17-10, Nihonbashi-Koamicho, Chuo-ku, Tokyo 103-8405 Japan
Phone: 03-6748-7722, Fax: 03-3663-6277

Changed on April 7, 2014:
Kunihiko Ozaki, Manager, Clinical Development Department II, Development Division, Mitsubishi Tanabe Pharma Corporation
17-10, Nihonbashi-Koamicho, Chuo-ku, Tokyo 103-8405 Japan
Phone: 03-6748-7722, Fax: 03-3663-6277

Changed on October 7, 2014:
Hironobu Tsuru, Manager, Clinical Development Department III, Development Division, Mitsubishi Tanabe Pharma Corporation
17-10, Nihonbashi-Koamicho, Chuo-ku, Tokyo 103-8405 Japan
Phone: 03-6748-7721, Fax: 03-3663-6277

6.1.6 Monitors

Monitors are given in Attachment 1 of Appendix 16.1.1a.

Scope of services: Confirming that human rights, safety, and the welfare of the patients are protected; that the study is conducted in compliance with the latest protocol and GCP, etc.; and that the study data are accurate and complete and can be examined with study-related records including source documents

6.1.7 Person in Charge of Audit

At the start of study:
Masahiro Kusuda, Group Manager, GCP Audit Group of Clinical & Research Quality Assurance Department in Pharmacovigilance & Quality Assurance Division, Mitsubishi Tanabe Pharma Corporation

2-2-6, Nihonbashi-Honcho, Chuo-ku, Tokyo 103-8405 Japan
Phone: 03-3241-3571, Fax: 03-3241-5205

Scope of services: Systematic and independent examination of work and documents related to the study to evaluate that work and records, analysis and accurate reports of data related to the study were conducted in accordance with Pharmaceutical Affairs Law, regulatory requirements including GCP ordinance, standard operating procedures and protocols.

Changed on April 1, 2012:
Masahiro Kusuda, Group Manager, Clinical Quality Assurance Group, Clinical & Research Quality Assurance Department, Pharmacovigilance & Quality Assurance Division, Mitsubishi Tanabe Pharma Corporation
2-2-6, Nihonbashi-Honcho, Chuo-ku, Tokyo 103-8405 Japan
Phone: 03-3241-3571, Fax: 03-3241-5205

Changed on May 7, 2012:
Masahiro Kusuda, Group Manager, Clinical Quality Assurance Group, Clinical & Research Quality Assurance Department, Pharmacovigilance & Quality Assurance Division, Mitsubishi Tanabe Pharma Corporation
17-10, Nihonbashi-Koamicho, Chuo-ku, Tokyo 103-8405 Japan
Phone: 03-6748-7706, Fax: 03-3663-6698

Changed on April 5, 2013:
Masahiro Kusuda, Clinical Quality Assurance Group, Clinical & Research Quality Assurance Department, Pharmacovigilance & Quality Assurance Division, Mitsubishi Tanabe Pharma Corporation
17-10, Nihonbashi-Koamicho, Chuo-ku, Tokyo 103-8405 Japan
Phone: 03-6748-7706, Fax: 03-3663-6698

Changed on October 1, 2013:
Masahiro Kusuda, Clinical Quality Assurance Department, Development Division, Mitsubishi Tanabe Pharma Corporation
17-10, Nihonbashi-Koamicho, Chuo-ku, Tokyo 103-8405 Japan
Phone: 03-6748-7743, Fax: 03-3663-6382

Changed on April 7, 2014:
Kyoko Ihara, General Manager, Clinical Quality Assurance Department, Development Division, Mitsubishi Tanabe Pharma Corporation
17-10, Nihonbashi-Koamicho, Chuo-ku, Tokyo 103-8405 Japan
Phone: 03-6748-7741, Fax: 03-3663-6382

Changed on October 1, 2015:
Kyoko Ihara, Manager of Sohyaku Assurance, Sohyaku Assurance Department in Sohyaku. Innovative Research Division, Mitsubishi Tanabe Pharma Corporation
17-10, Nihonbashi-Koamicho, Chuo-ku, Tokyo 103-8405 Japan
Phone: 03-6748-7741, Fax: 03-3663-6382

6.1.8 Statistical Analysis Manager

At the start of study:
Chikao Ishikawa, Group Manager, Clinical analysis group of Data Science Department in

Development Division, Mitsubishi Tanabe Pharma Corporation
17-10, Nihonbashi-Koamicho, Chuo-ku, Tokyo 103-8405 Japan
Phone: 03-6748-7735, Fax: 03-3663-6371

Scope of services: Responsible for statistical items of the study, preparation of a statistical analysis plan based on protocol, conducting statistical analyses and confirming them.

Changed on April 1, 2012:
Chikao Ishikawa, Section Manager, Biostatistics & Data Management Office, Data Science Center, Development Division, Mitsubishi Tanabe Pharma Corporation
2-2-6, Nihonbashi-Honcho, Chuo-ku, Tokyo 103-8405 Japan
Phone: 03-3241-4198, Fax: 03-3241-4717

Changed on May 7, 2012:
Chikao Ishikawa, Section Manager, Biostatistics & Data Management Office, Data Science Center, Development Division, Mitsubishi Tanabe Pharma Corporation
17-10, Nihonbashi-Koamicho, Chuo-ku, Tokyo 103-8405 Japan
Phone: 03-6748-7735, Fax: 03-3663-6371

Changed on April 7, 2014:
Chikao Ishikawa, Data Science Department, Development Division, Mitsubishi Tanabe Pharma Corporation
17-10, Nihonbashi-Koamicho, Chuo-ku, Tokyo 103-8405 Japan
Phone: 03-6748-7735, Fax: 03-3663-6371

6.1.9 Data Control Manager

At the start of study:
Yoshihiro Tanaka, Clinical DM Group, Data Science Department, Development Division, Mitsubishi Tanabe Pharma Corporation
2-2-6, Nihonbashi-Honcho, Chuo-ku, Tokyo 103-8405 Japan
Phone: 03-3241-7902, Fax: 03-3241-4717

Scope of services: Responsible for making an effort to comprehensively ensure the quality of the study data for quality control and retain the data collected by CRFs.

Changed on April 1, 2012:
Yoshiteru Ushirogawa, Manager, Clinical Pharmacology Department, Data Science Center, Development Division, Mitsubishi Tanabe Pharma Corporation
2-2-6, Nihonbashi-Honcho, Chuo-ku, Tokyo 103-8405 Japan
Phone: 03-3241-7902, Fax: 03-3241-4717

Changed on May 7, 2012:
Yoshiteru Ushirogawa, Manager, Data Management Department, Data Science Center, Development Department, Mitsubishi Tanabe Pharma Corporation
17-10, Nihonbashi-Koamicho, Chuo-ku, Tokyo 103-8405 Japan
Phone: 03-6748-7737, Fax: 03-3663-6371

Changed on April 5, 2013:
Yoshiteru Ushirogawa, Manager, Data Management Department, Data Science Center, Development Department, Mitsubishi Tanabe Pharma Corporation
17-10, Nihonbashi-Koamicho, Chuo-ku, Tokyo 103-8405 Japan
Phone: 03-6748-7737, Fax: 03-3663-6451

Changed on April 7, 2014:
Yoshiteru Ushirogawa, Group Manager, Data Management Group, Data Science Department, Development Division, Mitsubishi Tanabe Pharma Corporation
17-10, Nihonbashi-Koamicho, Chuo-ku, Tokyo 103-8405 Japan
Phone: 03-6748-7737, Fax: 03-3663-6451

6.1.10 Person Responsible for Clinical Pharmacological Analysis

At the start of study:
Kei Akimoto, Group Manager, Pharmacometrics Group, Clinical Pharmacology Department, Clinical Pharmacology Center, Development Division, Mitsubishi Tanabe Pharma Corporation
2-2-6, Nihonbashi-Honcho, Chuo-ku, Tokyo 103-8405 Japan
Phone: 03-3241-4735, Fax: 03-3241-4757

Scope of services: Responsible for issues related to pharmacokinetics of the study, preparation of pharmacokinetic analysis plan based on protocols, conducting pharmacokinetic analyses, and confirming them.

Changed on April 1, 2012:
Kei Akimoto, Manager, Clinical Pharmacology Department, Clinical Pharmacology Center, Development Division, Mitsubishi Tanabe Pharma Corporation
2-2-6, Nihonbashi-Honcho, Chuo-ku, Tokyo 103-8405 Japan
Phone: 03-3241-4735, Fax: 03-3241-4757

Changed on May 7, 2012:
Kei Akimoto, Manager, Clinical Pharmacology Department, Clinical Pharmacology Center, Development Division, Mitsubishi Tanabe Pharma Corporation
17-10, Nihonbashi-Koamicho, Chuo-ku, Tokyo 103-8405 Japan
Phone: 03-6748-7763, Fax: 03-3663-6449

Changed on April 5, 2013:
Shigekazu Nakajima, Manager, Clinical Pharmacology Department, Data Science Center, Development Division, Mitsubishi Tanabe Pharma Corporation
17-10, Nihonbashi-Koamicho, Chuo-ku, Tokyo 103-8405 Japan
Phone: 03-6748-7763, Fax: 03-3663-6449

Changed on April 7, 2014:
Atsuhiro Kawaguchi, Manager, Clinical Pharmacology Department, Development Division, Mitsubishi Tanabe Pharma Corporation
17-10, Nihonbashi-Koamicho, Chuo-ku, Tokyo 103-8405 Japan
Phone: 03-6748-7763, Fax: 03-3663-6449

6.1.11 Person Responsible for Pharmacokinetics and Other Measurement Items

At the start of study:
Naohisa Tsutsui, Group Manager, Bioscience Group, Clinical Pharmacology Department, Clinical Pharmacology Center, Development Division, Mitsubishi Tanabe Pharma Corporation
2-2-6, Nihonbashi-Honcho, Chuo-ku, Tokyo 103-8405 Japan
Phone: 03-3241-4734, Fax : 03-3241-4757

Scope of services: Responsible for measurement of drug concentration and pharmacodynamic indices in the study, preparing pharmacokinetic measurement protocols based on the protocols, conducting measurement of drug concentrations and pharmacodynamic indices, and confirming them.

Changed on April 1, 2012:
Naohisa Tsutsui, Manager, Clinical Pharmacology Department, Clinical Pharmacology Center, Development Division, Mitsubishi Tanabe Pharma Corporation
3-16-89, Kashima, Yodogawa-ku, Osaka 532-8505 Japan
Phone: 06-6300-2565, Fax: 06-6300-2586

Changed on April 5, 2013:
Masaki Sakai, Manager, Clinical Pharmacology Department, Data Science Center, Development Division, Mitsubishi Tanabe Pharma Corporation
2-6-18, Kitahama, Chuo-ku, Osaka 541-8505 Japan
Phone: 06-6205-5571, Fax: 06-6205-5241

Changed on April 7, 2014:
Masaki Sakai, Manager, Clinical Pharmacology Department, Mitsubishi Tanabe Pharma Corporation
2-6-18, Kitahama, Chuo-ku, Osaka 541-8505 Japan
Phone: 06-6205-5571, Fax: 06-6205-5241

6.1.12 Contract Research Organization

6.1.12.1 Data Control Operation Contract Facility

EPS Corporation
2-23, Shimomiyabicho, Shinjuku-ku, Tokyo 162-0822 Japan
Phone: 03-5684-7797, Fax: 03- 5684-4785

Scope of services: To perform data control operations according to the operation consignment agreement.

6.1.12.2 Electronic Data Capture (EDC) vendor

Oracle Corporation Japan
Oracle Aoyama Center, 2-5-8, Kita-Aoyama, Minato-ku, Tokyo 107-0061 Japan
Phone: 03-6834-6666

Scope of services: To perform operation, maintenance and control of the EDC system.

6.1.12.3 Statistical Analysis Facility

Takumi Information Technology Inc.
Ikebukuro Duplex B's, 2-40-13, Ikebukuro, Toshima-ku, Tokyo 171-0014 Japan
Phone: 03-5979-7381, Fax: 03-5979-7382

Scope of services: To perform part of the following operations of the sponsor according to the operation consignment agreement.

(1) Duties of the statistical analysis

(2) Duties related to quality control for the above duties

6.1.12.4 Investigational Product Control Operation Contract Facility

Warehouse Business Section I, Kobe Branch, Mitsubishi Logistics Corporation
1-7-4, Higashi Kawasaki-cho, Chuo-ku, Kobe 650-8691 Japan
Phone: 078-360-8030, Fax: 078-360-8050

Scope of services: To perform part of the investigational product control operations of the sponsor according to the operation consignment agreement.

6.1.12.5 Concentration Assay Facility

Osamu Aoki, Department Manager, Analytical Chemistry Department, Tanabe R&D Service Co., Ltd.
2-2-50, Kawagishi, Toda-shi, Saitama 335-8505 Japan
Phone: 048-433-2551, Fax: 048-433-2554

Scope of services: To measure and report drug concentrations according to according to the study protocol.

6.1.12.6 Laboratory Test Contract Facility

Operational Management Department, Medical Solutions Division, LSI Medience Corporation
Contract Manager: Fumihiro Totsuka Person in Charge of Business Promotion: Hideki Ito
3-30-1, Shimura, Itabashi-ku, Tokyo 174-8555 Japan
Phone: 03-5943-9270, Fax: 03-5375-9211

Scope of services: To perform laboratory tests and report the results according to the study-related standard operating procedures (SOP).

6.1.12.7 Emergency Contact Center

Clinical Control Headquarter, Bell Medical Solutions Inc.
Emergency Contact Manager: Miho Tanaka

Tokyu Bldg. East No. 3, 2-16-8, Minami Ikebukuro, Toshima-ku, Tokyo 171-0022 Japan
Phone: 0120-3274-99

Scope of services: To respond to emergency contacts at night and on holidays.

6.2 Coordinating Investigator

Tajiri Hitoshi, Senior Director, Department of Pediatrics, Osaka General Medical Center
3-1-56, Bandaihigashi, Sumiyoshi-ku, Osaka 558-8558 Japan
Phone: 06-6692-1201, Fax: 06-6695-3559

Scope of services: To coordinate interpretations of the study protocol and details of the study in participating study centers.

6.3 Safety Evaluation Committee

Akira Watanabe, Professor, Research Division for Development of Anti-Infective Agents, Institute of Development, Aging and Cancer, Tohoku University

Scope of services: If any serious adverse events including infections occurred during the period of the study, the safety evaluation committee members assessed the safety information and gave the sponsor advice on the continuation, revision or discontinuation of the study.

6.4 Study Centers and Investigators

A list of participating study centers and investigators is provided in Appendix 16.1.4.

Scope of services of investigators: Investigators agree on the study protocol prepared by the sponsor, prepare and revise the written information and consent form, select candidate patients and obtain consent from them, conduct the study, provide medical care and information to patients, give guidance and supervise subinvestigators and study collaborators, provide materials and information, provide help in monitoring and audit, report deviations from or changes of the study protocol and adverse events, prepare case report forms, and store documents or records related to this study.

7. Introduction

The study drug TA-650 (non-proprietary name: infliximab, trade name: Remicade®) is an anti-human tumor necrosis factor-α (TNFα) monoclonal antibody discovered and manufactured by Centocor, Inc. (present Janssen Biotech, Inc., US) using genetic engineering techniques, and that affects various inflammatory diseases related to TNFα by neutralizing TNFα and by damaging cells expressing membrane-bound TNFα. From 1998 to August 2014, TA-650 has been approved in 105 countries, including Japan and administered to approximately 2.21 million patients. In Japan, TA-650 has been approved as a treatment for Crohn’s disease, rheumatoid arthritis, ankylosing spondylitis, psoriasis vulgaris, psoriasis arthropathica, pustular psoriasis, erythrodermic psoriasis, ulcerative colitis, refractory uveoretinitis induced by Behcet’s disease, and gastrointestinal-, neuro- and vascular Behcet’s disease. In addition, dose increase and shortening of the dosing interval for rheumatoid arthritis and dose increase for Crohn’s disease, and gastrointestinal-, neuro- and vascular Behcet’s disease have been approved. Presently, the applications of partial changes in the approved items for a dose increase for Kawasaki disease and psoriasis have been submitted. TA-650 has been approved for Crohn’s disease and ulcerative colitis in pediatric patients overseas.

Ulcerative colitis is a diffuse non-specific inflammatory disease of unknown etiology, primarily affecting the mucosa membrane of the large intestine where erosion and ulcers are often formed. Ulcerative colitis is a disease with lesions continuously extending proximally from the rectum proximally, up to the entire colon, and is characterized by chronic bloody mucoid stools and bloody stools. Some patients with ulcerative colitis repeat relapse and remission, and require long-term medical control due to difficulties in treatment. At present, removal of the large intestine is the only therapy to radically treat ulcerative colitis, and ulcerative colitis is a specified intractable disease. The number of patients with ulcerative colitis has been increasing, and a medical care certificate for patients with ulcerative colitis was issued to more than 110,000 patients in fiscal 2009, when this study was planned, and more than 150,000 patients had the medical care certificate for patients with ulcerative colitis in fiscal 2013. Though the number of pediatric patients experiencing onset in childhood is small compared to adult patients, it is showing a trend to increase the same as in adult patients.

Primary treatment of ulcerative colitis is drug therapy. Some patients repeat relapse and remission, resulting in difficulty in treating the disease. The Guidelines for the Treatment of Ulcerative Colitis (2010) established by the “Intractable Inflammatory Bowel Disease Study Group” of the MHLW, which was the current version when this study was planned, stated that the therapeutic plan should be established based on severity and lesion extent. Patients with mild to moderate ulcerative colitis are treated with oral preparations or intestinal injection of aminosalicylates or intestinal injection of steroids. Patients refractory to the above agents or having a severe form of ulcerative colitis are treated with oral preparations or intravenous infusion of steroids. Steroid therapy is the primary treatment for ulcerative colitis, and a good remission induction therapy if used under an adequate dosing regimen. However, steroid therapy is less effective in maintaining remission, and administering steroids without any specific goal would cause more adverse drug reactions. Immunomodulators are used on steroid-dependent or resistant patients which are refractory cases. Treatment with cyclosporine, tacrolimus and cytapheresis is used for the purpose of remission induction; and azathioprine and 6-mercaptopurine are used for the purpose of remission maintenance. However, there exist numerous cases where patients are not adequately responding to treatment with existing drugs, and are eventually referred to surgery. Against this background, TA-650 was approved for use in patients with ulcerative colitis in June 2010, and positioned as a therapeutic drug for ulcerative colitis to be used in steroid-resistant or dependent patients

by the above-mentioned guideline for treatment. These problems relating to the therapy for ulcerative colitis are also observed in pediatric patients. Furthermore, pediatric ulcerative colitis is characterized by a higher likelihood of extended and/or severe lesions compared to adults. In addition, long-term administration of steroids may be likely to cause steroid-related complications such as failure to thrive and steroid dependence and therefore many patients receive active therapies such as immunomodulators.

The results of the C0168T72 study on pediatric patients with ulcerative colitis and the ACT1 and ACT2 studies in adult patients, each conducted in foreign countries suggested that efficacy, safety profile, and pharmacokinetics of TA-650 in children are similar to those in adults. Based on the results of the above studies, TA-650 was approved for treatment of ulcerative colitis in children under a dosing regimen of 5 mg/kg administration at Weeks 0, 2 and 6 followed by administration at 8-week intervals, which is the same regimen as that for adults, in the US and EU respectively in September 2011 and February in 2012.

In contrast, no domestic clinical trials of TA-650 in pediatric patients with ulcerative colitis have been conducted, and “children” is not clearly specified in the description of dosage and administration, therefore physicians may hesitate to use TA-650 on pediatric patients with ulcerative colitis who require administration of this drug. To resolve these problems, it was decided to conduct a clinical study of TA-650 in pediatric patients with ulcerative colitis to evaluate the efficacy, safety and pharmacokinetics of TA-650 when administered at a dose of 5 mg/kg at Weeks 0, 2 and 6 followed by administration at 8-week intervals at Weeks 14 and 22.

In preparing the protocol for this study, we held face-to-face consultations with the Pharmaceuticals and Medical Devices Agency on December 21, 2010. The minutes of this consultation (prepared on February 3, 2011) are attached as Appendix 16.1.13h.

In this consultation, we agreed to widely evaluate not only the pharmacokinetics but also the efficacy and safety, and to make a comprehensive judgment, since the evaluation would be performed based on the data obtained from limited number of patients in a clinical trial in pediatric patients with ulcerative colitis. In terms of evaluation indices, endoscopy causes physical and emotional suffering in patients, and also places a great burden on physicians as well as patients since pediatric patients as young as 6 to 8 years old sometimes undergo endoscopy under general anesthesia, it was therefore considered difficult to add endoscopy as an essential test item. However, since endoscopy is an important test item for evaluating the efficacy of the study drug in patients with ulcerative colitis, we agreed to perform endoscopy as much as possible in order to accumulate data. In addition, we agreed to give consideration to avoiding creating an imbalance to a certain age group among the target patient ages.

8. Study Objective

The purpose of this study was to evaluate efficacy of TA-650 up to Week 30 when administered to pediatric patients with moderate to severe ulcerative colitis at a dose of 5 mg/kg at Weeks 0, 2 and 6, followed by administration at 8-week intervals at Weeks 14 and 22 using the evaluation indices including CAI score. Safety and pharmacokinetics of TA-650 are also assessed.

Efficacy endpoints:

(1) CAI score
(2) CAI score-based remission
(3) Partial Mayo score
(4) Mayo score
(5) Mayo score-based response
(6) Mayo score-based remission
(7) Mucosal healing
(8) PUCAI score
(9) PUCAI score-based remission
(10) PUCAI score decrease by at least 20 points
(11) Steroid dose
(12) Steroid withdrawal

Safety endpoints:

(1) Adverse events and adverse drug reactions
- Adverse events (AEs)
- Adverse drug reactions (ADRs)

Pharmacokinetic endpoints:

(1) Serum infliximab concentration (serum TA-650 concentration)
(2) ATI

9. Investigational Plan

9.1 Overall Study Design and Plan–Description

The protocol (Ver. 02.00.00000, prepared on May 22, 2013) and the list of changes in the protocol are respectively attached as Appendix 16.1.1a and Appendix 16.1.1b, and the case report form (Ver. 01.00.00000, prepared on December 20, 2011) is attached as Appendix 16.1.2. All of the medical institutions that the sponsor requested to conduct the study used the protocol and the case report form sample prepared by the sponsor, and no medical institution version of each document was prepared.

9.1.1 Treatment Method Studied

TA-650 was administered at a dose of 5 mg/kg based on the body weight on each day of administration by intravenous drip infusion over at least a 2-hour period. Patients were given the study drug at the initial administration (Week 0) and at Weeks 2 and 6, and the responders measured by a CAI score (CAI score-based responder) determined at Week 8 were further given the study drug at Weeks 14 and 22. However, the CAI score-based non-responders were not given the study drug after Week 14.

9.1.2 Patient Population Studied and Planned Number of Patients to be Included

Patient population studied: Pediatric patients with moderate to severe ulcerative colitis

Planned sample size: 20 patients receiving TA-650

9.1.3 Level and Method of Blinding

Not applicable because this study was an open-label study.

9.1.4 Type of Control and Study Configuration

Not applicable because this study was an uncontrolled study.

9.1.5 Method of Assignment to Treatment

Not applicable because this study was an uncontrolled study.

9.1.6 Sequence and Duration of Study Period

The study design is shown in Figure 9.1–1.

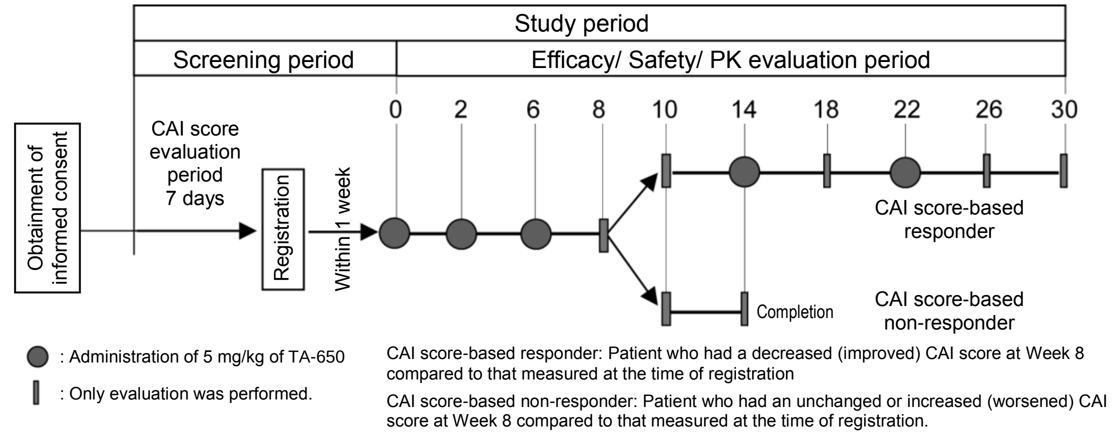

Figure 9.1–1 Study Design

Patients who had an unchanged or increased (worsened) CAI score at Week 8 compared to that measured at the time of registration (CAI score-based non-responders) were not given the study drug after Week 14, and completed the study after the end of evaluation of safety and pharmacokinetics at Weeks 10 and 14.

Study period: Period from the starting day of the screening period to the last day of the evaluation period.

Screening period: Period from the starting day of CAI score evaluation to the start of administration of the study drug.

Evaluation period: Period from the start of administration of the study drug to the evaluation day at Week 30. For CAI score-based non-responders and discontinued patients, the period from the start of administration of the study drug to the evaluation 8 weeks after the last administration.

[Definition of each evaluation period]

Efficacy evaluation period:

Period from the start of administration of the study drug to the evaluation day at Week 30. For CAI score-based non-responders, the period from the start of administration of the study drug to the evaluation at Week 8; for discontinued patients, the period from the start of administration of the study drug to the evaluation at the time of discontinuation.

Safety and pharmacokinetic evaluation period (= Evaluation period):

Period from the start of administration of the study drug to the evaluation day at Week 30. For CAI score-based non-responders and discontinued patients, the period from the start of administration of the study drug to the evaluation 8 weeks after the last administration.

9.1.7 Identification of When Patients were Randomized

Not applicable because this study was an uncontrolled study.

9.1.8 Various Committees Established and Their Roles

Based on the results obtained by previous domestic and overseas clinical studies and post-marketing safety information for TA-650, it is known that TA-650 should be administered while giving sufficient attention to serious infections such as tuberculosis, opportunistic infection and sepsis, so it was necessary to proceed with this study by paying careful attention to the safety of patients. Consequently, the safety evaluation committee was established for the purpose of evaluating safety information obtained during the study from an objective standpoint and providing the sponsor with advice concerning continuation, change or discontinuation of the study.

The safety evaluation committee consisted of one physician who was independent of the sponsor, the medical expert and investigators (subinvestigators), and possessed expertise in infections, and the member of the committee evaluated safety information in accordance with the operating procedures for the safety evaluation committee. The operating procedures for the safety evaluation committee are attached as Appendix 16.1.13b.

9.1.9 Interim Analyses

Interim analyses were not planned and performed.

9.2 Discussion of Study Design, Including the Choice of Control Groups

Reasons for open label and uncontrolled:

Pediatric ulcerative colitis is characterized by a higher likelihood of extended and/or more severe lesions compared to adults, a likelihood of steroid-related complications such as failure to thrive and a likelihood of having steroid dependence, so the therapy of ulcerative colitis in children aims at suppressing disease activity to improve their quality of life (hereinafter referred to as QOL) and avoiding failure to thrive. Since a study using placebo as a comparator would adversely affect the growth and development of patients to be studied in this study who have inadequate response to the existing therapies, use of placebo in this study was considered to present ethical difficulties. In addition, since the “pediatric” patients with “moderate to severe ulcerative colitis,” who received the medical care certificate for ulcerative colitis in fiscal 2009, to be studied in this study included those who might be able to maintain remission by existing therapies and those with indications for surgical procedures such as failure to thrive, undernutrition, and worsened QOL specific to children, it was considered that patients actually eligible for this study would be even fewer and the number of patients accumulated for this study would be limited. Consequently, this study was designed to be conducted as an open label and uncontrolled study.

9.3 Selection of Study Population

9.3.1 Inclusion Criteria

Patients who satisfied all of the following inclusion criteria were included in the study (including both inpatients and outpatients).

(1) Target disease: Ulcerative colitis

Patients who were diagnosed as having ulcerative colitis according to the draft of the Diagnostic Criteria established by the MHLW “Study Group for Specific Diseases/Intractable Inflammatory Bowel Disease” (revised on February 13, 2010) (Attachment 1 of Appendix 16.1.1a), and had been suffering for at least 3 months at the time of consent obtainment.

(2) Patients aged not less than 6 years and not more than 17 years (at the time of consent obtainment).

(3) Patients whose legal representative would give consent in writing, and who can give oral or written assent by themselves for participation in this study. (Depending on the understanding of each patient, written assent was obtained from patients of approximately junior high school age or older. For patients of approximately less than junior high school age, written assent was obtained if possible.)

(4) Patients with a CAI score of at least 7 and blood in stools score in a CAI score of at least 2 at the time of registration.

(5) Patients who satisfied at least one of the treatment experiences listed in the following 1) to 4).

1) 6-Mercaptopurine or azathioprine: Patients who had been using the drug for at least 12 weeks before the starting day of screening period, and receiving at a stable dose for at least 4 weeks before the starting day of screening period.

2) Steroids (oral preparations): Patients who had been using the drug at a stable dose of at least 1 mg/kg/day or at least 20 mg/day calculated on a prednisolone basis for at least 2 weeks before the starting day of screening period.

3) Patients who experienced either of the following within 5 years before the starting day of the screening period.

• Had used 6-mercaptopurine or azathioprine for at least 12 weeks, but had an inadequate response.

• Had used 6-mercaptopurine or azathioprine, and experienced an adverse drug reaction that made it difficult to continue the treatment.

4) Patients who experienced at least one of the following within 18 months before the starting day of screening period.

• Experienced an exacerbation or relapse of the disease in response to a reduction in the steroid dose, and failed to withdraw from steroid use.

• Had used a steroid, but had an inadequate response (No clinical response to steroids at a dose of at least 1 mg/kg/day or at least 40 mg/day calculated on a prednisolone basis for at least 2 weeks for oral preparations or at least 1 week for intravenous injection).

• Had used steroids and experienced an adverse drug reaction that made it difficult to continue the treatment.

(6) Patients who satisfied any of the following with regard to medication being used at the start of screening period.

1) 6-Mercaptopurine or azathioprine: Patients who had been using the drug for at least 12 weeks before the starting day of screening period, and receiving at a stable dose for at least 4 weeks before the starting day of screening period, or those who had not used the drug for at least 4 weeks before the starting day of screening period.

2) Steroids (oral preparations): Patients who had been using the drug at a stable dose for at least 2 weeks before the starting day of screening period, or had not used the drug for at least 2 weeks before the starting day of screening period.

3) 5-aminosalicylate agents (oral preparation) or salazosulfapyridine agents (oral preparation): Patients who had been using the drug at a stable dose for at least 2 weeks before the starting day of screening period, or had not used the drug for at least 2 weeks before the starting day of screening period.

[Rationale for Setting]

(1)(4)(5) These were established since this study targeted patients with ulcerative colitis who had an inadequate response to existing therapies.

(2) Based on the objectives of this study, since the draft of revision of guideline for treatment of pediatric ulcerative colitis (2008) (Working Group for Preparation of the Guideline for Treatment of Ulcerative Colitis, Japanese Society for Pediatric Gastroenterology, Hepatology and Nutrition) states that a child is defined as a person aged less than 18 years from the viewpoint that children are in the process of growing, the upper limit of age criterion was set as not more than 17 years old. In addition, from an epidemiological viewpoint, patients with ulcerative colitis aged less than 6 years old are considered to be very rare, so the requirement for an age of not less than 6 years old was set.

(3) This criterion was set in order to conduct the clinical trial ethically.

(6) Conditions for using therapeutic drugs commonly used for treatment of ulcerative colitis were made uniform for all patients before the start of efficacy evaluation in order to appropriately evaluate the efficacy of the study drug during the study period.

9.3.2 Exclusion Criteria

Patients who met any of the following exclusion criteria were excluded from the study.

(1) Patients who had total colitis (lesion extending proximal to the splenic flexure) and that satisfied either (1) or (2) below:

1) Patients diagnosed as needing a colectomy at the time of registration.

2) Patients who satisfied at least 4 of the following (a) to (e) at the time of registration.

(a) At least 6 episodes of bloody diarrhea daily

(b) Intense abdominal pain or rebound tenderness

(c) Persistent pyrexia of not less than 37.5°C

(d) Pulse rate: more than 90 beats/min

(e) Hemoglobin: less than 8.5 g/dL

(2) Patients who underwent surgery for ulcerative colitis within 8 weeks before registration, or those for whom surgery for ulcerative colitis was deemed necessary at the time of registration.

(3) Patients who underwent any surgical operation that required caution to prevent postoperative infections within 4 weeks before registration.

(4) Patients who had severe and symptomatic fibrotic stenosis in the large or small intestine.

(5) Patients who had evidence of intestinal stenosis or obstruction, or had intestinal stenosis or obstruction confirmed by enema X-ray, colonoscopy, or sigmoidoscopy within 6 months before registration.

[A stenosis was defined as a narrowing adjacent to which there is a dilation of the proximal intestinal tract on the edema X-ray or a narrowing that an endoscope cannot pass through.]

(6) Patients with a past or current history of fistula.

(7) Patients who had toxic megacolon.

(8) Patients who satisfied any of the following 1) to 4):

1) Patients with current or past dysplasia of the colonic mucosa.

2) Patients with an adenomatous colonic polyp.

3) Patients suffering from total colitis for at least 8 years.

4) Patients suffering from left-sided colitis for at least 10 years (lesion limited distal to the splenic flexure).

However, enrollment was allowed for patients who could be confirmed to have no evidence of dysplasia of the colonic mucosa or adenomatous colonic polyp by colon cancer screening (colonoscopy and biopsy) performed within 1 year before registration of patients who satisfied the above 3) or 4).

(9) Patients who underwent enterostomy.

(10) Patients who underwent extensive colectomy (e.g., post-resection colon is less than 30 cm in length).

(11) Patients who had a lesion limited to the rectum only or in the colon up to 20 cm.

(12) Patients who were previously treated with infliximab or other biological products (such as anti-TNFα agents and anti-IL-6 agents).

(13) Patients who had used immunomodulators (excluding azathioprine, and 6-mercaptopurine methotrexate) such as cyclosporine, tacrolimus (excluding for external use), methotrexate, mycophenolate mofetil and mizoribine within 8 weeks before the starting day of the screening period.

(14) Patients who received cytapheresis within 4 weeks before the starting day of screening period.

(15) Patients who used any of the following drugs/therapies within 1 week before the starting day of the screening period.

1) Total parenteral nutrition

2) Total enteral nutrition [Total enteral nutrition refers to the delivery of total nutrient requirements enterally (nutrient or digest diet nutrient), instead of consumption of a normal diet, low-residue diet, or semidigest diet nutrient, which is followed according to the investigator's (sub investigator's) instructions.]

3) Fasting [Fasting must be used for the purpose of treatment of ulcerative colitis according to the investigator's (sub investigator's) instruction.]

4) Transfusion

5) Steroids (injection/enema/suppository/hemorrhoid ointment)

6) 5-aminosalicylate preparations (enema)

7) Salazosulfapyridine preparations (suppository)

8) Ciprofloxacin or metronidazole used for the treatment of ulcerative colitis

9) Antidiarrheal drugs or drugs for the treatment of diarrhea given to control stool frequency (excluding on-demand based use)

10) Laxatives (excluding those used as pretreatment of sigmoidoscopy).

(16) Patients who were on chronic use of nonsteroidal anti-inflammatory drugs (hereinafter referred to as NSAIDs) (injection/oral/suppository). However, the temporary (a maximum of 4 weeks) use for the purpose of treatment of AEs or use of low-dose aspirin for prevention of heart attack, unstable angina, or transient ischemic attack was allowed.

(17) Patients with infections:

1) Patients complicated with any serious infection (active hepatitis, pneumonia, pyelonephritis, etc.) requiring hospitalization or with a history of these infections within 6 months before registration.

2) Patients complicated with opportunistic infection (cytomegalovirus infection, systemic fungal infection, pneumocystis pneumonia, nontuberculous mycobacterial infection, etc.) or with a history of these infections within 6 months before registration.

3) Patients complicated with active tuberculosis.

4) Patients with a history or a suspected diagnosis of tuberculosis infection. However, this criterion did not apply to use of antituberculosis drugs (isoniazid [INH], in principle) from at least 3 weeks before the scheduled starting day of administration of the study drug.

[Patients corresponding to either of the following conditions are defined as patients with suspected tuberculosis infection.]

• Patients with findings matching old pulmonary tuberculosis (including pleural adhesions or calcification only) by imaging examination.

• Patients exposed to other patients with active tuberculosis.

• Patients who tested positive for QuantiFERON (QFT) test or T-spot.TB test.

In patients whose judgment by QFT test or T-spot.TB test was put on hold or who were tuberculin test-positive (patients suspected of being infected with tuberculosis in consideration of the effect of Bacille de Calmette et Guérin [BCG] vaccination), treatment with antitubercular drugs was not mandatory in the case where investigators (subinvestigators) judged that suspected tuberculosis infection was able to be refuted based on the results of other screening tests for tuberculosis (such as chest X-ray, chest CT scan and medical examination).

Assessment of the results of imaging examinations was performed in consultation with the specialist as needed.

5) Patients with active hepatitis B or C, or patients who have been confirmed to be hepatitis B virus carriers.

6) Patients with confirmed diagnosis of infection with human immunodeficiency virus (HIV).

7) Patients with other chronic infection (chronic renal infection, chronic respiratory infection accompanied by bronchiectasis, chronic sinusitis, etc.).

(18) Patients with a history of hypersensitivity to mouse-derived proteins (murine-, chimera- and humanized antibodies, etc.).

(19) Patients with a complication or history of demyelinating diseases (multiple sclerosis, etc.).

(20) Patients with a complication of congestive cardiac failure.

(21) Patients with a history or a complication of lymphoproliferative disease, including lymphoma, or signs and symptoms suggestive of possible lymphoproliferative disease, such as lymphadenopathy of unusual size or location, or clinically significant hepatomegaly or splenomegaly.

(22) Patients with a family history of lymphoma or leukemia.

(23) Patients with malignant tumor or a history of malignant tumor within 5 years before registration.

(24) Patients with a laboratory test value corresponding to any of the following 10 test items at the time of registration. The test values measured after obtaining informed consent and within 2 weeks before registration.

1) Hemoglobin: <8.0 g/dL

2) WBC count: $<3500\times10^{6}$/L

3) Neutrophil count: $<1500\times10^{6}$/L (total count for stab and segmented cells)

4) Lymphocyte count: $<500\times10^{6}$/L

5) Platelet count: $<10\times10^{4}/\mu L$

6) AST (GOT): exceeding 2 times the upper limit of the reference range

7) ALT (GPT): exceeding 2 times the upper limit of the reference range

8) ALP: exceeding 2 times the upper limit of the reference range

9) HBs antigen, HBs antibody and HBc antibody: Positive

10) HIV antibody: Positive

(25) Patients with a complication of lupus-like syndrome and whose double stranded DNA antibody levels exceeded the upper limit of the laboratory reference range.

(26) Patients with a complication of any significant disease (refer to Grade 3 according to the Criteria for Classification of Seriousness of Adverse Drug Reactions [Appendix 16.1.1a, Attachment 2]).

(27) Patients who had been inoculated with live vaccines within 3 months before the start of administration of the study drug.

(28) Patients who had difficulties in repeated blood collection during the study period.

(29) Patients who did not consent to use contraception during the period from the starting day of study drug administration to 6 months after the last treatment.

(30) Female patients who were pregnant or potentially pregnant, or lactating mothers.

(31) Patients who had participated in another clinical study and had received another study drug within 12 weeks before obtainment of informed consent.

(32) Patients who were judged to be ineligible for the study by the investigator (subinvestigator).

Note) Each period was calculated as described below. The following examples show the minimum unit of year, month or week.

- At least one week before the starting day of the screening period means before the same day of the week during the week before the starting day of the screening period.
- One month before the starting day of the screening period means the same date during the month before the starting day of the screening period.
- One year before the starting day of screening period means the same date during the year before the starting day of screening period.

[Rationale for Setting]

(1) Patients with ulcerative colitis who were systemically ill were excluded to ensure the patient's safety.

(2) Any surgical procedure for ulcerative colitis before the start of the study and during the study affects the efficacy and safety evaluation of TA-650. Therefore, patients who underwent surgery and seemed likely to need surgery were excluded.

(3) (17) This criterion was set because TA-650 may affect immune responses, and may cause increased susceptibility to infections in these patients accordingly.

(4) (5) This criterion was set because patients listed in these criteria seemed likely to need surgery or affect the efficacy evaluation in terms of the number of stools, abdominal pain, etc.

(6) This criterion was set because patients listed in this criterion seemed likely to need surgery, which may affect efficacy or safety evaluation accordingly.

(7) This criterion was set because toxic megacolon is extremely severe and an indication for surgery.

(8) (24) (26) (27) These criteria were set to ensure the patient's safety.

(9) This criterion was set because it was considered difficult to make an appropriate efficacy evaluation in terms of the number of stools and rectal bleeding in patients who underwent enterostomy.

(10) (11) These criteria were set because it was considered inappropriate to assess TA-650 in patients with localized lesions.

(12) This criterion was set because the safety and efficacy in this study may be biased by the previous clinical evaluation of infliximab, the identical component as the study drug, etc. and the serum TA-650 concentration and ATI may also be affected. In addition, this criterion was set because other biological products may similarly affect the evaluation of safety and efficacy.

(13) to (16) These criteria were set because it was considered that the efficacy evaluation of TA-650 would be affected.

(18) This criterion was set because a hypersensitive reaction due to TA-650 administration may occur in these patients.

(19) (20) These criteria were set because TA-650 may induce relapse or aggravation of symptoms.

(21) to (23) These criteria were set because TA-650 may affect the immune response and therefore administration of TA-650 might increase the risk of development of malignant tumors such as malignant lymphoma.

(25) This criterion was set because TA-650 administration may cause symptoms that suggest lupus-like syndrome in these patients.

(28) This criterion was set because assessment of the study drug would be difficult.

(29) (30) These criteria were set because the safety of TA-650 with regard to reproductive and developmental toxicity has not been established in humans.

(31) This criterion was set in order to conduct this study in an ethical manner. In addition, it was set because the effects of drugs which have not been well established are unpredictable in terms of their efficacy and safety.

(32) This criterion was set in order to conduct this study in a safe and ethical manner.

9.3.3 Removal of Patients from Therapy or Assessment

(1) Discontinuation criteria for patients

If any of the following discontinuation criteria applied, treatment was discontinued.

1) Upon request from the legal representative or the patient to discontinue participation in the study.

2) When the investigator (subinvestigator) considered it inappropriate to continue the study for the relevant patient due to an exacerbation of ulcerative colitis (for example, use of prohibited concomitant drugs/therapies, excluding incision for drainage).

3) When the investigator (subinvestigator) considered it difficult to continue the study for the relevant patient due to AEs. If a lupus-like syndrome occurred, and the patient was found to be positive for double stranded DNA antibodies (double stranded DNA IgM antibody titer of ≥6 U/mL or double stranded DNA IgG antibody titer of >12 IU/mL), the study for the relevant patient was to be discontinued.
4) When the patient was found to be apparently ineligible for the study from the standpoint of safety assurance or efficacy evaluation after the start of administration of the study drug.
5) Other situations where the investigator (subinvestigator) considered it necessary to discontinue the study.

[Rationale for Setting]

These criteria were set in order to conduct the study in an ethical manner and to also take the patients' safety into consideration.

(2) Procedure for discontinuation of the study

When the study was discontinued during the evaluation period, the investigator (subinvestigator) took appropriate action for the relevant patient, and immediately notified the monitor of the matter. The investigator (subinvestigator) performed examinations and observations specified at the time of discontinuation and 8 weeks after the last treatment.

The investigator (subinvestigator) documented the day of discontinuation, reason for discontinuation and the details, circumstances that leading to discontinuation, and subsequent course after discontinuation in the CRF. When the study was discontinued due to AEs, the investigator (subinvestigator) documented AE terms leading to discontinuation in the discontinuation page in the CRF. The day of discontinuation was defined as the day when assessment at the time of discontinuation was performed (day when medical examination for assessment at the time of discontinuation was performed). When the assessment at the time of discontinuation was not able to be performed, the day when discontinuation was decided was referred to as the day of discontinuation.

The investigator (subinvestigator) followed patients for whom the specified observations/examinations were not able to be performed within 8 weeks after the last treatment or those who failed to visit the hospital after discontinuation by means of mail (sealed letter) or telephone etc. to obtain the information such as the reason and their subsequent courses, and documented the result in the discontinuation page in the CRF. The investigator (subinvestigator) were to collect Symptom Assessment Sheets from patients who did not visit the medical institutions as much as possible through mail or other method.

The investigator (subinvestigator) or the study collaborator collected the Symptom Assessment Sheet from patients who failed to visit the study site by means of mail etc. as much as possible.

(3) Discontinuation or suspension of the study

1) Criteria for discontinuation or suspension of the study

In the following cases, the sponsor examined the appropriateness of continuing the study in the entire or a part of medical institutions.

(a) When the sponsor obtained information related to the quality, efficacy, or safety of the study drug, or other important information for proper conduct of the study.

(b) When the sponsor was advised to terminate the study by the safety evaluation committee.

(c) When any revision to the protocol became necessary, but the medical institution was not able to deal with it.

(d) When any correction to the protocol, etc. was proposed by the director of the medical institution based on the opinions of the IRB, but the sponsor was not able to accept it.

(e) When discontinuation of the study was instructed by the director of the medical institution based on the decision of the IRB of the medical institution.

(f) When the medical institution seriously or persistently violated GCP, the protocol, or the study contract.

2) Discontinuation or suspension of the entire study based on the decision of the sponsor

Upon decision of discontinuation or suspension of the entire study, the sponsor was to immediately notify the director of the medical institution as well as regulatory authorities in writing of the matter and the reason for termination or suspension. Upon receipt of notification of discontinuation or suspension of the study from the sponsor, the director of the medical institution was to immediately notify the investigator and the IRB in writing of the matter and the details of the reason.

Upon receipt of notification of termination or suspension of the study from the sponsor through the director of the institution, the investigator was to immediately notify patients of the matter, and ensure appropriate treatment and other procedures for patients.

Handling of patients at discontinuation of the study should be performed according to "(2) Procedure for discontinuation of the study."

3) Discontinuation or suspension of the study at the relevant medical institution based on the decision of the investigator or the IRB

If the investigator decided discontinuation or suspension of the study at that investigator's own discretion, the investigator was to immediately notify the director of the medical institution in writing of the matter and the details of the reason. The director of the institution was to immediately notify the sponsor and the IRB of the matter in writing. If the IRB decided upon termination or suspension of the study at its own discretion, it was to immediately notify the director of the medical institution of the matter in writing. The director of the institution was to immediately notify the investigator and the sponsor of the matter in writing.

4) Discontinuation of the study based on cancellation of the contract with the medical institution

If the sponsor discontinued the study because the medical institution seriously or persistently violated GCP, the protocol, or the study contract during the study period, the sponsor was to immediately report the matter to regulatory authorities.

9.4 Treatments

9.4.1 Treatments

9.4.1.1 Dosage Regimen and Route of Administration of the Study Drug

TA-650 was administered at a dose of 5mg/kg based on the body weight of patient on each administration day by intravenous drip infusion over at least a 2-hr period. The study drug was administered at the initial treatment (Week 0) and Weeks 2 and 6, and CAI score-based responders based on determination of CAI score at Week 8 were further given the study drug at Weeks 14 and 22 in a similar manner. However, CAI score-based non-responders did not receive the study drug after Week 14.

9.4.1.2 Method of Preparation

The dose required for administration was calculated based on the patient's body weight (by rounding off to the whole number) on the day of administration, and diluted with approximately 250 mL of physiological saline. The dissolution of the study drug was performed before use, and treatment was started within 3 hours after dissolution. To patients whose body weight on the day of administration was less than 20 kg, the study drug was diluted with approximately 100 mL of physiological saline and administered.

9.4.1.3 Packaging and Labeling of the Study Drug

(1) Packaging

A vial with the attached surface label was packed into a box as shown below.

(2) Labeling

1) Labeling on the vial

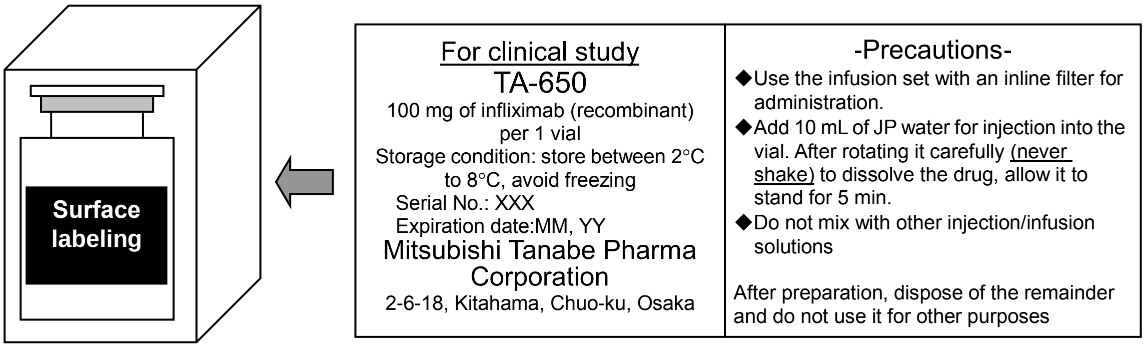

2) Labeling of the package

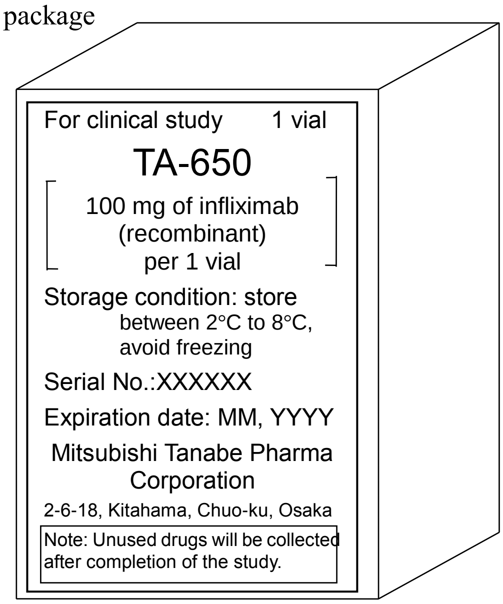

9.4.2 Identity of Study Drug

9.4.2.1 Name of the Study Drug

Name: TA-650

Non-proprietary name: Infliximab (recombinant)

9.4.2.2 Dosage Form and Content

TA-650 100 mg vial:

A vial containing 100 mg of infliximab supplied as a lyophilized product. It is an injection used by dissolving in water for injection (JP grade) and diluting with physiological saline (JP grade) before use.

9.4.2.3 Manufacturer and Batch Number

TA-650 100 mg vial: 110001, 120075 (batch number)

The study drug was obtained from Janssen Biotech, Inc.

9.4.2.4 Study Drug Management

The sponsor supplied the study drug to medical institutions after first concluding a clinical trial contract with the institutions. The study drug manager stored and managed the study drug according to the “Procedure for Study Drug Management” (Appendix 16.1.13a) specified by the sponsor. After completion of the study, the unused study drug, the study drug which was prescribed but returned before preparation, and empty boxes were returned to the monitor. The study drug was not used for purposes other than those specified in the protocol (such as other clinical studies, animal experiments and basic experiments).

9.4.3 Method of Assigning Patients to Treatment Groups

9.4.3.1 Allocation of the Study Drug

Not applicable because this is an uncontrolled study.

9.4.3.2 Registration of Patients

Patients were registered into the study in accordance with the procedures shown in the method for registration of patients “Flow chart of study procedures (Appendix 16.1.1a, Attachment 3)” and the following descriptions. The registration date was defined as the day when eligibility confirmation was completed.

After obtaining informed consent, the investigator (subinvestigator) completed the “Contact form for informed consent obtainment (Appendix 16.1.1a, Attachment 4)” and faxed it to the sponsor.

After obtaining informed consent, the investigator (subinvestigator) confirmed the eligibility of the patient by observations and examinations, and then filled in the “Registration Form (Appendix 16.1.1a, Attachment 5),” and faxed it to the sponsor. In the case where the period from CAI score evaluation day in the screening period to the initial administration of the study drug exceeded one week, the patient was to be re-registered after re-evaluation of the CAI score. At the time of this re-evaluation, the partial Mayo score and PUCAI score were also determined, and sigmoidoscopy was to be performed as much as possible.

9.4.4 Selection of Doses in the Study

TA-650 was administered at a dose of 5mg/kg based on the body weight of the patient on each day of administration by intravenous drip infusion over at least a 2-hr period. The study drug was administered at the initial treatment (Week 0) and Weeks 2 and 6, and CAI score-based responders based on

determination of CAI score at Week 8 were further given the study drug at Weeks 14 and 22 in a similar manner. However, CAI score-based non-responders did not receive the study drug after Week 14.

[Rationale for Setting]

In Japan, administration of TA-650 at a dose of 5 mg/kg at Weeks 0, 2 and 6 followed by administration at 8-week intervals has been approved for treatment of patients with moderate to severe ulcerative colitis who had insufficient response to the existing therapies. In foreign countries, based on the results of the C0168T72 study conducted on pediatric patients with ulcerative colitis and the ACTI study conducted in adult patients, it has been suggested that the efficacy and safety profiles and pharmacokinetics of the study drug in children are similar to those in adults. Consequently, in the US and Europe, administration of TA-650 at a dose of 5 mg/kg at Weeks 0, 2 and 6 followed by administration at 8-week intervals has been approved for treatment of moderate to severe active ulcerative colitis in pediatric patients, similarly to the dosage regimen for adults. Based on this approval, it was decided to administer TA-650 at a dose of 5 mg/kg at Weeks 0, 2 and 6, followed by administration at Weeks 14 and 22.

9.4.5 Selection and Timing of Dose for Each Patient

All of the patients were given TA-650 at a dose of 5 mg/kg. The timing of treatment for each patient is described in 9.4.1.1 Dosage Regimen and Route of Administration of the Study Drug.

9.4.6 Blinding

Not applicable because this is an open label study.

9.4.7 Prior and Concomitant Therapy

9.4.7.1 Prohibited Concomitant Drugs and Therapies

Use of the drugs and therapies listed in the following (1) to (15) was prohibited throughout the screening period and the efficacy evaluation period, and use of those listed in (16) and (17) was prohibited throughout the screening period and the evaluation period.

(1) Remicade®, or other biological products (such as anti-TNFα agents, anti-IL-6 agents).

(2) Immunomodulators (excluding azathioprine and 6-mercaptopurine) such as cyclosporine, tacrolimus (except for external preparation), methotrexate, mycophenolate mofetil and mizoribine.

(3) Steroids (injection, enema, suppository and hemorrhoid ointment).

Steroid injection was allowed for the purpose of treatment of AEs such as infusion reactions or pretreatment. However, pretreatment with steroids was allowed only after completion of the efficacy assessment.

(4) Salazosulfapyridine preparations (suppository)

(5) 5-aminosalicyate preparations (enema)

(6) Antibacterial agents for ulcerative colitis (ciprofloxacin and metronidazole)

The temporary use (a maximum of 3 weeks) was allowed for purposes other than the treatment of ulcerative colitis, e.g., treatment of infections.

(7) Laxatives
The use of laxatives was allowed as a pretreatment for sigmoidoscopy, colonoscopy, and other tests/examinations.

(8) Antidiarrheal drugs and drugs for the treatment of diarrhea

The temporary (not more than approximately 3 consecutive days) use was allowed in an unavoidable case, but use of these drugs were prohibited during the period from 3 days before each starting day of CAI score evaluation to the evaluation day.

(9) NSAIDs (injection, oral preparation and suppository)

The temporary (a maximum of 4 weeks) use for the purpose of treatment of AEs or use of low-dose aspirin for prevention of heart attack, unstable angina, or transient ischemic attack was allowed.

(10) Total parenteral nutrition

(11) Total enteral nutrition

Total enteral nutrition refers to the delivery of total nutrient requirements enterally (nutrient or digest diet nutrient), instead of consumption of normal diet, low-residue diet, or semidigest diet nutrient, which must be used according to the investigator’s (sub investigator’s) instructions.

(12) Fasting

Fasting must be used for the purpose of treatment of ulcerative colitis according to the investigator’s (sub investigator’s) instruction.

(13) Cytapheresis

(14) Surgery

1) Surgery for ulcerative colitis

2) Other surgeries that require caution against postoperative infections

(15) Transfusion

(16) Live vaccines

(17) Investigational products other than TA-650

[Rationale for Setting]

(1) This criterion was set as administration of infliximab which is the same component as the study drug and which might result in failure to evaluate the efficacy, safety and pharmacokinetics in this study. Concomitant use of other biological products was also prohibited since it might similarly affect the evaluation of efficacy and safety of the study drug.

(2) to (6) Use of these drugs and therapies was prohibited because it was deemed difficult to appropriately assess the efficacy in the presence of these drugs or therapies. However, temporary

use of ciprofloxacin or metronidazole for the purpose of treatment of AEs was allowed, taking the patient's safety into consideration. In addition, temporary use of steroid injection in patients who experienced AEs such as infusion reactions was allowed in consideration of the safety of patients.

(7), (8) Use of these drugs was prohibited because these drugs could affect the number of stools, which is a subscore of CAI score, partial Mayo score, Mayo score and PUCAI score. However, taking the actual treatment practice into consideration, temporary use of these drugs was allowed as a pretreatment for tests/examinations or in an unavoidable case.

(9) Since it has been reported that these drugs may exacerbate ulcerative colitis, use of these drugs was in principle prohibited due to the possibility of affecting the efficacy evaluation of the study drug. However, the temporary use for the purpose of treatment of AEs or use of low-dose aspirin for prevention of transient ischemic attack was allowed, taking the patient's safety into consideration.

(10) to (14)1), (15) Use of these drugs and therapies was prohibited because it was deemed difficult to appropriately assess the efficacy in the presence of these drugs or therapies.

(14)2) Use of the therapy was prohibited because potential postoperative infections were of concern and these might affect an efficacy evaluation of the study drug.

(16) Concomitant use of this therapy was prohibited because the response to vaccinations was unknown, and possibility of secondary infection with live vaccines could not be ruled out.

(17) Concomitant use of these drugs was prohibited because their effects on efficacy and safety were unknown.

9.4.7.2 Restricted Concomitant Drugs and Therapies

Medicines and therapies not included in "9.4.7.1 Prohibited Concomitant Drugs and Therapies" were permissible throughout the screening period and the evaluation period. However, use of steroids (oral preparations), azathioprine, 6-mercaptopurine, 5-aminosalicylate preparations (oral preparations) and salazosulfapyridine preparations (oral preparations) was only permitted under the following conditions.

(1) Steroids (oral preparations)

Use of steroids (oral preparations) was allowed under the condition that the dose remained stable in principle throughout the screening period and the efficacy evaluation period and additional usage or a dose increase was prohibited. However, reducing the dose in an unavoidable medical situation was allowed.

After the evaluation on the starting day of study drug administration, reducing the dose was allowed when a clinical improvement in the symptoms of ulcerative colitis was observed by the investigator (subinvestigator). The steroid (oral preparations) dose should be carefully reduced within the following reduction range at a maximum, taking the clinical findings and the patient's progress into due consideration.

[Reduction in steroid (oral preparation) dose]

Not less than 20 mg/day calculated on a prednisolone basis: Reduction by 10 mg/week calculated on a prednisolone basis at a maximum.

Not less than 10 mg/day and less than 20 mg/day calculated on a prednisolone basis: Reduction by 5 mg/week calculated on a prednisolone basis at a maximum.

Less than 10 mg/day calculated on prednisolone basis: Reduction by 2.5 mg/week calculated on prednisolone basis at a maximum.

If the patient experienced an exacerbation of the symptoms in response to a reduction in the dose or discontinuation of use of steroids (oral preparations), the temporary (a maximum of 4 weeks) use of the relevant steroid at a dose exceeding the dose used during the screening period was allowed to improve the symptoms. In such a case, the daily dose had to be reduced to the dose used during the screening period or lower, within 4 weeks from the dose change.

The temporary (a maximum of 4 weeks) use of steroids at a dose exceeding the dose used during the screening period was also allowed in unavoidable medical situations other than the case for treatment of the primary disease.

(2) Azathioprine, 6-mercaptopurine, 5-aminosalicylate preparations (oral preparations) and salazosulfapyridine preparations (oral preparations)

Use of these drugs was allowed in principle, only when used at a stable dose throughout the screening period and the efficacy evaluation period; additional use or dose increase was prohibited. However, after the evaluation on the starting day of study drug administration, reducing the dose was allowed only when it became necessary to reduce the dose for other reasons than the primary disease (AEs or other medical reasons). In such a case, the dose should be stable throughout the efficacy evaluation period after dose reduction or should not exceed the dose used during the screening period.

[Rationale for Setting]

The use of these drugs was allowed when used for a specified period at a constant dose before the start of efficacy evaluation because the use of these drugs under said conditions was considered unlikely to affect the efficacy evaluation of TA-650.

Steroid therapy is known to involve many ADRs, and to the steroid dose is preferably reduced as much as possible when clinical symptoms have improved. Consequently, it was decided to evaluate the steroid dose as one of the efficacy indices, and reduction of the dose or discontinuation of steroids (oral preparations) due to an improvement in the symptoms of the primary disease was allowed. The temporary increase in the dose was also allowed when patients experienced an exacerbation of the symptoms after dose reduction.

9.4.7.3 Precautions for Other Concomitant Drugs

(1) Vaccination

Due to potential risk of secondary infection caused by live vaccination during TA-650 treatment period, a criterion to exclude patients who had received live vaccine within 3 months before the start of TA-650 administration from the study was established, and live vaccination during the evaluation period was prohibited. In the case of vaccination with any inactivated vaccine during TA-650 treatment period, vaccination was to be given in consideration of an unknown response of TA-650 to vaccination.

Based on these, a patient's history of routine/voluntary vaccination and the past history of infections preventable by vaccination were checked before registration, and the necessity of vaccination in the patient was considered.

(2) Treatment for tuberculosis prevention

Patients who had a history of tuberculosis or who were suspected of having tuberculosis infection were given INH throughout the evaluation period from at least 3 weeks before the starting day of study drug administration. The dose was set at 10 to 15 mg/kg with a maximum of 300 mg/day as a guide. If the use of INH was discontinued due to ADRs etc. within 3 weeks before the start of study drug administration, the study drug was not administered to the relevant patient. If INH could not be used due to ADRs etc. after the start of study drug administration, then appropriate actions were to be taken in consultation with the specialist.

[Patients corresponding to either of the following conditions are defined as patients with suspected tuberculosis infection.]

- Patients with findings matching prior pulmonary tuberculosis (including pleural adhesions or calcification only) by imaging examination.
- Patients exposed to other patients with active tuberculosis.
- Patients who were positive for the QFT test or T-spot.TB test.

In patients whose judgment by the QFT test or T-spot.TB test was put on hold or who were tuberculin test-positive (patients suspected of being infected with tuberculosis in consideration of the effect of BCG vaccination), treatment with antitubercular drugs was not mandatory in the case where investigators (subinvestigators) judged that suspected tuberculosis infection could be refuted based on the results from other screening tests for tuberculosis (such as chest X-rays, chest CT scans and medical examinations).

Assessment of the results of imaging examinations was performed in consultation with the specialist as needed.

9.4.7.4 Descriptions of Concomitant Drugs and Therapies

The investigators (subinvestigators) documented the following information about the concomitant drugs and therapies used during the period from the screening period to the end of the efficacy evaluation period in the page for concomitant drugs and therapies in the CRF.

(1) Concomitant drug: name of the drug, reasons for use, reasons for dose change, daily dose, route of administration, treatment period

(2) Treatment and therapies for the purpose of treatment of ulcerative colitis: treatment and therapy, date of start, date of completion

Information about the following drugs did not require a description in the CRF.

- Physiological saline etc. used to dissolve injection products.
- Physiological saline and water for injection etc. used as a fluid replacement for infusion.

- Perioperative drugs used in surgery for treatment of AEs (anesthetics, infusion, physiological saline solution, disinfectants, etc.). However, information about the prohibited concomitant drugs and restricted concomitant drugs was recorded in the CRF.

The study collaborator was allowed to transcribe the data to the CRF only when the relevant data were recorded in the source documents.

9.4.8 Treatment Compliance

(1) Daily life guidance

The investigator (subinvestigator) or the study collaborator provided legal representatives and patients with daily life guidance with attention to the following points.

1) Patients should visit the medical institution and undergo medical examination and tests on the designated days. Patients who could not visit the medical institution on the designated days should make sure to contact the investigator (subinvestigator) or the study collaborator and follow their instructions.

2) Patients should carry and show their study participation card when they visit other hospitals and departments. Patients should inform the investigator (subinvestigator) or the study collaborator of the use of any drug prescribed by a physician not involved in this study or over-the-counter (OTC) drugs purchased at a pharmacy. Patients should make sure to inform the investigator (subinvestigator) or the study collaborator in advance about use of any additional drugs and vaccinations including influenza vaccines (special attention should be paid to prohibition of concomitant use of live vaccine) during study.

3) Patients should maintain their lifestyle (especially dietary habit and health food) to the extent possible.

4) Patients should immediately inform the investigator (subinvestigator) or the study collaborator of any physical abnormality such as a sign of infection (even a slight cold) , pregnancy, or clear exacerbation of ulcerative colitis symptoms (including frequency of stools, blood in stools, abdominal pain etc.), and ask them if a visit to the hospital is necessary.

5) Patients should use contraception during the period from registration to 6 months after the last treatment.

(2) Instruction on how to complete the Symptom Assessment Sheet

After obtainment of informed consent, the investigator (subinvestigator) or the study collaborator gave the Symptom Assessment Sheet to the patient or legal representative, explained how to complete the sheet and instructed them to fill out the sheet in regards to the following items every day for 7 days before the designated visit days, and make sure to bring it on the visit days.

1) Items entered in the Symptom Assessment Sheet

   - Number of stools per day
   - Blood in stools
   - Presence/absence of use of drugs for the treatment of constipation or diarrhea

2) How to complete the Symptom Assessment Sheet

- To fill out the sheet every day for 7 days before the designated visit days.
- To fill out the sheet using writing tools such as a ballpoint pen whose markings are not easily erased.
- To change or correct the description by crossing out with double lines so that the entry prior to correction is legible.
- Not to write in the spaces for medical institution use.
- To count evidence of blood even without stools as the number of stools.
- Not to count among the number of stools, an episode where the patient had an urgent desire to evacuate a stool but could not defecate.
- In terms of tenesmus and incomplete defecation, an episode such that the patient defecated several small stools in a very short term should be counted as one defecation.

9.5 Efficacy and Safety Variables

9.5.1 Efficacy and Safety Endpoints and Flow Chart

9.5.1.1 Observation, Examination and Investigation Items and Time Points (Schedule)

This study was conducted in accordance with the following flow chart and Table 9.5–1.

Flow chart for study procedures

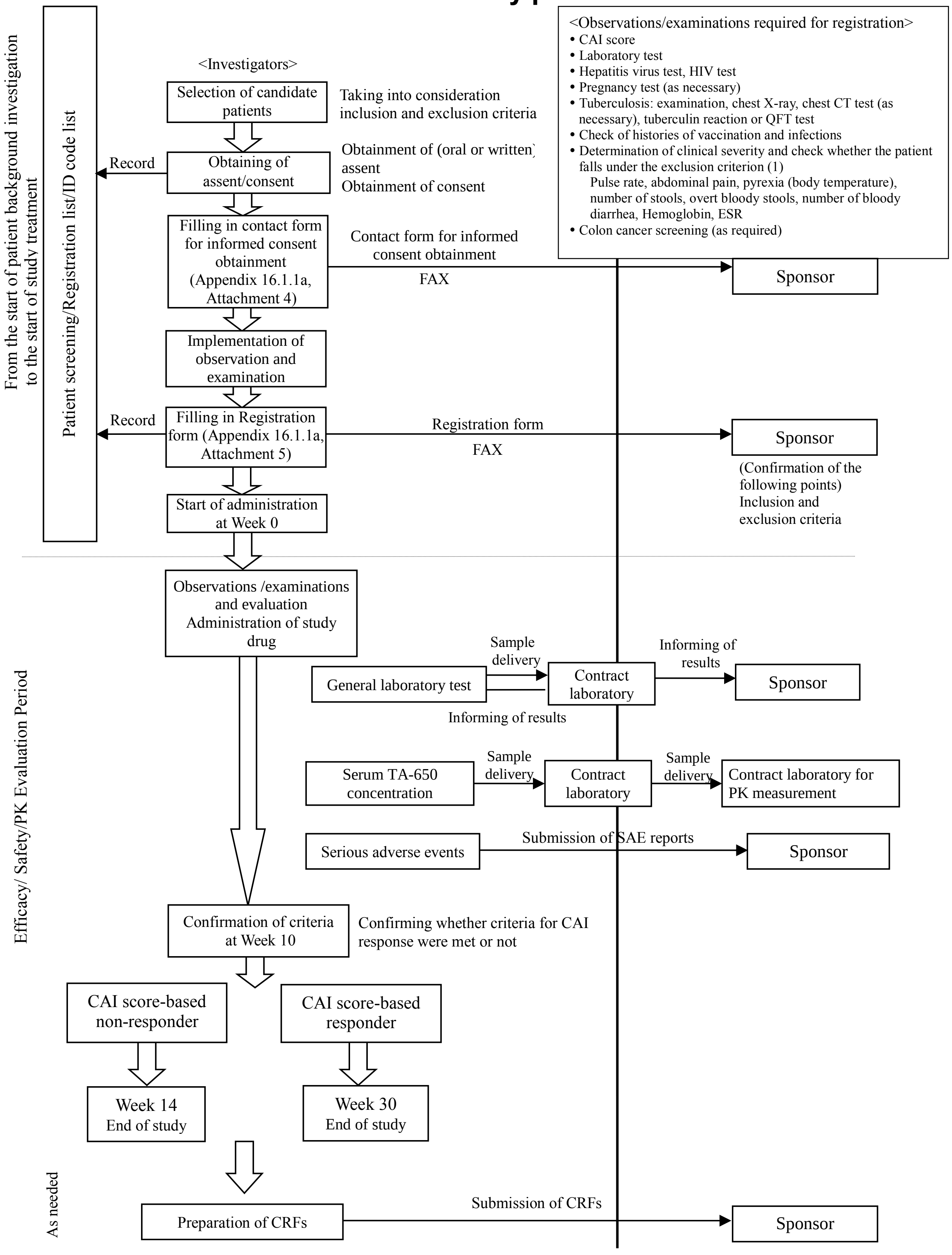

Prior to registration, the investigator (subinvestigator) performed the following examinations and observations to confirm the eligibility of the patients.

(1) Laboratory tests

The investigator (subinvestigator) measured the following laboratory parameters within 2 weeks before registration after obtaining of informed consent. When there were multiple laboratory test values, the value obtained on the closest day to the registration day was used, except for the values obtained by the centralized laboratory tests performed at the start of administration of the study drug.

Hemoglobin, WBC count, neutrophil count*, lymphocyte count, platelet count, AST (GOT), ALT (GPT), ALP, HBs antigen, HBs antibody, HBc antibody, HIV antibody and pregnancy test (only for female patients of physiologically childbearing potential)

* Neutrophil count: The total count of stab and segmented cells was used, if applicable.

(2) Tuberculosis testing

The investigator (subinvestigator) performed a medical interview, chest X-ray, chest CT scan (as needed), and tuberculin skin test or QFT test (T-spot.TB test may be used) within 4 weeks before registration after obtaining informed consent and confirming the presence or absence of respiratory infection including tuberculosis. If patients underwent multiple examinations, the data obtained on the closest day to the registration day was used.

The investigator (subinvestigator) administered antituberculosis drugs (INH, in principle) to patients who were found to have a history of tuberculosis infection or were suspected of having tuberculosis infection at least 3 weeks before the starting day of study drug administration.

[Patients corresponding to either of the following conditions are defined as patients with suspected tuberculosis infection.]

• Patients with findings matching old pulmonary tuberculosis (including pleural adhesions or calcification only) by imaging examination

• Patients exposed to other patients with active tuberculosis

• Patients who were positive for QFT test or T-spot.TB test

In patients whose judgment was made by QFT test or T-spot.TB test was put on hold or who were tuberculin test-positive (patients suspected of being infected with tuberculosis in consideration of the effect of BCG vaccination), treatment with antitubercular drugs was not mandatory in the case where investigators (subinvestigators) judged that suspected tuberculosis infection could be refuted based on the results of other screening tests for tuberculosis (such as chest X-ray, chest CT scan and medical examination).

Assessment of the results of imaging examinations was performed in consultation with the specialist as needed.

(3) Vaccination

The investigator (subinvestigator) checked a patient’s history of routine/voluntary vaccination and the past history of infections preventable by vaccination before registration, and considered the necessity for vaccination in the patient. Check of a patient’s history of vaccination and the past history of infections was performed by the registration day at the latest.

(4) Confirmation of clinical severity and whether the patient falls under the exclusion criterion (1)

The investigator (subinvestigator) checked the pulse rate, hemoglobin, ESR, number of stools, overt bloody stools, number of bloody diarrhea, pyrexia (body temperature), and abdominal pain at the time of registration, and confirmed that the patient did not fall under 9.3.2 Exclusion criteria (1) and determined the clinical severity of the primary disease based on these sets of information.

(5) Colon cancer screening

When the patient satisfied either of the following, the investigator (subinvestigator) was required to perform colon cancer screening (colonoscopy and biopsy) after obtaining of informed consent and obtaining consent by 4 days before the start of the screening period, and confirmed that there was no evidence of dysplasia of the colonic mucosa or adenomatous colonic polyp.

- Patients who had been suffering from total colitis for at least 8 years, and had not undergone colon cancer screening within 1 year before registration.
- Patients who had been suffering from left-sided colitis for at least 10 years, and had not undergone colon cancer screening within 1 year before registration.

A colonoscopy performed as the colon cancer screening could substitute for a sigmoidoscopy performed to determine the Mayo score.

Table 9.5–1 Study Schedule

		Informed consent	Screening period	Evaluation period										Non-responder		Discontinued patient	
				Week 0	Week 2	Week 6	Week 8	Week 10	Week 14	Week 18	Week 22	Week 26	Week 30	Week 10	Week 14	Day of discontinuation	8 weeks after last administration
Permissible time range for evaluation day (day)		–		–	±3	±3	±3	±7	±7	±7	±7	±7	±7	±7	±7	—	±7
Obtaining of informed consent		●															
Observations necessary at registration*1			●														
Background investigation			●														
Permissible time range for study drug administration (day)				–	±3	±3			±7		±7						
Study drug administration				●	●	●			●		●						
Height and body weight				●	●	●	●	●	●	●	●	●	●			●	
Efficacy	CAI score*2		●		●	●	●	●	●	●	●	●	●			●[a]	
	Partial Mayo score*3		●		●	●	●	●	●	●	●	●	●			●[a]	
	Sigmoidoscopy*4		●[b]										●[b,d]			●[a,b,d]	
	PUCAI score*5		●		●	●	●	●	●	●	●	●	●			●[a]	
Safety	Subjective symptoms and objective findings			←	—	—	—	—	—	—	—	—	—	—	—	—	→
	Blood pressure, pulse rate, and body temperature*6			●	●	●			●		●						
	General laboratory tests*7			●	●	●	●	●	●	●	●	●	●	●	●	●	●
	Immunoserological test*8			●					●				●		●	●	●
TNFα, IL-6*9				●													
CRP*10				●	●	●	●	●	●	●	●	●	●			●[a]	
Chest X ray, Chest CT scan (as needed)			●						●				●		●	●	
PK	Serum infliximab concentration*11			●[c]	●	●	●	●	●[c]	●	●[c]	●	●	●	●	●	●
	Antibodies to infliximab			●					●		●		●		●	●	●

When administration of the study drug was scheduled on the evaluation day, evaluation and examinations were performed before study drug administration.

a: These evaluations were not necessary when a CAI score-based non-responder discontinued the study after the evaluation at Week 8.

b: Performed as far as possible. However, when it was impossible to perform this examination during the screening period (in principle, performed after registration and by the start of the study drug administration. in unavoidable cases, it was allowed to perform the examination within 2 weeks from the starting day of study drug administration and by 4 days before the start of the screening period), it was not necessary to perform the examination at Week 30 or at the time of discontinuation.

c: Blood was collected twice in total, immediately before administration and 1 hour after completion of administration of TA-650.

d: The permissible time range for sigmoidoscopy was set as the range from -13 to +14 days.

(Example) Observation/examination items necessary at the time of registration and until the start of study drug administration (in the case where CAI score was determined on the registration day.)

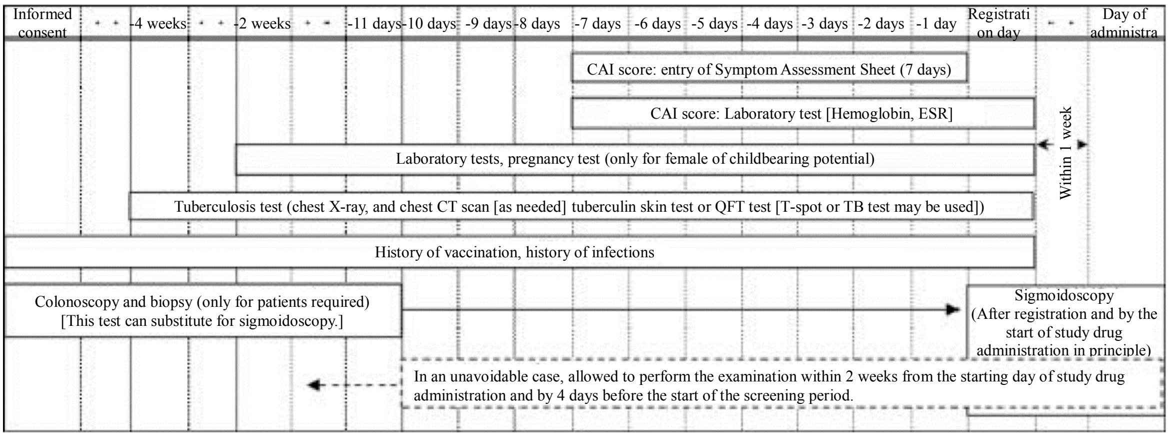

*1 Observation/examination items necessary at registration:

Activity of ulcerative colitis	CAI score
Laboratory tests	[Performed after obtaining of informed consent and within 2 weeks before registration]<br>Hemoglobin, WBC count, neutrophil count*, lymphocyte count, platelet count, AST (GOT), ALT (GPT), ALP, HBs antigen, HBs antibody, HBc antibody, HIV antibody and pregnancy test (only for female patients of physiologically childbearing potential)<br>*Neutrophil count: The total count of stab and segmented cells was used, if applicable.
Tuberculosis	[Performed after obtaining of informed consent and within 4 weeks before registration]<br>Medical interview, chest X-ray, chest CT scan (as needed), and tuberculin skin test or QFT test (T-spot.TB test may be used)
History of vaccination<br>Past history of infections	[Checked before registration]<br>Check of a history of vaccination and a past history of infections.
Determination of clinical severity and check whether or not the patient falls under the exclusion criterion (1)	[Assessed on the day of registration based on the following examinations/observations]<br>Pulse rate, abdominal pain, pyrexia (body temperature), number of stools, overt bloody stools, number of bloody diarrhea, hemoglobin, and ESR
Colon cancer screening (only for patients deemed necessary)	[Performed after obtaining of informed consent and by 4 days before the start of the screening period]<br>Colonoscopy and biopsy

- The study treatment was started within one week from the CAI evaluation day in the screening period. In the case where the period from CAI evaluation day in the screening period to the initial administration of the study drug exceeded one week, the patient was to be re-registered after re-evaluation of the CAI score of the relevant patient.
- When there were multiple laboratory test values, the value obtained on the closest day to the registration day was used, except for the values obtained by the centralized laboratory tests performed at the start of study drug administration.
- If chest X-ray, chest CT scan (as needed), tuberculin skin test, QFT test or T-spot.TB test were performed several times after obtaining consent, the data obtained on the closest day to the registration day were used.
- Either tuberculin skin test or QFT test (T-spot.TB test may be used) had to be performed. The results of tuberculin skin test were judged in consideration of the effect of BCG vaccination.
- Colon cancer screening was performed only for patients deemed necessary according to the exclusion criterion (8). A colonoscopy performed as colon cancer screening could substitute for sigmoidoscopy performed to assess the Mayo score.

*2 Examinations and observations necessary for determination of CAI scores:

Examinations (such as laboratory tests)	[Performed on the evaluation day] Body temperature<br>[Performed during the period from 7 days before the evaluation day to the evaluation day] Hemoglobin (measured in the institution), ESR (measured in the institution)
Clinical symptoms (observations/medial interview)	Investigator's global assessment of symptomatic state, abdominal pain/cramps, extraintestinal manifestations
Symptom Assessment Sheet (filled out by patients or legal representatives)	Number of stools per week, blood in stools (based on weekly average)
• For hemoglobin and ESR, the measurement values obtained in the medical institution during the period from 7 days before the evaluation day to the evaluation day were used.<br>• When administration of the study drug was scheduled on the evaluation day, evaluation and examinations were performed before administration of the study drug for the relevant week.<br>• When there were several hemoglobin and ESR data during the permissible time windows, the value obtained on the closest day to the CAI evaluation day was used.	

*3, *4 Examinations and observations necessary for determination of partial Mayo scores and Mayo scores:

Clinical symptoms (observations/medial interview)	Investigator's global assessment
Symptom Assessment Sheet (filled out by patients or legal representatives)	Number of stools, rectal bleeding
Sigmoidoscopy	Endoscopy (only for Mayo scores)
• Sigmoidoscopy was used for determination of Mayo scores.<br>• Sigmoidoscopy in the screening period was performed in principle, after registration and by the start of the study drug administration. In an unavoidable case, it was allowed to perform the examination within 2 weeks from the starting day of study drug administration and by 4 days before the start of the evaluation period.<br>• The permissible time range for sigmoidoscopy at Week 30 was set as between -13 days and +14 days. However, in consideration of the effect on the efficacy evaluation at Week 30, conducting sigmoidoscopy during the evaluation period for the CAI score based on Symptom Assessment Sheet was avoided as much as possible.	

*5 Observations necessary for determination of PUCAI scores:
The investigator obtained information from the patients about 6 subscores including abdominal pain, rectal bleeding, stool consistency of most stools, number of stools per 24 hours, nocturnal stools and activity level.

*6 Blood pressure, pulse rate and body temperature:
These parameters were measured immediately before the start of administration, every 30 minutes during administration, and also every 30 minutes for 2 hours after completion of administration. When there was at least a 15-minute interval between completion of administration and the last measurement before completion of administration, measurement was also performed at the time of completion of administration.

*7 General laboratory tests: Measurement of laboratory parameters was performed at the contract laboratory.

Hematology	Red blood cell count, hemoglobin, hematocrit, WBC count, differential count of WBC (neutrophils, eosinophils, basophils, monocytes and lymphocytes), and platelet count
Blood biochemistry	AST (GOT), ALT (GPT), ALP, LDH, γ-GTP, total protein, albumin, total cholesterol, total bilirubin, BUN, serum creatinine, and serum electrolyte (Na, K, Cl)
Urinalysis (qualitative)	Protein, glucose, occult blood, and urobilinogen

*8 Immunoserological test: Centralized measurement of antinuclear antibodies and double stranded DNA antibodies (double stranded DNA IgG antibodies and double stranded DNA IgM antibodies) was performed at the contract laboratory.

*9 TNFα, IL-6: Centralized measurement was performed at the contract laboratory.

*10 CRP: Centralized measurement was performed at the contract laboratory.

*11 Serum infliximab concentration: Blood samples were collected before administration when study drug administration was scheduled on the evaluation day. On the starting day of study drug administration and at Weeks 14 and 22, blood was collected twice in total before administration and one hour after completion of administration.

9.5.1.2 Patient Demographic Items

(1) Patient background data

The investigator (subinvestigator) investigated patient background factors listed below and recorded the obtained information in the CRFs. The study collaborator was allowed to transcribe information available from source documents to the CRFs.

1) Sex

2) Date of birth (year of grace)

3) Height (unit: cm, rounded off to the whole number)

4) Body weight (unit: kg, measured to the measurable number of digits)

5) Confirmation whether the relevant patient falls under the inclusion criteria (related to resistance to existing treatment)

6) Time of onset of ulcerative colitis (time of first onset)

7) Type (left-sided colitis/total colitis)

8) Clinical severity (mild/moderate/severe)

9) Clinical course (relapse-remitting type/chronic continuous type/acute fulminant [sudden onset] form/first attack form)

10) Number of stools per day before the development of disease (ulcerative colitis)*1

11) Drugs and therapies which had been used for treatment of ulcerative colitis*2

12) Colectomy for ulcerative colitis

13) History of treatments and surgeries (excluding colectomy) for ulcerative colitis

14) Complications*3

15) History of allergy

16) Tuberculosis tests

Medical interview (interview day, findings), chest X-ray (examination day, findings), chest CT scan (examination day, findings), and tuberculin skin test (assessment day, diameter of redness, diameter of induration) or QFT test or T-spot.TB test (blood sampling day, measured value, result of assessment), and presence or absence of treatment with antitubercular drugs from at least 3 weeks before the starting day of study drug administration.

17) History of vaccination

18) Past history of infections

Timing of investigation: at the time of registration (the investigation of complications was performed at the start of administration.)

*1: The value was expressed as whole numbers in principle, however if within a number range, the mean value of the maximum and minimum values was allowed to be used.

*2: Regarding drugs and therapies which had previously been used for treatment of ulcerative colitis, it was recorded in the CRFs whether a patient had been a non-responder to cyclosporine, tacrolimus, and cytapheresis or not within one year before registration.

*3: The presence or absence of complications at the start of administration was to be recorded in the CRFs, with detailed information of the complication, if present.

9.5.1.3 Efficacy Evaluation

9.5.1.3.1 Efficacy Endpoints

(1) CAI score

(2) CAI score-based remission

(3) Partial Mayo score

(4) Mayo score

(5) Mayo score-based response

(6) Mayo score-based remission

(7) Mucosal healing

(8) PUCAI score

(9) PUCAI score-based remission

(10) PUCAI score decrease by at least 20 points

(11) Steroid dose

(12) Steroid withdrawal

9.5.1.3.2 Confirmation of the Contents of the Symptom Assessment Sheet

Prior to CAI, partial Mayo, Mayo and PUCAI score assessment, the investigator (subinvestigator) or the study collaborator collected the Symptom Assessment Sheets from patients or legal representatives at the following evaluation time points, checked the contents of the sheets, and filled out the space for study site use. The original copy of the Symptom Assessment Sheet was retained at the medical institution, and a copy was submitted to the sponsor. Evaluation of CAI score-based non-responders was finished at Week 8. In addition, when CAI score-based non-responders discontinued the study after evaluation at Week 8, evaluation was not required at the time of discontinuation.

(1) Space filled out by patients and legal representatives

The investigator (subinvestigator) or the study collaborator confirmed that there was no inadequate/incomplete descriptions in the spaces filled out by patients or legal representatives during the medical examination at registration and at Weeks 2, 6, 8, 10, 14, 18, 22, 26, and 30 or at the time of discontinuation; if there was an inadequate/incomplete description, the investigator (subinvestigator) or the study collaborator asked the relevant patient or legal representative to make an additional description. If any correction was made to the sheet filled out by patients or legal representatives, the investigator (subinvestigator) or the study

collaborator checked with the relevant patient or legal representative, and affixed the seal for correction with the date.

(2) Space for study center use

During the medical examination at registration and at Week 2, 6, 8, 10, 14, 18, 22, 26, and 30 or at the time of discontinuation, if a drug for the treatment of constipation or diarrhea was used, the investigator (subinvestigator) or the study collaborator checked the relevant drug with the relevant patient or the patient's legal representative and documented the name of the drug. The investigator (subinvestigator) or the study collaborator also documented the presence/absence of pretreatment of sigmoidoscopy etc. in the space for study center use in the Symptom Assessment Sheet.

The investigator (subinvestigator) or the study collaborator assessed whether to use the data for efficacy evaluation or not based on the descriptions in the Symptom Assessment Sheet according to the following criteria, and documented the result of assessment in the space for study site use.

[Days to be excluded from assessment]*

- Days when drugs for the treatment of constipation or diarrhea were used
- Days when drugs having a potent antidiarrheal effect (e.g., loperamide hydrochloride, opium alkaloids, atropine sulfate-containing preparations) were used and for 2 days after use (3 days in total)
- Days when drugs that might affect stool frequency (e.g., laxatives) were used as pretreatment of sigmoidoscopy
- Days when colonoscopy or sigmoidoscopy was performed and for 3 days after the examination (4 days in total)

*: Days to be excluded from the evaluation period for CAI score (number of stools, blood in stools [based on weekly average]), partial Mayo score (number of stools, rectal bleeding) and PUCAI score.

(3) Space for investigator (subinvestigator)

The investigator (subinvestigator) confirmed that there were no inadequate descriptions in the Symptom Assessment Sheet at the time of registration, Weeks 2, 6, 8, 10, 14, 18, 22, 26 and 30, and the time of discontinuation of the study, and signed the sheet.

9.5.1.3.3 CAI Score

The CAI score was determined as a sum (0 to 29 points) of subscores for 7 evaluation items, including number of stools per week, blood in stools (based on weekly average), investigator's global assessment of symptomatic state, abdominal pain/cramps, temperature elevation due to ulcerative colitis, extraintestinal manifestations and laboratory tests (hemoglobin and ESR).

The investigator (subinvestigator) determined the CAI score based on the results of medical examination and tests (laboratory tests [Hemoglobin and ESR: measured in the institution], body temperature) and the data in the Symptom Assessment Sheet at the time of registration, Weeks 2, 6, 8, 10,

14, 18, 22, 26 and 30 and at the time of discontinuation, and documented the results of the determination in the CRF.

When administration of the study drug was scheduled on the evaluation day, the predetermined examinations and observations were performed before administration of the study drug. When sigmoidoscopy was scheduled on the evaluation day, the predetermined examinations and observations were performed before conducting the sigmoidoscopy. The evaluation of CAI score-based non-responders was discontinued at Week 8. When CAI score-based non-responders discontinued the study after the evaluation at Week 8, the evaluation at the time of discontinuation was not required.

When each of the scores determined by the investigator (subinvestigator) was recorded in the source documents such as medical record etc., the study collaborator was allowed to transcribe each score in the CRF.

(1) No. of stools weekly

The investigators (subinvestigators) determined the subscore based on the number of stools for 7 days before the evaluation day (the evaluation period for CAI score based on Symptom Assessment Sheet) excluding the days to be excluded from the evaluation specified in “9.5.1.3.2 Confirmation of the Contents of the Symptom Assessment Sheet,” according to the following conversion table, and recorded the score thus determined in the CRF. When the evaluation period of Symptom Assessment Sheet was less than 7 days, if assessment data for at least 4 days were obtained, the score was determined by conversion according to the calculation formula (Sum of the number of stools during the evaluation period of Symptom Assessment Sheet [for N days]) ×7/N, and recorded the calculated score in the CRF.

Part referenced in the Symptom Assessment Sheet: Number of stools		Score
Sum of the number of stools during the evaluation period of Symptom Assessment Sheet (7 days)	< 18 stools	0
	≥18 stools, ≤35 stools	1
	≥36 stools, ≤60 stools	2
	≥61 stools	3

(2) Blood in stools (based on weekly average)

The investigators (subinvestigators) determined the subscore based on blood in stools during the 7 days before the evaluation day (the evaluation period for CAI score based on Symptom Assessment Sheet) excluding the days to be excluded from the evaluation specified in “9.5.1.3.2 Confirmation of the Contents of the Symptom Assessment Sheet,” according to the following conversion table, and recorded the determined score in the CRF. When the evaluation period of Symptom Assessment Sheet was less than 7 days, if assessment data for at least 4 days were obtained, the score was determined according to the following conversion table, and the determined score was recorded in the CRF.

Part referenced in the Symptom Assessment Sheet: Blood in stools	Score	
"0" throughout the evaluation period of Symptom Assessment Sheet (7 days)	None	0
Other cases	Small amount: Bloody mucoid stools are not apparent, but blood in stools can be macroscopically confirmed.	2
"2" or "3" in 4 days or more (more than half) during the evaluation period of Symptom Assessment Sheet (7 days)	Evidence of bloody mucoid stools	4

(3) Investigator's global assessment of symptomatic state

The investigator (subinvestigator) examined patients on each evaluation day, made a global assessment of the clinical condition etc. for 7 days before the relevant evaluation day using the four-level rating scale shown below, and documented the score in the CRF.

0 = Good (No symptoms of ulcerative colitis)

1 = Average (for example, mildly symptomatic, but not interfering with activities of daily living such as work, school, and household)

2 = Poor (for example, symptomatic, and interfering with activities of daily living such as work and school commune, and household)

3 = Very poor (for example, symptomatic, and require rest in hospital)

(4) Abdominal pain/cramps

The investigator (subinvestigator) examined patients on each evaluation day, assessed abdominal pain/cramps for 7 days before the relevant evaluation day based on the information obtained from patients using the four-level rating scale shown below, and documented the score in the CRF.

0 = None (No symptoms)

1 = Mild (Occasionally annoying)

2 = Moderate (Always annoying)

3 = Severe (Too severe to bear)

(5) Temperature elevation due to ulcerative colitis (°C)

The investigator (subinvestigator) measured body temperature on each evaluation day, assessed it based on symptoms and findings using the two-level rating scale shown below, and documented the score in the CRF.

0 = 37-38°C (not more than 38°C)

3 = >38°C

(6) Extraintestinal manifestations

The investigator (subinvestigator) examined the presence/absence of extraintestinal manifestations (iritis, erythema nodosum, arthritis) on each evaluation day, and recorded the score in the CRF. The score was calculated by adding up the score for each of extraintestinal manifestations (0 to 9 points).

3 = Iritis

3 = Erythema nodusum

3 = Arthritis

(7) Laboratory findings

On each evaluation day, the investigator (subinvestigator) assessed the laboratory values of ESR and hemoglobin during the period from 7 days before the evaluation day to the evaluation day according to the categories shown below, and recorded the score in the CRF. Throughout the screening period and the efficacy evaluation period, the ESR and hemoglobin were to be measured at the relevant medical institution, and the places to measure these parameters were not changed. When there were multiple values measured, the value obtained on the closest day to the CAI score evaluation day was used.

1 = ESR > 50 mm in 1st h (ESR >50 mm/hr)

2 = ESR > 100 mm in 1st h (ESR >100 mm/hr)

4 = Hemoglobin < 100 g/L (Hb <10 g/dL)

When the measurement value of hemoglobin was less than 10 g/dL, the subscore for the relevant patient was determined to be 4 regardless of the measurement result of ESR. When the measurement result of ESR was not more than 50 mm/hr and the measurement result of hemoglobin was not less than 10 g/dL, the subscore was determined to be 0.

9.5.1.3.4 Partial Mayo Score and Mayo Score

The Mayo score consists of four subscores (stool frequency, rectal bleeding, physician’s global assessment and findings of endoscopy), each of which was assessed according to a four-level rating scale (0 to 3 points), and was determined from a total of the four subscores (0 to 12 points). In addition, the sum of the subscores (0 to 9 points) for stool frequency, rectal bleeding and physician’s global assessment was used as a partial Mayo score.

The investigator (subinvestigator) made an assessment based on the results of medical examination and the data in the Symptom Assessment Sheet to determine a partial Mayo score at the time of registration, at Weeks 2, 6, 8, 10, 14, 18, 22, 26 and 30, and the time of discontinuation. In addition, sigmoidoscopy was performed during the screening period (Performed in principle after registration and by the start of the study drug administration. In an unavoidable case, it was allowed to perform the examination within 2 weeks from the starting day of study drug administration and by 4 days before the start of the evaluation period.), at Week 30 and the time of discontinuation and sigmoidoscopy performed as much as possible to determine the Mayo score. The obtained assessment results were recorded in the CRF.

When administration of the study drug was scheduled on the evaluation day, the predetermined examinations and observations were performed before administration of the study drug for the relevant week. In addition, when sigmoidoscopy was scheduled on the evaluation day, the predetermined observations were performed before sigmoidoscopy. Evaluation of CAI score-based non-responders was discontinued at Week 8. In addition, when CAI score-based non-responders discontinued the study after evaluation at Week 8, an evaluation was not required at the time of discontinuation.

When each of the subscores determined by the investigator (subinvestigator) was recorded in the source documents such as medical records etc., the study collaborator was allowed to transcribe each subscore in the CRF.

(1) Stool frequency

The investigators (subinvestigators) determined the subscore based on the stool frequency for 3 days before the evaluation day (the evaluation period for partial Mayo score based on the Symptom Assessment Sheet) excluding the days to be excluded from the evaluation specified in “9.5.1.3.2 Confirmation of the Contents of the Symptom Assessment Sheet” and the daily stool frequency before the development of the disease (ulcerative colitis), according to the following conversion table, and recorded the determined score in the CRF.

CRF: Score		Part referenced in the Symptom Assessment Sheet: Stool frequency	
0	Normal number stools for this patient (as many as the number of stools per day before the development of the disease [ulcerative colitis])	<0.5	([Sum of the number of stools for 3 days] / 3) – (Number of stools per day before the development of the disease [ulcerative colitis])
1	1-2 stools more than normal (1 or 2 stools more than the number of stools per day before the development of the disease [ulcerative colitis])	≥0.5<br><2.5	
2	3-4 stools more than normal (3 or 4 stools more than the number of stools per day before the development of the disease [ulcerative colitis])	≥2.5<br><4.5	
3	5 or more stools more than normal (at least 5 stools more than the number of stools per day before the development of the disease [ulcerative colitis])	≥4.5	

The number of stools per day documented as demographic data in the CRF was used for the number of stools per day before the development of disease (ulcerative colitis). The number of stools per day before the development of disease (ulcerative colitis) was in principle expressed as a whole number; however if the number was within a number range, the mean value of the maximum and minimum values could be used (e.g., recorded as 1.5 for “1 to 2”).

(2) Rectal bleeding

The investigators (subinvestigators) recorded the worst (largest) subscore for rectal bleeding among those determined for the symptoms recorded in the Symptom Assessment Sheet for 3 days before the evaluation day (the evaluation period for partial Mayo score based on Symptom Assessment Sheet) excluding the days to be excluded from the evaluation specified in “9.5.1.3.2 Confirmation of the Contents of the Symptom Assessment Sheet” in the CRF in each evaluation day.

0 = No blood seen

1 = Streaks of blood with stool less than half the time

2 = Obvious blood with stool most of the time

3 = Blood alone passed

(3) Physician's global assessment

The investigator (subinvestigator) made a global assessment of the disease using a four-level rating scale shown below based on the above 3 subscores, medical interview (patient's complaints, general conditions, etc.), and other findings, etc. on each evaluation day, and recorded the score in the CRF.

0 = Normal	[As inactive as normal (phase of complete remission)]
1 = Mild disease	[Mildly active (mild)]
2 = Moderate disease	[Moderately active (moderate)]
3 = Severe disease	[Severely active (severe)]

(4) Findings of endoscopy

The investigator (subinvestigator) in principle performed sigmoidoscopy in the screening period (Performed after registration and by the start of the study drug administration. In an unavoidable case, it was allowed to perform the examination within 2 weeks from the starting day of study drug administration and by 4 days before the start of the evaluation period), at Week 30 and at the time of discontinuation as much as possible, and assessed findings of endoscopy using the atlas of endoscopic diagnosis (Appendix 16.1.1c, Attachment 5) as a guide based on a four-level rating scale shown below, and the determined score recorded in the CRF. When sigmoidoscopy was not performed in the screening period, the examination at Week 30 and at the time of discontinuation was not required.

0 = Normal or inactive disease

1 = Mild disease (erythema, decreased vascular pattern, mild friability)

2 = Moderate disease (marked erythema, absent vascular pattern, friability, erosions)]

3 = Severe disease (spontaneous bleeding, ulceration)

9.5.1.3.5 PUCAI Score

The PUCAI score was determined as a total of the subscores for each of the six evaluation items (0 to 85), including abdominal pain, rectal bleeding, stool consistency of most stools, number of stools per 24 hours, nocturnal stools and activity level.

The investigator (subinvestigator) assessed the information about the symptoms obtained from the patients by the medical interview at the time of registration, at Weeks 2, 6, 8, 10, 14, 18, 22, 26 and 30 and at the time of discontinuation, and recorded the results in the CRF.

When administration of the study drug was scheduled on the evaluation day, the evaluation was performed before administration of the study drug. In addition, when sigmoidoscopy was scheduled on the evaluation day, the evaluation was performed before conducting the sigmoidoscopy. Evaluation of CAI score-based non-responders was discontinued at Week 8. In addition, when CAI score-based non-responders discontinued the study after evaluation at Week 8, an evaluation was not required at the time of discontinuation.

When each of the subscores determined by the investigator (subinvestigator) was recorded in the source documents such as medical records etc. the study collaborator was allowed to transcribe PUCAI score in the CRF.

To determine PUCAI score, six evaluation items were assessed based on the information obtained from patients by the interview according to the following rules.

- An average of the conditions or values observed for 2 days before each evaluation day, excluding the days to be excluded from the evaluation specified in "9.5.1.3.2 Confirmation of the Contents of the Symptom Assessment Sheet," was used for determination of the score.
- In the case where clinical symptoms changed rapidly, the clinical condition in the last 24 hours was taken into consideration.

(1) Abdominal pain

0 = No pain

5 = Pain can be ignored

10 = Pain cannot be ignored

(2) Rectal bleeding

0 = None

10 = Small amount only, in less than 50% of stools

20 = Small amount with most stools

30 = Large amount (>50% of the stool content)

- When a large amount of blood was contained in almost every stool, "Large amount" was selected.

(3) Stool consistency of most stools

0 = Formed

5 = Partially formed

10 = Completely unformed

(4) Number of stools per 24 hours

0 = 0-2 stools

5 = 3-5 stools

10 = 6-8 stools

15 = >8 stools (more than 8 stools)

- In terms of tenesmus and incomplete defecation, an episode of the patient defecating several small stools in a very short term should be counted as one defecation.

(5) Nocturnal stools (any episode causing wakening)

0 = No

10 = Yes

(6) Activity level

0 = No limitation of activity

5 = Occasional limitation of activity

10 = Severe restricted activity

- "Occasional limitation of activity" means that the relevant patient can go to school or can perform an activity equivalent to going to school, but that patient's activity is limited (for example, the patient can go to school but cannot play during recess).
- "Severe restricted activity" means that the relevant patient cannot go to school or cannot perform an action equivalent to going to school.

9.5.1.4 Safety Evaluation

9.5.1.4.1 Safety Endpoints

(1) Adverse events (AEs) and adverse drug reactions (ADRs)

- AEs
- ADRs

(2) Objective findings

- Physical examination

Blood pressure, pulse rate and body temperature

- General laboratory tests

9.5.1.4.2 Objective Findings

(1) General laboratory tests

The investigator (subinvestigator) collected blood and urine samples on the starting day of study drug administration (Week 0), and at Weeks 2, 6, 8, 10, 14, 18, 22, 26 and 30, and the collected samples were delivered to the contract laboratory for measurement of the following laboratory parameters. For CAI score-based non-responders, laboratory tests were finished at Week 14. For discontinued patients, laboratory tests were performed at the time of discontinuation and 8 weeks after the last administration.

When administration of the study drug was scheduled on the examination day, collection of blood and urine samples was performed before study drug administration. Samples were collected by the contact laboratory. Test slips provided by the contract laboratory were stored by the medical institutions and the sponsor.

1) Hematology:

Red blood cell count, hemoglobin, hematocrit, white blood cell count, differential white blood cell (neutrophils, eosinophils, basophils, monocytes and lymphocytes), and platelet count

2) Blood biochemistry:

AST (GOT), ALT (GPT), ALP, LDH, γ-GTP, total protein, albumin, total cholesterol, total bilirubin, urea nitrogen (BUN), serum creatinine, and serum electrolyte (Na, K, Cl)

3) Urinalysis (qualitative):

Protein, glucose, occult blood, and urobilinogen

The amounts of blood and urine collected per each test were a respectively approximately 4 mL (including the sample for CRP measurement) and approximately 5 mL.

(2) Immunoserology

The investigator (subinvestigator) collected blood samples for measurement of antinuclear antibodies and double stranded DNA antibodies (double stranded DNA IgG and double stranded DNA IgM antibodies) on the starting day of study drug administration (Week 0), and at Weeks 14 and 30. For CAI score-based non-responders, the evaluation at Week 30 was not performed since the evaluation had been finished at Week 14. For discontinued patients, measurement was also performed at the time of discontinuation and 8 weeks after the last administration.

When administration of the study drug was scheduled on the examination day, collection of blood samples was performed before study drug administration. Samples were collected and measured by the contact laboratory. Test slips provided by the contract laboratory were stored by the medical institutions and the sponsor. The amount of blood collected per each test was approximately 3 mL.

(3) Physical examination

The investigator (subinvestigator) measured the blood pressure, pulse rate and body temperature immediately before the start of administration, every 30 minutes during administration, and every 30 minutes for 2 hours after the end of administration on each administration day. When there was at least a 15-minute interval between the end of administration and the latest measurement before the end of administration, measurement was also performed at the end of administration. The measurement results were recorded in the CRFs.

Case 1) When the duration of administration was 2 hr 00 min (an arrow indicates a measurement point)

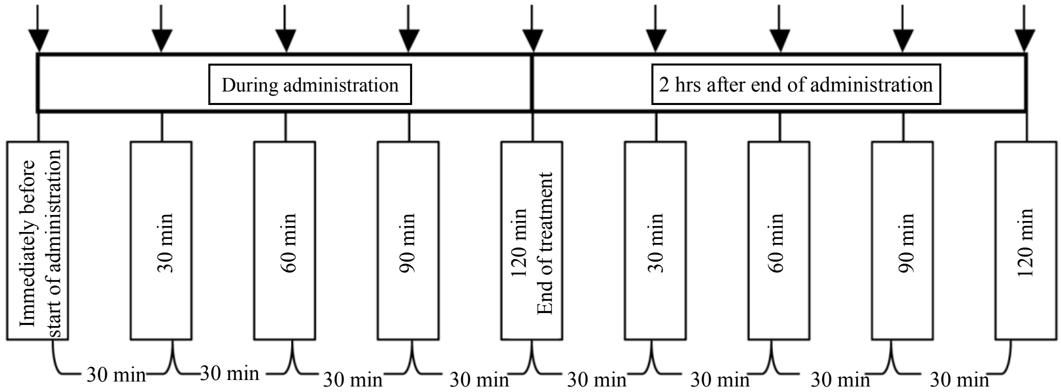

Case 2) When the duration of administration was 2 hr 15 min (an arrow indicates a measurement point)

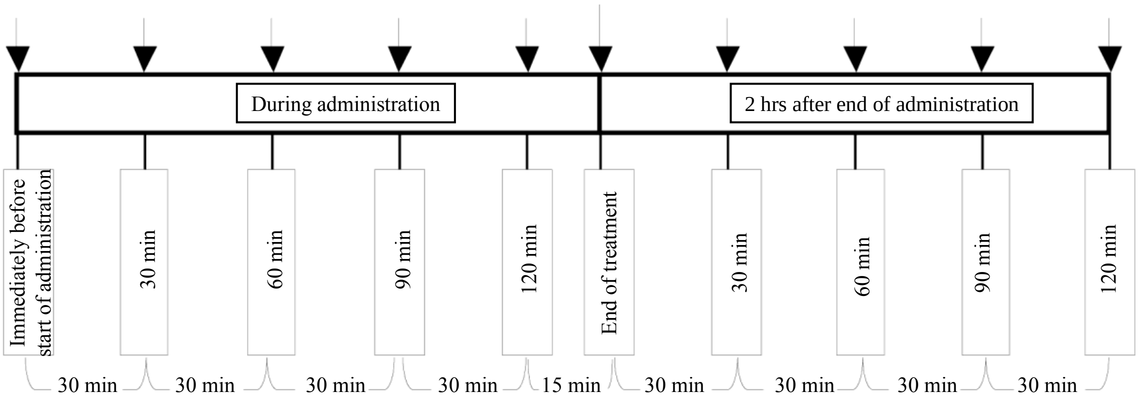

9.5.1.4.3 Adverse Events

An adverse event (AE) is any clinically unfavorable or unintended sign (including clinically significant abnormal laboratory values), symptom, or disease that occurred during the evaluation period after study drug administration regardless of causal relationship to the study drug. Worsening of the efficacy endpoints was not considered as an AE unless the event was serious or judged to be handled as an AE by the investigator (subinvestigator).

If an AE occurred, the investigator (subinvestigator) was to provide appropriate treatment. Regardless of the causal relationship with the study drug, the AE was followed in principle, until it was normalized or resolved to the extent that it was no longer assessed as an AE. If an AE was irreversible due to organic impairment, the patient was to be followed until the symptom stabilized or became unchanged.

(1) Symptoms or diseases

The investigator (subinvestigator) checked for the presence or absence of AEs at each visit from administration of the study drug to the end of the evaluation period by medical interview and examination.

In addition, to assess the presence or absence of respiratory infections including tuberculosis, chest X-rays and chest CT scans (as appropriate) were performed by the investigator

(subinvestigator) after obtaining informed consent and within 4 weeks before registration. Chest X rays and chest CT scans (as appropriate) were also performed at Weeks 14 and 30 during the evaluation period (only for CAI score-based responders) or at the time of discontinuation of the study to assess the presence or absence of abnormal findings. If any abnormal finding was observed, the disease or symptom causing the finding was handled as an AE.

(2) Objective findings

1) General laboratory test and physical examination (blood pressure, pulse rate and body temperature)

An abnormal value which was judged to be clinically significant* by the investigator (subinvestigator) was handled as an AE.

*: "A clinically significant abnormality" was assessed according to the following definitions:

- A case where the abnormal value was related to clinical signs or clinical symptoms. However, if these symptoms or signs had already been reported as AEs, it was not necessary to handle the relevant abnormal laboratory value as an AE.
- A case where any medical or surgical therapy for the relevant abnormal laboratory value was conducted.
- A case where the mode of administration (dose change, interventions or discontinuation) of the study drug was changed as a result of the relevant abnormal value.
- In addition to the above-mentioned cases, a case where the investigator (subinvestigator) judged the relevant abnormal value as clinically significant.

2) Immunoserological tests

Levels of anti-nuclear antibody, double stranded DNA IgG antibody and double stranded DNA IgM antibody deviating from the reference range shown below were considered as "abnormal," and those applicable to the following criteria for AEs were handled as AEs.

Test item	Reference range	Criteria for handling as AE
Anti-nuclear antibody	<40 times	Worsening of antibody titer by 3 ranks or more
Double stranded DNA IgG antibody	≤12 IU/mL	From negative to positive, from positive to positive (worsening)
Double stranded DNA IgM antibody	<6 U/mL	From negative to positive, from positive to positive (worsening)

3) Method and criteria for AE assessment

(a) Day of onset

The day of onset was defined as the day when the relevant symptom was observed or the examination day when the relevant abnormal laboratory value was found. If the day of onset was unclear, the reporting day of the relevant AE was used as the day of onset.

(b) Severity

Severity of AEs was classified according to the following criteria:

1. Mild: No interference with patient's daily living activities.

2. Moderate: Some interference with patient's daily living activities due to the event.

3. Severe: The patient cannot have daily living activities due to the event.

(c) Seriousness

Seriousness of AEs was classified as follows:

1. Non-serious: Other than those meeting the criteria of 2 below.

2. Serious: Those corresponding to the following a) to g)

a) Results in death,

b) Is life-threatening,

c) Requires hospitalization for treatment or prolongation of the existing hospitalization,

d) Results in disability,

e) May result in disability,

f) Is a serious case considered as serious as those listed in a) to e), or

g) Is a congenital anomaly/birth defect.

(d) Causal relationship with the study drug

The causal relationship with the study drug was classified as follows.

1. Definitely related

There is a temporal relationship between administration of the study drug and onset of the AE, and there were no other factors except for the study drug to explain the event, or no necessity to consider the influence by other factors except the study drug.

2. Probably related

There is a temporal relationship between administration of the study drug and onset of the AE, and the event was less likely to be explained by other factors such as primary disease, complications, and concomitant drugs and therapies.

3. Possibly related

The temporal relationship between administration of the study drug and onset of the AE cannot be ruled out, and the AE can be explained by other factors such as primary disease, complications, and concomitant drugs and therapies, but the relationship with the study drug cannot completely be ruled out.

4. Not related

There is no temporal relationship between administration of the study drug and onset of the AE.

The AE can be reasonably explained by other factors such as the primary disease, complications, and concomitant drugs and therapies.

AEs classified as other ratings than “Not related” were handled as ADRs.

(e) Outcome

The outcome of AEs was classified according to the following criteria.

1. Recovered: Normalized or resolved to a level that the event was no longer considered as an AE.
2. Ongoing: Not resolved at the time of assessment.
3. Death: Results in death due to the relevant AE.
4. Unknown: Outcome unknown because the patient died due to a cause other than the relevant AE, or the outcome cannot be confirmed because it was impossible to contact the patient.

(f) Day of outcome

The day of outcome was classified according to the following criteria.

Recovered: Day when the patient recovered. If the day of recovery is uncertain, the day when the outcome was confirmed or determined is used as the day of recovery.

Ongoing: Day when the ongoing AE was confirmed or determined.

Death: Day when the patient died. If the day of death is unclear, the day when the death was confirmed or determined is used as the day of death.

Unknown: Day when the patient died if the outcome was unknown because the patient died due to a cause other than the relevant AE. In other cases, the day when the outcome was confirmed or determined is used as the day of outcome for unknown criteria.

(g) Follow-up

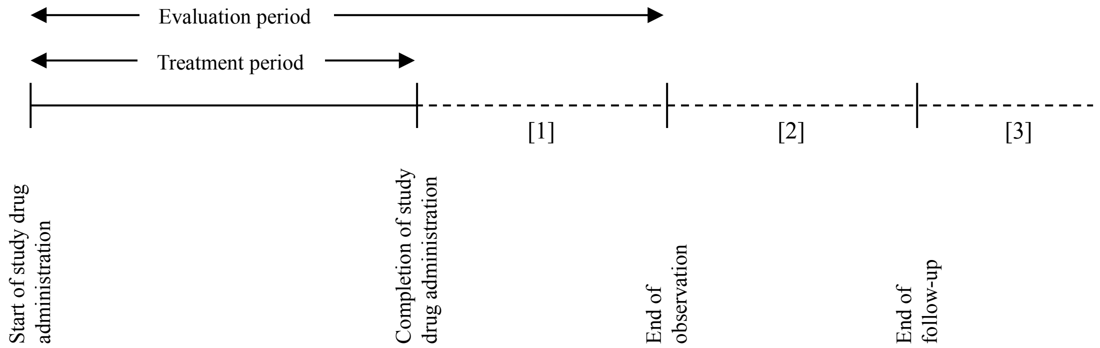

- The period [1] after completion of study drug administration was set as 56 days, and the presence or absence of AEs was assessed.
- The period [2] after the end of observation was set as 28 days, and the AEs that occurred during the evaluation period (the treatment period + [1]) were followed up.
- The clinical courses of the AEs followed up during the period [2] after the end of observation were recorded in the CRFs.
- When AEs had not been recovered, the day of outcome recorded in the CRF was the day of observation after the end of period [2] after the end of observation.
- ADRs that had not been recovered at the end of period [2] after the end of observation were followed up ([3]).

- If there was a reasonable cause for discontinuation of the investigation after the end of the evaluation period (after the end of period [1]), the cause was recorded in the CRFs and the follow-up was finished.

4) Entries to the CRF

If an AE was observed, the investigator (subinvestigator) recorded the following information on the AE page of the CRF, the name of the AE*, severity, seriousness, infections, day and time of onset (filled in the time of onset in the case where the AE occurred between the start of administration of the study drug and 2 hours after the end of administration), day of outcome, outcome, the treatment, and causal relationship with the study drug were recorded. If a disease name was recorded as the name of the AE, the symptoms or abnormal changes in laboratory test values associated with the disease, and abnormal findings in the chest X-ray/CT scan were not to be recorded in the AE page. Since safety evaluation for the immunoserological tests was performed separately, findings observed in the immunoserological tests were not to be recorded in the AE page.

When the outcome of an AE was assessed as other categories than "recovered" but follow-up was considered unnecessary, the rationale for judgment that follow-up was unnecessary, as well as the reason for assessment of the causal relationship with the study drug were recorded in the "comment column" for AEs. In addition, in the case where the relevant AE led to discontinuation of the study, the AE was recorded as an AE leading to discontinuation on the discontinuation page of the CRF.

The study collaborator was able to transcribe this information to the CRFs only when the information was described in the source documents such as medical records.

*: "AE name" was presented according to the following rules:

- The name of diagnosis is used in principle.
- If the diagnosis is uncertain, the symptoms are used.
- If several symptoms appear but are able to be represented as one diagnosis, the diagnosis is used.
- Surgical interventions are not included in AEs. If diseases or symptoms which require surgical interventions are observed, they are considered as AEs.

9.5.1.5 Other Laboratory Test Parameters

(1) TNFα, IL-6, CRP

The investigator (subinvestigator) collected blood samples only on the starting day of study drug administration (Week 0) for measurement of TNFα and IL-6, and on the starting day of study drug administration (Week 0) and at Weeks 2, 6, 8, 10, 14, 18, 22, 26 and 30 and at the time of discontinuation for measurement of CRP. For CAI score-based non-responders, measurement of these parameters was finished after the measurement at Week 8. In addition, when CAI score-based non-responders discontinued the study after evaluation at Week 8,

measurement of these parameters was not required at the time of discontinuation. When administration of the study drug was scheduled on the day of tests, the blood sample was collected before the study drug administration.

Blood samples for TNFα and IL-6 measurement were centrifuged, and a certain amount of serum or plasma obtained was collected and stored frozen. Samples for CRP measurement were handled in the same manner as those for general laboratory tests. Sample were collected and measured by the contract laboratory. Test slips provided by the contract laboratory were stored by the medical institution and the sponsor.

The amount of blood collected for each sampling was approximately 2 mL for TNFα and IL-6 measurement, and a total of approximately 4 mL for CRP measurement and blood biochemistry tests.

9.5.2 Appropriateness of Measurements

(1) Rationale for efficacy endpoints

Since this study was conducted on children, it was considered difficult to perform sigmoidoscopy, which is needed in order to determine the Mayo score in all of the patients. Therefore, it was decided to assess the efficacy of the study drug using the CAI score, which is a noninvasive score used as an evaluation index in the domestic clinical study of TA-650 in adults. The CAI score is an index containing a good balance of clinical symptoms and laboratory test data, and has been frequently used as a noninvasive evaluation index. The CAI score is also reported to have a high correlation with the Mayo score (r=0.92) [1)]. In addition to the CAI score, a partial Mayo score, which has been used in domestic and overseas clinical studies, was also evaluated. For patients who underwent sigmoidoscopy, the Mayo score was also evaluated. The PUCAI score, which has been used in overseas clinical studies in children, was also evaluated in this study.

In addition, the steroid dose on each evaluation day was used as an endpoint to evaluate the effect of TA-650 to reduce steroid dose in pediatric patients with ulcerative colitis.

(2) Rationale for safety endpoint

As with clinical studies of TA-650 conducted to date, general laboratory tests as well as measurement of anti-nuclear antibodies and double stranded DNA IgG and IgM antibodies were performed to monitor the occurrence of lupus-like symptoms. Since patients treated with TA-650 should also be carefully monitored for AEs such as infections including tuberculosis, a chest X-ray and chest CT scan (as appropriate) were performed.

9.5.3 Drug Concentration Measurements

9.5.3.1.1 Items and Timing of Examinations and Observations

The investigator (subinvestigator) collected blood samples for measurement of the serum infliximab concentration and ATI.

The drug concentration assay facility prepared a separate study protocol (Appendix 16.1.13d) before the start of measurement, then measured the serum concentration of infliximab and ATI, and prepared a

final report (Appendix 16.1.13e) for the measurement results. The person responsible for clinical pharmacological analysis prepared the pharmacokinetic analysis plan (Ver. 1.0) (prepared on January 19, 2015) (Appendix 16.1.9c) by the time the data were locked. After data were locked, pharmacokinetics of the study drug was analyzed and a pharmacokinetic analysis report (Appendix 16.1.9d) was prepared.

(1) Time points for blood collection

The investigator (subinvestigator) collected blood samples on the starting day of study drug administration (Week 0) and at Weeks 2, 6, 8, 10, 14, 18, 22, 26 and 30. For CAI score-based non-responders, the blood sampling was finished at Week 14. For discontinued patients, the blood sampling was also performed at the time of discontinuation and 8 weeks after the last administration.

When administration of the study drug was scheduled on the blood sampling day, the blood sample was collected before the study drug administration. However, on the starting day of study drug administration (Week 0) and at Weeks 14 and 22, the blood was collected twice in total before administration and 1 hour after completion of administration. For CAI score-based non-responders, there was no blood collection 1 hour after completion of administration at Week 14.

(2) Blood volume: approximately 2 mL per test

(3) Processing of blood samples

A certain volume of serum was obtained for pharmacokinetic analysis by centrifugation of collected blood (approximately 2 mL), and stored frozen below -20°C.

The cryopreserved samples for pharmacokinetic analysis were collected from the medical institutions by the contract laboratory. The contract laboratory delivered the samples to the drug concentration assay facility in accordance with the “operating procedures for shipment of samples” specified separately.

(4) Entries in the CRF

The investigator (subinvestigator) recorded the date and time of blood collection in the CRF.

[Rationale for Setting]

In reference to overseas ACT1 and ACT2 studies and domestic clinical studies in patients with ulcerative colitis, the sampling time points which were needed to study pharmacokinetics of TA-650 when administered at a dose of 5 mg/kg at Weeks 0, 2 and 6 followed by administration at Weeks 14 and 22 were set.

9.6 Data Quality Assurance

In order to maintain the quality and reliability of the present study, the sponsor performed “quality control of the study” in accordance with GCP standard operating procedures (SOP) of Mitsubishi Tanabe Pharma Corporation and “quality assurance of the study” in accordance with GCP audit SOP of Mitsubishi Tanabe Pharma Corporation. The medical institutions and investigators cooperated in quality control and quality assurance of the study implemented by the sponsor.

In quality control of the study, the monitors had direct access as appropriate to confirm that the present study was conducted in compliance with the study-related operating procedures of the medical institution, the current protocol and GCP. The monitors also confirmed that it was possible to verify that the descriptions in the CRFs reported from the investigator (subinvestigator) were accurate and complete, by referring to study-related records such as source documents. The monitoring procedures (Appendix 16.1.13e) specifying the monitoring procedures in details was prepared and monitoring was performed in accordance with the procedures.

In order to ensure that the study was conducted in compliance with the protocol and GCP, the auditor conducted audits in compliance with GCP audit SOP and confirmed that the quality of the study was appropriately controlled.

9.7 Statistical Methods Planned in the Protocol and Determination of Sample Size

9.7.1 Statistical and Analytical Plans

See the statistical analysis plan (Ver. 3.0) (Appendix 16.1.9a) and the pharmacokinetic analysis plan (Ver. 1.0) (Appendix 16.1.9c) for details.

9.7.1.1 Analysis Sets

(1) Efficacy analysis set

Efficacy analysis set was the full analysis set (hereafter referred to as FAS) except for the following patients:

(a) Patient without the target disease (ulcerative colitis).

(b) Patients who had received no study drug in the evaluation period.

(c) Patients without any available efficacy data in the evaluation period.

(2) Safety Analysis Set

Safety analysis set does not include the following patients.

(a) A patient who had received no study drug in the evaluation period.

(b) A patient without any available safety data after the start of study drug administration in the evaluation period.

(3) Pharmacokinetic Analysis Set

Pharmacokinetic analysis set was defined as described below, but handling of each case was decided in accordance with the arrangements made by the clinical conference.

Among the patients who had received at least one dose of the study drug, the population of patients from whom serum infliximab concentration or ATI data was obtained once or more times after administration of the study drug was used as the pharmacokinetic analysis set.

9.7.1.2 Data Handling

Obtained data were handled as described below. When it was required to consider data handling with respect to the items not stipulated in this section, rules for data handling and data handling were decided in accordance with the procedures stipulated in the sponsor's GCP SOP. Detailed procedures are described in the statistical analysis plan (Appendix 16.1.9a).

9.7.1.2.1 Missing Values

(1) In the case of a missing examination or the case where measurement was impossible due to problems with test samples, the relevant item was treated as a missing value.

(2) If the dose of the study drug was <75% of the dose specified for each administration day, data for efficacy evaluation obtained from the relevant administration to the next administration of the study drug was treated as missing data.

(3) In the case of violations involving prohibited concomitant drugs and therapies, all of the data obtained from the relevant patient is handled in accordance with the arrangements made by the clinical conference and is not treated as missing data.

9.7.1.2.2 Handling of Dropouts or Missing Values in Efficacy Evaluation Data

9.7.1.2.2.1 Permitted Time Range for Time Lag at Evaluation Time Point

In the tabulation of data by measurement time point, data falling within the permitted time range for the day of evaluation were selected, and imputation of the missing data by data outside the permitted range was not performed. The permitted time ranges were set as ±6 days for Weeks 2, 6 and 8, ±7 days for Week 10 and the range from -13days to +14days after Week 14.

For the efficacy evaluation (excluding other laboratory parameters), evaluation performed after study drug administration in the study drug administration week was not selected as the data for the relevant week. When multiple data existed within the permitted time range, data was selected in accordance with the following rules:

- Evaluation performed on the closest day to the baseline day should be selected.
- When there are two evaluations having the same time lag before and after the baseline day, the evaluation before the baseline should be selected
- When there are several evaluations on a single measurement day, evaluation of a label for the specified timing should be selected

9.7.1.2.2.2 Handling of Data at Each Time Point in Tabulation of Data by Week of Last Administration of Study Drug

The permitted time range by week of last administration of the study drug was set as shown below.

Last administration of study drug	Permitted time range (week)
Week 0	0, 2
Week 2	0, 2, 6

Week 6	0, 2, 6, 8, 10, 14
Week 14	0, 2, 6, 8, 10, 14, 18, 22
Week 22	0, 2, 6, 8, 10, 14, 18, 22, 26, 30

9.7.1.2.2.3 Handling of Efficacy Evaluation Data at Discontinuation

Efficacy evaluation at the time of discontinuation of the study was specified by the timing of the last administration of the study drug.

Evaluations until the next scheduled administration were candidate data for the time points.

Last administration of study drug	Permitted time range for candidate evaluation time point at discontinuation (week)
Week 0	2
Week 2	6
Week 6	10, 14
Week 14	18, 22
Week 22	26, 30

9.7.1.2.2.4 Handling of Data at Last Time Point

(1) CAI score-based non-responders

Case of patient who completed the treatment: Last evaluation obtained within the permitted time range for Week 8 was selected.

(2) CAI score-based responders

Case of patient who completed the treatment: Last evaluation obtained within the permitted time range for Week 30 was selected.

(3) Discontinued patient (CAI score-based non-responders and responders)

Discontinued case: The permitted time range for data at last time point was specified as listed below.

Last administration of study drug	Permitted time range (day)
Week 0	Last evaluation performed within 20 days from the initial administration is selected.
Week 2	Last evaluation performed within 48 days from the initial administration is selected
Week 6	Last evaluation performed within 112 days from the initial administration is selected.
Week 14	Last evaluation performed within 168 days from the initial administration is selected.
Week 22	Last evaluation performed within 224 days from the initial administration is selected.

9.7.1.2.2.5 Treatment Failure

For patients who met the following conditions (defined as “treatment failure”), the CAI score, partial Mayo score, Mayo score and PUCAI score determined at the time of registration were used for evaluation after the time point where the relevant patient met the conditions, regardless of the actual scores in terms of the efficacy endpoints and steroid dose. In addition, for imputation (TF) of data on the remission rate based on each score (CAI, Mayo and PUCAI), the Mayo score-based response rate, the rate of mucosal healing and the rate of patients who achieved a PUCAI score decrease by at least 20 points, the above mentioned patients were respectively handled as “failure to achieve remission,” “failure to achieve response,” “failure to achieve mucosal healing” and “failure to achieve a decrease by at least 20 points,”. For imputation of steroid dose data, the actual steroid dose was substituted by the steroid dose at Week 0, and in terms of steroid withdrawal, the relevant patients were handled as a “failure to achieve steroid withdrawal”

Treatment failure

(1) Patients who underwent colectomy or enterostomy

(2) Patients who discontinued the study due to exacerbation of the primary disease or inadequate response to the study drug

(3) Patients who experienced the start of administration or increase in a dose of the following drugs due to exacerbation of the primary disease, etc.

- Steroids (oral preparations, injection, enema, suppository)
- Azathioprine, 6-mercaptopurine
- Salazosulfapyridine preparations (oral preparation, suppository)
- 5-aminosalicylate acid preparations (oral preparations, enema)

In imputation of the data related to these treatment failures, the Last Observation Carried Forward (hereinafter referred to as LOCF) approach was applied first, where existing values which were obtained at the evaluation point immediately before the missing time point were used, and then TF imputation of the evaluation results obtained after the relevant time point was performed.

Whether a patient would be handled as a treatment failure or not was decided in accordance with the judgment made by the clinical conference.

9.7.1.2.2.6 Handling of Data on Each Score

(1) CAI score

Any missing data of CAI subscores at each time point were imputed with the last observed values (CAI subscore) (LOCF), and the LOCF data represented the subscore at the relevant time point, and were used for determination of CAI score at the relevant time point.

For discontinued patients and CAI score-based non-responders, the subscores at time points with no assessment data after discontinuation for the former and after Week 8 for the latter, were imputed in the same way, and the CAI score was determined.

(2) Partial Mayo score

Any missing data of the 3 partial Mayo subscores (stool frequency, rectal bleeding, physician's global assessment) at each time point were imputed within the last observed value (LOCF), and the LOCF data represented the subscore at the relevant time point, and was used for determination of the partial Mayo score at the relevant time point.

For discontinued patients and CAI score-based non-responders, the 3 subscores at the time points with no assessment data after discontinuation for the former and after Week 8 for the latter, were imputed in the same way, and the partial Mayo score was determined.

(3) Mayo score

The Mayo score was determined for patients who had undergone assessment of the findings of endoscopy subscores in the screening period. When findings of endoscopy at Week 30 were missing, these data were imputed with the evaluation at the time of discontinuation (LOCF), and the LOCF data were treated as the subscore at Week 30 and used to determine the Mayo score at Week 30.

In the case where the evaluation had not been performed at Week 30 or at the time of discontinuation, the Mayo scores were not determined due to lack of data to be used for imputation.

(4) PUCAI score

Any missing data of PUCAI subscores at each time point were imputed with the last observed values (PUCAI subscore) (LOCF), and the LOCF data represented the subscore at the relevant time point, and was used for determination of the PUCAI score at the relevant time point. For discontinued patients and CAI score-based non-responders, the subscores at the time points with no assessment data after discontinuation for the former and after Week 8 for the latter, were imputed in the same way, and the PUCAI score was determined.

(5) Remission based on each score (CAI, Mayo, PUCAI)

In terms of remission based on each score (CAI, Mayo, PUCAI), patients were evaluated on whether the patient achieved or failed to achieve remission based on each score (CAI, Mayo, PUCAI) determined in accordance with the above (1), (3) or (4), unless all of the subscores were missing at each time point.

When all of the subscores were missing, the relevant patient was assessed as having failed to achieve remission.

For discontinued patients and CAI score-based non-responders, data of the remission rates based on each score (CAI, Mayo, PUCAI) after discontinuation for the former and after Week 8 for the latter were imputed with failure to achieve remission.

(6) Mayo score-based response, mucosal healing

In terms of Mayo score-based response, patients were judged as whether having achieved or failed to achieve remission based on the Mayo score determined in accordance with the above (3), unless all of the subscores were missing at the relevant time point. For mucosal healing, patients were evaluated on whether having achieved or failed to achieve mucosal healing based on the subscore of findings of endoscopy determined in accordance with the above (3). In the case where the determination of subscores of findings of endoscopy had not been performed at Week 30 or at the time of discontinuation, judgment was not performed due to lack of data to be used for imputation.

For discontinued patients, the data on Mayo score-based response and mucosal healing at the time points with no assessment data after discontinuation were respectively imputed with failure to achieve remission based on Mayo score and failure to achieve mucosal healing.

(7) PUCAI score decrease by at least 20 points

In terms of a PUCAI score decrease by at least 20 points, patients were evaluated on whether they achieved or failed to achieve a PUCAI score decrease by at least 20 points based on the PUCAI score determined in accordance with the above (4), unless all of the subscores were missing at the relevant time point.

When all of the subscores were missing, the relevant patient was assessed as a failure to achieve a PUCAI score decrease by at least 20 points. For discontinued patients and CAI score-based non-responders, data on a PUCAI score decrease by at least 20 points at the time point with no assessment data after discontinuation for the former and after Week 8 for the latter, was imputed as non-applicable.

9.7.1.2.2.7 Handling of Data on Steroid Dose

(1) Steroid dose

The steroid dose at the time points after discontinuation for discontinued patients or at the time points after Week 8 for CAI score-based non-responders was imputed with the last observed value (steroid dose) (LOCF).

(2) Steroid withdrawal

In terms of steroid withdrawal at each time point, patients were assessed on whether steroid withdrawal was achieved or not based on the steroid dose calculated in accordance with the above (1).

For discontinued patients and CAI score-based non-responders, data on steroid withdrawal at the time point with no assessment data after discontinuation for the former and after Week 8 for the latter were imputed with failure to achieve steroid withdrawal.

9.7.1.2.3 Handling of Safety Evaluation Data

(1) Permitted time range for time lag at evaluation time point

In tabulation of data for each measurement point, data which fell within the permitted time range for the day of evaluation were selected, and no imputation of the missing data by data outside the permitted range was performed. The permitted time ranges were set as ±6 days for Weeks 2, 6 and 8, ±7 days for Week 10 and the range from -13days to +14days after Week 14.

For safety evaluation (including the endpoint [CRP] of other laboratory test), in the study drug administration week excluding the starting day of study drug administration (Week 0), evaluation performed after study drug administration was selected as the data for the relevant week. When multiple data existed within the permitted time range, data were selected in accordance with the following rules:

- Evaluation performed on the closest day to the baseline day should be selected.

- When there are two evaluations with the same time lag before and after the baseline day, the evaluation after the baseline should be selected.
- When there are several evaluations on a single measurement day, evaluation of a label for the specified timing should be selected.

The permitted time range by week of last administration of the study drug was set as shown below.

- For laboratory test values and other laboratory test parameter (CRP)

Last administration of study drug	Permitted time range (week)
Week 0	0, 2
Week 2	0, 2, 6
Week 6	0, 2, 6, 8, 10, 14
Week 14	0, 2, 6, 8, 10, 14, 18, 22
Week 22	0, 2, 6, 8, 10, 14, 18, 22, 26, 30

- For immunoserological tests

Last administration of study drug	Permitted time range (week)
Weeks 0, 2	0
Weeks 6, 14	0, 14
Week 22	0, 14, 30

9.7.1.2.4 Handling of Pharmacokinetic Data

(1) Handling of dropouts or missing values

In the case of missing examination values or the case where measurement was impossible due to problems with test samples, the relevant measurement result was treated as a missing value. Handling of other data was performed in accordance with the arrangements made by the clinical conference (approved on December 15, 2014) and PK/PD/PGx Data Handling Assessment (approved on December 24, 2014).

Handling of the results of ATI determination after administration of Remicade® was decided by the PK/PD/PGx Data Handling Assessment. Since it is clear that treatment with Remicade® would affect the ATI assessment, the results of ATI determination at the time point after administration of Remicade® (8 weeks after the last administration) for the relevant patients (Patient ID Code: TA-650UC-003-01, TA-650UC-022-02) was not included in the analysis, and treated as a missing value. ATI determination for the relevant patient was performed using the data obtained at the time points other than that where a missing value was obtained according to the ATI determination flow chart (Figure 13.6.7-2-1 and Figure 13.6.7-2-2) shown in 1) of the protocol for Determination of serum infliximab concentration and antibodies to infliximab (ATI) in “Clinical studies of TA-650 in pediatric patients with ulcerative colitis” (Appendix 16.1.13d).

(2) Handling of measurement values less than the limit of quantitation

When the result of measurement of serum infliximab concentration was less than the limit of quantitation (limit of quantitation: 0.1 μg/mL), the relevant result was treated as 0 μg/mL in the calculation, and represented as “BLQ.”

9.7.2 Efficacy Evaluation

The following analyses were performed in the efficacy analysis set.

9.7.2.1 CAI Score

(1) Endpoints

- CAI score, CAI score-based remission

(2) Analysis method

- Both of the results for which no imputation of missing data was performed (each evaluation point includes the “last” time point) and those for which TF imputation was applied were shown (Summary statistics of each subscore were excluded).
- Summary statistics of CAI score and CAI score change and the rate of CAI score-based remission at each evaluation point were calculated.

9.7.2.2 Partial Mayo Score

(1) Endpoint

- Partial Mayo score

(2) Analysis method

- Both of the results for which no imputation of missing data was performed (each evaluation point includes the “last” time point) and those for which TF imputation was applied were shown (Summary statistics of each subscore were excluded).
- Summary statistics of partial Mayo score and partial Mayo score change at each evaluation point were calculated.

9.7.2.3 Mayo Score

(1) Endpoints

- Mayo score, Mayo score-based response, Mayo score-based remission, and mucosal healing

(2) Analysis method

- Both of the results for which no imputation of missing data was performed (each evaluation point includes the “last” time point) and those for which TF imputation was applied were shown (Summary statistics of each subscore were excluded).

- Summary statistics of the Mayo score and Mayo score change and the rate of Mayo score-based response, the rate of Mayo score-based remission and the rate of mucosal healing at each evaluation point were calculated.

9.7.2.4 PUCAI Score

(1) Endpoints

- PUCAI score, PUCAI score-based remission, PUCAI score decrease by at least 20 points

(2) Analysis method

- Both of the results for which no imputation of missing data was performed (each evaluation point includes the “last” time point) and those for which TF imputation was applied were shown (Summary statistics of each subscore were excluded).
- Summary statistics of PUCAI score and PUCAI score change, and the rate of PUCAI score-based remission and the proportion of patients who achieved a PUCAI score decrease by at least 20 points at each evaluation point were calculated.

9.7.2.5 Steroid Dose

(1) Endpoints

- Steroid dose, steroid withdrawal

(2) Analysis method

- Among FAS, patients who had been using steroids (oral preparations) at the time of registration were included in the analysis set.
- Both of the results for which no imputation of missing data was performed (each evaluation point includes the “last” time point) and those for which TF imputation was applied were shown.
- Summary statistics of steroid dose and the rate of steroid dose change, and the rate of steroid withdrawal at each evaluation point were calculated.

9.7.3 Safety Evaluation

The following analyses were performed in the safety analysis set. Names of symptoms of AEs were coded using the Medical Dictionary for Regulatory Activities/ Japanese edition (MedDRA/J ver. 17.1).

9.7.3.1 Safety Data Summary

(1) Endpoints

AEs, AEs (SOC is other than investigations), ADRs, ADRs (SOC is other than investigations), serious AEs, serious ADRs, AEs leading to discontinuation, AEs by severity, AEs by time point, ADRs leading to discontinuation, infections, infections (ADRs), serious infections, serious infections (ADRs), infections leading to discontinuation of treatment, infusion reaction, infusion reaction (ADRs), serious infusion reaction, infusion reaction leading to discontinuation

of administration, AEs (immunoserological tests), general laboratory tests, physical examinations.

(2) Analysis method

The number of patients who experienced an event and the incidence rate (%) of the event are shown for each endpoint.

9.7.3.2 Breakdown of AEs

(1) Endpoints

AEs, ADRs, serious AEs, serious ADRs, AEs leading to discontinuation, infections, infections (ADRs), serious infections, serious infections (ADRs), infusion reaction and infusion reaction (ADRs).

(2) Analysis method

For each endpoint, the number of patients who experienced an event, incidence rate (%), and the number of events are listed by the overall events, System Organ Class (SOC) according to MedDRA/J, and SOC/preferred term (PT). SOCs were represented in ascending internationally agreed order, and PTs were represented in descending order for the number of patients who experienced the event, and ascending order for the PT code.

9.7.3.3 AEs (Immunoserological Tests)

For AEs (immunoserological tests), the number of patients who experienced the event, incidence rate (%) and the number of events are listed by “antinuclear antibody increased,” “double stranded DNA (IgG) antibody positive” and “double stranded DNA (IgM) antibody positive.”

9.7.3.4 Infusion Reaction

(1) Endpoints

Infusion reaction, infusion reaction (ADR)

(2) Analysis method

1) Incidence rate of infusion reactions by number of doses

The number of patients who experienced an event and the incidence rate (%) by number of doses is represented.

2) Incidence rate of infusion reactions per one dose

The overall number of doses (total number of doses in all patients), the number of events (the number of doses where any infusion reactions were observed) and the incidence rate of infusion reaction per one dose (the percentage of the number of events with respect to the overall number of doses) is represented.

3) Incidence rate of infusion reactions by ATI determination

The number of patients who experienced an event and the incidence rate (%) by ATI determination (negative, positive, inconclusive) is represented.

9.7.3.5 AEs Classified by Severity

(1) Endpoints

AEs, ADRs, infections and infections (ADRs)

(2) Analysis method

For each endpoint (other than immunoserological tests), the number of patients who experienced an event and the incidence rate (%) are listed by severity (mild, moderate and severe) as well as by overall events, SOC according to MedDRA/J, and SOC/ PT.

9.7.3.6 AEs by Timing of Occurrence

(1) Endpoints

AEs, ADRs, Infections and Infections (ADRs)

(2) Analysis method

For each endpoint (SOC is other than Investigations), the number of patients who experienced the event and the incidence rate (%) are listed by the following classification categories:

Classification categories for timing of occurrence: between the starting day of study drug administration (Week 0) and before administration at Week 14, between after administration at Week 14 and before administration at Week 22, between after administration at Week 22 and evaluation day at Week 30.

AEs which occurred on the day of study drug administration excluding those that clearly occurred after administration of the study drug such as infusion reactions, were tabulated as AEs which occurred before administration of the study drug.

9.7.3.7 Immunoserological Tests

(1) Endpoints

“Antinuclear antibody increased,” “double stranded DNA (IgG) antibody positive” and “double stranded DNA (IgM) antibody positive,”

(2) Analysis method

For each endpoint, the cross tabulation between each determination (negative, positive) and the time points at Weeks 0 (the starting day of administration), 14 and 30 and all of the time points after administration was performed.

9.7.3.8 General Laboratory Tests

(1) Endpoints

Hematology, blood biochemistry and urinalysis (qualitative)

(2) Analysis method

For each laboratory parameter, the number of patients who experienced an abnormal change and incidence rate (%) of abnormal change are listed by abnormal increase and abnormal decrease. In addition, for hematology and blood biochemistry, summary statistics of laboratory

test values (n, Median, Q1, Q3, Min and Max) at each evaluation point were calculated. Summary statistics were calculated based on the measurement results obtained from all of the patients, and not calculated by the age or sex of the patients.

For urinalysis (qualitative), the frequency distribution of urinalysis results in each of the assessment categories at each evaluation point is listed.

For urinalysis (qualitative, other than urobilinogen), frequency distribution of each judgment, "normal" and "abnormal" at each evaluation time point (only Weeks 8, 14, 22 and 30) is listed. For urinalysis (urobilinogen), the frequency distribution of each judgment "low," "normal" and "high," at each evaluation time point (only Weeks 8, 14, 22 and 30) is listed.

Since reference ranges of laboratory parameters for children vary depending on age and sex, the reference ranges of laboratory parameters were established by selecting reference values by sex and age (the age at informed consent obtainment is used) in reference to "New Pocket Guide of Laboratory Test Reference Values for Children"[2)]. Since no reference ranges for differential count of WBC are listed in this material, abnormal changes were not assessed for this parameter. Since it was judged that the reference ranges for urinalysis (qualitative) in adults can also be used in children without any clinical problems, the reference values (for adults) provided from the contract laboratory were used to assess abnormal changes.

9.7.3.9 Physical Examination

(1) Endpoints

Systolic and diastolic blood pressure, pulse rate, and body temperature

(2) Analysis method

For each endpoint, summary statistics (n, Mean, SD, Min and Max) by timing of administration and measurement time at each evaluation point are listed.

9.7.4 Other Evaluations

The following items were analyzed in the efficacy analysis set.

(1) Other laboratory parameter (CRP)

(2) Height and body weight

9.7.5 Evaluation of Pharmacokinetics

The following analyses were performed to examine the pharmacokinetics of TA-650 in the pharmacokinetic analysis set. Detailed analysis method is described in the pharmacokinetic analysis plan. The pharmacokinetic analysis plan was locked immediately after determination of the handling of pharmacokinetic-related data after the clinical conference.

(1) Endpoints

- Serum infliximab concentration (serum TA-650 concentration)
- ATI

(3) Timing of evaluation

Evaluation was performed before administration at Week 0, 1 hour after completion of administration at Week 0, before administration at Week 2, before administration at Week 6, at Week 8, at Week 10, before administration at Week 14, 1 hour after completion of administration at Week 14, at Week 18, before administration at Week 22, 1 hour after completion of administration at Week 22, at Week 26 and at Week 30.

(4) Analysis method

Summary statistics for the serum infliximab concentration at each evaluation point are listed.

Frequency distribution and proportion of ATI determination (negative, positive, inconclusive) are listed, and summary statistics of serum infliximab concentration at each evaluation point are listed by ATI determination results.

9.7.6 Determination of Sample Size

Target number of patients: 20 patients treated with the study drug

[Rationale for Setting]

Ulcerative colitis is designated as a specific disease, and a medical care certificate for patients with a specific disease was issued to approximately 110,000 patients in fiscal 2011 when this study was planned. Among these patients, the number of "pediatric" patients with "moderate to severe" ulcerative colitis, the target of this clinical study, is estimated to be approximately 1200. Since these patients included those who might be able to maintain remission by existing therapies and those with indications for surgical procedures such as failure to thrive, undernutrition, and worsened QOL specific to children, it was considered that patients actually eligible for the study would be even fewer, and the number of patients accumulated for this study was limited. Consequently, the target number of patients was set as 20 patients in terms of feasibility. In face-to-face advice from the Pharmaceuticals and Medical Devices Agency regarding this study, it had been recommended to examine the efficacy, safety and pharmacokinetics of the study drug comprehensively since the study would be conducted on a limited number of patients and it would be difficult to establish a certain achievement criteria for evaluation of efficacy and safety, and also recommended to give consideration so as not to create an imbalance to a certain age group among the target patient ages ranging from 6 years to 17 years, included both childhood (6 to 11 years old) and young adults (12 to 17 years old). Therefore, it was decided to accumulate a certain number of patients in both age groups.

Conducting this study on 20 patients was considered to allow for both efficacy and the safety evaluation as described below. Pharmacokinetic evaluation was conducted in the collected number of patients.

◆Efficacy

In the domestic clinical study of TA-650 in adult patients with ulcerative colitis, the CAI score-based remission rate at Week 8 (patients having a CAI score of not less than 7 and a score for blood in stools of not less than 2 at the time of registration among FAS) was 38.5% (30/78). To replicate this result in 20 patients, a 95%CI for this result would be as follows:

• 95%CI of CAI score-based remission rate (38.5%): 20.7% - 59.9%.

◆Safety

One of the ADRs of TA-650 to be noted was assumed to be infections. In domestic clinical studies in adult patients with ulcerative colitis, the incidence rate of infections (ADRs) was 18.3% (19/104). Assuming that the incidence rate of infections in adults is the same as that in children, an infection would be detected in one patient out of the target number of patients (20 patients) at a probability of 98.2%, and therefore the selected number of patients would allow for evaluation of the risk of infections of the study drug.

9.8 Changes in the Conduct of the Study or Planned Analyses

9.8.1 Revision of the Protocol

The study protocol has been revised twice since the start of this study. In the first revision, the principal changes were the duration of the follow-up of AEs, treatment to expression of malignancy and clarification of the procedure for reporting pregnancy and correction of erroneous descriptions. The principal change in the second revision was the addition of the procedure for tuberculosis testing. The study protocol (Ver. 02.00.00000, prepared on May 22, 2013) is attached in Appendix 16.1.1a, and the list of changes is attached in Appendix 16.1.1b.

9.8.2 Revision of Analysis Plans

The statistical analysis plan and the pharmacokinetic analysis plan were fixed respectively on December 15, 2014 (ver. 3.0) and January 19, 2015 (Ver. 1.0) before the database was fixed, and both plans are described in "9.7.1 Statistical and Analytical Plans."

10. Study Patients

10.1 Disposition of Patients

The disposition of patients in this study is shown in Figure 10.1–1, and the reasons for discontinuation are shown in Table 10.1–1.

In this study, informed consent was obtained from legal representatives of patients. Informed consent from legal representatives was obtained from 30 patients, and among these patients, 21 patients were registered and started to receive the study drug. Since one patient discontinued the study before CAI score evaluation at Week 8, 20 patients underwent CAI score evaluation at Week 8. Among the patients who underwent CAI score evaluation at Week 8, 18 patients were CAI score-based responders. Among these responders, 14 patients completed the study period, and 4 discontinued the study during the study period. The reasons for discontinuation were an occurrence of an AE in one patient, insufficient response to the study drug in one patient, and worsened primary disease in 2 patients. Two CAI score-based non-responders were observed, and both of them completed the evaluation at Week 14.

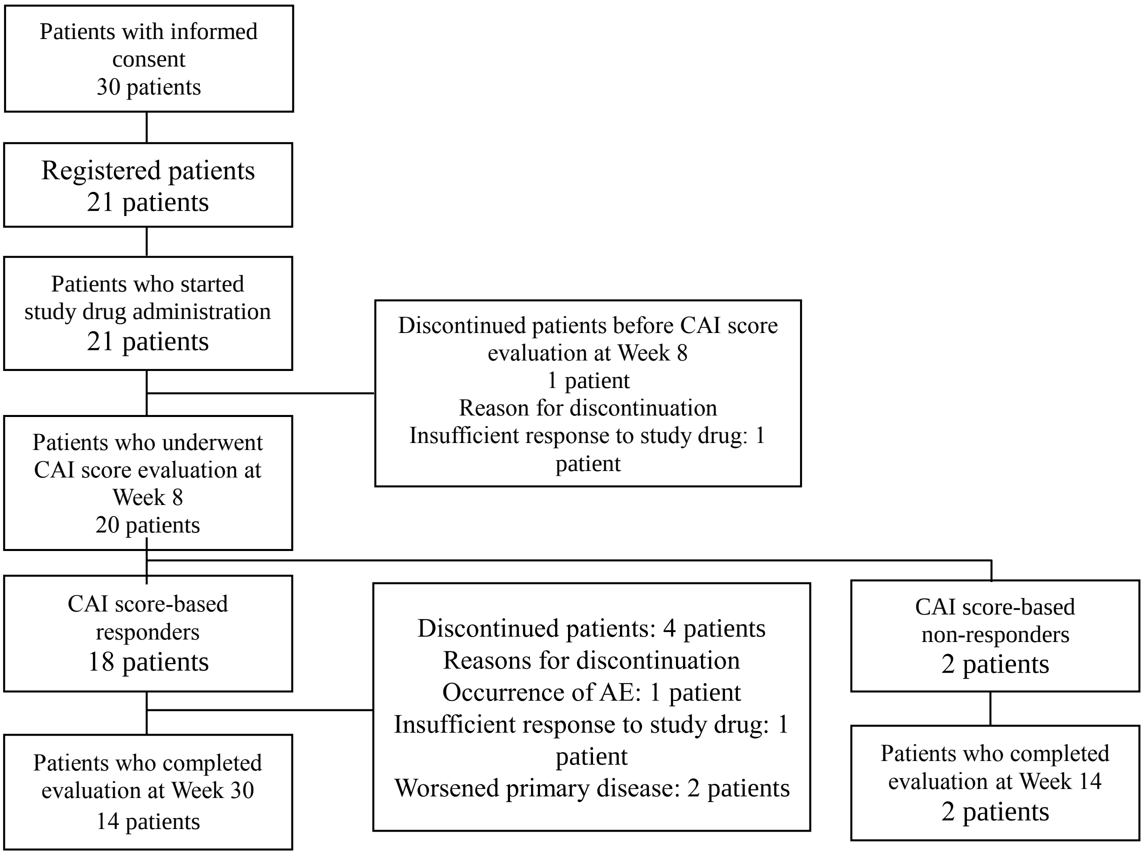

Figure 10.1–1 Disposition of Patients

Table 10.1–1 Reasons for Discontinuation

				n
Patients who received the study drug			Occurrence of AE	1
			Insufficient response	2
			Worsened primary disease	2
			Others	0
	Patients who underwent CAI score evaluation at Week 8		Occurrence of AE	1
			Insufficient response	1
			Worsened primary disease	2
			Others	0
		CAI score-based responders	Occurrence of AE	1
			Insufficient response	1
			Worsened primary disease	2
			Others	0
		CAI score-based non-responders	Occurrence of AE	0
			Insufficient response	0
			Worsened primary disease	0
			Others	0
	Patients who discontinued the study before CAI score evaluation at Week 8		Occurrence of AE	0
			Insufficient response	1
			Worsened primary disease	0
			Others	0

10.2 Protocol Deviations

Protocol deviations were categorized into the following 5 groups.

A: Patients who did not satisfy the inclusion criteria but were enrolled in the study

B: Patients who met any of the discontinuation criteria during the study period but did not discontinue the treatment

C: Patients who received inappropriate treatment or dose

D: Patients receiving any prohibited concomitant drug or therapy

E: Others

A list of all patients with deviations is provided in Appendix 16.2.2. There were 28 protocol deviations. No significant protocol deviations were observed.

11. Efficacy Evaluation

11.1 Data Sets Analyzed

Handling of the data obtained from 21 patients treated with the study drug was decided at the clinical conference held on December 15, 2014. The minutes of the clinical conference are provided in Appendix 16.1.13g. The data obtained from all of the patients treated with the study drug were used as the data set.

The efficacy analysis set was defined as the FAS excluding patients with a disease other than the target disease, patients who had never received the study drug during the evaluation period, and patients from whom no efficacy data were obtained during the evaluation period, and all of 21 patients who received the study drug were included in the efficacy analysis set. The pharmacokinetic analysis set included 21 patients from whom data for serum infliximab concentration or ATI were obtained at least once after administration among the patients who had received the study drug at least once.

11.2 Demographic and Other Baseline Characteristics

Table 11.2–1 shows the characteristics of patients who were included in the efficacy analysis set (FAS).

The proportions of male and female pediatric patients were respectively 52.4% (11/21) and 47.6% (10/21), showing similar proportions between male and female patients. The median age at the time of obtainment of consent was 14.0 years and as many as 81.0% (17/21) fell within the range from 12 to 17 years, and the median height and median body weight were respectively 158.0 cm and 45.40 kg. The median BMI was 17.78 kg/m^2, and 61.9% (13/21) of patients fell within the range of less than 18.5 kg/m^2. The median duration of disease was 2.10 years, and approximately 80% of the patients had experienced the disease for less than 3 years. In terms of extent of disease, 95.2% (20/21) patients had total colitis, and the most common clinical severity and clinical course were moderate and the relapse-remitting type respectively, each observed in 76.2% (16/21) of patients. In terms of drugs/therapies which had been used for treatment of ulcerative colitis, whether patients were non-responders to cyclosporine, tacrolimus or cytapheresis or not was assessed, and 9.5% (2.21) of patients were non-responders to cytapheresis, while no non-responders to cyclosporine or tacrolimus were found. In addition, no patients had experienced ulcerative colitis-related segmental resection or treatment, or surgical procedures (excluding intestinal resection).

In terms of resistance to existing therapies, 81.0% (17/21) did not respond to steroids (past or at the time of registration), and among these patients, patients who did not respond to steroids (failure of steroid withdrawal) accounted for the largest portion of 76.2% (16/21), while no patients who did not respond to steroids (severe) and no patients who were intolerant to steroids (past) were found. In addition, 47.6% (10/21) of patients did not respond to 6-mercaptopurine or azathioprine (past or at registration), while patients who were intolerant to 6-mercaptopurine or azathioprine (past) were only 4.8% (1/21), showing that few patients fell into this category.

In terms of concomitant drugs for the treatment of ulcerative colitis, aminosalicylates (oral preparations) were most commonly used in 90.5% (19/21) of patients, while steroids (oral preparations) and immunomodulators (azathioprine or 6-mercaptopurine) were respectively used in 57.1% (12/21) and 42.9% (9/21) of patients.

Table 11.2–1 Patient Characteristics (FAS)

Number of patients			(N=21)
Sex	n(%)	Male	11(52.4)
		Female	10(47.6)
Age (year)	n(%)	≥6, <12	4(19.0)
		≥12, ≤17	17(81.0)
	n		21
	Mean ± SD		13.7 ± 2.1
	Median		14.0
	[Q1 ,Q3]		12.0,15.0]
	Min-Max		10-17
Height (cm)	n(%)	<150	5(23.8)
		≥150, <160	8(38.1)
		≥160	8(38.1)
	n		21
	Mean ± SD		156.5 ± 10.1
	Median		158.0
	[Q1 ,Q3]		150.0,162.0]
	Min-Max		135-170
Body weight (kg)	n(%)	<40	5(23.8)
		≥40, <50	9(42.9)
		≥50	7(33.3)
	n		21
	Mean ± SD		44.79 ± 10.73
	Median		45.40
	[Q1 ,Q3]		40.00,54.50]
	Min-Max		25.6-62.6
BMI (kg/m^2)	n(%)	<18.5	13(61.9)
		≥18.5, <25.0	8(38.1)
		≥25.0	0
	n		21
	Mean ± SD		18.04 ± 2.86
	Median 18.40		17.78
	[Q1 ,Q3]		15.71,19.55]
	Min-Max		14.0-24.2
History of allergy	n(%)	Absent	8(38.1)
		Present	13(61.9)
Complications (excluding ulcerative colitis complications)	n(%)	Absent	6(28.6)
		Present	15(71.4)
Duration of disease (year)	n(%)	<1	4(19.0)
		≥1, <3	13(61.9)
		≥3, <5	2(9.5)
		≥5	2(9.5)
	n		21
	Mean ± SD		2.35 ± 1.64
	Median		2.10
	[Q1 ,Q3]		1.10,2.80]
	Min-Max		0.6-6.4
Extent of disease	n(%)	Left-sided colitis	1(4.8)
		Total colitis	20(95.2)
Clinical severity	n(%)	Mild	3(14.3)
		Moderate	16(76.2)
		Severe	2(9.5)
Clinical course	n(%)	Relapse-remitting type	16(76.2)
		Chronic continuous type	4(19.0)
		Acute fulminant (sudden onset) form	0
		First attack form	1(4.8)
Drug/therapy which had been previously used for treatment of ulcerative colitis	n(%)	Non-responder to cyclosporine	0
		Non-responder to tacrolimus	0
		Non-responder to cytapheresis	2(9.5)

Table 11.2–1 Patient Characteristics (FAS) (Continued)

Number of patients			(N=21)
UC related complications (extra-intestinal manifestations)	n(%)	Absent<br>Present	14(66.7)<br>7(33.3)
Previous segmental resection	n(%)	Absent<br>Present	21(100.0)<br>0
Other UC-related GI surgical procedures (excluding intestinal resection)	n(%)	Absent<br>Present	21(100.0)<br>0
Patients who did not respond to 6-mercaptopurine or azathioprine (at registration)*1	n(%)	Not applicable<br>Applicable	12(57.1)<br>9(42.9)
Patients who did not respond to 6-mercaptopurine or azathioprine (past) *2	n(%)	Not applicable<br>Applicable	16(76.2)<br>5(23.8)
Patients who did not respond to 6-mercaptopurine or azathioprine (past or at registration)*3	n(%)	Not applicable<br>Applicable	11(52.4)<br>10(47.6)
Patients who were intolerant to 6-mercaptopurine or azathioprine (past)*4	n(%)	Not applicable<br>Applicable	20(95.2)<br>1(4.8)
Patients who did not respond to steroids (at registration)*5	n(%)	Not applicable<br>Applicable	18(85.7)<br>3(14.3)
Patients who did not respond to steroids (failure of steroid withdrawal) (past or at registration)*6	n(%)	Not applicable<br>Applicable	5(23.8)<br>16(76.2)
Patients who did not respond to steroids (severe) (past or at registration)*7	n(%)	Not applicable<br>Applicable	21(100.0)<br>0
Patients who did not respond to steroids (past or at registration)*8	n(%)	Not applicable<br>Applicable	4(19.0)<br>17(81.0)
Patients who were intolerant to steroids (past)*9	n(%)	Not applicable<br>Applicable	21(100.0)<br>0
Concomitant drug (Steroids, oral)	n(%)	Absent<br>Present	9(42.9)<br>12(57.1)
Concomitant drug (Aminosalicylates, oral)	n(%)	Absent<br>Present	2(9.5)<br>19(90.5)
Concomitant drug (5-aminosalicylate preparations, oral)	n(%)	Absent<br>Present	4(19.0)<br>17(81.0)
Concomitant drug (Sulfasalazine preparation, oral)	n(%)	Absent<br>Present	18(85.7)<br>3(14.3)
Concomitant drug (Azathioprine, oral)	n(%)	Absent<br>Present	13(61.9)<br>8(38.1)
Concomitant drug (6-mercaptopurine, oral)	n(%)	Absent<br>Present	20(95.2)<br>1(4.8)
Concomitant drug (Immunomodulators [Azathioprine or 6-mercaptopurine])	n(%)	Absent<br>Present	12(57.1)<br>9(42.9)
IL-6 (pg/mL)	n(%)	<2<br>≥2, <5<br>≥5	9(42.9)<br>4(19.0)<br>8(38.1)
	n<br>Mean ± SD<br>Median<br>[Q1 ,Q3]<br>Min-Max		21<br>9.6063 ± 21.5610<br>2.5200<br>[1.2600,7.9600]<br>0.000-99.900
TNFα (pg/mL)	n(%)<br>0.55	<0.55<br>≥0.55, <1.0<br>≥1.0, <1.5<br>≥1.5	9(42.9)<br>1(4.8)<br>5(23.8)<br>6(28.6)
	n<br>Mean ± SD<br>Median<br>[Q1 ,Q3]<br>Min-Max		21<br>0.998 ± 1.080<br>1.020<br>[0.000,1.640]<br>0.00-3.41

*1: For at least 12 weeks before the starting day of the screening period, a patient had received 6-mercaptopurine or azathioprine, and had received at a stable dose for at least 4 weeks before the starting day of the screening period, but had an inadequate response.
*2: Within 5 years before the starting day of the screening period, a patient had received 6-mercaptopurine or azathioprine for at least 12 weeks but had an inadequate response.
*3: Patients who were categorized into either of those who did not respond to 6-mercaptopurine or azathioprine (past) or (at the time of registration).
*4: Within 5 years before the starting day of the screening period, a patient had received 6-mercaptopurine or azathioprine, and experienced an adverse drug reaction that made it difficult to continue treatment.
*5: For at least 2 weeks before the starting day of the screening period, a patient had received steroids at a dose of at least 1 mg/kg/day or 20 mg/day calculated on a prednisolone basis, but had an inadequate response.
*6: Within 18 months before the starting day of the screening period, a patient had experienced an exacerbation or relapse of the primary disease in response to a reduction in the steroid dose, and had difficulty in steroid withdraw.
*7: Within 18 months before the starting day of the screening period, a patient had used a steroid, but had an inadequate response to oral or intravenous steroid at a dose of at least 1 mg/kg/day or at least 40 mg/day calculated on prednisolone basis for at least 2 weeks for the former or at least 1 week for the latter).
*8: Patients who were categorized into either of those who did not respond to steroids (at the time of registration), those who did not respond to steroids (failure of steroid withdrawal) (past or at the time of registration), or those who did not respond to steroids (severe) (past or at the time of registration).
*9: Within 18 months before the starting day of the screening period, a patient had used steroids and experienced an adverse drug reaction that made it difficult to continue treatment.

The baseline data of individual endpoints (FAS) are shown in Table 11.2–2.

The CAI score (Mean ± SD) at the time of registration was 9.7 ± 2.7 points, and patients having a CAI score of 7 to 8 points, 9 to 10 points and not less than 11 points were respectively 42.9% (9/21), 28.6% (6/21) and 28.6% (6/21).The partial Mayo score (Mean ± SD) was 5.6 ± 1.6 points, and patients having a partial Mayo score of 4 to 6 points accounted for the largest percentage at 57.1% (12/21). The Mayo score (Mean ± SD) determined only in 8 patients who were able to undergo sigmoidoscopy was 7.0 ± 2.4, and moderate (Mayo score ranging from 6 to 10 points) and severe (Mayo score ranging from 11 to 12 points) conditions assessed based on the Mayo score were observed respectively in 50.0% (4/8) and 12.5% (1/8) patients. The PUCAI score (Mean ± SD) was 47.1 ± 15.2 points, and patients having a PUCAI score between 31 and 60 accounted for the largest percentage at 66.7% (14/21).

At the time of registration, 57.1% (12/21) of patients were using steroids, and all of these patients received steroids at a dose of not more than 1 mg/kg/day calculated on prednisolone basis. The median CRP was 0.20 mg/dL.

Table 11.2–2 Baseline Data of Endpoints (FAS)

Number of patients			(N=21)
CAI score	n(%)	7-8	9(42.9)
		9-10	6(28.6)
		≥11	6(28.6)
	n		21
	Mean ± SD		9.7 ± 2.7
	Median		9.0
	[Q1 ,Q3]		[8.0,11.0]
	Min-Max		7-16
CAI 1) Number of stools per week	n(%)	0	3(14.3)
		1	8(38.1)
		2	6(28.6)
		3	4(19.0)
CAI 2) Blood in stools (based on weekly average)	n(%)	0	0
		2	5(23.8)
		4	16(76.2)
CAI 3) Investigator's global assessment of symptomatic state	n(%)	0	0
		1	3(14.3)
		2	16(76.2)
		3	2(9.5)
CAI 4) Abdominal pain	n(%)	0	1(4.8)
		1	9(42.9)
		2	10(47.6)
		3	1(4.8)
CAI 5) Temperature elevation due to ulcerative colitis	n(%)	0	21(100.0)
		3	0
CAI 6) Extraintestinal manifestations	n(%)	0	18(85.7)
		3	3(14.3)
		6	0
		9	0
CAI 7) Laboratory findings	n(%)	0	13(61.9)
		1	5(23.8)
		2	1(4.8)
		4	2(9.5)

Table 11.2–2 Baseline Data of Endpoints (FAS) (Continued)

Number of patients				(N=21)
Partial Mayo score		n(%)	0-3	3(14.3)
			4-6	12(57.1)
			7-9	6(28.6)
		n		21
		Mean ± SD		5.6 ± 1.6
		Median		6.0
		[Q1 ,Q3]		[5.0,7.0]
		Min-Max		2-8
Mayo score (Excluding CAI score-based non-responders at Week 8)		n(%)	0-5	3(37.5)
			6-8	3(37.5)
			9-10	1(12.5)
			11-12	1(12.5)
		n		8
		Mean ± SD		7.0 ± 2.4
		Median		7.0
		[Q1 ,Q3]		[5.0,8.5]
		Min-Max		4-11
	Mayo 1) Stool frequency	n(%)	0	2(9.5)
			1	6(28.6)
			2	4(19.0)
			3	9(42.9)
	Mayo 2) Rectal bleeding	n(%)	0	2(9.5)
			1	3(14.3)
			2	16(76.2)
			3	0
	Mayo 3) Physician’s global assessment	n(%)	0	0
			1	3(14.3)
			2	16(76.2)
			3	2(9.5)
	Mayo 4) Findings of endoscopy (Excluding CAI score-based non-responders at Week 8)	n(%)	0	0
			1	1(12.5)
			2	6(75.0)
			3	1(12.5)

Table 11.2–2 Baseline Data of Endpoints (FAS) (Continued)

Number of patients				(N=21)
PUCAI score		n(%)	0-30	3(14.3)
			31-60	14(66.7)
			61-85	4(19.0)
		n		21
		Mean ± SD		47.1 ± 15.2
		Median		45.0
		[Q1 ,Q3]		[40.0,60.0]
		Min-Max		15-70
	PUCAI 1) Abdominal pain	n(%)	0	1(4.8)
			5	18(85.7)
			10	2(9.5)
	PUCAI 2) Rectal bleeding	n(%)	0	2(9.5)
			10	3(14.3)
			20	14(66.7)
			30	2(9.5)
	PUCAI 3) Stool consistency	n(%)	0	0
			5	14(66.7)
			10	7(33.3)
	PUCAI 4) Stool frequency per 24 hours	n(%)	0	1(4.8)
			5	11(52.4)
			10	4(19.0)
			15	5(23.8)
	PUCAI 5) Nocturnal stools (any episode causing wakening)	n(%)	0	11(52.4)
			10	10(47.6)
	PUCAI 6) Activity level	n(%)	0	3(14.3)
			5	16(76.2)
			10	2(9.5)
Steroid dose (mg/kg/day) (calculated on prednisolone basis)		n(%)	Absent	9(42.9)
			≤1	12(57.1)
			>1	0
		n		12
		Mean ± SD		0.25 ± 0.23
		Median		0.20
		[Q1 ,Q3]		[0.10,0.32]
		Min-Max		0.0-0.9
CRP (mg/dL)		n(%)	≤0.5	14(66.7)
			>0.5	7(33.3)
		n		21
		Mean ± SD		1.75 ± 3.68
		Median		0.20
		[Q1 ,Q3]		[0.00,0.60]
		Min-Max		0.0-15.1

11.3 Measurements of Treatment Compliance

The treatment compliance of individual patients is provided in Appendix 16.2.8f.

In the patient with patient ID code of TA-650UC-022-01, calculation of a dose of the study drug at Week 6 was incorrect due to a wrong conversion base for body weight, and 220 mg (97.78% of the specified dose) of the study drug was administered. However, since not less than 75% of the specified

dose was administered to the patient, it was judged that this deviation would not cause any problems in evaluation of efficacy and pharmacokinetics of the study drug.

In the patient with a patient ID code of TA-650UC-025-01, the duration of administration of the study drug at the starting day of study drug administration (Week 0) was 1 hour 52 minutes, which was shorter than the duration specified in the protocol (at least 2 hours). However, since the total amount of the specified dose was administered, it was judged that this deviation would not cause any problems in evaluation of efficacy of the study drug. In addition, no AEs were observed in this patient on the day of administration, therefore it was judged that this deviation would not cause any problems in evaluation of safety.

In the patient with patient ID code of TA-650UC-013-01, the day of study drug administration at Week 6 fell outside the permissible time range for study drug administration (Week 6: ±3 days) specified in the protocol. The study drug was administered 6 days after the specified day and the deviation from the specified day was not large, therefore it was judged that this deviation would not cause any problems in evaluation of efficacy.

11.4 Efficacy Results and Tabulations of Individual Patient Data

11.4.1 Analysis of Efficacy

The results of tabulation based on the data employed at each evaluation point were provided for each efficacy endpoint. In case data to be obtained by evaluation at Week 30 is missing due to discontinued patients and CAI score-based non-responders, the results of tabulation based on LOCF data obtained by using the values observed at the last evaluation point immediately before the missing point were provided as the value for the last time point.

When the data at each time point until Week 30 were missing, the results of tabulation after imputation were also provided. The data at a missing evaluation time point were first imputed by the LOCF approach using the values observed at the last evaluation point immediately before the missing point. Subsequently, for the evaluation points after the time point where the relevant patient met the conditions of TF, missing data were imputed with the data satisfying the rules for TF (see 9.7.1.2.2.5 Treatment Failure). Five patients met the conditions of TF, and all of these patients fell under the category of “Patients who discontinued the study due to exacerbation of the primary disease or inadequate response to the study drug,” and one out of the five patients also fell under the category of “Patients who experienced the start of administration or increase in a dose of steroids (oral preparations, injection, enema, and suppository) due to exacerbation of the primary disease, etc.,” Detailed information about the patients of TF is described in the minutes of clinical conference (see Appendix 16.1.13g for details).

11.4.1.1 Efficacy in Improving Symptoms

11.4.1.1.1 CAI Score

The CAI score is an activity index to evaluate disease activity, which is calculated as the sum (0 to 29 points) of subscores for 7 clinical conditions, consisting of the number of stools per week, blood in stools (based on weekly average), investigator’s global assessment of the symptomatic state, abdominal pain/cramps, temperature elevation due to ulcerative colitis, extraintestinal manifestations, and laboratory findings (hemoglobin or ESR), and a higher score indicates greater disease activity. In

addition, a difference calculated by subtracting the CAI score at the time of registration from the CAI score on each evaluation day was defined as a CAI score change.

Summary statistics of the CAI score and CAI score change at each evaluation point are shown in Table 11.4–1, and the time course of CAI score is shown graphically in Figure 11.4–1. Summary statistics and the time course based on data obtained by TF imputation are also respectively shown in Table 11.4–2 and Figure 11.4–2.

The CAI score (Mean ± SD) was 9.7 ± 2.7 at the time of registration and then decreased respectively over time to: 4.0 ± 3.2, 3.5 ± 3.5 and 3.2 ± 3.5 at Weeks 2, 6 and 8. Subsequently, the CAI score ranged from 2.5 ± 2.3 to 3.5 ± 2.2 during the period from Week 10 to Week 30, and was 5.6 ± 3.8 at the last time point. The CAI score changes (Mean ± SD) were -5.5 ± 2.7, -6.0 ± 4.0 and -6.2 ± 3.7 at Weeks 2, 6 and 8 respectively, indicating an increase in the degree of score change with time, and the score changes ranged from -5.9 ± 3.8 to -6.9 ± 3.8 during the period from Week 10 to Week 30, and were -4.1 ± 4.2 at the last time point. Both the CAI score and CAI score change showed the effect of the study drug in improving symptoms from Week 2, and the effect was maintained up to Week 30.

When using the data obtained by TF imputation, the CAI scores (Mean ± SD) at Weeks 2, 6 and 8 were 4.5 ± 3.9, 4.0 ± 4.3 and 3.8 ± 4.3 respectively, indicating a decreasing tendency, and the CAI score ranged from 4.1 ± 4.6 to 5.8 ± 4.1 during the period from Week 10 to Week 30. The CAI score changes (Mean ± SD) (TF) were -5.2 ± 2.9, -5.7 ± 4.2 and -5.9 ± 3.8 at Weeks 2, 6 and 8 respectively, indicating an increase in the degree of score change, and ranged from -3.9 ± 4.3 to -5.5 ± 4.4 during the period from Week 10 to Week 30. Both the CAI score and CAI score change showed similar tendencies to the data obtained before TF imputation.

Table 11.4–1 Summary Statistics of CAI Score and CAI Score Change (FAS)

	CAI score						CAI score change								
	n	Mean	SD	Median	Q1	Q3	Mean	SD	Median	Q1	Q3	Min	Max	Lower limit of 95% CI	Upper limit of 95% CI
At registration	21	9.7	2.7	9.0	8.0	11.0	-	-	-	-	-	-	-	-	-
Week 2	20	4.0	3.2	4.0	1.0	5.5	-5.5	2.7	-6.0	-7.0	-4.0	-10	0	-6.7	-4.2
Week 6	20	3.5	3.5	3.0	0.5	4.0	-6.0	4.0	-5.5	-8.0	-3.5	-13	3	-7.8	-4.1
Week 8	20	3.2	3.5	3.0	0.5	3.5	-6.2	3.7	-6.0	-8.0	-5.0	-13	2	-7.9	-4.5
Week 10	18	2.7	2.9	3.0	0.0	3.0	-6.5	3.9	-6.0	-10.0	-4.0	-13	1	-8.4	-4.6
Week 14	16	2.7	3.0	2.0	0.0	3.5	-6.6	4.3	-6.5	-9.5	-5.0	-13	2	-8.9	-4.4
Week 18	16	2.6	2.6	2.5	0.0	3.0	-6.8	4.0	-6.0	-10.0	-4.5	-13	1	-8.9	-4.6
Week 22	14	3.3	3.1	3.0	0.0	5.0	-6.1	4.6	-5.5	-10.0	-3.0	-13	2	-8.8	-3.5
Week 26	14	2.5	2.3	2.5	1.0	4.0	-6.9	3.8	-6.5	-10.0	-5.0	-14	1	-9.1	-4.7
Week 30	14	3.5	2.2	3.0	2.0	5.0	-5.9	3.8	-5.5	-8.0	-3.0	-14	0	-8.1	-3.8
Last	21	5.6	3.8	5.0	3.0	7.0	-4.1	4.2	-3.0	-6.0	-1.0	-14	2	-6.0	-2.2

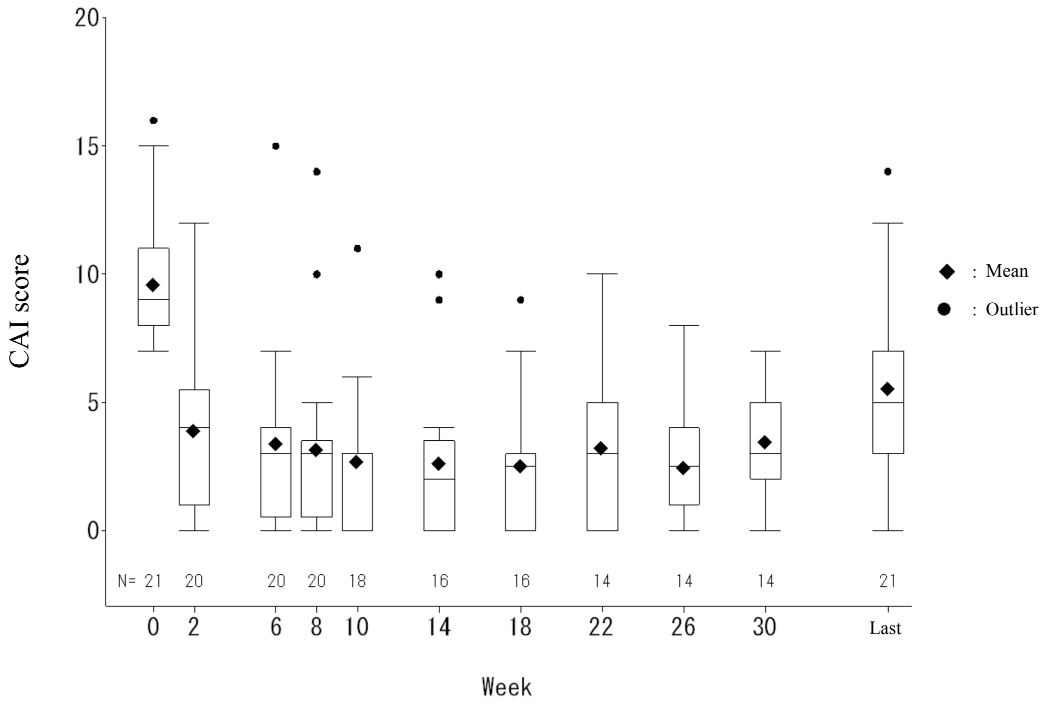

*An outlier is an observation outside the range which is 1.5 times the interquartile range. The lower end of the box, a horizontal line inside the box and the upper end of box are respectively Q1, Median and Q3. The upper and lower ends of the whisker are respectively minimum and maximum values excluding the outliers.

Figure 11.4–1 Time Course of CAI Score (FAS)

Table 11.4–2 Summary Statistics of CAI Score and CAI Score Change (TF) (FAS)

	CAI score						CAI score change (TF)								
	n	Mean	SD	Median	Q1	Q3	Mean	SD	Median	Q1	Q3	Min	Max	Lower limit of 95% CI	Upper limit of 95% CI
At registration	21	9.7	2.7	9.0	8.0	11.0	-	-	-	-	-	-	-	-	-
Week 2	21	4.5	3.9	4.0	1.0	6.0	-5.2	2.9	-6.0	-7.0	-4.0	-10	0	-6.5	-3.9
Week 6	21	4.0	4.3	3.0	1.0	4.0	-5.7	4.2	-5.0	-8.0	-3.0	-13	3	-7.6	-3.8
Week 8	21	3.8	4.3	3.0	1.0	4.0	-5.9	3.8	-6.0	-8.0	-5.0	-13	2	-7.6	-4.2
Week 10	21	4.1	4.6	3.0	0.0	6.0	-5.5	4.4	-6.0	-8.0	-2.0	-13	2	-7.5	-3.5
Week 14	21	4.7	4.8	3.0	1.0	9.0	-5.0	4.8	-6.0	-8.0	0.0	-13	2	-7.1	-2.8
Week 18	21	4.6	4.6	3.0	2.0	7.0	-5.1	4.6	-5.0	-8.0	0.0	-13	2	-7.2	-3.0
Week 22	21	5.7	4.6	5.0	3.0	9.0	-4.0	4.9	-3.0	-7.0	0.0	-13	2	-6.2	-1.8
Week 26	21	5.1	4.6	4.0	1.0	8.0	-4.5	4.7	-5.0	-8.0	0.0	-14	2	-6.7	-2.4
Week 30	21	5.8	4.1	5.0	3.0	8.0	-3.9	4.3	-3.0	-6.0	0.0	-14	2	-5.8	-1.9

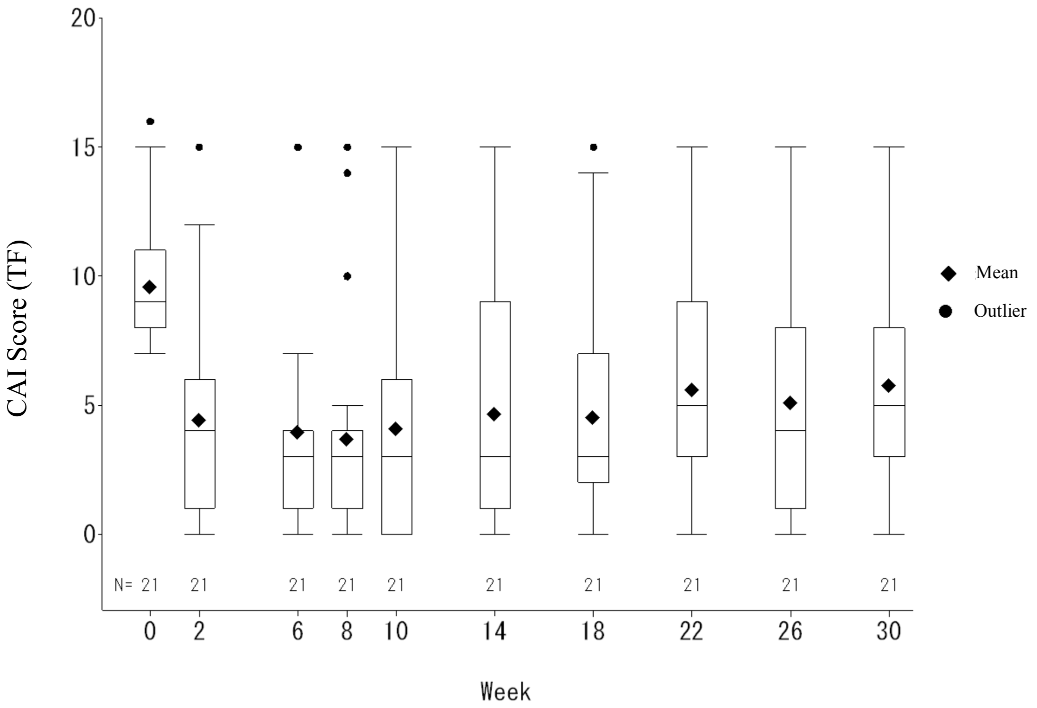

*An outlier is an observation outside the range which is 1.5 times the interquartile range. The lower end of the box, a horizontal line inside the box and the upper end of box are respectively Q1Median and Q3. The upper and lower ends of the whisker are respectively minimum and maximum values excluding the outliers.

Figure 11.4–2 Time Course of CAI Score (TF) (FAS)

The CAI score-based remission was defined as the case where a CAI score was not more than 4 on the evaluation day excluding the evaluation at the time of registration. The CAI score-based remission rates at each evaluation point are shown in Table 11.4–3. The CAI score-based remission rates calculated based on the data obtained by TF imputation are shown in Table 11.4–4.

The CAI score-based remission rate was 60.0% (12/20) at Week 2, and was increased to 80.0% (16/20) at both Weeks 6 and 8. Afterwards, the CAI score-based remission rate ranged from 64.3% to 87.5% during the period from Week 10 to Week 30, and was 42.9% (9/21) at the last time point. Based on the results of CAI score-based remission rate, the administration of TA-650 was observed to render the effect of leading at least 60% of the patients to achieve remission from Week 2, and the effect of the drug was confirmed to last up to Week 30.

When TF imputation was performed, the CAI score-based remission rates were 57.1% (12/21) at Week 2, and 76.2% (16/21) at both Weeks 6 and 8, and then ranged from 42.9% to 66.7% during the period from Week 10 to Week 30. The data obtained by TF imputation was confirmed to show a similar tendency to the data obtained before TF in that administerring the drug had the effect of leading patients to remission from Week 2 and the effect lasted.

Table 11.4–3 CAI Score-Based Remission Rate (%) (FAS)

	CAI score		
	n	Remission	%
Week 2	20	12	60.0
Week 6	20	16	80.0
Week 8	20	16	80.0
Week 10	18	14	77.8
Week 14	16	14	87.5
Week 18	16	14	87.5
Week 22	14	9	64.3
Week 26	14	12	85.7
Week 30	14	9	64.3
Last	21	9	42.9

Table 11.4–4 CAI Score-Based Remission Rate (%) (TF) (FAS)

	CAI score (TF)		
	n	Remission	%
Week 2	21	12	57.1
Week 6	21	16	76.2
Week 8	21	16	76.2
Week 10	21	14	66.7
Week 14	21	14	66.7
Week 18	21	14	66.7
Week 22	21	9	42.9
Week 26	21	12	57.1
Week 30	21	9	42.9

11.4.1.1.2 Partial Mayo Score

The partial Mayo score is an activity index to evaluate disease activity, which is calculated as the sum (0 to 9 points) of subscores for 3 clinical conditions, consisting of stool frequency, rectal bleeding and physician’s global assessment, and a higher score indicates greater disease activity. In addition, a difference calculated by subtracting the partial Mayo score at the time of registration from a partial Mayo score on each evaluation day was defined as a partial Mayo score change.

Summary statistics of partial Mayo score and partial Mayo score change at each evaluation point were shown in Table 11.4–5. Summary statistics of partial Mayo score and partial Mayo score change based on data obtained by TF imputation were shown in Table 11.4–6.

The partial Mayo score (Mean ± SD) was 5.6 ± 1.6 at the time of registration and decreased over time respectively to: 2.6 ± 2.3, 2.2 ± 2.0 and 1.7 ± 1.7 at Weeks 2, 6 and 8. Subsequently, the score ranged from 1.8 ± 1.7 to 2.8 ± 1.9 during the period from Week 10 to Week 30, and was 3.7 ± 2.2 at the last time point. The partial Mayo score changes (Mean ± SD) at Weeks 2, 6 and 8 were respectively -2.9 ± 2.2, -3.3 ± 2.2 and -3.8 ± 1.7, indicating an increase in degree of score change with time. The partial Mayo score change ranged from -2.6 ± 2.8 to -3.6 ± 2.5 during the period from Week 10 to Week 30, and was -1.9 ± 2.7 at the last time point. As shown above, both the partial Mayo score and partial Mayo score change showed the effect of the study drug in improving symptoms from Week 2, and the effect lasted up to Week 30.

When using the data obtained by TF imputation, the partial Mayo scores (Mean ± SD) at Weeks 2, 6 and 8 were respectively 2.9 ± 2.6, 2.5 ± 2.3 and 2.0 ± 2.1, indicating a decreasing tendency, and the partial Mayo score ranged from 2.6 ± 2.3 to 3.6 ± 2.2 during the period from Week 10 to Week 30. The partial Mayo score changes (Mean ± SD) were -2.7 ± 2.2, -3.1 ± 2.3 and -3.6 ± 1.9 respectively at Weeks 2, 6 and 8, indicating an increase in the degree of score change, and ranged from -2.0 ± 2.5 to -3.0 ± 2.5 during the period from Week 10 to Week 30. Both the partial Mayo score and partial Mayo score change showed similar tendencies to the data obtained before TF imputation in that the effect of the study drug to improve symptoms was observed from Week 2 and the effect lasted up to Week 30.

Table 11.4–5 Summary Statistics of Partial Mayo Score and Partial Mayo Score Change (FAS)

	Partial Mayo score						Partial Mayo score change								
	n	Mean	SD	Median	Q1	Q3	Mean	SD	Median	Q1	Q3	Min	Max	Lower limit of 95% CI	Upper limit of 95% CI
At registration	21	5.6	1.6	6.0	5.0	7.0	-	-	-	-	-	-	-	-	-
Week 2	20	2.6	2.3	2.5	0.5	4.5	-2.9	2.2	-3.0	-4.5	-0.5	-6	0	-3.9	-1.8
Week 6	20	2.2	2.0	2.5	0.0	3.0	-3.3	2.2	-3.5	-5.0	-1.0	-7	1	-4.3	-2.2
Week 8	20	1.7	1.7	1.5	0.0	3.0	-3.8	1.7	-3.0	-5.5	-3.0	-6	0	-4.6	-2.9
Week 10	18	2.2	2.1	2.5	0.0	3.0	-3.1	2.5	-3.0	-5.0	-2.0	-6	1	-4.3	-1.9
Week 14	16	1.9	1.9	2.0	0.0	3.0	-3.6	2.5	-4.5	-5.5	-1.5	-7	1	-4.9	-2.2
Week 18	16	2.1	1.8	2.5	0.0	3.0	-3.4	2.4	-3.5	-5.5	-2.0	-6	1	-4.7	-2.2
Week 22	14	2.4	2.1	3.0	0.0	4.0	-3.0	2.7	-3.5	-5.0	0.0	-7	1	-4.6	-1.4
Week 26	14	1.8	1.7	1.5	0.0	3.0	-3.6	2.4	-3.5	-5.0	-3.0	-7	3	-5.0	-2.3
Week 30	14	2.8	1.9	3.0	2.0	4.0	-2.6	2.8	-2.5	-5.0	-1.0	-7	4	-4.3	-1.0
Last	21	3.7	2.2	4.0	3.0	4.0	-1.9	2.7	-2.0	-3.0	0.0	-7	4	-3.1	-0.7

Table 11.4–6 Summary Statistics of Partial Mayo Score and Partial Mayo Score Change (TF) (FAS)

	Partial Mayo score (TF)						Partial Mayo score change (TF)								
	n	Mean	SD	Median	Q1	Q3	Mean	SD	Median	Q1	Q3	Min	Max	Lower limit of 95% CI	Upper limit of 95% CI
At registration	21	5.6	1.6	6.0	5.0	7.0	-	-	-	-	-	-	-	-	-
Week 2	21	2.9	2.6	3.0	1.0	5.0	-2.7	2.2	-3.0	-4.0	0.0	-6	0	-3.7	-1.7
Week 6	21	2.5	2.3	3.0	0.0	3.0	-3.1	2.3	-3.0	-5.0	-1.0	-7	1	-4.1	-2.0
Week 8	21	2.0	2.1	2.0	0.0	3.0	-3.6	1.9	-3.0	-5.0	-3.0	-6	0	-4.4	-2.7
Week 10	21	2.6	2.2	3.0	1.0	4.0	-3.0	2.3	-3.0	-5.0	-2.0	-6	1	-4.0	-1.9
Week 14	21	2.6	2.3	3.0	0.0	4.0	-3.0	2.5	-3.0	-5.0	0.0	-7	1	-4.1	-1.8
Week 18	21	2.7	2.2	3.0	1.0	4.0	-2.9	2.3	-3.0	-5.0	0.0	-6	0	-4.0	-1.8
Week 22	21	3.3	2.4	4.0	1.0	5.0	-2.2	2.6	-2.0	-4.0	0.0	-7	1	-3.4	-1.1
Week 26	21	2.9	2.4	3.0	1.0	4.0	-2.7	2.5	-3.0	-4.0	0.0	-7	3	-3.8	-1.5
Week 30	21	3.6	2.2	3.0	3.0	4.0	-2.0	2.5	-2.0	-3.0	0.0	-7	4	-3.2	-0.8

11.4.1.1.3 Mayo Score

The Mayo score is an activity index to evaluate disease activity, which is calculated as the sum (0 to 12 points) of subscores for 4 clinical conditions, consisting of those for the partial Mayo score and findings of endoscopy. Since this clinical study was conducted in pediatric patients taking into consideration the burden of the examination on patients, sigmoidoscopy performed to determine a Mayo score was not included as an essential test item, but it was decided to perform this examination whenever possible. It was also decided that a Mayo score would not be determined when the endoscopic examination was able to be performed only at one time point among the time of registration, Week 30 and the day of discontinuation, since it was impossible to compare Mayo scores between before and after administration of the study drug. Due to this, only 8 patients were assessed based on the Mayo score. In addition, a difference calculated by subtracting the Mayo score at the time of registration from a Mayo score on each evaluation day was defined as a Mayo score change.

Summary statistics of Mayo score and Mayo score change at each evaluation point were shown in Table 11.4–7. Summary statistics of the Mayo score and Mayo score change based on the data obtained by TF imputation were shown in Table 11.4–8.

The Mayo score (Mean ± SD) was 7.0 ± 2.4 at the time of registration and at week 30 had decreased to 4.9 ± 2.4. The Mayo score at the last time point was 5.0 ± 2.3. The Mayo score changes (Mean ± SD) at Week 30 and the last time point were respectively -2.6 ± 3.9 and -2.0 ± 3.9.

When TF imputation was performed, the Mayo score (Mean ± SD) at Week 30 was 4.8 ± 2.3, indicating a similar tendency to that obtained before TF imputation. In addition, the Mayo score change (Mean ± SD) at Week 30 based on the data obtained by TF imputation was -2.3 ± 3.7. Both the Mayo score and Mayo score change based on the data obtained by TF imputation showed a similar tendency to that obtained before TF imputation.

Table 11.4–7 Summary Statistics of Mayo Score and Mayo Score Change (FAS)

	Mayo score						Mayo score change								
	n	Mean	SD	Median	Q1	Q3	Mean	SD	Median	Q1	Q3	Min	Max	Lower limit of 95% CI	Upper limit of 95% CI
At registration	8	7.0	2.4	7.0	5.0	8.5	-	-	-	-	-	-	-	-	-
Week 30	7	4.9	2.4	5.0	4.0	6.0	-2.6	3.9	-2.0	-7.0	-1.0	-7	4	-6.1	1.0
Last	8	5.0	2.3	5.0	4.0	6.0	-2.0	3.9	-1.5	-5.5	0.5	-7	4	-5.3	1.3

Table 11.4–8 Summary Statistics of Mayo Score and Mayo Score Change (TF) (FAS)

	Mayo score (TF)						Mayo score change (TF)								
	n	Mean	SD	Median	Q1	Q3	Mean	SD	Median	Q1	Q3	Min	Max	Lower limit of 95% CI	Upper limit of 95% CI
At registration	8	7.0	2.4	7.0	5.0	8.5	-	-	-	-	-	-	-	-	-
Week 30	8	4.8	2.3	4.5	4.0	5.5	-2.3	3.7	-1.5	-5.5	-0.5	-7	4	-5.3	0.8

Mayo score-based response was defined as the case which satisfied both of the conditions, “a Mayo score was decreased by at least 30% and by at least 3 points compared to that measured at the time of registration” and “a rectal bleeding subscore was decreased by at least 1 point compared to that measured at the time of registration or the rectal bleeding subscore was not more than 1 point” at each evaluation point. Mayo score-based remission was defined as the case where a Mayo score on each evaluation day excluding the evaluation at the time of registration was not more than 2 points and all of the 4 subscores were not more than 1 point.

The Mayo score-based response rates and Mayo score-based remission rates are shown respectively in Table 11.4–9 and Table 11.4–10. The Mayo score-based response rates and Mayo score-based remission rates based on the data obtained by TF imputation are shown respectively in Table 11.4–11 and Table 11.4–12.

The Mayo score-based response rate and Mayo score-based remission rate at Week 30 were respectively 42.9% (3/7) and 14.3% (1/7), and those at the last time point were respectively 37.5% (3/8) and 12.5% (1/8).

The Mayo score-based response rate and Mayo score-based remission rate at Week 30 based on the data obtained by TF imputation were respectively 37.5% (3/8) and 12.5% (1/8), indicating similar values to those obtained before TF imputation.

Table 11.4–9 Mayo Score-Based Response Rate (%) (FAS)

	Mayo score		
	n	Response	%
Week 30	7	3	42.9
Last	8	3	37.5

Table 11.4–10 Mayo Score-Based Remission Rate (%) (FAS)

	Mayo score		
	n	Remission	%
Week 30	7	1	14.3
Last	8	1	12.5

Table 11.4–11 Mayo Score-Based Response Rate (%) (TF) (FAS)

	Mayo score (TF)		
	n	Response	%
Week 30	8	3	37.5

Table 11.4–12 Mayo Score-Based Remission Rate (%) (TF) (FAS)

	Mayo score (TF)		
	n	Remission	%
Week 30	8	1	12.5

11.4.1.1.4 PUCAI Score

The PUCAI score is an activity index for pediatric ulcerative colitis, which is calculated as the sum (0 to 85 points) of subscores for 6 clinical conditions, consisting of abdominal pain, rectal bleeding, stool consistency, stool frequency per 24 hours, nocturnal stool, and activity level. Similar to other scores, a higher PUCAI score indicates greater disease activity. In addition, a difference calculated by subtracting the PUCAI score at the time of registration from a PUCAI score on each evaluation day was defined as the PUCAI score change.

Summary statistics of PUCAI score and PUCAI score change at each evaluation point are shown in Table 11.4–13. Summary statistics of PUCAI score and PUCAI score change based on the data obtained by TF imputation are shown in Table 11.4–14.

The PUCAI score (Mean ± SD) was 47.1 ± 15.2 at the time of registration and decreased over time to 20.3 ± 16.3, 17.3 ± 17.5 and 12.5 ± 13.5 respectively at Weeks 2, 6 and 8. The PUCAI scores ranged from 12.2 ± 12.0 to 19.3 ± 18.8 during the period from Week 10 to Week 30, and were 28.8 ± 22.5 at the last time point. The PUCAI score changes (Mean ± SD) at Weeks 2, 6 and 8 were respectively -25.8 ± 17.5, -28.8 ± 21.1 and -33.5 ± 14.0. The PUCAI score changes ranged from -26.8 ± 27.8 to -34.1 ± 17.7 during the period from Week 10 to Week 30, and were -18.3 ± 28.4 at the last time point. Both the PUCAI score and PUCAI score change showed the effect of the study drug in improving symptoms from Week 2, and the effect lasted up to Week 30.

When using the data obtained by TF imputation, the PUCAI scores (Mean ± SD) decreased to 22.6 ± 19.3, 19.8 ± 20.6 and 15.2 ± 18.2 respectively at Weeks 2, 6 and 8, and ranged from 19.3 ± 20.5 to 27.6 ± 21.9 during the period from Week 10 to Week 30. The PUCAI score changes (Mean ± SD) based on the data obtained by TF imputation were -24.5 ± 18.0, -27.4 ± 21.5 and -31.9 ± 15.4 at respectively Weeks 2, 6 and 8, indicating an increase in the degree of score change, and ranged from -19.5 ± 25.8 to -27.9 ± 19.9 during the period from Week 10 to Week 30. Both the PUCAI score and PUCAI score change based on the data obtained by TF imputation showed a tendency similar to the data before TF imputation in that the effect of the study drug in improving symptoms was observed from Week 2 and the effect lasted up to Week 30.

Table 11.4–13 Summary Statistics of PUCAI Score and PUCAI Score Change (FAS)

	PUCAI score						PUCAI score change								
	n	Mean	SD	Median	Q1	Q3	Mean	SD	Median	Q1	Q3	Min	Max	Lower limit of 95% CI	Upper limit of 95% CI
At registration	21	47.1	15.2	45.0	40.0	60.0	-	-	-	-	-	-	-	-	-
Week 2	20	20.3	16.3	22.5	2.5	35.0	-25.8	17.5	-25.0	-40.0	-7.5	-55	0	-33.9	-17.6
Week 6	20	17.3	17.5	15.0	2.5	32.5	-28.8	21.1	-35.0	-45.0	-10.0	-70	10	-38.6	-18.9
Week 8	20	12.5	13.5	10.0	0.0	22.5	-33.5	14.0	-40.0	-42.5	-22.5	-55	0	-40.0	-27.0
Week 10	18	14.7	16.9	10.0	0.0	20.0	-30.6	19.1	-37.5	-45.0	-20.0	-55	10	-40.0	-21.1
Week 14	16	12.2	12.0	10.0	2.5	15.0	-34.1	17.7	-40.0	-42.5	-27.5	-60	5	-43.5	-24.6
Week 18	16	14.4	16.8	10.0	2.5	20.0	-31.9	21.0	-40.0	-42.5	-22.5	-60	25	-43.0	-20.7
Week 22	14	18.2	15.6	12.5	5.0	35.0	-27.9	24.2	-40.0	-45.0	-10.0	-60	20	-41.8	-13.9
Week 26	14	13.2	14.0	10.0	0.0	20.0	-32.9	20.5	-37.5	-45.0	-25.0	-60	25	-44.7	-21.0
Week 30	14	19.3	18.8	15.0	5.0	30.0	-26.8	27.8	-32.5	-40.0	-15.0	-65	50	-42.8	-10.7
Last	21	28.8	22.5	30.0	10.0	40.0	-18.3	28.4	-20.0	-35.0	0.0	-65	50	-31.3	-5.4

Table 11.4–14 Summary Statistics of PUCAI Score and PUCAI Score Change (TF) (FAS)

	PUCAI score						PUCAI score change (TF)								
	n	Mean	SD	Median	Q1	Q3	Mean	SD	Median	Q1	Q3	Min	Max	Lower limit of 95% CI	Upper limit of 95% CI
At registration	21	47.1	15.2	45.0	40.0	60.0	-	-	-	-	-	-	-	-	-
Week 2	21	22.6	19.3	25.0	5.0	35.0	-24.5	18.0	-25.0	-40.0	-5.0	-55	0	-32.7	-16.4
Week 6	21	19.8	20.6	15.0	5.0	35.0	-27.4	21.5	-35.0	-45.0	-10.0	-70	10	-37.2	-17.6
Week 8	21	15.2	18.2	10.0	0.0	25.0	-31.9	15.4	-40.0	-40.0	-20.0	-55	0	-38.9	-24.9
Week 10	21	19.3	20.5	15.0	5.0	25.0	-27.9	19.9	-35.0	-45.0	-20.0	-55	10	-36.9	-18.8
Week 14	21	19.5	19.7	15.0	5.0	25.0	-27.6	20.6	-35.0	-40.0	-5.0	-60	5	-37.0	-18.2
Week 18	21	20.0	19.7	15.0	5.0	30.0	-27.1	20.0	-30.0	-40.0	-5.0	-60	0	-36.2	-18.1
Week 22	21	26.9	20.6	25.0	10.0	40.0	-20.2	23.5	-15.0	-40.0	0.0	-60	20	-30.9	-9.5
Week 26	21	23.6	21.4	20.0	5.0	40.0	-23.6	22.5	-30.0	-40.0	0.0	-60	25	-33.8	-13.3
Week 30	21	27.6	21.9	20.0	10.0	40.0	-19.5	25.8	-20.0	-35.0	0.0	-65	50	-31.3	-7.8

The PUCAI score-based remission was defined as the case where the PUCAI score on each evaluation day excluding the evaluation at the time of registration was less than 10. The PUCAI score-based remission rates are shown in Table 11.4–15, and those rates based on the data obtained by TF imputation are shown in Table 11.4–16.

The PUCAI score-based remission rate was 35.0% (7/20) at Week 2 and increased to 40.0% (8/20) at both Weeks 6 and 8. Subsequently, the PUCAI score-based remission rates ranged from 28.6% to 42.9% during the period from Week 10 to Week 30, and were 19.0% (4/21) at the last time point. The study drug was confirmed to render the effect of leading patients to remission from Week 2 and the effect lasted.

When using the data obtained by TF imputation, the PUCAI score-based remission rates were 33.3% (7/21), 38.1% (8/21) and 38.1% (8/21) respectively at Weeks 2, 6 and 8, and ranged from 19.0% to 33.3% during the period from Week 10 to Week 30. The results of the evaluation using the data obtained by TF imputation showed a similar tendency to the results obtained before TF imputation in that the effect of the study drug was observed from Week 2 and the effect lasted.

Table 11.4–15 PUCAI Score-Based Remission Rate (%) (FAS)

	PUCAI score		
	n	Remission	%
Week 2	20	7	35.0
Week 6	20	8	40.0
Week 8	20	8	40.0
Week 10	18	7	38.9
Week 14	16	6	37.5
Week 18	16	6	37.5
Week 22	14	4	28.6
Week 26	14	6	42.9
Week 30	14	4	28.6
Last	21	4	19.0

Table 11.4–16 PUCAI Score-Based Remission Rate (%) (TF) (FAS)

	PUCAI score (TF)		
	n	Remission	%
Week 2	21	7	33.3
Week 6	21	8	38.1
Week 8	21	8	38.1
Week 10	21	7	33.3
Week 14	21	6	28.6
Week 18	21	6	28.6
Week 22	21	4	19.0
Week 26	21	6	28.6
Week 30	21	4	19.0

A decrease in PUCAI score by at least 20 points is a clinically significant change[3)], and the proportions of patients who achieved a decrease in PUCAI score by at least 20 points are shown in Table 11.4–17, and the proportions of patients who achieved a decrease in PUCAI score by at least 20 points based on the

data obtained by TF imputation are shown in Table 11.4–18. The patients having less than 20 points of PUCAI score at the time of registration were excluded from this tabulation.

The proportion of patients who achieved a PUCAI score decrease by at least 20 points was 68.4% (13/19) at Week 2, and increased to 73.7% (14/19) and 89.5% (17/19) respectively at Weeks 6 and 8. Subsequently, the proportion of patients ranged from 64.3% to 88.2% during the period from Week 10 to Week 30, and was 60.0% (12/20) at the last time point. The effect in improving the symptoms was observed from Week 2 and the effect was confirmed to last afterwards.

When using the data obtained by TF imputation, the proportions of patients who achieved a PUCAI score decrease by at least 20 points were 65.0% (13/20), 70.0% (14/20) and 85.0% (17/20) respectively at Weeks 2, 6 and 8, and ranged from 45.0% to 75.0% during the period from Week 10 to Week 30. The time course of the proportion of patients was similar to that based on the data obtained by TF imputation.

Table 11.4–17 Proportion (%) of Patients Who Achieved a Decrease in PUCAI Score by at least 20 points (FAS)

	n	At least 20	%
Week 2	19	13	68.4
Week 6	19	14	73.7
Week 8	19	17	89.5
Week 10	17	15	88.2
Week 14	16	13	81.3
Week 18	16	13	81.3
Week 22	14	9	64.3
Week 26	14	12	85.7
Week 30	14	10	71.4
Last	20	12	60.0

Table 11.4–18 Proportion (%) of Patients Who Achieved a Decrease in PUCAI Score by at least 20 points (TF) (FAS)

	n	At least 20	%
Week 2	20	13	65.0
Week 6	20	14	70.0
Week 8	20	17	85.0
Week 10	20	15	75.0
Week 14	20	13	65.0
Week 18	20	13	65.0
Week 22	20	9	45.0
Week 26	20	12	60.0
Week 30	20	10	50.0

11.4.1.2 Mucosal Healing Rate

Mucosal healing was defined as the case where the subscore for findings of endoscopy of Mayo score on each evaluation day excluding the evaluation at the time of registration was not more than 1. In addition, among the patients who underwent sigmoidoscopy at the time of registration and either Week 30 or the day of discontinuation, patients in whom the subscore for findings of endoscopy at the time of registration was not more than 1 were excluded from this tabulation.

The rates of patients who achieved mucosal healing are shown in Table 11.4–19, and those based on the data obtained by TF imputation are shown in Table 11.4–20.

The rates of patients who achieved mucosal healing at Week 30 and the last time point were respectively 33.3% (2/6) and 28.6% (2/7).

When using the data obtained by TF imputation, the rate of patients who achieved mucosal healing at Week 30 was 28.6% (2/7), indicating a similar tendency to that before TF imputation.

Table 11.4–19 Rate (%) of Mucosal Healing (FAS)

	n	Mucosal healing	%
Week 30	6	2	33.3
Last	7	2	28.6

Table 11.4–20 Rate (%) of Mucosal Healing (TF) (FAS)

	n	Mucosal healing	%
Week 30	7	2	28.6

11.4.1.3 Steroid Dose

The steroid dose (oral preparation, injection) in 12 patients who had been receiving steroids (oral preparations) at the time of registration was investigated. The steroid dose should be stable for at least 2 weeks before the starting day of the screening period, and initiation of steroid treatment or an increase in steroid dose were prohibited. After the starting day of study drug administration, when a clinical improvement in the symptoms of ulcerative colitis was confirmed by the investigator (subinvestigator), a decrease in steroid dose was allowed and performed in accordance with the rules specified in the protocol. A steroid dose at each evaluation week was calculated by dividing the mean value of steroid doses, calculated on prednisolone basis, administered during the period from 6 days before the CAI score evaluation day for the relevant week to the previous day of the evaluation day by body weight measured in the relevant week.

The steroid doses (oral preparations, injection) at each evaluation time point are shown in Table 11.4–21, and those based on the data obtained by TF imputation are shown in Table 11.4–22.

The median steroid dose was 0.20 mg/kg/day at the time of registration, and was decreased to 0.19 mg/kg/day, 0.17 mg/kg/day and 0.16 mg/kg/day respectively at Weeks 2, 6 and 8. The median steroid dose ranged from 0.04 mg/kg/day to 0.15 mg/kg/day during the period from Week 10 to Week 30, and was 0.05 mg/kg/day at the last time point.

When using the data obtained by TF imputation, the median steroid doses were 0.19 mg/kg/day, 0.17 mg/kg/day and 0.16 mg/kg/day respectively at Weeks 2, 6 and 8, and ranged from 0.05 mg/kg/day to 0.15 mg/kg/day during the period from Week 10 to Week 30, indicating the similar time course of steroid dose to that obtained before TF imputation.

Table 11.4–21 Summary Statistics of Steroid Dose (mg/kg/day) (Patients Who Had Been Receiving Steroids (Oral) at the Time of Registration among FAS)

	Steroid dose (mg/kg/day)									
	n	Mean	SD	Median	Q1	Q3	Min	Max	Lower limit of 95% CI	Upper limit of 95% CI
At registration	12	0.25	0.23	0.20	0.10	0.32	0.0	0.9	0.11	0.40
Week 2	12	0.24	0.21	0.19	0.10	0.32	0.0	0.8	0.10	0.37
Week 6	12	0.17	0.14	0.17	0.04	0.24	0.0	0.5	0.08	0.26
Week 8	12	0.15	0.12	0.16	0.04	0.20	0.0	0.4	0.07	0.23
Week 10	10	0.14	0.12	0.15	0.00	0.19	0.0	0.4	0.05	0.22
Week 14	9	0.11	0.12	0.09	0.00	0.17	0.0	0.4	0.02	0.20
Week 18	9	0.09	0.10	0.05	0.00	0.16	0.0	0.3	0.02	0.16
Week 22	8	0.08	0.09	0.04	0.00	0.17	0.0	0.2	0.01	0.15
Week 26	8	0.09	0.09	0.07	0.00	0.18	0.0	0.2	0.01	0.16
Week 30	8	0.18	0.29	0.10	0.02	0.16	0.0	0.9	-0.06	0.42
Last	12	0.13	0.24	0.05	0.00	0.16	0.0	0.9	-0.02	0.29

Table 11.4–22 Summary Statistics of Steroid Dose (mg/kg/day) (TF) (Patients who had been receiving steroid (oral) at the time of registration among FAS)

	Steroid dose (mg/kg/day) (TF)									
	n	Mean	SD	Median	Q1	Q3	Min	Max	Lower limit of 95% CI	Upper limit of 95% CI
At registration	12	0.25	0.23	0.20	0.10	0.32	0.0	0.9	0.11	0.40
Week 2	12	0.24	0.21	0.19	0.10	0.32	0.0	0.8	0.10	0.37
Week 6	12	0.17	0.14	0.17	0.04	0.24	0.0	0.5	0.08	0.26
Week 8	12	0.15	0.12	0.16	0.04	0.20	0.0	0.4	0.07	0.23
Week 10	12	0.14	0.11	0.15	0.03	0.19	0.0	0.4	0.06	0.21
Week 14	12	0.10	0.11	0.07	0.00	0.18	0.0	0.4	0.03	0.17
Week 18	12	0.09	0.09	0.05	0.00	0.17	0.0	0.3	0.03	0.15
Week 22	12	0.08	0.08	0.05	0.00	0.17	0.0	0.2	0.03	0.13
Week 26	12	0.08	0.08	0.05	0.00	0.17	0.0	0.2	0.03	0.14
Week 30	12	0.14	0.24	0.05	0.02	0.16	0.0	0.9	-0.01	0.29

The rate of steroid dose change was defined as the percentage of steroid dose change calculated by subtracting a steroid dose at each evaluation day from the steroid dose at the time of registration. Summary statistics of the rate of steroid dose change are shown in Table 11.4–23, and summary statistics of the rate of steroid dose change calculated using the data obtained by TF imputation are shown in Table 11.4–24.

The median rates of steroid dose change at Weeks 2, 6 and 8 were respectively -1.63, -25.05 and -43.91%, indicating an increase in degree of change with time. The median rate of steroid dose change ranged from -61.72% to -86.93%, and was -85.44% at the last time point.

When using the data obtained by TF imputation, the rates of steroid dose change were -1.63%, -25.05% and -43.91% respectively at Weeks 2, 6 and 8, and ranged from -45.01% to -79.31% during the period from Week 10 to Week 30, indicating that the time course of the rate of steroid dose change based on the data obtained by TF imputation was similar to that obtained before TF imputation. The results suggest that administration of TA-650 might enable steroid dose reduction.

Table 11.4–23 Summary Statistics of Rate (%) of Steroid Dose Change (Patients who had been receiving steroids (oral) at the time of registration among FAS)

	Rate of steroid dose change (%)							
	n	Mean	SD	Median	Q1	Q3	Min	Max
Week 2	12	-10.76	28.22	-1.63	-5.51	-1.17	-100.0	0.7
Week 6	12	-39.00	40.11	-25.05	-73.31	-4.36	-100.0	-0.6
Week 8	12	-44.96	39.72	-43.91	-78.94	-4.28	-100.0	1.1
Week 10	10	-55.39	38.56	-61.72	-100.00	-28.64	-100.0	0.6
Week 14	9	-64.39	40.15	-77.97	-100.00	-47.89	-100.0	0.0
Week 18	9	-71.25	34.18	-77.00	-100.00	-64.79	-100.0	-5.4
Week 22	8	-75.26	34.12	-86.93	-100.00	-63.22	-100.0	-1.8
Week 26	8	-68.15	39.87	-79.23	-100.00	-41.29	-100.0	-4.2
Week 30	8	-58.85	41.36	-73.26	-94.07	-18.79	-100.0	1.4
Last	12	-64.14	42.98	-85.44	-100.00	-18.79	-100.0	1.4

Table 11.4–24 Summary Statistics of Rate (%) of Steroid Dose Change (TF) (Patients who had been receiving steroids (oral) at the time of registration among FAS)

	Rate of steroid dose change (%) (TF)							
	n	Mean	SD	Median	Q1	Q3	Min	Max
Week 2	12	-10.76	28.22	-1.63	-5.51	-1.17	-100.0	0.7
Week 6	12	-39.00	40.11	-25.05	-73.31	-4.36	-100.0	-0.6
Week 8	12	-44.96	39.72	-43.91	-78.94	-4.28	-100.0	1.1
Week 10	12	-46.07	41.12	-45.01	-83.86	-1.50	-100.0	1.1
Week 14	12	-56.53	44.58	-75.37	-100.00	0.00	-100.0	1.1
Week 18	12	-61.68	41.98	-76.73	-100.00	-15.83	-100.0	1.1
Week 22	12	-58.42	45.24	-79.31	-100.00	-0.90	-100.0	1.1
Week 26	12	-53.68	46.43	-75.38	-100.00	-2.10	-100.0	1.1
Week 30	12	-47.48	45.37	-47.38	-94.07	0.00	-100.0	1.4

Among 12 patients who had been receiving steroid (oral preparations) at the time of registration, the case where the steroid dose on each evaluation day became 0 was defined as steroid withdrawal. The rates of steroid withdrawal at each evaluation time point are shown in Table 11.4–25, and those calculated based on the data obtained by TF imputation are shown in Table 11.4–26.

The rate of steroid withdrawal was 8.3% at Week 2, and increased to 25.0% at both Weeks 6 and 8. The rate of steroid withdrawal ranged from 25.0% to 37.5% during the period from Week 10 to Week 30, and was 41.7% at the last time point.

When using the data obtained by TF imputation, the rate of steroid withdrawal was 8.3% at Week 2, and increased to 25.0% at both Weeks 6 and 8, and ranged from 16.7% to 25.0% during the period from Week 10 to Week 30, indicating that the time course of the rate of steroid withdrawal based on the data obtained by TF imputation was similar to that obtained before TF imputation.

At week 30, 2 patients achieved steroid withdrawal, and one of these 2 patients also achieved CAI score-based remission. In addition, another patient who did not achieve CAI score-based remission at Week 30 achieved CAI score-based remission at many time points other than Week 30.

Table 11.4–25 Rate of Steroid Withdrawal (%)
(Patients Who Had Used Steroids [Oral Preparation]
at Registration among FAS)

	Rate of steroid withdrawal		
	n	Withdrawal	%
Week 2	12	1	8.3
Week 6	12	3	25.0
Week 8	12	3	25.0
Week 10	10	3	30.0
Week 14	9	3	33.3
Week 18	9	3	33.3
Week 22	8	3	37.5
Week 26	8	3	37.5
Week 30	8	2	25.0
Last	12	5	41.7

Table 11.4–26 Rate of Steroid Withdrawal (%) (TF)
(Patients Who Had Used Steroids [Oral Preparation]
at Registration among FAS)

	Rate of steroid withdrawal (TF)		
	n	Withdrawal	%
Week 2	12	1	8.3
Week 6	12	3	25.0
Week 8	12	3	25.0
Week 10	12	2	16.7
Week 14	12	3	25.0
Week 18	12	3	25.0
Week 22	12	3	25.0
Week 26	12	3	25.0
Week 30	12	2	16.7

11.4.1.4 Other Laboratory Test Parameters

Laboratory values for CRP (mg/dL) are shown in Table 11.4–27.

The median CRP was 0.2 mg/dL at the time of registration, and was maintained to be 0.00 mg/dL at all time points after Week 2.

Table 11.4–27 CRP (FAS)

	CRP (mg/dL)							
	n	Mean	SD	Median	Q1	Q3	Min	Max
Week 0	21	1.75	3.68	0.20	0.00	0.60	0.0	15.1
Week 2	20	0.22	0.53	0.00	0.00	0.10	0.0	2.1
Week 6	20	0.28	0.98	0.00	0.00	0.10	0.0	4.4
Week 8	19	0.29	0.65	0.00	0.00	0.40	0.0	2.7
Week 10	18	0.36	0.62	0.00	0.00	0.30	0.0	1.8
Week 14	16	0.08	0.12	0.00	0.00	0.10	0.0	0.4
Week 18	16	0.26	0.53	0.00	0.00	0.15	0.0	1.7
Week 22	14	0.13	0.29	0.00	0.00	0.10	0.0	1.1
Week 26	14	0.06	0.12	0.00	0.00	0.10	0.0	0.4
Week 30	14	0.09	0.14	0.00	0.00	0.10	0.0	0.5

11.4.2 Statistical/Analytical Issues

11.4.2.1 Adjustments for Covariates

Not applicable because this study was an uncontrolled study.

11.4.2.2 Handling of Dropouts, or Missing Data

Handling of dropouts or missing data was performed in accordance with the predefined “9.7.1.2 Data handling,”

11.4.2.3 Interim Analyses and Data Monitoring

No interim analysis was planned in this study.

11.4.2.4 Multicenter Studies

This study was conducted utilizing a multicenter design, and all of 17 institutions had patients included in the FAS. The number of patients by participating medical institutions is shown in Table 11.4–28. Since the number of patients per institution was small, ranging from 1 to 2, in this study, the treatment-by-center interaction was not investigated.

Table 11.4–28 Number of Patients by Participating Medical Institution

Medical institution	Number of registered patients
Hokkaido P.W.F.A.C.Sapporo-Kosei General Hospital	1
Sapporo Higashi Tokushukai Hospital	1
Gunma University Hospital	1
Saitama Children's Medical Center	2
Toho University Sakura Medical Center	1
Juntendo University Hospital	1
Japan Community Health Care Organization, TokyoYamate Medical Center	1
National Center for Child Health and Development	2
Yokohama City University Medical Center	2
Osaka City University Hospital	1
Osaka General Medical Center	1
Osaka Medical College Hospital	1
Hyogo College of Medicine Hospital	2
Kyushu University Hospital	1
Fukuoka University Chikushi Hospital	1
Kurume University Hospital	1
Oita Red Cross Hospital	1
Total	21

11.4.2.5 Multiple Comparison/Multiplicity

Since no test was performed in this study, no problems related to multiple comparison/multiplicity occurred.

11.4.2.6 Use of an "Efficacy Subset" of Patients

No analysis using the efficacy subset of patients was conducted in this study.

11.4.2.7 Active-control Studies Intended to Show Equivalence

Not applicable because this study was an uncontrolled study.

11.4.2.8 Examination of Subgroups

Since the efficacy analysis set included as few as 21 patients in this study, no statistical study of subgroups was conducted. However, patients included in the efficacy analysis set were divided into 2 groups, one including patients aged from 6 years to less than 12 years, and the other including those aged from 12 years to 17 years, and the median CAI scores, median partial Mayo scores and median PUCAI scores at Weeks 0 (at the time of registration) and 8, and changes in each scores and the rates of CAI score-based remission and PUCAI score-based remission at Week 8 are shown in Table 11.4–29.

The numbers of patients in the group of those aged from 6 years to less than 12 years (hereinafter referred to as the younger group) and the group of those aged from 12 years to 17 years (hereinafter referred to as the older group) were respectively significantly different at 4 and 17 patients, but the CAI scores, partial Mayo scores and PUCAI scores observed at Week 0 in both of the groups were almost similar. Though the numbers of patients in both group were different, in terms of each of the scores and

changes in each score, and the rates of CAI score based remission and PUCAI score-based remission at Week 8, the efficacy of the drug was not considered largely different between these groups.

Table 11.4–29 Summary of Efficacy Evaluation by Age

Endpoint			≥6, <12 n	≥6, <12 Median	≥6, <12 Remission (%)	≥12, ≤17 n	≥12, ≤17 Median	≥12, ≤17 Remission (%)
CAI score	Score	Week 0	4	10.0		17	9.0	
		Week 8	3	3.0		17	3.0	
	Change	Week 8	3	-6.0		17	-6.0	
	Remission rate	Week 8	3		3 (100.0)	17		13 (76.5)
Partial Mayo score	Score	Week 0	4	6.5		17	6.0	
		Week 8	3	0.0		17	2.0	
	Change	Week 8	3	-4.0		17	-3.0	
PUCAI score	Score	Week 0	4	52.5		17	45.0	
		Week 8	3	0.0		17	10.0	
	Change	Week 8	3	-45.0		17	-40.0	
	Remission rate	Week 8	3		2 (66.7)	17		6 (35.3)

Age (year)

11.4.3 Tabulation of Individual Response Data

The efficacy evaluation data at each evaluation time point for individual patients are provided in Appendix 16.2.6.

11.4.4 Drug Dose, Drug Concentration, and Relationships to Response

11.4.4.1 Serum Infliximab Concentration

The time course of serum infliximab concentration measured at each time point is shown in Table 11.4–30. Each patient was given the study drug at a dose of 5 mg/kg at Weeks 0 (the starting day of study drug administration), 2 and 6. Subsequently, each patient was judged whether the patient was a responder or a non-responder based on the CAI score obtained at Week 8, and only CAI score-based responders were given the study drug at a dose of 5 mg/kg at Weeks 14 and 22. In addition, since CAI score-based non-responders would be allowed to use the prohibited concomitant drugs and therapies after the end of evaluation at Week 8, only the data obtained by Week 8 were included in the tabulation for all of the patients (the overall population).

The median serum infliximab concentrations 1 hour after completion of administration at Week 0 in the overall population and CAI score-based responders were respectively 97.17μg/mL and 96.35 μg/mL and those in individual patients of CAI score-based non-responders (2 patients) were respectively 106.65μg/mL and 124.05 μg/mL. The median serum infliximab concentrations at Week 8 in the overall population and the CAI score-based responders were both 25.64 μg/mL, and those in individual patients of CAI score-based non-responders (2 patients) were respectively 13.74μg/mL and 28.09 μg/mL. The median trough serum infliximab concentration in CAI score-based responders at Weeks 14, 22 and 30 was respectively 2.58 μg/mL, 1.54 μg/mL, and 1.34 μg/mL, indicating that the serum infliximab concentration was maintained. Furthermore, the serum infliximab concentrations in CAI score-based responders obtained 1 hour after completion of administration at Weeks 0, 14 and 22 were respectively

96.35 µg/mL, 95.91 µg/mL, and 102.78 µg/mL, indicating that there was not a large difference in these concentrations. Taking into consideration these results, the study drug was not considered likely to accumulate in pediatric patients by multiple administrations of the study drug. In contrast, the serum infliximab concentrations at Week 14 in individual CAI score-based non-responders (2 patients) were respectively 0.26 µg/mL and 1.74 µg/mL which were lower than the median serum infliximab concentration at Week 14 in CAI-score-based responders.

Table 11.4–30 Serum Infliximab Concentration (µg/mL)

		n	Mean	SD	Median	Q1	Q3	Min	Max
Overall	Week 0, before administration	21	0.00	0.00	0.00	0.00	0.00	0.00	0.00
	Week 0, 1 hr after completion of administration	21	96.18	15.65	97.17	80.06	107.43	65.81	124.05
	Week 2, before administration	20	21.75	5.46	21.14	18.91	24.30	9.94	36.10
	Week 6, before administration	20	12.34	7.51	10.35	6.64	16.55	1.70	30.17
	Week 8	19	26.36	9.70	25.64	18.13	34.72	11.66	43.15
CAI score-based responder	Week 0, before administration	18	0.00	0.00	0.00	0.00	0.00	0.00	0.00
	Week 0, 1 hr after completion of administration	18	95.04	14.70	96.35	80.06	107.43	65.81	114.71
	Week 2, before administration	18	21.46	5.36	21.14	18.97	24.12	9.94	36.10
	Week 6, before administration	18	12.27	7.42	10.35	6.79	15.08	1.70	30.17
	Week 8	17	27.00	9.77	25.64	20.65	34.72	11.66	43.15
	Week 10	18	12.78	7.03	13.81	6.85	17.31	1.98	28.80
	Week 14, before administration	18	3.30	3.03	2.58	0.80	4.09	0.00	10.92
	Week 14, 1 hr after completion of administration	16	100.32	19.25	95.91	82.63	118.82	74.03	130.68
	Week 18	16	11.49	7.00	10.32	5.52	16.63	2.79	23.33
	Week 22, before administration	15	2.68	3.02	1.54	0.33	4.74	0.00	10.02
	Week 22, 1 hr after completion of administration	14	103.29	18.61	102.78	83.78	116.62	72.85	137.61
	Week 26	14	10.50	8.45	8.13	5.05	17.32	0.00	27.17
	Week 30	14	2.74	3.52	1.34	0.00	4.80	0.00	12.27
CAI score-based non-responder	Week 0, before administration	2	NC	NC	NC	NC	NC	0.00	0.00
	Week 0, 1 hr after completion of administration	2	NC	NC	NC	NC	NC	106.65	124.05
	Week 2, before administration	2	NC	NC	NC	NC	NC	18.84	30.04
	Week 6, before administration	2	NC	NC	NC	NC	NC	4.87	21.19
	Week 8	2	NC	NC	NC	NC	NC	13.74	28.09
	Week 10	2	NC	NC	NC	NC	NC	5.08	6.32
	Week 14	2	NC	NC	NC	NC	NC	0.26	1.74

NC: Unable to be calculated.

The proportions of patients having a serum infliximab concentration below the limit of quantitation (0.1µg/mL) at each time point are shown in Table 11.4–31. After study drug administration, only 5.6% (1/18), 6.7% (1/15), 7.1% (1/14) and 28.6% (4/14) of the CAI score-based responders had a serum infliximab concentration respectively below the limit of quantitation before administration at Week 14, before administration at Week 22, at Week 26 and Week 30, and a serum infliximab concentration not less than the limit of quantitation was maintained in most of the patients. In addition, no CAI score-based

non-responders had a serum infliximab concentration below the limit of quantitation after the study drug administration.

Table 11.4–31 Proportion of Patients with Serum Infliximab Concentration Below the Limit of Quantitation (0.1 μg/mL)

		n	BLQ	%
Overall	Week 0, before administration	21	21	100.0
	Week 0, 1 hr after end of administration	21	0	0.0
	Week 2, before administration	20	0	0.0
	Week 6, before administration	20	0	0.0
	Week 8	19	0	0.0
CAI score-based responder	Week 0, before administration	18	18	100.0
	Week 0, 1 hr after end of administration	18	0	0.0
	Week 2, before administration	18	0	0.0
	Week 6, before administration	18	0	0.0
	Week 8	17	0	0.0
	Week 10	18	0	0.0
	Week 14, before administration	18	1	5.6
	Week 14, 1 hr after end of administration	16	0	0.0
	Week 18	16	0	0.0
	Week 22, before administration	15	1	6.7
	Week 22, 1 hr after end of administration	14	0	0.0
	Week 26	14	1	7.1
	Week 30	14	4	28.6
CAI score-based non-responder	Week 0, before administration	2	2	100.0
	Week 0, 1 hr after end of administration	2	0	0.0
	Week 2, before administration	2	0	0.0
	Week 6, before administration	2	0	0.0
	Week 8	2	0	0.0
	Week 10	2	0	0.0
	Week 14	2	0	0.0

BLQ: Below the limit of quantitation.

11.4.4.2 Relationship between Serum Infliximab Concentration and Efficacy Endpoints

The serum infliximab concentrations at Week 8 in patients who achieved/failed to achieve CAI score-based remission are shown in Table 11.4–32. The median serum infliximab concentrations in patients who achieved CAI score-based remission and patients who failed to achieve CAI score-based remission in the overall population were respectively 24.42 μg/mL and 26.87 μg/mL. In addition, the serum infliximab concentrations at Week 30 in patients who achieved/failed to achieve CAI score-based remission are shown in Table 11.4–33. The median serum infliximab concentrations in patients who achieved CAI score-based remission and patients who failed to achieve CAI score-based remission were respectively 1.20 μg/mL and 1.48 μg/mL. These results showed that there were no significant differences in the median serum infliximab concentrations between patients who achieved CAI score-based remission and those who failed to achieve CAI score-based remission, and since only a small number of patients were included in this study, a clear relationship could not be obtained between the efficacy of the study drug and a serum infliximab concentration.

Table 11.4–32 Serum Infliximab Concentration at Weeks 8 in Patients Who Achieved/Failed to Achieve CAI Score-based Remission (μg/mL)

		Serum infliximab concentration (μg/mL)							
		n	Mean	SD	Median	Q1	Q3	Min	Max
Overall	Remission	15	26.30	9.91	24.42	18.13	34.72	11.66	43.15
	Failed to achieve remission	4	26.59	10.31	26.87	19.69	33.48	13.74	38.87
CAI score-based responder	Remission	15	26.30	9.91	24.42	18.13	34.72	11.66	43.15
	Failed to achieve remission	2	NC	NC	NC	NC	NC	25.64	38.87
CAI score-based non-responder	Remission	-	-	-	-	-	-	-	-
	Failed to achieve remission	2	NC	NC	NC	NC	NC	13.74	28.09

NC: Unable to be calculated.

Table 11.4–33 Serum Infliximab Concentration at Weeks 30 in Patients Who Achieved/Failed to Achieve CAI Score-based Remission (μg/mL)

	Serum infliximab concentration (μg/mL)							
	n	Mean	SD	Median	Q1	Q3	Min	Max
Remission	9	3.10	4.12	1.20	0.00	4.80	0.00	12.27
Failed to achieve remission	5	2.10	2.37	1.48	0.25	2.98	0.00	5.78

The CAI score changes by the serum infliximab concentration (μg/mL) at Weeks 8 and 30 are shown respectively in Table 11.4–34 and Table 11.4–35. In the evaluation of the median CAI score change at Week 8, the relationship between a serum infliximab concentration and CAI score change was not clear since all patients had a serum infliximab concentration of not less than 10 μg/mL. The median CAI score changes at Week 30 in patients having a serum infliximab concentration of less than 0.1 μg/mL, not less than 0.1 μg/mL and less than 1 μg/mL, not less than 1 μg/mL and less than 10 μg/mL, and not less than 10 μg/mL were respectively -4.5, NC (unable to be calculated), 6.0 and NC. The CAI score changes in individual patients included in the groups exhibiting NC were -4 and -3 for patients with a serum infliximab concentration of not less than 0.1 μg/mL and less than 1 μg/mL, and -10 for those with a serum concentration of not less than 10 μg/mL. Based on these results, though some subgroups by serum infliximab concentration included few patients, the tendency was observed for patients having a higher serum infliximab concentration to show a larger CAI score change.

Table 11.4–34 CAI Score Change by Serum Infliximab Concentration (μg/mL) at Weeks 8

		CAI score change							
	Serum infliximab concentration (μg/mL)	n	Mean	SD	Median	Q1	Q3	Min	Max
Overall	<0.1	-	-	-	-	-	-	-	-
	≥0.1, <1	-	-	-	-	-	-	-	-
	≥1, <10	-	-	-	-	-	-	-	-
	≥10	19	-6.2	3.8	-6.0	-8.0	-5.0	-13	2
CAI score-based responder	<0.1	-	-	-	-	-	-	-	-
	≥0.1, <1	-	-	-	-	-	-	-	-
	≥1, <10	-	-	-	-	-	-	-	-
	≥10	17	-7.0	2.9	-6.0	-8.0	-5.0	-13	-2
CAI score-based non-responder	<0.1	-	-	-	-	-	-	-	-
	≥0.1, <1	-	-	-	-	-	-	-	-
	≥1, <10	-	-	-	-	-	-	-	-
	≥10	2	NC	NC	NC	NC	NC	0	2

NC: Unable to be calculated

Table 11.4–35 CAI Score Change by Serum Infliximab Concentration (μg/mL) at Weeks 30

	Serum infliximab concentration (μg/mL)	CAI score change n	Mean	SD	Median	Q1	Q3	Min	Max
CAI score-based responder	<0.1	4	-4.3	3.5	-4.5	-7.0	-1.5	-8	0
	≥0.1, <1	2	NC	NC	NC	NC	NC	-4	-3
	≥1, <10	7	-7.0	4.0	-6.0	-10.0	-4.0	-14	-2
	≥10	1	NC	NC	NC	NC	NC	-10	-10

NC: Unable to be calculated

11.4.4.3 ATI

The determination results for ATI are shown in Table 11.4–36. In addition, the measurement results for ATI determination by patient are shown in Appendix 16.1.13e. In the overall population, the measurement results were inconclusive in 81.0% (17/21) of patients and negative in 19.0% (4/21) of patients, and no patients tested positive. In CAI score-based responders, 77.8% (14/18) of the patients were inconclusive and 22.2% (4/18) tested negative. In CAI score-based non-responders, 100.0% (2/2) were inconclusive and 0.0% (0/2) tested negative.

Table 11.4–36 ATI Determination

	Negative	Positive	Inconclusive
Overall*1	19.0% (4/21)	0.0% (0/21)	81.0% (17/21)
CAI score-based responder*2	22.2% (4/18)	0.0% (0/18)	77.8% (14/18)
CAI score-based non-responder*3	0.0% (0/2)	0.0% (0/2)	100.0% (2/2)

*1 Overall period among overall patients in whom ATI was determined.
*2 Until Week 30
*3 Until Week 14

11.4.5 Drug-Drug and Drug-Disease Interactions

Drug-drug and drug-disease interactions were not investigated in this study.

11.4.6 By-Patient Display

Data for individual patients in this study are provided in Appendix 16.2.4

11.4.7 Efficacy Conclusions

(1) The CAI score (Mean ± SD) was 9.7 ± 2.7 at the time of registration, but decreased over time to 4.0 ± 3.2, 3.5 ± 3.5 and 3.2 ± 3.5 respectively at Weeks 2, 6 and 8. The CAI score ranged from 2.5 ± 2.3 to 3.5 ± 2.2 during the period from Week 10 to Week 30, and that at the last time point was 5.6 ± 3.8. The CAI score changes (Mean ± SD) were -5.5 ± 2.7, -6.0 ± 4.0 and -6.2 ± 3.7 respectively at Weeks 2, 6 and 8, indicating an increase in the degree of score change with time, and ranged from -5.9 ± 3.8 to -6.9 ± 3.8 during the period from Week 10 to Week 30, and that at the last time point was -4.1 ± 4.2. In terms of both CAI score and CAI score change, the effect of the study drug in improving symptoms was observed from Week 2, and the effect lasted up to

Week 30. Data on both CAI score and CAI score change obtained by TF imputation showed a similar tendency to data obtained before TF imputation.

(2) The CAI score-based remission rate was 60.0% (12/20) at Week 2, and increased to 80.0% (16/20) at both Weeks 6 and 8, and ranged from 64.3% to 87.5% during the period from Week 10 to Week 30. The CAI score-based remission rate at the last time point was 42.9% (9/21). In terms of CAI score-based remission rate, the study drug was observed to have the effect of bringing the clinical condition of patients to remission from Week 2, and this effect was observed up to Week 30. Data obtained by TF imputation showed a similar tendency to data obtained before TF imputation.

(3) The partial Mayo score (Mean ± SD) was 5.6 ± 1.6 at the time of registration and decreased over time to 2.6 ± 2.3, 2.2 ± 2.0 and 1.7 ± 1.7 respectively at Weeks 2, 6 and 8, and ranged from 1.8 ± 1.7 to 2.8 ± 1.9 during the period from Week 10 to Week 30. The partial Mayo score at the last time point was 3.7 ± 2.2. The partial Mayo changes (Mean ± SD) at Weeks 2, 6 and 8 were respectively -2.9 ± 2.2, -3.3 ± 2.2 and -3.8 ± 1.7, indicating an increase in the degree of score change with time. The partial Mayo changes ranged from -2.6 ± 2.8 to -3.6 ± 2.5 during the period from Week 10 to Week 30, and the change at the last time point was -1.9 ± 2.7. In terms of partial Mayo score and partial Mayo score change, the effect of the study drug in improving symptoms was observed from Week 2, and the effect lasted up to Week 30. Data on partial Mayo score and partial Mayo score change obtained by TF imputation showed a similar tendency to data obtained before TF imputation.

(4) The Mayo score (Mean ± SD) was 7.0 ± 2.4 at the time of registration, but decreased to 4.9 ± 2.4 at Week 30. The Mayo score at the last time point was 5.0 ± 2.3.The Mayo score changes (Mean ± SD) at Week 30 and the last time point were respectively -2.6 ± 3.9 and -2.0 ± 3.9. Data on both the Mayo score and Mayo score change obtained by TF imputation showed a similar tendency to data obtained before TF imputation.

(5) The Mayo score-based response rate and Mayo score-based remission rate at Week 30 were respectively 42.9% (3/7) and 14.3% (1/7), and those at the last point were respectively 37.5% (3/8) and 12.5% (1/8). Both the Mayo score-based response rate and Mayo score-based remission rate calculated based on the data obtained by TF imputation were similar to those obtained before imputation.

(6) The PUCAI score (Mean ± SD) was 47.1 ± 15.2 at the time of registration, and decreased over time to 20.3 ± 16.3, 17.3 ± 17.5 and 12.5 ± 13.5 respectively at Weeks 2, 6 and 8. The score ranged from 12.2 ± 12.0 to 19.3 ± 18.8 during the period from Week 10 to Week 30, and was 28.8 ± 22.5 at the last time point. The PUCAI score changes (Mean ± SD) were -25.8 ± 17.5, -28.8 ± 21.1 and -33.5 ± 14.0 respectively at Weeks 2, 6 and 8, and ranged from -26.8 ± 27.8 to -34.1 ± 17.7 during the period from Week 10 to Week 30. The score at the last time point was -18.3 ± 28.4. In terms of both the PUCAI score and PUCAI score change, the effect of the study drug in improving symptoms was observed from Week 2, and lasted up to Week 30. Data on PUCAI score and PUCAI score change obtained by TF imputation showed a similar tendency to data obtained before TF imputation.

(7) The PUCAI score-based remission rate was 35.0% (7/20) at Week 2, and increased to 40.0% (8/20) at both Weeks 6 and 8. Afterward, the rate ranged from 28.6% to 42.9% during the period from Week 10 to Week 30, and was 19.0% (4/21) at the last time point. The drug was observed

to have the effect of leading the clinical condition of patients to remission from Week 2, and it was confirmed that the effect lasted. Data obtained by TF imputation showed a similar tendency to data obtained before TF imputation

(8) The proportion of patients who achieved a PUCAI score decrease by at least 20 points was 68.4% (13/19) at Week 2, and increased to 73.7% (14/19) and 89.5% (17/19) respectively at Weeks 6 and 8. Afterward, the proportion ranged from 64.3% to 88.2% during the period from Week 10 to Week 30, and was 60.0% (12/20) at the last time point. The effect of the study drug in improving the score was observed from Week 2, and the effect was confirmed to last. Data obtained by TF imputation showed a similar tendency to data obtained before TF imputation.

(9) The rate of mucosal healing at Week 30 and the last time point were respectively 33.3% (2/6) and 28.6% (2/7). Data obtained by TF imputation showed a similar tendency to data obtained before TF imputation.

(10) In 12 patients who had been using steroids (oral preparations) at the time of registration, the median steroid dose was 0.20 mg/kg/day at the time of registration, but decreased respectively to 0.19, 0.17 and 0.16 mg/kg/day at Weeks 2, 6 and 8. The median steroid dose ranged from 0.04 mg/kg/day to 0.15 mg/kg/day during the period from Week 10 to Week 30, and was 0.05 mg/kg/day at the last time point. The median changes of steroid dose were respectively -1.63%, -25.05% and -43.91% at Weeks 2, 6 and 8, and the degree of change increased over time. The median change of steroid dose ranged from -61.72% to -86.93% during the period from Week 10 to Week 30, and was -85.44% at the last time point. Data on both steroid dose and rate of steroid dose change obtained by TF imputation showed a similar tendency to data obtained before TF imputation, suggesting that the study drug may reduce the steroid dose.

(11) The rate of steroid withdrawal was 8.3% at Week 2, and increased with time to 25.0% at both Weeks 6 and 8. The rate of steroid withdrawal ranged from 25.0% to 37.5% during the period from Week 10 to Week 30, and was 41.7% at the last time point. Data obtained by TF imputation showed a similar tendency to data obtained before TF imputation. Two patients achieved steroid withdrawal at Week 30, and one of these patients also achieved CAI score-based remission.

(12) The patients included in the efficacy analysis set were divided into two groups, one for those aged not less than 6 years and less than 12 years, and the other for those aged not less than 12 years and not more than 17 years, and the efficacy of the study drug was evaluated for each group. As a result, both of the two groups included a different number of patients, but the efficacy of the study drug between the two groups was not considered significantly different.

(13) The median trough serum infliximab concentrations in CAI score-based responders were respectively 2.58, 1.54 and 1.34 μg/mL at Weeks 14, 22 and 30 indicating maintenance of the serum infliximab concentration.

(14) The median CAI score changes at Week 30 in each of the 4 groups by serum infliximab concentration, i.e. less than 0.1 μg/mL, not less than 0.1 and less than 1 μg/mL, not less than 1 and less than 10 μg/mL, and not less than 10 μg/mL, were respectively -4.5, NC (impossible to calculate), -6.0, and NC. The CAI score changes in individual patients included in the group which exhibited NC were -4 and -3 in the group of not less than 0.1 and less than 1 μg/mL, and -10 in the group of not less than 10 μg/mL. Based on these results, though some of the serum infliximab concentration groups included a small number of patients, a higher serum infliximab concentration was observed to show a trend toward better efficacy.

(15) Throughout the study period, the results of ATI determination were inconclusive in 81.0% (17/21) and negative in 19.0% (4/21) in the overall population, and no patients exhibited positive results.

Based on the above results, it was shown that TA-650 given to pediatric patients with moderate to severe ulcerative colitis at a dose of 5 mg/kg at Weeks 0, 2 and 6 was able to improve clinical symptoms of ulcerative colitis and achieve mucosal healing. Continuous administration at 8-week intervals at Weeks 14 and 22 was observed to maintain the effect of the drug. Also, administration of TA-650 might allow reducing the steroid dose or allow steroid withdrawal.

12. Safety Evaluation

12.1 Extent of Exposure

The safety analysis set included 21 patients who were given the study drug at least once and for whom safety data after the start of study drug administration were obtained.

Distribution of duration of the safety evaluation period is shown in Table 12.1–1. The mean duration of the safety evaluation period was 176.7 days, with the minimum of 61 days and the maximum of 239 days.

Table 12.1–1 Distribution of Duration (Day) of Safety Evaluation Period

Frequency distribution				Summary statistics			
≤55	56- 97	98- 153	≥154	n	Mean	Min	Max
0	5	2	14	21	176.7	61	239

The frequency distribution of the total number of doses is shown in Table 12.1–2. The mean number of doses was 4.3 times, and the minimum number of dose was one time in one patient. Most of the patients were given the study drug 5 times, which is the maximum number of doses.

Table 12.1–2 Frequency Distribution of Total Number of Doses per Patient

1	2	3	4	5	Mean
1	0	4	2	14	4.3

12.2 Adverse Events (AEs)

12.2.1 Brief Summary of Adverse Events

The incidence rates of AEs in the safety analysis population is presented in Table 12.2–1.

The incidence rates of AEs and ADRs were respectively 95.2% (20/21) and 71.4% (15/21). The incidence rates of serious AEs and serious ADRs were respectively 14.3% (3/21) and 4.8% (1/21). The incidence rate of AEs leading to discontinuation of the study was 4.8% (1/21), but no ADRs leading to discontinuation were observed. The incidence rates of infections and infections (ADRs) were respectively 61.9% (13/21) and 23.8% (5/21). Among infections, the incidence rates of serious infections and serious infections (ADRs) were both 4.8% (1/21). No infections leading to discontinuation of treatment were observed. The incidence rates of infusion reactions and infusion reactions (ADRs) were both 9.5% (2/21). No serious infusion reactions and infusion reactions leading to discontinuation of treatment were observed.

Table 12.2–1 Incidence rates of AEs

MedDRA version: 17.1

	(N=21)	
	n	(%)
AEs	20	(95.2)
AEs (SOC is other than investigations)	19	(90.5)
ADRs	15	(71.4)
ADRs (SOC is other than investigations)	9	(42.9)
Serious AEs	3	(14.3)
Serious ADRs	1	(4.8)
AEs leading to discontinuation of the study	1	(4.8)
ADRs leading to discontinuation of the study	0	(0.0)
Infections	13	(61.9)
Infections (ADRs)	5	(23.8)
Serious infections	1	(4.8)
Serious infections (ADRs)	1	(4.8)
Infections leading to discontinuation of treatment	0	(0.0)
Infusion reaction	2	(9.5)
Infusion reaction (ADRs)	2	(9.5)
Serious infusion reaction	0	(0.0)
Infusion reaction leading to discontinuation of treatment	0	(0.0)
AEs (immunoserological test)	12	(57.1)

12.2.2 Display of Adverse Events

Names of AEs were presented using the lowest level terms (LLT) in accordance with the ICH Medical Dictionary for Regulatory Activities Terminology/Japanese edition (MedDRA/J ver. 17.1). AEs were tabulated using preferred terms (PT) and classified according to the system organ class (SOC).

The lists of AEs and ADRs are shown respectively in Table 12.2–2 and Table 12.2–3.

Table 12.2–2 Adverse Events

MedDRA version: 17.1 Tabulation of SOC-PT

SOC	(N=21)		
PT	n	(%)	Number of events
Total	20	(95.2)	71
Infections and infestations	12	(57.1)	23
Nasopharyngitis	7	(33.3)	13
Upper respiratory tract infection	2	(9.5)	4
Enteritis infectious	2	(9.5)	2
Bronchitis	1	(4.8)	1
Gastroenteritis	1	(4.8)	1
Sinusitis	1	(4.8)	1
Subcutaneous abscess	1	(4.8)	1

Table 12.2–2 (Continued)

MedDRA version: 17.1 Tabulation of SOC-PT

SOC<br>PT	(N=21) n	(%)	Number of events
Blood and lymphatic system disorders	3	(14.3)	3
Iron deficiency anemia	2	(9.5)	2
Anaemia	1	(4.8)	1
Nervous system disorders	1	(4.8)	1
Headache	1	(4.8)	1
Eye disorders	1	(4.8)	1
Scleritis	1	(4.8)	1
Cardiac disorders	1	(4.8)	1
Vasculitis	1	(4.8)	1
Respiratory, thoracic and mediastinal disorders	4	(19.0)	4
Asthma	1	(4.8)	1
Rhinitis allergic	1	(4.8)	1
Upper respiratory tract inflammation	1	(4.8)	1
Oropharyngeal pain	1	(4.8)	1
Gastrointestinal disorders	11	(52.4)	13
Colitis ulcerative	2	(9.5)	2
Nausea	2	(9.5)	2
Vomiting	2	(9.5)	2
Abdominal pain lower	1	(4.8)	1
Abdominal pain upper	1	(4.8)	1
Cheilitis	1	(4.8)	1
Constipation	1	(4.8)	1
Enterocolitis	1	(4.8)	1
Pancreatitis	1	(4.8)	1
Autoimmune pancreatitis	1	(4.8)	1
Skin and subcutaneous tissue disorders	2	(9.5)	3
Acne	2	(9.5)	2
Pruritus	1	(4.8)	1
Musculoskeletal and connective tissue disorders	3	(14.3)	4
Arthralgia	1	(4.8)	1
Back pain	1	(4.8)	1
Myalgia	1	(4.8)	2
General disorders and administration site conditions	2	(9.5)	2
Chest discomfort	1	(4.8)	1
Infusion site pain	1	(4.8)	1
Investigations	12	(57.1)	15
Double stranded DNA antibody positive	12	(57.1)	12
Blood pressure decreased	1	(4.8)	1
Protein urine present	1	(4.8)	1
Antinuclear antibody increased	1	(4.8)	1
Injury, poisoning and procedural complications	1	(4.8)	1
Thermal burn	1	(4.8)	1

Table 12.2–3 Adverse Drug Reactions

MedDRA version: 17.1 Tabulation of SOC-PT

SOC<br>PT	(N=21)		
	n	(%)	Number of events
Total	15	(71.4)	27
Infections and infestations	4	(19.0)	6
Nasopharyngitis	2	(9.5)	3
Bronchitis	1	(4.8)	1
Subcutaneous abscess	1	(4.8)	1
Enteritis infectious	1	(4.8)	1
Blood and lymphatic system disorders	1	(4.8)	1
Iron deficiency anaemia	1	(4.8)	1
Gastrointestinal disorders	3	(14.3)	3
Enterocolitis	1	(4.8)	1
Nausea	1	(4.8)	1
Autoimmune pancreatitis	1	(4.8)	1
Skin and subcutaneous tissue disorders	1	(4.8)	1
Acne	1	(4.8)	1
Musculoskeletal and connective tissue disorders	2	(9.5)	2
Arthralgia	1	(4.8)	1
Myalgia	1	(4.8)	1
General disorders and administration site conditions	1	(4.8)	1
Infusion site pain	1	(4.8)	1
Investigations	12	(57.1)	13
Double stranded DNA antibody positive	12	(57.1)	12
Antinuclear antibody increased	1	(4.8)	1

12.2.3 Analysis of Adverse Events

12.2.3.1 Adverse Events (AEs) and Adverse Drug Reactions (ADRs)

The AEs and ADRs observed during the evaluation period are respectively listed in Table and Table 12.2–3. The SOCs of AEs which occurred in at least 20% of the patients included “infections and infestations” and “investigations” both having an incidence rate of 57.1% (12/21), and “gastrointestinal disorders” having an incidence rate of 52.4% (11/21). The AEs which occurred in at least 5% of the patients included “double stranded DNA antibody positive” having an incidence rate of 57.1% (12/21), “nasopharyngitis” having an incidence rate of 33.3% (7/21), “upper respiratory tract infection,” “enteritis infectious,” “iron deficiency anaemia,” “colitis ulcerative” (recorded as “worsened ulcerative colitis” by the investigator), “nausea,” “vomiting” and “acne” each having an incidence rate of 9.5% (2/21).

The SOCs of ADRs which occurred in at least 20% of the patients included “investigations” having an incidence rate of 57.1% (12/21). The ADRs which occurred in at least 5% of the patients included “double stranded DNA antibody positive” having an incidence rate of 57.1% (12/21) and “nasopharyngitis” having an incidence rate of 9.5% (2/21). Among both AEs and ADRs observed, “double stranded DNA antibody positive” and “nasopharyngitis” had a high incidence rates.

12.2.3.2 AEs and ADRs by Severity (Other Than Immunoserological Tests)

The AEs and ADRs (other than immunoserological tests) are listed respectively by severity in Table 14.3–1 and Table 14.3–2.

Severity of AEs was judged by the investigator (subinvestigator). AEs were classified as mild (no interference with patient's activities of daily living), moderate (some interfere with patient's activities of daily living due to the event) and severe (the patient cannot perform daily living activities due to the event) in terms of the effect of the relevant event on daily life of patients.

The incidence rates of AEs by severity were 61.9% (13/21) for mild, 23.8% (5/21) for moderate, and 4.8% (1/21) for severe. The severe AE was "colitis ulcerative" which was judged to be a worsened primary disease, and a causal relationship of this event with the study drug was ruled out. The incidence rates of ADRs by severity was 23.8% (5/21) for mild and 19.0% (4/21) for moderate, and no severe ADRs were observed.

12.2.3.3 AEs and ADRs by Evaluation Time Point

The incidence rate of AEs (SOC is other than investigations) and ADRs (SOC is other than investigations) are shown in Table 12.2–4 by evaluation time point.

The incidence rates of AEs and ADRs which occurred during the period "from the start of study drug administration (Week 0) until before administration at Week 14" was respectively 76.2% (16/21) and 28.6% (6/21), and among these, the incidence rates of infections and infections (ADRs) was respectively 42.9% (9/21) and 9.5% (2/21). The incidence rates of AEs and ADRs during the period "from the start of study drug administration (Week 0) until before administration at Week 14" was higher compared to those observed during the periods "from administration as Week 14 until before administration at Week 22" and "from administration at Week 22 until the evaluation day at Week 30,"

In contrast, the incidence rates of AEs and ADRs which occurred during the period "from administration at Week 22 until the evaluation day at Week 30" were respectively 35.7% and 0.0%, which were equivalent to or well below the incidence rates of AEs and ADRs which occurred during the period "from administration at Week 14 until before administration respectively at Week 22," 31.3% and 18.8%, indicating no increase in the incidence rates of AEs or ADRs associated with an increase in the number of doses. The incidence rates of infections and infections (ADRs) by evaluation time point showed a similar tendency to that observed for AEs and ADRs, and no increase in an incidence rate due to repeated dose was observed.

Table 12.2–4 Incidence rates of AEs (SOC is Other Than Investigations) and ADRs (SOC is Other Than Investigations) by Evaluation Time Point

MedDRA version: 17.1 Tabulation of SOC-PT

	AE (other than investigations)						ADR (other than investigations)					
	AE			Infection			ADR			Infection (ADR)		
	n	No. of event	%	n	No. of event	%	n	No. of event	%	n	No. of event	%
Starting day of study drug administration (Week 0) to before administration at Week 14	21	16	76.2	21	9	42.9	21	6	28.6	21	2	9.5
Administration at Week 14* to before administration at Week 22	16	5	31.3	16	5	31.3	16	3	18.8	16	3	18.8
Administration at Week 22* to evaluation day at Week30	14	5	35.7	14	1	7.1	14	0	0.0	14	0	0.0

Other than investigations: AEs excluding those SOC which are investigations

*: AEs which occurred on the day of study drug administration were considered to occur before administration of the study drug, except for AEs which definitely occurred after administration such as infusion reactions.

12.2.4 Listing of Adverse Events by Patient

Lists of AEs by patient are provided in Appendix 16.2.7a.

12.3 Deaths, Other Serious Adverse Events, and Other Significant Adverse Events

12.3.1 Listing of Deaths, Other Serious Adverse Events and Other Significant Adverse Events

12.3.1.1 Deaths

No deaths occurred throughout the evaluation period.

12.3.1.2 Other Serious Adverse Events

As other serious adverse events, “colitis ulcerative,” which was a worsened primary disease, and “enterocolitis” were observed respectively in 2 patients and one patient. A list of serious AEs by patient is shown in Appendix 16.2.7b.

12.3.1.3 Other Significant Adverse Events

In this study, infections leading to discontinuation of treatment and infusion reactions leading to discontinuation of treatment were defined as significant adverse events in the protocol. Infections are defined as adverse events which were judged as infections by the investigator (subinvestigator), and infusion reactions were defined as adverse events which occurred during administration of the study drug or within 2 hours after the end of administration. No significant adverse events occurred throughout the evaluation period.

12.3.2 Narratives of Deaths, Other Serious Adverse Events and Certain Other Significant Adverse Events

No deaths occurred throughout the evaluation period. The narratives of other serious AEs are shown in Table 14.3–5.

12.3.3 Analysis and Discussion of Deaths, Other Serious Adverse Events and Other Significant Adverse Events

12.3.3.1 Deaths

No deaths occurred throughout the evaluation period.

12.3.3.2 Other Serious Adverse Events

Serious AEs and serious ADRs are listed respectively in Table 12.3–1 and Table 12.3–2.

Throughout the evaluation period, serious AEs were observed in 14.3% (3/21) of patients. Detailed information about serious AEs is described below.

One patient who experienced “colitis ulcerative” was a male aged 13 years, and at the evaluation at Week 10, after receiving the study drug 3 times, abdominal pain, increased stool frequency and blood in stool were observed. On the next day of the evaluation, the relevant patient was admitted to the hospital and underwent endoscopy. Since the endoscopic findings showed a relapse of ulcerative colitis, this patient discontinued participation in the study. This discontinuation was judged due to an occurrence of an adverse event “colitis ulcerative” (described as “worsened ulcerative colitis” by the investigator). Subsequently, the symptoms were improved by receiving prednisolone treatment and cytapheresis and the patient was discharged from the hospital. The severity of this event was moderate. This event was the worsened primary disease, and a causal relationship of this event with the study drug was ruled out.

Another patient who experienced “colitis ulcerative” was a female aged 17 years, and judged as a CAI score-based non-responder at evaluation at Week 8. At the evaluation at Week 10 after receiving the study drug 3 times, increased stool frequency, mushy stools and pyrexia (38.0°C) were observed. Though the patient received an increased dose of prednisolone and cytapheresis, the symptoms were exacerbated. The patient was therefore admitted to the hospital to receive a combination of steroid pulse therapy and antibiotics 15 days after the evaluation at Week 10. However, since the symptoms were not improved even after the relevant therapy, the patient started to receive tacrolimus orally. As a result of colonoscopy, diffuse deep ulcer and pseudopolyposis were found, and persistent blood in stool, frequent diarrhea and increased nocturnal abdominal pain were observed. The relevant patient discontinued receiving all oral preparations, and after being transferred to another hospital, underwent surgery (subtotal removal of the large intestine and ileostomy). Subsequently, since the symptoms were improved, the patient was discharged from the hospital. The severity of this event was severe. This event was the worsened primary disease, and a causal relationship of this event with the study drug was ruled out.

The patient who experienced “enterocolitis” was a male aged 14 years, and a slight increase in CRP was observed in this patient in the evaluation at Week 18, after receiving the study drug 4 times. Subsequently, the patient had pyrexia (39.0°C), vomiting and increased stool frequency which resulted in awakening for defecation during sleeping. The patient was therefore admitted to the hospital 4 days after the evaluation at Week 18. Adenoviral enteritis was most suspected but results from test did not allow diagnosing the patient as having adenoviral infection. After admission to the hospital, the patient underwent only the intake of fluid replacement and water, achieved remission during the natural course of the disease without any drug therapy, and the patient was discharged from the hospital. The severity of this event was moderate. Since the relationship between the study drug and onset of the AE cannot be ruled out, the causal relationship of this event with the study drug was judged as “possibly related.”

The outcomes of all of these events were recovery.

Table 12.3–1 Serious Adverse Events

MedDRA version: 17.1 Tabulation of SOC-PT

SOC	(N=21)		
PT	n	(%)	No. of events

Total	3	(14.3)	3
Gastrointestinal disorders	3	(14.3)	3
Colitis ulcerative	2	(9.5)	2
Enterocolitis	1	(4.8)	1

Table 12.3–2 Serious Adverse Drug Reaction

MedDRA version: 17.1 Tabulation of SOC-PT

SOC	(N=21)		
PT	n	(%)	No. of events
Total	1	(4.8)	1
Gastrointestinal disorders	1	(4.8)	1
Enterocolitis	1	(4.8)	1

12.3.3.3 Other Significant Adverse Events

In this study, infections leading to discontinuation of treatment and infusion reactions (adverse events which occurred during administration of the study drug or within 2 hours after the end of administration) leading to discontinuation of treatment were defined as significant adverse events in the protocol. In this section, in addition to these events, AEs leading to discontinuation of study, infections and infusion reactions are discussed.

12.3.3.3.1 AEs Leading to Discontinuation

A list of AEs leading to discontinuation of the study in the overall population is shown in Table 12.3–3. AEs leading to discontinuation of the study were identified based on judgment by the investigator (subinvestigator).

As an AE leading to discontinuation, “colitis ulcerative,” which was a worsened primary disease, was observed in 4.8% (1/21) of patients. The detailed information about this event is described in Section 12.3.3.2.

Throughout the evaluation period, no ADRs leading to discontinuation were observed.

Table 12.3–3 AEs Leading to Discontinuation

MedDRA version: 17.1 Tabulation of SOC-PT

SOC	(N=21)		
PT	n	(%)	No. of events
Total	1	(4.8)	1
Gastrointestinal disorders	1	(4.8)	1
Colitis ulcerative	1	(4.8)	1

12.3.3.3.2 Infections

Infections and infections (ADRs) are respectively listed in Table 12.3–4 and Table 12.3–5. Infections were identified based on judgment by the investigator (subinvestigator).

The incidence rate of infections throughout the evaluation period was 61.9% (13/21). The SOC of infections which occurred in at least 20% of the patients was “infections and infestations,” having an incidence rate of 57.1% (12/21). Infections which occurred in at least 5% of the patients included “nasopharyngitis” having an incidence rate of 33.3% (7/21), “upper respiratory tract infection” and “enteritis infectious” each having an incidence rate of 9.5% (2/21).

The SOC of infections (ADRs) which occurred most frequently was “infections and infestations” having an incidence rate of 19.0% (4/21). The infection (ADRs) which occurred in at least 5% of the patients was “nasopharyngitis” having an incidence rate of 9.5% (2/21).

The serious infections and serious infections (ADRs) are respectively listed in Table 14.3–3 and Table 14.3–4.

“Enterocolitis” was observed as both serious infections and a serious infection (ADR) in 4.8% (1/21) of patients. The detailed information about this event is described in Section 12.3.3.2.

Throughout the evaluation period, no infections leading to discontinuation of treatment were observed.

Table 12.3–4 Infections

MedDRA version: 17.1 Tabulation of SOC-PT

SOC<br>PT	(N=21) n	(%)	No. of events
Total	13	(61.9)	26
Infections and infestations	12	(57.1)	23
Nasopharyngitis	7	(33.3)	13
Upper respiratory tract infection	2	(9.5)	4
Enteritis infectious	2	(9.5)	2
Bronchitis	1	(4.8)	1
Gastroenteritis	1	(4.8)	1
Sinusitis	1	(4.8)	1
Subcutaneous abscess	1	(4.8)	1
Respiratory, thoracic and mediastinal disorders	1	(4.8)	1
Upper respiratory tract inflammation	1	(4.8)	1
Gastrointestinal disorders	1	(4.8)	1
Enterocolitis	1	(4.8)	1
Skin and subcutaneous tissue disorders	1	(4.8)	1
Acne	1	(4.8)	1

Table 12.3–5 Infections (ADRs)

MedDRA version: 17.1 Tabulation of SOC-PT

SOC<br>PT	(N=21)<br>n	 (%)	 No. of events
Total	5	(23.8)	8
Infections and infestations	4	(19.0)	6
Nasopharyngitis	2	(9.5)	3
Bronchitis	1	(4.8)	1
Subcutaneous abscess	1	(4.8)	1
Enteritis infectious	1	(4.8)	1
Gastrointestinal disorders	1	(4.8)	1
Enterocolitis	1	(4.8)	1
Skin and subcutaneous tissue disorders	1	(4.8)	1
Acne	1	(4.8)	1

12.3.3.3.3 Infusion Reaction

Infusion reactions and infusion reactions (ADRs) are respectively listed in Table 12.3–6 and Table 12.3–7. Infusion reactions were defined as adverse events which occurred during administration of the study drug or within 2 hours after completion of administration.

The incidence rates of infusion reactions and infusion reactions (ADRs) were both 9.5% (2/21), and the incidence rates of “nausea” and “injection site pain” were both 4.8% (1/21). The severity of “nausea” and “injection site pain” was respectively judged as moderate and mild. Recovery from both of these events occurred during the day of onset. In addition, no serious infusion reactions and infusion reactions leading to discontinuation of treatment were observed.

Table 12.3–6 Infusion Reaction

MedDRA version: 17.1 Tabulation of SOC-PT

SOC<br>PT	(N=21)<br>n	 (%)	 No. of events
Total	2	(9.5)	2
Gastrointestinal disorders	1	(4.8)	1
Nausea	1	(4.8)	1
General disorders and administration site conditions	1	(4.8)	1
Infusion site pain	1	(4.8)	1

Table 12.3–7 Infusion Reaction (ADRs)

MedDRA version: 17.1 Tabulation of SOC-PT

SOC<br>PT	(N=21)<br>n	 (%)	 No. of events
Total	2	(9.5)	2
Gastrointestinal disorders	1	(4.8)	1
Nausea	1	(4.8)	1
General disorders and administration site conditions	1	(4.8)	1
Infusion site pain	1	(4.8)	1

The incidence rates of infusion reactions by the number of doses is shown in Table 12.3–8.

Infusion reactions were not observed after the first and second administrations, but occurred after the third administration and onward. The incidence rates of infusion reactions and infusion reactions (ADRs) was both 5.0% (1/20) after the third administration and were both 6.3% (1/16) after the fourth administration, and neither of these events occurred after the fifth administration.

Based on the above results, a tendency for the incidence rate of infusion reactions to increase along with repeated administration of the study drug was not observed.

Table 12.3–8 Incidence rates of Infusion Reactions by the Number of Doses

		n	No. of event	%
Infusion reaction	1	21	0	0.0
	2	20	0	0.0
	3	20	1	5.0
	4	16	1	6.3
	5	14	0	0.0
Infusion reaction (ADRs)	1	21	0	0.0
	2	20	0	0.0
	3	20	1	5.0
	4	16	1	6.3
	5	14	0	0.0

The incidence rates of infusion reactions per dose are shown in Table 12.3–9.

The incidence rates of infusion reactions per dose and infusion reactions (ADRs) per dose were both 2.2% (2/91).

Table 12.3–9 Incidence rates of Infusion Reactions per Dose

	Total No. of doses	No. of event	%
Infusion reaction	91	2	2.2
Infusion reaction (ADR)	91	2	2.2

Total No. of doses: Sum of total number of doses in all of the patients.
No. of event: Number of doses after which an infusion reaction occurred.
%: No. of event/Total No. of doses ×100

The incidence rates of infusion reactions by results from ATI determination (negative, positive, inconclusive) are shown in Table 12.3–10.

Infusion reactions and infusion reactions (ADRs) were observed only in patients who were inconclusive for ATI determination, and the incidence rates for both ware 11.8% (2/17).

Table 12.3–10 Incidence rates of Infusion Reactions by Results of ATI

		n	No. of event	%
Infusion reaction	Negative	4	0	0.0
	Positive	0	-	-
	Inconclusive	17	2	11.8
Infusion reaction (ADRs)	Negative	4	0	0.0
	Positive	0	-	-
	Inconclusive	17	2	11.8

12.3.3.3.4 Others

12.3.3.3.4.1 Immunoserological Tests

A list of AEs (immunoserological tests) in the overall population is shown in Table 12.3–11. For anti-nuclear antibody, an event where an antibody titer was worsened by 3 ranks or more compared to the previous measurement value was treated as an adverse event. For double stranded DNA IgM antibody and double stranded DNA IgG antibody, an event where the determination result changed from negative to positive or from positive to positive (worsening) was treated as an adverse event. A determination result for double stranded DNA IgG antibody titer exceeding 12 IU/mL and that for double stranded DNA IgM antibody titer of not less than 6 U/mL were judged as positive.

The incidence rate of AEs (immunoserological tests) was 57.1% (12/21), and the incidence rates of "double stranded DNA (IgM) antibody positive" and "antinuclear antibody increased" were respectively 57.1% (12/21) and 4.8% (1/21). No patients experienced a change from negative to positive to double stranded DNA IgG antibody, which is said to be correlated with lupus-like syndrome. In addition, lupus-like syndrome was not observed in any patients including those experienced these AEs (immunoserological tests).

Table 12.3–11 Adverse Events (Immunoserological Test)

	(N=21)		
	n	(%)	No. of events
Total	12	(57.1)	13
Immunoserological test	12	(57.1)	13
Antinuclear antibody↑	1	(4.8)	1
Double stranded DNA (IgM) antibody↑	12	(57.1)	12
Double stranded DNA (IgG) antibody↑	0	(0.0)	0

Other than the above-mentioned event, malignant tumor (including hepatosplenic T-cell lymphoma which has been observed in children and young adults), tuberculosis, demyelinating disease, interstitial pneumonia, delayed type hypersensitivity (including serum sickness-like reaction), congestive heart failure, serious blood disorder and phabdomyolysis, which are the events to be noted when using TA-650, were not observed. Throughout the evaluation period, pregnancies among female pediatric patients or spouses (partners) of male pediatric patients were not reported.

12.4 Clinical Laboratory Evaluation

12.4.1 List of Individual Laboratory Measurements by Patient and Each Abnormal Laboratory Value

Lists of laboratory values (general laboratory tests and immunoserological tests) by patient are provided in Appendix 16.2.8a to Appendix 16.2.8e.

12.4.2 Evaluation of Each Laboratory Parameter

12.4.2.1 Laboratory Values throughout the Study Period

The incidence rates of abnormal changes in laboratory values are shown in Table 14.3–6. In addition, summary statistics of hematology and blood biochemistry at each evaluation point are shown in Table 14.3–7, and frequency distribution of urinalysis (qualitative) results is shown in Table 14.3–8.

Since reference ranges for laboratory parameters in children vary depending on age and sex, the reference ranges for laboratory parameters were established by using reference values based on patient's sex and age (the age at informed consent obtainment is used) in reference to the "New Pocket Guide of Laboratory Test Reference Values for Children"[2]. For a differential count of WBC, for which no reference ranges are listed in this material, abnormal changes were not assessed. Since it was judged that the reference ranges for urinalysis (qualitative) in adults can also be used in children without any clinical problems, the reference values (for adults) provided from the contract laboratory were used to assess abnormal changes.

Summary statistics of each laboratory parameter at each evaluation point were not calculated by age or sex of patients, but were calculated using the measurement values from the overall population.

For general laboratory tests excluding urinalysis, abnormal changes defined below were assessed for each laboratory parameter.

– Laboratory value at Week 0 was within the reference range (including limits), and one or more values after treatment worsened beyond the reference range (not including limits) with a percent change from the laboratory value at Week 0 of at least 25%.
– Laboratory value at Week 0 deviated from the reference range, and one or more values after treatment worsened toward the same direction with a percent change from the laboratory value at Week 0 of at least 25%.
– Laboratory value at Week 0 deviated from the reference range, and one or more values after treatment worsened beyond the opposite limit of the reference range.
– Laboratory value at Week 0 was missing, and one or more values after treatment deviated from the reference range.

For urinalysis, abnormal changes defined below were assessed for each laboratory parameter.

– Laboratory value at Week 0 was within the reference range (including limits), and one or more values after treatment worsened beyond the reference range (not including limits) with a change of at least one rank.
– Laboratory value at Week 0 deviated from the reference range, and one or more values after treatment worsened toward the same direction with a change from laboratory value at Week 0 of at least one rank.
– Laboratory value at Week 0 deviated from the reference range, and one or more values after treatment worsened beyond the opposite limit of the reference range.
– Laboratory value at Week 0 was missing, and one or more values after treatment deviated from the reference range.

The laboratory parameters with an incidence rate of abnormal changes of at least 10% included urine protein (increased) and occult blood in urine (increased) each having an incidence rate of 47.6% (10/21), ALT (GPT) (increased) having an incidence rate of 33.3% (7/21), white blood cell count (decreased), ALP (decreased), γ-GTP (decreased), and BUN (decreased) each having an incidence rate of 14.3% (3/21).

Among the 7 parameters, for 5 laboratory parameters other than urinalysis (urine protein and occult blood in urine), median measurement values obtained at the starting day of study drug administration (Week 0) and at evaluation points thereafter were compared. For ALT (GPT), the median measurement value was 10.0 U/L at Week 0, and ranged between 9.0 U/L and 12.0 U/L after Week 2. For white blood cell count, the median measurement value was 8600.0/μL at Week 0, and ranged between 5800.0/μL and 7300.0/μL after Week 2. For ALP, the median measurement value was 382.0 U/L at Week 0, and ranged between 394.5 U/L and 545.0 U/L after Week 2. For γ-GTP, the median measurement value was 13.0 U/L at Week 0, and ranged between 10.5 U/L and 15.5 U/L after Week 2. For BUN, the median measurement value was 9.0 mg/dL at Week 0, and ranged between11.0 mg/dL and 13.0 mg/dL after Week 2. All of the median measurement values for these 5 test parameters fell within the ranges between the minimum and the maximum of reference values for children aged from 10 to 17 years and the range of ages in which the patients participating in this study were included. In addition, no AEs related to hematology and blood biochemistry were observed.

For urinalysis (urine protein and occult blood in urine), no significant changes in distribution were observed, but as an adverse event related to urinalysis, "Protein urine present" was observed in 4.8% (1/21) of the patients. Since the relevant patient was in menstruation at the time of the laboratory test, the investigator (subinvestigator) judged that this event was caused by menstruation, and a causal relationship of this event with the study drug was ruled out.

As described above, no changes which may cause clinical problems were observed in the above mentioned 7 laboratory parameters having an incidence rate of abnormal change of at least 10%.

12.4.2.2 Individual Patient Changes

Lists of laboratory values by patient are provided in Appendix 16.2.8a to Appendix 16.2.8e, and Appendix 16.2.8g.

12.4.2.3 Individual Clinically Significant Abnormalities

Among the abnormal changes in the laboratory values of general laboratory tests, no serious events or events leading to discontinuation of the study were observed.

12.5 Vital Signs, Physical Findings and Other Observations Related to Safety

12.5.1 Physical Examination (Systolic and Diastolic Blood Pressure, Pulse Rate, and Body Temperature)

Determination of physical examination (systolic and diastolic blood pressure, pulse rate, and body temperature) was performed immediately before the start of administration, every 30 minutes during administration, and every 30 minutes for 2 hours after the end of administration on each administration day. When there was at least a 15-minute interval between the end of administration and the latest measurement before the end of administration, measurement was also performed at the end of administration.

Time course of summary statistics of physical examination parameters are shown in Table 14.3–9. A list of measurement values of physical examination by patient is provided in Appendix 16.2.8f.

Mean measurement values for each physical examination parameter assessed for treatment with the study drug were as follows. Systolic blood pressure was between 99.6 to 111.3 mmHg, diastolic blood pressure was between 53.2 to 63.1 mmHg, pulse rate was between 75.6 to 88.3 beats/min, and the body temperature was between 36.45 to 37.06°C. Among all of the parameters, no significant changes were observed compared to the mean values measured before administration at Week 0.

Throughout the evaluation period, as events related to physical examinations, 'Blood pressure decreased' were observed in 4.8% (1/21) of patients. This event was judged before administration of the study drug on the examination day and a causal relationship of this event with the study drug was ruled out.

12.5.2 Immunoserological Tests

The distribution of determination results of immunoserological tests (anti-nuclear antibody, double stranded DNA IgG antibody and double stranded DNA IgM antibody) at each evaluation time point is shown in Table 12.5–1. A list of laboratory values of immunoserological tests by patient and a list of AEs related to immunoserological tests by patient are shown respectively in Appendix 16.2.8e and Appendix 16.2.7a.

Table 12.5–1 Immunoserological Tests

		Week 14		Week 30		All time points after administration*	
		Negative	Positive	Negative	Positive	Negative	Positive
Anti-nuclear antibody	Negative at Week 0	7	2	5	1	7	2
	Positive at Week 0	1	10	1	7	1	11
Double stranded DNA IgG antibody	Negative at Week 0	20		14		21	
	Positive at Week 0						
Double stranded DNA IgM antibody	Negative at Week 0	11	8	4	10	9	11
	Positive at Week 0		1				1

*Positive: Positive at all of the measurement points (Weeks 14 and 30) in patients who completed evaluation at Week 30 and the measurement points (Day of discontinuation/ 8 weeks after last administration, or Week 14/ day of discontinuation/ 8 weeks after last

administration) in discontinued patients. Positive at the measurement point (Week 14) in CAI score-based non-responders
Negative: Negative at all time points

12.5.2.1 Anti-Nuclear Antibody

The distribution of patients who tested negative/positive for anti-nuclear antibody at each evaluation time point by negative/positive result at the starting day of study drug administration (Week 0) is shown in Table 12.5–1.

Among 9 patients who tested negative at Week 0, 2 patients tested positive at either of the time points after administration.

12.5.2.2 Double stranded DNA Antibody

The distribution of patients who tested negative/positive for double stranded DNA antibodies (IgG antibody and IgM antibody) at each evaluation time point by negative/positive result at the starting day of study drug administration (Week 0) is shown in Table 12.5–1.

Among patients who tested negative for double stranded DNA IgG antibody at Week 0, no patients tested positive at either of the time points after administration. Among 20 patients who tested negative for double stranded DNA IgM antibody at Week 0, 11 patients tested positive at either of the time points after administration.

12.6 Safety Conclusions

Safety evaluation was performed on 21 patients who received the study drug at least once and from whom safety data after the start of administration of the study drug were obtained. Based on the evaluation, the following conclusions were obtained.

(1) The incidence rates of AEs and ADRs were respectively 95.2% (20/21) and 71.4% (15/21). The system organ classes (SOCs) of AEs having the highest incidence rate were “infections and infestations” and “investigations” each having an incidence rate of 57.1% (12/21), and “gastrointestinal disorders” with an incidence rate of 52.4% (11/21). The AEs with a high incidence rate were “double stranded DNA antibody positive” having an incidence rate of 57.1% (12/21) and “nasopharyngitis” having an incidence rate of 33.3% (7/21). The SOCs of ADRs having a high incidence rate was “investigations” having an incidence rate of 57.1% (12/21), and the ADR having a high incidence rate was “double stranded DNA antibody positive” having an incidence rate of 57.1% (12/21).

(2) The incidence rates of serious AEs and serious ADRs were respectively 14.3% (3/21) and 4.8% (1/21). The event “colitis ulcerative” (recorded as “worsened ulcerative colitis” by the investigator) occurred in 2 patients, but a causal relationship of this event with the study drug was ruled out. As a serious ADR, “enterocolitis” was observed. This event was moderate and recovery occurred without any treatment.

(3) The incidence rate of AEs leading to discontinuation of the study was 4.8% (1/21), and the event was “colitis ulcerative” (recorded as “worsened ulcerative colitis” by the investigator). No ADRs leading to discontinuation of the study were observed.

(4) The incidence rate of infections and infections (ADRs) were respectively 61.9% (13/21) and 23.8% (5/21). The event having the highest incidence rate was “nasopharyngitis” having an incidence rate of 33.3% (7/21). The incidence rate of serious infections was 4.8% (1/21), and the only one event was “enterocolitis.” No infections leading to discontinuation of treatment were observed.

(5) The incidence rates of infusion reactions and infusion reactions (ADRs) were both 9.5% (2/21). The events were moderate and mild, each observed in one patient, but recovery of both events was achieved on the same day they occurred. No serious infusion reactions and infusion reactions leading to discontinuation of treatment were observed.

(6) The incidence rate of immunoserological test-related AEs was 57.1% (12/21), and the incidence rates of “double stranded DNA (IgM) antibody positive” and “antinuclear antibody increased” were respectively 57.1% (12/21) and 4.8% (1/21). No patients were observed who experienced a change from negative to positive to double stranded DNA IgG antibody, which is said to be correlated with lupus-like syndrome. Furthermore, lupus-like syndrome did not occur in any patients.

(7) No events to be noted related to the study drug such as malignancy (including hepatosplenic T-cell lymphoma which has been reported in children and young adults), demyelinating disorders, interstitial pneumonia, liver dysfunction, delayed hypersensitivity (including serum sickness-like reaction), congestive cardiac failure, serious blood disorder and rhabdomyolysis, were observed.

(8) There were 7 laboratory parameters where the incidence rate of abnormal change in general laboratory values was not less than 10%, including urine protein (increase) and urinary occult blood (increase) each having an incidence rate of 47.6% (10/21), ALT (GPT) (decrease) having an incidence rate of 33.3% (7/21), white blood cell count (decrease), ALP (decrease), γ-GTP (decrease) and BUN (decrease) each having an incidence rate of 14.3% (3/21). No changes in laboratory parameters which would cause clinical problems were observed.

(9) The changes in summary statistics of physical examinations (systolic and diastolic blood pressure, pulse rate, and body temperature) did not show any problematic tendencies. As an AE related to physical examinations, “Decreased blood pressure” occurred in 4.8% (1/21) of patients, but a causal relationship of this event with the study drug was ruled out.

Based on the results from the evaluation of safety of the study drug for 30 weeks when administered to pediatric patients with moderate to severe ulcerative colitis up to Week 22, it was confirmed that this study drug was mostly tolerable. In addition, similarly to the safety profiles for the approved indications including ulcerative colitis in adults, some events related to infections, infusion reactions, and immunoserological tests were observed. There was no significant increase in the frequency and severity of the events of concern related to the study drug administration, and no new events to be noted were observed.

13. Discussion and Overall Conclusions

Ulcerative colitis is a diffuse nonspecific inflammatory disease of unknown etiology primarily affecting the mucosa membrane of the large intestine where erosion and ulcers are often formed, and the number of patients with ulcerative colitis has been increasing. The number of pediatric patients with onset in childhood is smaller than that of adult patients but has continued to increase similarly to adult patients. Primary treatment of ulcerative colitis is drug therapy, but there exist numerous cases where patients are not adequately responding to treatment with existing drugs and are eventually referred for surgery. Furthermore, pediatric ulcerative colitis is characterized by a higher likelihood of extended and/or severer lesions compared to adults, and steroid-related complications such as failure to thrive and steroid dependence caused by long-term treatment with steroids, and these characteristics become a problem in steroid therapy. Against this background, TA-650 was approved for use in patients with ulcerative colitis in Japan in June 2010, and was ranked as a therapeutic drug for ulcerative colitis to be used in steroid-dependent or resistant patients. However, no domestic clinical trials of TA-650 in pediatric patients with ulcerative colitis have been conducted, and “children” is not clearly specified in the description of dosage and administration, therefore physicians may hesitate to use TA-650 in pediatric patients with ulcerative colitis, who require administration of this drug, and this becomes a problem. In foreign countries, a Phase III study (Study No. C0168T72) was conducted on patients aged 6 to 17 years. As a result of this study, the efficacy and safety of TA-650 was confirmed when administered at a dose of 5 mg/kg at Weeks 0, 2 and 6, followed by administration at 8-week intervals, and TA-650 has been approved in Europe and US. Therefore a domestic clinical study of TA-650 in pediatric patients with ulcerative colitis was conducted to evaluate the efficacy, safety and pharmacokinetics of TA-650 when administered at a dose of 5 mg/kg at Weeks 0, 2 and 6 followed by administration at 8-week intervals at Weeks 14 and 22.

In this study, 21 patients received the study drug. The CAI score started to decrease from Week 2, and decreased over time at Weeks 6 and 8, and the decreased CAI score continued to be observed after Week 10 and afterward. In addition, decreases in partial Mayo score and PUCAI score with time until Week 8 and decreased scores after Week 10 were observed. In terms of CAI score-based remission, at least a half of the patients were judged to achieve remission at Week 2, and it was confirmed that this high remission rate was maintained until Week 30. In terms of PUCAI score-based remission, though the PUCAI score-based remission rate was not as high as the CAI score-based remission rate, some patients were judged to achieve remission at Week 2, and it was confirmed that the remission was maintained until Week 30. In addition, since it has been reported that a decrease in PUCAI score by at least 20 points is clinically significant, the proportion of patients who achieved a decrease in PUCAI score by at least 20 points was also evaluated. As a result, at least a half of the patients achieved a decrease in PUCAI score by at least 20 points at Week 2, and it was confirmed that this effect was maintained until Week 30. Based on the above results, it was suggested that TA-650 had the effect of improving the symptoms or achieving remission, within an early period after administration of Week 2, and the effect was maintained by continuous treatment with the study drug.

Sigmoidoscopy is necessary to determine a Mayo score, but sigmoidoscopy is a highly invasive examination for patients, therefore this examination was only to be performed in this study where possible. Only 8 patients were able to be evaluated by the Mayo score, which was less than a half of the patients receiving the study drug, but a decrease in the Mayo score was observed at Week 30. In addition, some patients achieved Mayo score-based response and Mayo score-based remission. Furthermore, it

was observed that 2 patients achieved mucosal healing at Week 30, suggesting that TA-650 had the effect of mucosal healing.

Use of steroids causes failure to thrive, a significant problem specific to children in addition to the ADRs which have been reported for adults. In the guideline for treatment of pediatric ulcerative colitis among the Guidelines for the Treatment of Ulcerative Colitis/Crohn's Disease (the version revised in 2014), it is described that steroids should not be used for the purpose of remission maintenance as steroids may cause failure to thrive, but the guideline also reports there are patients who failed to achieve steroid withdrawal due to exacerbation or relapse of symptoms associated with a reduction of steroid dose. In this study, 76.2% (16/21) of patients, more than a half, were judged as those who failed to achieve steroid withdrawal at the time of registration. In 12 patients who had been using steroids (oral preparations) at the time of registration, the steroid dose decrease began from Week 2, and continued to decrease over time at Weeks 6 and 8. The reduced steroid dose was also observed after Week 10. Two patients achieved steroid withdrawal at Week 30, and one of these patients achieved CAI score-based remission. Based on the above results, it was suggested that continuous treatment with TA-650 would enable reduction of steroid dose or steroid withdrawal.

The trough serum infliximab concentration in CAI score-based responders indicated maintenance of serum infliximab concentration, and the serum infliximab concentration 1 hour after completion of administration in CAI score-based responders was not largely different from the trough concentration, therefore multiple doses of TA-650 were not considered to cause accumulation of the drug. In addition, CAI score changes by serum infliximab concentration at Week 30 showed a tendency for the study drug to prove more effective the higher the serum infliximab concentration was, though some subgroups contained a small number of patients by serum infliximab concentration.

In terms of safety, the incidence rates of AEs and ADRs were respectively 95.2% (20/21) and 71.4% (15/21). The SOCs of AEs with a particularly high incidence rate were "infections and infestations" and "investigations." The only serious AE other than "colitis ulcerative," (a worsened primary disease) that was observed, was "enterocolitis." This event was judged as an infection, but it did not lead to discontinuation of the study because the relevant patient recovered without any treatment. In contrast, the AE leading to discontinuation was "colitis ulcerative" which occurred in 1 patient, and no ADRs leading to discontinuation were observed, and therefore TA-650 was considered mostly tolerable. In addition, no events to be noted related to the study drug, such as malignancy (including hepatosplenic T-cell lymphoma which has been reported in children and young adults), demyelinating disorders, interstitial pneumonia, liver dysfunction, delayed hypersensitivity (including serum sickness-like reaction), congestive cardiac failure, serious blood disorder and rhabdomyolysis, were observed.

Based on the above results, no significant differences from the safety profiles reported for the approved indications including ulcerative colitis in adults were observed, and the frequency and severity of the events which were of concern when the study drug was administered were not increased significantly, and no new events to be noted were observed.

As described above, administration of TA-650 to pediatric patients with ulcerative colitis at a dose of 5 mg/kg at Weeks 0, 2 and 6 demonstrated the effect of improving symptoms of ulcerative colitis. Furthermore, continuous administration of the drug at 8-week intervals at Weeks 14 and 22 was shown to maintain the therapeutic effect of the study drug and also suggested there was a mucosal healing effect and that the study drug might lead to a reduction of steroid dose or steroid withdrawal. In terms of safety,

TA-650 was mostly tolerable having a safety profile similar to the one which has been known for the drug, and no new events to be noted were observed.

14. Tables, Figures and Graphs Referred to but Not Included in the Text

14.1 Demographic Data

Not applicable.

14.2 Efficacy Data

Not applicable.

14.3 Safety Data

14.3.1 Displays of Adverse Events

Table 14.3–1 Adverse Events by Severity (Other Than Immunoserological Tests)

	(N=21)					
	Mild		Moderate		Severe	
	n	(%)	n	(%)	n	(%)
Total	13	(61.9)	5	(23.8)	1	(4.8)
Infections and infestations	11	(52.4)	1	(4.8)	0	(0.0)
Bronchitis	0	(0.0)	1	(4.8)	0	(0.0)
Nasopharyngitis	7	(33.3)	0	(0.0)	0	(0.0)
Upper respiratory tract infection	2	(9.5)	0	(0.0)	0	(0.0)
Enteritis infectious	2	(9.5)	0	(0.0)	0	(0.0)
Gastroenteritis	1	(4.8)	0	(0.0)	0	(0.0)
Sinusitis	1	(4.8)	0	(0.0)	0	(0.0)
Subcutaneous abscess	1	(4.8)	0	(0.0)	0	(0.0)
Blood and lymphatic system disorders	2	(9.5)	1	(4.8)	0	(0.0)
Iron deficiency anaemia	1	(4.8)	1	(4.8)	0	(0.0)
Anaemia	1	(4.8)	0	(0.0)	0	(0.0)
Nervous system disorders	1	(4.8)	0	(0.0)	0	(0.0)
Headache	1	(4.8)	0	(0.0)	0	(0.0)
Eye disorders	1	(4.8)	0	(0.0)	0	(0.0)
Scleritis	1	(4.8)	0	(0.0)	0	(0.0)
Cardiac disorders	1	(4.8)	0	(0.0)	0	(0.0)
Vasculitis	1	(4.8)	0	(0.0)	0	(0.0)
Respiratory, thoracic and mediastinal disorders	4	(19.0)	0	(0.0)	0	(0.0)
Asthma	1	(4.8)	0	(0.0)	0	(0.0)
Rhinitis allergic	1	(4.8)	0	(0.0)	0	(0.0)
Upper respiratory tract inflammation	1	(4.8)	0	(0.0)	0	(0.0)
Oropharyngeal pain	1	(4.8)	0	(0.0)	0	(0.0)
Gastrointestinal disorders	6	(28.6)	4	(19.0)	1	(4.8)
Colitis ulcerative	0	(0.0)	1	(4.8)	1	(4.8)
Nausea	1	(4.8)	1	(4.8)	0	(0.0)
Enterocolitis	0	(0.0)	1	(4.8)	0	(0.0)
Autoimmune pancreatitis	0	(0.0)	1	(4.8)	0	(0.0)
Vomiting	2	(9.5)	0	(0.0)	0	(0.0)
Abdominal pain lower	1	(4.8)	0	(0.0)	0	(0.0)
Abdominal pain upper	1	(4.8)	0	(0.0)	0	(0.0)
Cheilitis	1	(4.8)	0	(0.0)	0	(0.0)
Constipation	1	(4.8)	0	(0.0)	0	(0.0)
Pancreatitis	1	(4.8)	0	(0.0)	0	(0.0)
Skin and subcutaneous tissue disorders	2	(9.5)	0	(0.0)	0	(0.0)
Acne	2	(9.5)	0	(0.0)	0	(0.0)
Pruritus	1	(4.8)	0	(0.0)	0	(0.0)
Musculoskeletal and connective tissue disorders	2	(9.5)	1	(4.8)	0	(0.0)
Myalgia	0	(0.0)	1	(4.8)	0	(0.0)
Arthralgia	1	(4.8)	0	(0.0)	0	(0.0)
Back pain	1	(4.8)	0	(0.0)	0	(0.0)
General disorders and administration site conditions	2	(9.5)	0	(0.0)	0	(0.0)
Chest discomfort	1	(4.8)	0	(0.0)	0	(0.0)
Infusion site pain	1	(4.8)	0	(0.0)	0	(0.0)
Investigations	2	(9.5)	0	(0.0)	0	(0.0)
Blood pressure decreased	1	(4.8)	0	(0.0)	0	(0.0)
Protein urine present	1	(4.8)	0	(0.0)	0	(0.0)
Injury, poisoning and procedural complications	1	(4.8)	0	(0.0)	0	(0.0)
Thermal burn	1	(4.8)	0	(0.0)	0	(0.0)

Table 14.3–2 Adverse Events by Severity (Other Than Immunoserological Tests)

	(N=21)					
	Mild		Moderate		Severe	
	n	(%)	n	(%)	n	(%)
Total	5	(23.8)	4	(19.0)	0	(0.0)
Infections and infestations	3	(14.3)	1	(4.8)	0	(0.0)
Bronchitis	0	(0.0)	1	(4.8)	0	(0.0)
Nasopharyngitis	2	(9.5)	0	(0.0)	0	(0.0)
Subcutaneous abscess	1	(4.8)	0	(0.0)	0	(0.0)
Enteritis infectious	1	(4.8)	0	(0.0)	0	(0.0)
Blood and lymphatic system disorders	1	(4.8)	0	(0.0)	0	(0.0)
Iron deficiency anaemia	1	(4.8)	0	(0.0)	0	(0.0)
Gastrointestinal disorders	0	(0.0)	3	(14.3)	0	(0.0)
Enterocolitis	0	(0.0)	1	(4.8)	0	(0.0)
Nausea	0	(0.0)	1	(4.8)	0	(0.0)
Autoimmune pancreatitis	0	(0.0)	1	(4.8)	0	(0.0)
Skin and subcutaneous tissue disorders	1	(4.8)	0	(0.0)	0	(0.0)
Acne	1	(4.8)	0	(0.0)	0	(0.0)
Musculoskeletal and connective tissue disorders	1	(4.8)	1	(4.8)	0	(0.0)
Myalgia	0	(0.0)	1	(4.8)	0	(0.0)
Arthralgia	1	(4.8)	0	(0.0)	0	(0.0)
General disorders and administration site conditions	1	(4.8)	0	(0.0)	0	(0.0)
Infusion site pain	1	(4.8)	0	(0.0)	0	(0.0)

Table 14.3–3 Serious Infections

	(N=21)		
	n	(%)	No. of events
Total	1	(4.8)	1
Gastrointestinal disorders	1	(4.8)	1
Enterocolitis	1	(4.8)	1

Table 14.3–4 Serious Infections (ADRs)

	(N=21)		
	n	(%)	No. of events
Total	1	(4.8)	1
Gastrointestinal disorders	1	(4.8)	1
Enterocolitis	1	(4.8)	1

14.3.2 Listing of Deaths, Other Serious Adverse Events and Other Significant Adverse Events

Not applicable.

14.3.3 Narratives of Deaths, Other Serious and Certain Other Significant Adverse Events

Table 14.3–5 Narratives of Serious Adverse Events

Patient ID No.	Sex<br>Age*	Daily dose<br>Date of administration	Symptoms recorded in CRF	Worsened ulcerative colitis	Outcome<br>(Date of outcome)	Investigator’s comment
			Date	Course and treatment		
TA-650UC-011-01	Male<br>13 years	5 mg/kg<br>12/25/2012<br>01/09/2013<br>02/06/2013	11/2012	Relapse of ulcerative colitis, admitted to a neighbor hospital, placed at rest and received a conservative therapy.	Recovered<br>(04/17/2013)	This event is considered to be worsened ulcerative colitis, the primary disease, and it is judged that there was no causal relationship of this event with the study drug.
			12/12/2012	Admitted to our hospital to receive a treatment.		
			12/14/2012	The consent for this study was obtained.		
			12/25/2012	Start of administration of TA-650 at a dose of 5 mg/kg. No particular problems after administration.		
			01/09/2013	Administration of TA-650 at a dose of 5 mg/kg at Week 2. No particular problems after administration. Discharged from the hospital.		
			02/20/2013	Examinations at Week 8 as an outpatient. Achieved remission.		
			03/05/2013	Examinations at Week 10 as an outpatient. The patient complained of abdominal pain, increased stool frequency (5 to 6 diarrhea per day) and blood in stool. Relapse of ulcerative colitis. Endoscopic assessment was judged to be necessary.		
			03/06/2013	Admitted to the hospital.		
			03/08/2013	Endoscopy was performed. Relapse of ulcerative colitis was confirmed and participation in the clinical study was discontinued. Start of treatment with Predonine injection (20 mg/day) from the evening.		
			03/09/2013	Dose increase to 40 mg/day of Predonine injection. Stool frequency was twice.		
			03/10/2013	Start of treatment with Altat capsule (75 mg/day).		
			03/11/2013	The first LCAP was performed. Completed without any problems.		
			03/12/2013	Start of treatment with Bredinin tablet (300 mg/day).		
			03/15/2013	The second LCAP was performed. The patient complained of pain and redness on the left elbow, but these symptoms disappeared immediately. Stool frequency was one time.		
			03/18/2013	The third LCAP was performed. Pain and redness were observed on the left elbow but disappeared when returned to the patient’s room.		
			03/19/2013	Treatment with Predonine injection (40 mg/day) was changed to treatment with prednisolone tablet (40mg/day). Start of treatment with Imuran tablet (50 mg/day).		
			03/22/2013	The forth LCAP was performed. Pain and redness on the left elbow.		
			03/25/2013	The fifth LCAP was performed. No pain and redness were observed.		
			03/28/2013	The sixth LCAP was performed. No pain and redness were observed. Stool frequency was 0 to 1 time. Discharged from the hospital, and followed up as an outpatient.<br>Dose increase of Bredinin tablet from 300 mg/day to 400 mg/day. Dose decrease of Prednisolone tablet from 40 mg/day to 30mg/day. Instruction for dose decrease to 20 mg/day from April 6.		
			04/17/2013	Confirmed that the patient achieved remission at the time of clinic visit.		
<Causal relationship with the study drug> Can be ruled out.						

* Age at the time of onset of the serious AE.

Table 14.3–5 Narratives of Serious Adverse Events (Continued)

Patient ID No.	Sex<br>Age*	Daily dose<br>Date of administration	Symptoms recorded in CRF	Worsened ulcerative colitis	Outcome<br>(Date of outcome)	Investigator's comment
			Date	Course and treatment		
TA-650UC-024-01	Female<br>17<br>years	5 mg/kg<br>08/17/2012<br>08/31/2012<br>09/28/2012	08/01/2012	Obtainment of consent for participation in the study.	Recovered<br>(01/04/2013)	This event was observed in a non-responder who completed study drug administration on September 28.<br>This event is considered to be worsened ulcerative colitis, the primary disease, and it is judged that there was no causal relationship of this event with the study drug.
			08/17/2012	Administration of the study drug (08/17/2012- 09/28/2012).		
			10/15/2012	The patient was judged as a CAI score-based non-responder at the evaluation at Week 8.		
			10/24/2012	Visit to the hospital for evaluation at Week 10. Increased stool frequency, mushy stools and pyrexia (38.0 °C) were observed. The symptoms were considered to be due to worsened ulcerative colitis, and the dose of oral prednisolone was increased.		
			10/31/2012	Visit to the hospital due to abdominal pain and bleeding. It was decided to start cytapheresis from the next week on a twice-weekly basis.		
			11/06/2012	Cytapheresis was performed. Pyrexia of 39.0 to 39.99 °C had been observed from the previous day.<br>Since exacerbation of the symptoms was too drastic to wait for the patient's response to cytapheresis, it was planned to admit the patient to the hospital to give a combination of steroid pulse and antibiotics.		
			11/08/2012	Admission to the hospital. Start of steroid pulse therapy.		
			11/13/2012	There were still symptoms but they were improving.		
			11/22/2012	Cytapheresis (LCAP) was performed. LCAP was discontinued thereafter due to occurrence of hemolysis.		
			11/27/2012	GCAP was resumed.		
			11/28/2012	Since no improvement of the symptoms was observed, administration of oral tacrolimus was started. Colonoscopy was performed.<br>Diffuse deep ulcer and pseudopolyposis were found. Hemorrhagic.<br>Surgery was considered.		
			12/05/2012	Persistent blood in stools, frequent diarrhea and increased nocturnal abdominal pain		
			12/06/2012	Administration of all oral preparations was stopped. Oral intake of water and diet was stopped.		
			12/10/2012	Transferred to another hospital to undergo surgery.		
			12/14/2012	Surgery (subtotal removal of the large intestine and ileostomy) was performed.		
			12/28/2012	Discharged from the hospital.		
			01/04/2013	Visit our hospital as an outpatient. Recovery from this event was confirmed.		
<Causal relationship with the study drug> Can be ruled out.						

* Age at the time of onset of the serious AE.

Table 14.3–5 Narratives of Serious Adverse Events (Continued)

Patient ID No.	Sex<br>Age*	Daily dose<br>Date of administration	Symptoms recorded in CRF: Date	Enteritis<br>Course and treatment	Outcome<br>(Date of outcome)	Investigator's comment
TA-650PS-010-02	Male<br>14<br>years	5 mg/kg<br>11/25/2013<br>12/09/2013<br>01/06/2014<br>02/28/2014<br>04/28/2014	03/31/2014	Visit at Week 18. A slight increase in CRP was observed, but it was decided to follow up on the patient.	Recovered<br>(05/21/2014)	During the maintenance therapy with Remicade®, the patient's case was complicated by transient enteritis associated with pyrexia and increased CRP, and the enteritis was relieved without any change of antibiotics or therapeutic drugs for ulcerative colitis.<br>Based on the clinical course, adenoviral enteritis was largely suspected, but it was impossible to diagnose the patient as having an adenoviral infection from the feces and pharynx examinations.<br>After the onset on March 31, the peak in symptoms of enteritis was observed on April 3, and tendency to decreased stool frequency and reduced body temperature was observed when the patient visited the hospital on April 4. However, since the patient experienced awakening for defecation during sleep, the patient was admitted to the hospital to control the symptoms. After admission, the patient achieved remission during the natural course of the disease, and was discharged from the hospital on April 9.<br>Considering the course of the disease, enteritis due to some infection was mostly suspected. The patient achieved remission without antibiotic therapy.<br>This event was considered to possibly occur without administration of the study drug, but it cannot be ruled out that the onset of the event might be related to the study drug. However, since the remission of the event did not require a dose decrease or discontinuation of the study drug or an immunomodulator (azathioprine), there are considered no problems to continue to use of the study drug as the therapeutic drug for the primary disease.
			04/01/2014	Stool frequency was 10 times.		
			04/02/2014	Pyrexia (38.8 °C). Achieved remission by treatment with Calonal. Stool frequency was 15 times.		
			04/03/2014	Pyrexia (39 °C). Onset of vomiting at 6 p.m., and vomited 5 to 6 times. Stool frequency was at least 30 times.		
			04/04/2014	The body temperature was reduced, but the patient had several stools per hour. No nausea. Visited the hospital as an outpatient.<br>The patient was admitted to the hospital due to possible complication of enteritis.<br>Intake of fluid replacement and water, and followed up.		
			04/06/2014	Negative for adenovirus (feces, pharynx). Negative for CD toxin. Negative in fecal culture test.		
			04/07/2014	Decreased CRP. Stool frequency was decreased to 12 times, and tendency for improvement of symptoms was observed. No blood in stools.		
			04/08/2014	Stool frequency was 8 times. No blood in stools.		
			04/09/2014	Due to improvement of general condition, the patient was discharged from the hospital.		
			04/14/2014	Stool frequency was 8 times. No blood in stools. The condition of the patient was stable, but number of stools was high.		
			04/28/2014	Stool frequency was 6 times. No blood in stools. Diarrhea but sometimes formed stools. Administration of the study drug at Week 22.		
			05/21/2014	Stool frequency was 3 to 4 times. No blood in stool and abdominal pain. Diarrhea. The general condition was stable, and the laboratory values were improved, therefore recovery from the AE was judged.		

<Causal relationship with the study drug> Cannot be ruled out.

* Age at the time of onset of the serious AE.

14.3.4 List of Individual Laboratory Measurements by Patient and Each Abnormal Laboratory Value

Table 14.3–6 The incidence rates of abnormal changes in laboratory values

	Abnormal increase			Abnormal decrease		
	n	Increase	%	n	Decrease	%
RBC count (×10^4/μL)	21	1	4.8	21	0	0.0
Hemoglobin (g/dL)	21	0	0.0	21	0	0.0
Hematocrit (%)	21	0	0.0	21	0	0.0
WBC count (/μL)	21	2	9.5	21	3	14.3
Platelet count (×10^4/μL)	20	1	5.0	20	1	5.0
AST (GOT) (U/L)	21	2	9.5	21	1	4.8
ALT (GPT) (U/L)	21	1	4.8	21	7	33.3
ALP (U/L)	21	0	0.0	21	3	14.3
LDH (U/L)	21	1	4.8	21	2	9.5
γ -GTP (U/L)	21	2	9.5	21	3	14.3
Total protein (g/dL)	21	0	0.0	21	0	0.0
Albumin (g/dL)	21	2	9.5	21	1	4.8
Total cholesterol (mg/dL)	21	1	4.8	21	1	4.8
Total bilirubin (mg/dL)	21	1	4.8	21	2	9.5
BUN(mg/dL)	21	2	9.5	21	3	14.3
Serum creatinine (mg/dL)	21	0	0.0	21	1	4.8
Na (mEq/L)	21	0	0.0	21	0	0.0
K (mEq/L)	21	0	0.0	21	0	0.0
Cl (mEq/L)	21	0	0.0	21	0	0.0
Urine protein (Qualitative)	21	10	47.6	21	0	0.0
Urine glucose (Qualitative)	21	0	0.0	21	0	0.0
Occult blood in urine (Qualitative)	21	10	47.6	21	0	0.0
Urobilinogen (Qualitative)	21	0	0.0	21	0	0.0

Table 14.3–7 Summary Statistics of Hematology and Blood Biochemistry

		n	Median	Q1	Q3	Min	Max
RBC count (x10⁴/μL)	Week 0	21	414.0	389.0	463.0	275	510
	Week 2	20	435.5	403.0	461.0	278	509
	Week 6	20	450.0	385.5	486.5	258	534
	Week 8	19	444.0	385.0	494.0	263	509
	Week 10	19	466.0	407.0	506.0	268	557
	Week 14	20	466.5	394.5	495.0	266	553
	Week 18	16	465.5	425.5	496.5	323	550
	Week 22	16	473.0	418.5	485.5	307	553
	Week 26	14	480.0	437.0	502.0	312	511
	Week 30	14	473.5	455.0	513.0	289	543
Hemoglobin (g/dL)	Week 0	21	11.80	10.80	12.60	9.0	14.0
	Week 2	20	12.05	10.60	12.35	9.4	14.1
	Week 6	20	11.85	11.25	13.10	8.8	13.6
	Week 8	19	12.10	10.70	13.00	8.2	14.4
	Week 10	19	12.50	11.20	13.50	9.2	15.3
	Week 14	20	11.95	11.20	13.60	8.2	14.8
	Week 18	16	12.15	11.35	13.40	9.5	15.4
	Week 22	16	12.25	11.05	13.85	9.1	15.2
	Week 26	14	12.30	11.40	14.40	10.0	14.8
	Week 30	14	12.20	11.40	14.80	10.2	15.9
Hematocrit (%)	Week 0	21	35.10	32.60	38.00	28.9	41.6
	Week 2	20	36.45	33.00	38.50	29.1	41.8
	Week 6	20	36.90	33.60	39.10	28.2	42.3
	Week 8	19	37.60	31.50	39.20	25.4	43.6
	Week 10	19	38.30	36.10	40.10	27.8	45.7
	Week 14	20	36.80	34.40	39.95	24.5	44.8
	Week 18	16	37.60	34.55	40.15	29.6	46.2
	Week 22	16	37.35	34.75	41.00	29.1	45.1
	Week 26	14	37.60	35.80	41.90	31.8	43.6
	Week 30	14	37.35	34.80	43.30	30.0	47.4
WBC count (/μL)	Week 0	21	8600.0	6400.0	10500.0	4200	17000
	Week 2	20	6750.0	5850.0	7700.0	4300	14300
	Week 6	20	7150.0	5850.0	7850.0	4800	9300
	Week 8	19	6800.0	4700.0	8200.0	3400	9900
	Week 10	19	7300.0	5300.0	8400.0	3800	10600
	Week 14	20	7250.0	6100.0	8900.0	3400	13900
	Week 18	16	5900.0	4950.0	6950.0	3600	8200
	Week 22	16	6500.0	5100.0	8700.0	4000	12000
	Week 26	14	5800.0	5100.0	7200.0	3600	11800
	Week 30	14	6700.0	5400.0	8100.0	4200	18100

Table 14.3–7 Summary Statistics of Hematology and Blood Biochemistry (Continued)

		n	Median	Q1	Q3	Min	Max
Neutrophils (%)	Week 0	21	69.00	57.40	80.00	35.0	85.0
	Week 2	20	60.65	49.15	69.45	23.9	87.0
	Week 6	20	53.05	42.00	64.00	29.5	87.5
	Week 8	19	49.60	33.40	63.00	21.6	80.5
	Week 10	19	54.50	40.50	66.50	27.1	84.0
	Week 14	20	57.35	52.30	70.25	32.2	86.5
	Week 18	16	51.05	44.35	58.30	29.0	69.5
	Week 22	16	58.40	51.90	63.55	32.3	81.5
	Week 26	14	48.90	42.00	63.90	35.6	67.6
	Week 30	14	56.45	52.60	62.80	41.0	78.5
Eosinophils (%)	Week 0	21	3.60	1.00	6.00	0.0	18.5
	Week 2	20	3.50	1.60	4.75	0.0	14.0
	Week 6	20	3.50	1.00	7.35	0.3	22.0
	Week 8	19	3.60	2.00	6.00	0.9	29.5
	Week 10	19	3.00	1.00	7.70	0.3	13.3
	Week 14	20	2.40	1.00	5.60	0.0	17.5
	Week 18	16	3.40	1.80	5.05	1.0	19.5
	Week 22	16	3.45	2.15	4.85	0.5	9.5
	Week 26	14	3.00	1.80	4.90	0.1	11.0
	Week 30	14	3.95	2.00	6.00	0.0	13.5
Basophils (%)	Week 0	21	0.00	0.00	0.40	0.0	3.0
	Week 2	20	0.50	0.30	0.70	0.0	1.0
	Week 6	20	0.35	0.00	0.75	0.0	3.0
	Week 8	19	0.50	0.40	0.90	0.0	1.5
	Week 10	19	0.40	0.30	0.50	0.0	1.2
	Week 14	20	0.50	0.35	0.70	0.0	1.2
	Week 18	16	0.60	0.35	0.85	0.0	1.5
	Week 22	16	0.50	0.20	0.65	0.0	1.0
	Week 26	14	0.50	0.30	0.70	0.0	1.0
	Week 30	14	0.05	0.00	0.50	0.0	0.7
Monocytes (%)	Week 0	21	6.50	5.00	8.50	4.0	13.5
	Week 2	20	6.50	4.50	7.30	3.0	12.5
	Week 6	20	7.85	5.35	9.00	3.8	13.0
	Week 8	19	7.00	6.00	10.50	4.0	24.0
	Week 10	19	8.30	6.10	9.50	5.0	15.0
	Week 14	20	6.50	5.20	9.05	4.4	12.1
	Week 18	16	9.25	5.95	10.25	4.1	12.5
	Week 22	16	7.10	5.35	8.10	3.5	10.0
	Week 26	14	7.70	5.50	11.70	4.8	14.0
	Week 30	14	5.75	4.10	9.50	3.0	13.0

Table 14.3–7 Summary Statistics of Hematology and Blood Biochemistry (Continued)

		n	Median	Q1	Q3	Min	Max
Lymphocytes (%)	Week 0	21	18.00	14.00	24.80	5.5	49.6
	Week 2	20	25.55	20.25	34.35	8.5	64.0
	Week 6	20	29.75	22.70	44.60	7.5	64.0
	Week 8	19	34.40	24.60	46.30	7.5	68.0
	Week 10	19	31.90	20.10	42.70	5.5	59.0
	Week 14	20	28.10	20.75	37.50	7.5	59.6
	Week 18	16	36.50	31.15	40.40	17.6	57.0
	Week 22	16	29.55	24.50	36.95	14.5	54.3
	Week 26	14	39.40	26.50	44.70	17.5	54.2
	Week 30	14	31.50	28.10	38.80	16.3	44.2
Platelet count (x10^4/μL)	Week 0	20	36.40	30.40	42.40	18.6	57.6
	Week 2	19	32.00	26.20	39.90	18.6	56.0
	Week 6	19	32.20	24.80	37.80	19.1	48.9
	Week 8	18	28.70	26.10	34.30	15.5	49.2
	Week 10	19	31.20	24.80	36.50	14.3	54.5
	Week 14	19	27.30	23.20	39.10	14.6	43.3
	Week 18	15	29.60	22.90	37.80	15.3	40.4
	Week 22	15	29.60	26.60	35.90	16.3	45.9
	Week 26	13	27.70	26.40	31.60	19.8	42.8
	Week 30	13	27.20	24.60	32.00	16.2	44.3
AST(GOT)(U/L)	Week 0	21	17.0	16.0	20.0	12	47
	Week 2	20	19.0	16.0	22.5	12	30
	Week 6	20	19.0	17.5	21.5	11	26
	Week 8	19	20.0	17.0	22.0	11	26
	Week 10	20	19.5	16.5	21.5	11	35
	Week 14	20	19.0	16.0	21.0	11	25
	Week 18	16	19.5	18.0	22.5	11	49
	Week 22	16	19.5	16.5	21.0	12	32
	Week 26	14	19.5	17.0	23.0	13	30
	Week 30	14	19.0	16.0	21.0	11	31
ALT(GPT)(U/L)	Week 0	21	10.0	8.0	12.0	7	49
	Week 2	20	12.0	9.0	16.5	7	35
	Week 6	20	10.0	8.5	13.5	6	21
	Week 8	19	10.0	8.0	13.0	6	18
	Week 10	20	9.5	7.0	12.5	5	18
	Week 14	20	10.5	9.5	14.5	5	23
	Week 18	16	10.0	8.0	13.0	5	28
	Week 22	16	9.0	7.0	13.0	6	50
	Week 26	14	9.5	8.0	12.0	7	32
	Week 30	14	9.0	8.0	12.0	6	29

Table 14.3–7 Summary Statistics of Hematology and Blood Biochemistry (Continued)

		n	Median	Q1	Q3	Min	Max
ALP (U/L)	Week 0	21	382.0	244.0	472.0	115	785
	Week 2	20	394.5	238.5	460.5	110	990
	Week 6	20	426.5	329.0	566.0	115	1143
	Week 8	19	451.0	278.0	560.0	111	1095
	Week 10	20	470.5	310.5	597.5	108	1057
	Week 14	20	407.5	267.5	657.5	185	823
	Week 18	16	545.0	344.0	744.5	200	948
	Week 22	16	542.5	291.0	708.0	205	910
	Week 26	14	511.5	300.0	699.0	208	980
	Week 30	14	496.0	245.0	611.0	208	833
LDH (U/L)	Week 0	21	179.0	153.0	247.0	126	436
	Week 2	20	187.0	146.0	211.0	137	494
	Week 6	20	184.0	159.5	227.5	143	453
	Week 8	19	192.0	152.0	238.0	133	494
	Week 10	20	195.5	162.5	248.5	119	455
	Week 14	20	181.0	151.5	266.5	132	460
	Week 18	16	199.5	167.0	249.0	128	435
	Week 22	16	185.0	171.0	235.0	136	375
	Week 26	14	188.5	164.0	240.0	146	391
	Week 30	14	186.0	163.0	278.0	126	383
γ-GTP (U/L)	Week 0	21	13.0	9.0	18.0	5	42
	Week 2	20	15.5	11.5	18.5	7	28
	Week 6	20	11.5	9.5	14.5	7	21
	Week 8	19	12.0	8.0	15.0	6	20
	Week 10	20	10.5	8.5	14.5	4	17
	Week 14	20	11.5	8.5	16.5	5	92
	Week 18	16	10.5	8.5	15.0	6	19
	Week 22	16	10.5	8.0	12.5	6	59
	Week 26	14	11.0	7.0	13.0	6	25
	Week 30	14	11.0	8.0	13.0	6	25
Total protein (g/dL)	Week 0	21	7.10	6.80	7.40	6.5	8.4
	Week 2	20	7.35	7.00	7.75	6.8	8.2
	Week 6	20	7.30	7.10	7.65	6.7	8.4
	Week 8	19	7.40	7.00	7.90	6.3	8.2
	Week 10	20	7.45	7.15	7.85	6.4	8.7
	Week 14	20	7.25	6.80	7.75	6.6	8.3
	Week 18	16	7.40	7.05	7.70	6.5	8.5
	Week 22	16	7.10	6.85	7.80	5.8	8.6
	Week 26	14	7.50	7.30	7.90	6.6	8.6
	Week 30	14	7.30	7.20	7.90	7.0	8.5

Table 14.3–7 Summary Statistics of Hematology and Blood Biochemistry (Continued)

		n	Median	Q1	Q3	Min	Max
Albumin (g/dL)	Week 0	21	4.00	3.80	4.40	3.4	4.8
	Week 2	20	4.35	4.20	4.55	3.8	4.9
	Week 6	20	4.25	4.20	4.45	3.9	4.8
	Week 8	19	4.40	4.10	4.50	3.8	4.9
	Week 10	20	4.30	4.15	4.60	3.8	5.0
	Week 14	20	4.25	4.00	4.40	3.5	4.9
	Week 18	16	4.30	4.10	4.50	3.9	5.2
	Week 22	16	4.20	4.05	4.35	3.1	5.0
	Week 26	14	4.40	4.20	4.60	3.8	5.1
	Week 30	14	4.30	4.10	4.40	4.0	5.0
Total cholesterol (mg/dL)	Week 0	21	143.0	120.0	170.0	89	220
	Week 2	20	167.0	146.0	186.0	98	266
	Week 6	20	155.0	127.0	169.5	98	229
	Week 8	19	147.0	126.0	162.0	90	201
	Week 10	20	157.0	131.5	181.0	102	240
	Week 14	20	152.5	137.0	175.5	97	232
	Week 18	16	148.0	125.0	165.5	95	232
	Week 22	16	158.0	130.0	168.0	93	197
	Week 26	14	149.5	142.0	162.0	114	212
	Week 30	14	154.5	140.0	178.0	104	192
Total bilirubin (mg/dL)	Week 0	21	0.40	0.30	0.50	0.2	0.9
	Week 2	20	0.50	0.40	0.65	0.2	1.7
	Week 6	20	0.50	0.35	0.70	0.3	1.0
	Week 8	19	0.50	0.40	0.70	0.2	1.3
	Week 10	20	0.50	0.40	0.75	0.2	1.2
	Week 14	20	0.50	0.30	0.60	0.2	1.3
	Week 18	16	0.40	0.35	0.80	0.3	1.0
	Week 22	16	0.50	0.35	0.75	0.3	1.4
	Week 26	14	0.55	0.30	0.80	0.3	3.0
	Week 30	14	0.60	0.40	0.70	0.3	1.2
BUN (mg/dL)	Week 0	21	9.0	7.0	11.0	5	17
	Week 2	20	12.5	9.0	14.5	6	24
	Week 6	20	11.0	10.0	12.5	5	16
	Week 8	19	11.0	9.0	14.0	6	16
	Week 10	20	11.5	10.0	13.0	7	16
	Week 14	20	11.5	9.5	13.5	8	24
	Week 18	16	11.0	9.0	13.0	6	16
	Week 22	16	11.0	9.5	13.5	7	16
	Week 26	14	11.5	9.0	13.0	7	16
	Week 30	14	13.0	10.0	14.0	6	14

Table 14.3–7 Summary Statistics of Hematology and Blood Biochemistry (Continued)

		n	Median	Q1	Q3	Min	Max
Serum creatinine (mg/dL)	Week 0	21	0.490	0.450	0.560	0.34	0.72
	Week 2	20	0.500	0.460	0.570	0.34	0.70
	Week 6	20	0.495	0.420	0.595	0.32	0.77
	Week 8	19	0.560	0.430	0.590	0.38	0.83
	Week 10	20	0.515	0.460	0.595	0.33	0.76
	Week 14	20	0.485	0.455	0.520	0.34	0.63
	Week 18	16	0.540	0.475	0.585	0.31	0.71
	Week 22	16	0.510	0.415	0.590	0.36	0.72
	Week 26	14	0.565	0.390	0.610	0.31	0.69
	Week 30	14	0.520	0.400	0.590	0.35	0.72
Na (mEq/L)	Week 0	21	139.0	138.0	140.0	134	142
	Week 2	20	139.0	138.0	140.0	136	143
	Week 6	20	140.0	139.0	141.0	137	143
	Week 8	19	140.0	139.0	141.0	138	143
	Week 10	20	139.5	139.0	140.5	136	143
	Week 14	20	139.0	138.0	140.0	136	143
	Week 18	16	140.0	139.0	141.0	137	143
	Week 22	16	140.5	138.5	141.0	136	142
	Week 26	14	140.0	138.0	142.0	137	143
	Week 30	14	140.0	139.0	141.0	136	141
K (mEq/L)	Week 0	21	3.80	3.70	4.10	3.1	4.6
	Week 2	20	3.90	3.70	4.00	3.2	4.3
	Week 6	20	3.80	3.60	4.05	3.5	4.5
	Week 8	19	4.00	3.60	4.10	3.1	4.4
	Week 10	20	4.00	3.80	4.15	3.4	4.4
	Week 14	20	3.95	3.80	4.25	3.2	4.4
	Week 18	16	4.10	3.85	4.20	3.2	4.9
	Week 22	16	3.85	3.70	4.10	3.4	4.5
	Week 26	14	3.90	3.60	4.20	3.3	4.6
	Week 30	14	4.10	3.70	4.30	3.5	4.7
Cl (mEq/L)	Week 0	21	101.0	100.0	103.0	93	105
	Week 2	20	102.0	100.5	102.5	98	104
	Week 6	20	103.0	101.0	104.0	98	105
	Week 8	19	103.0	102.0	105.0	97	107
	Week 10	20	102.5	101.0	104.0	98	107
	Week 14	20	103.0	100.5	104.0	98	108
	Week 18	16	103.0	102.0	104.5	98	106
	Week 22	16	104.0	101.0	105.0	98	107
	Week 26	14	102.5	102.0	104.0	99	105
	Week 30	14	103.0	101.0	104.0	98	105

Table 14.3–8 Frequency Distribution of Determination Results in Urinalysis (Qualitative)

N: Normal, A: Abnormal

		Week 8		Week 14		Week 22		Week 30	
		N	A	N	A	N	A	N	A
Urinary protein (qualitative)	Week 0 N	10	4	13	2	12		7	3
	Week 0 A	4	1	4	1	2	2	2	2
Urinary glucose (qualitative)	Week 0 N	19		20		16		14	
	Week 0 A								
Occult blood (qualitative)	Week 0 N	16	3	19	1	16		13	1
	Week 0 A								

L: Low value, N: Normal, H: High value

		Week 8			Week 14			Week 22			Week 30		
		L	N	H	L	N	H	L	N	H	L	N	H
Urinary urobilinogen (qualitative)	Week 0 L												
	Week 0 N		19			20			16			14	
	Week 0 H												

Table 14.3–9 Summary Statistics of Physical Examinations

				n	Mean	SD	Min	Max
Systolic blood pressure (mmHg)	Week 0	Before administration	Before administration	21	106.3	13.0	88	132
		During administration	After 30 min	21	102.0	11.4	79	122
			After 60 min	21	103.0	11.7	81	129
			After 90 min	21	107.8	11.7	90	133
			After 120 min	21	104.6	10.4	89	125
		After administration	After 30 min	21	107.4	11.2	88	128
			After 60 min	21	107.7	13.0	89	144
			After 90 min	21	105.6	12.9	89	145
			After 120 min	21	106.5	12.8	85	142
	Week 2	Before administration	Before administration	20	103.5	8.0	88	122
		During administration	After 30 min	20	105.3	9.7	88	127
			After 60 min	20	105.1	12.6	86	126
			After 90 min	20	105.9	12.0	89	130
			After 120 min	20	105.2	10.7	89	131
		After administration	After 30 min	20	106.6	13.1	86	135
			After 60 min	20	107.9	15.1	85	133
			After 90 min	20	109.5	13.9	88	136
			After 120 min	20	111.3	15.5	86	145
	Week 6	Before administration	Before administration	20	102.2	11.2	80	126
		During administration	After 30 min	20	101.0	10.6	86	124
			After 60 min	20	101.8	11.7	82	132
			After 90 min	20	100.9	11.5	83	125
			After 120 min	20	102.7	12.3	82	128
		After administration	After 30 min	20	103.9	14.3	81	137
			After 60 min	20	102.9	13.3	75	128
			After 90 min	20	104.5	14.4	81	140
			After 120 min	20	105.5	13.9	81	129
	Week 14	Before administration	Before administration	16	101.2	12.3	84	124
		During administration	After 30 min	16	102.0	11.1	80	124
			After 60 min	16	99.6	15.3	76	134
			After 90 min	16	101.2	10.5	86	122
			After 120 min	16	103.5	12.4	87	124
		After administration	After 30 min	16	101.4	7.0	92	115
			After 60 min	16	105.2	11.5	92	131
			After 90 min	16	103.8	15.1	70	125
			After 120 min	16	109.7	10.1	98	130
	Week 22	Before administration	Before administration	14	106.0	12.3	86	125
		During administration	After 30 min	14	102.4	12.8	86	128
			After 60 min	13	104.3	11.7	87	127
			After 90 min	13	101.5	13.3	83	128
			After 120 min	14	106.4	12.8	88	131
		After administration	After 30 min	14	103.6	15.1	82	137
			After 60 min	14	106.5	12.5	88	132
			After 90 min	14	107.8	14.4	94	140
			After 120 min	14	109.8	14.2	90	139

Table 14.3–9 Summary Statistics of Physical Examinations (Continued)

				n	Mean	SD	Min	Max
Diastolic blood pressure (mmHg)	Week 0	Before administration	Before administration	21	60.7	10.3	42	84
		During administration	After 30 min	21	58.5	8.9	44	81
			After 60 min	21	57.0	10.6	33	77
			After 90 min	21	60.0	9.5	40	77
			After 120 min	21	60.0	8.5	40	75
		After administration	After 30 min	21	62.4	11.2	42	89
			After 60 min	21	60.9	9.0	46	78
			After 90 min	21	58.5	9.9	42	87
			After 120 min	21	56.6	10.1	40	76
	Week 2	Before administration	Before administration	20	56.8	9.0	40	72
		During administration	After 30 min	20	62.4	8.6	45	80
			After 60 min	20	58.4	9.9	38	77
			After 90 min	20	61.0	12.1	41	82
			After 120 min	20	60.2	7.3	41	71
		After administration	After 30 min	20	59.6	8.7	44	74
			After 60 min	20	58.9	10.5	40	83
			After 90 min	20	63.1	10.0	40	80
			After 120 min	20	60.0	9.6	39	73
	Week 6	Before administration	Before administration	20	56.3	7.9	40	74
		During administration	After 30 min	20	54.3	9.8	37	74
			After 60 min	20	55.2	7.8	36	68
			After 90 min	20	56.1	11.5	42	84
			After 120 min	20	56.1	10.9	34	70
		After administration	After 30 min	20	56.8	7.7	45	69
			After 60 min	20	57.5	7.9	44	70
			After 90 min	20	57.6	8.4	37	74
			After 120 min	20	59.4	7.7	44	75
	Week 14	Before administration	Before administration	16	57.4	8.4	40	72
		During administration	After 30 min	16	53.2	7.1	38	69
			After 60 min	16	55.4	16.4	36	106
			After 90 min	16	54.5	6.9	40	65
			After 120 min	16	56.0	10.0	42	79
		After administration	After 30 min	16	56.9	11.9	44	81
			After 60 min	16	58.0	9.1	44	75
			After 90 min	16	57.3	9.8	40	76
			After 120 min	16	59.6	6.8	48	69
	Week 22	Before administration	Before administration	14	57.6	9.3	41	75
		During administration	After 30 min	14	55.4	8.5	43	70
			After 60 min	13	59.2	9.2	43	73
			After 90 min	13	53.9	10.0	30	73
			After 120 min	14	54.1	9.2	38	77
		After administration	After 30 min	14	59.2	10.5	39	81
			After 60 min	14	57.5	8.3	46	74
			After 90 min	14	60.5	5.8	52	72
			After 120 min	14	60.2	9.8	49	77

Table 14.3–9 Summary Statistics of Physical Examinations (Continued)

				n	Mean	SD	Min	Max
Pulse rate (beats/min)	Week 0	Before administration	Before administration	21	86.6	16.3	59	125
		During administration	After 30 min	21	82.3	19.2	53	133
			After 60 min	21	85.8	18.9	59	136
			After 90 min	21	85.0	19.0	59	136
			After 120 min	21	88.3	17.3	62	139
		After administration	After 30 min	21	85.7	13.9	62	109
			After 60 min	21	87.3	13.6	65	109
			After 90 min	21	86.9	12.6	66	107
			After 120 min	21	87.1	13.4	67	115
	Week 2	Before administration	Before administration	20	78.7	14.6	61	115
		During administration	After 30 min	20	78.7	13.0	60	111
			After 60 min	20	80.2	16.5	57	109
			After 90 min	20	79.3	15.9	54	113
			After 120 min	20	78.2	13.3	54	105
		After administration	After 30 min	20	82.5	11.6	63	101
			After 60 min	20	84.5	9.6	68	102
			After 90 min	20	87.2	10.6	65	106
			After 120 min	20	85.9	9.6	67	101
	Week 6	Before administration	Before administration	20	83.7	13.6	60	109
		During administration	After 30 min	20	80.6	14.5	60	103
			After 60 min	20	80.7	15.5	56	102
			After 90 min	20	80.2	17.2	52	115
			After 120 min	20	83.2	15.3	60	111
		After administration	After 30 min	20	83.0	14.7	63	109
			After 60 min	20	82.7	13.2	65	115
			After 90 min	20	81.3	12.5	64	108
			After 120 min	20	82.7	13.3	62	112
	Week 14	Before administration	Before administration	16	78.6	12.7	55	101
		During administration	After 30 min	16	76.2	12.5	54	92
			After 60 min	16	76.8	12.0	54	100
			After 90 min	16	77.0	13.3	56	98
			After 120 min	16	75.6	12.9	56	101
		After administration	After 30 min	16	79.6	12.7	60	100
			After 60 min	16	78.8	10.7	62	93
			After 90 min	16	79.3	13.8	54	102
			After 120 min	16	81.6	10.8	64	104
	Week 22	Before administration	Before administration	14	80.5	14.9	51	108
		During administration	After 30 min	14	78.9	13.4	59	108
			After 60 min	13	78.4	13.6	61	109
			After 90 min	13	78.8	11.9	63	102
			After 120 min	14	78.1	13.6	59	107
		After administration	After 30 min	14	80.6	11.7	63	104
			After 60 min	14	81.2	12.2	64	98
			After 90 min	14	81.3	10.3	61	98
			After 120 min	14	83.6	12.4	63	107

Table 14.3–9 Summary Statistics of Physical Examinations (Continued)

				n	Mean	SD	Min	Max
Body temperature (°C)	Week 0	Before administration	Before administration	21	36.55	0.64	35.7	38.1
		During administration	After 30 min	21	36.75	0.55	35.7	38.2
			After 60 min	21	36.98	0.75	36.2	39.5
			After 90 min	21	37.06	0.85	36.1	40.1
			After 120 min	21	37.06	0.79	35.8	39.3
		After administration	After 30 min	21	36.76	0.52	36.0	38.3
			After 60 min	21	36.83	0.54	35.8	38.3
			After 90 min	21	36.66	0.49	35.8	37.8
			After 120 min	21	36.70	0.55	35.2	37.9
	Week 2	Before administration	Before administration	20	36.57	0.49	35.5	37.2
		During administration	After 30 min	20	36.63	0.47	35.7	37.3
			After 60 min	20	36.58	0.42	35.5	37.2
			After 90 min	20	36.64	0.44	35.6	37.4
			After 120 min	20	36.73	0.42	35.7	37.4
		After administration	After 30 min	20	36.73	0.32	36.0	37.1
			After 60 min	20	36.80	0.26	36.2	37.2
			After 90 min	20	36.74	0.35	35.7	37.2
			After 120 min	20	36.65	0.40	35.9	37.2
	Week 6	Before administration	Before administration	20	36.61	0.38	35.6	37.3
		During administration	After 30 min	20	36.73	0.33	35.7	37.1
			After 60 min	20	36.80	0.22	36.5	37.3
			After 90 min	20	36.85	0.32	35.9	37.4
			After 120 min	20	36.72	0.40	35.6	37.4
		After administration	After 30 min	20	36.72	0.30	36.2	37.2
			After 60 min	20	36.75	0.31	36.0	37.3
			After 90 min	20	36.73	0.32	36.1	37.2
			After 120 min	20	36.72	0.40	35.8	37.3
	Week 14	Before administration	Before administration	16	36.45	0.37	35.8	37.2
		During administration	After 30 min	16	36.60	0.28	35.9	37.0
			After 60 min	16	36.73	0.30	36.2	37.2
			After 90 min	16	36.62	0.32	35.9	37.1
			After 120 min	16	36.64	0.36	35.6	37.1
		After administration	After 30 min	16	36.61	0.38	35.6	37.1
			After 60 min	16	36.56	0.47	35.7	37.2
			After 90 min	16	36.67	0.34	35.8	37.1
			After 120 min	16	36.57	0.37	35.9	37.1
	Week 22	Before administration	Before administration	14	36.57	0.44	35.6	37.4
		During administration	After 30 min	13	36.59	0.53	35.7	37.3
			After 60 min	12	36.67	0.59	35.1	37.4
			After 90 min	12	36.61	0.52	35.8	37.2
			After 120 min	13	36.65	0.34	36.1	37.2
		After administration	After 30 min	13	36.81	0.46	36.0	37.6
			After 60 min	13	36.72	0.54	35.4	37.4
			After 90 min	13	36.80	0.56	35.2	37.6
			After 120 min	13	36.75	0.42	35.6	37.2

15. Reference List

1) Turner D, Seow CH, Greenberg GR, Griffiths AM, Silverberg MS, Steinhart AH. A systematic prospective comparison of noninvasive disease activity indices in ulcerative colitis. Clin Gastroenterol Hepatol. 2009;7(10):1081-8. Epub 2009 Jul 1.

2) Written and edited by Toshiaki Tanaka. New Pocket Guide of Laboratory Test Reference Values for Children. Tokyo: Jiho; 2009.

3) Turner D, Otley AR, Mack D, Hyams J, de Bruijne J, Uusoue K, et al. Development, validation, and evaluation of a pediatric ulcerative colitis activity index: a prospective multicenter study. Gastroenterology. 2007;133(2):423-32.

16. Appendices

16.1 Study Information

16.1.1 Protocol and Protocol Amendments

16.1.1a Protocol (Ver. No.: 02.00.00000, Date of preparation: May 22, 2013)

16.1.1b Comparison table for protocol amendments

16.1.2 Sample Case Report Form

16.1.2 Case Report Form (Ver. No.: 01.00.00000, Date of preparation: December 20, 2011)

16.1.3 List of IRBs, Representative Written Information for Patient and Sample Consent Form

16.1.3a List of IRBs (confirmed date and name/title of members)

16.1.3b Final Version of Written Information and Consent Form (draft by sponsor)
(Ver.: 06.00.00000, Date of preparation: August 6, 2014)

16.1.3c List of Revisions in Written Information and Consent Form (draft by sponsor)

16.1.3d List of Written Information and Consent Form (for medical institution)

16.1.3e Final Version of Written Information and Assent Form (for patients aged 13 years or older, draft by sponsor)
(Ver. 05.00.00000, Date of preparation: August 6, 2014)

16.1.3f List of Revisions in Final Version of Written Information and Assent Form (for patients aged 13 years or older, draft by sponsor)

16.1.3g List of Written Information and Assent Form (for patients aged 13 years or older, for medical institution)

16.1.3h Final Version of Written Information and Assent Form (for patients aged 12 years or younger, draft by sponsor)
(Ver. 01.00.00000, Date of preparation: December 20, 2011)

16.1.3i List of Written Information and Assent Form (for patients aged 12 years or younger, for medical institution)

16.1.4 List and Description of Investigators and Other Important Participants in the Study

16.1.5 Signature of Sponsor's Responsible Medical Officer

16.1.6 Listing of Batch Numbers of Test Drug/Investigational Product Administered to Individual Patients

16.1.7 Randomized Scheme and Code (Patient Identification and Allocated Treatment)

Not applicable.

16.1.8 Documentation of Audit Procedures and Audit Certificates

16.1.9 Documentation of Statistical Methods

16.1.9a Statistical Analysis Plan (Ver. 3.0)

16.1.9b Statistical Analysis Report (Ver. 1.0)

16.1.9c Pharmacokinetic Analysis Plan (Ver. 1.0)

16.1.9d Pharmacokinetic Analysis Report (Ver. 1.0)

16.1.10 Documentation of Inter-Laboratory Standardization Methods and Quality Assurance Procedures

Not applicable.

16.1.11 Publication Based on the Study

Not applicable.

16.1.12 Important Publications Referenced in the Report

Not applicable.

16.1.13 Other Appendices

16.1.13a Procedures for Study Drug Management (Ver. 6.0)

16.1.13b Written Operating Procedures for Safety Evaluation Committee (Ver. 1.0)

16.1.13c Endoscopic Photography Atlas (Attachment 5)

16.1.13d Study Protocol
(Determination of serum infliximab concentration and antibodies to infliximab [ATI])

16.1.13e Final Report

(Determination of serum infliximab concentration and antibodies to infliximab [ATI])

16.1.13f Written Procedures for Monitoring

16.1.13g Minutes of Clinical Conference

16.1.13h Minutes of Face-to-face Consultation Meeting with PMDA

16.1.14 Documantation of Pharmacokinetics (if appricable)

16.2 Patient Data Listings

16.2.1 Patients Who Discontinued

16.2.2 Protocol Deviations

16.2.3 Patients Excluded from the Efficacy Analysis

See 16.1.13g.

16.2.4 Demographic Data

16.2.4a List of Patient Background 1

16.2.4b List of Patient Background 2

16.2.4c List of Patient Background 3

16.2.5 Compliance and/or Drug Concentration Data

See 16.2.8f and 16.1.13e

16.2.6 Individual Efficacy Response Data

16.2.6a List of Efficacy by Patient 1 (CAI score) (TF) (FAS)

16.2.6b List of Efficacy by Patient 1 (CAI subscore) (LOCF) (FAS)

16.2.6c List of Efficacy by Patient 2 (Partial Mayo score/Mayo score) (TF) (FAS)

16.2.6d List of Efficacy by Patient 2 (Partial Mayo subscore/Mayo subscore) (LOCF) (FAS)

16.2.6e List of Efficacy by Patient 3 (PUCAI score) (TF) (FAS)

16.2.6f List of Efficacy by Patient 3 (PUCAI subscore) (LOCF) (FAS)

16.2.6g List of Efficacy by Patient 4 (Steroid dose) (FAS)

16.2.7 Listing of Adverse Events by Patient

16.2.7a List of Adverse Events by Patient (Safety analysis set)

16.2.7b List of Serious Adverse Events by Patient (Safety analysis set)

16.2.8 List of Clinical Laboratory Test Values by Patient

16.2.8a List of Hematology Data by Patient (Safety analysis set)

16.2.8b List of Blood Biochemistry Data by Patient 1 (Safety analysis set)

16.2.8c List of Blood Biochemistry Data by Patient 2 (Safety analysis set)

16.2.8d List of Urinalysis (Qualitative) Data by Patient (Safety analysis set)

16.2.8e List of Immunoserological Tests Values by Patient (Safety analysis set)

16.2.8f List of Physical Examination (Blood Pressure, Pulse Rate, Body Temperature) Data by Patient (Safety analysis set)

16.2.8g List of Other Clinical Laboratory Test Values by Patient (CRP) (FAS)

16.2.8h List of Height and Weight Data by Patient (FAS)

16.3 Case Report Forms

16.3.1 CRFs for Deaths, Other Serious Adverse Events and Withdrawals for AE

Not applicable.

16.3.2 Other CRFs Submitted

Not applicable.
