# Supplementary material for: Correction: Infliximab for pediatric patients with Crohn’s disease: A Phase 3, open-label, uncontrolled, multicenter trial in Japan
Source: PLoS One. 2022 Apr 19;17(4):e0261932. doi: 10.1371/journal.pone.0261932 (PMC9017882; doi:10.1371/journal.pone.0261932)
Supplement: S1 File — (PDF) [file pone.0261932.s001.pdf]

Clinical study of TA-650 in patients with pediatric Crohn’s disease

Protocol

Sponsor
Mitsubishi Tanabe Pharma Corporation

Protocol number: TA-650-20
Version: 01.00.00000
Creation Date: November 21, 2011

Protocol summary

1. Study title

Clinical study of TA-650 in pediatric patients with Crohn’s disease

2. Objective

To evaluate the efficacy of TA-650 using PCDAI and other evaluation indicators in pediatric patients with moderate to severe Crohn’s disease after TA-650 administration at a dose of 5 mg/kg at weeks 0, 2, and 6, then every 8 weeks after week 14 up to week 46, and at a dose of 10 mg/kg if the effect is attenuated. The safety and pharmacokinetics are also evaluated.

3. Study patients

3.1 Study patients

Pediatric patients with moderate to severe Crohn’s disease

3.2 Inclusion criteria

Patients who meet all the following inclusion criteria at the time of the enrollment (regardless of inpatients or outpatients) are eligible for this study.

1) Indication studied: Crohn’s disease
Patients who have been diagnosed at least 3 months before the first day of the screening period according to the “Draft criteria for the diagnosis of Crohn’s disease” (revised on February 9, 2011) of the Inflammatory Bowel Disease Research Committee of the Ministry of Health, Labour and Welfare (Attachment 1).
2) Patients between 6 and 17 years of age (at the time of obtaining informed consent).
3) Patients who gave oral or written assent and whose legal representatives gave written consent to the patients’ participation to the clinical study (obtain written assent from patients of approximately junior high school age and older while it depends on patients’ ability to understand; also, obtain written assent as far as possible from patients under approximately junior high school age).
4) Patients with PCDAI of over 30 in the screening period due to insufficient effects of conventional therapy (appropriate therapy such as nutrition therapy or drug therapy), regardless of the presence or absence of external fistula.
5) Patients treated with nutrition therapy or drug therapy or both that meet the following criteria on the first day of the screening period:
   (1) Enteral nutrition (elemental diet and polymeric diet) (including total enteral nutrition)
   Patients who have been treated at a constant dose for at least 2 weeks before the first day of the screening period; or patients who have not been treated for at least 2 weeks before the first day of the screening period if not treated on the first day of the screening period.
   (Total enteral nutrition is defined as complete enteral nutrition [elemental diet and

polymeric diet] prescribed by the investigator, without taking any ordinary meals, low residue meals or a semidigest diet.)

(2) Azathioprine, 6-mercaptopurine, methotrexate
Patients who have been treated for at least 16 weeks before the first day of the screening period, and have been treated at a constant dose for at least 8 weeks before the first day of the screening period; or patients who have not been treated for at least 8 weeks before the first day of the screening period if not treated on the first day of the screening period.

(3) Steroids (oral drugs, suppositories, or enemas)
Patients who have been treated for at least 8 weeks before the first day of the screening period, and have been treated at a constant dose for at least 2 weeks before the first day of the screening period; or patients who have not been treated for at least 2 weeks before the first day of the screening period if not treated on the first day of the screening period.

(4) 5-Aminosalicylates (mesalazine and salazosulfapyridine)
Patients who have been treated for at least 8 weeks before the first day of the screening period, and have been treated at a constant dose for at least 4 weeks before the first day of the screening period; or patients who have not been treated for at least 4 weeks before the first day of the screening period if not treated on the first day of the screening period.

(5) Metronidazole, ciprofloxacin
Patients who have been treated at a constant dose for at least 2 weeks before the first day of the screening period; or patients who have not been treated for at least 2 weeks before the first day of the screening period if not treated on the first day of the screening period.

3.3 Exclusion criteria

Patients who meet any one of the following exclusion criteria are ineligible for this study.

1) Patients with severe intestinal strictures (strictures which may affect the number of defecations, etc, or dilation of the colon or strictures in the proximal small bowel observed on barium radiograph, or strictures precluding the insertion of endoscope), a diagnosis of short bowel syndrome, or previous stoma surgery.
2) Patients who have a history of treatment with infliximab, or biologics (anti-TNFα agents and anti-IL-6 agents, etc).
3) Patients who are currently or have been treated with total parenteral nutrition (TPN) within 4 weeks before the first day of the screening period.
(Total parenteral nutrition is defined as the administration of nutrition fluid containing carbohydrates, amino acids and electrolytes via a central vein to provide total nutrition exclusively.)
4) Patients who are currently or have been treated with fasting therapy within 4 weeks before the first day of the screening period.
(Fasting therapy is prescribed by the investigator or sub-investigator for the treatment of Crohn’s disease. However, it does not include fasting during total enteral nutrition.)
5) Patients who are currently or have been treated with immunomodulators (excluding

azathioprine, 6-mercaptopurine and methotrexate) such as cyclosporine, tacrolimus (excluding external preparations), mycophenolate mofetil, and mizoribine within 4 weeks before the first day of the screening period.

6) Patients who are currently or have been treated with steroids (injections) within 4 weeks before the first day of the screening period.
7) Patients who have been treated with laxatives or enemas or both, antidiarrheals (such as Lopemin), or opiates within 3 days before the first day of the screening period.
8) Patients who are currently or have been treated with leukocytapheresis within 3 months before the first day of the screening period.
9) Patients who have undergone surgery (requiring attention to post-operative infection) within 4 weeks before the first day of the screening period.
10) Patients with abscesses. Patients who underwent the drainage of an abscess are allowed to participate if drainage had been completed at least 4 weeks before the enrollment and if the abscess has disappeared at the time of the enrollment.
11) Patients with significant internal fistula (which may require surgery, etc).
12) Infections:
    (1) Patients with a complication, or a history within 6 months before the enrollment of, serious infections requiring hospitalization (such as active hepatitis, pneumonia, or pyelonephritis).
    (2) Patients with a complication, or a history within 6 months before the enrollment of, opportunistic infections (such as cytomegalovirus infection, systemic fungal infection, Pneumocystis pneumonia, and nontuberculous mycobacterial infection).
    (3) Patients with a complication of active tuberculosis.
    (4) Patients with a history of or suspected tuberculosis infection. However, patients who have undergone an antitubercular drug (isoniazid [INH] in principle) for 3 weeks or longer before the first study drug administrationmay be included in the study.
    (Patients are suspected to have tuberculosis when they meet any one of the following criteria)
        • Imaging findings meet the criteria for obsolete pulmonary tuberculosis (including pleural adhesion and calcification alone).
        • Physical contact with patients with active tuberculosis.
        • Positive QuantiFERON (QFT) result.
        For patients with unclear QFT results or positive tuberculin test results (when tuberculosis infection is strongly suspected taking the effect of BCG inoculation into consideration), the administration of an antitubercular drug is not necessary when the investigator or sub-investigator judges that suspected tuberculosis infection can be denied based on other tuberculosis screenings (such as chest X-ray, chest CT, inspection, etc).
        The specialist may be consulted for evaluation of results from imaging, etc.
    (5) Patients with a complication of active hepatitis B or C, or patients confirmed to be hepatitis B virus carriers.
    (6) Patients confirmed to have HIV infection.
    (7) Patients with a complication of other chronic infections (such as chronic renal infection, chronic respiratory infection with bronchiectasis, and chronic sinusitis).

13) Patients with a history of hypersensitivity to proteins of mouse origin (such as mouse antibodies, chimeric antibodies, and humanized antibodies).
14) Patients with a complication or a history of demyelinating diseases (such as multiple sclerosis).
15) Patients with a complication of congestive heart failure.
16) Patients with a complication or a history of lymphoproliferative disorders including lymphoma, signs of lymphoproliferative disorders such as lymphadenopathy of untypical size or location, or clinically relevant hepatomegaly or splenomegaly.
17) Patients with a family history of lymphoma or leukemia.
18) Patients with a complication, or a history within 5 years before the enrollment of, a malignant tumor.
19) Patients who show any one of the following 10 laboratory test results at the time of the enrollment. The laboratory test must be conducted after consent and within 2 weeks before the enrollment.
    (1) Hemoglobin level: < 8.5 g/dL
    (2) White blood cell count: < 3,500 × 10^6/L
    (3) Neutrophil count: < 1,500 × 10^6/L (when stab and segmented cells are counted, the value should be combined)
    (4) Lymphocyte count: < 500 × 10^6/L
    (5) Platelet count: < 10 × 10^4/μL
    (6) AST (GOT): > twice the upper limit of the reference range
    (7) ALT (GPT): > twice the upper limit of the reference range
    (8) ALP: > twice the upper limit of the reference range
    (9) HBs antigen, HBs antibody, HBc antibody: positive
    (10) HIV antibody: positive
20) Patients who have any signs of lupus-like syndrome and an anti-dsDNA antibody level exceeding the normal range of the contracted laboratory testing facility.
21) Patients with a complication of serious diseases (refer to Grade 3 in the Standards for Classification of Serious Adverse Drug Reactions [Attachment 2]).
22) Patients who have undergone inoculation with a live vaccine within 3 months before the first study drug administration.
23) Patients from whom continuous blood collection is difficult during the study period.
24) Patients who will not use contraception during the study period and for 6 months after the final study drug administration.
25) Female patients who are pregnant, breast-feeding, or may be pregnant.
26) Patients who had participated in another clinical study and had received an investigational drug within 12 weeks before giving consent.
27) Patients otherwise judged by the investigator or sub-investigator to be ineligible to participate in the clinical study.

3.4 Discontinuation criteria

Patients who meet any one of the following criteria are withdrawn from the study.

1) When a legal representative or a patient requests discontinuation.
2) When the investigator or sub-investigator judges that it is difficult for the patient to continue the clinical study due to deterioration of symptoms of Crohn’s disease (such as when surgery is conducted for the treatment of

Crohn’s disease).

3) When the investigator or sub-investigator judges that it is difficult for the patient to continue the clinical study due to the occurrence of adverse events (AEs), etc.

   Study participation is also discontinued if a patient develops symptoms suggestive of lupus-like syndrome and becomes anti-dsDNA antibody positive (anti-dsDNA IgM antibody level exceeding 6 U/mL, or anti-dsDNA IgG antibody level exceeding 12 IU/mL).

4) When it becomes apparent that a patient is ineligible for participation in the study from the view of safety assurance or efficacy evaluation after the study has started.

5) Otherwise, when the investigator or sub-investigator judges that participation of the patient in the clinical study should be discontinued.

4. Study design

Open-label, non-comparative, multi-center clinical study

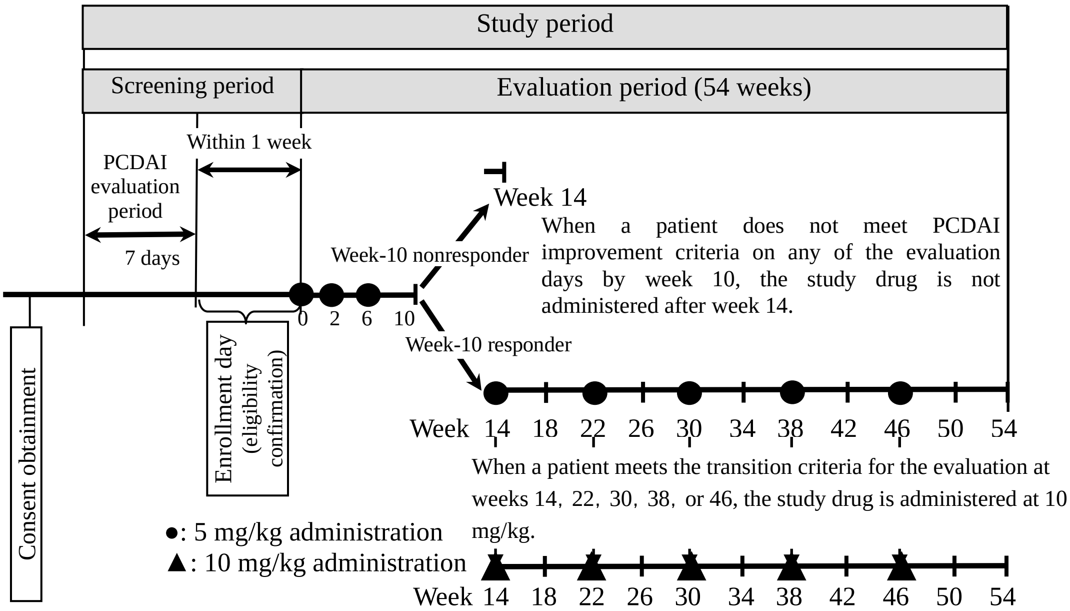

Study period:	The period from the first day of the screening period to the last day of the evaluation period.
Screening period:	The period from the first day of PCDAI evaluation to the first study drug administration.
Evaluation period:	The period from the first study drug administration to the evaluation day at week 54. However, the period is until evaluation at week 14 for week-10 nonresponders, and until 8 weeks after the final study drug administration for patients whose study participation has been discontinued.

5. Study drug, dose and dose regimen

5.1 Name of study drug

Name: TA-650
Nonproprietary name: Infliximab (recombinant)
Dosage form and content: Lyophilized, containing 100 mg as infliximab in a vial

5.2 Dose and dose regimen

TA-650 will be intravenously infused at 5 mg per kg body weight on each study drug administration day as a single dose slowly over at least 2 hours.

To patients who are administered at the first study drug administration (week 0), and weeks 2 and 6 and who meet the criteria of PCDAI improvement[*1)] at least once at the evaluations at weeks 2, 6 and 10 (week-10 responders), TA-650 is administered at 8-week intervals thereafter until week 46. In addition, patients who meet the transition criteria[*2)] on the evaluation days at weeks 14, 22, 30, 38, or 46 receive

TA-650 at 10 mg per kg body weight, and similarly on the subsequent days of study drug administration. On the other hand, to patients who are administered at the first study drug administration (week 0), and weeks 2 and 6 and do not meet the criteria of PCDAI improvement at any of the evaluation time points at weeks 2, 6 and 10 (week-10 nonresponders), TA-650 is not administered from week 14 onward.

*1) PCDAI improvement: When PCDAI on the evaluation day is reduced by at least 15 points compared to that at the time of the enrollment, and it does not exceed 30 points.

*2) Transition criteria: The transition criteria for dose increase to 10 mg/kg are defined as when patients meet one of the two items below from week 14 onward:

1. When PCDAI on the evaluation day is increased by at least 15 points compared to the lowest PCDAI observed at weeks 2, 6 and 10 of study drug administration.
2. When PCDAI on the evaluation day exceeds 30 points.

5.3 Administration period

Forty-six weeks (8 doses at weeks 0, 2, 6, 14, 22, 30, 38 and 46); or 6 weeks (3 doses at weeks 0, 2 and 6) for week-10 nonresponders

6 Concomitant medications and therapies

6.1 Prohibited concomitant medications and therapies

The following drugs and therapies are prohibited in the screening period and the period until the evaluation at week 54 is conducted. For week-10 nonresponders, the drugs and therapies shown below are prohibited until evaluation at week 14. Regarding a patient whose study participation has been discontinued, items 1) to 8) are prohibited until evaluation on the day of discontinuation, and items 9) and 10) are prohibited until the evaluation 8 weeks after the final study drug administration.

1) Remicade® and biologic agents (anti-TNFα agents and anti-IL-6 agents, etc)

2) Total parenteral nutrition
(Total parenteral nutrition is defined as the administration of nutrition fluid containing carbohydrates, amino acids and electrolytes via a central vein to provide total nutrition exclusively.)

3) Fasting
(Fasting prescribed by the investigator [sub-investigator] for the treatment of Crohn’s disease. However, it does not include fasting during total enteral nutrition.)

4) Surgical procedures
   (1) Surgical procedures for the treatment of Crohn’s disease (drainage is not regarded as surgery)
   (2) Operations requiring attention to post-operative infection

5) Immunomodulators such as cyclosporine, tacrolimus (excluding the external preparations), mycophenolate mofetil, and mizoribines (however, azathioprine, 6-mercaptopurine, methotrexate are excluded.)

6) Leukocytapheresis
7) Blood transfusion
8) Steroids (injections)
The use of steroids is permitted as treatment or pretreatment for AEs such as infusion-related reactions. However, the pretreatment should be conducted after the efficacy evaluation.
9) Live vaccines
10) Other investigational drugs than TA-650

6.2 Restricted concomitant medications or therapies

The concomitant use of other medications or therapies than those stipulated in the above-mentioned section "6.1 Prohibited concomitant drugs and therapies" are permissible during the study period. However, drugs for the treatment of Crohn's disease, laxatives or enemas or both, antidiarrheals (such as Lopemin), opiates, or enteral nutrition may only be used under the following conditions:

1) Medications for the treatment of Crohn's disease
The following medications for the treatment of Crohn's disease should be administered at a constant dose during the screening period and the period until the evaluation at week 54, and the start of new treatment or an increase in the dose is not allowed. For week-10 nonresponders and for patients whose study participation is discontinued, the medications should be administered at a constant dose until evaluation at week 14 and until evaluation on the discontinuation day, respectively, and the start of new treatment is not allowed. However, the dose of azathioprine, 6-mercaptopurine, methotrexate, steroids (oral drugs, suppositories, and enemas), metronidazole, and ciprofloxacin can be changed after the first administration of the study drug within a range not exceeding the dose in the screening period.

- Azathioprine, 6-mercaptopurine, and methotrexate
- Steroids (oral drugs, suppositories, and enemas)
- Aminosalicylates (mesalazine and salazosulfapyridine)
- Metronidazole and ciprofloxacin

2) Laxatives or enemas or both, antidiarrheals (such as Lopemin), or opiates
These can be used concomitantly throughout the study period; however, they are prohibited from 3 days before the start day of PCDAI evaluation to the PCDAI evaluation day.
3) Enteral nutrition (including total enteral nutrition)
Enteral nutrition should be administered at a constant dose during the screening period and the period until the evaluation at week 54. For week-10 nonresponders and for patients whose study participation is discontinued, it should be administered at a constant dose until evaluation at week 14 and until evaluation on the discontinuation day, respectively, and the start of new treatment is not allowed. However, after the first administration of the study drug, a dose may be changed according to improvement in the clinical symptoms of Crohn's disease.

[Total enteral nutrition is defined as complete enteral nutrition (elemental diet, polymeric diet) without any ordinary meals, low residue meals or a semidigest diet prescribed by the investigator.]

6.3 Precautions of other allowed concomitant medications

1) Vaccination
Inoculation with a live vaccine immediately after TA-650 administration may pose a risk of secondary infection. Therefore, patients who have received live vaccines within 3 months before TA-650 administration are excluded, and inoculation with a live vaccine during the evaluation period is prohibited. In addition, for the patients who receive inoculation with an inactivated vaccine during the TA-650 administration period, a possible influence on vaccination should be taken into consideration. Based on these, check the history of regular or voluntary vaccination, as well as the history of infections that are preventable by vaccination of the patient to examine the need for vaccination.

2) Antitubercular drugs
To patients who have a history of or suspected tuberculosis, INH is administered for a period of 6 to 9 months (9 months as far as possible) from at least 3 weeks prior to the first study drug administration. The dose of 10 to 15 mg/kg is used as a guide, and the maximum dose is 300 mg per day. If the administration of INH is discontinued for reasons including adverse effects within 3 weeks before the first administration of the study drug, the study drug will not be administered. If the administration of INH becomes impossible for reasons including adverse effects after the first administration of the study drug, a specialist must be consulted.

(Patients are suspected to have tuberculosis when they meet any one of the following criteria)

- Imaging findings meet the criteria for obsolete pulmonary tuberculosis (including pleural adhesion and calcification alone).
- Physical contact with patients with active tuberculosis.
- Positive QuantiFERON (QFT) result.

For patients with unclear QFT results or positive tuberculin test results (when tuberculosis infection is strongly suspected taking the effect of BCG inoculation into consideration), the administration of an antitubercular drug is not necessary when the investigator or sub-investigator judges that suspected tuberculosis infection can be denied based on other tuberculosis screenings (such as chest X-ray, chest CT and inspection).

The specialist may be consulted for evaluation of results from imaging.

7 Evaluation endpoints

7.1 Efficacy endpoints

1) PCDAI
2) PCDAI change
3) PCDAI clinical response rate
4) PCDAI clinical remission rate
5) Corticosteroid dosage
6) CRP
7) CRP change
8) Number of draining fistulas

7.2 Safety evaluation

AEs and side effects

7.3 Pharmacokinetic evaluation

1) Serum infliximab concentration (serum TA-650 concentration)
2) Anti-infliximab antibody (antibody to TA-650)

8 Number of patients

Twenty patients

9 Study period

November 2011 to July 2014

10 Screening and evaluation schedule

		Day of informed consent	Screening period	Evaluation period (54 weeks)															Week-10 NR	Discontinued participation	
				Week 0	Week 2	Week 6	Week 10	Week 14	Week 18	Week 22	Week 26	Week 30	Week 34	Week 38	Week 42	Week 46	Week 50	Week 54	Week 14	At discontinuation	8 weeks after the final study drug administration
Permitted range of evaluation (days)		-		-	±3	±3	±7	±7	±7	±7	±7	±7	±7	±7	±7	±7	±7	±7	±7	-	±7
Obtainment of written consent		●																			
Screening items conducted at the enrollment*1			●																		
Patient demographics examination			●																		
Permitted range of study drug administration (days)					±3	±3		±7		±7		±7		±7		±7					
Study drug administration				●	●	●		●		●		●		●		●					
Height and weight				●	●	●	●	●	●	●	●	●	●	●	●	●	●	●	●	●	
Efficacy	PCDAI*2		●		●	●	●	●	●	●	●	●	●	●	●	●	●	●	●	●	
Efficacy	CRP			●	●	●	●	●	●	●	●	●	●	●	●	●	●	●	●	●	
Efficacy	Number of draining fistulas*3		●				●					●						●		●	
Safety	Sign and symptoms			←	─	─	─	─	─	─	─	─	─	─	─	─	─	─	─	─	→
Safety	Blood pressure, heart rate, body temperature*4			●	●	●		●		●		●		●		●					
Safety	General laboratory tests*5			●	●	●	●	●	●	●	●	●	●	●	●	●	●	●	●	●	●
Safety	Immunoserology*6			●				●				●				●		●	●	●	●
Chest X-ray, Chest CT (as needed)			●					●				●						●	●	●	
Other laboratory test items*7				•																	
Pharmacokinetics	Serum concentration of infliximab*8			◎	●	●	●	○	●	◎	●	○	●	○	●	◎	●	●	●	●	●
Pharmacokinetics	Anti-infliximab antibody*9			●				●		●		△		△		●		●	●	●	●

NR: nonresponder

◎: Blood will be collected twice in total; once before TA-650 administration and once at 1 hour after completion of study drug administration.

○: Blood will be collected before TA-650 administration. However, if the patient meets the transition criteria (see P.6) on any evaluation days at weeks 14, 30 or 38, blood will be collected 1 hour after completion of the TA-650 administration (10 mg/kg) at the relevant evaluation time point.

△: Evaluate the anti-infliximab antibodies at the evaluation time point if the transition criteria (see P.6) are met on one of the evaluation days at weeks 30 or 38.

<u>On the days of study drug administration, evaluation and testing are conducted before study drug administration.</u>

***1 Screening items conducted at the enrollment:**

Crohn's disease activity	PCDAI
Laboratory tests	(Examine the following items between after the consent obtainment and within 2 weeks before the enrollment)<br>Hemoglobin, white blood cell count, neutrophil count, lymphocyte count, platelet count, AST (GOT), ALT (GPT), ALP, HBs antigen, HBs antibody, HBc antibody, HIV antibody, and pregnancy test (only females judged as physiologically capable of becoming pregnant)
Tuberculosis test	(Examine the following items between after the consent obtainment and within 4 weeks before the enrollment)<br>Interview, chest X-ray, chest CT (as needed), tuberculin test or QFT test
Vaccination history<br>Infection history	(Examine the following items before the enrollment)<br>Vaccination history and infection history
• Study drug administration is started within 1 week after the PCDAI evaluation day during the screening period. If the interval from the PCDAI evaluation day in the screening period to the first study drug administration is 1 week or longer, one more PCDAI evaluation is conducted for the enrollment.<br>• When there are multiple test values, use the one closest to the enrollment date, except for test values intensively measured at the first study drug administration.<br>• If there are multiple chest X-ray, chest CT, tuberculin test or QFT test results after the consent obtainment, use the result closest to the enrollment date.<br>• It is required to conduct either the tuberculin test or the QFT test. Additionally, tuberculin test results should be judged taking the effect of BCG inoculation into consideration.	

Neutrophil count: When stab and segmented cells are counted, the value should be combined.

***2 Test and observation items required for PCDAI calculation (efficacy evaluation):**

Laboratory tests and other tests	• Laboratory tests (hematocrit, ESR, and albumin)<br>Laboratory tests are performed during the period from 7 days before the PCDAI evaluation day to the evaluation day.<br>• Other tests (height and weight)<br>Other tests are performed on the PCDAI evaluation day.
Clinical symptoms (observation and interview)	• Abdominal pain, stools (per day), general status, and fever<br>Patients are asked at the interview about symptoms within 7 days before the PCDAI evaluation day.<br>• Physical findings of abdominal and rectal lesions and extraintestinal symptoms<br>Physical findings are observed on the PCDAI evaluation day.
• As for hematocrit, ESR, and albumin, use values measured in-hospital during the period from 7 days before the PCDAI evaluation day to the evaluation day; if multiple measurements are conducted, use the measured value closest to the PCDAI evaluation day.<br>• On the days of study drug administration, testing, observation and interview should be conducted before study drug administration.	

***3 Number of draining fistulas:** The number of draining fistulas, that is, skin fistulas

or anal fistulas for which drainage is observed when it is lightly pressed.

***4 Blood pressure, heart rate, body temperature**: The parameters are measured immediately before the first study drug administration, at every 30 minutes during study drug administration, and at every 30 minutes from the completion of study drug administration to 2 hours after the completion of study drug administration. If the interval between the completion of study drug administration and the measurement immediately before the completion of study drug administration is 15 minutes or longer, measure at the completion of study drug administration, too.

***5 General Laboratory tests**: Measured by a contracted laboratory testing facility

Hematology	Red blood cell count, hemoglobin, hematocrit, white blood cell count, differential white blood count (neutrophils, eosinophils, basophils, monocytes, and lymphocytes), and platelet count
Blood biochemistry	AST(GOT), ALT(GPT), ALP, LDH, γ-GTP, total protein, albumin, total cholesterol, total bilirubin, BUN, serum creatinine, and serum electrolyte (Na, K, and Cl)
Urinalysis (qualitative)	Protein, glucose, occult blood, and urobilinogen

***6 Immunoselorogy:** Anti-nuclear antibody and anti-dsDNA antibodies (anti-dsDNA IgG antibody and anti-dsDNA IgM antibody) are measured intensively at the contracted laboratory testing facility.

***7 Other laboratory test items**: IL-6, TNFα, transferrin, prealbumin, and retinol-binding protein are measured intensively at the contracted laboratory testing facility.

***8 Serum infliximab concentration**: On the days of study drug administration, blood will be collected before the study drug administration. At the first study drug administration (week 0) and weeks 22 and 46, blood will be collected twice in total, that is, once before the study drug administration and once 1 hour after the completion of study drug administration. However, if patients meet the transition criteria on any evaluation days at weeks 14, 30 or 38, blood will be collected 1 hour after the completion of TA-650 administration (10 mg/kg) at the relevant evaluation time point.

***9 Anti-infliximab antibody**: On the days of study drug administration, blood will be collected before the study drug administration. Also, if patients meet the transition criteria (see P.6) on any evaluation days of weeks 30 and 38, blood will be collected at the relevant evaluation time point.
